# Colon cancer associated genes exhibit signatures of positive selection at functionally significant positions

**DOI:** 10.1186/1471-2148-12-114

**Published:** 2012-07-12

**Authors:** Claire C Morgan, Kabita Shakya, Andrew Webb, Thomas A Walsh, Mark Lynch, Christine E Loscher, Heather J Ruskin, Mary J O’Connell

**Affiliations:** 1 Bioinformatics and Molecular Evolution Group, School of Biotechnology, Dublin City University, Glasnevin, Dublin 9, Ireland; 2 Centre for Scientific Computing & Complex Systems Modelling (SCI-SYM), Dublin City University, Glasnevin, Dublin 9, Ireland; 3 School of Computing, Dublin City University, Glasnevin, Dublin 9, Ireland; 4 Immunomodulatory Research Group, School of Biotechnology, Dublin City University, Glasnevin, Dublin 9, Ireland

**Keywords:** Positive selection, Colon cancer, Adaptive evolution, Protein functional shift, Selective pressure, Evolutionary medicine

## Abstract

**Background:**

Cancer, much like most human disease, is routinely studied by utilizing model organisms. Of these model organisms, mice are often dominant. However, our assumptions of functional equivalence fail to consider the opportunity for divergence conferred by ~180 Million Years (MY) of independent evolution between these species. For a given set of human disease related genes, it is therefore important to determine if functional equivalency has been retained between species. In this study we test the hypothesis that cancer associated genes have different patterns of substitution akin to adaptive evolution in different mammal lineages.

**Results:**

Our analysis of the current literature and colon cancer databases identified 22 genes exhibiting colon cancer associated germline mutations. We identified orthologs for these 22 genes across a set of high coverage (>6X) vertebrate genomes. Analysis of these orthologous datasets revealed significant levels of positive selection. Evidence of lineage-specific positive selection was identified in 14 genes in both ancestral and extant lineages. Lineage-specific positive selection was detected in the ancestral Euarchontoglires and Hominidae lineages for STK11, in the ancestral primate lineage for CDH1, in the ancestral Murinae lineage for both SDHC and MSH6 genes and the ancestral Muridae lineage for TSC1.

**Conclusion:**

Identifying positive selection in the Primate, Hominidae, Muridae and Murinae lineages suggests an ancestral functional shift in these genes between the rodent and primate lineages. Analyses such as this, combining evolutionary theory and predictions - along with medically relevant data, can thus provide us with important clues for modeling human diseases.

## Background

Mouse models are currently used to research many human cancers including colon cancer. On a genome wide scale, mouse protein sequences share 78.5% sequence identity with human counterparts [1]. With such high levels of sequence identity it may seem reasonable to expect that many orthologs between mouse and human would have conserved functions. However, in the ~180 Million Years (MY) of independent evolution [2], it is possible that certain proteins have functionally diverged. One example of ortholog divergence between human and mouse is the *TDP1* gene, required in Topo1-DNA complex repair, protein sequence similarity of 81%. A point mutation from an adenine to a guanine at position 1478 in human *TDP1* is linked with a disorder known as SCAN1 that results in cerebellar atrophy and peripheral neuropathy. However, this mutation in mouse does not result in the same condition/phenotype [3]. Specific mutations in any of the following genes in human result in disease: BCL10, PKLR and SGCA, but the same mutations in the mouse homologs do not result in phenotypic change to a disease state [4]. BRCA1 is heavily implicated in breast cancer in humans, with BRCA1^+/−^ women having a 50% risk of developing breast cancer, while BRCA^+/−^ mice do not exhibit increased susceptibility to cancer [5]. These observed differences in phenotype could potentially be the result of protein functional shifts in cancer-associated genes between human and mouse. While the analysis of the mouse lineage versus human is important from an evolutionary medicine perspective to determine/predict those specific cases where mouse may not effectively model the human disease phenotype, the analysis of all other lineages frames these results in the context of all mammals. Therefore, in this study we have not only examined the human and mouse lineages but all lineages leading to extant species in our dataset. This allows us to gain a greater understanding of the level of lineage-specific functional shift that has occurred in colon cancer associated genes.

Positive selection is the retention and spread of advantageous mutations throughout a population and has long been considered synonymous with protein functional shift. There are a number of driving forces for positive selection including external mechanisms such as adaptation to different ecological niches and response to disease and internal mechanisms such as co-evolution and compensatory mutations [6], all of which are relevant to the data and species we are analyzing. At the molecular level, the ratio of nonsynonymous substitutions per nonsynonymous site (*dN*) to synonymous substitutions per synonymous site (*dS*) is known as ω, and indicates the selective pressure at work in that sequence. If ω > 1 it signifies positive selective pressure, ω = 1 signifies neutral evolution, while ω < 1 indicates purifying selective pressure. Previous work assessed the level of positive selection present in mammal genomes and estimated 5%-9% of genes in mammals are under positive selection under a Bayesian framework, and thus provides us with a reference or expected level of positive selection for our analysis [7,8].

Here we have applied a Maximum Likelihood method based on codon models of evolution to assess the selective pressures across our dataset [9]. These methods are far more robust than alternatives such as the sliding window approach [10], nonetheless they do suffer from limitations and have strict criteria in terms of dataset size for statistical robustness [11,12]. Another feature of sequence evolution that can negatively impact on a selective pressure analysis is recombination [13]. To evaluate the robustness of the Likelihood Ratio Tests (LRTs), simulations have been performed that show that type 1 error rates can be up to 90% with relatively high rates of recombination in protein coding sequences resulting in the misinterpretation of recombination as positive selection [13]. We have incorporated a test for recombination for all genes in the dataset prior to the ML selective pressure analysis. Recent studies using these codon models of evoluton in an ML framework have combined evolutionary predictions of positive selection with biochemical verification of functional affects of these substitutions [14-16], and thus support the link between positive selection and protein functional shift.

We have taken colon cancer as an example for our study given the large amount of mutation and epigenetic data available for this form of cancer [17]. Lineage-specific positive selection in genes associated with colon cancer is strongly suggestive of functional shift and could have serious implications in the use of certain lineages for modeling colon cancer.

Colorectal cancer (CRC) is the third most commonly diagnosed cancer in males and second in females and we have focused on this in our study [18]. CRC arises through the accumulation of multiple genetic and epigenetic changes. Genetic changes consist of both somatic and germline (i.e. heritable) mutations. The genes in which there are germline mutations that are highly associated with the development of colon cancer are analyzed here (22 genes in total) and are referred to throughout this manuscript as “colon cancer associated genes”. Colon cancer associated genes work in conjunction with other proteins and pathways and can be thought of as contributing to, rather than being the single cause of colon cancer (note: these genes also have other functions outside of their association that may contribute to selective pressure variation in different lineages). Epigenetic changes such as hypermethylation of certain genes, loss of imprinting and acetylation/phosphorylation/methylation of particular histones are also implicated in cancer. Detailed information on colon cancer epigenetics have been made available to the community through the StatEpigen biomedical resource [17]. Other events such as loss of heterozygosity, microsatellite instability and CpG island methylator phenotype can also play an important role.

Hereditary Non-Polyposis Colorectal Cancer (HNPCC) is a hereditary predisposition for the development of colorectal cancer, and accounts for 3% of all colon cancer cases [19]. The 22 genes we have analyzed were selected based on the presence of known germline mutations associated with colon cancer. What follows is a brief description of each gene in the study. The genes linked with HNPCC are: MLH1, PMS2, MSH2, MSH6, and PMS1, all of which are members of the MMR DNA repair pathway [19].

**Figure 1:**
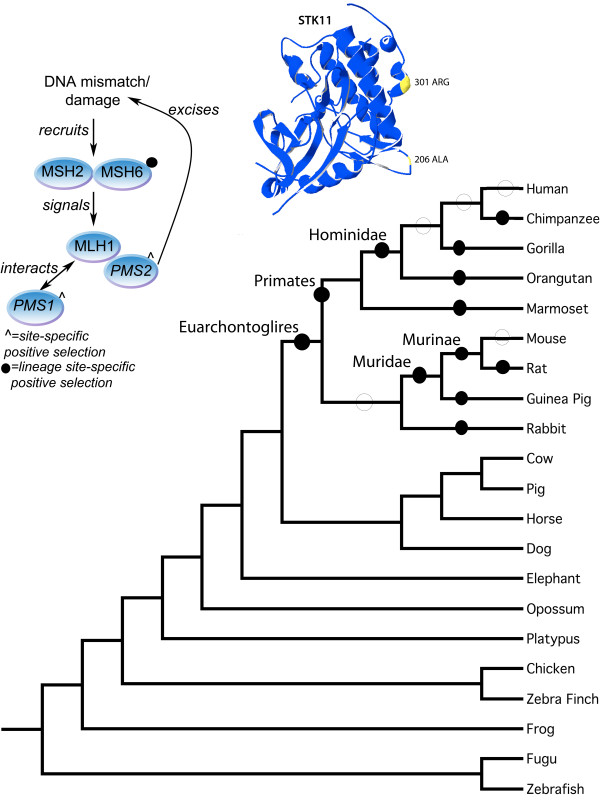
**Phylogeny of animal species used in this study.** The ancestral lineages tested in the analysis are labeled with their corresponding names as used throughout the text. Those lineages where positive selection was detected are labeled with filled circles, no evidence of positive selection is denoted with an empty circle.

MLH1 (mutL homologue 1) is a mismatch repair gene and is commonly associated with HNPCC. Missense mutations in MLH1 occur in the C-terminal domain, which is responsible for constitutive dimerization with the mismatch repair endonuclease PMS2 [20]. Studies have also shown that microsatellite instability (MSI) is the molecular fingerprint of a deficient mismatch repair system. It is estimated that some 15% of colorectal cancers display MSI owing to the epigenetic silencing of MLH1, and/or germline mutation in any one of the following mismatch repair genes: PMS2, MLH1, MSH2, and MSH6 [21]. The mismatch repair endonuclease PMS2 is known to interact with MLH1 and is a component of the post-replicative DNA mismatch repair system (MMR). PMS2 is recruited to cleave damaged DNA this recruitment is triggered by the binding of MSH2 and MSH6 proteins to dsDNA mismatches followed by the recruitment of MLH1 (Figure 1). PMS1 is also involved in the repair of DNA mismatches, and it can form heterodimers with MLH1. Additional genes in our study include the tumor suppressor gene TP53, CDH1, MUTYH, and APC. TP53 is a hub protein in the cellular DNA damage response pathway known as the P53 signaling pathway [22], it is linked with colorectal cancer and many other cancers. The genes CDH1, MUTYH, and APC also interact with one another in addition to their involvement in the MMR pathway described above. For example, CDH1 and APC combine to act as a ubiquitin ligase, which is involved in glycolysis regulation during the cell cycle [23]. In fact, most of the colon cancer associated genes in this study can be grouped into critical pathways, such as apoptosis, DNA damage control, and cell cycle signaling [24]. 

To assess if there is evidence for protein functional shift from the patterns of substitution in colon cancer associated genes we have carried out selective pressure analyses using codon models of lineage-specific rate heterogeneity.

## Methods

### Sequence data assembled

The colon cancer gene dataset used in this study consists of 22 genes taken from the Cancer Gene Census at the Sanger Institute [25]. All 22 genes have reported cases of germline mutations that are associated with colon cancer (See Table 1 for summary of data, detail on the complete dataset is available in Additional file 1 ). Using Compara data from Ensembl [26,27], single gene orthologs were identified for each gene across the vertebrate genomes chosen. The 21 species were selected based on genome coverage. These included representatives from 3 of the 4 main lineages of Eutheria, namely Afrotheria, Euarchontoglires, and Laurasiatheria, as well as outgroup species including platypus, zebrafish, and zebra finch (see Additional file 1). 

**Table 1:** Colon Cancer Gene Set analyzed in this study

**Gene (HGNC code)**	**Ensembl Identifier**	**Taxa Number**^**2**^	**Alignment Length**^**3**^	**Syndrome**	**Tumor Types Observed**	**Pathway(s)**	**References**^**4**^
APC	ENSG00000134982	20	9177	Familial adenomatous polyposis (FAP)	Colon, thyroid, stomach, intestine	APC	[19,24]
ATM	ENSG00000149311	18	9189	Ataxia telangiectasia (A-T)	Leukemia, lymphoma, colorectal	CIN	[24], [17]
BHD	ENSG00000154803	20	1737	Birt-Hogg-Dube syndrome	Renal, colon	AMPK, mTOR, STAT	[24]
BMPR1A	ENSG00000107779	19	1596	Juvenile polyposis	Gastrointestinal	SMAD	[24]
CDH1	ENSG00000039068	15	2649	Familial gastric carcinoma	Stomach	AP	[ [24,28] (E-cadherin)]
MADH4	ENSG00000141646	16	1656	Juvenile polyposis	Gastrointestinal	SMAD	[ [24], (SMAD4)]
MET	ENSG00000105976	21	4146	Hereditary papillary renal cell carcinoma (HPRCC)	Kidney, colorectal	RAS, PI3K, STAT, Beta-catenin, Notch	[24]
MLH1	ENSG00000076242	19	2274	Hereditary non-polyposis colon cancer (HNPCC)	Colon, uterus	MMR	[24]
MSH2	ENSG00000095002	18	2802	Hereditary non-polyposis colon cancer (HNPCC)	Colon, uterus	MMR	[24]
MSH6	ENSG00000116062	19	4101	Hereditary non-polyposis colon cancer (HNPCC)	Colon, uterus	MMR	[24]
MUTYH	ENSG00000132781	21	1569	Attenuated Polyposis	Colon	BER	[24]
NF1	ENSG00000196712	17	8523	Neurofibromatosis type I	Neurofibroma, colon	RTK	[24]
PMS1	ENSG00000064933	20	2799	Hereditary non-polyposis colon cancer (HNPCC)	Colon, uterus	MMR	
PMS2	ENSG00000122512	21	2592	Hereditary non-polyposis colon cancer (HNPCC)	Colon, uterus	MMR	[24]
PTEN	ENSG00000171862	18	1209	Cowden syndrome	Hamartoma, glioma, colorectum	PI3K	[24], [17]
SDHB	ENSG00000117118	18	840	Hereditary paraganglioma, Carney–Stratakis	Paragangliomas, pheochromocytomas, gastrointestinal	HIF1	[24]
SDHC	ENSG00000143252	16	507	Hereditary paraganglioma, Carney–Stratakis	Paragangliomas, pheochromocytomas, gastrointestinal	HIF1	[24]
STK11	ENSG00000118046	18	1320	Peutz-Jeghers syndrome	Intestinal, ovarian, pancreatic, colorectal	PI3K	[24,29]
TP53	ENSG00000141510	16	1185	Li-Fraumeni syndrome/sarcoma	Breast, sarcoma, adrenal, brain, colorectal	p53	[24,29]
TSC1	ENSG00000165699	18	3495	Tuberous sclerosis	Hamartoma, kidney, colorectal	PI3K	[24,29]
TSC2	ENSG00000103197	19	5436	Tuberous sclerosis	Hamartoma, kidney, colorectal	PI3K	[24,29]
VHL	ENSG00000134086	18	639	Von Hippel-Lindau syndrom	Kidney, colorectal	HIF1	[24]

Each of the 22 genes analyzed in this study are detailed, including their HGNC approved gene symbols, and Ensembl gene IDs. The total number of species analyzed for each gene and the overall length of alignment in base pairs are also given. The syndrome, tumor type observed and pathway involved are detailed. References citing alternative gene names are identified using rounded parentheses.

### Multiple sequence alignment

The coding DNA sequences of the single gene orthologs were translated and the resulting amino acid sequences were aligned using the default parameters in ClustalW 2.0.12 [30,31]. We mapped gaps from the amino acid multiple sequence alignment (MSA) to the corresponding nucleotide sequences to produce a nucleotide alignment. All alignments were reviewed for quality and any poorly aligned regions were manually edited using Se-Al [32]. All alignments are available in Additional file 2.

### Alignment criteria for selective pressure analysis

It has been shown through computer simulations that sequence length has an impact on the power to infer positive selection [33]. Power was also found to increase with greater taxonomic representation and greater divergence, although extreme levels of divergence were found to cause a reduction in power. Simulations have shown that the presence of longer foreground branches also increased the power of the test statistic, but extremely long foreground branches reduced the power [34]. To increase the statistical power of the analyses performed here we have therefore only considered single gene families containing 6 or more taxa, and alignment lengths of greater than 500 amino acids.

### Recombination analysis

Recombination events can result in the incorrect detection of positive selection. To reduce the detection of potential false positives from our analyses, we have implemented GENECONV (version 1.81a) [35]. GENECONV detects gene conversion events between ancestors of sequences in a multiple sequence alignment. Default parameters where employed, and 10,000 randomly permuted datasets were generated for each Single Gene Orthologous family and global inner fragments were listed if P-value <= 0.05 or smaller.

### Selective pressure analysis using codon models of evolution

Selective pressure analyses were performed using Codeml from PAML version 4.4 [36,37]. Because each gene family analyzed was composed of single gene orthologs, pruned species phylogenies were used as per previous publications [2,38]. Codeml implements a number of codon-based models in a Maximum Likelihood framework that can be used to test alternative and nested evolutionary hypotheses. Three different types of codon model were used in this study: (i) a homogeneous model (Model 0) - a single ω-value is estimated for the entire alignment; (ii) site-heterogeneous models - the sites of the gene sequence are grouped into two or more site classes, each with its own ω-value; and (iii) lineage-specific heterogeneous models - a different ω parameter is estimated for different site classes in combination with different lineages [9,36,39].

Seven site-heterogeneous models were used, we have retained conventional annotations for these models: Model 1a (Nearly Neutral), Model 2a (Selection), Model 3 Discrete (k = 2), Model 3 Discrete (k = 3), Model 7, Model 8 and Model 8a. Two lineage-specific heterogeneous models were used: Model A and Model A Null. These models have been applied similarly elsewhere [40].

The goodness-of-fit of the different models was assessed statistically using a likelihood ratio test (LRT). The LRT compares the log-likelihoods of a null model with the alternative model. For hierarchically nested models, the test statistic of an LRT approximates the *χ*^2^ distribution with degrees of freedom equal to the number of additional free parameters in the alternative model compared to the null model. Because of this, the critical value of the test statistic can be determined from standard statistical tables. If the p-value of the test statistic exceeds that critical value (i.e. if the alternative model fits the data significantly better than the null model), then the null model can be rejected. For example, if the test statistic of an LRT comparing Model 1a (Nearly Neutral) with Model 2a (Selection) is greater than the critical value determined from the *χ*^2^ distribution, Model 1 a can be rejected. If ω_1_ > 1 under Model 2a, positive selection may be inferred. Additional file 3 shows the set of LRTs used for selective pressure analysis.

In cases where positive selection is inferred, the posterior probability of a site belonging to the positively selected class is estimated using two calculations: Naïve Empirical Bayes (NEB) or Bayes Empirical Bayes (BEB). If both BEB and NEB are predicted, we will preferentially use the BEB results as these have been shown to be more robust [37].

In-house software was designed to prepare all files for analysis and to process all output. PAML output files were parsed for parameter estimates and log likelihood values, and LRTs were performed (see Additional file 3). Where positively selected sites were inferred under a given model, positively selected sites were mapped to the sequence (or sequences) of interest and included in the summary file (see Additional file 4). This software was used to reduce the scope for human error in setting up and interpreting PAML analyses and is available from the authors on request. Functional annotation of sites under positive selection for each protein was obtained from UniProt [41].

### Human population analysis

Selective pressures within the present day human population were analyzed for those genes with evidence of lineage-specific positive selection in the human ancestral lineages. The online tool SNP@Evolution^2^ and HapMap release II source data was used to look at variations within the East Asian (A), Northern and Western European (C), and African Yoruba (Y) populations. The “integrated haplotype score” or iHS, described first in [42], was employed here as a test for directional selection. The iHS is standardized using genome wide empirical distributions, it has an approximate normal distribution allowing for direct comparisons of the score across genes, and it outperforms in comparison to other available approaches [42]. A derived allele that has been segregating in the population receives a large iHS (> + 2) while a large negative iHS (<−2) indicates that the derived allele has increased in frequency.

## Results and discussion

Starting with a dataset of 22 genes, we identified single gene orthologs across 21 complete vertebrate genomes. Ortholog identification resulted in families with between 15 and 21 taxa, and alignment lengths of between 507 and 9,189 base pairs thus satisfying the criteria described in the materials and methods section. The test for recombination on all 22 genes is summarized in Additional file 5. The analysis revealed that only the TP53 protein showed significant levels of recombination, the regions where recombination was present were noted and compared to regions where positive selection was detected. If these regions overlapped - the positive selection result was deemed a false positive.

To assess the selective pressure variation, we performed both site- and lineage-specific selective pressure analyses and subsequently assessed the statistical significance of all results via LRT analyses to ascertain the codon evolutionary model of best fit. In those cases where the ω value vastly exceeds 1, we have simply denoted them as ω > > 1 throughout the manuscript, as there is no biological significance for these extremely large ω values (the precise numbers are shown in the Tables throughout). The lineage-specific analyses are more pertinent to the main focus of the paper, i.e. - the identification of species-specific patterns of substitution in these colon cancer associated genes. Therefore the lineage-specific results have been described in detail in the following section. Site-specific results are briefly summarized on a gene-by-gene basis. All positively selected sites were assessed using functional information from the Uniprot database [41]. The model of best fit along with associated parameter estimates are described and a summary table for each of the 22 genes is given in Additional file 4.

### Lineage-specific selective pressure analyses

Lineage-specific models of codon evolution were assessed at multiple phylogenetic depths, (i) the extant lineages within the Euarchontoglires clade, and (ii), all ancestral lineages leading from the Euarchontoglires to modern mouse and human were also tested independently as depicted in Figure 1. Analysis of the extant human and mouse lineages did not yield evidence of positive selection. Conversely, analysis of the lineages within the Euarchontoglires clade resulted in significant evidence of lineage-specific positive selection, 6 genes in ancestral lineages and 12 in extant lineages, see Figure 1. The STK11 gene showed evidence of positive selection in the Euarchontoglires ancestral lineage and again in the Hominidae ancestral lineage. CDH1 showed patterns of substitution conducive with positive selection in the ancestral primate lineage. In the ancestral Muridae lineage there is evidence for positive selection acting on the TSC1 gene. The ancestral Murinae lineage showed evidence of positive selection for both MSH6 and SDHC, see Table 2 for summary.

**Table 2:** Summary of parameter estimates and likelihood scores for the model of best fit showing evidence of positive selection

**Gene**	**Model**	**lnL**	**Parameter Estimates**	**Positive Selection**	**BEB Positively Selected Sites**
**Lineage-Specific Analyses**					
**Euarchontoglires Ancestral Branch**					
STK11	modelA	−8602.921472	p_0_ = 0.93299, p_1_ = 0.05633, p_2_ = 0.01007, p_3_ = 0.00061 ω_0_ = 0.03346, ω_1_ = 1.00000, ω_2_ = 197.90897	Yes	3 > 0.50, 1 > 0.95, 0 > 0.99
**Primate Ancestral Branch**					
CDH1	modelA	−16658.03484	p_0_ = 0.75454, p_1_ = 0.23453, p_2_ = 0.00834, p_3_ = 0.00259 ω_0_ = 0.05683, ω_1_ = 1.00000, ω_2_ = 10.20516	Yes	9 > 0.50, 1 > 0.95, 0 > 0.99
**Hominidae Ancestral Branch**					
STK11	modelA	−8601.056009	p_0_ = 0.93574, p_1_ = 0.05920, p_2_ = 0.00476, p_3_ = 0.00030 ω_0_ = 0.03323, ω_1_ = 1.00000, ω_2_ = 44.31709	Yes	3 > 0.50, 2 > 0.95, 1 > 0.99
VHL	modelA	−4263.853291	p_0_ = 0.73748, p_1_ = 0.25109, p_2_ = 0.00853, p_3_ = 0.00290 ω_0_ = 0.05985, ω_1_ = 1.00000, ω_2_ = 220.34533	Yes	1 > 0.50, 0 > 0.95, 0 > 0.99
**Chimpanzee Extant Branch**					
TSC2	modelA	−42659.27711	p_0_ = 0.90352, p_1_ = 0.09434, p_2_ = 0.00194, p_3_ = 0.00020 ω_0_ = 0.04404, ω_1_ = 1.00000, ω_2_ = 190.09480	Yes	6 > 0.50, 2 > 0.95, 2 > 0.99
VHL	modelA	−4262.098043	p_0_ = 0.73571, p_1_ = 0.25251, p_2_ = 0.00877, p_3_ = 0.00301 ω_0_ = 0.05976, ω_1_ = 1.00000, ω_2_ = 262.72662	Yes	3 > 0.50, 0 > 0.95, 0 > 0.99
**Gorilla Extant Branch**					
MSH2	modelA	−19485.4338	p_0_ = 0.92233, p_1_ = 0.06298, p_2_ = 0.01375, p_3_ = 0.00094 ω_0_ = 0.06427, ω_1_ = 1.00000, ω_2_ = 999.00000	Yes	46 > 0.50, 34 > 0.95, 18 > 0.99
TSC2	modelA	−42569.22884	p_0_ = 0.89862, p_1_ = 0.08796, p_2_ = 0.01222, p_3_ = 0.00120 ω_0_ = 0.04339, ω_1_ = 1.00000, ω_2_ = 999.00000	Yes	27 > 0.50, 14 > 0.95, 12 > 0.99
MSH6	modelA	−34009.90221	p_0_ = 0.78382, p_1_ = 0.18418, p_2_ = 0.02591, p_3_ = 0.00609 ω_0_ = 0.06974, ω_1_ = 1.00000, ω_2_ = 999.00000	Yes	46 > 0.50, 34 > 0.95, 18 > 0.99
ATM	modelA	−69374.08393	p_0_ = 0.80673, p_1_ = 0.17971, p_2_ = 0.01109, p_3_ = 0.00247 ω_0_ = 0.09745, ω_1_ = 1.00000, ω_2_ = 999.00000	Yes	48 > 0.50, 23 > 0.95, 19 > 0.99
**Orangutan Extant Branch**					
TSC1	modelA	−24068.71106	p_0_ = 0.79963, p_1_ = 0.18828, p_2_ = 0.00978, p_3_ = 0.00230 ω_0_ = 0.08020, ω_1_ = 1.00000, ω_2_ = 999.00000	Yes	13 > 0.50, 6 > 0.95,5 > 0.99
TSC2	modelA	−42673.92339	p_0_ = 0.90414, p_1_ = 0.09295, p_2_ = 0.00263, p_3_ = 0.00027 ω_0_ = 0.04433, ω_1_ = 1.00000, ω_2_ = 40.47366	Yes	9 > 0.50, 0 > 0.95, 0 > 0.99
**Marmoset Extant Branch**					
TSC2	modelA	−42616.04524	p_0_ = 0.89841, p_1_ = 0.09019, p_2_ = 0.01035, p_3_ = 0.00104 ω_0_ = 0.04325, ω_1_ = 1.00000, ω_2_ = 235.10448	Yes	38 > 0.50, 9 > 0.95
MSH6	modelA	−34009.90221	p_0_ = 0.78382, p_1_ = 0.18418, p_2_ = 0.02591, p_3_ = 0.00609 ω_0_ = 0.06974, ω_1_ = 1.00000, ω_2_ = 999.00000	Yes	45 > 0.50, 16 > 0.95, 12 > 0.99
VHL	modelA	−4262.443441	p_0_ = 0.72045, p_1_ = 0.22453, p_2_ = 0.04195, p_3_ = 0.01307 ω_0_ = 0.05886, ω_1_ = 1.00000, ω_2_ = 90.26952	Yes	10 > 0.50, 0 > 0.95, 0 > 0.99
ATM	modelA	−69583.23068	p_0_ = 0.81640, p_1_ = 0.18148, p_2_ = 0.00173, p_3_ = 0.00038 ω_0_ = 0.09939, ω_1_ = 1.00000, ω_2_ = 46.82466	Yes	2 > 0.50, 0 > 0.95, 0 > 0.99
**Muridae Ancestral Branch**					
TSC1	modelA	−24126.17894	p_0_ = 0.80995, p_1_ = 0.18416, p_2_ = 0.00481, p_3_ = 0.00109 ω_0_ = 0.08293, ω_1_ = 1.00000, ω_2_ = 999.00000	Yes	1 > 0.59, 0 > 0.95, 0 > 0.99
**Murinae Ancestral Branch**					
SDHC	modelA	−3846.690164	p_0_ = 0.87666, p_1_ = 0.08131, p_2_ = 0.03846, p_3_ = 0.00357 ω_0_ = 0.15340, ω_1_ = 1.00000, ω_2_ = 253.61375	Yes	9 > 0.50, 2 > 0.95, 1 > 0.99
MSH6	modelA	−34190.13821	p_0_ = 0.79911, p_1_ = 0.19671, p_2_ = 0.00335, p_3_ = 0.00082 ω_0_ = 0.07057, ω_1_ = 1.00000, ω_2_ = 126.22513	Yes	3 > 0.50, 1 > 0.95, 0 > 0.99
**Rat Extant Branch**					
MADH4	modelA	−6092.186945	p_0_ = 0.93360, p_1_ = 0.01536, p_2_ = 0.05021, p_3_ = 0.00083 ω_0_ = 0.01379, ω_1_ = 1.00000, ω_2_ = 102.33013	Yes	24 > 0.50, 11 > 0.95, 10 > 0.99
NF1	modelA	−37750.29866	p_0_ = 0.96609, p_1_ = 0.02476, p_2_ = 0.00892, p_3_ = 0.00023 ω_0_ = 0.02265, ω_1_ = 1.00000, ω_2_ = 999.00000	Yes	39 > 0.50, 10 > 0.95, 10 > 0.99
**Guinea pig Extant Branch**					
TSC1	modelA	−24116.58577	p_0_ = 0.80206, p_1_ = 0.18611, p_2_ = 0.00961, p_3_ = 0.00223 ω_0_ = 0.08093, ω_1_ = 1.00000, ω_2_ = 284.22603	Yes	9 > 0.50, 4 > 0.95, 0 > 0.99
NF1	modelA	−37849.50819	p_0_ = 0.97375, p_1_ = 0.02506, p_2_ = 0.00116, p_3_ = 0.00003 ω_0_ = 0.02414, ω_1_ = 1.00000, ω_2_ = 171.64068	Yes	3 > 0.50, 1 > 0.95, 0 > 0.99
**Rabbit Extant Branch**					
MLH1	modelA	−19516.63525	p_0_ = 0.80595, p_1_ = 0.18541, p_2_ = 0.00703, p_3_ = 0.00162 ω_0_ = 0.05262, ω_1_ = 1.00000, ω_2_ = 7.52747	Yes	5 > 0.05, 3 > 0.95, 0 > 0.99
MUTYH	modelA	−15911.6175	p_0_ = 0.61027, p_1_ = 0.37605, p_2_ = 0.00846, p_3_ = 0.00522 ω_0_ = 0.07703, ω_1_ = 1.00000, ω_2_ = 998.99697	Yes	5 > 0.50, 4 > 0.95, 3 > 0.99
SDHC	modelA	−3822.683246	p_0_ = 0.57771, p_1_ = 0.06636, p_2_ = 0.31926, p_3_ = 0.03667 ω_0_ = 0.12047, ω_1_ = 1.00000, ω_2_ = 3.59059	Yes	51 > 0.50, 10 > 0.95, 8 > 0.99
ATM	modelA	−69582.95152	p_0_ = 0.81572, p_1_ = 0.18045, p_2_ = 0.00313, p_3_ = 0.00069 ω_0_ = 0.09930, ω_1_ = 1.00000, ω_2_ = 7.41594	Yes	6 > 0.50, 0 > 0.95, 0 > 0.99
BHD	modelA	−13523.51719	p_0_ = 0.90728, p_1_ = 0.05930, p_2_ = 0.03137, p_3_ = 0.00205 ω_0_ = 0.02817, ω_1_ = 1.00000, ω_2_ = 6.50017	Yes	10 > 0.50, 7 > 0.95, 1 > 0.99
**Site-specific Analyses**					
CDH1	m8	−16589.88768	p = 0.21848, p_0_ = 0.99291, p_1_ = 0.00709, q = 0.80842 ω=4.53766	Yes	15 > 0.5, 1 > 0.95, 0 > 0.99
PMS1	m8	−26480.39761	p = 0.61337, p_0_ = 0.93580, p_1_ = 0.06420, q = 1.93110 ω=1.32691	Yes	25 > 0.50, 1 > 0.95, 0 > 0.99
PMS2	m8	−27449.3651	p = 0.29104, p_0_ = 0.91064, p_1_ = 0.08936, q = 1.31619 ω=1.28855	Yes	37 > 0.50, 1 > 0.95, 0 > 0.99
MUTYH	m8	−15797.6226	p = 0.37255, p_0_ = 0.97242, p_1_ = 0.02758, q = 1.00900 ω=2.44412	Yes	18 > 0.5, 1 > 0.95, 0 > 0.99
TP53	m8	−8688.19126	p = 0.40362, p_0_ = 0.94645, p_1_ = 0.05355, q = 1.77507 ω=1.97385	Yes	13 > 0.5, 3 > 0.95, 0 > 0.99

The model of best fit is summarized below for those genes with evidence of positive selection. The lineage-specific results for each lineage tested from the Euarchontoglires ancestor to modern lineages are shown in the top panel and the site-specific results are shown in the bottom panel. The model abbreviations are as per main text. P refers to the number of free parameters estimated in that model. BEB = Bayes Empirical Bayes estimations. The number of positively selected sites identified can be found the final column, sites are separated by the posterior probability cutoffs of 0.50, 0.95, and 0.99.

In the following section, we have analyzed the positively selected sites for those genes with evidence of lineage-specific positive selection in the context of their potential functional relevance for those genes. This was carried out for all genes where functional sites and/or domains have been elucidated. All sites described were calculated via Bayes Empirical Bayes (BEB) analysis (unless otherwise specified). In all cases we are assessing the potential functional importance of residues based on their sequence position. There are instances where we identify stretches of protein sequence under positive selection - there is a possibility that these sites may have very different functions despite their sequence position. For a total 16 of the 22 genes there were partial or complete 3D structures available. However, many of the positively selected sites identified were located in regions that were not yet fully resolved at the structural level, and so only the 3D model for STK11 is given. 

#### Positive selection in the Euarchontoglires Ancestral branch

The most ancestral branch tested was the Euarchontoglires ancestral branch, i.e. the ancestor of the Primate, Rodent and Glires clades as depicted in Figure 1. The STK11 alignment consists of 18 taxa and was the only gene that showed evidence of positive selection in this ancestral lineage. STK11 (Serine/Threonine-protein kinase 11) plays an essential role in G1 cell cycle arrest and acts as a tumor suppressor. It phosphorylates and activates members of the AMPK-related subfamily of protein kinases [43,44],). Mutations in STK11 cause Peutz-Jeghers syndrome (PJS), this is a rare autosomal dominant disorder characterized by multiple gastrointestinal hamartomatous polyps and an increased risk of various neoplasms including gastrointestinal cancer [45,46]. From the literature we currently know of 17 sites across this gene that when mutated are associated with colon-cancer. The Euarchontoglires ancestral lineage has 1.1% of sites under positive selection (ω > > 1). The positively selected residues were located on the 3D structure of this enzyme (See Figure 1 inset). Position 206 with a Posterior Probability (PP) = 0.889 is a hydrophobic Alanine or Valine in Euarchontoglires species and is a negatively charged Glutamic acid or positively charged Lysine in non-Euarchontoglires species. This residue also lies in close proximity to sporadic cancer site A205T and colorectal cancer site D208N in Human [47]. Positively selected position 301 in Euarchontoglires (P = 0.885) is present in Euarchontoglires species as an Arginine residue and all non-Euarchontoglires as an uncharged Glutamine residue. Site 301 is close to R297K and region 303–306 both of which have been implicated in PJS [48].

#### Positive selection in the Primate Ancestral branch

The branch leading from the Euarchontoglires ancestor towards the primates was analyzed, we have termed this branch the ancestral Primate branch as depicted in Figure 1. The CDH1 dataset consists of 15 taxa and following LRT analyses showed evidence of lineage-specific positive selection in 1.1% of sites in the Primate Ancestor (ω=10.21). Positively selected sites were compared to human Swiss-Prot entry (P12830) and it was found that position 604, with a PP of 0.549, falls in close proximity to gastric cancer variant R598Q [49]. At position 604 Primates have a negatively charged Glutamic acid while non-primates have a polar uncharged Glutamine.

#### Positive selection in the Hominidae Ancestral branch

**Figure 2:**
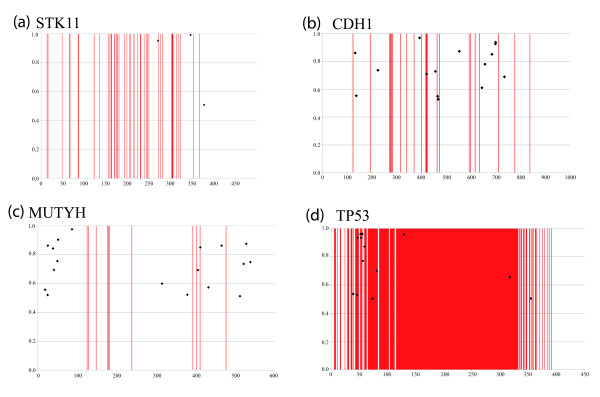
**Positive selection analysis for 4 genes: (a) STK11, (b) CDH1, (c) MUTYH, and (d) TP53.** The x-axis depicts the gene from start to end of alignment. The Y-axis is the posterior probability. The vertical red bars on each graph represent the known cancer causing variants from human populations. The black dots on each graph represent the positively selected sites identified in this study.

The next branch in the primate clade is that leading to modern great apes, i.e. Hominidae, as depicted in Figure 1. This lineage also showed evidence of positive selection again in the STK11 gene in 0.51% of sites, with ω > > 1. See Figure 2(a) and Table 2. The positively selected positions were compared to the human Swiss-Prot sequence (Q15831). Position 347 represents a radical substitution, as the Hominidae code for an Alanine (a small hydrophobic residue) whereas the Murinae lineage encode an Arginine at this position (a basic, hydrophilic, and positively charged residue). For positively selected site 378, the ancestral Hominidae lineage encodes the polar residue Serine, while the closely related species studied encode the small amphiphilic Glycine. The functions of these specific sites have not been reported thus far in the literature but are likely to be of considerable interest as they mark adaptations unique to the ancestral Hominidae.

A second gene showing evidence of positive selection in the Hominidae ancestral branch is the VHL dataset consisting of 18 taxa. The VHL gene encodes Von Hippel-Lindaue tumour suppressor protein. Mutations occurring in this gene can result in von Hippel-Lindau disease (VHDL) - a dominantly inherited familial cancer syndrome [50]. VHL exhibited weak evidence of positive selection with 1.1% of sites in the ancestral Hominidae lineage under positive selection. There was one amino acid that had low coverage in the alignment (present only in 6/18 species), as this is a very weak results we have not expanded upon it any further.

#### Positive selection in the Extant Primate branches

Analysis of modern non-human primates also identified positive selection in a number of genes. In VHL positive selection was detected in the Chimpanzee lineage where 1.2% of sites have ω > > 1, and also in the Marmoset lineage where 5.5% sites have ω > > 1. Sites under positive selection were compared against human Swiss-Prot entry (P40337), however the region (1–60) was only represented by 11/18 species in the alignment and therefore we do not have sufficient confidence in these positions to explore these sites in more detail.

The MSH6 gene dataset contained 19 taxa and showed evidence of positive selection in both the Gorilla and Marmoset lineages each displaying 3.2% of sites with ω > > 1. Gorilla and Marmoset extant lineages were compared against human (P52701) Swiss-Prot entry. No relevant functional information could be extracted from positively selected sites in Gorilla, however 2/45 positively selected sites in Marmoset fall in close proximity to cancer variants. In marmoset, positively selected site 803 (PP = 0.551) coincides with Human colorectal cancer variants D803G [51] and V800A [52]. Position 803 in Marmoset is a negatively charged Glutamic acid while in all other mammals it is a small negatively charged Aspartic acid. Positively selected site 1099 in Marmoset (PP = 0.614) is located between human colorectal cancer variants R1095H [53] and T1110C [54].

MSH2 alignment consists of 18 taxa. The function of the MSH2 protein is in post-replicative DNA mismatch repair system (MMR). Mutations in MSH2 result in hereditary non-polyposis colorectal cancer type 1 (HNPCC1) [55]. Lineage-specific positive selection was identified in 1.5% of sites within the extant Gorilla lineage with ω > > 1. Positively selected sites were compared against human Swiss-Prot sequence (P43246). All 15 of the BEB identified sites occur between amino acid position 124–142 which overlaps with the region containing variants N127S, N139S and I145M associated with HNPCC1 [55].

Tuberous sclerosis 2 protein (TSC2) interacts with TSC1 protein and mutations in this gene can cause tuberous sclerosis type 2 [56]. The alignment of TSC2 consisted of 19 taxa. Lineage-specific positive selection was identified in the following extant lineages, the percentage of sites under positive selection in each lineage is shown in brackets, in all cases ω > > 1: Chimpanzee lineage (0.2%), Gorilla (1.3%), Orangutan (0.29%), and, Marmoset (1.1%). Positively selected sites were compared against human Swiss-Prot sequence (P49815) however the functional information was not available to contextualize these results.

ATM acts as a DNA checkpoint sensor by activating checkpoint signaling upon double strand breaks [57]. The alignment of ATM consisted of 18 taxa and positive selection was detected in the following lineages (again the percentage of the alignment under positive selection is shown in brackets): Gorilla (1.4%, ω > > 1), Marmoset (0.21%, ω > > 1), and Rabbit (0.38%, ω = 7.42). BEB significant sites were compared to human (Q13315) and mouse (Q62388) Swiss-Prot entries to determine the functional relevance of selected sites. In the Gorilla lineage positively selected site 2067 (PP = 0.787), where in humans a substitution of Alanine to Aspartate in this same position can result in Ataxia telangiectasia (AT) which is a severe disease that causes weakened immune function and greater predisposition to cancer [57]. No other functionally relevant information was found upon comparison of Swiss-Prot information against either Marmoset or Rabbit.

The extant Orangutan lineage also showed evidence of positive selection in the TSC1 gene for 1.2% of its alignment (ω > > 1). Positively selected sites were compared against human Swiss-Prot sequence (Q92574) and mouse Swiss-Prot sequence (Q9EP53) however there was insufficient information to extrapolate the potential functional impact of these sites.

### Human population level analysis using HapMap data

Genes displaying evidence of positive selection in lineages leading to *Homo sapiens,* i.e. the primate and Hominidae lineages (STK11, CDH1 and VHL), were further analyzed to determine if there is evidence for ongoing positive directional selection in modern day human populations. The integrated haplotype score, iHS [42], was calculated for each SNP in STK11, CDH1 and VHL genes across African Yorubu (Y), East Asian (A) and European (C) populations. One intronic SNP in the SDK11 gene, had an iHS score of +2.0385 in European populations. In the CDH1 gene, two intronic SNPs with iHS scores of +2.0433 and +2.5838 respectively were identified in the East Asian populations. iHS scores of greater than +2 indicate that these alleles are segregating at a significant rate within their given populations. No population level directional selection was identified in the VHL gene in modern humans.

#### Positive selection in the Ancestral Muridae branch

The ancestral Muridae branch marks the most recent common ancestor of modern mouse, rat and guinea pig species and is depicted in Figure 1. Tuberous sclerosis 1 protein (TSC1) interacts with TSC2 and acts as a tumour suppressor gene [56]. Defects in TSC1 cause tuberous sclerosis type 1 which is an autosomal dominant multi-system disorder. There were a total of 18 taxa analysed in for the TSC1 gene and 0.59% of sites in the Muridae ancestral lineage were identified with ω > > 1. As before for TSC1: positively selected sites were compared against human Swiss-Prot sequence (Q92574) and mouse Swiss-Prot sequence (Q9EP53) however there was insufficient information to extrapolate potential functional impacts of these sites.

#### Positive selection in the Ancestral Murinae branch

The ancestral Murinae branch defines the most recent common ancestor of mouse and rat. In total there were two genes identified as being under positive selection in the Murinae lineage. The first is the MSH6 gene that acts as a DNA mismatch repair protein and is a component of the post–replicative DNA mismatch repair system [58]. MSH6 also heterodimerizes with MSH2 to form MutS-alpha, a protein complex that functions by binding to DNA mismatches and initiating DNA repair [59]. Mutations in MSH6 have been reported to cause HNPCC type 5 [60], atypical HNPCC, and familial colorectal cancers (suspected or incomplete HNPCC) [61]. The MSH6 dataset consists of 19 taxa. Lineage-specific analysis of the ancestral Murinae lineage revealed 0.42% of the sites (3 residues) in MSH6 under positive selection, ω > > 1 (see Table 2). The corresponding Swiss-Prot sequence (P54276) lacked functional details for these positions, therefore, potential functional effects remain unknown. However, examination of the alignment at this position revealed the substitution of residues with unrelated biochemical properties at these positions. At positively selected site 374 (numbered as per Swiss-Prot entry), the Murinae lineage has a Proline whereas remaining species tested encode either Glutamic acid, Aspartic acid, or Lysine. As Proline produces “kinks” in the α-helical regions of proteins, such a substitution could alter the protein structure substantially. Positively selected site 759 is a Leucine in the Murinae, all other non-outgroup species encode aliphatic residues (Isoleucine or Valine). The ancestral Murinae has a Cysteine at Swiss-Prot position 1259 while all other species have an Alanine at this position. These residues are of specific interest for further *in vitro* functional assaying given their uniqueness to the rodent clade and their retention in all modern rodents tested.

The second gene with evidence of positive selection on the ancestral Murinae lineage is the SDHC (Succinate dehydrogenase cytochrome b 560 subunit, mitochondrial) gene. The SDHC function is to act as a membrane-anchoring subunit for the SDH protein. Defects in this protein are reported in paragangliomas and gastric stromal sarcomas [62]. The dataset for the SDHC consists of 16 taxa. Lineage-specific positive selection was detected in the ancestral Murinae lineage with 4.2% of sites (9 residues) in this protein with ω > > 1 (Table 2). Comparison with the human sequence from Swiss-Prot (Q99643) and mouse sequence (Q9CZB0) placed 8 of these sites either in transmembrane or topological domains across the gene, with the additional positively selected residue (position 128) neighboring a metal binding site at position 127.

#### Positive selection in the Extant Rabbit branch

The SDHC gene again showed evidence of positive selection, this time in the extant Rabbit lineage with 35.59% of sites under positive selection (ω = 3.59). 15/51 positively selected sites were identified as occurring within 10 amino acid positions of the metal binding site 127, also mentioned in the ancestral Murinae analysis. While there are extremely high levels of positive selection identified in the rabbit lineage, no other relevant functional information could be gleaned from the databases at this point.

The MUTYH alignment consisted of 21 taxa and showed evidence of lineage-specific positive selection in 1.4% of sites in the extant Rabbit lineage (ω > > 1). Positively selected sites were compared to human (Q9UIF7) and mouse (Q99P21) Swiss-Prot entries, however no relevant functional information could be extrapolated. Radical substitutions occurred in all 5 BEB sites in the extant Rabbit lineage, three of which are at positions 485–487 in the Nudix hydrolase domain.

The MLH1 gene codes for a critical protein involved with the post-replicative DNA mismatch repair system. Defects in this gene result in hereditary non-polyposis colorectal cancer type 2 (HNPCC2) [63]. The alignment of MLH1 consists of 19 taxa and again positive selection was detected in the extant rabbit lineage in 0.87% of sites (ω = 7.53). Positively selected sites were compared against human Swiss-Prot sequence (P40692) and mouse Swiss-Prot sequence (Q9JK91). At amino acid position 120, Rabbit has a polar uncharged Serine residue while all other species tested have a hydrophobic Alanine residue. This positively selected site falls in a region dense with HNPCC2 variants at positions A111V, T116K, T117M, Y126N, A128P [63-65]. Positively selected residues in Rabbit: 209, 478 and 514, each fall within 8 amino acid positions of HNPCC2 variants: V213M, R474Q and V506A [66]. And position 478 identified as under positive selection also lies in close proximity to a colorectal cancer variant R472I [67].

Finally, the BHD gene showed evidence of positive selection in the extant Rabbit lineage. The function of the BHD gene is still largely unknown, however it is thought that it may be a tumour suppressor and it may be involved in colorectal tumorigenesis [68]. The alignment consisted of 20 taxa and positive selection was detected in 3.34% of sites (ω = 6.5), again unique to the Rabbit lineage. BEB significant sites were compared to human (Q8NFG4) and mouse (Q8QZS3) Swiss-Prot entries to determine their functional relevance. All 10 of the positively selected sites in Rabbit occur in a small region from position 61–83 and border a known human cancer variant at position 79 that when mutated from Serine to Tryptophan results in sporadic colorectal carcinoma.

#### Positive selection in the Extant Rodent and Guinea Pig branches

MADH4 is the co-activator and mediator of signal transduction by TGF-beta. Defects in MADH4 result in pancreatic, colorectal, juvenile polyposis syndrome, juvenile intestinal polyposis and primary pulmonary hypertension [69,70]. The Rat lineage has lineage-specific positive selection in the MADH4 gene where 5.1% of sites are evolving with ω > > 1 (number of taxa = 16). Positively selected sites were compared to human (Q13485) and mouse (P97471) Swiss-Prot entries. The majority of positively selected residues in this protein are sequential with 18/24 sites under positive selection in the rat lineage within 10 amino acid positions of the natural human variant 493. When position 493 is mutated from Aspartate to Histidine pancreatic carcinoma is induced [71].

NF1 is thought to be a regulator of RAS activity [72]. Defects in NF1 can cause colorectal carcinoma and breast cancer [70]. The NF1 dataset consists of 17 taxa. Lineage-specific positive selection was identified in 0.92% of sites in Rat (ω > > 1) and 0.12% of sites in guinea pig (ω > > 1). BEB significant sites were compared to human (P21359) and mouse (Q04690) Swiss-Prot sequences, however there was no functionally relevant information available.

TSC1 also shows evidence of positive selection in the extant guinea pig lineage where 1.2% of the sites have ω > > 1. As before, the positively selected sites were compared against human Swiss-Prot sequence (Q92574) and mouse Swiss-Prot sequence (Q9EP53) however there was insufficient information to extrapolate potential functional impacts of these sites.

#### Results of site-specific selective pressure analyses

The site-specific results may be beneficial to those working on rational mutagenesis and/or the identification of functionally important regions in these colon cancer associated genes and so these results have been summarized. We have identified five genes that have signatures of site-specific positive selection, namely: CDH1, MUTYH, PMS1, PMS2 and TP53, representing ~23% of the dataset. For each of these five genes, the model of best fit was the site-heterogeneous model “model 8”, see Table 2 for summary.

Defects in the CDH1 member of the Cadherin family are linked to hereditary diffuse gastric cancer [24,28]. The CDH1 alignment contained 15 taxa and site-specific analyses revealed 0.71% sites evolving under strong positive selection, ω = 4.54, see Table 2. We compared these sites to the human Swiss-Prot entry (P12830) to obtain relevant functional information, see Figure 2(b). The vast majority of positively selected sites (12 sites) in the protein are within the extracellular topological domain (positions 155–709). Many of these positively selected sites are in close proximity to natural cancer variants. For example, position 421 is under position selection and resides within a region (418–423) known to be missing in gastric carcinoma samples [73]. Positions 457, 465, and 467 are under positive selection and map in close proximity to natural variant E463Q found in gastric carcinoma samples [49]. Position 700 resides within the metalloproteinase cleavage site (700–701) of CDH1. Position 735 is in close proximity to a gamma-secretase/PS1 cleavage site (731–732) [74], and position 553 is in close proximity to a glycosylation site (558), essential for the posttranslational modification of proteins [75]. In the CDH1 gene, the majority of species tested (8/15) have hydrophobic residues (Isoleucine, Valine, Leucine) at position 553, the glires group (mouse, rat, guinea pig and rabbit) have small residues (Alanine, Serine, Threonine), but human, gorilla, and dog have large aromatic residues (Phenylalanine) that could significantly alter the protein structure and may affect binding at the glycosylation site at position 558.

The MUTYH dataset consisted of 21 taxa and site-specific analysis identified 18 sites under positive selection (ω = 2.44), representing 2.8% of the MUTYH protein (Table 2). A total of 10 unique sites are reported as natural cancer variants in human (Q9UIF7), see Figure 2(c). Positively selected sites 406 and 412 are in close proximity to natural cancer variants at positions 402 and 411. Positively selected sites 521, 528 and 538 also map in close proximity to natural variants, 526 and 531 respectively. Also of note are the replacement substitutions observed at Swiss-Prot positions 406 and 412, these are radical with potential effects on protein structure. At position 406 there is a large aromatic Trytophan in Primates, and a hydrophobic Leucine and Valine present in the Glires. At position 412 there is an hydrophobic Leucine in Primates and a positively charged Histidine in the Glires.

PMS1 (postmeiotic segregation increased 1) encodes a DNA mismatch repair protein and this dataset consists of 20 taxa. Defects in PMS1 are reported to cause hereditary non-polyposis colorectal cancer type 3 (HNPCC3) [76]. Analysis of PMS1 identified site-specific model of codon evolution model 8 as best fit, estimating 25 positively selected sites (6.4% of the alignment) with ω = 1.33 (Table 2). We compared these sites against human Swiss-Prot sequence P54277. Positively selected site 387 resides in close proximity to position 394 - a natural variant (M394T) reported in incomplete HNPCC and HNPCC3 [77]. Due to limited functional data it was unfeasible to study the remaining 24 sites. However, due to PMS1 function in DNA mismatch repair, these positively selected sites could prove ideal as candidates for mutagenesis studies in the future.

Mismatch repair endonuclease PMS2 (postmeiotic segregation increased 2) is a component of the post-replicative DNA mismatch repair system [78]. Defects in PMS2 are reported in HNPCC [76]. The PMS2 dataset contained 21 taxa and site-specific analysis identified 8.9% of sites under positive selection in this PMS2 protein, ω = 1.29 (Table 2). Functional relevance of these sites was determined by comparison to Human Swiss-Prot sequence (P54278). The vast majority of sites (32) reside within the 430–645 region of the alignment. This region of the alignment is highly variable and could not be not improved manually. Functional characterization for this region is also lacking and therefore we could not assess functional relevance. Outside this region, two positively selected sites, 402 and 406 (PP = 0.632 and 0.728 respectively) flank a phosphoserine modification site (403) [79]. Both substitutions are radical and could affect the function at position 403.

TP53 (cellular tumor antigen p53) acts as a tumor suppressor by inducing apoptosis or arresting growth depending on the physiological circumstances and cell type [80]. The TP53 protein (P04637) is 393 residues in length with 343 of these sites reported as natural variants that cause/lead to cancer including but not limited to colorectal and gastric cancers [41,81,82]. In our analysis of TP53 we have 16 taxa. Mutations in this gene radically affect function and therefore we would expect to find evidence of strong purifying selection across sites and lineages. However, results indicate that site specific positive selection is at work with 13 sites under positive selection, ω = 1.97. See Figure 2(d) and Table 2 for detailed analyses. On inspection of these 14 sites, we determine that 11 are located within the first region of the protein (positions 1–83), a region responsible for interaction with the methyltransferase HRMT1L2 and the recruiting of promoters to the TP53 gene [83]. We identified a cluster of positively selected sites, namely positions 46 and 47, along with an additional 7 sites within ten residues 39, 52, 53, 54, 55, 56, and 59 (see Additional file 4). Mutation of position 46 can abolish phosphorylation by HIPK2 and acetylation of K-382 by CREBBP [84]. Region 66–110 of TP53 is involved in interaction with WWOX protein and we have identified two sites (Swiss-Prot positions: 72 and 81), under positive selection within this region. Positively selected position 129, is located within a region reported to interact with HIPK1 (100–370) and AXIN1 (116–292), and in addition is also located within a region (positions 113–236) that is required for interaction with FBX042. Positively selected residue 355 is located within the CARM1 interaction region (300–393), the HIPK2 interacting region (319–360), and the oligomerization region (325–356).

## Conclusion

The results we have presented are indicative of selective pressures acting in a lineage-specific manner. The positively selected sites we have identified in this study frequently reside in regions of functional importance, such as glycosylation sites, protease cleavage sites, and sites known to interact with proteins involved in DNA damage repair pathways. Also of note, positively selected residues are frequently located at, or in close proximity to, known cancer associated sites although the statistical significance of these coincidences cannot be concluded with such a small sample sizes. Larger sample sizes and more complete functional information will be hugely beneficial in resolving whether these positively selected residues are most likely positioned to or at variants associated with cancer.

In using the mouse as a model organism for colon cancer, we are making an assumption that the orthologs in both species are functioning in precisely the same way despite ~ 180 MY of independent evolution. We found no evidence of functional divergence in the extant human and mouse lineages for the genes analyzed. However, upon testing the lineages leading from the MRCA of mouse and human, i.e. Euarchontoglires, positive selection has occurred on certain ancestral branches and in specific extant lineages. In the ancestral lineages of primates, rodents and glires there is evidence of positive selection in 6 of the 22 genes tested (this includes the VHL result but as from Table 2 it is clear that this is a weak result). In total, considering all lineages analyzed including extant lineages, we have detected lineage-specific positive selection in 64% of the genes analyzed (i.e. 14/22 genes). Studies on the levels of polymorphism observed in *Drosophila* species indicate that positive selection is pervasive in this species with positive selection present in ~25% of the genes [85]. Previous studies on the levels of positive selection in primates compared to rodents and in the Hominidae reveal much lower levels of positive selection in the range of 5-9% of genes in the genome [7,8]. If these previous analyses were to act as a measurement of expectation then we should have identified only 1 gene under positive selection in this dataset that is comprised of mammals for the most part (taking the Drosophila data as the upper bound we would expect in the region of 6 genes with evidence of positive selection).

On grouping the cancer associated genes according to their involvement in functional pathways we determined that the MMR DNA damage response pathway has evidence of positive selection in 3 components of the pathway – 2 of which are site-specific and one of which is specific to the ancestral Murinae lineage suggesting a specific selective pressure in this clade for this process. The site-specific analyses identified a total of 5 genes that are positively selected: CDH1, MUTYH, PMS1, PMS2 and TP53. These results are important for contributing to our understanding of fundamental functions of these proteins and have provided potential targets for rational mutagenesis.

Overall, these results indicate that the function of certain proteins associated with colon cancer display distinct lineage-specific patterns of substitution indicative of positive selection in the ancestral human and mouse lineages. There are a number of selective pressures on any given protein that can contribute to patterns of substitution that are “*falsely”* indicative of positive selection. The necessity to continue to interact with protein partners may be a strong driving force in the evolution of the proteins in this study as many form functional complexes with one another or other proteins [86]. Compensatory mutations may also contribute to elevated levels of ω [87]. The effective population size (*N*_*e*_) of the species tested vary enormously, with estimations for modern human populations in the range of *N*_*e*_ = 7,500 to 3,100 [88], while estimations for modern mouse populations range from *N*_*e*_ = 58,000 to 25,000 [89] and this large difference in *N*_*e*_ may also contribute to detection of false positives. We have also detected weak evidence for ongoing selective pressure in the human genome on the STK11 and CDH1, but these signals of selection may be artifacts of the very small effective population size of modern humans. Smaller *N*_*e*_ values are associated with increased fixation of slightly deleterious substitutions and subsequent elevated ω values [90]. Such slightly deleterious mutations in turn can lead to additional compensatory substitutions that become fixed. Teasing apart substitutions that have become fixed due to positive selection from slightly deleterious substitutions fixed due to small *N*_*e*_*[*[91] will aid in a more complete understanding of protein evolution in the future.

## Abbreviations

BEB: Bayes empirical bayes; *dN*: Nonsynonymous substitutions per nonsynonymous site; *dS*: Synonymous substitutions per synonymous sites; LRT: Likelihood ratio test; ML: Maximum likelihood; MY: Millions of years; *N*_*e*_: Effective population size; NEB: Naïve empirical bayes; PP: Posterior probability.

## Competing interests

The authors declare no conflict of interest.

## Authors’ contributions

CCM and KS carried out all data assembly. KS, CCM and AEW carried out all homolog identification and MSAs. CCM carried out all data quality and phylogeny analyses. CCM, KS, AEW, and TAW carried out all selective pressure analyses and designed the necessary software. ML carried out all structural analyses. All authors participated in drafting the manuscript. MJO'C conceived of the study, its design and coordination and drafted the manuscript. All authors read and approved the final manuscript.

## Supplementary Material

Additional file 1 **Details of the data used in the analysis, the 21 species and their genome coverage.** Orthologs that were not found by the Ensembl genome browser are labeled in black, orthologs identified are shown in white. Click here for file

Additional file 2 **Complete set of all multiple sequence alignments used in the analysis.** The data is presented on a gene-by-gene basis in nexus format. Click here for file

Additional file 3 Likelihood ratio tests performed and their associated significance values. Click here for file

Additional file 4 **Full set of models, associated likelihood scores and parameter estimates for all genes in the colon cancer gene dataset.** This information is given alphabetically on a gene-by-gene basis. All estimated parameters, Likelihood values and BEB or NEB sites are listed. Click here for file

Additional file 5 **Full set of recombination test results on a per gene and per species basis.** The value highlighted in yellow for TP53 represents a region where recombination was detected with reasonable confidence that also coincided with a positively selected residue (i.e. false positive). Click here for file
