# Supplementary material for: Colon cancer associated genes exhibit signatures of positive selection at functionally significant positions
Source: BMC Evol Biol. 2012 Jul 12;12:114. doi: 10.1186/1471-2148-12-114 (PMC3563467; doi:10.1186/1471-2148-12-114)
Supplement: Additional file 1 — Details of the data used in the analysis, the 21 species and their genome coverage. Orthologs that were not found by the Ensembl genome browser are labeled in black, orthologs identified are shown in white. [file 1471-2148-12-114-S1.doc]

**Supplementary File 1: Details of the data used in the analysis, the 21 species and their genome coverage.**

| Species | Assembly^1^ | APC | ATM | BHD | BMPR1A | CDH1 | MADH4 | MET | MLH1 | MSH2 | MSH6 | MUTYH | NF1 | PMS1 | PMS2 | PTEN | SDHB | SDHC | STK11 | TP53 | TSC1 | TSC2 | VHL |
| --- | --- | --- | --- | --- | --- | --- | --- | --- | --- | --- | --- | --- | --- | --- | --- | --- | --- | --- | --- | --- | --- | --- | --- |
| Human | GRCh37p2 |  |  |  |  |  |  |  |  |  |  |  |  |  |  |  |  |  |  |  |  |  |  |
| Chicken | WASHUC2 |  |  |  |  |  |  |  |  |  |  |  |  |  |  |  |  |  |  |  |  |  |  |
| Chimpanzee | CHIMP2.1 |  |  |  |  |  |  |  |  |  |  |  |  |  |  |  |  |  |  |  |  |  |  |
| Cow | Btau_4.0 |  |  |  |  |  |  |  |  |  |  |  |  |  |  |  |  |  |  |  |  |  |  |
| Dog | CanFam_2.0 | |  |  |  |  |  |  |  |  |  |  |  |  |  |  |  |  |  |  |  |  |  |
| Elephant | loxAfr |  |  |  |  |  |  |  |  |  |  |  |  |  |  |  |  |  |  |  |  |  |  |
| Frog | JGI4.1 |  |  |  |  |  |  |  |  |  |  |  |  |  |  |  |  |  |  |  |  |  |  |
| Fugu | FUGU4.0 |  |  |  |  |  |  |  |  |  |  |  |  |  |  |  |  |  |  |  |  |  |  |
| Gorilla | gorGor3 |  |  |  |  |  |  |  |  |  |  |  |  |  |  |  |  |  |  |  |  |  |  |
| Guinea Pig | cavPor3 |  |  |  |  |  |  |  |  |  |  |  |  |  |  |  |  |  |  |  |  |  |  |
| Horse | EquCab2 |  |  |  |  |  |  |  |  |  |  |  |  |  |  |  |  |  |  |  |  |  |  |
| Marmoset | culJac3 |  |  |  |  |  |  |  |  |  |  |  |  |  |  |  |  |  |  |  |  |  |  |
| Mouse | NCBIM37 |  |  |  |  |  |  |  |  |  |  |  |  |  |  |  |  |  |  |  |  |  |  |
| Opossum | monDom5 |  |  |  |  |  |  |  |  |  |  |  |  |  |  |  |  |  |  |  |  |  |  |
| Orangutan | PPYG2 |  |  |  |  |  |  |  |  |  |  |  |  |  |  |  |  |  |  |  |  |  |  |
| Pig | Sscrofa9 |  |  |  |  |  |  |  |  |  |  |  |  |  |  |  |  |  |  |  |  |  |  |
| Platypus | OANA5 |  |  |  |  |  |  |  |  |  |  |  |  |  |  |  |  |  |  |  |  |  |  |
| Rabbit | oryCun2.0 |  |  |  |  |  |  |  |  |  |  |  |  |  |  |  |  |  |  |  |  |  |  |
| Rat | RGSC3.4 |  |  |  |  |  |  |  |  |  |  |  |  |  |  |  |  |  |  |  |  |  |  |
| Zebra Finch | teaGut3.2.4 |  |  |  |  |  |  |  |  |  |  |  |  |  |  |  |  |  |  |  |  |  |  |
| Zebrafish | Zv9 |  |  |  |  |  |  |  |  |  |  |  |  |  |  |  |  |  |  |  |  |  |  |
