# Supplementary material for: Colon cancer associated genes exhibit signatures of positive selection at functionally significant positions
Source: BMC Evol Biol. 2012 Jul 12;12:114. doi: 10.1186/1471-2148-12-114 (PMC3563467; doi:10.1186/1471-2148-12-114)
Supplement: Additional file 2 — Complete set of all multiple sequence alignments used in the analysis. The data is presented on a gene-by-gene basis in nexus format. [file 1471-2148-12-114-S2.doc]

#NEXUS

BEGIN DATA;

DIMENSIONS NTAX=15 NCHAR=2649;

FORMAT DATATYPE=DNA INTERLEAVE=yes GAP=-;

[Gene Name: CDH1]

[Name: Human Len: 2649 Check: 0]

[Name: Chimpanzee Len: 2649 Check: 0]

[Name: Cow Len: 2649 Check: 0]

[Name: Dog Len: 2649 Check: 0]

[Name: Elephant Len: 2649 Check: 0]

[Name: Gorilla Len: 2649 Check: 0]

[Name: Guinea_Pig Len: 2649 Check: 0]

[Name: Horse Len: 2649 Check: 0]

[Name: Marmoset Len: 2649 Check: 0]

[Name: Mouse Len: 2649 Check: 0]

[Name: Opossum Len: 2649 Check: 0]

[Name: Orangutan Len: 2649 Check: 0]

[Name: Platypus Len: 2649 Check: 0]

[Name: Rabbit Len: 2649 Check: 0]

[Name: Rat Len: 2649 Check: 0]

MATRIX

Human ATGGGCCCTT GGAGCCGCAG CCTCTCGGCG CTGCTGCTGC TGCTGCAGGT

Chimpanzee ATGGGCCCTT GGAGCCGCAG CCTCTCAGCG CTGCTGCTGC TGCTGCAGGT

Cow ATGGGCCCTC GGTGCCGCAA CCTCTCTGCG CTCCTGCTGC TGCTGCAGGT

Dog ---------- ---------- ---------- ---------- ----------

Elephant ---------- ---------- ---------- ---------- ----------

Gorilla ATGGGCTCTT GGAGCCGCAG CCTCTCGGTG CTGCTGCTGC TGCTGCAGGT

Guinea_Pig ---------- ---------- ---------- ---------- ----------

Horse ---------- ---------- ---------- ---------- ----------

Marmoset ATGGGCCCTT TGAGCCGCAG CCACTCCGCG CTGCTGCTGC TGCTGCAGGT

Mouse ATGGGAGCCC GGTGCCGCAG CTTTTCCGCG CTCCTGCTCC TGCTGCAGGT

Opossum ---------- ---------- ---------- ---------- ----------

Orangutan ATGGGCCCTT GGAGCCGCAG TCTCTCGGCG CTGCTGCTGC TGCTGCAGGT

Platypus ---------- ---------- ---------- ---------- ----------

Rabbit ATGGGTCCTC CACGCCGCGG TTCTCCCGCG CTGCTGCTGC TGCTGCAGGT

Rat ATGGGAGCCC GGTGCCGCAG CTTCTCCGCG CTCCTGCTCC TGCTGCAGGT

Human CTCCTCTTGG CTCTGCCAGG AGCCGGAGCC CTGCCACCCT GGCTTTGACG

Chimpanzee CTCCTCTTGG CTCTGCCAGG AGCCGGAGCC CTGCCACCCT GGCTTTGACG

Cow GTCATCGTGG CTCTGCCAGG AGCCGGAGCC CTGCATTCCT GGCTTTGGTG

Dog ---------- ---------- ---------- ---------- ----------

Elephant ---------- ---------- ---------- ---------- ----------

Gorilla TTCCTCTTGG CTCTGCCAGG AGCCGGAGCC CTGCCACCCT GGCTTTGACG

Guinea_Pig ---------- ---------- ---------- ---------- ----------

Horse ---------- ---------- ---------- ---------- ----------

Marmoset CTCGTATAGG GTCTGCCAAG AGCCGGAGTC CTGCCACCCC GGCTTTGATT

Mouse CTCCTCATGG CTTTGCCAGG AGCTG----- -TGCAGTCCC GGCTTCAGTT

Opossum ---------- ---------- ---------- ---------- ----------

Orangutan CTCCTCTTGG CTCTGCCAGG AGCCGGAGCC CTGCCACCCC GGCTTTGACG

Platypus ---------- ---------- ---------- ---------- ----------

Rabbit ATCCTCGACG CTCTGCCAGG AGCCCGAGCC CTGCCACCCC GGCTTTGGCG

Rat CTCCTCGTGG CTTTGTCAGC AGCCGGACTC CTGCCGTCCC GGCTTCAGTT

Human CCGAGAGCTA CACGTTCACG GTGCCCCGGC GCCACCTGGA GAGAGGCCGC

Chimpanzee CCGAGAGCTA CACGTTCACG GTGCCCCGGC TCCACCTGGA GAGAGGCCGC

Cow CTGAGAGTTA CACGTTCACC GTGCCCCGGC GGAACTTGGA GAGAGGGCGA

Dog ---------- ---------- ---------- ---------- ----------

Elephant ---------- ---------- ---------- ---------- ----------

Gorilla CCGAGAGCTA CACGTTCACG GTGCCCCGGC GCCACTTGGA GAGAGGCCGC

Guinea_Pig ---------- ---------- ---------- ---------- ----------

Horse ---------- ---------- ---------- ---------- ----------

Marmoset CCGAGAGCTA CACATTCACG GTGCCTCGGC GGCACCTGGA GAGAGGCCGC

Mouse CCGAGGTCTA CACCTTCCCG GTGCCGGAGA GGCACCTGGA GAGAGGCCAT

Opossum ---------- ---------- ---------- ---------- ----------

Orangutan CCGAGAGCTA CACGTTCACG GTGCCCCGGC GCCACCTGGA GAGAGGCCGC

Platypus ---------- ---------- ---------- ---------- ----------

Rabbit CCGAGAGCTA CAGGTTCACC GTGCCCCGGC GGCACTTGGA GAGAGGCCGA

Rat CCGAGGTCTA CACCTTCCTG GTGCCGGAGA GGCACCTGGA GAGAGGCCAC

Human GTCCTGGGCA GAGTGAATTT TGAAGATTGC ACCGGTCGAC AAAGGACAGC

Chimpanzee GTCCTGGGCA GAGTGAATTT TGAAGATTGC ACCGGTCGAC AAAGGACAGC

Cow GTCCTAGGCA GAGTGAGTTT TGAAGGATGC GCTGGCCTAC CAAGGACAGT

Dog ---------- --GTGAGTTT TGAAGGATGC ACCGGTCTAC CTAGGACAGC

Elephant ---------- --GTGAGCTT TGAAGGATGC ACTGGTCGAC CAAGGACAGC

Gorilla GTCCTGGGCA GAGTGAATTT TGAAGATTGC ACCGGTCGAC AAAGGACAGC

Guinea_Pig ---------- --GTGAGTTT TGAAGGATGC ACAGGCCGAC CAAGAACAGT

Horse ---------- ---------- ---------- ---------- ----------

Marmoset GTCCTGGGCA GAGTGAGTTT TGAAGATTGC ACTGGTCGAC CAAGGACAGC

Mouse GTCCTGGGCA GAGTGAGATT TGAAGGATGC ACTGGCCGGC CAAGGACAGC

Opossum ---------- --GTTACCTT TGAAAGCTGT TCTGGAAGAA AACTGAATGC

Orangutan GTCCTGGGCA GAGTGAATTT TGAAGATTGC ACGGGTCGAC AAAGGACAGC

Platypus ---------- --GTGAGTTT TGAAGACTGC TCCGGAAGCA GGAGAACAGC

Rabbit GTCCTGGGCA GAGTGTACTT CGAAGGCTGC ACAGGCCGAC CGAGGACGGC

Rat ATCCTGGGCA GAGTGAAATT TGAAGGATGC ACCGGCCGTC CAAGGACAGC

Human CTATTTTTCC CTCGACACCC GATTCAAAGT GGGCACAGAT GGTGTGATTA

Chimpanzee CTATTTTTCC CTCGACACCC GATTCAAAGT GGGCACAGAT GGTGTGATTA

Cow CTATGTTTCT GATGACACCC GATTCAAAGT GCACACAGAT GGCGTGCTTA

Dog CTATGTTTCT GATGACACCC GATTCAAAGT GGGCACAGAT GGTGTGATTA

Elephant CTATTTTTCT GAAGACACCC GATTCAAAGT GGCCACAGAT GGTGTGGTTA

Gorilla CTATTTTTCC CTCGACACCC GATTCAAAGT GGGCACAGAT GGTGTGATTA

Guinea_Pig TTACTATTCT TCTGACTCCC GATTCAAAGT GGGTACAGAT GGTGTAGTTA

Horse ---------- ---------- ---------- ---------- ---------A

Marmoset CTACTTTTCC GTCGACACCC GATTCAAAGT GGGCACAGAT GGCGTGATTA

Mouse CTTCTTTTCG GAAGACTCCC GATTCAAAGT GGCGACAGAC GGCACCATCA

Opossum TTATTTCTCT GAAGATTCCA GAATTAGAGT GACTCCTGAA GGTTTCGTAA

Orangutan CTATTTTTCC CTCGACACCC GATTCAAAGT GGGCACAGAT GGTGTGATTA

Platypus TTACCTTCTT GAAGACACAC GATTCAAAGT GGCAACCAAT GGAGTGGTTG

Rabbit CTACTTCTCT GACGACTCTC GCTTCCAAGT GGGCACGGAC GGCGTGATCA

Rat CTTCTTTTCT GAAGACTCCC GATTCAAAGT GTCTACAGAT GGCGTCATCA

Human CAGTCAAAAG GCCTCTACGG TTTCATAACC CACAGATCCA TTTCTTGGTC

Chimpanzee CAGTCAAAAG GCCTCTACGG TTTCATAACC CACAGATCCA TTTCTTGGTC

Cow CAGTCAGACG ACCTGTACAC CTTCATCGTC CAGAGCTAAG TTTTCTTGTC

Dog CAGTCAAGCG GCCTCTACAA CTTCATAAAC CAGAGATAAG TTTTCTTGTC

Elephant CGGTCAGATG GCCTGTACAA CTTCATAATC CAGAGACCAG CTTTCTTGTC

Gorilla CAGTCAAAAG GCCTCTACGG TTTCATAACC CACAGATCCA TTTCTTGGTC

Guinea_Pig CAGTCAAACG GCCCCTGCAG TTTCACAAAC CAGAGATCAG TTTTTCTATC

Horse TGGTCAAGCG GCCTCTACAG CTTCATAATC CGGAGATGAG TTTTCTTGTC

Marmoset CAGTCAAAAG GCCTGTACAA CTTCATAACC CACAGATCCA TTTTCTGGTC

Mouse CAGTGAAGCG GCATCTAAAG CTCCACAAGC TGGAGACCAG TTTCCTCGTC

Opossum GGGTCAAGAG ACCTCTACAG CTCCATAATT TAGAAACAAG CTTTTCTATC

Orangutan CAGTCAAAAG GCCTCTACGG TTTCATAACC CACAGATCCA TTTCTTGGTC

Platypus CAGTCAAGCA TCCTGTATGG CTTCATAACT TAGAAATGAG TTTCAACATC

Rabbit CCGTGAAGCG GCCCCTGCAG CTGCACAGGC CGGAAACCAG TTTCCACGTC

Rat CAGTCAAACG GCATCTAAAG CTTCACAAGC TGGAGACCAG TTTTCTCGTC

Human TACGCCTGGG ACTCCACCTA CAGAAAGTTT TCCACCAAAG TCACGCTGAA

Chimpanzee TACGCCTGGG ACTCCACCTA CAGAAAGTTT TCCACCAAAG TCACGCTGAA

Cow CATGCCTGGG ACTCCACCCA CAGGAAGCTC TCCACCAAAG TGACACTGGA

Dog CATGCCTGGG ACTCCAGCCG CAGGAAGCTC TCCACCAGAG TTAGGCTGAA

Elephant CATGCCTGGG ACTCCACCCG CAGAAAGCTT TCCACCAAAG TGACACTGAA

Gorilla TATGCCTGGG ACTCCACCTA CAGAAAGTTT TCCACCAAAG TCACGCTGAA

Guinea_Pig CATTCCTGGG ACTCTACCTT CAGAAAGTTT TCCACTGAAG TCACACTGAA

Horse CATGCCTGGG ACTCCAGCCG CAGGAAGCTC TCTACCAAGG TTACCGTGAA

Marmoset TACGCCTGGG ACTCCACCTA CAGAAAGTTT TCCACCAAAG TCACGCTGCA

Mouse CGCGCCCGGG ACTCCAGTCA TAGGGAGCTG TCTACCAAAG TGACGCTGAA

Opossum CAATCCTGGG ATGCTACAGG TAAAAAGTTC TTAACCAAAC TGATACTGAA

Orangutan TACGCCTGGG ACTCCACCTA CAGAAAGTTT TCCACCAAAG TCACGCTGAA

Platypus CATGCTTGGG ATGCCACTGG CAACAAGCTC TCTACTAAAG TTACCCTGAA

Rabbit CACGCCTGGG ACTCCAGCTA CAGGAAGCTC TCCACCAAGG TCACGCTGAA

Rat CATGCCTGGG ACTCCAGTTA CAGGAAGCTT TCTACCAAAG TGACACTGAA

Human TACAGTGGGG CACCACCACC GCCCCCCGCC CCATCAGGCC TCCGTTTCTG

Chimpanzee TACAGTGGGG CACCACCACC GCCCCCCGCC CCATCAGGCC TCTGTTTCTG

Cow GGTATCAGCG CACCACCACC ACCACCACAG TCATCATGAC TCTCCCTCTG

Dog GGCAGCGACG CACCACCACC AC------CA CCATCATGAT GCTCCCTCTA

Elephant GGTCACTGGG CACCACCGAC ACCACCAACA GCATCATGAC TCTCTCTCTG

Gorilla TACAGTGGGG CACCACCACC GCCCCCCGCC CCATCAGGCC TCTGTTTCTG

Guinea_Pig GGCAGAGGGA CGGCACCATC GCAAGCATTC GAGCCACGCA GCCCAGCCTT

Horse GGCAGCCACG CACGAGCACC ACCACCACCA CCACCATGAG GCTGTCTCTG

Marmoset GGCAGTGGGG CAC------- --------CC CCATCATGAC TCTGTTCCTG

Mouse GTCCATGGGG CACCACCATC ACCGGCACCA CCACCGCGAC CCTGCCTCTG

Opossum GAGATCACCA AAACACCAGC GGCACCTCCA TCATGGAGAG GAATTCCCAG

Orangutan TACAGTGGGG CACCACCACC GCCCCCTGCC GCATCAGGCC TCTGTTTCTG

Platypus AAGAGCAGCA CAACACCATC ACCAGCACTG TGCTCGTGAG TCTTCTCCTG

Rabbit GGCCGTGGGG CCGCACCGCA ACCACCACCA TCGCCATGAG TCTTCTCCTG

Rat GTCCCTGGGC CACCACCACC ACCGGCATCA CCACAGAGAC CCTGTCTCTG

Human GAATCCAAGC AGAATTGCTC ACATTTCCCA ACTCCTCTCC TGGCCTCAGA

Chimpanzee GAATCCAAGC AGAATTGCTC ACATTTCCCA ACTCCTCTCC TGGCCTCAGA

Cow GAACCCAGAC GGAAGTGCTC ACATTTCCTG GTCCCCACCA TGGTCTCAGG

Dog AAACCCAGAC AGAGGTGCTC ACATTTCCCA GTTCCCAGCA TGGACTCAGA

Elephant GACCCCAGAC AGAAGTGCTC ATGTTTCCCG ACTTCCATCA TGGGCTCCGA

Gorilla GAATCCAAGC AGAATTGCTC ACATTTCCCA ACTCCTCTCC TGGCCTCAGA

Guinea_Pig TGACTCAGCT AGAAGTGATC AAATTTCCCA ACTTCCACCA TGGTCTCAGA

Horse GAACCCATAC AGAAGTGCTC ACATTTCCCA GCTCCCACCA TGGTCTTAGA

Marmoset AATCCCACAT GGAAGTGCTC ACATTTCCCA ACTCCTCTCA TAGCCTCAGA

Mouse AATCCAACCC AGAGCTGCTC ATGTTTCCCA GCGTGTACCC AGGTCTCAGA

Opossum CAGAAGAGAG AGAAGTGCTG ATATTCCCTG CCACCCACTC TGGTCTCAAG

Orangutan GAATCCAAGC AGAATTGCTC ACATTTCCCA ACTCCTCTCC TGGCCTCAGA

Platypus GAGAACATTC AAAAGTGCTG CTGTTCCCTC AATCCCACTC TGGTCTGAAG

Rabbit GAGCCAGCAC GGAAGTGCTC ACGTTTCCCG ACTCCCACCA TGGTCTCAGA

Rat AATCCAACCC AGAGCTGCTC ACGTTTCCCA GCTTTCACCA GGGTCTGAGA

Human AGACAGAAGA GAGACTGGGT TATTCCTCCC ATCAGCTGCC CAGAAAATGA

Chimpanzee AGACAGAAGA GAGACTGGGT TATTCCTCCC ATCAGCTGCC CAGAAAATGA

Cow AGACAGAAGA GAGACTGGGT TATTCCTCCT ATCAGCTGCC CAGAAAATGA

Dog AGACAGAAGA GAGACTGGGT TATCCCTCCT ATCAGCTGCC CGGAAAACGA

Elephant AGACAGAAGA GAGACTGGGT TATCCCTCCT ATTAGCTGCC CAGAAAATGA

Gorilla AGACAGAAGA GAGACTGGGT TATTCCTCCC ATCAGCTGCC CAGAAAATGA

Guinea_Pig AGGCAGAAGA GAGACTGGGT TATCCCTCCC ATCAGCTGCT CAGAAAATGA

Horse AGACAGAAGA GAGACTGGGT TATCCCTCCT ATCAGCTGCC CAGAAAATGA

Marmoset AGACAGAAGA GAGACTGGGT TATCCCTCCC ATCAGCTGCC CAGAAAATGA

Mouse AGACAGAAAC GAGACTGGGT CATCCCTCCC ATCAGCTGCC CCGAAAATGA

Opossum AGGCAGAAGA GAGACTGGGT TATCCCTCCT ATTAGCTGCC CTGAGAATGA

Orangutan AGACGGAAGA GAGACTGGGT TATTCCTCCC ATAAGCTGCC CAGAAAATGA

Platypus AGACAGAAGA GGGACTGGGT TATCCCTCCC ATCAACTGTC CTGAGAATGA

Rabbit AGACAGAAGA GGGACTGGGT TATTCCTCCC ATCAGTTGCC CAGAAAATGA

Rat AGACAGAAAC GAGACTGGGT CATCCCTCCC ATCAACTGCC CGGAAAATCA

Human AAAAGGCCCA TTTCCTAAAA ACCTGGTTCA GATCAAATCC AACAAAGACA

Chimpanzee AAAAGGCCCA TTTCCTAAAA ACCTGGTTCA GATCAAATCC AACAAAGACA

Cow GAAAGGCCCG TTTCCTAAGT CGCTGGTCCA GATCAAATCT AACAAGGAGA

Dog GAAAGGCCCA TTTCCTAAAA ACCTGGTTCA GATCAAGTCT AACAGGGACA

Elephant AAAAGGCCCA TTTCCTAAAA ACCTGGTTCG GATCAAATCT AGCAGGGACA

Gorilla AAAAGGCCCA TTTCCTAAAA ACCTCGTTCA GATCAAATCC AACAAAGACA

Guinea_Pig AAAAGGCCCG TTTCCTAAAA GACTGGTTCA GATCAAATCC AACAGGGACA

Horse GAAAGGCCCA TTTCCTAAGA ATCTGGTTCA GATAAAATCT AACAGGGACA

Marmoset AAAAGGCCCA TTTCCTAAAA ACCTGGTTCA GATCAAATCC AACAAAGACA

Mouse AAAGGGCGAA TTTCCAAAGA ACCTGGTTCA GATCAAATCC AACAGGGACA

Opossum GAAGGGTCCA TTCCCTAAAC AACTGGTGCA GATCAAGTCA AACCGAGATA

Orangutan AAAAGGCCCG TTTCCTAAAA ACCTGGTTCA GATCAAATCC AACAAAGACA

Platypus GAAGGGACCG TTCCCTAAAA ATCTGGTTCA GATCAAATCC AACAGAGACA

Rabbit AAAAGGCCCA TTTCCTAAAA ACCTGGTCCA GATCAAGTCC AACAGGGACA

Rat AAAGGGCGAA TTCCCCCAGC GACTGGTTCA GATCAAATCC AACAGGGACA

Human AAGAAGGCAA GGTTTTCTAC AGCATCACTG GCCAAGGAGC TGACACACCC

Chimpanzee AAGAAGGCAA GGTTTTCTAC AGCATCACTG GCCAAGGAGC TGACACACCC

Cow AAGAAACCCA AGTTTTCTAC AGCATCACTG GCCAAGGAGC TGATACACCC

Dog AAGAAATCAA GGTTTTCTAC AGCATCACTG GCCAAGGAGC TGACGCACCT

Elephant AAGAAATTGA GGTTTTCTAC AGCATCACTG GTGAAGGAGC TGATAGACCT

Gorilla AAGAAGGCAA GGTTTTCTAC AGCATCACTG GCCAAGGAGC TGACACACCC

Guinea_Pig AAGAAACCAA GGTTTTCTAC AGCATCACTG GCCAAGGAGC TGACACGCCT

Horse AAGAAACCAA GGTTTTCTAT AGCATCACTG GCCAAGGAGC CGACACACCC

Marmoset AAGAAGCCAA GGTTTTCTAT AGCATCACTG GCCAAGGAGC TGACACGCCC

Mouse AAGAAACAAA GGTTTTCTAC AGCATCACCG GCCAAGGAGC TGACAAACCC

Opossum AAGAAACTAA AGTTTTCTAC AGCATCACAG GCCAAGGAGC AGATAGACCT

Orangutan AAGAAGGCAA GGTTTTCTAC AGCATCACTG GCCAAGGAGC TGACACACCC

Platypus AGGAGACCAG GGTTTACTAC AGTATCACTG GCCAAGGAGC AGATAAGCCT

Rabbit AGGAGACCCA GGTCTTCTAC AGCATCACCG GCCAGGGAGC CGACACGCCG

Rat AAGAGACAAC GGTTTTCTAC AGCATCACCG GCCCAGGAGC TGACAAACCC

Human CCTGTTGGTG TCTTTATTAT TGAAAGAGAA ACAGGATGGC TGAAGGTGAC

Chimpanzee CCTGTTGGTG TCTTTATTAT TGAAAGAGAA ACAGGATGGC TGAAGGTGAC

Cow CCTGTCGGTG TTTTTATTAT TGAAAGAGAA ACAGGATGGT TAAAAGTGAC

Dog CCTGTTGGTG TGTTTATTAT TGAAAGAGAA ACAGGATGGC TGAAGGTGAC

Elephant CCTGTCGGTG TGTTCGTTAT TGAAAGAAAA ACAGGATGGC TGAAGGTGAC

Gorilla CCTGTTGGTG TCTTTATTAT TGAAAGAGAA ACAGGATGGC TGAAGGTGAC

Guinea_Pig CCTGTTGGGG TGTTTATTAT TGAAAGAGAA ACAGGATGGC TGATGGTGAC

Horse CCTGTTGGTG TGTTTATTAT TGAAAGAGAA ACAGGATGGT TAAAGGTGAC

Marmoset CCTGTTGGTG TCTTTATTAT TGAAAGAGAA ACAGGGTGGC TGAAAGTGAC

Mouse CCCGTTGGCG TTTTCATCAT TGAGAGGGAG ACAGGCTGGC TGAAAGTGAC

Opossum CCACAAGGTG TCTTTATTAT TGAAAGAGAA ACCGGAATCT TGAAAGTGAC

Orangutan CCTGTTGGTG TCTTTATTAT TGAAAGAGAA ACAGGATGGC TGAAGGTGAC

Platypus CCCATGGGGA TCTTCACTAT TGAAAGAGAA TCTGGGTGGC TGAAAGTGAC

Rabbit CCTGTTGGTG TCTTCATCAT CGAGAGGGAG ACGGGATGGC TGAAGGTGAC

Rat CCTGTTGGCG TTTTCATCAT TGAGAGGGAG ACAGGCTGGC TGAAAGTGAC

Human AGAGCCTCTG GATAGAGAAC GCATTGCCAC ATACACTCTC TTCTCTCACG

Chimpanzee AGAGCCTCTG GATAGAGAAC GCATTGCCAC ATACACTCTC TTCTCTCATG

Cow ACAGCCTCTG GATAGAGAAC AGATTGCCAA GTACATTCTC TTCTCTCATG

Dog TGAGCCTCTG GATAGAGAAC AAATTGCTAA GTACATTCTC TACTCTCATG

Elephant GCGGCCTCTG GATAGAGAAC AAATTTCCAA TTACACTCTC TTGTCTCATG

Gorilla AGAGCCTCTG GATAGAGAAC GCATTGCCAC ATACACTCTC TTCTCTCATG

Guinea_Pig AGAGCCTCTG GATAGAGAAA AAATTGCCAA CTACACTCTC TTATCTCACG

Horse AGAGCCTCTG GATAGAGAAG AAATTTCCAA GTACATTCTC TTCTCTCATG

Marmoset AGAGCCTTTG GATAGAGAAC AAATTGCCAC CTACACTCTC TTCTCCCATG

Mouse ACAGCCTCTG GATAGAGAAG CCATTGCCAA GTACATCCTC TATTCTCATG

Opossum AGCACCTTTG GACAGAGAAG ACATTGCAAA ATATGTCCTT TTCTCTCATG

Orangutan AGAGCCTCTG GATAGAGAAC GCATTGCCAC ATACACTCTC TTCTCTCATG

Platypus GGAACCGCTG GACAGAGAAA AAATTCCAAA ATACATTCTT CAATCCCATG

Rabbit GGAGCCTCTG GACCGAGAAC ACATCGCCAA GTACACGCTC TTCTCTCACG

Rat GCAGCCTCTG GACAGAGAAG CCATTGACAA GTACCTTCTC TACTCTCATG

Human CTGTGTCATC CAACGGGAAT GCAGTTGAGG ATCCAATGGA GATTTTGATC

Chimpanzee CTGTGTCATC CAACGGGAAT GCCGTTGAGG ATCCAATGGA GATTTTGATC

Cow CCGTGTCTTC AAACGGACAA GCCATTGAAG AGCCTATGGA GATTGTGATC

Dog CCGTATCTTC TAATGGGAAT GCGGTTGAAG ACCCAATGGA GATCGTGATC

Elephant CTGTGTCATC AAATGGAGAT GCAGTCGAAG ACCCAATGGA GATTGTGATT

Gorilla CTGTGTCATC CAACGGGAAT GCGGTTGAGG ATCCAATGGA GATTTTGATC

Guinea_Pig CCGTGTCATC AAACGGGAAT GCAGTTGAGG ATCCCATGGA GATTGTGATC

Horse CTGTGTCTTC GAATGGGAAT GCAATCGAGG ACCCGATGGA GATTGTGATC

Marmoset CTGTGTCATC CAATGGGAAT GCTATTGAGG ACCCAATGGA GATTGTGATA

Mouse CCGTGTCATC AAATGGGGAA GCGGTGGAGG ATCCCATGGA GATAGTGATC

Opossum CTGTGTCTTC AAATGGCATA CCTGTGGAGG ATCCAATGGA GATTGTGATT

Orangutan CTGTGTCATC CAACGGGAAC GCAGTTGAGG ACCCAATGGA GATTTTGATC

Platypus CAGTGACTTC TGATGGGAAG ACCGTGGAAG AACCTATGGA GATTGTGATT

Rabbit CCGTGTCGTC TAACGGGAAC GCCATTGAGG ACCCGATGGA GATCGTGATC

Rat CTGTGTCATC AAATGGGGAA GCCGTGGAGG ATCCCATGGA GATAGTGGTC

Human ACGGTAACCG ATCAGAATGA CAACAAGCCC GAATTCACCC AGGAGGTCTT

Chimpanzee ACGGTGACCG ATCAGAATGA CAACAAACCC GAATTCACCC AGGAGGTCTT

Cow ACCGTGACCG ACCAGAATGA CAACAAGCCC CAGTTCACCC AGGAGGTCTT

Dog ACGGTGACAG ATCAGAATGA CAACAAGCCC GAGTTCACCC AGGCAGTCTT

Elephant ACCGTGACAG ATCAGAACGA CAATAAGCCC GTATTCACCC AGGCTGTCTT

Gorilla ACGGTGACCG ATCAGAATGA CAACAAGCCC GAATTCACCC AGGAGGTCTT

Guinea_Pig ACAGTGACAG ATCAGAATGA CAACAGGCCT CAGTTCATCA AGGAGGTCTT

Horse ACGGTGACAG ATCAGAATGA CAACAAGCCC GAGTTCACCC AGCTGGTCTT

Marmoset ACCGTGACTG ATCAGAATGA CAACAAGCCC GAGTTCACCC AGGAGGTCTT

Mouse ACAGTGACAG ATCAGAATGA CAACAGGCCA GAGTTTACCC AGCCGGTCTT

Opossum ACAGTAACAG ATCAGAATGA TAATCGTCCT GAGTTTACTC AGGAGGTCTT

Orangutan ACAGTGACCG ATCAGAATGA CAACAAGCCC GAATTCACCC AGGAGGTCTT

Platypus ACCGTGACTG ATCAGAATGA CAACAAGCCC CAGTTCACCC AGGAGGTCTT

Rabbit ACGGTGACGG ACCAGAACGA CAACAGGCCT GAGTTCACCC AGGAGGTCTT

Rat ACAGTCACAG ATCAGAATGA CAACAGGCCA GAGTTTATCC AGGAGGTCTT

Human TAAGGGGTCT GTCATGGAAG GTGCTCTTCC AGGAACCTCT GTGATGGAGG

Chimpanzee TAAGGGGTCT GTCATGGAAG GTGCTCTTCC AGGAACCTCT GTGATGGAGG

Cow CAAGGGGTCT GTCCTGGAAG GCGCTCTTCC AGGAACCTCT GTGATGCAGG

Dog CCAAGGATCT GTCACGGAAG GTGCCCTTCC AGGCACCTCT GTGATGCAGG

Elephant TCAAGGGTCT GTCATGGAAG GCGCTGTCCC AGGAACCTCA GTGATGCAGG

Gorilla TAAGGGGTCT GTCATGGAAG GTGCTCTTCC AGGAACCTCT GTGATGGAGG

Guinea_Pig TGAGGGATCT GTCATGGAAG GCGCTCTTCC AGGGACCTCC GTGATGCAGG

Horse TGAAGGGTCT GTCATGGAAG GTGCTCTTCC AGGAACCTCT GTGATGCAGG

Marmoset TCACGGGTCT GTCATGGAAG GTGCTCTTCC AGGAACCTCC GTGATGGAGG

Mouse TGAGGGATTC GTTGCAGAAG GCGCTGTTCC AGGAACCTCC GTGATGAAGG

Opossum CAATGGGTTC GTGATGGAAG GAGCTCATCC AGGTACCTCT GTGATGAAAG

Orangutan TAAGGGGTCT GTCATGGAAG GTGCTCTTCC AGGAACCTCT GTGATGGAGG

Platypus TGAGGGTTCA GTAGTCGAAG GATCCTCTCC AGGAACCTCT GTGATGCAAG

Rabbit CAAGGGCTCG GTCATGGAAG GCGCGCTACC AGGAACGTCC GTGATGCAGG

Rat TGAGGGATCT GTTGCAGAAG GCGCTCTTCC AGGAACCTCC GTGATGCAGG

Human TCACAGCCAC AGACGCGGAC GATGATGTGA ACACCTACAA TGCCGCCATC

Chimpanzee TCACAGCCAC AGACGCGGAC GATGATGTGA ACACCTACAA TGCCGCCATC

Cow TCACGGCCAC AGATATAGAT GACGAGGTGA ACACCTACAA CGCTGCCATC

Dog TGACAGCCAC AGATGCGGAT GATGATGTGA ATACCTACAA CGCTGCCATC

Elephant TCACAGCCAC AGACGCGGAT GACGATGTGA ACACCTACAA TGCTGCCATC

Gorilla TCACAGCCAC AGACGCGGAC GATGATGTGA ACACCTACAA TGCCGCCATC

Guinea_Pig TCTCAGCCAC TGACGCAGAT GAT---CCTA ACACCTACAA TGCTGAAATC

Horse TCACGGCCAC AGATGCAGAC GATGACGTGA ATACCTACAA TGCTGCCATT

Marmoset TCTCAGCCAC AGACGCAGAC GATGATGTGA ACACCTACAA TGCCGCCATC

Mouse TCTCAGCCAC CGATGCAGAC GATGACGTCA ACACCTACAA CGCTGCCATC

Opossum TGTCGGCCAC TGATAAAGAT GATGATGTGA ACACCTACAA TGCTATGATC

Orangutan TCACAGCCAC AGACGCGGAC GATGATGTGA ACACCTACAA TGCTGCCATC

Platypus TCAACGCCAC AGATAAGGAT GAT---CCGA ACACTTACAA TGCAGCGCTT

Rabbit TCACGGCTAC GGACGCGGAC GATGACGTGA ACACCTACAA TGCTGCCATC

Rat TCTCAGCCAC TGATGCAGAC GATGACATAA ACACCTACAA TGCTGCCATC

Human GCTTACACCA TCCTCAGCCA AGATCCTGAG CTCCCTGACA AAAATATGTT

Chimpanzee GCTTACACCA TCCTCAGCCA AGATCCTGAG CTGCCTGACA AAAATATGTT

Cow GGTTACAAAA TCCTAGCCCA AGATCCCATG CTGCCGCACA ACAAAATGTT

Dog GCTTACAGCA TCCTCACACA AGACCCCCTC CTGCCTAGCA GCATGATGTT

Elephant GTTTATACCA TTGAAAACCA GGAGCCTACC TTGCCTCATG ACAACATGTT

Gorilla GCTTACACCA TCCTCAGCCA AGATCCTGAG CTGCCTGACA AAAATATGTT

Guinea_Pig GCTTACACCA TCCTCAGCCA AGAGCCCACA TTGCCTCACA GCCAAATGTT

Horse GTGTACTCCA TCCTCAGCCA AGAGCCTATG CTGCCTCACA AGCAAATGTT

Marmoset GCTTACACCA TCCTCAGCCA AGCGCCTGAG CTGCCTTACC AAAAGATGTT

Mouse GCCTACACCA TCGTCAGCCA GGATCCTGAG CTGCCTCACA AAAACATGTT

Opossum GCTTATTCCA TTCTCAGCCA GGAGCCCAGG TCTCCCCATG ATGGTATGTT

Orangutan GCTTACACCA TCCTCAGCCA AGATCCTGAG CTGCCTGACA AAAATATGTT

Platypus GTTTACTCTA TCGTCAGCCA GGATCCTAAA GATCCTCAGG ATAACATATT

Rabbit GCCTACGAAA TCCTCAGCCA GGAGCCCGAG CTGCCGCACA AGAACATGTT

Rat GCCTACACCA TCCTCAGCCA AGATCCTGAG CTGCCTCACA AAAACATGTT

Human CACCATTAAC AGGAACACAG GAGTCATCAG TGTGGTCACC ACTGGGCTGG

Chimpanzee CACCATTAAC AGGAACACAG GAGTCATCAG TGTGGTCACC ACTGGGCTGG

Cow CACCATCAAC AAGGAAACAG GCGTCATCAG TGTGCTCACC ACCGGGCTGG

Dog CACTATCAAC AAGGACACAG GAGTCATCAG CGTGCTCACC ACTGGGCTGG

Elephant CACCATCAAC CGGGACACAG GAGTCATTAG TGTGGTCACC AGTGGGCTGG

Gorilla CACCATTAAC AGGAACACAG GAGTCATCAG TGTGGTCACC ACTGGGCTGG

Guinea_Pig TACCATCAAC CGGATCACAG GGGTCATCAG TGTGGTTACC ACCGGGCTGG

Horse CACCATCAAC AGGGACACGG GAGTCATCAG TGTGCTCACC ACCGGGCTGG

Marmoset CACAATTAAT AAGGACACAG GAGTCATCAG TGTGCTCACC ACCGGGCTGG

Mouse CACTGTCAAT AGGGACACCG GGGTCATCAG TGTGCTCACC TCTGGGCTGG

Opossum CACCATCAAC CGGGGAACTG GAGTCATCAG CGTGCTCACA ACTGGCCTAG

Orangutan CACCATTAAC AGGAACACAG GAGTCATCAG TGTGGTCACC ACTGGGCTGG

Platypus CACCATTAAT CCAACTTCCG GCATGATCAC TTTGCTCACA TCAGGACTGG

Rabbit CAGGATCAAC CCGCAGACCG GAGTCATCAG TGTGGTCACC ACCGGGCTGG

Rat CACTGTCAAC CGGGACACTG GGGTCATCAG TGTGGTCACC TCCGGACTGG

Human ACCGAGAGAG TTTCCCTACG TATACCCTGG TGGTTCAAGC TGCTGACCTT

Chimpanzee ACCGAGAGAG TTTCCCTACG TATACCCTGG TGGTTCAAGC TGCTGATCTT

Cow ACCGTGAGAG TTTTCCCACA TACACCCTGA TGGTCCAAGC AGCAGACCTT

Dog ACCGAGAGGG TGTCCCCATG TACACCTTGG TGGTTCAGGC TGCTGACCTG

Elephant ATCGAGAGAG TGTTCCCAAG TATACCCTGG TGGTTAAAGC TACTGACCTT

Gorilla ACCGAGAGAG TTTCCCTACG TATACCCTGG TGGTTCAAGC TGCTGACCTT

Guinea_Pig ACCGAGAGAG TTATCCCACA TATACCCTGG TGGTTCAAGC TGCTGACCTT

Horse ACCGGGAGAG TTTTCCCACA TATACCCTAG TGGTCCAGGC TGCTGACCTT

Marmoset ACCGAGAGAG TTTCCCTACA TATACGCTGG TGGTCCAGGC TGCTGACCTT

Mouse ACCGAGAGAG TTACCCTACA TACACTCTGG TGGTTCAGGC TGCTGACCTT

Opossum ACAGAGAGAC CAACCCACAA TATACACTGA TTGTCCAAGC TGCTGACTTA

Orangutan ACCGAGAGAG TTTCCCTACA TATACCCTGG TGGTTCAGGC TGCTGACCTT

Platypus ACAGAGAGAC TATCCCGCAG TATAAACTGA TTGTACAAGT TGCTGACATG

Rabbit ACCGAGAGAC GTACCCCGAG TACACCCTGG TCGTTCAGGC TGCTGACCTC

Rat ACCGAGAGAG TTACCCTACA TATACTCTGG TGGTTCAGGC TGCCGACCTT

Human CAAGGTGAGG GGTTAAGCAC AACAGCAACA GCTGTGATCA CAGTCACTGA

Chimpanzee CAAGGTGAGG GGTTAAGCAC AACAGCAACA GCTGTGATCA CAGTCACTGA

Cow AACGGCGAAG GCTTGAGCAC AACTGCAACG GCCGTGATCA CAGTCTTGGA

Dog CAAGGCGAAG GCTTAACTAC AACTGCAACA GCTGTGATCA CAGTCACTGA

Elephant CAAGGTTTGG GCCTGAGTAC ATCAGCAACA GCTATGATTA CAGTCAGGGA

Gorilla CAAGGTGAGG GGTTAAGCAC AACAGCAACA GCTGTGATCA CAGTCACTGA

Guinea_Pig CAAGGTGAAG GCTTAAGCGC GATAGCAAAA GCCGTGATCA CAGTGACTGA

Horse CAAGGTGAGG GCTTAAGTAC AACTGCCACA GCCGTGATCA CAGTCACTGA

Marmoset CAAGGAGAAG GGTTAAGCAC AACAGCAACA GCTGTGATCA CAGTCACTGA

Mouse CAAGGTGAAG GCTTGAGCAC AACAGCCAAG GCTGTGATCA CTGTCAAGGA

Opossum GATGGAGAGG GCCTAAGCAC CACAGCCACA GCGGTGATTA CGGTCACGGA

Orangutan CAAGGTGAGG GGTTAAGCAC AACAGCGACA GCTGTGATCA CAGTCACGGA

Platypus AATGGCGAAG GCTTAAGTAC AACGGCTACA GCAGTAATAA ATGTTGCCGA

Rabbit CAAGGCGAGG GCTTGAGCAC CACGGCGGAA GCGGTGATCA CGGTCACTGA

Rat CAAGGCGAAG GCCTAAGCAC AACAGCAAAA GCTGTGATCA CTGTCAAGGA

Human CACCAACGAT AATCCTCCGA TCTTCAATCC CACCACGTAC AAGGGTCAGG

Chimpanzee CACCAACGAT AACCCTCCGA TCTTCAATCC CACCACGTAC AAGGGTCAGG

Cow CACCAATGAT AATGCTCCCA GATTCAACCC AACCACGTAC GTGGGGTCGG

Dog CATCAATGAT AACCCCCCCA TCTTCAACCC AACCACGTAC CAGGGACGGG

Elephant CAACAACGAC AACCCTCCCA TCTTCAACCC GACAATGTAC GAAGGTACGG

Gorilla CACCAACGAT AACCCTCCGA TCTTCAATCC CACCACGTAC AAGGGTCAGG

Guinea_Pig CGTCAACGAT AACCCTCCCA TCTTCAACCC GACCATGTAT GAGGGTCAAG

Horse CGCCAATGAC AACCCTCCCG TCTTCAACCC CACCTTGTAC GAGGGGCAGG

Marmoset TGCCAACGAT AACCCTCCGA TCTTCAATCC CACCACGTAC CAGGGTCAGG

Mouse TATTAATGAC AACGCTCCTG TCTTCAACCC AAGCACGTAT CAGGGTCAAG

Opossum CACAAATGAC AATCCTCCTA TCTTCAACCC AACCACGTAT GTAGGCACAG

Orangutan CACCAACGAT AACCCTCCGG TCTTCAATCC CACCACGTAC AAGGGTCAGG

Platypus CAGCAATGAT AACCCACCTA TCTTCGACTC CCCAGTGTAT GTAGGGCAGG

Rabbit CATCAACGAC AACCCTCCCG TCTTCAACCC CACCACGTAC GTGGGCCAGG

Rat TATTAATGAC AACGCTCCCA TCTTCAACCC AAGCACGTAC CAGGGTCAAG

Human TGCCTGAGAA CGAGGCTAAC GTCGTAATCA CCACACTGAA AGTGACTGAT

Chimpanzee TGCCTGAGAA CGAGGCTAAC GTCATAATCA CCACACTGAA AGTGACTGAT

Cow TGCCTGAGAA CGAGGCTAAT GTGGCCATCA CCACACTCAC AGTGACTGAT

Dog TGCCTGAGAA CAAGGCTAAC GTCGAAATCG CTGTACTCAA AGTGACGGAT

Elephant TTCCTGAGAA TAAGGCCAAT GCTGATATCA CCAGGCTCAA AGTGACTGAT

Gorilla TGCCTGAGAA CGAGGCTAAC GTCGTAATCA CCACACTGAA AGTGACTGAT

Guinea_Pig TGCCTGAGAA TGAGGCCGAT GCAATCATTA CTACGCTCAA AGTGACTGAT

Horse TGCCTGAGAA CCAGGCTGAT GTCCTAATCA CCAGACTCAA AGTGACGGAT

Marmoset TGCCCGAGAA CGAGGCTAAC TTCAAAATCG CCACACTGAA AGTGACTGAT

Mouse TGCCTGAGAA TGAGGTCAAT GCCCGGATCG CCACACTCAA AGTGACCGAT

Opossum TGTATGAGAA TGAAGCAGAT GCCATCATTA CAAAATTGAA AGTGACGGAT

Orangutan TGCCTGAGAA CGAGGCTAAC GTCGTAATCA CCACACTGAA AGTGACTGAT

Platypus TGCGGGAGAA TGAGGTAAAT GCTGAAATTG TAAGACTAAA AGTGACTGAT

Rabbit TGCCCGAGAA CGAGGCCAAC TACCTGGTTG CCACGCTCAA AGTGACCGAC

Rat TGCTTGAGAA TGAGGTCGGT GCCCGTATTG CCACACTCAA GGTGACTGAT

Human GCTGATGCCC CCAATACCCC AGCGTGGGAG GCTGTATACA CCATATTGAA

Chimpanzee GCTGATGCCC CCAATACCCC AGCGTGGGAG GCTGTATACA CCATATTGAA

Cow GCCGACGACC CCAACACCCC GGCATGGGAG GCTGTTTACA CAGTATTAAA

Dog GCTGATGTCC CCGATACCCC GGCCTGGAGG GCTGTGTACA CCATATTGAA

Elephant GCTGATGTCC CTGACACCCC AGCGTGGAAG GCCAAGTACA ACATATTAAA

Gorilla GCTGATGCCC CCAATACCCC AGCGTGGGAG GCTGTATACA CCATATTGAA

Guinea_Pig GATGATGCCC CCAATACCCC AGCGTGGCAG GCCGTGTACA CCATCTTGAA

Horse GCCGACGTCC CCAACACCCC GGCATGGGAG GCCGTGTACA CCATATTAAA

Marmoset GCTGACGCCC CCAACACCCC GGCGTGGGAG GCTGTATACA CCATATTGAA

Mouse GATGATGCCC CCAACACTCC GGCGTGGAAA GCTGTGTACA CCGTAGTCAA

Opossum GCAGATGTTC CCAACACACC AGAATGGAAT GCTGTATATT CTGTTCTAAA

Orangutan GCTGATGCCC CCAGTACCCC AGCATGGGAG GCTGTATACA CCATATTGAA

Platypus GCTGACGTCC CTAATACCAA AGCATGGAAA GCAGTGTACT CTGTTAATAA

Rabbit AAAGATGAGC CTAACACCCC GGCCTGGGAG GCTGTGTACA CCGTATTGAA

Rat GATGATGCCC CCAACACTCC AGCGTGGAAT GCTGTGTACA CCGTAGTCAA

Human TGATGATGGT GGACAATTTG TCGTCACCAC AAATCCAGTG AACAACGATG

Chimpanzee TGATGATGGT GGACAATTTG TCGTCACCAC AAATCCAGTG AACAACGATG

Cow TGATAACGAG AAGCAATTTA TCGTCGTCAC AGACCCAGTC ACCAATGAAG

Dog CAATAACAAT GATCAATTTG TTGTCACCAC AGACCCAGTA ACTAACGACG

Elephant TGATAATGAG AAGCAATTTG TTGTCATCAC AGATCCAGTA ACGAATGAAG

Gorilla TGATGATGGT GGACAATTTG TCGTCACCAC AAATCCAGTG AACAACGATG

Guinea_Pig TGATCCGGAC AAGCAATTCA TTGTCACCAC AGACCCAATA ACCAACAATG

Horse TGATAATGAG CAGCAATTTG TTGTCACCAC AGACCCAGTA ACCAATGAAG

Marmoset TGATAATGAG GGGCAATTTG TCGTCACCAC AGATCCAGTG ACCAACGATG

Mouse CGATCCTGAC CAGCAGTTCG TTGTTGTCAC AGACCCCACG ACCAATGATG

Opossum TGATGAACAG GGTCTCTTTG CCGTTATCAC TGACCCCAAA ACCAATGAAG

Orangutan TGATAATGGT GGACAATTTG TCGTCACCAC AAATCCAGTG AACAACGATG

Platypus TGAA---GAC GGTTTCTTTG TTGTCACCAC TGACCCAGGA ACCAATGAAG

Rabbit CGATCCTGAG GGCCAGTTCA TTGTCACCAC GGACCCGGCC ACCAATGACG

Rat TGATCCTGAT CATCAGTTCA CTGTCATCAC AGACCCCAAG ACCAACGAGG

Human GCATTTTGAA AACAGCAAAG GGCTTGGATT TTGAGGCCAA GCAGCAGTAC

Chimpanzee GCATTTTGAA AACAGCAAAG GGCTTGGATT TTGAGGCCAA GCAGCAGTAC

Cow GCACTCTGAA AACAGCTAAG GGCTTGGATT TTGAGGCCAA GCAGCAGTAC

Dog GCATTTTGAA AACAACTAAG GGCTTGGATT TTGAGGACAA GCAGCAGTAT

Elephant GCATTCTGAG AACAGCTAAG GGCTTAGATT TTGAGGCCAA GCAGCAGTAC

Gorilla GCATTTTGAA AACAGCAAAG GGCTTGGATT TTGAGGCCAA GCAGCAGTAC

Guinea_Pig GCATTCTGAA AACAACTAAG GGATTGAATT TTGAGGCCAA GCAGCAATAC

Horse GCATTTTGAA AACAGCTAAG GGCTTAGATT TTGAGGCCAA GCAGCAGTAT

Marmoset GCATTTTGAA AACAGCAAAG GGCTTGGATT TTGAGGCCAA GCAGCAGTAC

Mouse GCATTTTGAA AACAGCCAAG GGCTTGGATT TTGAGGCCAA GCAGCAATAC

Opossum CAATCTTGAA AACAGCCAAG GGTTTGGATT TTGAGACCAA ACAGCAATAC

Orangutan GCATTTTGAA AACAGCAAAG GGCTTGGATT TTGAGGCCAA GCAGCAGTAC

Platypus GAATATTAAA AACTGCCAAG GGCTTGGACT TTGAAACCAA AAACAAGTAC

Rabbit GCATCCTGAA GACAGCCAAG GGCTTGGACT TCGAGGCGAA GCAGCAGTAC

Rat GCATTCTGAA AACAGCCAAG GGCTTGGATT TTGAGGCCAA GCAGCAGTAC

Human ATTCTACACG TAGCAGTGAC GAATGTGGTA CCTTTTGAGG TCTCTCTCAC

Chimpanzee ATTCTACACG TAGCAGTGAC GAATGTGGTA CCTTTTGAGG TCTCTCTCAC

Cow ATCCTGTACG TGGCAGTGAC AAATGTGGCC CCCTTTGAAG TCACTCTCCC

Dog GTCTTGTACG TGACTGTGGT GAACGTGACC CCGTTTGAGG TCATCCTCTC

Elephant ATTCTATATG TGACAGCTGA GAATGAGGTC CCCTTTGTGG TCAGTCTTTC

Gorilla ATTCTACACG TAGCAGTGAC GAATGTGGTG CCTTTTGAGG TCTCTCTCAC

Guinea_Pig ATTCTACATG TGACAGCAAC GAATGCAGTA CCCTTTGAAG TGATTCTTCA

Horse AATCTGTTTG TGACCGTGAA GAATGTGGCC CCCTTTGAGG TCACTCTCTC

Marmoset ATTCTACATG TGGCAGTAAC AAATGTGGCC CCATTTGAGG TCTCTCTCAC

Mouse ATCCTTCATG TGAGAGTGGA GAACGAGGAA CCCTTTGAGG GGTCTCTTGT

Opossum ATTCTTCACG TAACAGCAGT GAACGAAGTT CCTTTTGTTG TCACTCTCCC

Orangutan ATTCTACACG TAGCAGTGAC GAATGTAGTG CCTTTTGAGG TCTCTCTCAC

Platypus ACGCTATATG TAACTGTAGT GAACGAAGAT CCATTTGCAA TCGAACTCCA

Rabbit ATTCTCCACG TGACCGTGCA GAACGCCGTC CCCTTCGAGG TGGCCCTCCC

Rat ATTCTGCACG TGACAGTGGA AAATGAGGAG CCCTTTGAGG GGTCTCTTGT

Human CACCTCCACA GCCACCGTCA CCGTGGATGT GCTGGATGTG AATGAAGCCC

Chimpanzee CACCTCCACA GCCACCGTCA CCGTGGATGT GCTGGATGTG AATGAAGCCC

Cow CACTTCCACA GCCACCGTCA CTGTGGATGT GATAGATGTG AATGAAGCCC

Dog CACCTCCACA GCCACTGTCA CTGTGGACGT GGAAGATGTG AATGAAGCCC

Elephant CACCTCCACA GCCACAGTGA CTGTGGATGT GATAGATGTG AATGAAGCCC

Gorilla CACCTCCACA GCCACCGTCA CCGTGGATGT GCTGGATGTG AATGAAGCCC

Guinea_Pig GACCTCCACA GCCACTGTCA CTGTAGATGT GCTGGACATC AATGAAGCCC

Horse CACCTCCACA GCCACTGTCA CCGTGGACGT GATGGATGTG AACGAAGCCC

Marmoset CACCTCCACA GCTACTGTCA TCGTGGATGT GCTGGATGTG AATGAAGCCC

Mouse CCCTTCCACA GCCACTGTCA CTGTGGACGT GGTAGACGTG AATGAAGCCC

Opossum CACCTCTACA GCCACAGTCA CTGTGGATGT GCTGGATGTC AATGAAGCCC

Orangutan CACCTCCACA GCCACTGTCA CCGTGGATGT GCTGGATGTG AATGAAGCCC

Platypus TACCTCCACA GCAACTGTCA CTGTGGATGT AATGGATGAG AATGAAGCTC

Rabbit CACCTCCACG GCCACCGTGA CTGTGGACGT GGTGGACAGG AACGAAGCCC

Rat CCCTTCCACA GCCACTGTCA CCGTGGATGT GGTAGACGTG AATGAAGCCC

Human CCATCTTTGT GCCTCCTGAA AAGAGAGTGG AAGTGTCCGA GGACTTTGGC

Chimpanzee CCATCTTTGT GCCTCCTGAA AAGAGAGTGG AAGTGTCGGA GGACTTTGGC

Cow CCATCTTTGT GCCTCCTCAA AAGAGAGTGG AAGTGCCCGA GGACTTTGGC

Dog CCATCTTCAT CCCTTGCCCA AAGGTAGTGT CAATCCCTGA AGACTTTGGT

Elephant CCATCTTCAT GCCTCCTATC AAGAGAGTGG AAGTGCCCGA AGACTTTGGT

Gorilla CCATCTTTGT GCCTCCTGAA AAGAGAGTGG AAGTGTCCGA GGACTTTGGC

Guinea_Pig CCATCTTTGT ACCCCCTGAA AAAAGAGTAG ATGTACCAGA AGACCTTCAG

Horse CCATCTTTGT GCCTCCCCAA AAGAGAGTGG AAGTGCCTGA AGACTTCGGC

Marmoset CCATCTTTGT GCCTCTTGAA AAGAAAGTGA CAGTGTCCGA GGACTTTGGT

Mouse CCATCTTTAT GCCTGCGGAG AGGAGAGTCG AAGTGCCCGA AGACTTTGGT

Opossum CAGTCTTTAT ACCACCAGTT ATGAGAGTGG AGATACCAGA GGATTTTGGA

Orangutan CCATCTTTGT GCCTCCTGAA AAGAGAGTGG AAGTGTCCGA GGACTTTGGC

Platypus CTGTCTTTGT GCCACCAGTC AAGAAAGTGG AAGTGCCAGA AGACTTTGCT

Rabbit CCATCTTTGT GCCTCCCGAG AAGAGGGTGG AGGTGCCCGA GGACTTCGGC

Rat CCATTTTTGT GCCTGCGGAG AAGAGAGTCG AGGTGCCTGA GGACTTTGGT

Human GTGGGCCAGG AAATCACATC CTACACTGCC CAGGAGCCAG ACACATTTAT

Chimpanzee GTGGGCCAGG AAATCACATC CTACACTGCC CAGGAGCCAG ACACATTTAT

Cow GTGGGCCTGG AGATCACATC CTATACTGCC CGGGAGCCAG ACACATTTAT

Dog GTGGGCCAGG AAATCACATC CTACACCGCC GAGGATCCAG ATACATATAT

Elephant GTGGGCCAGG AGATCACGTC CTACACTGCC CAGGATCCAG ACAAGTTTAT

Gorilla GTGGGCCAGG AAATCACATC CTACACTGCC CAGGAGCCAG ACACATTTAT

Guinea_Pig GTGGGCTTGG AAATCACATC CTACACAGCT CGGGAGCCAG ACACATTTAT

Horse GTGGGCCTGG AAATCACATC CTACACTGCC CAGGATCCCG ACAAGTTTAT

Marmoset GTGGGCCAGG AAATCACATC CTACACTGCC CGGGAGCCAG ACACATTTAT

Mouse GTGGGTCAGG AAATCACATC TTATACCGCT CGAGAGCCGG ACACGTTCAT

Opossum GTGGGAGACC AAATTACATC CTATACTGCT CAAGATCCTG ACAAGTTTAT

Orangutan GTGGGCCAGG AAATCACATC CTACACTGCC TGGGAGCCAG ACACATTTAT

Platypus GTCGGGCAAG AGATTACTAC CTACGAGGCT CAGGATCCAG ACAAGTTTCT

Rabbit GTGGGGCTAG AAATCACATC CTACACCGCC CGGGACCCGG ACACATTCAT

Rat GTGGGTCTGG AGATCGCATC TTACACTGCG CGAGAGCCAG ACACATTCAT

Human GGAACAGAAA ATAACATATC GGATTTGGAG AGACACTGCC AACTGGCTGG

Chimpanzee GGAACAGAAA ATAACATATC GGATTTGGAG AGACACTGCC AACTGGCTGG

Cow GGAACAGAAG ATCACGTATC GGATTTGGAG GGACACTGCC AACTGGCTGG

Dog GGAACAGAGG ATAACGTATC GGATTTGGAG GGATGCTGCC GGTTGGCTGG

Elephant GCAACAGAAG ATAACGTATC GAATTTGGAG GGATACTGCC AACTGGCTGG

Gorilla GGAACAGAAA ATAACATATC GGATTTGGAG AGACACTGCC AACTGGCTGG

Guinea_Pig GGAGCAGAGG ATTACGTACC GGATTTTGAG GGACCCTGCC AACTGGTTGG

Horse GGAACAGAAG ATAACGTATC GGATTTGGAG GGATGCTGCC AACTGGCTGA

Marmoset GGAACAGAAA ATAACGTATC GGATTTGGAG AGACACTGCC AATTGGCTGG

Mouse GGATCAGAAG ATCACGTATC GGATTTGGAG GGACACTGCC AACTGGCTGG

Opossum GACACAGAAG ATAATGTATC GCATTTGGAG GGATCCAGCT AATTGGCTAG

Orangutan GGAACAGAAA ATAACGTATC GGATTTGGAG AGACACTGCC AACTGGCTGG

Platypus CCAACAGAAG ATAACGTATC GCATTTGGAG GGATGACGCT AAATGGCTGG

Rabbit GCAGCAGAAG ATAACGTATC GGATTTGGAG GGATGCCGCC AACTGGCTGG

Rat GGAACAGAAG ATCACGTATC GGATTTGGAG GGACACTGCC AATTGGCTGG

Human AGATTAATCC GGACACTGGT GCCATTTCCA CTCGGGCTGA GCTGGACAGG

Chimpanzee AGATTAATCC GGACACTGGT GCCATTTCCA CTCGGGCTGA GCTGGACAGG

Cow AGATTAATCC AGAAACGGGT GCCATTTCCA CTCGGGCTGA GTTGGACAGA

Dog AGGTTAATCC AGAATCTGGT GCCATTTTCA CTCGGGCTGA GCTGGACAGA

Elephant AGATTAATCC GGACACTGGG GCCATTTCCA CTCGGGCTGA GTTGGACAGA

Gorilla AGATTAATCC GGACACTGGT GCCATTTCCA CTCGGGCTGA GCTGGACAGG

Guinea_Pig AGATTAACCC AGAAAGCGGT GCCATTACCA GTCGTGCTGA GCTGGACAGA

Horse AGATTAATCC TGACACTGGT GCCATTTCCA CTCAGGCTGA GTTGGACAGA

Marmoset AGATTAATCC GGACACTGGT GCCATTTCCA CTCGGGCTGA GCTGGACAGG

Mouse AGATTAACCC AGAGACTGGT GCCATTTTCA CGCGCGCTGA GATGGACAGA

Opossum ATGTTAATCC TGACTCTGGT GTCATTTCTA CTCGGGACAG TCTGGATAGA

Orangutan AGATTAATCC GGACACTGGT GCCATTTCCA CTCGGGCTGA GCTGGATAGG

Platypus AAATTAACCC GGATTCTGGG CTTATTTCCC CTCGGGCTAG TATGGATAGA

Rabbit AGATTAACCC AGAGACGGGT GCCATCTCCA CGCGGGCGGA GCTGGACCGG

Rat AGATTAACCC AGAGACTGGG GTCATTTCCA CTCGGGCTGA GATGGACAGA

Human GAGGATTTTG AGCACGTGAA GAACAGCACG TACACAGCCC TAATCATAGC

Chimpanzee GAGGATTTGG AGCACGTGAA GAACAGCACG TACACAGCCC TAATCATAGC

Cow GAGGATGTCG ATCATGTGAA GAACAGCACG TACACGGCCC TCATTATAGC

Dog GAGGATTTTG AGCACGTGAA GAATAGCACG TATGAAGCCC TCATTATAGC

Elephant GAGGACTTGG AGCATGTGAA GAACAGCACA TACACGGCCC TCATTATTGC

Gorilla GAGGATTTTG AGCACGTGAA GAACAGCACG TACACAGCCC TAATCATAGC

Guinea_Pig GAGGCCTCTG ATTATGTGAA GAACAGCACG TACATAGCCC TCATCATCGC

Horse GAGAACTTGG AGCACGTGAA GAACAGCACG TACACGGCCC TCATTATAGC

Marmoset GAGAACTTAG AGCATGTGAA GAACAGCACA TACACAGCCC TCATCATAGC

Mouse GAAGACGCTG AGCATGTGAA GAACAGCACA TATGTAGCTC TCATCATCGC

Opossum GAGAATGTTG AGTTTGTGAA GAATAGTACC TACACAGCCC TCATTGTGGC

Orangutan GAGGATGTTG AGCACGTGAA GAACAGCACA TACACAGCCC TCATCATAGC

Platypus GAGAATATTG AATACGTGAA GAACAACACC TACACTGCCC TTATCGTGGC

Rabbit GAGGACACAG ACTATGTCAA GAACAGCACG TACACGGCGC TCATCATCGC

Rat GAAGATTCGG AGCATGTGAA GAACAGCACG TATACAGCTC TCATCATTGC

Human TACAGACAAT GGTTCTCCAG TTGCTACTGG AACAGGGACA CTTCTGCTGA

Chimpanzee TACAGACAAT GGTTCTCCAG TTGCTACTGG AACAGGGACA CTTCTGCTGA

Cow CACTGACAAT GGTTCTCCAC CTGCCACTGG GACAGGCACC CTGCTCTTGT

Dog CATTGACAAC GGTTCTCCAG TTGCTACTGG AACGGGAACT CTTCTACTGG

Elephant TACTGACGAT GGCTCTCTAC CTGCTACTGG TACTGGAACC TTTATTCTGA

Gorilla TACAGACAAT GGTTCTCCAG TTGCTACTGG AACAGGGACA CTTTTGCTGA

Guinea_Pig CACCGACAAT GGTTCTCCGA TCGCCACCGG AACAGGAACC CTCCTCTTGC

Horse CACGGACAAC GGTTCTCCAC TTGCTACTGG AACGGGAACC CTTCTTCTGA

Marmoset TACAGATAAT GGTTCTCCAG TTGCTACTGG AACAGGGACG CTTCTGCTGA

Mouse CACAGATGAT GGTTCACCCA TTGCCACTGG CACGGGCACT CTTCTCCTGG

Opossum TGTGGATGAT GGATCTCCTC CAGCTACAGG CACGGGAACC CTTCAGTTGT

Orangutan TACAGACAAC ---------- ---------- ---------- ----------

Platypus TGTTGATAAT GGTAACCCCG CAACCACTGG TACTGGAACC CTTCTCCTGA

Rabbit CACGGACAAC GGTTCCCCAC TTGCCACCGG CACTGGAACC CTTCTCCTGA

Rat CACAGATGAT GGTTCACCCA TTGCCACTGG CACAGGGACT CTTCTCCTGG

Human TCCTGTCTGA TGTGAATGAC AACGCCCCCA TACCAGAACC TCGAACTATA

Chimpanzee TCCTGTCTGA TGTGAATGAC AATGCCCCCA TACCAGAACC TCGAACTATA

Cow TCCTCGATGA TGTGAATGAC AATGGCCCCG TACCAGAACC CCGGACCATG

Dog TCCTCTCTGA TGTGAATGAC AATGGCCCCA TTCCAGAACC TCGAAATATG

Elephant TCCTCTCTGA TGTGAATGAC AATGCCCCCA TACCAGACCC TCGAAGTATG

Gorilla TCCTGTCTGA TGTGAATGAC AACGCCCCCA TACCAGAACC TCGAACTATA

Guinea_Pig ACCTCTCGGA CGTGAATGAC AATGGCCCCG TACCAGAACC CCGAACCATG

Horse TCCTCTCCGA TGTGAATGAC AATGGCCCTA TACCAGAACC TCGAAATATG

Marmoset TCCTGTCTGA TGTGAATGAC AATGCCCCCA TACCAGAACC CCGAAATTTG

Mouse TCCTGTTAGA CGTCAATGAC AACGCTCCCA TCCCAGAACC TCGAAACATG

Opossum TTCTCTTAGA TGTGAATGAT AATGGCCCAG TACCAGAGCC TCGAAATTTT

Orangutan ---------- ---------- ---------- ---------- ----------

Platypus CCCTCTCGGA CGTGAACGAT AATGGGCCGA TACCAGACCC TCGGAAGATG

Rabbit TCCTGTCTGA CGTGAATGAC AACGCGCCGG TGCCTGACCC GCGCAGGATG

Rat TCCTGTCAGA CGTCAACGAC AATGCTCCCA TCCCAGAACC TCGAAATATG

Human TTCTTCTGTG AGAGGAATCC AAAGCCTCAG GTCATAAACA TCATTGATGC

Chimpanzee TTCTTCTGTG AGAGGAATCC AAAGCCTCAG GTCATAAACA TCATTGATGC

Cow GACTTCTGCC AGAGGAATCC TGAGCCTCAC ATCATCAACA TCAATGATCC

Dog GACTTCTGCC AGAAAAACCC ACAGCCTCAT GTCATCAACA TCATTGATCC

Elephant GACTTCTGCC AGGTGGACCC ACAGCCTCAT ACCATATACA TCATTGATCC

Gorilla TTCTTCTGTG AGAGGAATCC AAAGCCTCAG GTCATAAACA TCATTGATGC

Guinea_Pig CACTTCTGCC AAAGGGACCC ACAGCCTCAT ACCATAACCA TCGTAGATCC

Horse GACTTCTGCC AGAAGGATCC GCAGCCTCAT ATCATCAACA TCATTGACCC

Marmoset TACTTCTGCG AGAGGAATCC AGAGCCTCAA GTCATCAACA TCATTGATGC

Mouse CAGTTCTGCC AGAGGAACCC ACAGCCTCAT ATCATCACCA TCTTGGATCC

Opossum GACATTTGCC AGAGGAATCC AGAGCCTCAG GTTATAAATA TTATTGATCC

Orangutan ---------- ---------- ---------- ---------- ----------

Platypus GACTTCTGTC AGCGGGATTA TCAGGCCCAC ACTATTCACA TCATCGATAA

Rabbit GACTTCTGCC AGAGGGACCC ACAACCTCAG CTCATAAACA TCATCGATCC

Rat CAGTTCTGCC AGAGAAACCC GAAGCCCCAT GTCATCACCA TCTTGGATCC

Human AGACCTTCCT CCCAATACAT CTCCCTTCAC AGCAGAACTA ACACACGGGG

Chimpanzee AGACCTTCCT CCCAATACAT CTCCCTTCAC AGCAGAACTA ACACACGGGG

Cow TGATCTCCCT CCGAACACCT CCCCCTTTAC AGCAGAACTG ACACATGGGG

Dog AGATCTTCCC CCCAACACAT CTCCCTTCAC AGCAGAACTA ACACACGGCG

Elephant AGACCTTCCC CCCAATACTT CTCCCTTCAC AGCGGAACTA ATGTATGGGG

Gorilla AGACCTTCCT CCCAATACAT CTCCCTTCAC AGCAGAACTA ACACACGGGG

Guinea_Pig AGATCTGCCC CCCAACACTT ACCCCTTCAT GGCCGAACTC ACACACGGCG

Horse AGATCTTCCC CCCAACACAT CCCCCTTCAC AGCAGAACTA ACACATGGGG

Marmoset AGACCTTCCT CCCAATACAT CTCCCTTCAC GGCAGAACTA ACACATGGGG

Mouse AGACCTTCCC CCCAACACGT CCCCCTTTAC TGCTGAGCTA ACCCATGGGG

Opossum AGACCTTCCA CCAAACACTT CTCCTTTCAT GGCAGAATTG ACACACGGAG

Orangutan ---------- ---------- ---------- ---------- ----------

Platypus GGATCTTCCT CCAAATACCT ATCCTTTCCA GGCAGAACTT ACACACGGAG

Rabbit CGACCTTCCT CCCAATACCT CCCCCTTCAC CGCAGAGCTC ACGCACGGGG

Rat AGACCTTCCC CCAAACACAT CCCCCTTCAC TGCAGAGCTC ACCCATGGGG

Human CGAGTGCCAA CTGGACCATT CAGTACAACG ACCCAACCCA AGAATCTATC

Chimpanzee CGAGTGCCAA CTGGACCATT CAGTACAACG ACCCAACCCA AGAATCTATC

Cow CGAGTGTCAA TTGGACCATT GAGTACAATG ACCAAGAACG TGAGTCTCTG

Dog CAAGTGTCAA CTGGACCATC GAGTACAATG ACCCAGCTCG TGAATCTCTA

Elephant CAAGTGTCAA CTGGTCCATC GAGTACAAAG ATGCAGCCCG AGAGTCTCTT

Gorilla CGAGTGCCAA CTGGACCATT CAGTACAACG ACCCAACCCA AGAATCTATC

Guinea_Pig CAAGTGTCAA TTGGACCATT GAGTACAAAG ACTCAGCCCA AGACACTCTC

Horse CAAGTGTCAA CTGGACCATC GAGTACAATG ACCCAGCCCA CGAATCTCTT

Marmoset CGAGTGCCAA CTGGACCATT GAGTACAATG ACCCAACCCA AGAATCTCTC

Mouse CCAGCGTCAA CTGGACCATT GAGTATAATG ACGCAGCTCA AGAATCTCTC

Opossum CTAGTGTTAA CTGGACTATT GAATTTAATG ACAATGCAAG AGAATCTTTG

Orangutan ---------- ---------- ---------- -----GCCCA AGAATCTATC

Platypus CTGGTGTTAA CTGGACTTTG AAAATGAATA CGGAAACAAG AGATGAACTG

Rabbit CCAGCGTCAA CTGGACCATT GAGTACAGTG ACCCACGTCA AGAGTCTATC

Rat CCAGCGTCAA CTGGACCATT GAGTACAATG ACGCAGAACA AGAATCTCTC

Human ATTTTGAAGC CAAAGATGGC CTTAGAGGTG GGTGACTACA AAATCAATCT

Chimpanzee ATTTTGAAGC CAAAGATGGC CTTAGAGGTG GGTGACTACA AAATCAATCT

Cow ATTTTGAAGC CAAAGAAAAC CTTAGAGCTG GGTGACCACA AAATCAATCT

Dog ATTTTGAAGC CAAAGAAAAC TTTAGAGTTG GGTGACTACA AAATAAATCT

Elephant CTTTTGATGC CAAAGGTTCG TTTAGAGCTG GGTGACTACA AAGTACATCT

Gorilla ATTTTGAAGC CAAAGATGGC CTTAGAGGTG GGTGACTACA AAATCAATCT

Guinea_Pig ATCTTGAAGC CAAAGAAAAC CATGGAAGTG GGCGACTACA AAATCAACCT

Horse GTTTTGAAGC CAAAGAAAAT CCTAGAGTTG GGTGACTACA AAATAAATCT

Marmoset ATTTTGAAGC CAAAGATATA CTTAGAGGTG GGTGAGTACA AAATCAATCT

Mouse ATTTTGCAAC CAAGAAAGGA CTTAGAGATT GGCGAATACA AAATCCATCT

Opossum ATTTTAAGGC CAAAGAAAGA ATTAGAAATT GGTGAGTACA AGATCAATCT

Orangutan ATTTTGAAGC CGAAGATGGC CTTAGAGGTG GGTGACTACA AAATCAATCT

Platypus ACCCTAATAC CAAAAAAGTC GCTCGAAGTG GGAGAGTACA AGATCAATCT

Rabbit ATTTTGAAGC CAAAGAAGAC TTTAGAGCTG GGCGACTACA AAATTAACCT

Rat ATTTTGCAAC CAAGAAAGGA CTTAGAGATT GGCGAATACA AAATCAATCT

Human CAAGCTCATG GATAACCAGA ATAAAGACCA AGTGACCACC TTAGAGGTCA

Chimpanzee CAAGCTCATG GATAACCAGA ATAAAGACCA AGTGACCACC TTAGAGGTCA

Cow CAAGCTCATA GACAACCAGA ACAAAGACCA GGTGACCACA CTTGATGTGC

Dog CAAGCTCACA GATAACCAGA ACAAGGACCA GGTGACCACC CTAGATGTGT

Elephant CAAGCTCACG GATAACCAGA ATAAAGACCA GGTGACCACC TTAGATGTCT

Gorilla CAAGCTCATG GATAACCAGA ATAAAGACCA AGTGACCACC TTAGAGGTCA

Guinea_Pig CAAGCTCATT GATAACCAGA ACAAAGACCA AGTGACCACC TTGGATATCA

Horse CAAGCTCATG GATAACCAGA ACAAAGACCA AGTGACCACC TTAGATGTGG

Marmoset CAAGCTCATG GATAACCAGA ATAAAGACCA AGTGACCACC TTAGAGGTTC

Mouse CAAGCTCGCG GATAACCAGA ACAAAGACCA GGTGACCACG TTGGACGTCC

Opossum CAAGCTGACA GACAACCAGA ATAAAGACCA AGTAACCACC TTGGAGGTGT

Orangutan CAAGCTCATG GATAACCAGA ATAAAGACCA AGTGACCACC TTAGAGGTCA

Platypus CAAGCTGACA GACAACCAGA ACAAGGACCA AGTGACCACT TTGGAGGTCT

Rabbit GAAGCTCGCA GACAACCAGA ACAAAGACCA GGTGACCACC CTGGATGTGC

Rat CAAGCTCTCG GATAACCAGA ATAAAGACCA GGTGACCACG TTGGAGGTCC

Human GCGTGTGTGA CTGTGAAGGG GCCGCTGGCG TCTGTAGGAA GGCACAGCCT

Chimpanzee GCGTGTGTGA CTGTGAAGGG GCCGCCGGCG TCTGTAGGAA GGCACAGCCT

Cow ACGTGTGTGA CTGTGATGGG ATCGTCAGCA ACTGCAGGAA GGCCACGGCC

Dog TTGTGTGCGA CTGCGAAGGT GTCGTCAACA GCTGCAAGAG GACGGCGCCT

Elephant TTATGTGTGA CTGCGAAGGG GCCGTCAACA CCTGTAAGAG ACATATGCCA

Gorilla GCGTGTGTGA CTGTGAAGGG GCCGCCGGCG TCTGTAGGAA GGCACAGCCT

Guinea_Pig GCGTGTGTGA CTGTGAAGGG ACCGTCACCA ACTGTATGAG GGCAAATTAT

Horse CTGTGTGTGA CTGTGAAGGG GCCGTCACCA ACTGTGGGAG GAACAGGCAG

Marmoset ACGTATGTGA CTGTGAAGGG ACCGCCAACG TCTGTCGAAA GGCACAGTAT

Mouse ATGTGTGTGA CTGTGAAGGG ACGGTCAACA ACTGCATGAA GGCGGGAATC

Opossum ACGTGTGTGA CTGTGAAGGG GCCGTATCGA AGTGCACGAA GGCAGGTGTA

Orangutan GCGTGTGTGA CTGTGAAGGG GTCGCCGGCG TCTGTAAGAA GGCACAGCCT

Platypus ATGTGTGTGA CTGTGAAGGG ACAGTCACAA ACTGTAAAAA GATTGGGCCA

Rabbit TCGTGTGTGA CTGCGAGGGC GCCGTCAACA ACTGCATGCG GGCCACGTAC

Rat ACGTGTGTGA CTGTGAAGGG ACCGTCAACA ACTGCATGAA GGCGATCTCC

Human ---GTCGAAG CAGGATTGCA AATTCCTGCC ATTCTGGGGA TTCTTGGAGG

Chimpanzee ---GTCGAAG CAGGATTGCA AATTCCTGCC ATTCTGGGGA TTCTTGGAGG

Cow TTTGCTGAAG CAGGATTGCA AGTTCCCGCC ATCCTGGGGA TCCTCGGAGG

Dog TACGCCGAAG CAGGCTTGCA GGTTCCTGCC ATCTTGGGCA TTCTCGGAGG

Elephant ATTGCTAACG CAGGATTGCC AGTTTCTGCT GTTTTGGGGA TTCTCGGAGG

Gorilla ---GTCGAAG CAGGATTGCA AATTCCTGCC ATTCTGGGGA TTCTTGGAGG

Guinea_Pig ---GTAGAAG CGGGACTGCA GGTGCCTGCC ATCCTGGGCA TTCTTGGAGG

Horse GTTGCTGAAG CAGGATTGCA GGTTTCTGCC ATTCTGGGGA TTCTTGGAGG

Marmoset ---GTCGAAG CAGGATTGCA AGTTCCTGCC ATCCTGGGGA TTCTTGGAGG

Mouse ---GTGGCAG CAGGATTGCA AGTTCCTGCC ATCCTCGGAA TCCTTGGAGG

Opossum ATTGCTCAGA CCGGCATGGA TGTACCAGCT ATCCTGGGAA TACTGGGAGG

Orangutan ---ATCGAAG CAGGATTGCA AATTCCTGCC ATTCTGGGGA TTCTTGGAGG

Platypus ATCGCTGCTA GCAGTATGGG GGTACCAGCC ATCCTGGGAA TTCTCGGAGG

Rabbit ---GTGGAAG CGGGCCTGCA GGTCCCCGCC ATTCTGGGCA TTCTCGGAGG

Rat ---CTGGAAG CAGGATTACA AGTTCCCGCC ATCCTTGGAA TCCTGGGAGG

Human AATTCTTGCT TTGCTAATTC TGATTCTGCT GCTCTTGCTG TTTCTTCGGA

Chimpanzee AATTCTTGCT TTGCTAATTC TGATTCTGCT GCTCTTGCTG TTTCTTCGGA

Cow CATCCTTGCT TTTCTGATCC TTATTTTGCT GCTTCTGCTA CTTGTTCGGA

Dog AATCCTCGCT CTACTAATCC TGATTCTGCT GCTTCTGCTA TTTGTTCGGA

Elephant AATCCTTGCT TTGCTAATCC TGATTCTGCT GCTCCTGCTA TTTCTTCGGA

Gorilla AATTCTTGCT TTGCTAATTC TGATTCTGCT GCTCTTGCTG TTTCTTCGGA

Guinea_Pig AATCCTTGCT TTGCTAATTC TGATTCTACT GCTTCTACTG TTTATTCGGA

Horse CATCCTTGCT TTGCTACTCC TGATTCTGCT ACTTCTGCTA TTTCTTCGGA

Marmoset AATTCTTGCT TTGCTAATCC TGATTCTACT GCTCTTGCTG TTTCTTCGGA

Mouse GATCCTCGCC CTGCTGATTC TGATCCTGCT GCTCCTACTG TTTCTACGGA

Opossum AATCCTTGCA CTGCTAATTT TATTGCTGCT TTTGCTCCTC TTTATTCGAA

Orangutan AATTCTTGCT TTGCTAATTC TGATTCTGCT GCTCCTGCTG TTTCTTCGGA

Platypus AATCCTTGCC TTGCTTCTCC TGTTGCTGCT GCTTCTCCTG TTTGTGCGAA

Rabbit AATCCTGGCT CTGCTCATCC TGGTTCTGCT GCTGCTGCTG TTTGTCCGGA

Rat GATCCTGGCC CTCCTGATTC TGATCCTCCT GCTCCTACTG TTTCTACGGA

Human GGAGAGCGGT GGTCAAAGAG CCCTTACTGC CCCCAGAGGA TGACACCCGG

Chimpanzee GGAGAGCGGT GGTCAAAGAG CCCTTACTGC CCCCAGAGGA TGACACCCGG

Cow GGAGAAGGGT GGTCAAAGAG CCCTTACTGC CCCCAGAAGA TGACACCCGG

Dog GGAGAAGGGT GGTCAAAGAG CCCTTACTTC CCCCAGAAGA TGACACCCGG

Elephant GGAGAAGAGT GGTCAAAGAG CCCTTACTGG CCCCTGAGGA TGACACTCGG

Gorilla GGAGAGCGGT GGTCAAAGAG CCCTTACTGC CCCCAGAGGA TGACACCCGG

Guinea_Pig GGAAAGGGGC GGTCAAAGAG CCCTTACTGC CCCCAGAAGA TGACACGCGA

Horse GAAGAGGGGT GGTCAAAGAG CCCTTACTGC CCCCAGAAGA TGACACCCGG

Marmoset GGAGAACCGT GGTCAAAGAG CCCTTACTGC CCCCGGAGGA TGACACCCGG

Mouse GGAGAACGGT GGTCAAAGAG CCCCTGCTGC CACCAGATGA TGATACCCGG

Opossum GAAGGCCAGC AGTTAAAGAG CCATTACTGC CCCCCGAGGA TGACACCCGG

Orangutan GGAGAGCGGT GGTCAAAGAG CCCTTACTGC CCCCAGAGGA TGACACCCGG

Platypus GAAGACATGT GGTGAAAGAG CCTTTGCTGC CCCCAGAAGA TGACACCCGG

Rabbit GGAGAGGGGT GGTCAAAGAG CCCTTACTGC CCCCAGAGGA CGACACCCGG

Rat GGAGAACGGT GGTCAAAGAG CCCTTGCTGC CACCAGATGA CGATACCCGG

Human GACAACGTTT ATTACTATGA TGAAGAAGGA GGCGGAGAAG AGGACCAGGA

Chimpanzee GACAACGTTT ATTACTATGA TGAAGAAGGA GGCGGAGAAG AGGACCAGGA

Cow GACAATGTGT ATTACTATGA TGAAGAAGGA GGTGGAGAAG AAGATCAGGA

Dog GACAATGTTT ATTACTATGA TGAAGAAGGA GGTGGAGAGG AGGATCAGGA

Elephant GACAACGTTT ATTACTATGA TGAAGAAGGA GGTGGAGAAG AGGACCAGGA

Gorilla GACAACGTTT ATTACTATGA TGAAGAAGGA GGCGGAGAAG AGGACCAGGA

Guinea_Pig GACAATGTTT ATTACTATGA TGAAGAAGGC GGTGGAGAAG AGGACCAGGA

Horse GACAATGTTT ATTACTATGA TGAAGAAGGA GGTGGAGAAG AAGACCAGGA

Marmoset GACAATGTTT ATTACTATGA TGAAGAAGGA GGCGGAGAAG AGGATCAGGA

Mouse GACAATGTGT ATTACTATGA TGAAGAAGGA GGTGGAGAAG AAGACCAGGA

Opossum GACAACGTGT ATTATTATGA TGAAGAAGGA GGGGGAGAAG AAGATCAGGA

Orangutan GACAACGTTT ATTACTATGA CGAAGAAGGA GGCGGAGAAG AGGACCAGGA

Platypus GATAATGTTT ATTACTATGA TGAAGAGGGA GGTGGCGAAG AAGACCAGGA

Rabbit GACAACGTGT ATTACTACGA CGAGGAAGGA GGCGGAGAGG AGGACCAGGA

Rat GACAATGTGT ATTACTATGA TGAAGAGGGA GGTGGAGAAG AAGACCAGGA

Human CTTTGACTTG AGCCAGCTGC ACAGGGGCCT GGACGCTCGG CCTGAAGTGA

Chimpanzee CTTTGACTTG AGCCAGTTGC ACAGGGGCCT GGACGCTCGG CCTGAAGTGA

Cow CTTTGACTTG AGCCAGTTAC ATAGGGGCCT GGATGCTCGG CCTGAAGTGA

Dog CTTTGACTTG AGCCAGTTGC ACAGGGGCCT GGATGCTCGG CCTGAAGTGA

Elephant CTTTGACTTG AGCCAGTTGC ACAGGGGCCT GGATGCTCGA CCTGAAGTGA

Gorilla CTTTGACTTG AGCCAGTTGC ACAGGGGGCT GGACGCTCGG CCTGAAGTGA

Guinea_Pig TTTTGACTTG AGCCAGTTGC ACAGGGGCCT GGATGCTCGG CCTGAAGTGA

Horse CTTTGACTTG AGCCAGTTAC ACAGGGGCCT GGATGCTCGG CCCGAAGTGA

Marmoset CTTTGACTTG AGCCAGTTGC ACAGGGGCCT AGACGCTCGG CCTGAAGTAA

Mouse CTTTGATTTG AGCCAGCTGC ACAGGGGCCT GGATGCCCGA CCGGAAGTGA

Opossum CTTTGACCTG AGCCAGTTGC ACAGGGGCCT GGATGCTCGA CCAGAAGTGA

Orangutan CTTTGACTTG AGCCAG---C ACAGGGGC-- -GACGCTCGG CCTGAA---A

Platypus CTTTGACTTG AGCCAGTTGC ATAGGGGCCT GGATGCTCGA CCTGAAGTTA

Rabbit CTTTGACTTG AGCCAGCTGC ACAGAGGCCT GGATGCTCGG CCCGAAGTCA

Rat CTTTGATTTG AGCCAGCTGC ACAGGGGCCT TGATGCCAGA CCGGAAGTGA

Human CTCGTAACGA CGTTGCACCA ACCCTCATGA GTGTCCCCCG GTATCTTCCC

Chimpanzee CTCGTAACGA CGTTGCACCA ACCCTCATGA GTGTCCCCCG GTACCTTCCC

Cow CTCGCAATGA CGTGGCACCA ACCCTCATGA GTGTGCCCCA GTACCGACCC

Dog CTCGCAATGA TGTGGCCCCA ACCCTCCTGA GTGTGCCCCA GTATCGGCCC

Elephant TTCGCAATGA TGTGGCACCT ACCCTCATGA GTGTGCCCCA GTATCGTCCC

Gorilla CTCGTAACGA CGTTGCGCCA ACCCTCATGA GTGTCCCCCG ATACCTTCCC

Guinea_Pig TTCGCAATGA CGTAGCTCCG ACCCTCATGA GTGTGCCCCA GTATCGTCCC

Horse TTCGCAATGA TGTGGCGCCA ACCCTCTTGA GTGTACCTCA ATATCGGCCC

Marmoset CTCGCAACGA TGTCGCACCA ACCCTCATGA GCGTTCCCCG GTACCTTCCC

Mouse CTCGAAATGA TGTGGCTCCC ACCCTCATGA GCGTGCCCCA GTATCGTCCC

Opossum CCCGTAATGA TGTTGCACCA ACTCTTATGA GTGTCCCCCA GTATCGCCCT

Orangutan CTCGT----- ---------- ---------- ---------- ----------

Platypus CCCGTAACGA TGTAGCCCCA ACTTCCATGA GTGTTCCCCA GTACCGCCCT

Rabbit TTCGCAATGA CGTGGCCCCG ACCCTCATGA GTGTGCCCCA GTACCGTCCC

Rat TTCGAAATGA TGTGGCTCCC ACCCTCATGA GCATGCCCCA GTATCGTCCC

Human CGCCCTGCCA ATCCCGATGA AATTGGAAAT TTTATTGATG AAAATCTGAA

Chimpanzee CGCCCTGCCA ATCCCGATGA AATTGGAAAT TTTATTGATG AAAATCTGAA

Cow CGCCCTGCCA ATCCTGATGA AATTGGAAAC TTTATTGATG AAAACCTGAA

Dog CGCCCTGCCA ATCCTGATGA AATTGGAAAC TTTATTGATG AAAACCTGAA

Elephant CGCCCCGCCA ATCCTGATGA AATTGGAAAT TTTATTGATG AAAACCTGAA

Gorilla CGCCCTGCCA ATCCCGATGA AATTGGAAAT TTTATTGATG AAAATCTGAA

Guinea_Pig CGCCCTGCCA ATCCCGATGA AATTGGGAAT TTTATTGACG AAAACCTGAA

Horse CGCCCAGCCA ATCCTGATGA AATTGGAAAC TTCATTGATG AGAACCTGAA

Marmoset CGCCCTGCCA ATCCCGATGA AATTGGAAAT TTTATTGATG AAAATCTGAA

Mouse CGTCCTGCCA ATCCTGATGA AATTGGAAAC TTCATCGATG AAAACCTGAA

Opossum CGCCCTGCGA ATCCGGATGA AATTGGCAAT TTTATTGATG AGAACCTCAA

Orangutan ---------- ---------- ---------- ---------- --AACCTGAA

Platypus CGCCCAGCCA ACCCAGATGA AATTGGCAAT TTTATTGATG AGAACCTTAA

Rabbit CGACCTGCCA ATCCTGACGA AATCGGGAAC TTCATTGATG AGAACCTGAA

Rat CGTCCAGCCA ATCCTGATGA AATCGGGAAC TTCATCGATG AAAACCTGAA

Human AGCGGCTGAT ACTGACCCCA CAGCCCCGCC TTATGATTCT CTGCTCGTGT

Chimpanzee AGCGGCTGAT ACTGACCCCA CAGCCCCGCC TTATGATTCT CTTCTCGTGT

Cow GGCAGCTGAT AGTGACCCCA CTGCCCCACC CTATGACTCT CTGCTGGTGT

Dog GGCAGCGGAC ACTGACCCTA CTGCTCCTCC TTATGACTCT CTGCTCGTGT

Elephant GGCGGCTGAT AGCGACCCCA CTGCTCCCCC TTATGACTCT CTGCTTGTGT

Gorilla AGCGGCTGAT ACTGACCCCA CAGCCCCGCC TTATGATTCT CTTCTCGTGT

Guinea_Pig GGCTGCGGAC AGTGACCCCA CAGCGCCCCC TTACGACTCT CTGCTTGTGT

Horse GGCAGCCGAT AGTGACCCCA CTGCCCCCCC TTACGACTCT CTGCTCGTGT

Marmoset GGCGGCTGAT AGTGACCCCA CGGCCCCACC TTATGATTCT CTGCTCGTGT

Mouse GGCAGCCGAC AGCGACCCCA CGGCACCCCC TTACGACTCT CTGTTGGTGT

Opossum GGCTGCTGAC AGTGACCCCA CAGCACCCCC GTACGACTCT CTGCTGGTGT

Orangutan GGCGGCTGAT ACTGACCCCA CAGCCCCGCC TTATGATTCT CTGCTCGTGT

Platypus GGCTGCAGAC AGTGACCCCA CTGCCCCACC CTATGATTCT TTACTGGTGT

Rabbit GGCGGCCGAC AGCGACCCCA CAGCGCCCCC GTACGACTCC CTGCTGGTGT

Rat GGCAGCGGAC AGTGACCCCA CAGCGCCCCC TTACGACTCT CTGTTGGTGT

Human TTGACTATGA AGGAAGCGGT TCCGAAGCTG CTAGTCTGAG CTCCCTGAAC

Chimpanzee TTGACTATGA AGGAAGCGGT TCCGAAGCTG CTAGTCTGAG CTCCCTGAAC

Cow TTGATTATGA AGGAAGTGGT TCCGAAGCTG CTACTCTGAG CTCCCTGAAC

Dog TTGACTATGA AGGAAGCGGT TCTGAAGCTG CTAGTCTGAG CTCCTTGAAC

Elephant TTGACTATGA AGGAAGCGGT TCTGAAGCTG CTAGTCTGAG CTCTCTGAAC

Gorilla TTGACTATGA AGGAAGCGGT TCCGAAGCTG CTAGTCTGAG CTCCCTGAAC

Guinea_Pig TCGACTACGA AGGCAGTGGT TCCGAGGCTG CGGACCTGAG CTCCCTGAAC

Horse TTGACTATGA AGGAAGCGGT TCTGAAGCTG CTACTCTGAG CTCCCTGAAC

Marmoset TTGACTATGA AGGAAGTGGT TCCGAAGCTG CTAGTCTGAG CTCCCTGAAC

Mouse TCGATTACGA GGGCAGTGGT TCTGAAGCCG CTAGCCTGAG CTCACTGAAC

Opossum TTGATTATGA AGGAAGTGGC TCCGAAGCGG CCTCCCTGAG CTCACTAAAC

Orangutan TTGACTATGA AGGAAGCGGT TCCGAAGCTG CTAGTCTGAG CTCCCTGAAC

Platypus TTGATTATGA AGGAAGCGGC TCTGAAGCAG CATCCCTCAG TTCTCTGAAC

Rabbit TCGACTACGA GGGCAGCGGC TCCGAGGCTG CCAGCCTCAG CTCCCTGAAC

Rat TTGACTATGA GGGGAGTGGT TCTGAAGCTG CCTCCCTGAG CTCGCTGAAC

Human TCCTCAGAGT CAGACAAAGA CCAGGACTAT GACTACTTGA ACGAATGGGG

Chimpanzee TCCTCAGAGT CAGACAAAGA CCAGGACTAT GACTACTTGA ACGAATGGGG

Cow TCCTCAGAGT CAGACCAAGA CCAGGACTAT GACTACCTGA ATGAATGGGG

Dog TCCTCAGAGT CAGACCAAGA CCAGGACTAT GACTACCTGA ATGAATGGGG

Elephant TCCTCAGAGT CAGACCAAGA CCAGGACTAT GACTACTTGA ACGAATGGGG

Gorilla TCCTCAGAGT CAGACAAAGA CCAGGACTAT GACTACTTGA ACGAATGGGG

Guinea_Pig TCCTCGGAGT CGGACCGGGA CCAGGACTAT GACTACTTGA ACGAGTGGGG

Horse TCCTCGGAGT CGGACCAAGA CCAGAACTAC GACTACCTGA ACGAATGGGG

Marmoset TCCTCGGAGT CAGACAAAGA CCAGGACTAT GACTACTTGA ACGAATGGGG

Mouse TCCTCTGAGT CGGATCAGGA CCAGGACTAC GATTATCTGA ACGAGTGGGG

Opossum TCTTCTGGCT CAGATGAAGA CCAGGATTAC GATTATCTGA ATGAATGGGG

Orangutan TCCTCAGAGT CAGACAAAGA CCAGGACTAT GACTACTTGA ACGAATGGGG

Platypus TCTTCTGGCT CAGATCAGGA CCAGGACTAC GACTACTTGA ACGAATGGGG

Rabbit TCCTCAGAGT CCGACCAAGA CCAGGACTAC GACTACCTGA ACGAGTGGGG

Rat TCCTCTGAGT CAGATCAGGA CCAGGACTAC GATTATCTGA ACGAATGGGG

Human CAATCGCTTC AAGAAGCTGG CTGACATGTA CGGAGGCGGC GAGGACGAC

Chimpanzee CAATCGCTTC AAGAAGCTGG CGGACATGTA CGGAGGTGGC GAGGACGAC

Cow CAATCGCTTC AAGAAGCTGG CGGACATGTA TGGAGGCGGC GAGGATGAC

Dog CAATCGCTTC AAGAAGCTGG CGGACATGTA TGGAGGTGGC GAGGACGAC

Elephant CAATCGCTTC AAGAAGCTGG CAGATATGTA TGGAGGCGGT GAGGACGAC

Gorilla CAATCGCTTC AAGAAGCTGG CCGACATGTA CGGAGGCGGC GAGGACGAC

Guinea_Pig CAATCGCTTC AAGAAGCTGG CGGACATGTA CGGAGGGGGC GAGGACGAC

Horse CAATCGCTTC AAGAAGCTGG CGGACATGTA CGGAGGTGGC GAGGACGAC

Marmoset CAATCGCTTC AAGAAGCTGG CAGACATGTA CGGAGGCGGC GAGGATGAC

Mouse CAACCGATTC AAGAAGCTGG CGGACATGTA CGGCGGTGGT GAGGACGAC

Opossum CAATCGTTTT AAAAAGCTGG CTGACATGTA TGGAGGAGGG GAAGATGAC

Orangutan CAATCGCTTC AAGAAGCTGG CAGACATGTA CGGAGGCGGC GAGGACGAC

Platypus CAATCGCTTC AAGAAGTTGG CTGATATGTA TGGTGGTGGG GAGGATGAC

Rabbit CAACCGCTTC AAGAAGCTGG CGGACATGTA CGGGGGCGGC GAGGACGAC

Rat CAACCGGTTC AAGAAGCTGG CCGATATGTA TGGTGGCGGC GAAGAAGAC

;

END;

BEGIN DATA;

DIMENSIONS NTAX=20 NCHAR=2799;

FORMAT DATATYPE=DNA INTERLEAVE=yes GAP=-;

[Gene Name: PMS1]

[Name: Human Len: 2799 Check: 0]

[Name: Chimpanzee Len: 2799 Check: 0]

[Name: Gorilla Len: 2799 Check: 0]

[Name: Marmoset Len: 2799 Check: 0]

[Name: Orangutan Len: 2799 Check: 0]

[Name: Mouse Len: 2799 Check: 0]

[Name: Guinea_Pig Len: 2799 Check: 0]

[Name: Rabbit Len: 2799 Check: 0]

[Name: Cow Len: 2799 Check: 0]

[Name: Dog Len: 2799 Check: 0]

[Name: Horse Len: 2799 Check: 0]

[Name: Pig Len: 2799 Check: 0]

[Name: Elephant Len: 2799 Check: 0]

[Name: Opossum Len: 2799 Check: 0]

[Name: Platypus Len: 2799 Check: 0]

[Name: Chicken Len: 2799 Check: 0]

[Name: Zebra_Finch Len: 2799 Check: 0]

[Name: Zebrafish Len: 2799 Check: 0]

[Name: Fugu Len: 2799 Check: 0]

[Name: Frog Len: 2799 Check: 0]

MATRIX

Human ATGAAACAAT TGCCTGCGGC AACAGTTCGA CTCCTTTCAA GTTCTCAGAT

Chimpanzee ATGAAACAAT TGCCTGCGGC AACAGTTCGA CTCCTTTCAA GTTCTCAGAT

Gorilla ---------- ---------- ---------- ---------- ----------

Marmoset ATGAAACAGT TGCCTGCGGC AACAGTTCGA CTCCTTTCAA GTTCTCAGAT

Orangutan ATGAAACAAT TGCCTGCGGC AACAGTTCGA CTCCTTTCAA GTTCTCAGAT

Mouse ATGAAACAGT TGCCTGCAGC AACAGTTCGC CTCCTGTCCA GTTCTCAGAC

Guinea_Pig ATGAAACAGT TGCCTGCAGC AACAGTTCGT CTTCTTTCAA GTTCCCAGAT

Rabbit ATGAAACAGT TGCCTGCAGC AACAGTTCGC CTCCTTTCAA GTTCTCAGAT

Cow ATGAAACAGT TGTCCGCAGC AACAGTTCGC CTACTTTCAA GCTCTCAAGT

Dog ATGAAACAGT TGCCTGCAGC AACAGTGCGC CTCCTTTCAA GCTCTCAGAT

Horse ATGAAACAGT TGCCTGCAGC AACAGTTCGC CTCCTTTCAA GTTCTCAGAT

Pig ATGAAACAGT TGCCTGCAGC AACAGTTCGC CTCCTTTCAA GTTCTCAAAT

Elephant ATGAAACAGT TGCCTGCAGC TACAGTGCGC CTCCTTTCAA GTTCTCAGAT

Opossum ATGAAGCAGT TGCCTGCAGC AACTGTTCGT CTCCTTTCAA GTTCTCAAGT

Platypus ATGAAGCAGC TGCCGGCGGC CACCGTGCGC CTGCTGTCCA GTTCTCAGGT

Chicken ATGAAGCAGC TGTCAGCAGA AACCGTTCGT CTCCTTTCCA GTTCTCAAGT

Zebra_Finch ATGAAACAGC TGCCTGGAGA GACAATTCGG CTCCTTTCAA GTTCTCAGGT

Zebrafish ATGAAGGCGC TTCCCCCCGA GACTGTGCGT CTGCTGTGCA GCTCTCAGGT

Fugu ATGAAGCAGC TGCCTCCAGA CACGGTGCGG CTTCTGTCCA GCTCCCAGGT

Frog ATGCATTGCT TGCCATCAGC TACAATCCGC TTGCTTTCCA GCTCCCAAGT

Human CATCACTTCG GTGGTCAGTG TTGTAAAAGA GCTTATTGAA AACTCCTTGG

Chimpanzee CATCACTTCG GTGGTCAGTG TTGTAAAAGA GCTTATTGAA AACTCCTTGG

Gorilla ---------- ---------- ---------- ---------- ----------

Marmoset TATCACTTCA GTGGTCAGTG TTGTAAAAGA GCTCATTGAA AACTCCTTGG

Orangutan CATCACTTCA GTGGTCAGTG TTGTAAAAGA GCTTATTGAA AACTCCTTGG

Mouse AATCACGTCA GTGGTCAGCG TTGTGAAAGA GCTCATTGAA AACTCCTTGG

Guinea_Pig AATCACTTCT GTGGTCAGTG TTGTAAAAGA GCTGGTCGAA AACTCCCTGG

Rabbit AATCACTTCA GTGGTCAGTG TTGTAAAAGA GCTCATTGAA AACTCACTGG

Cow AATCACTTCC GTGGTCAGTG TTGTAAAAGA GCTCATTGAA AACTCCTTGG

Dog AATCACTTCA GTGGTCAGTG TGGTAAAAGA GCTCCTTGAA AACTCTTTGG

Horse AATCACTTCA GTGGTCAGTG TTGTAAAAGA GCTCATTGAA AACTCCTTGG

Pig AATCACTTCA GTGGTCAGTG TTGTAAAAGA GCTCATTGAA AACTCCTTGG

Elephant AATCACTTCA GTGGTCAGTG TTGTAAAAGA GCTCATTGAA AACTCCTTGG

Opossum GATCACTTCT GTGATCAGTG TCATAAAAGA GCTAATAGAA AATTCATTGG

Platypus GATCACGTCC GTGGCCAGCG TGGTGAAGGA GCTGGTCGAG AATTCTCTGG

Chicken GATTACATCA GTTGTCAGCG TGGTGAAGGA GCTGATAGAA AACTCCTTGG

Zebra_Finch GATTACTTCA GTTGTCAGTG TGGTAAAGGA GCTGATAGAA AATTCCTTGG

Zebrafish TATTACATCT GTCCTGAATG TTGTCAAGGA ACTGATCGAG AATTCACTTG

Fugu CATCACGTCT GTGGTTAATG TTGTCAAAGA GCTGTTGGAA AATTCCCTGG

Frog GATTACCTCA GTGGTTAGTG TGGTAAAGGA ACTAGTAGAA AATGCTTTAG

Human ATGCTGGTGC CACAAGCGTA GATGTTAAAC TGGAGAACTA TGGATTTGAT

Chimpanzee ATGCTGGTGC CACAAGCGTA GATGTTAAAC TGGAGAACTA TGGATTTGAT

Gorilla ---------- ---------- ---------- ---------- ----------

Marmoset ATGCTGGCGC CACAAGCATA GATGTTAAAC TGGAGAACTA TGGATTTGAT

Orangutan ATGCTGGTGC CACAAGCATA GATGTTAAAC TG-------- ----------

Mouse ATGCCGGAGC CACCAGCATT GAAGTTAAAC TGGAGAACTA TGGATTTGAT

Guinea_Pig ATGCTGGTGC CACAAGCATA GATGTGAAAC TGGAAAACCA TGGATTTGAT

Rabbit ATGCGGGTGC CACAAGCATA GATGTTAAAT TGGAAAACTA TGGATTTGAT

Cow ATGCCCGTGC CACAAGCATA GATGTTAAAC TGGAAAACTA TGGATTTGAT

Dog ATGCAGGTGC CACAAGCATA GATGTTAAAC TGGAGAACTA TGGATTTGAT

Horse ATGCTGGTGC CACAAGCATA GATGTTAAAC TGGAGAACTT TGGGTTTGAT

Pig ATGCTGGTGC CACAAGCATA GATGTTAAAC TGGAGAACTA TGGATTTGAT

Elephant ATGCTGGTGC TACAAGCATA GATGTTAAAC TGGAGAACTA TGGATTTGAT

Opossum ATGCTGGTGC CACAAATATA GATGTTAAAC TGGAAAATTA TGGATTTGAT

Platypus ACGCCGGAGC CACCAGCATC GAGGTGAAAC TGGAGAACTA CGGCCTGGAG

Chicken ATGCCAGTGC CACTACTATT GACATTAGAC TGGAGAATTA CGGGTTTGAC

Zebra_Finch ATGCCAGTGC TACAGGCATT GATATTAAAC TGGAGAATTA TGGGTTTAGC

Zebrafish ATGCTGGTTC CTCAAGCCTT GAAGTCAAAC TGGAGAATTA TGGACTGGAT

Fugu ATGCTGGGGC CACCAGCATT GACGTCAAGC TGGAGAACTA TGGTCTGGAG

Frog ATGCTAACGC GACCAGCATT GAAATTAAGT TGGAGAATTT TGGATTTGAC

Human AAAATTGAGG TGCGAGATAA CGGGGAGGGT ATCAAGGCTG TTGATGCACC

Chimpanzee AAAATTGAGG TGCGAGATAA CGGGGAGGGT ATCAAGGCTG TTGATGCACC

Gorilla ---------- ---------- ---------- ---------- ----------

Marmoset AAAATTGAGG TTCGAGATAA CGGGGAGGGT ATCAAGGCTG CTGATGCACC

Orangutan ---------- ---------- ---------- ---------- ----------

Mouse AAAATTGAGA TTCGGGACAA TGGTGAGGGC ATCAAGGCTG TAGATGTCCC

Guinea_Pig AAAATTGAGG TCCGAGACAA TGGTGAAGGT ATCAAGGCTG TGGATGCCCC

Rabbit AAAATTGAGG TGCGAGACAA TGGTGAAGGT GTCAAGGCTG TTGATGCCCC

Cow AAAATTGAGG TGCGAGACAA TGGTGAAGGT ATCAAGGCTA TTGATGCACC

Dog AAAATTGAGG TACGAGACAA TGGTGAGGGT ATCAAGGCCA TTGATGCACC

Horse AAAATTGAGG TGCGAGACAA TGGTGAGGGC ATCAAAGCCA TTGATGCACC

Pig AAAATTGAGG TGCGAGACAA TGGTGAGGGT ATCAAGGCTG TTGATGCACC

Elephant AAAATTGAGG TGCGAGACAA TGGTGAGGGT GTCAAGGCCG TTGATGCACC

Opossum AAGATAGAAG TTCGAGACAA TGGTGATGGA ATCAAAGCTA TTGATGCACC

Platypus AAGATCGAAG TGCGTGACAA CGGGCAGGGA ATCCGTGCGG TGGACGCGCC

Chicken AAAATAGAAG TAAGAGACAA TGGCAATGGA ATCAAGGTCG CTGATGTTCC

Zebra_Finch AAAATCGAAG TAAGAGACAA TGGCAGTGGA ATTAAGGTTG ATGATGTTCC

Zebrafish CGCATTGAAG TGCGAGACAA TGGTTCTGGT ATTAAAGCTA CTGATGTTTC

Fugu CGAATAGAGG TCCGTGATAA CGGTCATGGA ATCAAAGCCG CGGACACCCC

Frog AAAATTGAAG TGCGGGATAA TGGAAAAGGT ATCAAAAGTG ATGACACCCC

Human TGTAATGGCA ATGAAGTACT ACACCTCAAA AATAAATAGT CATGAAGATC

Chimpanzee TGTAATGGCA ATGAAGTACT ACACCTCAAA AATAAATAGT CATGAAGATC

Gorilla ---------- ---------- ---------- ---------- ----------

Marmoset TGTGATGGCA ATGAAGTACT ATACCTCAAA AATAAATAGT CATGAAGATC

Orangutan ---------- ---------- ---------- ---------- ----------

Mouse TGTAATGGCA GTGAAGTACT ACACCTCGAA GATCAGCAGT CATGAAGACC

Guinea_Pig TGTGATGGCA GTGAAATACT ATACCTCCAA AATAAGCAGT CACGAAGATC

Rabbit CGTAATGGCA GTGAAGTACT ATACCTCAAA AATAAACAGT CATGAGGATC

Cow TGTAATGGCA GTAAAGTACT ACACCTCCAA AATAAGTAGT CATGAAGACC

Dog TGTAATGGCA GTAAAATACT ATACTTCAAA AATAAATAGT CATGAAGATC

Horse AGTGATGGCA ATAAAGTACT ACACCTCAAA AATAAACAGT CATGAAGATC

Pig TGTAATGGCA GTGAAGTACT ACACCTCAAA AATAAATAGT CATGAAGACC

Elephant TGTAATGGCA ATGAAGTACT ATACCTCAAA AATAAATAGT CATGAAGATC

Opossum TGTGATGGCA ATAAAGCATT ATACCTCAAA AATAAACAGT CATGAAGACC

Platypus CGTCATGGCG GTCAGGCACT ACACCTCCAA GATCAGCAGC CACGAGGACC

Chicken TGTTATGGCA ATTAAACACT ACACCTCAAA AATAAGCTCT TCTGAGGATC

Zebra_Finch AGTTATGGCA ATTAAACACT ACACCTCAAA AATCAGCTCT TCTGAGGACC

Zebrafish TGTGATGGCT GTAAAACACT ATACGTCTAA AATCTCTTGC CATGAAGACC

Fugu CGTGATGGCC GTCAGGCATT TCACGTCAAA GATCTGCACC CACGATGATC

Frog TGTTATGGGC GTCAAACATT ATACTTCTAA AATTAACAGC CATGATGACT

Human TTGAAAATTT GACAACTTAC GGTTTTCGTG GAGAAGCCTT GGGGTCAATT

Chimpanzee TTGAAAATTT GACAACTTAC GGTTTTCGTG GAGAAGCCTT GGGGTCAATT

Gorilla ---------- ---------- ---------- ---------- ----------

Marmoset TTGAAAATTT AACAACTTAT GGTTTTCGTG GAGAAGCCTT GGGGTCAATT

Orangutan ---------- ---------- ---------- ---------- ----------

Mouse TTGGAAATCT GACAACTTAT GGTTTTCGTG GTGAAGCCTT GGGGTCAATA

Guinea_Pig TTGAAAACTT GAAAACCTAT GGTTTTCGTG GGGAAGCATT AGGGTCAATT

Rabbit TTGAACGTTT GACAACTTAC GGTTTTCGTG GCGAAGCGTT GGGGTCAATT

Cow TTGAAAATTT GACAACGTAT GGTTTTCGTG GAGAAGCCTT GGGGTCAATT

Dog TTGAAAATTT GACAACTTAC GGTTTTCGTG GAGAAGCCTT GGGATCAATT

Horse TTGAAAATTT GACGACTTAC GGTTTTCGTG GAGAAGCTTT GGGCTCAATT

Pig TTGAAAATTT GACAACTTAT GGTTTTCGTG GAGAAGCCTT GGGGTCAATT

Elephant TTGAAAATTT GACAACTTAT GGTTTTCGTG GAGAAGCCTT GGGGTCAATT

Opossum TTGAAAATTT GACGACTTAT GGTTTTCGTG GGGAAGCTTT GGGGTCAATT

Platypus TGGACCAGCT GACCACCTAC GGCTTCCGCG GGGAGGCCCT GGGCTCCGTG

Chicken TTGAAAGGCT GACAACATAT GGTTTCCGTG GGGAAGCCCT GGGATCAATT

Zebra_Finch TTGAAAGGCT GACAACATAC GGTTTCCGTG GGGAAGCTTT GGGATCGATT

Zebrafish TGGAACAGCT AGAGACTTAC GGCTTCAGAG GAGAAGCTCT GGCATCCATA

Fugu TGGAGCATCT GGAGACGTAC GGCTTCCGAG GAGAGGCCCT GGGTTCGGTC

Frog TGGAAACATT AGAAACATAT GGCTTTCGTG GGGAAGCCCT CGCTTCAATC

Human TGTTGTATAG CTGAGGTTTT AATTACAACA AGAACGGCTG CTGATAATTT

Chimpanzee TGTTGTGTAG CTGAGGTTTT AATTACAACA AGAACGGCTG CTGATAATTT

Gorilla ---------- ---------- ---------- ---------- ----------

Marmoset TGTTGTGTAG CTGAGGTTTT AATTACAACA AGGACGGCTG CTGATAATTT

Orangutan ---------C CCCAGGTTTT AATTACAACA AGAACGGCTG CTGATAATTT

Mouse TGTAATGTTG CGGAGGTGGT AGTTACAACA AGGACATCTG CTGATGACTT

Guinea_Pig TGTTGCGTAG CTGAGGTTTT AATTACAACA AGAACAGCTG ATGATAATTT

Rabbit TGTTGTGTTG CTGAGGTTTC AATTACAACA AGGACAGCTG CTGATAATTT

Cow TGTTGTATAG CCGAGGTTTC AATTACAACA AAGACAGCTG CTGATAACTT

Dog TGTTGTGTAG CTGAGGTTTT AATTACTACA AGAACTGCTT CTGATAATTT

Horse TGTTGTGTCG CTGAGGTCTC AATTATTACA AGGACGGCTG CTGATAATTT

Pig TGTTGTGTAG CCGAGGTTTT AATTACAACA AGGACAGCTT CTGATAACTT

Elephant TGTTGTGTAG CAGAGGTTTT AATTACAACA AGGACAGCTG CTGATAATTT

Opossum TGTTGTATAG CTGAGGTTTT AATTACAACA AAGACAGCTG CTGATAACTT

Platypus TGCTGCATCG CCGAGGTTTT GATCACGACA AAGACAGCGG CGGACAACTT

Chicken TGTTGCATAT CAGAGGTTTT GGTCACAACT AGGACAGCTG ATGATGATAT

Zebra_Finch TGCAGCATAT CAGAAGTTTT GGTCACAACA AAGACAGCTG CTGATGATTT

Zebrafish TGTGCCATCT CAGAGGTGAT TATTACTACC AAAACTGCAG ATGACGATTT

Fugu TGTGCCGTGG CTGAGGTGAC AGTCATCACA AAGACAGAGG AGGACGACGT

Frog TGCTCTGTGG CAGAGGTTCA TATTGCTACA AAGACTCTTG AAGATGATTT

Human TAGCACCCAG TATGTTTTAG ATGGCAGTGG CCACATACTT TCTCAGAAAC

Chimpanzee TAGCACCCAG TATGTTTTAG ATGGCAGTGG CCGCATACTT TCTCAGAAAC

Gorilla ---------- ---------- ---------- ---------- ----------

Marmoset TAGCACCCAG TATGTTTTAG ATGGCAGTGG CCACATACTT TCTCAGAAAC

Orangutan TAGCACCCAG TATGTTTTAG ATGGCAGTGG CCACATACTT TCTCAGAAAC

Mouse TAGCACTCAG TATGTTTTAG ATGGCAGTGG CCACATACTT TCTCAGAAGC

Guinea_Pig TAGCACCCAG TATGTTTTAG ATGGCAGTGG CCACATAGTT TCTCAAAAAC

Rabbit TAGCACCCAG TATGTTTTAG ATAGCAGTGG CCACATAGTT TCTCAAAAAC

Cow TAGTACCCAG TATGTTTTAG ATGGTGGTGG TCACATAATT TCTCAAAAAC

Dog TAGCACCCAG TATGTTTTAG ATGGTAGTGG CCACATAATT TCTCAGAAAC

Horse TAGCACCCAG TATGTTTTAG ATGGTAGTGG CCACATAATT TCTCAAAAAC

Pig TAGCACCCAG TATGTTTTAG ATGGCAGTGG CCACATAATT TCTCAAAAAC

Elephant TAGCACCCAG TATGTTTTAG ATGGCAGTGG CCACATAATT TCTCAAAAAC

Opossum TAGCACCCAG TATGTTTTAG ATAGCAGTGG TCATATAGTT TCTCAAAAGC

Platypus CAGCACCCAG TATGTTTTGG ACAGCAGCGG CCACGTCACT GCGCAGAAAC

Chicken CAGCACTCAG TATGTTTTGG ATAGTAGTGG GCATGTAACG TCCCAAAAGC

Zebra_Finch CAGCATTCAG TATGCTTTGG ATAGCAATGG ACATGTAACC TCTAAAAAGC

Zebrafish TAGCATTCAG TATTCAGTGG ATCACAATGG ACAGATCGTC TCTCAGAAGC

Fugu CAGCACTCAG TACACACTTA ATTTCACAGG AGGGATTGTT TCAAAGAAGC

Frog CAGCAAGTTG TATATCTTAG ATAGCAGTGG TCATGTTGTT TCCCAAAAGC

Human CTTCACATCT TGGTCAAGGT ACAACTGTAA CTGCTTTAAG ATTATTTAAG

Chimpanzee CTTCACATCT TGGTCAAGGT ACAACTGTAA CTGCTTTAAG ATTATTTAAG

Gorilla ---------- ---------- ---------- ---------- ----------

Marmoset CTTCACATCT TGGTCAAGGT ACAACCGTAA CTGCTTTAAG ATTATTTAAG

Orangutan CTTCACATCT TGCTCAAGGT ACAACTGTAA CTGCTTTAAG ATTATTTAGG

Mouse CTTCACATCT TGGTCAAGGT ACAACTGTAA CTGCTCTAAA GTTGTTTAAG

Guinea_Pig CTTCACATCT TGGTCAAGGT ACAACTGTAA CGGCTTTAAG ATTATTTAAG

Rabbit CTTCACATCT TGGTCAAGGT ACAACTGTAA CTGCCTTAAG ATTGTTTAAG

Cow CTTCACATCT TGGTCAAGGT ACAACTGTAA CTGCTTTACG ATTATTTAAG

Dog CTTCACATCT TGGTCAAGGT ACAACTGTAA CTGTTTTAAA ATTGTTTAAG

Horse CTTCACATCT TGGTCAAGGT ACAACTGTAA CTGCTTTAAA ATTGTTTAAG

Pig CTTCACATCT TGGTCAAGGT ACAACTGTAA CTGCTTTAAA ATTGTTTAAG

Elephant CCTCACATCT TGGTCAAGGT ACAACTGTTA CTGCTTTAAG ATTATTTAAG

Opossum CTTCACATCT TGGACAAGGT ACAACAGTAA CTGCACTGAA ATTATTTAAG

Platypus CCTCACACCT CGGACAAGGG ACCACCGTCA CGGTCCTGCG ACTGTTCAAG

Chicken CTTCCCATCT TGGTCAAGGT ACAACAGTTA CAGTCCTCAA GTTGTTTAAG

Zebra_Finch CTTCCCATCT TGGGCAAGGT ACTACAGTAA CAGTCCTAAA TTTGTTTAAG

Zebrafish CATCTCATCT TGGCCAGGGC ACCACGGTGT GTGCGGCAAA TCTGTTCAAG

Fugu CATCTCACTT AGGTCAAGGA ACAACTGTGA ACGTGCTGAA GCTTTTCAAG

Frog CTTCCCATCT TGGTCAGGGT ACTACTGTCA GTGTGCATAA ACTGTTCAAG

Human AATCTACCTG TAAGAAAGCA GTTTTACTCA ACTGCAAAAA AATGTAAAGA

Chimpanzee AATCTACCTG TAAGAAAGCA GTTTTACTCA ACTGCAAAAA AATGTAAAGA

Gorilla ---------- ---------- ---------- ---------- ----------

Marmoset AATCTACCTG TAAGAAAGCA GTTTTACTCA ACTGCTAAAA AATGTAAAGA

Orangutan AATCTACCTG TAAGAAAGCA GTTTTACTCA ACTGCAAAAA AATGTAAAGA

Mouse AATCTGCCTG TAAGAAAACA ATTTTACTCA ACAGCTAAAA AGTGTAAAGA

Guinea_Pig AATCTGCCTG TAAGAAAGCA GTTTTACTCA ACTGCTAAAA AATGTAAAGA

Rabbit AATCTGCCTG TAAGAAAGCA ATTTTACTCA ACTGCTAAAA AATGTAAAGA

Cow AATCTACCTG TAAGAAAGCA ATTTTACTCA ACTGCTAAAA AATGTAAAGA

Dog AATCTACCTG TAAGAAAGCA GTTCTATTCA ACTGCTAAAA AATGTAAAGA

Horse AATCTACCTG TAAGAAAGCA ATTTTATTCA ACTGCTAAAA AATGTAAAGA

Pig AATCTACCTG TCAGAAAGCA ATTTTACTCA ACTGCTAAAA AATGTAAAGA

Elephant AACCTACCTG TAAGAAAGCA ATTTTATTCA ACTGCTAAAA AATGTAAAGA

Opossum AATCTTCCTG TAAGAAAACA ATTTTATTCA ACAGCTAGAC GATGTAAGGA

Platypus AACCTCCCCG TGCGGAAGCA GTTCTTCTCC ACGGCCCAGA AGCGCAAAGA

Chicken AATCTTCCTG TGAGAAAACA GTTTTACTCA ACAAATAGAA AATGTAAGGA

Zebra_Finch AATCTTCCTG TAAGAAAGCA GTTTTACTCA ACAAACAGAA AATGTAAGGA

Zebrafish AACCTTCCGG TTAGACGGCA GTATTATTCC AACACTAAAA AATGTAAAGA

Fugu AATCTCCCAG TGAGACGACA ATACTATTCT TCTACCAAGA AATGCAAAGA

Frog AATTTACCTG TAAGAAAGCA ATATTATTCT ACAATCAAGA AGTGTAAAGA

Human TGAAATAAAA AAGATCCAAG ATCTCCTCAT GAGCTTTGGT ATCCTTAAAC

Chimpanzee TGAAATAAAA AAGATCCAAG ATCTCCTCAT GAGCTATGGT ATCCTTAAAC

Gorilla ---------- ---------- ---------- ---------- ----------

Marmoset TGAAATAAAA AAGATCCAAG ATCTCCTCAT GAGCTATGGT ATCCTTAAAC

Orangutan TGAAATAAAA AAGATCCAAG ATCTCCTCAT GAGCTATGGT ATCCTTAAAC

Mouse TGAACTAAAA AATGTACAGG ACCTTCTCAT AAGCTACGGT GTCCTGAAAC

Guinea_Pig TGAAATAAAA AAGGTCCAGG ATCTCCTCAT AAGCTATGGT ATCATTAAAC

Rabbit TGAAATTAAA AAGGTTCAAG ATCTCCTCAT AAGCTATGGT ATCCTTAAGC

Cow TGAAATTAAA AAGATTCAAG ATCTCCTTAT AAGCTATGGT ATACTTAAAC

Dog TGAAATAAAA AAGGTCCAGG ATCTCCTCAT AAGCTATGGT ATACTTAAAC

Horse TGAAATAAAA AAAGTCCAAG ATCTCCTCAT AAGCTATGGT ATACTTAAAC

Pig TGAAATTAAA AAGGTCCAAG ATCTCCTCAT AAGCTATGGT GTACTTAAAC

Elephant TGAAATAAAA AAAGTCCAAG ATCTCCTCAT AAGCTATGGT ATTCTTAAAC

Opossum TGAGTTAAAA AAAATCCAAG ACCTTCTTAT AGCCTATGGC ATTCTCAAAC

Platypus GGAGCTGAAG CGCGTGCAGT CCATCCTCAT GGCCTACGGC CTCCTCAAGC

Chicken AGAACTGAAA AAAGTCCACG ACCTCCTGAT AGCATATGGC ATCATAAAGC

Zebra_Finch AGAGCTGAAA AAAGTCCAAG ATCTACTGAC AGCATATGGT ATCATAAAAC

Zebrafish TGAGCTTAAG AGGGTCCAGA ACTTACTCAT GGCTTATGCT GTAATAAAGC

Fugu GGAACTGAAG AAAGTGCAGG ACCTGCTGTT GGCTTACGCC ATTATCAAGC

Frog GGAGATTAGA AATATCCAGG AGCTACTGAT GGCTTATGGA ATCGTAACAC

Human CTGACTTAAG GATTGTCTTT GTACATAACA AGGCAGTTAT TTGGCAGAAA

Chimpanzee CTGACTTAAG GATTGTCTTT GTACATAACA AGGCAGTTAT TTGGCAGAAA

Gorilla ---------- ---------- ---------- --GCAGTTAT TTGGCAGAAA

Marmoset CTGACTTAAG GATTGTTTTT GTACATAACA AGGCAGTTAT TTGGCAGAAA

Orangutan CTGACTTAAG GATTGTTTTC GTACATAACA AGGCAGTTAT TTGGCAGAAA

Mouse CTGATGTGAG GATTACTTTT GTACATAATA AGGCAGTTAT TTGGCAGAAA

Guinea_Pig CTGACTTAAG GATTCTTTTT GTACATAATA AGGCAGTTAT TTGGCAGAAA

Rabbit CTGACTTAAG GATTGTTTTT GTACATAACA AGGCAGTCGT TTGGCAAAAA

Cow CTGATGTAAG GATTGTTTTT ATACATAACA AGGCAATTAT TTGGCAGAAA

Dog CTGAGTTAAG GATTGTGTTT ATACATAACA AGGCAGTTAT CTGGCAGAAG

Horse CTGACTTAAG GATTGTTTTT GTACATAACA AGGCAGTTAT TTGGCAGAAA

Pig CTGAAGTAAG GCTTGTTTTT ATACACAACA AGGCAATTAT TTGGCAGAAA

Elephant CTGACTTAAG GATTATTTTT ATACATAACA AGGCAGTTAT TTGGCAGAAA

Opossum CTGAATTAAG GATTATTTTT ACACATAACA AGGTAATTAT TTGGCAGAAA

Platypus CCCAGCTCAG GGTCGTCTTC ACGCACAACA AGGCCGTGGT GTGGCAGAAG

Chicken CAGAACTGAG AATAACCTTT GTACATAATA AGGCAGTTAT TTGGCAGAAG

Zebra_Finch CAGACCTGAG GATAACATTA ACACATAATA AGGCTGTTAT TTGGCAGAAG

Zebrafish CTGAGTTACG GGTCACGCTG AGCCACAACA AGGCTGTGGT CTGGCAGAAG

Fugu CTGAACTGAG GCTCACTTTG GTTCACAACA AAGTGGTGTT GTGGCAGAAG

Frog CAAATTTAAG GATTCTGTTC ACCAATAATA AGGCTCTTGT ATGGCAAAAA

Human AGCAGAGTAT CAGATCACAA GATGGCTCTC ATGTCAGTTC TGGGGACTGC

Chimpanzee AGCAGAGTAT CAGATCACAA GATGGCTCTC ATGTCAGTTC TGGGGACTGC

Gorilla AGCAGAGTAT CAGATCACAA GATGGCTCTC ATGTCAGTTC TGGGGACTGC

Marmoset ACCAGAGTAT CAGGTCACAA GATGGCTCTC ATGTCGGTTC TGGGGACTGC

Orangutan AGCAGAGTAT CAGATCACAA GATGGCTCTC ATGTCAGTTC TGGGGACTGC

Mouse AGCAGAGTTC CAGATCACAG GATGGCTCTA ATGTCGGTTC TGGGAACTGC

Guinea_Pig AGTAGAGTGC CTGACCACAA GATGGCTCTC ATGTCGGTTC TGGGGACTGC

Rabbit ACTAAAGTAT CAGACCATAA GATGGCTCTC ATGTCAGTTC TGGGGACTGC

Cow ACTAGAGTAT CAGATCATAA AATGGCTCTC ATGTCTGTTC TGGGGACTAC

Dog ACTAGAGTAT CCGATCGTCA GGTGGCTCTC CGGGCTGTTC TGGGGACGGC

Horse ACGAGAGTAT CAGATCATAA GATGGCTCTC ATGTCTGTTC TGGGGACTGC

Pig ACTAGAGTAT CAGATCATAA AATGGCTCTC ATGTCTGTTC TGGGGACTGC

Elephant ACCAGAGTAT CAGATCACAA GATGGCTCTT ATGTCTGTTC TGGGAACTGC

Opossum AGCAGGGTTT CAGATCATAA AACAGCTTTG ATGGCAGTTC TGGGAACTTC

Platypus AGCAGAGTGT CGGACCATAA AATGGCCCTC ATGTCCATCT TGGGGAAAGC

Chicken AACAGGGTGT CAGATCACAA AATGGCCTGT ATGTCAGTTC TGGGAACAGC

Zebra_Finch ACCAGGGTAT CAGATCACAA AATGGCCTGT ATGTCAATTC TGGGAACAGC

Zebrafish TCCAGGGTGT CGGATCACCG AACTGCCCTC ATGGCCGTGC TCGGAGCAGC

Fugu GCCAAAGTCC CCGATCACAG GAGCGCTCTC GTTGCCACGT TGGGGCCCGG

Frog TCAAAGACGT CTGACCATAA GATGGCATTT ATGTCAGTTG TGGGAAGTGC

Human TGTTATGAAC AATATGGAAT CCTTTCAGTA CCACTCTGAA GAATCTCAGA

Chimpanzee TGTTATGAAC AATATGGAAT CCTTTCAGTA CCACTCTGAA GAATCTCAGA

Gorilla TGTTATGAAC AATATGGAAT CCTTTCAGTA CCACTCTGAA GAATCTCAGA

Marmoset TGTTATGAAC AATATGAAAT CCTTTGAGTA CCACTCTGAA GACTCTCAGA

Orangutan TGTTATGAGC AATATGGAAT CCTTTCAGTA CCACTCTGAA GAATCTCAGA

Mouse TGTCATGGGC AACATGGAAT CTGTTGAGCA GCACTGTGAA CAGTCGCAGA

Guinea_Pig CATTATGGGC AACTTGGAGT CTTTTCAGTA CCACTCAGAA GAATCTCAGA

Rabbit TGTTATGGGC AACATGGAAT CCTTTCAGTG CTGCTCTGAA GAATCTCAGG

Cow TGTTATGGGC AATATGGAAT CCTTTCAGTA CCACTGTGAA GAATCTCAGA

Dog CGTGATGGGC AACATGGAAC CGTTTCAGTA CCACCCTGAA GACTCACAGA

Horse TGTTATGGGC AATATGGAAT CCGTGCAGTA CCACTCAGAA GAATCTCAGA

Pig TGTTATGGGG AATATGGAAG CCTTTCAGTA CCACTCTGAA GAATCTCAGA

Elephant CATTATGGGC AATATGGAAT CCTTTCAGCA CCACTCTGAA GAGTCTGAGA

Opossum CATTATGAGC AATATGGCAG CCTTTGAGCA TCACTATGAA GAATCTCAGA

Platypus CGTCATGGAC AACCTGGCAC CTTTCCAGTA CCAGTCCGCG GACTCAGAGA

Chicken CGTTATGGGC AGTATGGTAC CTTTTCAACA CTGCTGTGAA GATCCTGAGA

Zebra_Finch CGTTATGAGC AGTATGGTAC CTTTTCAACA CTGCTGCGAA TATCCTGAGA

Zebrafish GTCCGTAGCC AACATGCTCC CCGTCCAGCA TCACCAGGAG CAACCAGAGA

Fugu TGTCGTTGTC AACTTGCTCC CTTGCCACCA TCGCCAGGAG CAACCAGAGA

Frog CATCATGAAT TGCATGGTTC CTATTCAATA CCAATCCGAA GACCCAAAGA

Human TTTATCTCAG TGGATTTCTT CCAAAGTGTG ATGCAGACCA CTCTTTCACT

Chimpanzee TTTATCTCAG TGGATTTCTT CCAAAGTGTG ATGCAGACCA CTCTTTCACT

Gorilla TTTATCTCAG TGGATTTCTT CCAAAGTGTG ATGCAGACCA CTCTTTCACT

Marmoset TTTATCTCAG TGGATTTCTT CCAAAGTGTG ATGCAGACCA CTCTTTCACC

Orangutan TTTATCTCAG TGGATTTCTT CCAAAGTGTG ATGCAGACCA CTCTTTCACT

Mouse TTTACCTAAG TGGATTCTTC CCAAAGCACG ATGCAGACCA CAATTCCACA

Guinea_Pig TTCATTTAAG TGGATTCCTT CCAAAGCTTG ATGCAGATCA CTCTTCAACA

Rabbit TCTCTCTAAG TGGATTTCTT CCAAAGCGTG ATGCAGACCA CTCTTTGACA

Cow TTTTTCTAAG TGGATTTCTT CCAAAGCATG ATGCAGACCA CTCTTGCACA

Dog TTTATCTAAG TGGATTTCTT CCAAAGCATG ATGTAGACCA CTCTTCAACG

Horse TTTATCTAAG TGGATTTCTT CCAAAGCATG ATGCGGACCA CTCTTTCACA

Pig TTTATCTAAG TGGATTTCTT CCAAAACATA ATGCAGACCA CTCTGTCACA

Elephant TTTATCTCAG TGGATTTCTT CCAAAGCATG ATGCAGACCA CTCTCTCACC

Opossum TTTATTTAAG TGGATTTCTT CCAAAGCTTG ACTCTGATCA CTCTTTGACA

Platypus TGAGTCTGAG CGGATTTCTC CCCAAGCCCG ACGCGGACCA CTCCGTGACA

Chicken TAAATCTTTC TGGATTTCTT CCAAAAGCTG AGTCAGACAG CTCTTTGACA

Zebra_Finch TAAATCTTTC TGGATTTCTC CCAAAAGCTG AGTCAGACAC TTCTTTGACA

Zebrafish TCCACATCGA TGGATTCTTT CCCAAACCGG GCTCAGATTT GAATTCCACC

Fugu TTTTTTTAGA AGGCTTTTTT CCAAAGCCGG GAGCAGACTA CTCCTCTACA

Frog TTTTTATTAA TGGCTATCTA CCCAAGCCAT ATGCCGATAA CACTACAACC

Human AGTCTTTCAA CACCAGAAAG AAGTTTCATC TTCATAAACA GTCGACCAGT

Chimpanzee AGTCTTTCAA CACCAGAAAG AAGTTTCATC TTCATAAACA GTCGACCAGT

Gorilla AGTCTTTCAA CACCAGAAAG AAGTTTCATC TTCATAAACA GTCGACCAGT

Marmoset AGTCTTTCAA CCCCAGAAAG AAGTTTCATC TTCATAAACA GTCGACCAGT

Orangutan AGTCTTTCAA CACCAGAAAG AAGTTTCATC TTCATAAACA GTCGACCAGT

Mouse AGTCTTTCAA CCCCAGAGAG AAGTTTCATC TTTATTAATA GTCGGCCAGT

Guinea_Pig AGTCTTTCGA CTCCAGAGAG AAGTTTCATC TTTATAAATA ATCGACCCAT

Rabbit AGTCTTTCAA CTCCAGAAAG GAGTTTCATC TTTATAAACA GTCGACCAAT

Cow AGTCTTTCAA CCCCAGAAAG GAGTTTCATC TTTATAAACA GTCGACCAGT

Dog AGTCTTTCAA CCCCAGAAAG GAGTTTTATC TTTATAAACA ATCGACCAGT

Horse AGTCTTTCAA CCCCAGAAAG GAGTTTCATC TTTATCAACA ATCGACCAGT

Pig AGTCTTTCAA CCCCAGAAAG GAGTTTCATC TTTATAAACA GTCGACCAGT

Elephant AGTCTTTCAA CTCCAGAAAG GAGTTTCATC TTTATAAATA ATCGACCAGT

Opossum AATCTTTCAA CCCCAGAAAG GAGTTTTATT TTCATAAATA ATCGACCAGT

Platypus AGCTTGTCCA CCGCAGAAAG AAGTTTCCTG TTCATCAACA GCCGCCCAGT

Chicken AGTCTCTCAA GCTCAGAACG GAGTTTTATT TTTGTAAATA GTCGTCCAGT

Zebra_Finch AGCCTTTCAA GTTCAGAAAG GAGTTTCATT TTTATAAACA ATCGTCCGGT

Zebrafish AGCTCCTCTA CGACTGATAA GTCATTCATC TTTGTAAACA GTCGACCTGT

Fugu AGCTCCTCAC ACCCTGACAA AACATTTATA TTCGTCAACA ACAGACCCGT

Frog AGCCTCTCAA GTTCAGAAAA AAGTTTTCTC TTTATAAACC GTCGACCTAT

Human ACATCAAAAA GATATCTTAA AGTTAATCCG ACATCATTAC AATCTGAAAT

Chimpanzee ACATCAAAAA GATATCTTAA AGTTAATCCG ACATCATTAC AATCTGAAAT

Gorilla ACATCAAAAA GATATCTTAA AGTTAATCCG ACATCATTAC AATCTGAAAT

Marmoset ACATCAAAAA GATATCTTAA AGTTAATCCG ACATCATTAC AATCTGAAAT

Orangutan ACATCAAAAA GATATCTTAA AGTTAATCCG ACATCATTAC AATCTGAAAT

Mouse ACATCAAAAA GATATACTAA AGTTAATTCG ACGCTATTAT AATCTGAAGT

Guinea_Pig CCATCAGAAA GACATATTAA AGTTAATCCG ACAGTACTAT AATCTGAAGT

Rabbit ACATCAAAAA GACATATTAA AGTTAATCCG ACAGTATTAC ACTCTGAAGT

Cow ACACCAAAAA GATATATTGA AGCTAATCCG ACATTATTAC AATCTGAAAT

Dog ACATCAAAAA GACATATTAA AGCTAATCCG ACATTATTAC AATCTGAAGT

Horse ACACCAAAAA GACATATTAA AGTTAATCCG ACATTATTAC AATCTGAAGT

Pig ACACCAAAAA GACATATTAA AGCTTATCCG ACATTATTAC AATCTGAAAT

Elephant ACATCAAAAA GACATATTAA AGTTAATTCG GCATTATTAC AATCTGAAGT

Opossum ACATCAGAAA GACATCCTAA AGTTAATTCG TCATTACTAC ACTGAGAAAT

Platypus GCGCCAGAGA GACATCTTAA AGGTCATTCG CCAGTACTGC GGTCGCCGGG

Chicken TCATCAGAAG ---------- --GAAATACT AAAGTACTAC AGTGTGCTGA

Zebra_Finch TCTTCAGAAG ---------- --TTAATTCG ACAATACTAC AGTCAGGTGA

Zebrafish GCATCATAAA GAGATACTTA AGCTTATCAA GCAGTATTAC ACCAGTGCTC

Fugu CCACCACAAA GACATCATGA AGCTGTTGCG GCAACACTAC ACTGCACAGT

Frog TTATCACAAA GAAATACTGA AGATGGTGCG TTGGTATCAC AACCAGAGTT

Human GCCTAAAGGA ATCTACTCGT TTGTATCCTG TTTTCTTTCT GAAAATCGAT

Chimpanzee GCCTAAAGGA ATCTACTCGT TTGTATCCTG TTTTCTTTCT GAAAATCGAT

Gorilla GCCTAAAGGA ATCTACTCGT TTGTATCCTG TTTTCTTTCT GAAAATCGAT

Marmoset GCCTAAAGGA ATCTACTCGT TTGTATCCTA TTTTCTTTCT GAAAATTGAT

Orangutan GCCTAAAGGA ATCTACTCGT TTGTATCCTG TTTTCTTTCT GAAAATCGAT

Mouse GCCTAAAGGA GTCTACCCGC TTGTATCCCA TTTTCTTTCT GAAAATTGAT

Guinea_Pig GCCTCAAGGA ATCTTCTCGT TTGTATCCCA TTTTCTTTCT GAAAATTGAT

Rabbit GTCTAAAGGA ATCTACTCGT TTGTATCCCA TTTTCTTTCT GAAAATTGAT

Cow GTCTAAAGGA ATCTACTCGT TTGTATCCCA TTTTCTTTCT GAAAATTGAC

Dog GCCTAAAGGA AGCCAGTCGT TTGTATCCCA TTTTCTTTCT GAAAATTGAT

Horse GCCTAAAGGA ATCTACTCGT TTGTATCCCA TTTTCTTTCT GAAAATTGAT

Pig GCCTAAAGGA AACTACTCGG TTGTATCCCA TTTTCTTTCT GAAAATTGAT

Elephant GCCTAAAGGA ATCCACTCGT TTGTATCCCA TTTTTTTTCT AAAAATTGAT

Opossum GCCTAAAAGA GTCAACTCGT TCTTATCCAG TTTTCTTCAT GAAAATTGTT

Platypus GCCAGAAGGA GACCACTCAC CTTTACCCCG TCTTCTTCGT CAACATCGTC

Chicken TGCAAAAGGA CAGCACACGC TTATACCCTG TGTTCTTTCT GAATATTACT

Zebra_Finch CACAAAAGGA ATGTACTCGT TCATATCCTG TTTTCTTTTT GAGTATTACT

Zebrafish AGTCTAACAG TGAAAGCCGC CGATATCCCA CTTTTATGAT GAACATCACC

Fugu ATCCGGATGA TGGAAGCCGT ---TATCCCG TCCTTATGCT CAGCCTCACC

Frog TAAACAAAGG GACCCCTCGC TGCTATCCAG TATTTTTTAT GAATATTGAA

Human GTTCCTACAG CTGATGTTGA TGTAAATTTA ACACCAGATA AAAGCCAAGT

Chimpanzee GTTCCTACAG CTGATGTTGA TGTAAATTTA ACACCAGATA AAAGTCAAGT

Gorilla GTTCCTACAG CTGATGTTGA TGTAAATTTA ACACCAGATA AAAGTCAAGT

Marmoset GTTCCTACAG CTGATGTTGA TGTAAATATA ACACCAGATA AAAGTCAAGT

Orangutan GTTCCTACAG CTGATGTTGA TGTAAATTTA ACACCAGATA AAAGTCAAGT

Mouse GTTCCATCAG CTGAGGTGGA TATAAATCTA ACACCAGATA AAAGTCAAGT

Guinea_Pig GTTCCTACAA CTGATGTGGA TGTAAATCTA ACACCAGATA AAAGTCAAGT

Rabbit GTTCCTACAG CTGATGTGGA TGTAAATTTA ACACCAGATA AAAGTCAAGT

Cow GTTCCAACAG CTGATGTAGA TGTGAATTTA ACACCTGATA AAAGTCAAGT

Dog GTTCCTACAG CTGATGTGGA TGTAAATATA ACACCAGATA AAAGTCAAGT

Horse GTACCTACCG CTGATGTGGA TGTAAATTTA ACACCAGATA AAAGTCAAGT

Pig GTTCCTACAG TTGATGTGGA TGTGAATTTA ACACCAGATA AAAGTCAAGT

Elephant GTGCCTACAG CTGATGTCGA TGTAAATTTA ACACCAGATA AAAGTCAAGT

Opossum GTGCCTGCAG CTGATGTAGA TGTAAATTTA ACACCTGATA AAAGCCAAGT

Platypus GTGCCCACGG CGTCGGTCGA CGTCAACCTG ACCCCTGATA AGAGTCGTGT

Chicken GTACCTGCCT CTGCTGTGGA TGTGAACGTA ACACCTGATA AAACTGAAGT

Zebra_Finch GTACCTGCCT CTGCTGTGGA TGTGAATATA ACACCTGATA AAACACAAGT

Zebrafish ATACCGGCCT CAACTGTGGA CGTTAACCTG ACACCTGATA AAACTGAAGT

Fugu GTGTCTCCAT CCTCGGTCGA TGTCAACCTG ACCCCGGACA AGACCCAGGT

Frog CTTCCTGCTT CATGTTTGGA TGTGAATTTA ACTCCAGATA AAACTCAAAT

Human ATTATTACAA AATAAGGAAT CTGTTTTAAT TGCTCTTGAA AATCTGATGA

Chimpanzee ATTATTACAA AATAAGGAAT CTGTTTTAAT TGCTCTTGAA AATCTGATGA

Gorilla ATTATTACAA AATAAGGAAT CTGTTTTAAT TGCTCTTGAA AATCTGATGA

Marmoset ATTATTACAG AATAAGGAAT CTGTTTTAAT TGCTCTTGAA AATCTGATGA

Orangutan ATTATTACAA AATAAGGAAT CTGTTTTAAT TGCTCTTGAA AATCTGATGA

Mouse GTTATTACAG AATAAGGAAT CTGTTTTAAT TGCTCTTGAA AATCTGATGA

Guinea_Pig ATTATTACAG AATAAGGAAT CTGTTTTAAT TGCTCTTGAA AATCTGATGA

Rabbit ATTATTACAG AATAAGGAAT CTGTTTTAAT TGCTCTTGAA AATCTGATGA

Cow ATTGTTACAG AATAAGGAAT CTGTTTTAAT CGCTCTTGAA AATCTGATGA

Dog GTTAATACAG AATAAGGAAG CTGTTTTAAT TGCTCTTGAA AATCTGATGA

Horse ATTATTACAG AATAAGGAAT CTATTTTAAT TGCTCTTGAA AATCTGTTGA

Pig ATTATTACAG AATAAGGAGT CTGTTTTAAT TGCTCTTGAA AATCTGATGA

Elephant ATTGCTACAG AATAAGGAAT CTGTTTTGAT TGCTCTTGAA AACCTGATGA

Opossum ATTATTACAA AATAAGGAAT CACTTTTAAT TGCTGTTGAA AATGTGTTGA

Platypus GTTCCTGCAA AACAAGGAGT CCGTGCTGCT GGCTGTGGAG ACGGTGCTGG

Chicken TCTGCTGCAG TATAAGGAGT CTGTCTTACT TGCTGTTGAA AATGTGTTGA

Zebra_Finch TTTGCTGCAT TATAAGGAAT CTGTCTTACT TGCAGTTGAA AATGTGTTGA

Zebrafish GATGCTACAG AATAAGGATG AAGTTCTGTT GTCAGTGGAG ACTATGCTAA

Fugu TCTTCTTCAC GACAAGGAGG CCGTGCTTAT CGCCCTGGAG GCATTTCTGG

Frog TATGCTTCAA AATAAGGAGT TTGTCCTACA TGCTGTTGAG AATGTCCTGA

Human CGACTTGTTA TGGACCATTA CCTAGTACAA ATTCTTATGA AAATAATAAA

Chimpanzee CGACTTGTTA TGGACCATTA CCTAGTACAA ATTCTTATGA AAATAATAAA

Gorilla CGACTTGTTA TGGACCATTA CCTAGTACAA ATTCTTATGA AAATAATAAA

Marmoset CGACTTGTTA TGGACCACTA CCTAGTACAA ATTCTTGTGA AAATAACAAA

Orangutan CGACTTGTTA TGGACCATTA CTTAGTACAA ATTCTTATGA AAATAATAAA

Mouse TGACCTGTTA CGGACCACTA CCCACTACAG AGTCTTCTGA AAATAACAGA

Guinea_Pig CAACTTGTTA TGGACCACTA CCTAGTACAG AGTGTTATGA AAATAATAAA

Rabbit CAACTTGTTA TGGACCACTA CCTAGTACAA ATTCTTGTGA AAGTAATAAA

Cow CGACTCGTTA CGGATCACTA CCTAGTACAA ATTCTTACGA AAGTAATAAA

Dog CGACTTGTTA TGGATCACTA CCTAGTACAA ATTCTTATGA AAATAATAAA

Horse CGACTTGTTA TGGATCACTA CCTAGTACAA ATTCTTATGA AAATAATGAA

Pig CGACTTGTTA TGGATCACTA CCTAGTACAA ATTCTTATGA AAGTGATAAA

Elephant CGGCTTGTTA TGGACCACTG CTTAGTACAA ATTCTTGTGA AAATAACAAA

Opossum CATCATGGTA TGGACCACTA ATTAGTACAG ATTCTCATGA AAATAATAAA

Platypus CAGGCTGGTA CGGGCCACTC CCTGGAGCC- ---------- ----------

Chicken AATCTTTGTA TGGGCCACTA CCTGCTACGG TCTCTGGTGA AGCTAATAAA

Zebra_Finch AATCACTGTA TGGGCCACTA CCTGCTGCAG TCCCTGGTGA AAGTAATAAA

Zebrafish TCTCCTTGTA TGGATACTCT AATGGTGAAG AGAAAACTGC AGGAAATCCC

Fugu TGTCCCTGTA TGGCTTTCGG CCTACTGATG ACGCTCTGAG GCCGCTCAGC

Frog GATCTGTGTA C--------- CCAGATTCAC AAATACTAGA AACTCATAAA

Human ACAGATGTTT CCGCAGCTGA CATCGTTCTT AGTAAAACAG CAGAAACAGA

Chimpanzee ACAGATGTTT CCGCAGCTGA CATCGTTCTT AGTAAAACAG CAGAAACAGA

Gorilla ACAGATGTTT CCGCAGCTGA CATCGTTCTT AGTAAAACAG CAGAAACAGA

Marmoset ACAGATGTTT CCGCAGCTGA CATCGTTCTT AGTAAAACAG CAGAAACAGA

Orangutan ACAGATGTTT CTGCAGCTGA CATCGTTCTT AGTAAAACAG CAGAAACAGA

Mouse GTAGATATTT CCTCAGCCAA CATGGTTGTT AGTCAAACAG CCGAAACAGA

Guinea_Pig ACAGATGTTT CCTCAATTGA CATAGTTGTT AGTAAAACAG CAGAAACAGA

Rabbit ACAGATGTTT CCTTAGCTGA CATGGTTGTT ACTAAAACAG CAGAAACAGA

Cow ACAGATGTTT CCTCAGCTGA CATGGTTGTT AGTAAAACAA CAGAAACAGA

Dog ACAGATGTTT CCTCAGCTGA CATGGTTGTT AGTAATACAA CAGAAACAGA

Horse ACAGATGTTT CTTCAGCTGA CACGGTTGTT AGTAAAACAG CAGAAACAGA

Pig ACAGATGTTT CCTCAGCTGA CACGGTTGTT AATAAAGCAA CAGAAACAGA

Elephant ACAGATGTTT CCTCAGCTGG CACGGTTGTT AATAGAACAG CACAAGCAGA

Opossum GCAGATGTTT CCTCAGTTGA CATGACTGTT AACAGAACAG TACAAACAGA

Platypus ---------- ---------- ---------- ---------- ----------

Chicken ACAGATGTTA CCTCAGAAGA CATGTTTGTT CACAAAGCAG GACAAACAGA

Zebra_Finch ACAGATGTTA CCTCAGAAGA CATGTTTGTC CATAGAACAG ACCAAACAGA

Zebrafish TCTGATGTTA CTTCATTGGA T---GAGCCT AAACGGACTT CCCCAGATAT

Fugu TCCGAGACAT CGTGCGTACC CTCTGAAGCG GCGTGGCCCG AGGAGGAGGC

Frog GATGCAATGC CTGAAGACAC TGTAACTAAT AATACAGATA ATTCCAATAC

Human TGTGCTTTTT AATAAAGTGG AATCATCTGG AAAGAATTAT TCAAATGTTG

Chimpanzee TGTGCTTTTT AATAAAGTGG AATCATCTGG AAAGAATTAT TCAAATGTTG

Gorilla TGTGCTTTTT AATAAAGTGG AATCATCTGG AAAGAATTAT TCAAATGTTG

Marmoset TATGCTTTTC ACTAAAATGG AATCATCTGG AAAGAATTAT CCAAATGTTG

Orangutan TGTGCTTTTT AATAAAATGG AATCATCTGG AAAGAATTAT TCAAATGTTG

Mouse TGTGCTTTTT AATAAAATGG AATCATTTGG AAATAATTAT CCAAATGCTG

Guinea_Pig TGTGCTCTTT AATAAAATGG AATCATCTGG AAATAATTAC CCAAATGTTG

Rabbit TGTGCTTTTT AATAAAATGG AATCATCTGG AAATAATCAT TCAAATGTTG

Cow TGTGCTTCTT ACTAAGATGG AAACATCTGG AAATAATTAT CCAAATGTTG

Dog TGTGCTTTTT AATAAGATGG AATCATCTGG AAATAATTAT CCAAATGTTG

Horse TGTGCTTTTT AATAAGATGG AATCATCTGG AAATAATTAT CCAAATGTTG

Pig TGTGCTTTTT ------ATGG AAACATGTGG AAATAATTAT CCAAATGTTG

Elephant TGCACTTTTT AATGAAACAG AATCATCTGG AAATAATTAT CCAAATGTTG

Opossum TGTGGATGTT AATGAAATGG AATCATCTGG AAATAGTTAT TCAGATGTGG

Platypus ---------- ---------- ---------- ---------- ----------

Chicken TGTGGTTGTT AATGAAAAGG AACCATCTGG AAACAATGAT CTACATGCTC

Zebra_Finch TGTGGCTGTT AATGAAATGG GACCATCTGG AAATGATGAG CTACATGCTC

Zebrafish TAGTTTAAAG CACCCAGAAC TGCCCGAGGA TTCTTCCTCG AATGCAGAAA

Fugu CAGCCTCGCC CCCACAGACC TCTTTGGTGA CGCTGATGCA ---GCAGTAA

Frog ACACCTTGTT GGAAACAAAA TCACAAGGAA TGATGGGGAA TTATTTAGTC

Human ATACTTCAGT CATTCCATTC CAAAATGATA TGCATAATGA TGAATCTGGA

Chimpanzee ATACTTCAAT CATTCCATTC CAAAATGATA TGCATAATGA TGAATCTGGA

Gorilla ATACTTCAAT CATTCCATTC CAAAATGATA TGCATAATGA TGAATCTGGA

Marmoset ATACTTCAGT CATTGTTTTC CAAAATGATG TGCATAATGA TGAATCTGTA

Orangutan ATACTTCAAT CATTCCATTC CAAAATGATA TGCATAATGA TGAATCTGGA

Mouse ATACTTCGGC TATTCACTTC CAA------- -----AAGGA TGAATCTAGA

Guinea_Pig ATACTTCAGT CATTCCTTTT CCAAGTGATG TACATAATGA TGAATCAAGA

Rabbit ATACTGCAAC CGTTTCTTTT CAAAATGATG TATATAATGA TGAGTCTGGT

Cow ATACTTCAGC CATTCCTTTC CAAAATGATG TGCGTAATGA TACATCTGGG

Dog ATACTTCAGC CATTCCTTTC CAAAATGATG TGTGTAATGA TAAATCTGGG

Horse ATACTTTAGC CATTCCTTTT CAAAATGACG TGCATAATGA TGAATCTGTA

Pig ATACTTCAGC CATTCTTTTC CAAAATGATG TGCATGATGA TAAATCTGGG

Elephant ATACTTCAGC CATTCCTTCC CAAAATGAAG TACAGAATGA TCAATCTGAA

Opossum ATATTTCAAC CTTTTCATTC CAACATGACA TGCAGAATTG TGAATCAGGA

Platypus ---------- ---------- ---------- ---------- ----------

Chicken ATACTTCATT CCTTTCATTC AACAGTGATG TGCAAAGTTA TCAAGCAGGA

Zebra_Finch ATACTTCATT CCTTTCACTC AGCAGTGATG TGCAAAACTG TCAAGCAGGA

Zebrafish AACAGGACGG TGAGCTCTCC TTGAGCAACA CCGCAAACAC CAGCTCTTCT

Fugu GGGCTGACGC TCCACCGGAC AGCCAGACCT CAAACGGCAG CTCGTCATCC

Frog CATGCACTGT TACAAACAGT ATTCAAGAAG AAAAGCAATC TGAGAACTCT

Human AAAAACACTG ATGATTGTTT AAATCACCAG ATAAGTATTG GTGACTTTGG

Chimpanzee AAAAACACTG ATGATTGTTT AAATCACCAG ATAAGTATTG GTGACTTTGG

Gorilla AAAAACACTG ATGATTGTTT AAATCACCAG ATAAGTATTG GTGACTTTGG

Marmoset AAAAACACTG ATGATTGTTT AAATCACCAG ATAAGTGTTG GTGACTTTGG

Orangutan AAAAACAATG ATGATTGTTT AAATCACCAG ATAAGTATTG GTGACTTTGG

Mouse AAAAACATTG ATCATCATTT AAATGAACAG ATAAATATAG GCAATTATTT

Guinea_Pig GAAAACACTA ATAAT---TT AAATCAGCAG ATAAGTATTG ATGAACATTG

Rabbit GAAGGAACTA ATCTTGGTTT AAATCAGCAG ACAAGTGTTG GTGTCCATTG

Cow GAAAACACTA ATTATTGTTT AAATCATCAG GTACATATTG GTGACCATTA

Dog AAAAACACTA ATTATTGTTT AAATAATCAG ATAAATGTTG ATGACCATTG

Horse AAAAACACTA ATTATTGTTC TGATCCTCAG CTACATGTTG ATGACCATTA

Pig AACAACACTG ATTTTTGTGT AGATCCTCAG ATACATGTTG CTGACCATTT

Elephant AAAAACACTA GTAATTGTTT AAATCATCAG TTATGTGTTG GTGATCATTG

Opossum AATAACACTG AGGTCTGCTT AAAAAAACAG ATAATTTGTC ATGATAATCC

Platypus ---------- ---------- ---------- ---------- ----------

Chicken AAAAACACAG AGATCTGTTT AAACCATCAG ATATTCTCTG GTGATACTGT

Zebra_Finch AAAAACACAG AGATCTGCTT AAATCATCAG ACATTTAGTG GTGATAATGT

Zebrafish TCGATTTCGG AGGATTGGGT GATCAACAGA AGTGATTTGG ACAGT---AT

Fugu TCTCTAGCAG AGGACTGGAT CGTTAACCAG ATGCCAGCTG GTTCTGAGTC

Frog TTAGAGTATC AAAAATTGGA AAGGGATAAA ATTCCTTATT CTGATAATGG

Human TTATGGTCAT TGTAGTAGTG AAATTTCTAA CATTGATAAA AACACTAAGA

Chimpanzee TTATGGTCAT TGTAGTAGTG AAATTTCTAA CATTGATAAA AACACTAAGA

Gorilla TTATGGTCAT TGTAGTAGTG AAGTTTCTAA CATTGATAAA AACACTAAGA

Marmoset TTATGGTCAT TGTAGTAGTG AAAATTCTGA CATTGATAAA AACACTGAGA

Orangutan TTATGGTCAT TGTAGTAGTG AAATTTCTAA CGTTGATAAA AACACTAAGA

Mouse TGATGGTCAT TTTAGTGGTG ACATTAAT-- ---------- ------AAGG

Guinea_Pig TGATGATCAT TTTAATAGTG AAAACTGTAG CATTAATAAG GACACCAGAA

Rabbit TGATAGTCAT TTTAGTAGTG AAAATGCTAG CATTGGTAAG AACACTAGAA

Cow TGATGATTAT TTTAATAGTG AAAATTCTAG CATTGATAAG AATGATGGAA

Dog TGATGATCAT TTTAATAGTG AAAATTCTAA CATCGATAAG AACACTAGAA

Horse TGATGATCAT TTTAGTAGTG AAGATTCTAG CATTGTTAAG AACACTAGAA

Pig TGGTGATCAT TTTAATAGTG AAAATTCTAG CATTGATAAG AACACTTCT-

Elephant TGATCGTCAT TTCAGCAGTG AAAATTCTAG TATTGATAAG GACAGTAGAA

Opossum TCATGGT--- TTTGATGGTG ACATTTCTAA TACTGATAAG ATAAAGCAAA

Platypus ---------- ---------- ---------- ---------- ----------

Chicken ACATAGTTGC CTAGAAGAAA AAGAGGCTTC TAAGAATGAT GCCTTTCAAG

Zebra_Finch ACACAGTTGC TTGGACAAAA GAGAGGTTTC TAAGAGTGAT GCCTTTCCAG

Zebrafish TAATTGCTCA GTACCTGTTG ACGATGTGGT AATGAAC--- ----------

Fugu TAACGCGTCC CTCCACGACC AAAACGACCT TCTGTCT--- ----------

Frog TGAGGTTGCC TACAGTGTGT CTGAAAGCAC TGATGGTTTT CTATTAAATA

Human ATGCATTTCA GGACATTTCA ATGAGTAATG TATCATGGGA GAACTCTCAG

Chimpanzee ATGCATTTCA GGACATTTCA ATGAGTAATG TATCATGGGA GAACTCTCAG

Gorilla ATGCATTTCA GGACATTTCA ATGAGTAATA TATCATGGGA GAACACTCAG

Marmoset ATGCTTTTCA AGACATTGCA ATGAGTAATT TATCATGGGA GAACACTCAG

Orangutan ATGCATTTCA AGACATTTCA ATGAGTAATG TATCATGGGA GAACACTCAG

Mouse ACACATTTCA AAATATTCCT ATGAATAATT TATCATCAGA GACTAATCAG

Guinea_Pig ATACACACCA AAAAATCTCA ATGAATAACT TGCCATCAGA GGATGGCCAG

Rabbit ATACATCCCA AGAAATCCCA GTGAATAATT TATCGTGGGA AGATGCCCAG

Cow ATGCATTTAA AGAAATCCCA GAGAATAAGT CATCGTGTGA GGACACCCAA

Dog GTATATTTCA GGAGATCCCA ATGAATAATT TATCATGTGA GGATGCCCAA

Horse ATTTATTTCA GGAGATTTCA ATGAATAATT CATCATGTGA GGACGCCCAG

Pig --------CA AGGAATCCCA ATGAATAATT TACCATTGAA AAACACCCAA

Elephant ATACATTTCC AGAGATCCCA GTGAATAGTT TATCATGTGA AGATGCCCAA

Opossum ATACATTTCA AGACATCTTC ATAAATAATT TTTCATGTAA GAATGACGCA

Platypus ---------- ---------- ---------- ---------- ----------

Chicken AAGGTTCCTT ACGGTTATTG TGTGAGGAGG AGCAGGGGCA AAATACAACT

Zebra_Finch ACAGTTCCTC AAATTTATTG CGTGAGGAGG AACAGGGACA GAATATGGCT

Zebrafish ---------- ---------- ---------- ---------- ----------

Fugu ---------- ---------- ---------- ---------- ----------

Frog ATGAATCTTC CACGCTACAG GGTAAAGCCA GTGCT----- -------GAC

Human ACGGAATATA GTAAAACTTG TTTTATAAGT TCCGTTAAGC ACACCCAGTC

Chimpanzee ACGGAATATA GTAAAACTTG TTTTATAAGT TCCGTTAAGC ACACCCAGTC

Gorilla ACGGAATATA GTAAAACTTG TTTTATAAGT TCTGTTAAGC ACACCCAGTC

Marmoset ATGGAATATA GTAAAACTTG TTTTGTAGAT TCCATTAAGC ACATCCAGTC

Orangutan ACGGAATATA GTAAAACTTG TTTTGTAGGT TCCATTAAGC ACACCCAGTC

Mouse AGTAATCACA GTGAAACTCA TTCTGTAGGT TCTATGGAGC ACATCCAAGG

Guinea_Pig AGTGCAGATA GTGACACTTG TTTTGTGAGT TCTGTTAAGC ATGCCCAACT

Rabbit AGTGGATATG GTGAAACTTG TCTTGTGGGT TCTATAAAGC ACGTCCAATC

Cow GAGAAGAATG GTGAAACTTG GTCTGTGGAT TCTGTTGGGC AAGCCCAATC

Dog AAGGAATGTA GTGAAACTTG TTTTGTAGTT TCTGTTAAGC ATACCCAATC

Horse AAGGAATATA GTGAAACTTG TTATGTAGAT TCTGTTAAGC ATGCCCAATC

Pig AAGGAATATA GTGAAACTCG TCTGGTGGAT TCTGTTGAGC ATCCC---AA

Elephant AATGAATGTC ATGAAACTTG TTTTGTAGGT TCTATTAAGC ATATCCAATC

Opossum AGCCAACATA ATGAAACTTT TAATCTAAAT GCTCTAAAGC ATGTTCAGTC

Platypus ---------- ---------- ---------- ---------- ----------

Chicken GGAATTCCAA GTAACAGTGC TCCTGTAGAT CCTGGGTCTA AAGAAGAAGG

Zebra_Finch GATATTCAAA GTAATAGTGT TGCTGTGGAT CCTGAGTCTA AAAAAGCCAA

Zebrafish ---------T CTACAGCTGA TCTGAACTCC AACTCTCCAA AGGCGTCTGA

Fugu ---------G TAGAAACAAA CGTAATAAAC AAGCTACCTG AGATGCTGGA

Frog TTTGGATTAC AGTTTGCTGT TAGAAACAGT AATTTAATGG ATGTTCAATC

Human AGAAAATGGC AATAAAGACC ATATAGATGA GAGTGGGGAA AATGAGGAAG

Chimpanzee AGAAAATGGC AATAAAGACC ATATAGATGA GAGTGGGGAA AATGAGGAAG

Gorilla AGAAAATGGC AATAAAGACC ATATAGATGA GAGTGGGGAA AATGAGGAAG

Marmoset AGAAAATGGC AATAAAGACC ATATAGATGA GAGTGGGGAA AATGAGGAAG

Orangutan AGAAAATGGC AATAAAGACC ATATAGATGA GAGTGGGGAA AATGAGGAAG

Mouse AGAAAATGGC AGGAATGGT- --ACAGGAGG AAGTGGGGGA GATGGGAGAG

Guinea_Pig AGGAAATGAT GGTCGAGGCC ATATTGATGA AAAG---AGG GAAAATGAAG

Rabbit AGAAAATGGC AGTAAAATCT ATACAAATGA AAGTGGGAGA CTTGAGGAGG

Cow AGAAAATGGC AATAAAGGCC ACAGAGATGA GAGTGGAGAA AATGAAGAAG

Dog AGAAAATGGC AATAAAAGCA ATAGAGATGA GAATAGGAAA AATGAGGAAG

Horse ACAAAATGGT AGTAAAGGCC ATATAAATGA GAGTGTGAAA AATGAAGAAG

Pig TAAAAATGCC AATAAAGATC ATATAGAGGA A--------- AATGAGGAAG

Elephant GGAAAATGGC GATAAAGGCC ATAGAAATGA GAGTGGGGAA AACAAGGAAG

Opossum TATAAATGGC AATGGAGATG GTATTAATGA GGTGGAAAAA AATGTGGCAG

Platypus ---------- ---------- ---------- ---------- ----------

Chicken TGGATGCCTT TTGAGTTCTG AT-------- -------AAT GCTAAAGAAG

Zebra_Finch TGAGCACCTT TTGAGTTCTG ATATTGACAA AATAGATAAA AATGAGGAAA

Zebrafish GAGTGACAGC GGTCCAGAG- ---------- ---------- ----------

Fugu GGACAACGCC TCCACAGACG AG-------- ---------- ----------

Frog TAACAACACT TTGGATTCTG TACAGGTTTT AGAAAAGGAT ACATGGAAAA

Human AAGCAGGTCT TGAAAACTCT TCGGAAATTT CTGCAGATGA GTGGAGCAGG

Chimpanzee AAGCAGGTCT TGAAAACTCT TCAGAAATTT CTGCAGATGA GTGGAGCAGG

Gorilla AAGCAGGTCT TGAAAACTCT TCGGAAATTT CTGCAGATGA GTGGAGCAGG

Marmoset AAGCAGTCCT TGAAAACTCT TCAGAAATTT CTGCAGATGA TTGGAGCAGG

Orangutan AAGCAGGTCT TGAAAACTCT TTGGAAATTT CTGCAGATGA GTGGAGCAGG

Mouse AAACACTTCC TGAGAAAGCT CTGGAAATCT GTGCAGACGA TTGGAGCAAA

Guinea_Pig CAGAAGCTCC TGAGAAGTCT CTGGAAATCT CTGCAGATGA CTGGAGCAAG

Rabbit AAGCAGTTCC TGAGAAGTCT TTGGAAATTT GTGCAGATGA CTGGAGCAAG

Cow CAGCAGTTCC TGAGAAGTCT GCGGAAATTT GTGTAGATGA CTGGAGCAAA

Dog GAGCAGTTCC TGAGAAGCCT TTGGAAATCT CTGCAGATGA CTGGAGCAAG

Horse CAGCAGTTCC TGAGAAGTCT TTGGAAATTT GTGCAGATGA CTGGAGCAAG

Pig AAGCAGTCCT TGAGAAGTCT TTGGAAATTT GTGCAGATGA CTGGAGTAAG

Elephant CAGCAGTCCT TGAGACATCT TTAGAAATTT GTGCAGATGA CTGGAGCAAG

Opossum CAACACTATC TGAAATATCT TCTGAAATCT GTGCAGATGA CTGGAGTAAG

Platypus ---------- ---------- ---------- ---------- ----------

Chicken CCTTAGTCCC TGAAGACTTA CCTGAAATCT CTGCTGATAA TTGGAGTATG

Zebra_Finch TCTTAGTCCC TAAGGACTTA CCTGAAATCT CTGCTGATAG TTGGAGTATG

Zebrafish ---------- ---------- AGCCAGATAT CTGCCGAGAA CTGGAGTGCG

Fugu ---------- ----CGCAGA GGCCAGTTCT CAGCTGAGGA CTGGAGCCGT

Frog CAAAAACACT GACTAAGGTC CCTGATATTG GTGATGATAT TTGGAGTAAA

Human GGAAATATAC TTAAAAATTC AGTGGGAGAG AATATTGAAC CTGTGAAAAT

Chimpanzee GGAAATATAC TTAAAAATTC AGTGGGAGAG AATATTGAAC CTGTGAAAAT

Gorilla GGAAATACAC TTAAAAATTC AGTGGGAGAG AACATTGAAC CTGTGAAAAT

Marmoset GGACATATAC TTAAAAATTC AGTGGGAGAG AATATTGAAC CTGTGAAAAT

Orangutan GGAAATATAC TTAAAAATTC AGTGGGAGAG AATATTGAAC CTGTGAAAAT

Mouse GGAAATTTGT GT---AATTC AATGGGAGAG AATATCGAAC CTGTGAAAAT

Guinea_Pig GGAAATGCTC TGAAGAATTC AATGGGAGAG AATATTGAAC CTGTGAAAAT

Rabbit GGAAATGCAT TTAAGAATTC GATGGGAGAG AATATTGAAC CTGTGAAAAT

Cow GGAAATATAC TT---AATTC TAGAGGAGAA AACATTGAAC CTGTGAAAAT

Dog GGAAATACAC TTAAAAATTC AATGGGAGAA AACATTGAAC CTGTGAAAAT

Horse GGAAATGTAC TTAAAAATTC AATGGGAGAA AACATTGAAC CTGTGAAAAT

Pig GGAAATGTAC TTAAAAATTC AAGAGGAGAA AACATTGAAC CTGTGAAAAT

Elephant GGAAATGTAC TTAAAAATTC AATGGGAACA AACATTGAAC CTGTGAAAAT

Opossum GGAAATGCAC TTAAAAATTC AGTAGGAAAG AACATTGAAC CTGTGGAAAT

Platypus ---------- ---------- ---------- ---------- ----------

Chicken GGAAATGCAT TTAAAAACTC CTCAGGAGAC AACCTGGAAC CTGTGAAGGT

Zebra_Finch GGAAGTGCAT TTAAAGACAT TCTGGGAGAC AAGCTGGAAC CTGTTAAGAT

Zebrafish GGCAGGGCCT TCTCCAATCA AATCACTGGG GAACTAGAAC CTGTCAAACT

Fugu GGCACAGCTT TGACTGACCC CGTCTCAGGA AGGCTTCAGC CCGTCAGTAT

Frog GGAAGTACTT TTAAAGACTC GCTTGGTGAT AACCTAGAAC CTATAAAGAT

Human TTTAGTGCCT GAA---AAAA GTTTACCATG TAAAGTAAGT AATAATAATT

Chimpanzee TTTAGTGCCT GAA---AAAA GTTTACCATG TAAAGTAAGT AATAATAATT

Gorilla TTTAGTGCCT GAA---AAAA GTTTACCATG TAAAGTAAGT AATAATAATT

Marmoset TTTAGTGCCA CAA---AAAA GTTTACCATG TAAAGAAAGT AATAATAATC

Orangutan TTTAGTGCCT GAA---AAAA GTTTACCATG TAAAGTAAGT AATAATAATT

Mouse TTTAGTGCCA CAA---AAGA GCTTATCATG TAAAGTGACC ---AGTAGTC

Guinea_Pig TTTAGTGCCC CAG---GAAA GTTTAGCACA TAAAGTAAGC AAGAGTAATC

Rabbit TTTAGTGCCT CAA---GAAA GTTCAGCATG TAAAGCAAGT GATAATAATC

Cow TTTAGTGCCT CAA---AAAA GTTTAGTGAG TAATGTAAAT AATAATAATA

Dog TTTAGTGCCT CAA---GAAA GTTCAGCGTG TAAACAA--- AATAATGCTT

Horse TTTAGTGCCT CAA---GAAA GTTCTGCATG TAAAGTGAGT AATAATAATC

Pig TTTAATGCCT CAA---GAAA GTTTAGCATG TAAAGTAAGC AATAATAATA

Elephant TTTAGTGCCA CAG---GAAA GTTTAGCATG TAGTAAT--- ---------A

Opossum TTTTGTGCCA CAAGCAAAAA GTTTAACATC TGACCTAAAT AACTGTAATC

Platypus ---------- ---------- ---------- ---------- ----------

Chicken TTTAATTCCT GACGCTGGAG GAGCAATTAA TAAGCAAAGT CGTAGCGATG

Zebra_Finch CTTGATTCCT GAAGTTGGAG GGACAACTAA TAAGCAAAAT TACACTGGTG

Zebrafish GCACATCCCA AAG------- ---------- ---------- ATTGAAGACC

Fugu CCACCAACCA CCA------- ---------- ---------- ACCAAC----

Frog ATTAAATTTA TCTGCAGAGA CTCCTTTTGT CAAAAACACT ----------

Human ATCCAATCCC TGAACAAATG AATCTTAATG AAGATTCATG TAACAAAAAA

Chimpanzee ATCCAATCCC TGAACAAATG AATCTTAATG AAGATTCATG TAACAAAAAA

Gorilla ATCCAATCCC TGAACAAATG AATCTTAATG AAGATTCATG TAACAAAAAA

Marmoset ATCCAAGCCC TGAACAAAAG AATCTCAATG AAGGTCCATG TAACAAAAAA

Orangutan ATCCAAGCCC TGAACAAACG AATCTTAATG AAGATCCATG TAACAAAAAA

Mouse ACCCACGCCT TGAACCAGAG ACTCTCAGTG ATGGTCCTTG TAGCAGAACA

Guinea_Pig ATTCCAGCCT TGAACAAGAG AATCTCAGTG ATGGCCCTTG TAACAGAAAA

Rabbit ATCCAAGCCC TGAACAAACG AAGCTCAGTG AAGGTCCTTC CAACAAAAAA

Cow ATACAAGTCC TGAACAAAAG AATCTCTGTG AAAGTTCCTG TGACAAAAAT

Dog ATCCAAGTCC TGAACTAAAG AATCTCAGTG AAAGTTCCTG TAGCAAAAAA

Horse TTCCAAGCTC TGAACAAAAG AATCTCAGTG AAGGTGCCTA TAACAAAAAA

Pig ATTCAAGTCC TGAGCAAAAG AATCTCAGTG AAAGTTTCTG TGACAAAAAA

Elephant ATCCCAGCCC TGAATCAAAG AATCTCAGTG AAGGTACCTG TAACAAAAAG

Opossum AACAAAATCC TGAACAGAAG AGTCCCAGTA AAAGCCCCAG TAAAAAGAAA

Platypus ---------- ---------- ---------- ---------- ----------

Chicken AACAAAGCCA CGATCCACAA ------ATCC CAGATCCAAG TATAAGGAAA

Zebra_Finch AAAAAAGCAG TGATCCACAA ------AGCT CAAATCAAAG CATAAAGAAA

Zebrafish TTGGAGGAAC CGAGGCCAAG AGCGTGAAGT CTAGCCCATC TAAAAAGCCC

Fugu -----GTCTC TGCTCCAGAC CAGGCTCTGA GCAGCCCCGA CAAAAAGACG

Frog -----GAAGA TAGTAATATT CACAATTCAA AGAATGAAAA TACCAAACCT

Human TCAAATGTAA TAGATAATAA ATCTGGAAAA GTTACAGCTT ATGATTTACT

Chimpanzee TCAAATGTAA TAGATAATAA ATCTGGAAAA GTTACAGCTT ATGATTTACT

Gorilla TCAAATGTAA TAGATAATAA ATCTGGAAAA GTTACAGCTT ATGATTTACT

Marmoset TCAAATGTAA TACATAATAA ATCTGGACAA GTTACAGCTT ATGATTTACT

Orangutan TCAAATGTAA TAGATAATAA ATCTGGAAAA GTTACAGCTT ATGATTTACT

Mouse TCAGATGTAA TCCATAACAG ATCTGGACGG CTTACAGCCT ATGACTTAAT

Guinea_Pig TCAAATGTGA TAGATAACAA GTCTGGACAG CTCACAGCCT ATGATTTAAT

Rabbit TCAAATGTCA TAGATAACAA AACTGGACAG CTTACAGCTT ATGATTTAAT

Cow TCAAATGTGG TAGATAACAA ATCTGGACAG CTTACAGCTT ATGATTTAAT

Dog TCAAATGTGG TAGATAACAA ATCTGGACAG CTTACAGCAT ATGATTTAAT

Horse TCAAATGTGG TAGATAACAA ATCTGGACAG CTTACAGCTT ATGATTTAAT

Pig TCAAATGTGA TAGATAACAA ATCTGGACAG CTTACAGCTT ATGATTTAAT

Elephant TCAAATGTGG TTGATAACAA ATCTGGACAG CTTACAGCTT ATGATTTAAT

Opossum TCAAATGTGA TAGATGACAA ATGTGGGCAA CTTACAGCCT ATGATTTAAT

Platypus ---------- ---------- ---------- ---------- ----------

Chicken ACAAATGTAA TAGATGAGAA AACAGGACAC ATAACGGCTT ATGACTTAAT

Zebra_Finch AAAAATGTGA TAAGTGAAAA ATTTGGACAT GTGACAGCTT ATGACTTAAT

Zebrafish TCTAACGTGA TTGTGGAGAA AATGGCCAAG CTGACAGCCT ATGAGCTTAT

Fugu CTGAACGCCA TAACGGAGAA ACGCGCTACG CTGACGGCTT ATGACCTGAT

Frog TTAAATGTCA TTAGTGAAAA GTCTGGATTT GTCACTGCCT ATGACTTAAT

Human TAGCAATCGA GTAATCAAGA AACCCATGTC AGCAAGTGCT CTTTTTGTTC

Chimpanzee TAGCAATCGA GTAATCAAGA AACCCATGTC AGCAAGTGCT CTTTTTGTTC

Gorilla TAGCAATCGA GTAATCAAGA AACCCATGTC AGCAAGTGCT CTTTTTGTTC

Marmoset TAGCAATCGA GCAATCAAGA AACCCATGTC AGCAAGTGCT CTTTTTGTTC

Orangutan TAGCAATCGA GTAGTCAAGA AACCCATGTC AGCAAGTGCT CTTTTTGTTC

Mouse TAGCAGTCGA GCAGTCAAGA AGCCCATGTC AGCCAGTGCC CTGTTCATTC

Guinea_Pig TAGCAATCGA GTAATCAAGA AACCCATGTC TGCAAGTGCC CTTTTTACTC

Rabbit TAGCAGTCGA GTAATCAGGA AGCCCATGTC AGCAAGTGCC CTTTTTGTTC

Cow TAGCAATCGA GTAATCAAGA AACCCATGTC AGCAAGCGCC CTTTTTGTTC

Dog CAGCAGTCGA GTAGTCAAGA AACCCATGTC AGCAAGCGCC CTTTTTGTTC

Horse TAGCAATCGA GTAATCAAGA AACCCATGTC AGCAAGCGCC CTTTTTGTTC

Pig TAGCAATCGA GTAATCAAGA AACCCATGTC AGCAAGAGCC CTTTTTGTTC

Elephant TAGCAATCGT GTAATCAAGA AACCCATGTC AGCAAGTGCT CTTTTTATTC

Opossum TAGCAGTCGA GTAATTAAGA AGCCCATTTC AGCAAGAGAC CTTTTTATTC

Platypus ---------- ---------- ---------- ---------- ----------

Chicken TAGTGGTCGA ATAATAAGGA AATCAAAGTC TGCATTTGAA CACTTCATCC

Zebra_Finch TAATAGCAAA ATAATAAAGA AACCAAAGTC TGCATTTGAA TTTTTCACAC

Zebrafish CAGCAACCGC TCCGTGCGTC AGCCCTCGTC TGCCTTTTCG CTGTTTGAGC

Fugu CAGCAGCCGG GCCATGAGGG CACCTCTGTC CCCCGCCGCT CTGTTCGAGA

Frog AAGTGAAAAA GCAATCAAGA AACCATTGTC TGCAGTGGGT TATTTCATAC

Human AAGATCATCG TCCTCAGTTT CTCATAGAAA ATCCTAAGAC TAGTTTAGAG

Chimpanzee AAGATCATCG TCCTCAGTTT CTCATAGAAA ATCCTAAGAC TAGTTTAGAG

Gorilla AAGATCATCG TCCTCAGTTT CTCATAGAAA ATCCTAAGAC TAGTTTAGAG

Marmoset AAGATCGTCG TGCTCAGTTT CTCATAGAAA ATCCTAAGAC TAGTTTAGAG

Orangutan AAGATCATCG TCCTCAGTTT CTCATAGAAA ATCCTAAGAC TAGTTTAGAG

Mouse AAGACCACCG CACTCAGTTC CTCACAGAGA ATCCCAAGAC TGGTTTGGAG

Guinea_Pig AAGAGCATCG TGCTCAGTTT CTCATAGAAA ATCCTAAGAC TAGTTTAGAG

Rabbit AAGATCACCG TCCTCAGTTT CTCACGGAAA ACCCTAAGAC CAGTATGGAG

Cow AAGATCATCG TGCTCAGTTT CTCACAGAAA ATTCCAAGAC CAGTTTGGAA

Dog AAGATCATCG TGCTCAGTTT CTCACAGAAA ATTCTAAGAC CAGTTTGGAG

Horse AAGATCATCG TGCTCAGTTT CTCACAGAAA ATTCTAAGAC CGGTTTGGAG

Pig AAGATCATCG TGCTCAGTTT CTCACAGAAA ATTCCAAGAC TAGTTTGGAA

Elephant AAGATTATCG TGCTCGGTTT CTCACAGAAA ATTCTAAGAC TAATTTGAAG

Opossum AAGATCAGCG TTCAAAATTT CTCAGTGAAA ATTCAAAGAC CCATCTAGAG

Platypus ---------- ---------- ---------- ---------- ----------

Chicken AAGAGTATCG TTCAAAATTT GTTTCTGAAA ATCCCAGGTG TAGCATGAAG

Zebra_Finch ATGAGTGTCG TCCAAAATTG ATAGATGATA ATCCAAAGAC CAGCATGAAT

Zebrafish AAGACACCAG ATCGCAGGTC CTCCAGGAGA ACCCAAAAGC CAGTCCTCAG

Fugu AGGAGGCCAG GGCCGACGTC CTGAGGGAGA AACCCGCAGC CGGCCTGCAG

Frog AGGCAGAACG ATCCAAACTT ATTGATGATA ACCCAGTGGC TAGTGTTGAG

Human GATGCAACAC TACAAATTGA AGAACTGTGG AAGACATTGA GTGAAGAGGA

Chimpanzee GATGCAACAC TACAAATTGA AGAACTGTGG AAGACATTGA GTGAAGAGGA

Gorilla GATGCAACAC TACAAATTGA AGAACTGTGG AAGACATTGA GTGAAGAGGA

Marmoset GATGCAACAG TACAAATTGA AGAACTGTGG AAGACACTGA GTGAAGAGGA

Orangutan GATGCAACAC TACAAATTGA AGAACTGTGG AAGACATTGA GTGAAGAGGA

Mouse GATGCGACAG AACAAATCAA AGCACGATGG GAGACACTGA GTGAAGAGGA

Guinea_Pig GATGCAACTG TACAAATTGA AGAACTGTGG AAGACATTGA GTGAAGAGGA

Rabbit GAAGCAACAG TGCAGATTGA AGAACTGTGG AAGACATTGA GTGAAGAGGA

Cow GATGCAACAG TACAAATTGA AGAACTGTGG AAGACATTGA GTGAAGAGGA

Dog GATGCAACAG TACAAATTGA AGAACTGTGG AAGACATTGA GTGAAGAGGA

Horse GATGCAACAG TACAAATTGA AGAACTGTGG AAGACATTGA GTGAAGAGGA

Pig GATGCTACAG CACAAATTGA AGAACTGTGG AAGACATTGA GTGAAGAGGA

Elephant GATGCAGCAG TACAAATTGA AGAACTGTGG AAGACATTGA GTGAACAGGA

Opossum GATATGACAG TACAAGCTGA AGAACTGTGG AAGACATTGA ATGAAGAGGA

Platypus ---------- ---------- ---------- ---------- ----------

Chicken GACATCCTAT TGAAAATTGA AGAGCAGTGG AAGAATTTAA ATGAAGAAGA

Zebra_Finch GACATCCTGC TGACAATTGA AGAGCAGTGG AAGAATTTGA ATGAAGAGGA

Zebrafish GATGTCACAG CTGCTGCGAA AGAGAGATGG GAAAGCCTCG GGGAGGAAGA

Fugu GATATCAGCG CCGCTGTTCA CGAGAGGTGG AAAAATCTGA GGGAGGAAGA

Frog GAGATTTCTT CAAAAGGAAA GGATCTCTGG GGTAAACTCA GTCAAGATAA

Human AAAACTGAAA TATGAAGAGA AGGCTACTAA AGACTTGGAA CGATACAATA

Chimpanzee AAAACTGAAA TATGAAGAGA AGGCTACTAA AGACTTGGAA CGATACAATA

Gorilla AAAACTGAAA TATGAAGAGA AGGCTACTAA AGACTTGGAA CGATACAATA

Marmoset AAAACTGAAA TATGAAGAGA AGGCTACTAA AGACTTGGAA CGATACAATA

Orangutan AAAACTGAAA TATGAAGAGA AGGCTACTAA AGACTTGGAA CGATACAATA

Mouse AAAGCACAAA TACGAGGAAA AGGCTAAAAA AGACTTGGAA CGATACAATA

Guinea_Pig GAAACTCAAA TATGAAGAAA AGGCTACTAA AGATTTGGAA CGATACAATA

Rabbit AAGACTGAAA TATGAAGAGA AGGCTACAAA AGACTTGGAA CGATACAATA

Cow AAAACTGAAA TATGAAGAGA AAGCTGCTAA AGACTTGGAA AGATACAATA

Dog AAAACTGAAA TATGAAGAAA AGGCTACTAA AGATTTGGAA CGATACAATA

Horse AAAACTGAAA TATGAAGAGA AGGCTTCTAA AGACTTGGAG CGATATAATA

Pig AAAAGTAAAA TATGAAGAGA AGGCTACTAA AGACTTGGAA CGATACAATA

Elephant AAAACTGAAA TACGAAGAGA AGGCTGCTAA GGACTTGGAG CGATACAATA

Opossum AAGGATGAAA TATGAAGATA AAGCTGCTAA AGATCTAGAG CGATACAATA

Platypus ---------- ---------- ---------- ---------- ----------

Chicken AAAGCAGGAG TATGAACTAA AAGCTGCTAG AGACCTGGAG CGGTACAACA

Zebra_Finch AAAACAGAAT TACGAAATAA AAGCCACTAA GGACCAGGAA CGGTACAACA

Zebrafish CCGCAAAAAG TATGAAGGCA AGGCCGAAAA GTCTCTGAAC GCCTACAACC

Fugu TCGCAAAAAA TTTGGGATGG CCCAGACTTT GAGAATTAAA ACTTTGACAT

Frog GAAATCCAAG TATGAAGAAA AAGCAGCTAA TAATTTGGAG AGGTACAATC

Human GTCAAATGAA GAGAGCCATT GAACAGGAGT CACAAATGTC ACTAAAAGAT

Chimpanzee GTCAAATGAA GAGAGCCATT GAACAGGAGT CACAAATGTC ACTAAAAGAT

Gorilla GTCGAATGAA GAGAGCCATT GAACAGGAGT CACAAATGTC ATTAAAAGAT

Marmoset GTCAAATGAA GAGAGCCATT GAACGGGAGT CACAAATATC ATTAAAAGAT

Orangutan ATCAAATGAA GAGAGCCATT GAACAGGAGT CACAAATATC ATTGAAAGAT

Mouse ACCAAATGAA GAAAGCTATT GAACAGGAGA CACAAGTATC ATTGAAAGAC

Guinea_Pig ATCAAATGAA GAAAGCCATT GAACAAGAGA CACAAATACC ATTAAAAGAT

Rabbit ATCAAATGAA AAGAGCCCTT GAACAGGAGT CACAAATACC GTTAAAAGAT

Cow AGCAAATAAA GAGAGCCATC GAACAGGAGT CACAAGTATC ATTAAAAGAT

Dog GGCAAATCAA GAGAGCCATT GAACAGGAGT CACAAATATC ATTAAAAGAT

Horse AGCAAATCAA GAGAGCCATT GAACAGGAGT CACAAACACC AATAAAAGAT

Pig GGCAAATCAA GAAAGCCATT GAACAGGAGT CACAAATATC ATTAAAAGAT

Elephant GGCAAATGAA GAGAGCCACT GAACAGGAGT CACAAATATC ATTAAAAGAT

Opossum AGCAAATCAA GAAGGCTACT GAACAAGGGT TACAAAGAAC AACAAAAGAA

Platypus ---------- ---------- ---------- ---------- ----------

Chicken GAGAGGCTAA CAAAGCTATG CAGCAAGCAG CACGCAGACC TGTAAAGGAG

Zebra_Finch GAGAGGTCAA GAAAGCCAAT GCACAGCCAA TGCACCGACC AGCAAAGGAG

Zebrafish TTCAA----- ---------- AGGAAGAGGG CAGCGGAGGG AGGCGTCCAG

Fugu TGATGATGAT TTTAAAGTAT GAAGAGAAGG CCCAGAAGCA CCAGGCGCAG

Frog TGCAGATAGC TATGGCAACA GGAGAACATA TTCAAAAG-- -CCAAAAGAC

Human GGCAGAAAAA AGATAAAACC CACCAGCGCA TGGAATTTGG CCCAGAAGCA

Chimpanzee GGCAGAAAAA AGATAAAACC CACCAGCGCA TGGAATTTGG CCCAGAAGCA

Gorilla GGCAGAAAAA AGATAAAACC CACCAACGCA TGGAATTTGG CCCAGAAGCA

Marmoset GGCAGAAAAA AGATAAAACC CACCAGCACA TGGAATTTGG CCCAGAAGCA

Orangutan GGCAGAAAAA AGATAAAACC CACCAGCGCA TGGAATTTGG CTCAGAAGCA

Mouse GGCAGGAAAA AA---AGGGC CACCAGTTCG TGGGGCTGGA CACAGAAACA

Guinea_Pig GGCAGGAAAA AGATAAAATC CACCAATGTC TGGAGTTTGG CACAGAAGCA

Rabbit GGCAGAAAAA AGATAAAACA TACCAGTGCG TGGAATTTGG CACAGAAACA

Cow GGGAAAAAAA GGATGAAACC CACCAGTGTG TGGAATTTGG CACAGAAGCA

Dog GGCAGAAAAA AGATAAAGCC CACCAGTGCA TGGAATTTGG CACAGAAGCA

Horse GGCAGAAAAA AGATAAAACC CACCAGTGCA TGGAATTTGG CACAGAAGCA

Pig GGGAAAAAAA AGATAAAACC CACCAGCGCG TGGAATTTGG CACAGAGGCA

Elephant GGCGGGAAAA AGACAAAACC CATCAGTGTG TGGCATTTGG CCCAGAAGGA

Opossum AATGAAAAAA GATCAAAATT AACAACAATG CAGAATCTGG CACAAAAACA

Platypus ---------- ---------- ---------- ---------- ----------

Chicken AACGAAAGAC AG-------- ---------- ---------- -----AAGCC

Zebra_Finch GCAGAAAAAC AC-------- ---------- ---------- -----AAACC

Zebrafish TGCGGAGAGG GCAAGAAGCA GATGAGCGCC GAGTCTCCAT TGAACGGCGT

Fugu CACGAGCAGA GGACCAAACT GGCCTCTGCT GAAGGTCCCA CCGAGGGCCA

Frog ATAGAGAAAA GACATAAGCT CTCTTCTGGA CCTAGCCCTT CTCGGAAAGT

Human CAAGTTAAAA ACCTCATTAT CTAATCAACC AAAACTTGAT GAACTCCTTC

Chimpanzee CAAGTTAAAA ACCTCATTAT CTAATCAACC AAAACTTGAT GAACTCCTTC

Gorilla CAAGTTAAAA ACCTCATTAT CTAATCAACC AAAACTTGAT GAACTCCTTC

Marmoset CAAGTTGAAA ACCTCATTAT CTAATCAACC AAAACTTGAT GAACTCTTTC

Orangutan CAAGTTGAAA ACCTCATTAT CTAATCAACC AAAACTTGAT GAACTCCTTC

Mouse TAAGGTGAAA GACTCATCT- --AATCAGCC AAAACTTGAT GAGCTCTTTC

Guinea_Pig CAAGTTGAAA ACCTCATTAT CTAATCAGCC GAAACTCGAT GAGCTCTTTC

Rabbit CAAGTTGAAA GCTTCATTAT CTAATCAGCC AAAACTCGAT GAGCTCTTTC

Cow CAAGTTGAAA ACCTCACCAT CTAATCAGCC AAAACTTGAT GAGCTCTTTC

Dog CAAGTTGAAA ACTTCATTGT CTAATCAGCC AAAACTTGAT GAGCTCTTTC

Horse CAAATTAAAA ACTTCGTTA- --ATTCAGCC AAAACTTGAT GAGCTCTTTC

Pig CAAGTTGAAA ACTTCGTTAT CTAATCAGCC AAAACTTGAT GAGCTCTTTC

Elephant CAAGTTGAAA ACTTCATTAT CTCATCAGCC AAAACTTGAT GTGCTCTTTC

Opossum CAAGCTAAAA ACTTCATTTT CTAATCAGCC AAAACTTGAT GAGCTTTTTC

Platypus ---------- ---------- ---------- ---------- ----------

Chicken CAGGCTGAAG GCTTCTCTTC CCAAGCAGCA GAAACTTGAC AAAATGTTGC

Zebra_Finch CAAGTTAAAG AGTTCTGCAT CTGACCAGCA GAAGCTTGAC AAGATATTTC

Zebrafish GAAGCGCAAA GCCCCCCTCG CCAACCAGCA GGCCCTGGAC AAACTCTTCC

Fugu GAAACGCAAG GCCCCGCCGT CCAACCAGAA ACTGCTGGAC GAGCTCTTCC

Frog AAAATTAAAA ACACCCCTTT GTAACCAGCA AATACTGGAC AAGTTATTT-

Human AGTCCCAAAT TGAAAAAAGA AGGAGTCAAA ATATTAAAAT GGTACAGATC

Chimpanzee AGTCCCAAAT TGAAAAAAGA AGGAGTCAAA ATATTAAAAT GGTACAGATC

Gorilla AGTCCCAAAT TGAAAAAAGA AGGAGTCAAA ATATTAAAAT GGTACAGATC

Marmoset AGTCCCAAAT TGAAAAAAGG ---AGTCAAA ATATTAAAAT GGTACAGATC

Orangutan AGTCCCAAAT TGAAAAAAGG ---AGTCAAA ATATTAAAAT GGTACAGATC

Mouse AGTCCCAGAA TGAAAAGAAG AAGAGTGAAA ATATTAAAAT AACAGAGATT

Guinea_Pig AGTCCCAAAC TGAAAAT--- ---------- --ATTAAAAT AATACAGGTC

Rabbit AGTCTCATGT TGAAAAAAAA AGGAATGACA ATATTAAAAT AGTATCCATC

Cow AGGCCCAAAT CGAAAAAAAA AAA---CAAA ACATTAAAAT AGTTCAGATC

Dog AATCCCATAT TGAAAAAAAA ACT---CAAA ACATTAAAGT AGTTCAGATC

Horse AGTCCCAAAT TGAAAAAAAA AAGGACCAAA ACATTAAAGT AGTTCAGATC

Pig AGTCCCAATT TGAAAAAAAG AAC---CAAA ACATTAAAAT AGTTCAGATC

Elephant AGTCCCAAAT TGAAAAAAAA AAGACCCAAA GTATTAAAGT AGTACAGGTT

Opossum AAAGTGAGAA AAGGAAAAGC AAGTCACAAA ATATAAAAAT TTTACAGATT

Platypus ---------- ---------- ---------- ---------- ----------

Chicken AAATTGATAA GAAACCAAAG AGTAATCAGC CTGTGAAGGT TGTTACAGTG

Zebra_Finch AGATTGAAAA GAAGGGGAAA CTGGAACAGC CTGTGAAAAT TGTGACAGTG

Zebrafish AGCCCTCCAG TAAA------ AGGAGCCCTG CCAAAATCTC CAAACCCCTC

Fugu AGCCTCAGAA GAAGAGCGGG ACTCCTGCCC CCAAGCCCTC GCTGCCCCTT

Frog --CTGTCTCA AGAAAAGAAA AAAAACAAAC CAATTAGTAG TGTACAAGTA

Human CCCTTTTCTA TGAAAAACTT AAAAATAAAT TTTAAGAAAC AAAACAAAGT

Chimpanzee CCCTTTTCTA TGAAAAACTT AAAAATAAAT TTTAAGAAAC AAAACAAAGT

Gorilla CCCTTTTCTA TGAAAAACTT AAAAATAAAT TTTAAGAAAC AAAACGAAGT

Marmoset CCCTTTTCTA TGAAAAACTT AAAAATAAAT TTTGAGAAAC AAAACCAAGT

Orangutan CCCTTTTCTA TGAAAAACTT AAAAATAAAT TTTAAGAAAC AAGACAAAGT

Mouse CCCTTTTCTA TGGAAAACTT AAAAGCGAAC TTGAAGAAAC AAAAGGAAGT

Guinea_Pig CCTTTTTCTA TGAAAAACTT GAAAATAAAT TTTGAGAAAC AAAAGAGATT

Rabbit CCTTTCTCTA TGGAAAAGTT AAAAGTAAAT TTTGAGAAAC AAAATAAATT

Cow CCCTTCTCTA TGGAAAACTT AAAAAAGAAT TTTGATACAC AGAAGAAATT

Dog CCTTTTTCTA TGGAA----- ---------- ---------- ----------

Horse CCCTTTTCTA TGGAAAACTT ACAGAAAAAT TTTGAGAAAC AT---AAATT

Pig CCCTTCTCTA TGGAAAACTT AAAAAAGAAT TTTGATAAAC ATAACAAATT

Elephant CCCTTTTCTA TGAAAAACTT GAAAATTAAT TTTGAGAAGC ATAAGAAGTT

Opossum CCTTTCTCTA TGAAAACCTT AAAACTTAAG TATAGGAAGC ATATGAAAAG

Platypus ---------- ---------- ---------- ---------- ----------

Chicken CCTTTTTCTC TGAGTGCCTG TAGACCTCAG TTTCAGAGGC ATGAGAAG--

Zebra_Finch CCTTTTGCTC TGAGTTCCTG TAGACGTCAC CTTCAGAGAC CAGAAAGA--

Zebrafish CCGTTCACTA TCGCAACCCT CAAGCAGAGT CTTAACCTCC TCTCCGAGCA

Fugu CCATGCAACA TTCCCTCCCT TCGGCTGCGG CTTCAGCGCC TTTCCTCCCA

Frog GCCTTTAAGT TAAGCAAATT AAAACAACAG ATTTCTAGGC TCACAGACCA

Human TGACTTAGAA GAGAAGGATG AACCTTGCTT GATCCACAAT CTCAGGTTTC

Chimpanzee TGACTTAGAA GAGAAGGATG AACCTTGCTT GATCCACAAT CTCAGGTTTC

Gorilla TGACTTAGAA GAGAAGGATG AACCTTGCTT GATCCACAAT CTCAGGTTTC

Marmoset TGACTTAGAA GAGAAGGATG AACCTTGCTT GATCCACAAT CTCAGGTTTC

Orangutan TGACTTAGAA GAGAAGAATG AACCTTGCTT GATCCACAAT CTCAGGTTTC

Mouse TGACTTAGAG GAGAAGGATG AGATTTACCT GATCCACAGT CTCAAGTTTC

Guinea_Pig TGGCTTAGAA GACAAAGATG AGCTTTACTT GATCCACAAT CTCAAGTTTC

Rabbit TGACTTAGAA GACAAAGATG AACAATGTTT CATCCACAAT CTTAGCTTTC

Cow TGACTTAGAA GAAAAGGACG AATTTTGCTT GATCCACAAT CTCAAGTTTC

Dog -GACTTAGAA GATAAGGAAG AACTTTGCTT GATCCACAAT CTCAAGTTTC

Horse TGACTTAGAA GAAAAGGACG AACTTTGCTT GATCCACAAT CTCAAGTTTC

Pig TGACTTAGAA GAAAAGGATG AACTCTGCTT GATCCATAGT CTCAAGTTTC

Elephant TGACTTAGAA GAGAAGGATG AACTTTGCTT GATCCACAAT CTTAAGTTTC

Opossum TGATTTACTA TATAAAGAAG AACTTTGCCT GATCCATCAT TTAAGTTTTC

Platypus ---------- ---------- ---------- ---------- ----------

Chicken -AGTGATTCA GATAAGCATG AGCTTTGCCT GATCCGTCGC CAGAGTTTTC

Zebra_Finch -AATGTTTCG GATGAACACA AGCTTTTCCT GATCCGTCGT CAGAGTTTTC

Zebrafish GAGCAGCTCA AGTGTGCAGG GCCTCAGGCT TGTAAACCGC TTGGCTTCTC

Fugu GAGCAGCGCG GTGCCACGGG GCCTCCGTCT TGTAAACCAA CTGGCCTCTC

Frog ACAAAACTCT GTGAAAGAAG AATTTTGCTT CATAAATAAG CTAAGCTTCC

Human CTGATGCATG GCTAATGACA TCCAAAACAG AGGTAATGTT ATTAAATCCA

Chimpanzee CTGATGCATG GCTAATGACA TCCAAAACAG AGGTAATGTT ATTAAATCCA

Gorilla CTGATGCATG GCTAATGACA TCCAAAACAG AGGTAATGTT ATTAAATCCA

Marmoset CTGATGCATG GCTAATGACA TCCAAAACAG AGGTAATTTT ATTAAATCCA

Orangutan CTGATGCATG GCTAATGACA TCCAAAACAG AGGTAATGTT ATTAAATCCA

Mouse CTGATTCGTG GCTGGTCACG TCCAGAGCCG ATGTCATGTT ACTGAACCCA

Guinea_Pig CTGATGCCTG GCTAGTTACA TGCAAAACAG AGGTCATGTT ACTAAATCCA

Rabbit CTGATGCGTG GTTAATGATA TCCAAAACAG AGGTGATGTT ACTAAATCCA

Cow CTGACGCATG GCTGATTACA TCCAAAACAG AAATAATGTT GTTAAATTCA

Dog CTGATGCGTG GCTAATTACA TCCAAAACAG AGGTGATGTT ATTAAATCCT

Horse CTGATGGGTG GCTAATTACA TCCAAAACAG AGGTGATGTT ACTAAATCCA

Pig CTGATGCATG GCTAATTACA TCCAAAACAG AGATAATGTT ATTAAATCCA

Elephant CTGATGCCTG GCTAATTGCA TCCAAAAAAG AGGTAATGTT ATTAAATCCA

Opossum CTGATGCATG GATAATTGCT TCCCAAAAAG AGATCATGTT ATTAAACCCA

Platypus ---------- ---------- ---------- ---------- ----------

Chicken CTGATATCTG GATATTTGCT ACTGAAAAAA AGCTAATGTT GCTGAATCCA

Zebra_Finch CTGATGTCTG GATACTTGCT ACTGAAAACA ATGTTAAGTT GCTGAATCCA

Zebrafish ACGGTGCCTG GGTCGTTTTA TGCGGCAGAA AGCTCATGTT GTTGAACCCG

Fugu GGAGCGCCTG GGTCATTTTA TGCGGTCAGA GGCTCATGTT GTTAAACCCA

Frog CTGGTGCATG GATTGTAGCA TCAGGGTCAG AGATTGCTCT CTTGAATCCC

Human TATAGAGTAG AAGAAGCCCT GCTATTTAAA AGACTTCTTG AGAATCATAA

Chimpanzee TATAGAGTAG AAGAAGCCCT GCTATTTAAA AGACTTCTTG AGAATCATAA

Gorilla TATAGAGTAG AAGAAGCCCT GCTATTTAAA AGACTTCTTG AGAATCATAA

Marmoset TATAGAGTAG AAGAAGCCCT GCTATTTAAA AGACTTCTTG AGAATCATAA

Orangutan TATAGAGTAG AAGAAGCCCT GCTATTTAAA AGACTTCTTG AGAATCATAA

Mouse TACAGAGTGG AGGAAGCCCT GCTATTTAAA AGACTTCTTG AGAATCATAA

Guinea_Pig TATAGAGTAG AAGAAGCCCT GCTATTTAAG AGACTTCTTG AGGATCATAA

Rabbit TATAGAGTAG AGGAAGCCCT GCTATTTAAA AGACTTCTTG AGAACCATAA

Cow TATAGAGTGG AAGAAGCTCT GCTATTTAAA AGACTTCTTG AGAATCACAA

Dog TACAGAGTGG AAGAAGCCCT CCTATTTAAA AGACTTATTG AGAATCATAA

Horse TATAGAGTGG AAGAAGCCCT GCTGTTTAAA AGACTTCTTG AGAATCATAA

Pig TATAGAGTGG AAGAAGCTCT GCTGTTTAAA AGACTTCTTG AGAATCATAA

Elephant TATAGAATGG AAGAAGCCCT GCTGTTTAAA AGACTTCTTG AGAATCATAA

Opossum TACAGAGTGG AAGAAGCTTT ACTATTTAAA AGACTTCTTG AAAATCATAA

Platypus ---------- ---------- ---------- ---------- ----------

Chicken TATAGATTGG AAGAAGCCCT GCTGCGTAAA AGATTGCTGA TGAACCATAA

Zebra_Finch TATAGATTGG AAGAGGCCCT GCTATGTAAG AGATTATTGG TGAACCATAA

Zebrafish TTTCGAGTGG AGGAGGCCTT GCTGTTTAAA AGACTTCTGG AGGATAATAT

Fugu TTTCGAGTGG AGGAAGCCTT GCTGTTTAAG AGACTTCTAG AGAATAATAT

Frog TATAGGGCTG AGGAAGCTTT GATATTTAAA CAGCTTTTAA AGAACCATAA

Human ACTTCCTGCA GAGCCACTGG AAAAGCCAAT TATGTTAACA GAGAGTCTTT

Chimpanzee ACTTCCTGCA GAGCCACTGG AAAAGCCAAT TATGTTAACA GAGAGTCTTT

Gorilla ACTTCCTGCA GAGCCACTGG AAAAGCCAAT TATGTTAACA GAGAGTCTTT

Marmoset ACTTCCTGCT GAGCCACTGG AAAACCCAAT TATGTTAACA GAGAGTCTGT

Orangutan ACTTCCTGCA GAGCCACTGG AAAAGCCAAT TATGTTAACA GAGAGTCTTT

Mouse ACTCCTTGCA GAACCACTGG AAAAGCCAAT TATATTAACA GAGAGTCTTC

Guinea_Pig ACTTCCTGCA GAGCCACTGG AAAAGCCAAT TATATTAACA GACAGTCTTT

Rabbit ACTTCCTGCA GAGCCACTGG AAAAGCCAAT TATTTTAACA GAGAGTCTTT

Cow ACTTCCTGCA GAGCCACTGG AAAAACCAAT TATATTAACA GAGAGTCTTT

Dog ACTTCCTGCA CAGCCCCTGG AAAAGCCAAT TATATTAACA GAGAGTCTTT

Horse ACTTCCTTCA GAGCCCCTGG AAAAGCCAAT TATATTAACA GAGAGTCTTT

Pig ACTTCCTGCA GAACCACTGG AAAAGCCAAT TATATTAACA GAGAGTCTTT

Elephant ACTTCCTGCA GAGCCACTGG AAAATCCAAT TATACTAACA GAGAGTCTTT

Opossum ACTTCCTGCA GAGCCATTGG AGAAGCCAAT GATATTAACA GAGAGTCTTT

Platypus ---------- ---------- ---------- ---------- ----------

Chicken GCTTCCAGTA GAGAAACTGG AGAAGCCAGT TGTGTTATCA GACAGTCTTC

Zebra_Finch GCTTCCTGTA GAGAAACTGG ACACTCCAAT TGTGTTAACA GACAGTCTTA

Zebrafish ACTTCCAACC GTGAGGCTGC AGACCCCCGT ACTGCTGACA GATGGGGTGC

Fugu ACTCCCAGCA GTGAGTCTGC AGACCCCTAT ACAGTTAACA GATGGAAGTC

Frog AATATCTGCA GAAAAGTTGG ATTCTCCAAT TGTAATAACT GATAGCCTCT

Human TTAATGGATC TCATTATTTA GACGTTTTAT ATAAAATGAC AGCAGATGAC

Chimpanzee TTAATGGATC TCATTATTTA GACGTTTTAT ATAAAATGAC AGCAGATGAC

Gorilla TTAATGGATC TCATTATTTA GACGTTTTAT ATAAAATGAC AGCAGATGAC

Marmoset TTGATGGATC TCATTATTTA GACATTTTAT ATAAAATGAC AGCCGGTGAC

Orangutan TTAATGGATC TCATTATTTA GACGTTTTAT ATAAAATGAC AGCAGATGAC

Mouse TTAATGGGTC TCATTATCTA GAGGTTTTAT ATAAAATGTC AACAGTTGAA

Guinea_Pig TTAATGGATC TCATTATTTA GAGGTCTTAT ATAAAATGAC AACTGATGAC

Rabbit TTAATGGATC TCATTATTTA GAGATTTTAT ATAAAATGAC AGCAGATGAC

Cow TTAATGGATC CCATTATTTA GAGATTTTAC ATAAAATGAC AGCAGATGAC

Dog TTAATGGATC TCATTATTTA GAGATGTTAT GTAAAATGAC CACAGATGAT

Horse TTAATGGATC TCATTATTTA GAGATTTTAT ATAAAATGAC AGCAAATGAT

Pig TTAATGGATC TCATTATTTA GAGATTTTAT ATAAAATGAC AACAGATGAC

Elephant TTGGTGGATC TCATTATTTA GAGGTTTTAT GTAAAATGAC AACAGATGAC

Opossum TTAATGGGTC CATTTATTTA GAGGTCTTAA GTAAAATGAC AATGGATGAT

Platypus ---------- ---------- ---------- ---------- ----------

Chicken TTGGTGAATC TCACTATATG GATGTTCTTC ATAAAATGCA GAAGGATTAC

Zebra_Finch TTGGTGGAGC TCAGTATATG GCTGCTCTCT ATAAAATGCA GAAGAATTAC

Zebrafish TCGGTGGGCC AGAGTACATG GATGTCCTTC TGAACATGAA GAAAGATGGT

Fugu TAGGAGGAGC AGAATATACC AAAGCCTTGT GCAGCATGGA GAAACAGAGC

Frog TAGGAGGGTC GCAGTATTTA GATGCTCTGT TAAGCATGCA AAAGGACTCC

Human CAAAGATACA GTGGATCAAC TTACCTGTCT GATCCTCGTC TTACAGCGAA

Chimpanzee CAAAGATACA GTGGATCAAC TTACCTGTCT GATCCTCGTC TTACAGCGAA

Gorilla CAAAGATACA GTGGATCAAC TTACCTGTCT GATCCTCGTC TTACAGCGAA

Marmoset CAGAGATTCA GTGGATCAAC TTACCTGTCT GATCCTCGTC TTACAGCAAA

Orangutan CAAAGATACA GTGGATCAAC TTACCTGTCT GATCCTCGTC TTACAGCGAA

Mouse GAAAGAGGCA GTGGATCAGC TTACCTGTGT GACCCTCGTC TTACAGCAAA

Guinea_Pig CAGCGATATG ATGGATCAAG TTATCTATCA GATCCTCGTC TTACAGCAAA

Rabbit CAGCAATACA GTGGATCAAC TTACCTGTCT GATCCTCGTC TTACAGCAAA

Cow CAAAGGTACA GCGGATCAAT TTACCTGTCC GATCCTCGTC TTACAGCAAA

Dog CAGAGATATA GTGGATCGAC TTACCTGTCC GATCCTCGTC TTACAGCAAA

Horse CAAGGATACA GTGGATCAAC TTACCTGTGC GATCCTCGTC TTACAGCAAA

Pig CCAAGATACA GTGGATCAAC TTATCTGTCT GATCCTCGTC TTACTGCAAA

Elephant CAAGGATACA GTGGATCAAC CTACCTGTCT GATCCTCGTC TTACAGCAAA

Opossum CAAGATTTCA GTGGCTCAAC CTACTTGTCT GATCCACGCC TTACAGCAAA

Platypus ---------- --GGGTCGAC TTCACTGACC GATCCACGTC TTGTAGCGAA

Chicken CAGGGATTAA ATGGATTGAG TTATTTGTCA GATCCAAGGC TTGTAGCAAA

Zebra_Finch CACAGTTTCC ATGGATCAGG TTATTTGTCA GATCCAAGGC TTGTAGCAAA

Zebrafish CCTGAATTTA ATGGAGACAT CAGCTTAACT GATCCCAGGC TTGTGGCAAA

Fugu CCCGAATTAA GCGGAGAGGT GTTCTTCTGT GATCCCCGGC TCGTTGCCAA

Frog CCAAAGCCAA ATGGAGAAAC ATACTTTTCA GATCCCAGAC TTACTGCTAA

Human TGGTTTCAAG ATAAAATTGA TACCAGGAGT TTCAATTACT GAAAATTACT

Chimpanzee TGGTTTCAAG ATAAAATTGA TACCAGGAGT TTCAATTACT GAAAATTACT

Gorilla TGGTTTCAAG ATAAAATTGA TACCAGGAGT TTCAATTACT GAAAATTACT

Marmoset TGGTTTCAAG ATAAAATTGA TACCAGGAGT TTCAATTACC GAAAATTACA

Orangutan TGGTTTCAAG ATAAAACTGA TACCAGGAGT TTCAATTACT GAAAATTACT

Mouse TGGCTTCAAG ATCAGATTGA CACCAGGAGT TTCCAGTACT GAGAACTACT

Guinea_Pig TGGTTTCAAG ATAAAGTTGA TACCAGGAGC TTCTACTGCT GAAAACTACT

Rabbit TGGTTTCAAG ATAAAATTGA TACCAGGAGT TTCCATTGCT GAAAACTACT

Cow TGGTTTCAAG ATAAAATTGA TACCAGTTAT AGGCATGACA CAGCGATTTT

Dog CGGTTTCAAG ATAAAATGGA TACCAGGTGT TTCCATTGCT GAAGACTACT

Horse TGGTTTCAAG ATAAAATTGA TACCAGGAGT TTCTATTGCA GAAAACTACT

Pig TGGTTTCAAG ATAAAATTGA TACCAGGAGC TTCCATGGCT GAAAACTATT

Elephant TGGTTTCAAG ATAAAATTAA TACCAGGAGT TTCCATTGCT GAAAACTACT

Opossum TGGATTTAGG ATAAAAATGA TACCAGGAAC TTCAGTCACT GAAAACCATC

Platypus CGGTTTCGAG ATCAGACTGA TTCCAGGGCC GTCGGCTGAA GAAGATCGCC

Chicken TGGCTTCCAG ATAAAAGTGA TAGAAGGTGC TTCAGCTACA GAAAGCCATA

Zebra_Finch TGGATTCCAG ATAAAAGTGA TAGAAGGTGT TTCGGCTACA GAAAGTCATC

Zebrafish CGGTTTTGAG ATTAGGATGA TTTCAGGTCC GCAGTCTTCG GAGAGGCATG

Fugu TGGCTTTAAA GTCAGACTCA TCCCAGCGCT CCCGTCAGCT GAGAGACATC

Frog TGGGTTTCTA ATTAAAATAA TGCCAGGAAA GTCACCTGTT GAGAACCACA

Human TGGAAATAGA AGGAATGGCT AATTGTCTCC CATTCTATGG AGTAGCAGAT

Chimpanzee TGGAAATAGA AGGAATGGCT AATTGTCTCC CATTCTATGG AGTAGCAGAT

Gorilla TGGAAATAGA AGGAATGGCT AATTGTCTCC CATTCTATGG AGTAGCAGAT

Marmoset TGGAAATAGA AGGAATGGCT AATTGTCTCC CATTCTATGG AATAGCAGAT

Orangutan TGGAAATAGA AGGAATGGCT AATTGTCTCC CATTCTATGG AGTAGCAGAT

Mouse TGGAAATAGA AGGAATGGCT CAGTGTCTCC CATTCTATGG AATAATGGAT

Guinea_Pig TGGAAATAGA AGGAATGGCT AATTGTCTCC CATTCTATGG AATAATGGAT

Rabbit TGGAAATAGA AGGAATGGCT AATTGTCTCC CCTTCTATGG AATAACAGAT

Cow TACCCATATC CACAAAATGT TCCTGCATTC CATTCTATGG AGTAATGGAT

Dog TGGAAATAGA AGAAATGGCT AATTGCCTCC CATTCTATGG AGTGATGGAC

Horse TGGAAATAGA AGGAATGGCT AATTGTCTCC CATTCTATGG AGTGATGGAT

Pig TGGAAATAGA AGGAATGGCT AATTGTCTTC CATTCTATGG AGTAATGGAT

Elephant TAGAAATAGA AGGAATGGCT AATTGTCTTC CATTCTATGG AGTAATGGAT

Opossum TGGAAATAGA AGGAATGGCT AATTGTCTCC CATTCTATGG AATATTGGAT

Platypus TGGAGATAGA GGGGCTGGCC AGCTGCCTGC CCTTCTACGG GGTCTCGGAC

Chicken TGGAAATAGA AGGAATGGCT AATTGTCTGT CGTATTATGG CATTTCTGAT

Zebra_Finch TGGAAATAGA AGGGATGGCA AATTGTCTGC CATATTATGG AATCTCTGAT

Zebrafish TTGAGGTGAT GGGCATGGCC GACTGCATGC CGTTCTTCGG GATAGGAGAC

Fugu TGGAAGTGAC AGCCATGGCG GACTGCGTGC CTTTCCTCGG TGTGGAGGAC

Frog TTGCGATTGA AGGGATGGCT AGCGGCTTGC CTTTTTATGG AGTGTCAGAT

Human TTAAAAGAAA TTCTTAATGC TATATTAAAC AGAAATGCAA AGGAAGTTTA

Chimpanzee TTAAAAGAAA TTCTTAATGC TATATTAAAC AGAAATGCAA AGGAAGTTTA

Gorilla TTAAAAGAAA TTCTTAATGC TATATTAAAC AGAAATGCAA AGGAAGTTTA

Marmoset TTAAAAGAAA TTCTTAATGC TATCTTAAAC AAAAATGCAA AGGAAGTTTA

Orangutan TTAAAAGAAA TTCTTAATGC TATATTAAAC AAAAATGCAA AGGAAGTTTA

Mouse TTAAAGGAAA TTCTTAATGC TATAGTAAAC AAAAATGCAA AGGAGATTTA

Guinea_Pig TTAAAAGAAA TTCTTAATGC TATATTAAAC AAAAATGCAA AAACAATTTA

Rabbit TTAAAAGAAA TTCTTAATGC CATATTATAC AAAAATGCAA AGGAAATATT

Cow TTAAAAGAAA TCCTTAATGC TATATTAAAC AAAAATGCAA AGGAAGTTTA

Dog TTAAAAGAAA TTCTTAATGC TATATTAAAC AAAAATGCAA AAGAAGTTTA

Horse TTAAAAGAAA TTCTTAATGC TATATTAAAC AAAAATGCCA AGGAAGTTTA

Pig TTAAAAGAAA TTCTTAATGC TATATTAAAT AAAAATGCAA AGGAAGTTTA

Elephant TTAAAAGAAA TTCTGAATGC TATATTAAAC AAAAATGCAA AGGAAGTTTA

Opossum TTAAAGGAAA TTCTTAATGC GGTGCTAAAT AAAAATGCGA AGGAAGTTTA

Platypus CTCAAGGAGA TCCTGGGCGC CGTGCTGGCC GGAGATGCCC AGGAGCTGCG

Chicken CTGAAAGAAA TTCTGAATGC AGTGGTTAAT AGAAATGCAA AGGAAGTGTA

Zebra_Finch TTGAAAGAAA TTCTGAATGC AGTAGTTAAC AGGAATGCAA AGGAAGTATA

Zebrafish CTGAGGGAGA TTCTGCAGGC TATAAAGGCC CGCGGAGCAA AAACCGTGGC

Fugu CTCAGGGAGA TCCTGACCGC AGTTCTTCAC GGGAAGGCGC GGAGCGTGAA

Frog CTAAAGGAAA TCCTAAGTTT TATACTAATT AACAAAAGCA ATAAACTATG

Human TGAATGTAGA CCTCGCAAAG TGATAAGTTA TTTAGAGGGA GAAGCAGTGC

Chimpanzee TGAATGTAGA CCTCGCAAAG TGATAAGTTA TTTAGAGGGA GAAGCAGTGC

Gorilla TGAATGTAGA CCTCGCAAAG TGATAAGTTA TTTAGAGGGA GAAGCAGTGC

Marmoset TGAATGTAGA CCTCGCAAAG TGATAAGTTA TTTAGAGGGA GAAGCAGTGC

Orangutan TGAATGTAGA CCTCGCAAAG TGATAAGTTA TTTAGAGGGA GAAGCAGTGC

Mouse TGAATGTAGA CCTCGAAAAG TAATAAATTA TCTGGAGGGA GAAGCCGTCC

Guinea_Pig TGAATGTAGA CCTCGCAAAG TGATAAGTTA TTTAGAGGGA GAAGCAGTTC

Rabbit TGAATGTAGA CCTCGTAAAG TAATAAATTA TTTAGAGGGA GAAGCAGTGC

Cow CGAATGTAGA CCTCGCAAAG TGATAAGTTA TTTAGAGGGA GAAGCAGTAC

Dog TGAATGTAGA CCTCGAAAAG TGATAAGTTA TTTAGAGGGG GAAGCAGTGC

Horse TGAATGTAGA CCTCGCAAAG TGATAAGTTA TTTAGAGGGA GAAGCGGTGC

Pig TGAATGTAGA CCTCGCAAAG TGATGAGTTA CTTAGAGGGA GAAGCAGTAC

Elephant CGAATGTAGA CCTCGAAAAG TGATAAGTTA TCTAGAGGGA GAAGCAGTGC

Opossum TGAATGTAGA CCTCGTAAAG TAGTTAGTTA TTTAGAGGGA GAAGCAGTAC

Platypus TCAGTGCCGG CCACGCAAAG TGGTCAACTA CTTACAGGGA GAAGCGGTGC

Chicken TGAATGTAGA CCTCTCAAAG TGATAAACTA CTTGGAAGGA GAAGCAGTGC

Zebra_Finch TGAATGTAGG CCTCTCAAAG TGATAAATTA CTTGGAGGGA GAAGCAGTAT

Zebrafish GCAATGTAGA CCTCGCAAAG TCTCACACTA CCTCGAGAGC GAGGCAGTGA

Fugu AGAGTGTCGA CCACTTAAAG TCACAAACTA CTTAAAAGGC GAAGCGGTGC

Frog TGACTGTAGA CCAATTAAAG TGTGGAATTA TCTTGAGGGA GAAGCTGTAA

Human GTCTATCCAG ACAATTACCC ATGTACTTAT CAAAAGAGGA CATCCAAGAC

Chimpanzee GTCTATCCAG ACAATTACCC ATGTACTTAT CAAAAGAGGA CATCCAAGAC

Gorilla GTCTATCCAG ACAATTACCC ATGTACTTAT CAAAAGAGGA CATCCAAGAC

Marmoset GTCTATCCAG ACAATTACCC ATGTACTTAT CAAAAGAAGA CATCCAAGAC

Orangutan GTCTATCCAG ACAATTACCC ATGTACTTAT CAAAAGAGGA CATCCAAGAC

Mouse GTCTATCCAG ACAGCTACCC ATGTACTTAC CAAGAGAGGA TGTCCAAGAT

Guinea_Pig GACTGTCTCG ACAGTTACCC ATGTACTTAC CAAAAGAAGA CATCGAAGAT

Rabbit GTTTATCTAG ACAGTTACCC ATGTACTTAC CAAAAGAGGA CATCCAAGAT

Cow GTCTGTCTAG ACAATTACCC ATGTACTTAT CAAAAGAGGA CATGCAAGAT

Dog GTCTGTCTAG ACAGTTGCCT ATGTACTTAT CCAAAGAGGA CGTCCAAGAC

Horse GTCTATCTAG ACAGTTACCC ATGTACTTAT CAAAAGAGGA TGTCCAAGAC

Pig GTCTATCTAG ACAGTTACCC ATGTACTTAT CAAAAGAAGA CATCCAAGAC

Elephant GTCTGTCTAG ACAGCTACCC ATGTACTTAT CGAAAGAGGA CATCCTAGAC

Opossum GTCTTTCTCG ACAGCTACCT TTGTATTTAT CAAAAGAGGA TGTGCAAGAT

Platypus GCCTGTCCCG CCAACTGCCC CTTCACCTGT CCAAGGAGGA CGTGCTGGAC

Chicken GTTTAGCTCG GCAGCTGCCC TTACATTTGT CAAAAGAAGA TGTAGAAAAC

Zebra_Finch GTTTAGCACG GCAGCTGCCC TTACATTTGT CAAAAGAAGA TGTGCAAAAC

Zebrafish GACTAGCAAG ACAGTTACCT CTCAGTCTCT CAAGAGCCGA TGTTACAAAC

Fugu GACTTGTCCG TCAGCTGCCC GCGAGTCCGT CCCGGGCGGA CGTGGAGGAA

Frog GATTGTCTCG CCAGCTACCT ATAAACCTTT CCAAAGAAGA TGTGCATGAC

Human ATTATCTACA GAATGAAGCA CCAGTTTGGA AATGAAATTA AAGAGTGTGT

Chimpanzee ATTATCTACA GAATGAAGCA CCAGTTTGGA AATGAAATTA AAGAGTGTGT

Gorilla ATTATCTACA GAATGAAGCA CCAGTTTGGA AATGAAAATA AAGAGTGTGT

Marmoset ATTATCTACA GAATGAAGCA CCAGTTTGGA AATGAAATTA AAGGATGTGT

Orangutan ATTATCTACA GAATGAAGCA CCAGTTTGGA AATGAAATTA AAGGGTGTGT

Mouse CTTATCTATA GAATGAAGCA CCAGTTCGGA AAAGAAATTA AAGGCTGTGT

Guinea_Pig ATTATCTGTA GAATGAAGCA CCAGTTTGGA AATGTAATTA AAGGGTGTGT

Rabbit ATTATCTACA GAATGAAGCA TCAATTTGGA AATGAAATTA AAGGATGTGT

Cow ATTATCTACA GAATGAAGCA CCAGTTTAGA AATGAAATTA CACAGTGTGT

Dog ATTATCTATA GAATGAAGGA CCAATTTGGA AGTGAAATTA AAGAGTGTGT

Horse ATTATCTACA GAATGAAGCA CCAATTTGGA AATGAAATTA AAGGGTGTGT

Pig ATTATCTACA GAATGAAGAA CCAGTTTGGA AATGAAATTA AAGGGTGTGT

Elephant ATTGTCCACA GAATAAAGCA CCAACTTGGA AGTGAAATTA AAGGGTGTGT

Opossum ACTATATATA GGATGAAGCT ACAACTGGGA GATGACATTA AGGGATGTGT

Platypus ACGATGGCCA GGATGAAAGA CCAGCTCGGG AGCGAGACCC AGACCTGCGT

Chicken ACAATTTACA GGATGAAGCA ACAGCTTGGA AAGGAAAATA AAGGCTGTGT

Zebra_Finch ACAATTTACA GGATGAAGCA ACAGCTTGGA AAGGAAAATA AAGGCTGTGT

Zebrafish ACACTTCGCC GAATGCAACA GGAACTTCAA GACGAAAGTC AGGTCTGCAT

Fugu ACGCTGCGGA GGATGGAGCG GCAGCTTGGT GAGAAGAACC GGACCTGCAT

Frog ATCATGCACA GGCTAAACAA TCAATGTGGA AGTGAAACTA AAGCTTGTCT

Human TCATGGTCGC CCATTTTTTC ATCATTTAAC CTATCTTCCA GAAACTACA

Chimpanzee TCATGGTCGC CCATTTTTTC ATCATTTAAC CTATCTTCCA GAAACTACA

Gorilla TCATGGTCGC CCATTTTTTC ATCATTTAAC CTATCTTCCA GAAACTACA

Marmoset TCATGGTCGT CCATTTTTTC ATCATTTAAC CTATCTTCCA GAAACTACA

Orangutan TCATGGTCGT CCATTTTTTC ATCATTTAAC CTATCTTCCA GAAACTACA

Mouse TCATGGCCGC CCATTTTTTC ATCATTTGAC CCATCTTCCA GGAACTTCA

Guinea_Pig TCATGGCCGC CCATTTTTTC ATCATTTAAC CCATCTTCCA GAAACTTCA

Rabbit TCATGGTCGG CCATTTTTTC ATCACTTAAC ACATATTCCA GAAACAACA

Cow TCATGGTCGC CCATTTTTTC ATCATTTAAC CCATCTTCCA GAAGCTACA

Dog TCATGGTCGC CCATTTTTTC ATCATTTGAC ACATCTTCCA GAAGCTACC

Horse TCATGGTCGC CCATTTTTTC ATCATTTAAC CCATCTTCCA GAAGCTACA

Pig TCATGGTCGC CCCTTTTTTC ATCATTTAAC TAGTCTTGCT GAAGCTACA

Elephant TCATGGTCGC CCGTTTTTTC ATCATTTAAC TCATCTTCCA GAAACT---

Opossum TCATGGTCGC CCATTTTTTC ATCACTTAAC ATACCTTCCA AAAACTATA

Platypus CCACGGCCGA CCGTTCTTCC ATCACTTGGC GGATATCCCG CGGGCCGCT

Chicken TCATGGTCGT CCTTTTTTTC ACCACTTAAC AGATATTCCA GAAGTTAAC

Zebra_Finch TCATGGTCGT CCTTTTTTTC ATCACTTAAC AGATATTCCA GAAGTTGAC

Zebrafish TCACAGGCTG CCATTTTTTC ACAATCTGGT GACCCTTCCT GAAACCGAG

Fugu CCACGGCCGA CCCTTCCTCC AACACATGGG CGACGTCCCT TCTTCAGAA

Frog CCATGGTCAT CTGTTTTTTC ATCATTTAGC AGATGTTCCT AAAACTGAT

;

END;

BEGIN DATA;

DIMENSIONS NTAX=19 NCHAR=2274;

FORMAT DATATYPE=DNA INTERLEAVE=yes GAP=-;

[Gene Name: MLH1]

[Name: Human Len: 2274 Check: 0]

[Name: Chimpanzee Len: 2274 Check: 0]

[Name: Gorilla Len: 2274 Check: 0]

[Name: Orangutan Len: 2274 Check: 0]

[Name: Rat Len: 2274 Check: 0]

[Name: Guinea_Pig Len: 2274 Check: 0]

[Name: Mouse Len: 2274 Check: 0]

[Name: Rabbit Len: 2274 Check: 0]

[Name: Dog Len: 2274 Check: 0]

[Name: Elephant Len: 2274 Check: 0]

[Name: Cow Len: 2274 Check: 0]

[Name: Horse Len: 2274 Check: 0]

[Name: Opossum Len: 2274 Check: 0]

[Name: Platypus Len: 2274 Check: 0]

[Name: Frog Len: 2274 Check: 0]

[Name: Chicken Len: 2274 Check: 0]

[Name: Zebra_Finch Len: 2274 Check: 0]

[Name: Fugu Len: 2274 Check: 0]

[Name: Zebrafish Len: 2274 Check: 0]

MATRIX

Human ATGTCGTTCG TGGCAGGGGT TATTCGGCGG CTGGACGAGA CAGTGGTGAA

Chimpanzee ATGTCGTTCG TGGCAGGGGT TATTCGGCGG CTGGACGAGA CAGTGGTGAA

Gorilla ATGTCGTTCG TGGCAGGGGT TATTCGGCGG CTGGACGAGA CAGTGGTGAA

Orangutan ATGTCGTTCG TGGCAGGCGT TATTCGGCGG CTGGACGAGA CAGTGGTGAA

Rat ATGGCGTTTG TAGCAGGAGT TATTCGGCGC CTGGACGAGA CGGTAGTGAA

Guinea_Pig ATGTCGCTTA TAGCGGGGGT TATTCGGCGG CTTGATGAGA CAGTGGTCAA

Mouse ATGGCGTTTG TAGCAGGAGT TATTCGGCGT CTGGACGAGA CGGTAGTGAA

Rabbit ATGTCGCTCG TGGCGGGGGT TATCCGGCGG CTGGACGAGA CAGTGGTGAA

Dog ATGTCGCTCG TAGCGGGGGT TATTCGGCGG TTGGACGAGA CAGTGGTGAA

Elephant ATGTCGTTCG TGGCGGGGGT GATCCGCCGG CTAGACGAGA CAGTGGTGAA

Cow ATGTCGCTTG TAGCGGGGGT TATCCGGCGG CTGGACGAGA CAGTGGTGAA

Horse ATGTCGCTTG TAGCAGGGGT TATCCGGCGC CTGGATGAGA CAGTGGTGAA

Opossum ATGTCGCTAG TAGCCGGGGT GATTAGGCGT CTGGATGAGA AAGTGGTGAA

Platypus ---------- ---------- ---------- ---------- ----------

Frog CTGTTCATAA TGGCGGGGGT TATTAGGCGT CTGGAAGAGA CAGTGGTTAA

Chicken ATGGCGCTGG TAGCTGGCGT CATCCGGAGG CTGGATGAGG CTGTGGTGAA

Zebra_Finch ---------- ---------- ---------- ---------- ----------

Fugu GCTGCGAACA TGGCAGGCGT TATCCGGAGG CTCGACGAGA CTGTTGTCAA

Zebrafish ------ATGA TGGCGGGAGT CATCCGCAGA CTCGACGAGA CTGTAGTGAA

Human CCGCATCGCG GCGGGGGAAG TTATCCAGCG GCCAGCTAAT GCTATCAAAG

Chimpanzee CCGCATCGCG GCGGGGGAAG TTATCCAGCG GCCAGCTAAT GCTATCAAAG

Gorilla CCGCATTGCG GCGGGGGAAG TTATCCAGCG GCCAGCTAAT GCTATCAAAG

Orangutan CCGCATCGCG GCGGGGGAAG TTATCCAGCG GCCAGCTAAT GCTATCAAAG

Rat TCGCATAGCG GCGGGAGAAG TTATCCAGCG GCCGGCCAAT GCTATCAAAG

Guinea_Pig CCGCATTGCG GCAGGAGAAG TTATCCAGCG TCCTGCTAAT GCCATCAAAG

Mouse CCGCATAGCG GCGGGGGAAG TCATTCAGCG GCCGGCCAAT GCTATCAAAG

Rabbit CCGCATCGCG GCGGGGGAAG TTATCCAGCG GCCGGCTAAT GCCATCAAAG

Dog CCGCATCGCG GCAGGGGAAG TTATCCAGCG GCCGGCTAAT GCCATCAAGG

Elephant CCGCATCGCG GCGGGAGAGG TTATCCAGCG ACCGGCTAAT GCCATCAAGG

Cow CCGCATCGCG GCAGGGGAAG TTATCCAGCG GCCGGCTAAT GCCATCAAGG

Horse CCGCATTGCA GCAGGGGAAG TTATCCAGCG TCCGGCTAAT GCCATCAAGG

Opossum CCGCATCGCT GCAGGGGAGG TCATCCAGCG CCCCGCGAAC GCCATCAAGG

Platypus ---------- ---------- ---------- ---------- ----------

Frog TCGTATAGCT GCTGGGGAGG TTATCCAGAG GCCGGCCAAT GCAATCAAGG

Chicken CCGCATCGCG GCCGGCGAGG TCATCCAGAG ACCGGCAAAC GCCATCAAGG

Zebra_Finch ---------- ---------- ---------- ---------- ----------

Fugu CCGAATCGCC GCTGGTGAAG TTATCCAACG TCCTGCTAAT GCCGTCAAAG

Zebrafish CCGCATCGCA GCCGGAGAGA TTATCCAGCG GCCTGCCAAT GCTATCAAAG

Human AGATGATTGA GAACTGTTTA GATGCAAAAT CCACAAGTAT TCAAGTGATT

Chimpanzee AGATGATTGA GAACTGTTTA GATGCAAAAT CCACAAGTAT TCAAGTGATT

Gorilla AGATGATTGA GAACTGTTTA GATGCAAAAT CCACAAGTAT TCAAGTGATT

Orangutan AGATGATTGA GAAC------ ---------- ---------- ----------

Rat AGATGATTGA AAACTGTTTA GATGCAAAAT CTACAAATAT TCAAGTGATT

Guinea_Pig AGATGATTGA AAACTGTTTA GATGCAAAGT CCACAAGTAT TCAAGTGATT

Mouse AGATGATAGA AAACTGTTTA GATGCAAAAT CTACAAATAT TCAAGTGGTT

Rabbit AAATGATTGA GAACTGTTTA GATGCAAAAT CCACGAGTAT TCAAGTTGTT

Dog AGATGATTGA GAACTGTTTA GATGCAAAAT CCACCAGTAT CCAAGTGGTT

Elephant AGATGATTGA GAACTGTTTA GATGCGAAAT CTACAAGTAT TCAAGTGGTT

Cow AAATGATTGA GAACTGTTTA GATGCAAAAT CCACAAGTAT TCAAGTGGTC

Horse AGATGATTGA GAACTGTTTG GATGCAAAAT CCACAAGTAT TCAAGTGGTT

Opossum AGATGATCGA GAACTGCCTG GACGCCCGGG CCTCGGCCAT CCAAGTGACG

Platypus ---------- -------TTG GATGCCAAAT CCACAAGTAT CCAGGTGACG

Frog AAATGATTGA AAACTGTTTA GATGCCAAAT CGACCTCCAT CCAGGTGACA

Chicken AGATGATAGA GAACTGTTTG GATGCTAAAT CTACAAGTAT CCAGGTAGTA

Zebra_Finch ---------- -------TTG GATGCTAAAT CTACGAGTAT CCAGGTAGTA

Fugu AACTGATAGA GAACTGTCTG GATGCCAAGT CCACCAACAT CCAGGTGACA

Zebrafish AGATGATGGA GAACTGTTTG GATGCAAAGT CTACGAACAT CCAGATAACA

Human GTTAAAGAGG GAGGCCTGAA GTTGATTCAG ATCCAAGACA ATGGCACCGG

Chimpanzee GTTAAAGAGG GAGGCCTGAA GTTGATTCAG ATCCAAGACA ATGGCACCGG

Gorilla GTTAAAGAGG GAGGCCTGAA GTTGATTCAG ATCCAAGACA ATGGCACTGG

Orangutan ---------- ---------- ---------- ---------- ----------

Rat GTTAAAGAAG GAGGCCTGAA GCTAATTCAG ATCCAAGACA ATGGCACTGG

Guinea_Pig GTTAAAGAGG GAGGTCTAAA GTTGATTCAG ATCCAAGACA ATGGCACTGG

Mouse GTTAAGGAAG GTGGCCTGAA GCTAATTCAG ATCCAAGACA ATGGCACTGG

Rabbit GTTAAAGAAG GAGGCCTGAA GTTGATTCAG ATCCAAGACA ATGGCACTGG

Dog GTTAAAGAGG GAGGCCTCAA GTTGATTCAG ATCCAAGACA ATGGCACTGG

Elephant GTGAAAGAGG GAGGCCTGAA ATTGATTCAG ATCCAAGACA ACGGCACTGG

Cow GTTAAAGAGG GAGGCCTGAA GTTGATTCAG ATTCAAGACA ATGGCACTGG

Horse ATTAAAGAGG GAGGCCTGAA GCTGATTCAG ATCCAAGACA ATGGCACTGG

Opossum GTGAGAGAGG GCGGACTGAA GCTGATCCAG GTCCAGGACA ATGGCAGCGG

Platypus GTTAAAGAAG GAGGGCTGAA ATTAATTCAG ATCCAAGACA ATGGCACCGG

Frog GTGAAGGATG GAGGCATGAA ACTTATCCAG ATTCAGGACA ACGGAACTGG

Chicken GTCAAAGAAG GTGGTCTGAA GCTCATCCAG GTCCAAGATA ACGGCTGTGG

Zebra_Finch GTTAAAGAAG GTGGTCTGAA GTTCATCCAG GTTCAGGATA ACGGCTGTGG

Fugu GTGAAAGATG GCGGACTGAA GGTTCTACAG ATTCAGGATA ACGGCACTGG

Zebrafish GTGAAAGAGG GCGGACTGAA ACTCATCCTT ATTCAGGACA ATGGCACTGG

Human GATCAGGAAA GAAGATCTGG ATATTGTATG TGAAAGGTTC ACTACTAGTA

Chimpanzee GATCAGGAAA GAAGATCTGG ATATTGTATG TGAAAGGTTC ACTACTAGTA

Gorilla GATCAGGAAA GAAGATCTGG ATATTGTATG TGAAAGGTTC ACTACTAGTA

Orangutan -------AAA GAAGATCTGG ATATTGTATG TGAAAGGTTC ACTACTAGTA

Rat AATCAGGAAG GAAGATTTGG ATATTGTGTG TGAGAGGTTC ACTACAAGTA

Guinea_Pig AATCAGGAAA GAAGATCTGG ATATTGTATG CGAAAGGTTC ACAACTAGTA

Mouse AATCAGGAAG GAAGATCTGG ATATTGTGTG TGAGAGGTTC ACTACGAGTA

Rabbit AATCAGGAAG GAAGATCTGG ATATTGTGTG TGAGAGGTTC ACTACAAGTA

Dog GATTAGGAAA GAAGATCTGG ATATTGTGTG TGAGAGATTC ACTACAAGTA

Elephant GATCAGGAAG GAAGATCTGG ACATTGTGTG TGAGAGGTTC ACTACAAGTA

Cow GATCAGGAAA GAAGATCTTG AGATTGTGTG TGAGAGGTTC ACCACAAGTA

Horse GATCAGGAAA GAAGATCTGG ATATTGTGTG TGAGAGGTTC ACTACAAGTA

Opossum GATCCGGAGG GAAGACCTGG ACATCGTGTG CGAGCGCTTC ACCACCAGCA

Platypus AATCAGGAAA GACGACTTGG CCATCGTGTG TGAAAGGTTC ACCACAAGCA

Frog GATTAGGAAG GAAGACTTAG ACATAGTGTG TGAGAGATTT ACTACAAGCA

Chicken TATCAGGAAG GAAGATCTGC ACATTGTTTG TGAGAGATTT ACTACCAGTA

Zebra_Finch TATCAGAAAA GAAGATCTCG ACATTGTATG TGAGAGATTC ACTACAAGTA

Fugu CATTAGGAAG GAAGATATGG AAATAATCTG TGAAAGGTTC ACCACCAGCA

Zebrafish AATTAGAAAA GATGATATGG AAATAGTCTG TGAGCGTTTT ACGACCAGCA

Human AACTGCAGTC CTTTGAGGAT TTAGCCAGTA TTTCTACCTA TGGCTTTCGA

Chimpanzee AACTGCAGTC CTTTGAGGAT TTAGCCAGTA TTTCTACCTA TGGCTTTCGA

Gorilla AACTGCAGTC CTTTGAGGAT TTAGCCAGTA TTTCTACCTA TGGCTTTCGA

Orangutan AACTGCAGTC CTTTGAGGAT TTAGCCAGTA TTTCTACCTA TGGCTTTCGA

Rat AACTGCAGAC CTTTGAGGAT TTAGCTAGTA TTTCCACCTA TGGCTTTCGT

Guinea_Pig AACTGCAGTC CTTTGAGGAC TTAGCTAGTA TTTCTACTTA CGGCTTTCGG

Mouse AACTGCAGAC TTTTGAGGAT TTAGCCAGTA TTTCTACCTA TGGCTTTCGT

Rabbit AGCTGCAGTC CTTTGAGGAT TTAGCCAGCA TCTCTACCTA CGGCTTTCGG

Dog AACTGCAGTC CTTTGAGGAT TTAGCTAGTA TTTCTACCTA TGGCTTTCGA

Elephant AACTGCAGTC CTTTGAGGAT TTAGCCCGTA TTTCTACCTA TGGCTTCCGG

Cow AACTGCAGTC CTTTGAGGAT TTAGCTCATA TTTCTACCTA TGGCTTTCGG

Horse AACTGCAGTC TTTTGAGGAT TTAGCTAAGA TTTCTACCTA TGGCTTTCGG

Opossum AGCTACGGGC CTTCGAGGAC CTGGCCAGCA TCGCCACCTA CGGCTTCCGG

Platypus AGCTGCAGTC TTTCGAAGAC CTGGCTAGTA TTTCTACCTA TGGCTTCAGG

Frog AGCTACAGTC ATTTGAAGAT TTGTCCAATA TTTCCACTTA CGGATTTCGA

Chicken AACTGCAGAA ATTTGAAGAC TTGGCTAGTA TTTCAACGTA TGGTTTTAGG

Zebra_Finch AACTGCAAAA ATTTGAAGAC TTGGCTAGTA TTTCTACATA TGGTTTTAGG

Fugu AACTGCAGAC CTTTGAGGAC CTCTCAGCTA TTGCAACCTA TGGATTTAGA

Zebrafish AGCTGCAGTC TTTTGAAGAT TTGTCATCCA TCGCAACATA CGGATTCAGA

Human GGTGAGGCTT TGGCCAGCAT AAGCCATGTG GCTCATGTTA CTATTACAAC

Chimpanzee GGTGAGGCTT TGGCCAGCAT AAGCCATGTG GCTCATGTTA CTATTACAAC

Gorilla GGTGAGGCTT TGGCCAGCAT AAGCCATGTG GCTCATGTTA CTATTACAAC

Orangutan GGTGAGGCTT TGGCCAGCAT AAGCCATGTG GCTCATGTTA CTATTACAAC

Rat GGTGAGGCTT TGGCAAGCAT AAGCCATGTG GCCCATGTCA CTATTACAAC

Guinea_Pig GGCGAGGCTT TGGCCAGCAT AAGTCACGTG GCTCATGTTA CTATTACAAC

Mouse GGTGAGGCAT TGGCAAGCAT AAGCCATGTG GCCCATGTCA CTATTACAAC

Rabbit GGCGAGGCTT TGGCCAGCAT AAGCCACGTG GCTCATGTTA CTATTACCAC

Dog GGTGAGGCTT TGGCTAGCAT CAGCCATGTG GCGCATGTTA CTATTACAAC

Elephant GGCGAGGCTT TGGCCAGCAT CAGCCACGTG GCTCACGTTA CTATTACGAC

Cow GGTGAGGCTC TGGCCAGCAT AAGCCACGTG GCTCACGTCA CCATTACAAC

Horse GGTGAGGCTT TGGCCAGCAT AAGCCACGTG GCTCATGTTA CTATTACAAC

Opossum GGGGAGGCCC TGGCCAGCAT TAGCCACGTG GCCCACGTGA CCGTGACCAG

Platypus GGCGAGGCCC TGGCCAGCAT AAGTCACGTG GCCCACGTGA CGGTGACCAC

Frog GGAGAAGCAT TGGCCAGCAT AAGTCACGTG GCTCATGTTA CAATAACCAC

Chicken GGTGAGGCAT TGGCTAGCAT CAGTCATGTT GCCCATGTTA CGGTAACAAC

Zebra_Finch GGCGAGGCCT TGGCTAGCAT CAGTCATGTT GCCCACGTTA CAGTAACAAC

Fugu GGAGAGGCCC TTGCTAGTAT AAGTCATGTT GCGCATGTGA CCATTACAAC

Zebrafish GGAGAAGCCC TCGCCAGTAT AAGTCATGTA GCTCACGTCA CCATCACCAC

Human GAAAACAGCT GATGGAAAGT GTGCATACAG AGCAAGTTAC TCAGATGGAA

Chimpanzee GAAAACAGCT GATGGAAAGT GTGCATACAG AGCAAGTTAC TCAGATGGAA

Gorilla GAAAACAGCT GATGGAAAGT GTGCATACAG AGCAAGTTAC TCAGATGGAA

Orangutan GAAAACAGCT GATGGAAAGT GTGCGTACAG AGCAAGTTAC TCAGATGGAA

Rat CAAAACAGCT GATGGGAAAT GTGCATACAG AGCAAGTTAC TCAGATGGAA

Guinea_Pig CAAAACAACT GATGGAAAGT GTGCATACAG AGCAAGTTAC TCAGATGGAA

Mouse CAAAACAGCT GATGGGAAAT GTGCGTACAG AGCAAGTTAC TCAGATGGAA

Rabbit CAAGACCAGT GATGGAAAGT GTGCATACAG AGCGAGTTAC TCAGATGGAA

Dog CAAAACAGCT GAGGCAAAAT GTGCATACAG AGCAACTTAT TCGGATGGAA

Elephant CAAAACAGCC GATGGAAAAT GTGCGTTCAG GGCAAATTAC TCAGATGGAA

Cow CAAAACGGCT GACGGGAAGT GTGCATACAG AGCTCATTAC TCAGATGGAA

Horse TAAAACAGCT GATGGAAAGT GTGCATACAG AGCAAATTAC TCAGATGGAA

Opossum CAAGACTGCG GAGGCCAAGT GCGCCTACAG AGCAAGCTAC TCAGATGGGA

Platypus GAAAACGGCG GATGGCAAAT GTGCCTACAG AGCCAGTTAT TCCGACGGCA

Frog AAAAACAGCA GATGGAAAAT GTGCATACAG AGCAAGCTAT GCAGATGGCA

Chicken TAAAACAGCT GATGCAAAGT GTGCATACAG AGCTTCTTAC AGTGATGGAA

Zebra_Finch TAAAACAGCT GATGCAAAGT GTGCATTCAG AGCTACTTAC AGTGATGGAA

Fugu AAAAACAGCT GATGCCAAAT GTGCCTACAG GGCCAGCTAC ACCGATGGAA

Zebrafish AAAAACAGCA GATGCTAAAT GTGCTTACAG GGCCAATTAC TGTGATGGGA

Human AACTGAAAGC CCCTCCTAAA CCATGTGCTG GCAATCAAGG GACCCAGATC

Chimpanzee AACTGAAAGC CCCTCCTAAA CCATGTGCTG GCAATCAAGG GACCCAGATC

Gorilla AACTGAAAGC CCCTCCTAAA CCATGTGCTG GCAATCAAGG GACCCAGATC

Orangutan AACTGAAAGC TCCTCCTAAA CCATGTGCTG GCAATCAAGG GACCCAGATC

Rat AGCTACAAGC CCCTCCTAAA CCATGTGCAG GCAACCAGGG TACCCTGATC

Guinea_Pig AGCTGAAAGC TCCTCCTAAG CCATGTGCAG GCAATCAGGG GACCCAAATC

Mouse AGCTGCAAGC CCCTCCTAAA CCCTGTGCAG GCAACCAGGG CACCCTGATC

Rabbit AGCTGAAAGC TCCTCCTAAG CCATGTGCCG GCAATCAGGG GACGCAGATC

Dog AGCTGAAAGC TCCTCCTAAA CCGTGTGCAG GCAATCAGGG GACCCAGATC

Elephant AGCTGAAGGC GCCTCCTAAC CCGTGTGCGG GCAATCAGGG GACCCAGATC

Cow AGCTGAAAGC ACCTCCTAAA CCGTGTGCAG GCAACCAAGG GACCCAGATC

Horse AGCTGAAAGC TCCTCCTAAA CCATGTGCAG GCAATCAAGG GACGCAGATC

Opossum AGCTCAAGGC ACCTCCTAAG CCATGTGCGG GGAACCAGGG GACGCAGATC

Platypus AACTGAAGTC GCCCCCGAGA CCTTGCGCCG GGAACCAGGG GACCCTGATC

Frog AGCTAAAAAC ACCTCCAAAA CCTTGTGCTG GAAACCAAGG AACCCAGATT

Chicken AAATCAAAGC CCCCCCAAAA CCCTGTGCTG GAAACCAAGG GACACAGATC

Zebra_Finch AAATCAAAGC CCCTCCAAAG CCCTGTGCTG GAAACCAAGG AACTCAGATC

Fugu AACTGAAAGG ACCACCCAAA CCTTGTGCTG GCAATCAGGG AACTCAGATT

Zebrafish AACTAAAATC TCCACCCAAA CCCTGTGCTG GAAACCAGGG GACACTGATT

Human ACGGTGGAGG ACCTTTTTTA CAACATAGCC ACGAGGAGAA AAGCTTTAAA

Chimpanzee ACGGTGGAGG ACCTTTTTTA CAACATAGCC ACGAGGAGAA AAGCTTTAAA

Gorilla ACGGTGGAGG ACCTTTTTTA CAACATAGCC ACGAGGAGAA AAGCTTTAAA

Orangutan ACGGTGGAGG ACCTTTTTTA CAACATAGCC ACGAGGAGAA AAGCTTTAAA

Rat ACGGTGGAAG ACCTTTTTTA CAACATAATC ACAAGGAGGA AAGCTTTAAA

Guinea_Pig ACGGTGGAGG ACCTTTTTTA TAACATAACC ACAAGAAGAA AAGCTTTAAA

Mouse ACGGTGGAAG ACCTTTTTTA CAACATAATC ACAAGGAGGA AAGCTTTAAA

Rabbit ACGGTGGAGG ACCTTTTTTA CAACATAGCC ACAAGGAGGA AAGCTTTAAA

Dog ACGGTGGAGG ACCTTTTTTA CAACATATCC ACGAGGAGGA AAGCTTTAAA

Elephant GTGGTGGAGG ACCTTTTTTA TAACATAGCC ACGAGGAGGA AAGCGTTAAA

Cow ACGGTGGAAG ACCTTTTTTA CAACATATCC ACGAGGAGGA AAGCTTTAAA

Horse ACGGTGGAGG ACCTTTTTTA CAACATATCC ACGAGGAGGA AAGCTTTAAA

Opossum ACGGTGGAAG ACCTTTTTTA CAACGTCGCC ACAAGGAGGA AAGCTTTAAA

Platypus ACGGTCGAGG ACCTGTTCTA CAACGTAGGC ACCAGGAGGA AAGCGTTAAA

Frog TCGGTTGAAG ATCTTTTTTA TAATATTTCA ACAAGGCGAA AAGCTCTAAA

Chicken ATGGTGGAAG ATCTTTTTTA CAATGTAAAT ACAAGGAGGA AAGCTTTAAA

Zebra_Finch ACAGTTGAAG ATCTTTTTTA CAATGTAAAT ACAAGGAGGA AGGCTTTAAA

Fugu CTTGTGGAGG ATCTTTTCTA TAATGTGTCC ACAAGGAGAA AAGCTTTAAA

Zebrafish TCTGTAGAGG ATCTGTTTTA TAATGTGTCT ACGCGACGGA AAGCCCTAAA

Human AAATCCAAGT GAAGAATATG GGAAAATTTT GGAAGTTGTT GGCTATTCAG

Chimpanzee AAATCCAAGT GAAGAATATG GGAAAATTTT GGAAGTTGTT GGCTATTCAA

Gorilla AAATCCAAGT GAAGAATATG GGAAAATTTT GGAAGTTGTT GGCTATTCCA

Orangutan AAATCCAAAT GAAGAATATG GGAAAATTTT GGAAGTTGTT GGCTATTCAA

Rat AAATCCCAGT GAAGAGTACG GGAAGATTTT GGAAGTTGTT GGCTACTCAA

Guinea_Pig AAATCCAAGT GAAGAGTATG GGAAAATTTT GGAAGTTGTT GGCTATTCAG

Mouse AAATCCAAGT GAAGAGTACG GAAAAATTTT GGAAGTTGTT GGCTATTCAA

Rabbit AAATCCAAGC GAAGAGTATG GGAAGATTTT GGAAGTTGTT GGCTATTCAA

Dog AAATCCAAGT GAAGAGTATG GGAAAATTTT GGAAGTTGTT GGCTATTCAA

Elephant AAATCCAAGT GAAGAGTATG GGAAGATTTT GGAAGTTGTT GGCTATTCAA

Cow AAACCCAAGT GAAGAGTATG GAAAAATTTT GGAAGTTGTT GGCTATGCAG

Horse AAATCCAAGT GAAGAGTATG GGAAAATTTT GGAAGTTGTT GGCTATTCAA

Opossum GAACCCCAGC GAGGAGTACG GGAAGATCTT GGACGTGGTG GGCTATTCTG

Platypus AAATCCCAGC GAAGAGTACG GGAAGATCCT GGACGTCGTC GGCTACTCCG

Frog GAGTCCAAGT GAAGAGCATG CAAGAATAGT AGAAGTTGTC AGCTATGCTA

Chicken AAATCCGAGT GAAGAGTATG CAAAAATACT AGAAGTTGTT GGCTATGCCA

Zebra_Finch GAACCCAAAT GAAGAATATG CAAAAATACT AGAAGTTGTT AGCTATGCCA

Fugu AAGCCCCACT GATGAGTACT CGAGGATTGT AGATGTTGTC AGTTATGCCA

Zebrafish GAGCCCCAGT GAGGAATATT CCAGAATCGT CGAGGTTGTG AGCTACGCCA

Human TACACAATGC AGGCATTAGT TTCTCAGTTA AAAAACAAGG AGAGACAGTA

Chimpanzee TACACAATGC AGGCATTAGT TTCTCAGTTA AAAAACAAGG AGAGACAGTA

Gorilla TACACAATGC AGGCATTAGT TTCTCAGTTA AAAAACAAGG AGAGACAGTA

Orangutan TACACAATGC AGGCATTAGT TTCACAGTTA AAAAACAAGG AGAGACAGTA

Rat TACACAATTC AGGAATTAGT TTCTCAGTTA AAAAACAAGG CGAGACAGTC

Guinea_Pig TACATAACTC AGGCATTAGT TTCTCAGTTA AGAAACAAGG TGAGACGGTT

Mouse TACACAATTC AGGCATTAGT TTCTCAGTTA AAAAACAAGG TGAGACAGTA

Rabbit TACACAATTC AGGCATTAGT TTTTCGGTTA AAAAACAAGG AGAGACCGTG

Dog TACACAATTC AGGCATTAGT TTCTCAGTTA AAAAACAAGG CGAGACCGTA

Elephant TACACAATTC AGGCATTAGT TTCTCAGTTA AAAAACACGG TGAGACGGCG

Cow TACACAATTC GGGCATTGGT TTCTCAGTTA AAAAACAAGG TGAAACTGTA

Horse TACACAATTC AGGCATTAGT TTCTCAGTTA AAAAACAGGG TGAGACAGTA

Opossum TCCACAACTC GGGTGTCAGC TTCTCCGTGA AAAAGCAAGG AGAAACGGTG

Platypus TACACAACTC GGGCATCAGT TTCTTAGTGA GAAAACAAGG GGAGACGGCG

Frog TCCATAACTC TGGAATTAGT TTTTCAGTCA AAAAGCAAGG TGAAACTATG

Chicken TCCATAACTC AGGTATCAGC TTTTCAGTTA AAAAGCAAGG CGATACTGTG

Zebra_Finch TCCATAACTC AGGCATCAGC TTTTCAGTTA AAAAGCAAGG TGACACTGTG

Fugu TCCATAACTC AGGAAAGAGT TTTTCTGTCA AAAAGCAAGG AGAGACTGTA

Zebrafish TTCACAACTC TGGGAAAAGT TTTTCTGTCA AGAAGCAAGG TGAGATGGTT

Human GCTGATGTTA GGACACTACC CAATGCCTCA ACCGTGGACA ATATTCGCTC

Chimpanzee GCTGATGTTA GGACACTACC CAATGCCTCA ACCGTGGACA ATATTCGCTC

Gorilla GCTGATGTTA GGACACTACC CAATGCCTCA ACCGTGGACA ATATTCGCTC

Orangutan GCCGATGTTA GGACACTACC CAATGCCTCA ACCGTGGACA ATATTCGCTC

Rat TCTGATGTCA GAACACTACC CAATGCCACG ACCGTAGACA ACATTCGCTC

Guinea_Pig GCTGACATCA GAACATTATC CGGTGCCACA GTTGTAGACA ATATTCGCTC

Mouse TCTGATGTCA GAACACTGCC CAATGCCACA ACCGTGGACA ACATTCGCTC

Rabbit GCTGATGTCC GAACGTTACC TGGCGCCACA ACTGTGGACA ATATTCGCTC

Dog GCCGATGTTA GGACACTGCC CAATGCCACA ACGGTGGACA ATATTCGCTC

Elephant GCTGACGTTA GAACGCTGTC CAACGCCACA ACTGTGGACA ACATTCGCTC

Cow GCTGACGTCA GGACCCTGCC AAATGCCACC ACCGTGGACA ACATTCGCTC

Horse GCTGACGTTA GAACACTGCC CAATGCCACA ACCGTGGACA ATATTCGCTC

Opossum CCTGACATCA GAACCTTGAC AAATGCCACG GTGGTGGACA ACATCCGATC

Platypus GCCGACATCC GCACCCTGCC CGACGCCACC ACGGTGGACA ACATCCGCTC

Frog GCAGATGTGA GAACATTATC TAATGCAACT ACAGTAGACA ATATCAGAAC

Chicken TCAGATGTTA GAACTTTATC AAACGCTACC ACAGTGGACA ACATTAGGTC

Zebra_Finch TCGGATGTTA GAACTTTATC CAATGCCTCA ACAGTGGACA ACATCAGGGC

Fugu GCAGATGTGA GGACCTTAGC CAATGCATCT GTGGTGGATA ATATCCGAGG

Zebrafish GCAGATGTGA AGACTCTCCC AAACGCCTCT GTGCTGGACA ACATCCGTGT

Human CATCTTTGGA AATGCTGTTA GTCGAGAACT GATAGAAATT GGATGTGAGG

Chimpanzee CATCTTTGGA AATGCTGTTA GTCGAGAACT GATAGAAATT GGATGTGAGG

Gorilla CATCTTTGGA AATGCTGTTA GTCGAGAACT GATAGAAATT GGATGTGAGG

Orangutan CATCTTTGGA AATGCTGTTA GTCGAGAACT GATAGAAATT GGATGTGAGG

Rat CATCTTTGGA AATGCTGTTA GTCGAGAACT GATCGAAGTT GGGTGTGAGG

Guinea_Pig CATCTTTGGA AATGCTGTTA GTCGAGAATT GATAGAAGTT GGATGTGAGG

Mouse CATCTTTGGA AATGCGGTTA GTCGAGAACT GATAGAAGTT GGGTGTGAGG

Rabbit CATCTTTGGA AACGCTGTTA GTCGAGAGCT GATAGAAGTT GGGTGTGAGG

Dog CATCTTTGGA AATGCTGTCA GCCGAGAACT GATAGAAGTT GGGTGTGAGG

Elephant CATCTTTGGA AATGCTGTTA GTCGGTATGA CGTGTCATCT GGGTGCAAGA

Cow CATCTTTGGA AATGCTGTTA GCCGAGAATT GATAGAAGTT GAATGTGAGG

Horse CATCTTTGGA AATGCCGTTA GCCGAGAATT GATAGAAGTT GGGTGTGAGG

Opossum CATCTTTGGA AATGCCGTGA GCAGAGAACT GATCGAAGTG GGCTGCGATG

Platypus CGTCTTCGGA TGCGTCGTCA GCCGGGTCCC CGTGAGCGTG GGTGCGGGGG

Frog TGTTTTTGGA AATGCAGTGA GCAGGGAACT CATAGAAGTA GGATGTGAAG

Chicken CATCTTTGGA AATGCTGTTA GCAGGGAACT CATAGAAGTG GGCTGTGAAG

Zebra_Finch CATCTTTGGA AATGCTGTTA GCAGGGAACT CATAGAAGTG GGCTGCGAAG

Fugu AGTTTTCGGC AATGCTGTCA GCAGGGAGCT GATTGAAGTT GCCTGTGAGG

Zebrafish GGTGTTTGGA GTCGCAGTTA GCAGGGAGCT GATTGAAGTT GAATGCGAGG

Human ATAAAACCCT AGCCTTCAAA ATGAATGGTT ACATATCCAA TGCAAACTAC

Chimpanzee ATAAAACCCT AGCCTTCAAA ATGAATGGTT ACATATCCAA TGCAAACTAC

Gorilla ATAAAACCCT AGCCTTCAAA ATGAATGGTT ACATATCCAA TGCAAACTAC

Orangutan ATAAAACCCT AGCCTTCAAA ATGAATGGTT ACATATCCAA TGCAAACTAC

Rat ATAAAACCCT AGCTTTCAAA ATGAATGGCT ATATCTCGAA TGCAAACTAC

Guinea_Pig ATAAAACCCT GGCTTTCAAA ATGAATGGCT ACATATCTAA TGCAAACTAC

Mouse ATAAAACCCT AGCTTTCAAA ATGAATGGCT ATATATCGAA TGCAAACTAT

Rabbit ATCCAACCCT TGCTTTCAAA ATGAATGGCT ACATATCCAA CGCGAACTAC

Dog ATAAAACCCT AGCCTTCAAA ATGAATGGCT ACATATCCAA TGCAAATTAC

Elephant CAAAGTATTC AGTCTTTAAG CAAAATCAGT ACATATCCAA TGCAAACTAC

Cow ATAAAACCCT TGCCTTCAAA ATGAATGGCT ACATATCCAA TGCAAACTAT

Horse ATAAAACACT AGGCTTCAAA ATGAATGGCT ACATATCCAA TGCGAACTAC

Opossum ACCCGCTGCT CGCCTTCAGA ATGAAGGGCT TCATCTCCAA CGCCAACTAC

Platypus ACGGAGCGTT CTCCCCGGAG GTCCGGCGTT GGGAGCGAAG CCCAGGGCCG

Frog AGGAGAAACT GGCATTTAAA ATGAAAGGTT ACGTGACTAA TGCCAACTAT

Chicken ATGCAAATCT GGCCTTTAAA ATGAAAGGCT ACATCACGAA TGCCAACTAC

Zebra_Finch ATGCAAATCT GGCCTTTAAA ATGAAAGGTT ACATCACTAA TGCAAACTAC

Fugu ATCAGAAGCT CGCCTTCAAG ATGAAAGGCT ACGTCTCTAA TGCAAATTAT

Zebrafish ATCAGAAATT TGCTTTTAAA GTGAAGGGCT ACATCTCCAA TGCTAACTAC

Human TCAGTGAAGA AGTGCATCTT CTTACTCTTC ATCAACCATC GTCTGGTAGA

Chimpanzee TCAGTGAAGA AGTGCATCTT CTTACTCTTC ATCAACCATC GTCTGGTAGA

Gorilla TCAGTGAAGA AGTGCATCTT CTTACTCTTC ATCAACCATC GTCTGGTAGA

Orangutan TCAGTGAAGA AGTGCATCTT CTTACTCTTC ATCAACCATC GTCTGGTAGA

Rat TCAGTGAAGA AGTGCATTTT CCTACTCTTC ATCAACCACC GGCTGGTGGA

Guinea_Pig TCGGTGAAGA AGTGCATTTT CTTACTCTTC ATCAACCATC GTCTGGTAGA

Mouse TCAGTGAAGA AGTGCATTTT CCTACTCTTC ATCAACCACC GTCTGGTAGA

Rabbit TCAGTGAAGA AGTGTATCTT CTTGCTGTTC ATCAACCATC GCCTGGTAGA

Dog TCAGTGAAGA AGTGCATCTT CTTACTCTTC ATCAACCATC GTCTGGTAGA

Elephant TCGGTGAAGA AGTGCATCTT CTTACTCTTC ATCAACCATC GTCTGGTTGA

Cow TCAGTGAAGA AGTGCATCTT CTTACTCTTC ATCAACCATC GTCTGGTAGA

Horse TCAGTGAAGA AGTGCATCTT CTTACTCTTC ATCAACCATC GTCTGGTAGA

Opossum TCCGTGAAGA AGTGCATCTT CCTGCTCTTC ATCAACCACC GCCTGGTGGA

Platypus TCCCCGAGGC CGACTCCTCT TTCCGTCGGC CCCTCAGATC GGCTGGTGGA

Frog TCTATGAAAA AGTGCATTTT CTTACTCTTC ATTAATGCAA GACTAGTTGA

Chicken TCTGTGAAGA AATGTATATT TTTACTCTTC ATAAACCATC GGCTGGTAGA

Zebra_Finch TCTGTGAAGA AATGTATATT TTTACTCTTC ATAAACCATC GGTTGGTAGA

Fugu TCAGTGAAGA AATGCATCCT TATCCTCTTC ATTAACCATC GTCTGGTGGA

Zebrafish TCTGTCAAGA AATGCATCCT TATCCTTTTT ATCAATCATC GATTGGTGGA

Human ATCAACTTCC TTGAGAAAAG CCATAGAAAC AGTGTATGCA GCCTATTTGC

Chimpanzee ATCAACTTCC TTGAGAAAAG CCATAGAAAC AGTGTATGCA GCCTATTTGC

Gorilla ATCAACTTCC TTGAGAAAAG CCATAGAAAC AGTGTATGCA GCCTATTTGC

Orangutan ATCAACTTCC TTGAGAAAAG CCATAGAAAC AGTGTATGCA GCCTATTTGC

Rat ATCGGCTGCC CTGAAGAAAG CCATAGAAGC TGTATATGCA GCATATTTAC

Guinea_Pig ATCAACCTCA TTGAGAAAAG CCATAGAAAT GGTGTATGCA GCATATTTGC

Mouse ATCAGCTGCC TTGAGAAAAG CCATTGAAAC TGTATATGCA GCATACTTGC

Rabbit ATCGACCTCT TTGAAGAAAG CCATAGAAAC AGTGTATGCA GCGTATTTGC

Dog GTCAACTTCC TTAAGAAAAG CCATTGAAAC AGTATATGCA GCATATTTGC

Elephant ATCAGCCTCC TTGAGAAAAG CCATAGAAAC GGTTTACGCG GCATACCTGC

Cow GTCAGCTTCC TTAAGAAAAG CCATAGAAAC GGTGTATGCA GCGTATTTGC

Horse ATCGACTTCC TTGAGAAAAG CCATAGAAAC GGTGTATGCA GCATATTTGC

Opossum GTCCAGCGCG CTGAGGAAGG CCGTGGAGAG CGTGTATGCC GCGTACCTGC

Platypus TTCCACGGCG CTGAAGAGGG CTATCGAAAA CGTCTACGTG GCCTATCTGC

Frog ATCCACAGCA TTAAAGAAAG CAATAGAAAC TGTGTATGCT GCTTACTTAC

Chicken GTCAACTGCT TTGCGGAAAG CCATAGAGAC TGTGTATGCT GCTTATCTGC

Zebra_Finch GTCAGCCGCT CTGCGCAAAG CCATAGAAAC TGTGTACGCT GCTTATTTGC

Fugu GTCGAGCATG TTAAAGAAAG CCATCGAAAA CGTTTACGGC GCCTATCTTC

Zebrafish GTCTAGTGCC TTGAAGAAAG CAATTGAGAC CGTCTACACC GCTTATCTTC

Human CCAAAAACAC ACACCCATTC CTGTACCTCA GTTTAGAAAT CAGTCCCCAG

Chimpanzee CCAAAAACAC ACACCCATTC CTGTACCTCA GTTTAGAAAT CAGTCCCCAG

Gorilla CCAAAAACAC ACACCCATTC CTGTACCTCA GTTTAGAAAT CAGTCCCCAG

Orangutan CCAAAAACAC ACACCCATTC CTGTACCTCA GTTTAGAAAT CAGTCCCCAG

Rat CCAAAAACAC GCACCCATTC CTGTACCTCA GTTTGGAAAT CAGTCCTCAG

Guinea_Pig CCAAAAACAC ACACCCGTTC CTATACCTCA GTTTAGAAAT CAGCCCTCAG

Mouse CCAAAAACAC ACACCCATTC CTGTACCTCA GTTTGGAAAT CAGCCCTCAG

Rabbit CCAAAAACAC ACACCCGTTC CTGTACCTCA GTTTGGAAAT CAGCCCCCAG

Dog CCAAGAACAC ACACCCTTTC CTGTACCTCA GTTTAGAGAT CAGCCCCCAG

Elephant CCAAAAACAC ACACCCATTC CTGTACCTCA GTTTAGAAAT CAGCCCCCAG

Cow CCAAAAGCAC ACACCCGTTC CTGTACCTCA GTCTAGAAAT AAGCCCCCAG

Horse CCAAAAATAC ACACCCGTTC CTGTACCTCA GTTTAGAAAT CAGCCCCCAG

Opossum CCAAGAACTC GCACCCGTTT CTGTACCTGA GCCTGGAGAT CGCCCCCCAG

Platypus CCAAAAACAC CCACCCGTTC CTCTACCTCA GCCTGGAGAT CGCCCCCCAG

Frog CAAAAAATAC ACATCCTTTC CTGTACTTAA GCCTAGAAAT AGCTCCTCAA

Chicken CAAAAAGCAC TCATCCATTC TTATACTTAA GCCTGGAAAT AGCCCCTAAA

Zebra_Finch CAAAAAGCAC GCACCCATTC CTATACTTAA GCTTGGAAAT AGCCCCCCAG

Fugu CCAAGAACAC GCACCCCTTT CTTTACCTCA GTTTACAGAT CGCTCCTCAG

Zebrafish CCAAAAACAC TCATCCTTTT CTTTATCTCA GTTTAGAGAT TGCTCCTCAA

Human AATGTGGATG TTAATGTGCA CCCCACAAAG CATGAAGTTC ACTTCCTGCA

Chimpanzee AATGTGGATG TTAATGTGCA CCCCACAAAG CATGAAGTTC ACTTCCTGCA

Gorilla AATGTGGATG TTAATGTGCA CCCCACAAAG CATGAAGTTC ACTTCCTGCA

Orangutan AATGTGGATG TTAATGTGCA CCCCACAAAG CATGAAGTTC ACTTCCTGCA

Rat AACGTGGATG TCAATGTGCA CCCCACCAAG CATGAAGTTC ACTTTCTCCA

Guinea_Pig AATGTGGATG TCAATGTGCA CCCTACAAAA CATGAGGTTC ACTTTCTACA

Mouse AACGTGGACG TCAATGTACA CCCCACCAAG CACGAAGTTC ACTTTCTGCA

Rabbit AATGTGGATG TCAACGTGCA CCCTACAAAA CACGAAGTTC ACTTCCTGCA

Dog AACGTGGACG TCAATGTACA TCCCACGAAG CACGAAGTTC ACTTCCTGCA

Elephant AACGTGGATG TCAACGTGCA CCCAACGAAG CATGAGGTCC ACTTCCTGCA

Cow AATGTGGATG TCAACGTGCA CCCCACGAAG CACGAGGTTC ACTTCCTGCA

Horse AATGTGGATG TCAATGTGCA CCCCACAAAG CACGAAGTTC ACTTCCTGCA

Opossum AACGTGGATG TCAACGTGCA TCCCACCAAG CACGAGGTCC ACTTCCTCCA

Platypus AACGTGGACG TGAACGTCCA CCCGACCAAG CACGAGGTCC ATCTCCCC--

Frog AATGTTGATG TGAATGTGCA CCCTACAAAG CATGAGGTAC ATTTTCTCCA

Chicken AATGTAGATG TGAATGTGCA CCCTACAAAA CACGAGGTCC ACTTCCTTCA

Zebra_Finch AATGTAGATG TGAATGTGCA TCCTACAAAA CATGAGGTCC ATTTTCTTCA

Fugu AATATAGATG TCAATGTCCA TCCCACAAAG CACGAGGTCC ACTTTTTGCA

Zebrafish AACATTGATG TAAACGTTCA CCCGACAAAA CATGAGGTGC ACTTCCTGCA

Human CGAGGAGAGC ATCCTGGAGC GGGTGCAGCA GCACATCGAG AGCAAGCTCC

Chimpanzee CGAGGAGAGC ATCCTGGAGC GGGTGCAGCA GCACATCGAG AGCAAGCTCC

Gorilla CGAGGAGAGC ATCCTGGAGC GGGTGCAGCA GCACATCGAG AGCAAGCTCC

Orangutan CGAGGAGAGC ATCCTGGAGC GGGTGCAGCA GCACATCGAG AGCAAGCTCC

Rat TGAGGAGAGT ATTCTGGAGC GTGTGCAGCA GCACATCGAG AGCAAGCTAT

Guinea_Pig TGAGGAGAGC ATCTTGGAGC GGGTGCAGCA GCACATTGAG AGCAAGCTCT

Mouse CGAGGAGAGC ATTCTGCAGC GTGTGCAGCA GCACATTGAG AGCAAGCTGC

Rabbit CGAGGAGAGC ATCCTGGCGC GGGTGCAGCA GCACATTGAG AGCAAGCTCC

Dog CGAGGACAGC ATCCTGGAGC GGGTGCAGCA GCACATCGAG AGCAAGCTCT

Elephant CGAGGACAGC ATCCTGGAGC GAGTACAGCA GCACATCGAG AGCAAGCTTC

Cow TGAGGACAGC ATCCTGGAAC GGCTGCAGCA GCACATCGAG AGCAGGCTCC

Horse TGAGGACAGC ATCCTGGAGC GGGTGCAGCA GCACATTGAG AGCAAGCTCC

Opossum CGAGGACAGC ATCCTGGAGC GCGTGCAGCA GCACATCGAG GGCCGCCTCC

Platypus -GAGGACAGC ATCCTGGAGC GGGTGCAGCA GCACACCCTC TTGCTTCCGC

Frog TGAAGATAGC ATCATAGAGA GGGTGCAGCA GCACATTGAA AGTAAACTGC

Chicken TGAAGACAGC ATTCTAGAGC GTGTGCAACA ACATGTAGAG AGCAAGCTGC

Zebra_Finch TGAAGACAGT ATTCTGGAGC GTGTGCAACA ACATGTAGAG AGCAAGTTAT

Fugu TGAAGACAGT GTCATTGAGA GTGTTCAGAA ACACATCGAG AGCAAACTTC

Zebrafish CGAGGACTCC ATCATCGAGA GCATTCAGAA GCACATCGAG AGTAAACTCC

Human TGGGCTCCAA TTCCTCCAGG ATGTACTTCA CCCAGACTTT GCTACCAGGA

Chimpanzee TGGGCTCCAA TTCCTCCAGG ATGTACTTCA CCCAGACTTT GCTACCAGGA

Gorilla TGGGCTCCAA TTCCTCCAGG ATGTACTTCA CCCAGACTTT GCTACCAGGA

Orangutan TGGGCTCCAA TTCCTCCAGG ATGTACTTCA CCCAGACTTT GCTACCAGGA

Rat TGGGCTCCAA TTCATCCAGG ATGTATTTTA CCCAGACCTT GCTTCCAGGA

Guinea_Pig TGGGTGCCAA CTCTTCCAGG ATGTACTTCA CTCAGACTTT GCTACCAGGA

Mouse TGGGCTCCAA TTCCTCCAGG ATGTATTTCA CCCAGACCTT GCTTCCAGGA

Rabbit TGGGCTCCAA CTCCTCCCGC ATGTACTTCA CCCAGACTTT GCTGCCAGGA

Dog TGGGCTCCAA CTCCTCCAGG ATGTACTTCA CCCAGACTTT ACTACCAGGT

Elephant TGGGCTCCAA CTCCTCCAGG ACATACTTCA CCCAGACTTT GCTGCCAGGA

Cow TGGGCTCCAA CGCCTCCAGG ACCTACTTCA CCCAGACTTT ACTGCCAGGT

Horse TGGGCTCCAA CTCCTCCAGG ATGTACTTCA CCCAGACTTT ACTACCAGGT

Opossum TGGGCGCCAA TTCCTCTCGC ACCTACTTCA CGCAGACGCT GCTGCCGGGC

Platypus TC-------- ---------- ---------- --CAGACTCT GCTGCCCGGC

Frog TGGGATCAAA CTCTTCCAGA ATGTACTTCA CACAGCATTT TGTACAAGGA

Chicken TGGGTTCCAA TTCTTCAAGG ATGTACTTCA CTCAGACATT GCTTCCAGGG

Zebra_Finch TGGGCTCTAA TTCCTCAAGG ATGTACTTCA CTCAGACATT GCTTCCAGGA

Fugu TTGGCTCCAA TTCATCCCGT ACCTACTTCA CTCAGACATT GCTCCCAGGG

Zebrafish TCGGCTCCAA TTCCTCTCGC ACGTACTTCA CACAGACTCT ACTTCCGGGA

Human CTTGCTGGCC CCTCTGGGGA GATGGTTAAA TCCACAACAA GTCTGACCTC

Chimpanzee CTTGCTGGCC CCTCTGGGGA GATGGTTAAA TCCACAACAA GTCTGACCTC

Gorilla CTTGCTGGCC CCTCTGGGGA GATGGTTAAA TCCACAACAA GTCTGACCTC

Orangutan CTTGCTGGCC CCTCTGGGGA GATGGTTAAA TCCACAACAA GTCTGATCTC

Rat CTTGCTGGGC CCTCTGGGGA AGCCGTTAAA TCCACGACAG GCATAGCTTC

Guinea_Pig CTTGCTAGTT CCTCTGGGGA GGTAGTTAAA TCTACACCAG GTATGACCTC

Mouse CTTGCTGGGC CTTCTGGGGA GGCAGCTAGA CCCACGACAG GGGTGGCTTC

Rabbit CTTGCTGGCC CCTCTGGGGA CGTGATCAAA CCCGCAGCAG GAGTGGCCTC

Dog CTTGCTGGCC CCTCTGGAGA GGTGGTTAAA TCCACAACGG GTGCGACCCC

Elephant CCTGCTGGCC CCTCTGGGGA GGTGGTTAAG TCCACAGTCA GTGGGGCCCC

Cow CTTCCTGGCC CCTCTGGGGA AGCAGTTAAA TCCACAGCGA GTGTGACATC

Horse CTTCCTGGCC CCTCTGGGGA TTCGGTTAAA TCCACAGGGG GTGTGATCCC

Opossum ATGGCCGGTC CCGTGCCCGA AGGGGCGAAG TCTGCGGTCG GGGCCTCGGC

Platypus CTCGCCGACC CTTCCGGGGA GGAGGCCACG CAGTCGTCCG CT---GGCGG

Frog TACACTGGCA GGGACGGAAA AAAAATAAAA AGAAACAAGA ATAACACAGT

Chicken GCCGAATGTT CTTCCAGTGA GGTGGTAAAA TCAGCAGCA- --AGCTCTTC

Zebra_Finch GCTGACTGCT CTTCCAATGA GGTAGTAAAA TCAGCAGCA- --AACTCTTC

Fugu CTATCGGTCT CAGGTGCCAG TGAAGTTAAG CCTTCC---- ----------

Zebrafish CTTTCAGCAT CT---GCAAG CGTGGCAAAA GCTTCT---- ----------

Human GTCTTCTACT TCTGGAAGTA GTGATAAGGT CTATGCCCAC CAGATGGTTC

Chimpanzee GTCTTCTACT TCTGGAAGTA GTGATAAGGT CTATGCCCAC CAGATGGTTC

Gorilla GTCTTCTACT TCTGGAAGTA GTGATAAGGT CTATGCCCAC CAGATGGTTC

Orangutan CTCTTCTACT TCTGGAAGTA GTGATAAGGT CTATGCCCAC CAGATGGTTC

Rat CTCATCTACT AGTGGAAGTG GCGACAAGGT TCACGCTTAC CAGATGGTCC

Guinea_Pig CTCATCTGCT TCTGGAAACG GTGACAAGGT CTATGCTTAC CAGATGGTTC

Mouse CTCATCCACT AGTGGAAGTG GCGACAAGGT CTACGCTTAC CAGATGGTCC

Rabbit TGCCTCTGCC TCCGGAAGTG GTGACAGGGT CTACGCCCAC CACATGGTCC

Dog C---TCATCT ACTGGAAGTG GCGACAAGGT CTATGCCCAC CAGATGGTCC

Elephant TTCTTCTGCT TCTGGAAGTG GAGACAAGGT CTACGCCCAC CAGATGGTTC

Cow CTCATCCACT GCTGGAAGTG GTGACAGAGT GTATGCCCAC CAGATGGTCC

Horse CTCATCCTCT TCTGGAAGTG GCGACAAGAT TTATGCCCAC CAAATGGTCC

Opossum GCCGCCTCCG GGGCCCAGC- --GACAGGGT CTCAGCCCAG CACATGGTCC

Platypus TCCCCCCTCC CAGGGCCCCG GCGGCAAGGT GTCCGCCCAC CGGACGGTCC

Frog AACATCCCAG AAGAAGTCCA GTGAAAAGGT TTATGCTCGC CAGATGGTAC

Chicken TACGGCTGCA AAGGGAACCA GTGATAAAGT TTATGCACAC CAAATGGTCC

Zebra_Finch TGTGGTTACC AAGGGAAACA GTGACAAAGT TTATGCGCAT CAGATGGTCC

Fugu -ATCGTGGCA TTGGAGTCCG CTGAGCGAGT CTACGCGCAT CAGATGGTGA

Zebrafish -AGTTCCTCA GCAGATTCCC AGGAGCGAGT TTACGCCCAT CAGATGGTCC

Human GTACAGATTC CCGGGAACAG AAGCTTGATG CATTTCTGCA GCCTCTGAGC

Chimpanzee GTACAGATTC CCGGGAACAG AAGCTTGATG CATTTCTGCA GCCTCTGAGC

Gorilla GTACAGATTC CCGGGAACAG AAGCTTGATG CATTTCTGCA GCCTCTGAGC

Orangutan GTACAGATTC CCGGGAACAG AAGCTTGATG CATTTCTGCA GCCTCTGAGC

Rat GTACAGACTC TCGGGACCAG AAGCTCGATG CCTTTATGCA GCCTGTAAGC

Guinea_Pig GTACAGATTC TCGAGATCAG AAGCTTGATG CCTTTCTGCA GCCCATGAGC

Mouse GTACGGACTC CCGGGAGCAG AAGCTTGACG CCTTTCTGCA GCCTGTAAGC

Rabbit GCACAGATTC GCGGGAACAG AAGCTCGATG CCTTCCTGCA GCCCGTGAGC

Dog GCACAGATTC ACGAGAACAG AAGCTCGATG CCTTTCTGCA GCCCATGAGC

Elephant GTACAGATTC CCGGGAACAG AAACTCGATG CCTTTCTGCA GCCTGTGAGC

Cow GCACAGACTG CCGGGAACAG AAGCTCGATG CCTTCCTGCA GCCTGTGAGC

Horse GCACAGATTC TCGGGAACAG AAGCTCGATG CCTTCTTGCA GCCTGTGAGC

Opossum GCACTGATTC CCGCGAGCAG AAGCTCGATG CCTTTCTGCA GCCCGTGAGC

Platypus GCACCGACGC CCGGGACCAG AAGCTCGAGG CCTTCCTGCT AGATAGACGG

Frog GTACAGACTC CAGGGAGCAG AAGTTGGATG CTTTTCTTCA GCCTGTGAAC

Chicken GCACTGATTC CCGAGAGCAG AAGTTGGATG CTTTTCTTCA GCCAGTGAAC

Zebra_Finch GCACTGATTC CCGAGAGCAG AAAATGGATG CTTTTCTTCA GCCAGTGAAC

Fugu GGACGGACTG TCGCGCGCAG AAGCTGGATG CTTTCCTCCA GCCAAAAGAA

Zebrafish GCACTGACAG TAAAGCCCAG AAGCTTGATG CATTTCTCCA GCCATCAGCC

Human AAACCCCTGT CCAGTCAGCC CCAGGCCATT GTCACAGAGG ATAAGACAGA

Chimpanzee AAACCCCTGT CCAGTCAGCC CCAGGCCATT GTCACAGAGG ATAAGACAGA

Gorilla AAACCCCTGT CCAGTCAGCC CCAGGCCATT GTCACAGAGG ATAAGACAGA

Orangutan AAACCCCTGT CCAGTCAGCC CGAGGCCATT GTCACAGAGG ATAAGACAGA

Rat AGGCGTCTGC CCAGCCAGCC CCAGGACCCT GTCCCTGGGA ACAGGACAGA

Guinea_Pig AAGGCCCTGC CCAGTCAGCC CCAGGCCCAT GACCCAGGGG CTAAGGTACA

Mouse AGCCTTGGGC CCAGCCAGCC CCAGGACCCT GTCCGAGGGG CCAGGACGGA

Rabbit CAGCCACTGT CGAGTCAGCC TCCGGCCACT GTCCCGGAGG GTCAGACAGA

Dog AAGGCTCTGT CCAGTCAGCT GCAGGCTGTT GTCCCAGAAG ACAGGACCGA

Elephant AAGGGTGTGT CCAGCTGGCC GCAGGCCCAA GTCCCAGAGG ACAACACAGA

Cow AAGGCCCTGT CCAGTCAGCC CCAGGCTGTC GTCCCAGAGC ACAGGACAGA

Horse AGGGTTCCGT CCAGTCAGCC TCAGGCCGTT GTCCCAGAGG ACAGGACAGA

Opossum AGGTCCTCGG TCGAGCGCCC CGAGGGT--- ---------- ----------

Platypus GCTCCTCTAC CCTCGCGCAC CCTCTCC--- ---CGGCCTG ACGAAACGCA

Frog AGACCTATTC TCTCAGACTG CCAAGCT--- ---CCTGACA ATGCTGAAGG

Chicken AACCCCCTGA GTGCAGGCCC CACCGAAGAG ACAACAGGAG ATAAAGTGGG

Zebra_Finch AACTCCCTAA GTTCAGGCCC CACTGAAGTG ACAACAGGGG TTAATGCAAG

Fugu AAGCAG---C TTCCTGAACC AGCCGGG--- ---CCAAGCT GCACAGAGGC

Zebrafish TCA------- --TCTTCATC AACTGCT--- ---GCTCAAA GAAAGACAGA

Human TATTTCTAGT GGCAGGGCTA GGCAGCAAGA TGAGGAGATG CTTGAACTCC

Chimpanzee TATTTCTAGT GGCAGGGCTA GGCAGCAAGA TGAGGAGATG CTTGAACTCC

Gorilla TATTTCTAGT GGCAGGGCTA GGCAGCAAGA TGAGGAGATG CTTGAACTCC

Orangutan TATTTCTAGT GGCAGGGCTA GGCAGCAAGA TGAGGAGATG CTTGAACTCC

Rat GGGCTCTCCT GAAAAAGCCA TGCAGAAGGA TCAGGAGATG TCTGAGCTCC

Guinea_Pig TGGTTCTAGT TGCAGTGCTG GGCAGCAGGA TGAGGAGATG CTTCAAATCC

Mouse GGGATCTCCT GAAAGGGCCA CGCGGGAGGA CGAGGAGATG CTTGCTCTCC

Rabbit CGGTTCCAGT GGCGGGGCTG GGCAGCCAGA CGAGGAGATG CCGGAACTCC

Dog TGTTTCCAGT GGCAGGACTG GGCAGCAAGA TGAGGAAATG TTTGAACTCC

Elephant TGGTTCTAGT GGCAGGGCCA GGCAGCAAGA TGAGGAGATG CTCGAACTCC

Cow TGCTTCTAGC AGTGGGACCA GGCAGCAGGA CGAGGAGATG CTTGAACTCC

Horse TGCTTCTAGT GGTGGGGCCA GGCAGGAAGA TGAGGAAATG CTTGAACTCC

Opossum -CGGACGGCG GCTGGAGCCG GACAGCCGGA TGAGGAGATG GAGGAGCTGC

Platypus CGGTATCTCC GGGGGCGAGG AGAGGGCCGC GGGGCGTTTT GCCGTGGCGT

Frog TCAGAAAGAT AGCAGACTTC CCTCAGAGGA TACAGAGATG GCAGAAACAA

Chicken ACCTCCAGAG GGCACAGTTA GGCCACAGGA CGCTGAAATG GAAGACGTGA

Zebra_Finch ACCTCCAGAG GGTGCAGACA GACCCCAGGA TGCTGAAATG GAAGAAAAT-

Fugu TGCGGTTGAT CCC---GCCA AGTCTGACAG AGCTGACTTT GATGAAATGG

Zebrafish AAAAACCTCC AGC---ACGT CAACTGCTGT ACAAGACTTG GTGGAGCTGG

Human CAGCCCCTGC TGAAGTGGCT GCCAAAAATC AGAGCTTGGA GGGGGATACA

Chimpanzee CAGCCCCTGC TGAAGTGACT GCCAAAAATC AGAGCTTGGA GGGGGATACA

Gorilla CAGCCCCTGC TGAAGTGGCT GCCAAAAATC AGAGCTTGGA GGGGGATACA

Orangutan CAGCCCCTGC TGAAATGGCT GCCAAAAATC AGAGCTTGGA GGGGGATACA

Rat CGGCCGCCAT GGAAGCAGCT GCTGACAGTG CGAGTTTGGA GAGAGAATCA

Guinea_Pig CAGCCCCTGA GGTGGCAGCC ACCAAGAGTC AGAACGTGGA AGGAGATACA

Mouse CAGCCCCCGC TGAAGCAGCT GCTGAGAGTG AGAACTTGGA GAGGGAATCA

Rabbit CGGCCCCTGC TGAGGCAGCC GCTGAGACTC GGAGCTTGGA GGGGGATGTG

Dog CAGAGCCTGA TCAAGTGGCT GCCACAAATC AGGGCATGGA GGAGGAGACA

Elephant CAGCCCCTGC CCAAGTGGCT GCCGAGAACC AGGGAATGGA AGAGGAAGCA

Cow CAGCTCCTGC TGCAGTGGCT GCGAAGAGTC AGGCCTTAGA GGACGATGCA

Horse CAGACCCGGC GGAAGTGGCT GCCGTGAATC AAGGATCGAA GGGGGATGCA

Opossum CCGAGCCGGC CGCAGGCGCC CCAAGGACGC GCTCGCTCTG GCCACGTGGG

Platypus TTTCCCTGAC GGATCTTCCC CGATTCCCCC GCCCCCCGCC TTCACCGAGT

Frog GTGATCTGGA AATGCTTGAT GTTCTTGCTA CCGCCAAC-- ----------

Chicken GTGAGCTTCT TGAAACAGCT GATCTGATGG CTGACGTTCA GCAGGATGCA

Zebra_Finch -----CCAGT GAAGCCTGGG GGGCCAAGTG GATGCTTGTC TCCTGAGACT

Fugu ACACTGCTGA TCTGTTAGAG GCTGTTGAAC AGGGGGGAGA GGTAGTAATG

Zebrafish ACGACGCAGA GCTGCTGACA GCTGCTGTGG AGCCG----- -------TGT

Human ACAAAGGGGA CTTCAGAAAT GTCAGAGAAG AGAGGACCTA CTTCCAGC--

Chimpanzee ACAAAGGGGA CTTCAGAAAT GTCAGAGAAG AGAGGACCTA CTTCCAGC--

Gorilla ACAAAGGGGA CTTCAGAAAT GTCAGAGAAG AGAGGACCTA CTTCCAGC--

Orangutan ACAAAGGAGA CTTCAGAAAT GTCAGAGAAG AGAGGACCCA CTTCCAGC--

Rat GTGATTGGGG CTTCGGAAGT GGCACCCCCA GCGGCACCCC CTTCCAGTCC

Guinea_Pig AACAAGGGTG CTTTTGAAAC TACAGAAAAG AGAGCACCTG CTTCCAGCCC

Mouse CTAATGGAGA CTTCAGACGC AGCCCAGAAG GCGGCACCCA CTTCCAGTCC

Rabbit ACAAGGGGGA CTTTGGAAAC ATCAGAGAAG AGAGGACCCA GCTCCAGCCC

Dog ACAGAGGGGA CTTCGGAAAC ATCAGAGAAG AAAGGGCCCC CTTCCAGCCC

Elephant ACAAAGGAGC CTTCAGAAAC A---GAGAAG AGGGGGCCCA CCCTGAGCCC

Cow ACAATGAGGG CTGCAGACCT GGCCGAGAAG AGAGGGCCCT CTTCCAGCCC

Horse ACAAAGGAGA CTTCAGAGAC GTCAGAGAAG AGGGGGCCCC CTTCCAGCCC

Opossum CTGTTGGGCA GTTCTCCCTC CCCGTCCCAG TTCCCGAAGG CTTCGATTCC

Platypus ACCGGGGGAG GCTCCGAGCT CAGCGGAGCG ATGGGGCGGG GAGGCCATCG

Frog ------CCAG ACAATTCTGT GCTGAAACAA AAAGAAACTG ATGACTCCAC

Chicken GTGATGCCTG GAGGGCCGAG T--------- AAGAGCGGAC ACTTGTCTCC

Zebra_Finch GTGCTTTCTC GAGGCCACAG TCTTCTGAGG GAATCTGATT CCCTCTCCCC

Fugu GATGTCAAAG ACAGTAGTGC ACAAAGATTT ATGTCAGAGT TTAAATGCCC

Zebrafish GGCGGAAAGG ATCCTCAGAC TGATGCTCAA CCTCCTGGTG ATGAAGCACC

Human ----AACCCC AGAAAGAGA- --CATCGGGA AGATTCTGAT GTGGAAATGG

Chimpanzee ----AACCCC AGAAAGAGA- --CATCGGGA AGATTCTGAT GTGGAAATGG

Gorilla ----AACCCC AGAAAGAGA- --CATCGGGA AGATTCTGAT GTGGAAATGG

Orangutan ----AACCCC AGAAAGAGA- --CATCGGGA AGATTCTGAT GTGGAAATGG

Rat AGGCAGCTCC AGAAAGAGA- --CATCGGGA GGACTCCGAT GAGGAAATGA

Guinea_Pig AGGCAATCCC AGAAAGAGA- --CATCGGGA AGATTCTGAT GTGGAAATGA

Mouse AGGAAGCTCC AGAAAGAGA- --CATCGGGA GGACTCTGAT GTGGAAATGG

Rabbit AGGCCTCCCC AGAAAGAGAT GT---CGAGA AGATTCTGCT GTGGAAATGA

Dog AGGCAACCCC AGAAAGAGGC CT---CGGGA AAGCTCTGAT GTAGAAATGG

Elephant AGGTGTTCCC AGGAAGAGGC CT---CGGGG AGACTCTGAT GTGGAGATGT

Cow CGAGAACCCC AGAAAGAGAC CT---CGGGA AGACTCTGAT GTGGAAATGG

Horse GGGCAACCCC AGAAAAAGA- --CATCGAGA AGGCTCTGAT GTGGAAATGG

Opossum TGGGTCGCCC AGGCAGAAGA GGCCTCGGGA GGATTCTGAT GTGGAAATGA

Platypus GGGCGGA--- ---AAGAGGG CGCGGGAGGA TTCAGACGGA GAG---ACGG

Frog AAACATTGTA AGGAAACGAC CACGAAAAGA ATCTGATGTG ATG---GTTG

Chicken TGAGAAAGCG CGAAAGAGAC CACGGGAAGA CACAGACATA CAA---ATGG

Zebra_Finch A------GTA AAAAATGGAC CACGGGAAGA CATGGATGTA GAA---CTGG

Fugu AACTAGCAGC AGAAAACGAC CCCGGAATGA G--------- ---------C

Zebrafish ACCA------ AGGAAAAGGC CTCATGTTGA G--------- ---------G

Human TGGAAGATGA TTCCCGAAAG GAAATGACTG CAGCTTGTAC CCCCCGGAGA

Chimpanzee TGGAAGATGA TTCCCGAAAG GAAATGACTG CAGCTTGTAC CCCCCGGAGA

Gorilla TGGAAGATGA TTCCCGAAAG GAAATGACTG CAGCTTGTAC CCCCCGGAGA

Orangutan TGGAAGATGA TTCCCGAAAG GAAATGACTG CAGCTTGTAC CCCGCGGAGA

Rat TGGAAAATGA TTCCCGGAAG GAAATGACAG CTGCTTGCTA CCCCCGGAGG

Guinea_Pig TAGAAAGTGG TTCCCAGAAG GAAATGACAG CAGCCTGTAT CCCTCGGAGA

Mouse TGGAAAATGC TTCCGGGAAG GAAATGACAG CTGCTTGCTA CCCCAGGAGG

Rabbit TGGAAGATGA GTCCCGGAAG GAAATGACAG CAGCCTGCAC GCCCCGGAGG

Dog TGGAAGATGA TTCCCGAAAG GACATGACAG CAGCATGTAC CCCTCGACGG

Elephant TGGAAGACGA TTCTCAAAAG GAAATGACAG CAGCCTGCAC CCCGCGGCGA

Cow TGGAGGATGC ATCCCGAAAG GAGATGACAG CCGCTTGTAC CCCCCGGAGA

Horse TGGAAGGTGA TTCCAGAAAG GACATGACAG CAGCTTGTAC TCCCCGGAGA

Opossum GGGAGGAT-- ---------- GAGATGACGG CGGCCTGCTG CCCCCGGAGG

Platypus AGGAGGAGGA AGGCGGCCAG GAGATGACGG CGGGAGGCGC CCCTCGCCGG

Frog AGGAAGATGA GTCGGTAAAG TCTTTGACAG CTGCCTCCAT GCCAAGAAGA

Chicken AA---GAAGA CAACAGAAAG GAAATGACTG CTGCCTGCAC CCCTAAGAGA

Zebra_Finch AGAAAGATGA CACCAGAAAG GACATGACTG CTGCCTGCAC CCCTAGAAGA

Fugu AGAACAAGGA CGAGGATGAG GCGCTCACAG CTGCAGCCAC ACCCAAAAGA

Zebrafish AGGTGAAGGA GGAT------ ---TTGACAG CTGCTTCTCT TCCCAGGAGA

Human AGGATCATTA ACCTCACTAG TGTTTTGAGT CTCCAGGAAG AAATTAATGA

Chimpanzee AGGATCATTA ACCTCACTAG CGTTTTGAGT CTCCAGGAAG AAATTAATGA

Gorilla AGGATCATTA ACCTCACTAG TGTTTTAAGT CTCCAGGAAG AAATTAATGA

Orangutan AGGATCATTA ACCTCACTAG TGTTTTGAGT CTCCAGGAAG AAATTAATGA

Rat AGGATCATTA ACCTCACCAG TGTTTTGAGT CTCCAGGAAG AAATTAACGA

Guinea_Pig AGGATCATTA ACCTTACAAG TGTCCTGAGT CTCCAGGAAG AAATCAGTGA

Mouse AGGATCATTA ACCTCACCAG CGTCTTGAGT CTCCAGGAAG AGATTAGTGA

Rabbit AGAATCATTA ACCTCACCAG CGTTTTGAGT CTCCGTGAAG AAATTAATGA

Dog AGGATCATCA ACCTCACCAG TGTTCTGAGC CTCCAGGAAG AAATTAACGA

Elephant AGGATTATTA ACCTCACCAG CGTTTTGAAT CTCCAGGAAG AAATTAACGA

Cow AGGATCATCA ACCTTACCAG TGTTCTGAGT CTCCAGGAGG AGATCAACGA

Horse AGGATCATTA ACCTCACCAG TGTTCTGAGT CTCCAAGAAG AAATTAATGA

Opossum AGGGTGGTCA AGCTGACCAG CGTCCTGACG CTGCAGGATG AGATCAGCGA

Platypus AGGGTCATCA ACCTGACCAG CATCCTCACC CTCCGGGAAG AGATCGACGA

Frog AGAATTATAA ATTTGACAAG TATACTGAGT CTGCAGAAGA AGATTGAAGA

Chicken AGAATTATCA ATTTGACCAG TGTGTTGACT CTTCAGGAGG AAATTAGCAA

Zebra_Finch AGAATTATCA ACTTGACTAG TGTGTTGACT CTCCAGGAAG AAATTAATAA

Fugu CGAGTTATAA AACTGACCAG TATTAAAGAG CTCAGAGCTG AGATGTGTGA

Zebrafish CGAATCGTTA AACTGACCAG CATAAAGGAG CTTCGGGACC AGATTGAGCT

Human GCAGGGACAT GAGGTTCTCC GGGAGATGTT GCATAACCAC TCCTTCGTGG

Chimpanzee GCGGGGACAT GAGGTTCTCC GGGAGATGTT GCATAACCAC TCCTTCGTGG

Gorilla GCGGGGACAT GAGGTTCTCC GGGAGATGTT GCATAACCAC TCCTTCGTGG

Orangutan ACGGGGACAT GAGGTTCTCC GGGAGATGTT GCATAACCAC TCCTTCGTGG

Rat CCGGGGTCAT GAGACTCTCC GGGAGATGCT CCGTAACCAT ACCTTTGTGG

Guinea_Pig GCGGGGACAT GAGACTCTCC GGGAGATGTT CCAAAACCAC TCCTTTGTGG

Mouse GCGGTGCCAT GAGACTCTCC GGGAGATGCT CCGTAACCAT TCCTTTGTGG

Rabbit GCGGGGACAT GAGACTCTCC GGGAGATGCT GCGTGACTCC TCCTTCGTGG

Dog GCGGGGACAT GAGACACTCC GAGAGATGCT GCATAACCAC TCCTTTGTGG

Elephant GCGAGGGCAT GAGGCTCTCC GGGAGATGCT GTATAACCAC TCCTTTGTGG

Cow GCGGGGCCAT GAGACTCTCC GGGAGATGCT GCATAACCAC TCCTTTGTGG

Horse ACGGGGACAT GAGACTCTCC GGGAGATGCT GCATAACCAT TCCTTTGTGG

Opossum GAGAGGCCAT GAGGCTCTCC GGGAGATGCT GCGTGACCAC TCCTTCGTGG

Platypus GCGCGGCCAT CGGCCCCTCC GGGAGCTGCT GCACGGTCAC TCGTTCGTGG

Frog GTGTGGTCAC TCAAGTCTTC AGGACATGCT GCGCAATCAT TCCTTTGTTG

Chicken CCAATCACAT GAAAGTCTTC AGGAGATGCT GCGTGATCAC TCATTTGTTG

Zebra_Finch CCAGGCACAT GCAAAACTCC AGGAGATGCT ACATGAGCAC TCATTTGTTG

Fugu GAACACGCAC ACAGGGCTTC AGGAAATGCT GCAGAACCAC TCATTTGTGG

Zebrafish GCAAACTCAC AAAGGTTTAC AGGAGCTGCT GCAGAATCAC TCATTTGTAG

Human GCTGTGTGAA TCCTCAGTGG GCCTTGGCAC AGCATCAAAC CAAGTTATAC

Chimpanzee GCTGTGTGAA TCCTCAGTGG GCCTTGGCAC AGCATCAAAC CAAGTTATAC

Gorilla GCTGTGTGAA TCCTCAGTGG GCCTTGGCAC AGCATCAAAC CAAGTTATAC

Orangutan GCTGTGTGAA TCCTCAGTGG GCCTTGGCAC AGCATCAAAC CAAGTTATAC

Rat GCTGTGTGAA TCCTCAGTGG GCCTTGGCAC AGCACCAGAC CAAGCTCTAC

Guinea_Pig GCTGTGTGAA TCCTCAGTGG GCCTTGGCAC AGCATCAGAC CAAGTTATAC

Mouse GCTGTGTGAA TCCTCAGTGG GCCTTGGCAC AGCACCAGAC CAAGCTCTAC

Rabbit GCTGTGTGAA TCCTCAGTGG GCCCTGGCGC AGCATCAGAC CAAGCTGTAC

Dog GCTGCGTGAA TCCTCAGTGG GCCCTGGCTC AGCATCAGAC GAAGTTATAC

Elephant GCTGTGTGAA CCCTCAGTGG GCCCTGGCAC AGTATCAGAC CAAGCTATAC

Cow GCTGCGTGAA TCCTCAGTGG GCCTTGGCAC AGCATCAGAC CAAGTTATAC

Horse GTTGTGTGAA TCCTCAGTGG GCCTTGGCAC AGCATCAGAC CAAGTTATAC

Opossum GCTGCGTGAG CCCGCAGTGG GCGCTGGCCC AGTACCAGAC CCGGCTCTAC

Platypus GCTGCGTGAG TCCGCAGTGG GCGCTGGCCC AGTTCCAGAC CAAGCTGTAC

Frog GCTGTGTCAA TCCCCAGTGG GCTTTAGCTC AGTTTCAGAC AAAGTTATAT

Chicken GCTGTGTCAG CCCTCAGTGG GCTCTGGTGC AGCATCGGAC AAAACTGTAT

Zebra_Finch GTTGCGTCAG TCCTCAGTGG GCTCTGGCCC AGTACCAGAC AAAACTGTAC

Fugu GTTGCATCAA CCTCCAGTGG GCTCTGATCC AACATCGCAC TAAACTGTAC

Zebrafish GTTCAGTCAG TCCTCAGTGG ACTTTGGTGC AGCACCAGAC CAAACTCTAC

Human CTTCTCAACA CCACCAAGCT TAGTGAAGAA CTGTTCTACC AGATACTCAT

Chimpanzee CTTCTCAACA CCACCAAGCT TAGTGAAGAA CTGTTCTACC AGATACTCAT

Gorilla CTTCTCAACA CCACCAAGCT TAGTGAAGAA CTGTTCTACC AGATACTCAT

Orangutan CTTCTCAACA CCACCAAGCT TAGTGAAGAA CTGTTCTACC AGATACTCAT

Rat CTCCTCAACA CTACCAAGCT CAGTGAAGAA CTCTTCTACC AGATACTCAT

Guinea_Pig CTTCTGAACA CCACCAAGCT CAGTGAAGAA TTGTTCTACC AGGTGCTCAT

Mouse CTCCTCAACA CTACCAAGCT CAGTGAAGAG CTGTTCTACC AGATACTCAT

Rabbit CTTCTCAACA CCACCAAACT GAGTGAAGAG CTGTTCTACC AGATACTCAT

Dog CTTCTCAACA CCACCAAATT GAGTGAAGAA CTGTTCTATC AGATACTCAT

Elephant CTGCTCAACA CCACCAAGCT CAGTGAGGAA CTGTTCTACC AGATACTCAT

Cow CTTCTCAACA CCACCAGACT TAGTGAAGAA CTGTTCTACC AGATTCTCGT

Horse CTTCTCAACA CCACCAAACT TAGTGAAGAA CTGTTCTACC AGATACTCAT

Opossum CTCCTCAACA CCACGTGCCT CAGCGAAGAG CTCTTCTACC AGATCCTGAT

Platypus CTGCTCAACA CCACCAAACT CAGGGAGGGA GTGGGTGAGA GGCGTTGGCG

Frog CTTCTAAACA CCACTAAACT CAGCCAAGAA TTATTTTACC AAATACTCAT

Chicken CTTCTCAATA CAACAAAACT CAGCCAAGAA CTTTTCTACC AGATACTTAT

Zebra_Finch CTTCTCAATA CAACAAAACT CAGCCAAGAA CTCTTCTACC AGATACTTAT

Fugu CTGCTTAACA CCACCAATCT CAGCCAGGAG CTTTTCTACC AAATATTGAT

Zebrafish CTGCTCAATA CAACCAAACT CAGTCAGGAG CTGTTTTATC AAATTTTAGT

Human TTATGATTTT GCCAATTTTG GTGTTCTCAG GTTATCGGAG CCAGCACCGC

Chimpanzee TTATGATTTT GCCAATTTTG GTGTTCTCAG GTTATCGGAG CCAGCACCGC

Gorilla TTATGATTTT GCCAATTTTG GTGTTCTCAG GTTATCGGAG CCAGCACCGC

Orangutan TTATGATTTT GCCAATTTTG GTGTTCTGAG GTTATCGGAG CCAGCACCGC

Rat TTACGATTTT GCCAACTTCG GTGTTCTCAG GTTATCGGAA CCAGCACCAC

Guinea_Pig TTACGACTTT GCCAATTTTG GTGTTTTGAG GCTATCGGAG CCAGCACCAC

Mouse TTATGATTTT GCCAACTTTG GTGTTCTGAG GTTATCGGAA CCAGCGCCAC

Rabbit TTATGATTTT GCCAATTTTG GTGTTCTGAG ATTATCGGAG CCGGCTCCGC

Dog TTATGATTTT GCCAATTTTG GTGTTCTGAG GCTCTCGGAG CCTGCACCGC

Elephant TTATGACTTC GCCAATTTTG GTGTTCTGAG ATTATCGGAG CCAGCACCAC

Cow TTATGATTTT GCCAATTTTG GTGTTCTGAG GTTATCGGAG CCAGCACCAC

Horse TTATGATTTT GCCAATTTTG GTGTTCTGAG GTTATCGGAG CCAGCACCAC

Opossum CTGTGACTTC GCCAACTTCG GCATTCTGAG GCTCTCGGAG CCGGCGCCCC

Platypus CGAGGAGGAC CCGTCGGTCG GTCGGTTGGG CCGCCAGGAG CCGGCCCCAC

Frog TTATGACTTT GGAAACTTCG GCATAATGAA ATTATCTGAG CCAGCTCCAC

Chicken TTATGACTTT GCAAACTTTG GCGTTCTGAG ACTCTCTGAG CCAGCTCCTT

Zebra_Finch TTATGACTTT GCAAACTTTG GAGTCTTAAG GTTGTCTGAG CCAGCTCCTT

Fugu CTACGACTTT GGAAACTTTG GCGTTCTGAG ACTCTCTGAA CCTGCTCCGC

Zebrafish CTACGACTTT GGTAACTTCG GCGTTCTTCG ATTGTCGAAT CCAGCGCCGC

Human TCTTTGACCT TGCCATGCTT GCCTTAGATA GTCCAGAGAG TGGCTGGACA

Chimpanzee TCTTTGACCT CGCCATGCTT GCCTTAGATA GTCCAGAGAG TGGCTGGACA

Gorilla TCTTTGACCT CGCCATGCTT GCCTTAGATA GTCCAGAGAG TGGCTGGACA

Orangutan TCTTTGACCT AGCCATGCTT GCCTTAGATA GTCCAGAGAG TGGCTGGACA

Rat TCTTTGACCT CGCCATGCTG GCCTTAGACA GTCCTGAAAG TGGCTGGACA

Guinea_Pig TCTTTGACCT TGCCATGCTC GCCTTAGACA GTCCTGAGAG CGGCTGGACG

Mouse TCTTCGACCT GGCCATGCTG GCCTTAGACA GTCCTGAAAG TGGCTGGACA

Rabbit TCTTTGACCT CGCCATGCTG GCCTTGGACA GTCCTGAGAG TGGCTGGACG

Dog TGTTTGAGCT GGCCATGCTC GCCCTGGACA GTCCCGAGAG CGGCTGGACG

Elephant TCTTCGACCT CGCCATGCTG GCTTTAGACA GTCCCGAGAG CGGCTGGACA

Cow TCTTTGACCT GGCCATGCTC GCCTTAGACA GTCCCGAGAG TGGCTGGACA

Horse TCTTTGACCT CGCCATGCTC GCCTTAGATA GTCCCGAAAG TGGCTGGACA

Opossum TCTATGACCT GGCCATGCTC GCCCTGGACC ACCCCGAGAG CGGCTGGACG

Platypus TCCACACTCT GGCCATGCTG GCCTTGGACG GCGCGGAAAG CGGCTGGACG

Frog TGTATGACTT GGCAATGTTA GCCTTGGATA GTACAGAGAG TGGGTGGACA

Chicken TATATGAGCT TGCAATGCTT GCTTTAGAGG ACCCAGAGAG TGGCTGGACA

Zebra_Finch TATATGAGCT TTCAATGCTT GCTTTAGAGG ACCCTGAAAG TGGCTGGACG

Fugu TTTATGATCT GGCCATGCTG GCTCTGGACT CCGAAGGCAG TGGCTGGACA

Zebrafish TCTATGATCT GGCCATGTTG GCTCTGGATT CAGAGGAAAG TGGATGGACA

Human GAGGAAGATG GTCCCAAAGA AGGACTTGCT GAATACATTG TTGAGTTTCT

Chimpanzee GAGGAAGATG GTCCCAAAGA AGGACTTGCT GAATACATTG TTGAGTTTCT

Gorilla GAGGAAGATG GTCCCAAAGA AGGACTTGCT GAATACATTG TTGAGTTTCT

Orangutan GAGGAAGATG GTCCCAAAGA AGGACTTGCT GAATACATTG TTGAGTTTCT

Rat GAGGAAGATG GCCCAAAGGA AGGACTTGCA GAATACATTG TTGAGTTTTT

Guinea_Pig GAAGAAGATG GTCCCAAAGA AGGACTTGCT GAGTACATTG TTGAGTTTCT

Mouse GAGGACGACG GCCCGAAGGA AGGGCTTGCA GAGTACATTG TTGAGTTTCT

Rabbit GAGGAGGATG GCCCCAAGGA GGGGCTCGCC GAGTACATCG TGGAGTTCCT

Dog GAGGAGGATG GTCCCAAAGA AGGACTTGCT GAGTACATTG TTGAGTTTCT

Elephant GAGGAGGATG GCCCCAAAGA AGGACTTGCC GAATACATTG TTGAGTTTCT

Cow GAGGAGGATG GTCCCAAGGA AGGACTTGCT GAGTACATTG TTGAGTTTCT

Horse GAGGAAGATG GCCCCAAAGA AGGACTTGCC GAGTACATTG TTGAATTTCT

Opossum GAGGAAGACG GCCCCAAGGA AGGCCTGGCC GAGTACATCG TGGAGTTTCT

Platypus GAGGAAGACG GCCCCAAGGA AGGCCTGGCC GACTACATCG TGGAGTTCCT

Frog GAAGAAGATG GCCCTAAAGA AGGACTTGCT GAATATATTG TACAGTTTTT

Chicken GAAGAAGATG GCCCAAAAGA AGGTCTTGCT GAGTACATAG TTGAGTTTCT

Zebra_Finch GAAGAAGATG GCCCAAAAGA AGAGCTTGCT GAGTACATTG TGGAGTTTTT

Fugu GAAGAAGATG GGCCTAAAGA AGGTCTGGCC CAGTATATAG TGGATTTCCT

Zebrafish GAGGAGGATG GTCCGAAAGA AGGACTGGCC CAGTATATTG TGGATTTCCT

Human GAAGAAGAAG GCTGAGATGC TTGCAGACTA TTTCTCTTTG GAAATTGATG

Chimpanzee GAAGAAGAAG GCTGAGATGC TTGCAGACTA TTTCTCTTTG GAAATTGATG

Gorilla GAAGAAGAAG GCTGAGATGC TTGCAGACTA TTTCTCTTTG GAAATTGATG

Orangutan GAAGAAGAAG GCTGAGATGC TTGCAGACTA TTTCTCTTTG GAAATTGATG

Rat GAAGAAGAAA GCCGAGATGC TTGCAGACTA TTTCTCTGTG GAAATTGATG

Guinea_Pig GAAAAAGAAA GCGGAGATGC TGGCAGACTA TTTCTCTTTG GAAATTGATG

Mouse GAAGAAGAAA GCGGAGATGC TTGCAGACTA TTTCTCTGTG GAGATCGATG

Rabbit CAAGAAGAAG GCCGAGATGC TTGCAGACTA CTTTTCCCTG GAAATTGACG

Dog GAAGAAGAAG GCTGAGATGC TCGCAGACTA TTTCTCTCTG GAAATTGATG

Elephant GAAGAAGAAG GCTGAGATGC TTACAGACTA TTTCTCTCTG GAAATTGATA

Cow GAAAAAGAAG GCTGAGATGC TTGCAGACTA CTTCTCTCTG GAGATTGATG

Horse GAAAAAGAAG GCTGAGATGC TTGCAGACTA TTTCTCTTTG GAAATTGATG

Opossum GAAGAGGAAG GCCGAGATGC TGGCCGACTA CTTCTCCCTG GAAATCGATG

Platypus GAAGAAGAAG GCGGAGATGC TGGCGGACTA CTTCTCGCTG GAGATCGACG

Frog AAAGAAGAAA GCAGAGATGA TGGCAGACTA CTTCTCACTT GAAATTGACC

Chicken GAAAAAGAAG ACTGAAATGT TGAAAGATTA TTTCTCTCTT GAAATTGATG

Zebra_Finch GAAAAAGAAG ACTGAAATGT TGAAAGATTA TTTCTCTCTT GAAATTGATG

Fugu GAAGAGGAAA GCAGAGCTGC TGGGTGACTA CTTCTCCGTG GAGATTGACC

Zebrafish TAAGCAGAAA GCAGAAATGC TGGAAGAATA CTTTTCCTTG GAAATAGATG

Human AGGAAGGGAA CCTGATTGGA TTACCCCTTC TGATTGACAA CTATGTGCCC

Chimpanzee AGGAAGGGAA CCTGATTGGA TTACCCCTTC TGATTGACAA CTATGTGCCC

Gorilla AGGAAGGGAA CCTGATTGGA TTACCCCTTC TGATTGACAA CTATGTGCCC

Orangutan AGGTAGGGAA CTTGATTGGA TTACCCCTTC TGATTGACAA CTATGTGCCC

Rat AGGAAGGGAA CCTGATTGGA TTACCTCTCC TGATTGATAG CTATGTGCCG

Guinea_Pig AGGAAGGGAA CCTGAATGGA TTACCCCTTC TGATCGACAA CTATGTGCCC

Mouse AGGAAGGGAA CCTGATTGGA TTACCTCTTC TGATTGACAG CTATGTGCCA

Rabbit AGGAAGGGAA CCTCACTGGA TTACCCCTGC TGATCGACAA CTATGTGCCC

Dog AGGAAGGGAA CCTGATTGGA TTACCCCTGC TGATTGACAA CTATGTGCCC

Elephant AGGAAGGGAA CCTGATTGGA TTACCCCTTC TGATTGACAA CTATGTGCCC

Cow AGGAAGGGAA CCTGGTTGGA TTACCCCTTC TGATCGACAA CTATGTGCCC

Horse AGGAAGGGAA CCTGATTGGA TTACCCCTTC TGATCGACAA CTATGTGCCC

Opossum AGGAAGGAAA CGTGACCGGC CTGCCCCTTC TCCTGGACAA CTACGTGCCC

Platypus AGGAAGGAAA CCTGATAGGA CTGCCCCTTC TGATCGACAA CTACATCCCC

Frog AGGAAGGTAA CCTTATAGGA TTACCTATAC TGCTAGACAA CTACATCCCT

Chicken AGGAAGGAAA CCTTACTGGA TTACCACTTC TGATAGACAA CTACATTCCC

Zebra_Finch AGGAAGGAAA TCTTAGTGGG TTACCACTTC TGATAGACAA CTATGTCCCA

Fugu AGGAAGGAAA CCTAACAGGA TTGCCATTAC TGCTTGACAA GTACACCCCC

Zebrafish CAGAGGGAAA CCTGACAGGT CTGCCAATGC TGTTGGATAA TTACACTCCT

Human CCTTTGGAGG GACTGCCTAT CTTCATTCTT CGACTAGCCA CTGAGGTGAA

Chimpanzee CCTTTGGAGG GACTGCCTAT CTTCATTCTT CGACTAGCCA CTGAGGTGAA

Gorilla CCTTTGGAGG GACTGCCTAT CTTCATTCTT CGACTAGCCA CTGAGGTGAA

Orangutan CCTTTGGAAG GACTGCCTAT CTTCATTCTT CGACTAGCCA CTGAGGTGAA

Rat CCTTTGGAGG GACTGCCTAT CTTCATTCTT CGACTGGCCA CTGAGGTGAA

Guinea_Pig CCTTTGGAGG GACTGCCCAT CTTTATCCTC CGACTAGCCA CAGAGGTAAA

Mouse CCTTTGGAGG GACTGCCTAT CTTCATTCTT CGACTGGCCA CTGAGGTGAA

Rabbit CCTCTGGAGG GCCTGCCCCT CTTCATCCTT CGACTGGCCA CTGAGGTGAA

Dog CCTTTAGAGG GTCTGCCTAT CTTCATTCTT CGACTAGCCA CGGAGGTGAA

Elephant CCTCTGGAGG GACTGCCTAT CTTCATCCTT CGACTGGCCA CTGAGGTGAA

Cow CCGCTGGAGG GTCTGCCTAT CTTCATCCTC CGACTGGCCA CAGAGGTGAA

Horse CCTTTGGAGG GTCTGCCTAT CTTCATTCTT CGACTAGCCA CTGAGGTGAA

Opossum CAACTGGAGG GGCTCCCCAT GTTCGTCCTG CGCTTGGCCA CCGAGGTAAA

Platypus CCGCTTGAGG GACTCCCGAT GTTCATCCTT CGCTTGGCCA CCGAGGTCAA

Frog CCTATGGAAG GATTGCCATT GTTTATTTTG CGCCTAGCCA CAGAGGTCAA

Chicken CTGCTGGAGG GACTACCGAT GTTCATCCTT CGTTTGGCCA CTGAGGTAAA

Zebra_Finch CCATTGGAAG GACTGCCTAT GTTTATCCTG CGCTTGGCCA CAGAGGTAAA

Fugu ATCATGGAGG GTCTCCCAAT GTTTATCCTG CGCCTGGCCA CTGAGGTGAA

Zebrafish GCCATGGAGG GACTGCCAAT GTTCATTTTG CGTTTAGCCA CTGAGGTGAA

Human TTGGGACGAA GAAAAGGAAT GTTTTGAAAG CCTCAGTAAA GAATGCGCTA

Chimpanzee TTGGGACGAA GAAAAGGAAT GTTTTGAAAG CCTCAGTAAA GAATGCGCTA

Gorilla TTGGGACGAA GAAAAGGAAT GTTTTGAAAG CCTCAGTAAA GAATGCGCTA

Orangutan TTGGGATGAA GAAAAGGAAT GTTTTGAAAG CCTCAGTAAA GAATGTGCTA

Rat CTGGGATGAA GAAAAGGAGT GTTTCGAAAG TCTCAGTAAA GAATGTGCTG

Guinea_Pig TTGGGATGAA GAAAAGGAAT GTTTTGAAAG CCTCAGTAAA GAATGTGCTA

Mouse TTGGGATGAA GAAAAGGAGT GTTTTGAAAG TCTCAGTAAA GAATGTGCTA

Rabbit CTGGGATGAA GAAAAGGAAT GCTTTGAGAG CCTCAGTAAA GAATGTGCTA

Dog CTGGGATGAA GAGAAGGAAT GTTTTGAAAG CCTCAGTAAA GAATGTGCTA

Elephant TTGGGATGAA GAAAAGGAGT GTTTCGAAAG CCTCAGTAAA GAATGTGCGC

Cow CTGGGATGAA GAAAAGGAAT GTTTTGAAAG CCTCAGTAAA GAATGTGCGA

Horse CTGGGATGAA GAAAAGGAAT GTTTTGAAAG CCTTAGTAAA GAATGTGCTA

Opossum CTGGGACGAA GAGAAGGAAT GTTTCCAAAG CCTCAGCAAG GAGTGTGCCC

Platypus CTGGGATGAA GAAAAGGAGT GTTTCGAGAG CCTCAGTAAA GAATGCGCCA

Frog TTGGGATGAT GAGAAGGAGT GCTTTGAAAG TTTTAGCAAG GAATGCTCCA

Chicken CTGGGATGAA GAGAAGGAGT GCTTTGAAAC CCTAAGTAAA GAACTGTCTA

Zebra_Finch CTGGGATGAA GAAAAGGAGT GTTTTGAAAG CCTGAGTAAA GAACTAGCCA

Fugu CTGGGACAAT GAAAAGGAGT GCTTCAGAGA CTTCAGCAGG GAGTGCAGCA

Zebrafish CTGGGATAAA GAAAAGGAGT GTTTCCGTGA ATTCAGTGTT GAATGCAGTC

Human TGTTCTATTC CATCCGGAAG CAGTACATAT CTGAGGAGTC GACCCTCTCA

Chimpanzee TGTTCTACTC CATCCGGAAG CAGTACATAT CTGAGGAGTC GACCCTCTCA

Gorilla TGTTCTACTC CATCCGGAAG CAGTACATAT CTGAGGAGTC GACCCTCTCA

Orangutan TGTTCTACTC CATCCGGAAG CAGTACATAT CTGAGGAGTC GACCCTCTCA

Rat TGTTTTACTC CATTCGGAAG CAATACATAC TGGAGGAGTC AGCCCTCTCA

Guinea_Pig TGTTTTACTC CATCCGGAAG CAGTATGTAT CAGAGGAGTC GACCCTCTCG

Mouse TGTTTTACTC CATTCGGAAG CAGTATATAC TGGAGGAGTC GACCCTCTCA

Rabbit TGTTCTACTC GGTCCGCAAG CAGTACATAT CTGAGGAGTC GACCCTGTCA

Dog TGTTTTACTC CATTCGGAAG CAGTATATAT CTGAGGAGTC GACCCTCTCG

Elephant TGTTTTACTC CATCCGGCAG CAGTATGTAT GTGAGGGGTC CACCCTCTCC

Cow TGTTTTACTC CATCCGGAAG CAGTATGTGT CTGCAGAGTC AACCCTTTCA

Horse TGTTCTACTC CATCCGGAAG CAGTATATAT CTGAGGAGTC AACCCTCTCA

Opossum GCTTCTACGC CATCAGGAAG CAGTATGCCT TCCCACTCAC ATCCCACCTT

Platypus TGTTTTACTC CATCAGGAAG CAATACGTCG CGGAGGACCC CACGCCCTCG

Frog TGTTTTACTC TATTCGGAAG CAGTATATAT TAGAAGAATC T------TCC

Chicken TGTTCTACTC CATTAGAAAA CAATATATAA TAGATGAAAC CAACCTGACG

Zebra_Finch TGTTCTACTC CATCAGAAAG CAGTATATAA TAGAGGAATC CAACCCAACA

Fugu TGTTCTACTC CATCAGGAAA GAATTCATCC TGGAC----- ----------

Zebrafish ATTTCTACTC CATAAGGAAA AGCTACACAC TGGAG----- ----------

Human GGCCAGCAGA GTGAAGTGCC TGGCTCCATT CCAAACTCCT GGAAGTGGAC

Chimpanzee GGCCAGCAGA GTGAAGTGCC TGGCTCCATT CCAAACTCCT GGAAGTGGAC

Gorilla GGCCAGCAGA GTGAAGGGCC TGGCTCCATT CCAAACTCCT GGAAGTGGAC

Orangutan GGCCAGCAGA GTGAAGTGCC TGGCTCCATT CCAAACTCCT GGAAGTGGAC

Rat GGCCAGCAGA GTGACATGCC TGGCTCCCCT TCAAAGCCCT GGAAGTGGAC

Guinea_Pig AGCCAGCAGA GTGAAGTGTC AGGCTCCACT CCAAACCCCT GGAAGTGGAC

Mouse GGCCAGCAGA GTGACATGCC TGGCTCCACG TCAAAGCCCT GGAAGTGGAC

Rabbit GGCCAGCAGA GCAAAGCCGC TGGCTCCAGC GTGAACTCCT GGAAGTGGAC

Dog GGCCAGCAGA GTGAAGTATG CGGCTCCTCT GCAAACCCCT GGAAGTGGAC

Elephant GGCCAGCAGA GTGAAGTGCC TGGCTCTGCT GCAAAGCCCT GGAAGTGGAC

Cow GGCCAGCAGA GTGAAGTGCC TGGCTCCACT GCAAACCCCT GGAAGTGGAC

Horse GGCCAGCAGA GTGAAGTGCC TGGTGCCACT CGAAACCCCT GGAAGTGGAC

Opossum TCTCTGCAGA AGGAAGAGTG TGAATCCTCT CCAGTGTCTT GGAAGTGGAC

Platypus GACTCTCAGG GTGAGATGCC CGGCTCCTCC CCGGTGTCGT GGAAGTGGAC

Frog AGTAATGCGG ACAATGAATC CTTAACAGGG TCTTCCTCTT GGCGCTGGAC

Chicken CGCTCTCAGA ATGAGGATTC TGACTCTGGT CCACCGCCAT GGAAATGGAC

Zebra_Finch AACTCTCAGA ATGAAGAATC TGAGTCTGGT TCAACAACAT GGAAATGGAC

Fugu GGGGAGCCAG GAGAGGAGCA GGAGACTGAG GTGACCTCAT GGCGGTGGAA

Zebrafish GCAGACGCAG ATGAGCCACA GGATGCTGAG ATGAGC---T GGCAGTGGAA

Human TGTGGAACAC ATTGTCTATA AAGCCTTGCG CTCACACATT CTGCCTCCTA

Chimpanzee TGTGGAACAC ATTGTCTATA AAGCCTTGCG CTCACACATT CTGCCTCCTA

Gorilla TGTGGAACAC ATTGTCTATA AAGCCTTGCG CTCACACATT CTGCCTCCTA

Orangutan TGTGGAACAC ATTGTCTATA AAGCCTTGCG CTCACACATT CTGCCTCCTA

Rat TGTGGAACAC ATTATCTATA AAGCCTTCCG CTCACACCTT CTGCCTCCGA

Guinea_Pig TGTGGAACAC ATTGTTTACA AAGCCTTCCG CTCACATCTT ATGCCTCCAA

Mouse TGTGGAGCAC ATTATCTATA AAGCCTTCCG CTCACACCTC CTACCTCCGA

Rabbit CGTGGAACAC GTCGTGTACA AGGCCTTCCG CTCCCACCTG CTGCCCCCGA

Dog TGTGGAACAC ATTGTCTATA AAGCCTTCCG CTCACACCTT CTGCCTCCCA

Elephant TGTGGAGCAC GTCGTCTATA AAGCCTTCCG CTCACACCTC CTGCCTCCTA

Cow TGTGGAACAC GTCATTTATA AAGCCTTCCG CTCCCACCTG CTGCCTCCAA

Horse TGTGGAACAC ATTGTCTATA AAGCTTTCCG CTCACACCTT CTGCCTCCTA

Opossum GGTGGAGCAC ATTCTCTACA AAGCCTTCCG GTCCCATCTC TTCCCCCCAA

Platypus GGTGGAGCAC GTCATCTACA AGGCCTTCAG GTCCCACGTC CTTCCCCCTA

Frog TACAGAACAC ATTCTATACA AAGCCTTCAG AACTCACCTG CTACCTCCAA

Chicken AGTGGAGCAT GTTGTTTACA AAGCCTTTAG GACTCATCTT TTACCTCCTA

Zebra_Finch TGTGGAACAT GTGCTTTACA AAGCTTTTAG GACTTATCTT TTACCTCCTA

Fugu AGTTGAACAC GTCATCTTTA AGGCTTTCCG GATGCTCTTC AGTCCTCCAA

Zebrafish GGTGGAGCAT GTGCTTTTCA AAGCTTTGCG CTCTCTCTTC AGTCCTGCCA

Human AACATTTCAC AGAAGATGGA AATATCCTGC AGCTTGCTAA CCTGCCTGAT

Chimpanzee AACATTTCAC AGAAGATGGA AATATCCTGC AGCTTGCTAA CCTGCCTGAT

Gorilla AACATTTCAC AGAAGATGGA AATATCCTGC AGCTTGCTAA CCTGCCTGAT

Orangutan AACATTTCAC AGAAGATGGA AATATCCTGC AGCTTGCTAA CCTGCCTGAT

Rat AGCATTTCAC AGAAGATGGC AATGTCCTGC AGCTCGCCAA CCTGCCAGAT

Guinea_Pig AACATTTCAC AGAAGATGGA AATGTCCTGC AGCTTGCTAA CCTTCCAGAT

Mouse AGCATTTCAC AGAAGATGGC AATGTCCTGC AGCTTGCCAA CCTGCCAGAT

Rabbit AACACTTCAC GGAGGACGGA AACATCCTGC AGCTCGCTAA CCTGCCCGAC

Dog AACATTTCAC AGAAGATGGA AATATCCTAC AGCTTGCTAA CCTGCCCGAT

Elephant AACATTTCAC AGAAGATGGG AATATCCTGC AGCTTGCTAA CCTGCCTGAT

Cow AACACTTCAC AGAAGATGGG AACATCCTGC AGCTTGCTAA CCTGCCTGAT

Horse AACATTTCAC AGAAGATGGA AACATCCTGC AGCTTGCCAA CCTGCCCGAT

Opossum CACATTTCTC CGAGGATGGG AACATCCTGC AGCTCGCCAA CCTGCCAGAT

Platypus AACACCTCAC TGAGGACGGC ACCATCCTCC AGCTGGCCAA TCTTCCAGAT

Frog AGCCCTTTTC AGAAGATGGA AGCATCCTTC AGCTTGCAAA TCTCCCTGAC

Chicken AACACTTCAC AGATGATGGC AACATTTTGC AGCTTGCTAA CCTGCCTGAC

Zebra_Finch AACACTTTGC AGAAGATGGC AACATTTTGC AGCTTGCTAA CCTGCCTGAC

Fugu AGAAGTTTAG CGAGGACGGA ACTGTGTTGC AGATCGCCAA CCTCCCTGAT

Zebrafish AACACTTGAG TGAAGACGGC AGTGTCCTGC AGATCGCCAG CCTGCCTGAC

Human CTATACAAAG TCTTTGAGAG GTGT

Chimpanzee CTATACAAAG TCTTTGAGAG GTGT

Gorilla CTATACAAAG TCTTTGAGAG GTGT

Orangutan CTATACAAAG TCTTTGAGAG GTGT

Rat CTGTACAAAG TCTTTGAGCG GTGC

Guinea_Pig CTATACAAAG TCTTTGAAAG GTGT

Mouse CTATACAAAG TCTTTGAGCG GTGT

Rabbit CTATACAGAG TCTTTGAGAG GTGC

Dog CTGTACAAAG TCTTCGAGAG GTGT

Elephant CTATACAGAG TCTTTGAGAG GTGC

Cow CTCTACAAAG TCTTCGAGAG GTGC

Horse CTATACAGAG TCTTTGAGAG GTGT

Opossum CTCTACAAAG TGTTTGAGAG GTGC

Platypus CTGTACAAAG TCTTCGAGCG GTGC

Frog TTATATAAAG TGTTTGAAAG ATGC

Chicken TTGTATAAAG TTTTTGAGAG ATGC

Zebra_Finch TTGTATAAAG TTTTTGAAAG ATGT

Fugu CTTTATAAAG TGTTTGAGAG GTGT

Zebrafish CTCTACAAAG TGTTTGAAAG ATGC

;

END;

BEGIN DATA;

DIMENSIONS NTAX=18 NCHAR=2802;

FORMAT DATATYPE=DNA INTERLEAVE=yes GAP=-;

[Gene Name: MSH2]

[Name: Human Len: 2802 Check: 0]

[Name: Chimpanzee Len: 2802 Check: 0]

[Name: Orangutan Len: 2802 Check: 0]

[Name: Gorilla Len: 2802 Check: 0]

[Name: Marmoset Len: 2802 Check: 0]

[Name: Guinea_Pig Len: 2802 Check: 0]

[Name: Mouse Len: 2802 Check: 0]

[Name: Rat Len: 2802 Check: 0]

[Name: Cow Len: 2802 Check: 0]

[Name: Dog Len: 2802 Check: 0]

[Name: Rabbit Len: 2802 Check: 0]

[Name: Horse Len: 2802 Check: 0]

[Name: Elephant Len: 2802 Check: 0]

[Name: Chicken Len: 2802 Check: 0]

[Name: Zebra_Finch Len: 2802 Check: 0]

[Name: Zebrafish Len: 2802 Check: 0]

[Name: Frog Len: 2802 Check: 0]

[Name: Fugu Len: 2802 Check: 0]

MATRIX

Human ATGGCGGTGC AGCCGAAGGA GACGCTGCAG TTGGAGAGCG CGGCCGAGGT

Chimpanzee ATGGCGGTGC AGCCGAAGGA GACGCTGCAG TTGGAGAGCG CGGCCGAGGT

Orangutan ATGGCGGTCC AGCCGAAGGA GACGCTGCAG TTGGAGAGCG CGGCCGAGGT

Gorilla ATGGCGGTGC AGCCGAAGGA GACGCTGCAG TTGGAGAGCG CGGCCGAGGT

Marmoset ---------- ---------- ---------- ---------- ----------

Guinea_Pig ATGGCGGTGC AGCCTAAGGA GACGCTGCAG CTGGAGAGCG TGGCCGAGGC

Mouse ATGGCGGTGC AGCCTAAGGA GACGCTGCAG TTGGAAGGCG CGGCCGAGGC

Rat ATGGCGGTGC AGCCTAAGGA GACGCTGCAG TTGGAGGGCG CGGCCGAGGT

Cow ATGGCGGTGC AGCCGAAGGA CACCCTGCAG CTGGATAGCG CGGCCGAGGT

Dog ATGGCGGTGC AGCCCAAGGA GACGCTGCAG CTGGAGAGCG CGGCCGAGGT

Rabbit ATGGCGGTGC AGCCGAAGGA CACGCTGCAG CTGGAGAGCG CGGCCGAAGT

Horse ATGGCGGTGC AGCCCATGGA GACGCTGCAG CTGGAGAGCG CGGCCGAGGC

Elephant ---------- ---------- ---------- ---------- ----------

Chicken ---------- ---------- ---------- ---------- ----------

Zebra_Finch ---------- ---------- ---------- ---------- ----------

Zebrafish ATGGCGGTGC AACCTAAACA GAATCTGTCT ATGGACAGCG CGTCTGAACA

Frog ---------- ---------- ---------- ---------- ----------

Fugu ATGGCGGTGC AACCGAAGCA CAACCTTTCG ATGGACTCTG CAGCTGAGAG

Human CGGCTTCGTG CGCTTCTTTC AGGGCATGCC GGAGAAGCCG ACCACCACAG

Chimpanzee CGGCTTCGTG CGCTTCTTT- --GGCATGCC GGAGAAGCCG ACCACCACAG

Orangutan CGGCTTCGTG CGCTTCTTTC AGGGCATGCC GGAGAAGCCG ACCACCACGG

Gorilla CGGCTTCGTG CGCTTCTTTC AGGGCATGCC GGAGAAGCCG ACCACCACAG

Marmoset ---------- ---------- ---------- ---------- ----------

Guinea_Pig CGGCTTCGTG CGTTTCTTCC AGGCCATGCC GGAGAAGCCG ACCACCACTG

Mouse GGGCTTCGTG CGCTTCTTTG AGGGCATGCC GGAGAAGCCG AGCACCACGG

Rat GGGCTTCGTA CGCTTCTTTG AGGGCATGCC GGAGAAGCCG AGCACCACGG

Cow CGGCTTTGTG CGCTTCTTCC AAGGCATGCC GGAGAAGCCG ACCACCACAG

Dog CGGCTTCGTG CGCTTCTTCC AGGCCATGCC GGAGAAGCCG ACCACCACGG

Rabbit CGGCTTCGTG CGCTTCTTCC AGGGCATGCC GGAGAAGCCC ACCACTACCG

Horse CGGCTTCGTG CGCTTCTTCC AGGGCATGCC GGAGAAGCCG ACCACA---G

Elephant ---------- ---------- ---------- ---------- ----------

Chicken ---------- ---------- ---------- ---------- ----------

Zebra_Finch ---------- ---------- ---------- ---------- ----------

Zebrafish TGGATTCCTC AATTTTTACT TTTCGATGTC CGACAAACCA GACACCACGG

Frog ---------- ---TTCTACC AGTCCATGCC CGAGAAGCCC GACACCACTG

Fugu CGGATTTGTG AACTTCGTTT TCTCCCTTCC GGACAAACCG GACACCACCT

Human TGCGCCTTTT CGACCGGGGC GACTTCTATA CGGCGCACGG CGAGGACGCG

Chimpanzee TGCGCCTTTT CGACCGGGGC GACTTCTAT- --GCGCACGG CGAGGACGCG

Orangutan TGCGCCTTTT CGACCGCGGC GACTTCTATA CGGCGCACGG CGAGGACGCG

Gorilla TGCGCCTTTT CGACCGGGGC GACTTCTATA CGGCGCACGG CGAGGACGCG

Marmoset ---------- ---------- ---------- ---------- ----------

Guinea_Pig TGCGCCTCTT CGACCGTGGC GACTTTTACA CGGCGCACGG CGAGGACGCG

Mouse TGCGCCTCTT CGACCGCGGC GACTTTTACA CGGCGCACGG AGAGGACGCG

Rat TGCGCCTCTT CGACCGCGGC GACTTTTACA CCGCGCACGG CGAGGACGCG

Cow TGCGCCTTTT TGACCGGGGT GACTTCTACA CCGCGCACCG GGAGGACGCA

Dog TGCGCCTCTT CGACCGGGGC GACTTCTACA CGGCGCACCA CGAGGACGCG

Rabbit TGCGCCTTTT CGACAGGGGC GACTTCTACA CGGCGCACGG GGAGGACGCG

Horse TGCGCCTCTT CGACCGGGGG GACTTCTACA CGGCGCACGG CGAGGACGCG

Elephant ---------- ---------- ---------- ---------- ----------

Chicken ---------- ---------- ---------- ---------- ----------

Zebra_Finch ---------- ---------- ---------- ---------- ----------

Zebrafish TGCGAGTGTT TGATAGAAAC GATTACTATA CTGTGCACGG GAAAGATGCG

Frog TGCGGGTGTT CGATAGGAAC GACTACTACA CGGTGCACGG AGGCGATGCA

Fugu TCCGGGTGTT TGACCGCAGC GACTTCTACA CGGTTCACGG GAAAGACGCC

Human CTGCTGGCCG CCCGGGAGGT GTTCAAGACC CAGGGGGTGA TCAAGTACAT

Chimpanzee CTGCTGGCCG CCCGGGAG-- ---------- ---------- ----------

Orangutan CTGCTGGCCG CCCGGGAGGT GTTCAAGACC CAGGGGGTGA TCAAGTACAT

Gorilla CTGCTGGCCG CCCGGGAGGT GTTCAAGACC CAGGGGGTGA TCAAGTACAT

Marmoset ---------- ---------- ---------- ---------- ----------

Guinea_Pig CTGCTGGCCG CCCGTGAGGT GTTCAAGACG CAGGGGGTGA TCAAGTACCT

Mouse CTGCTGGCGG CCCGCGAGGT GTTCAAGACC CAGGGCGTGA TCAAGTACAT

Rat CTGCTGGCGG CCCGCGAGGT GTTCAAGACC CAAGGCGTGA TCAAGTACAT

Cow CTGCTGGCCG CCCGCGAAGT GTTCAAGACC CAGGGGGTGG TCAAGTATAT

Dog CTGCTGGCCG CCCGAGAGGT GTTCAAGACG CAGGGGGTAA TCAAGTACCT

Rabbit CTGCTGGCCG CCCGGGAGGT GTTCAAGACC CAGGGGGTGG TCAAGTACAT

Horse CTGCTGGCCG CCCGGGAGGT GTTCAAG--- CAGGGGGTGG TCAAGTACAT

Elephant ---------- ---------- ---------- ---------- -----TTTTT

Chicken ---------- ---------- ---------- ---------- ----------

Zebra_Finch ---------- ---------- ---------- ---------- ----------

Zebrafish ATTTTTGCAG CTAAAGAGGT TTTCAAGACT AATGGAGTCA TCAAAAATCT

Frog ATCTTTGCCG CTAAAGAAGT TTTCAAGACC AATGGGGTGA TCAAGTACTT

Fugu ATTTACGCAG CGAGGGAGGT TTTCAAGACG AACGGAGTCA TCAAATACCT

Human GGGGCCGGCA GGAGCAAAGA ATCTGCAGAG TGTTGTGCTT AGTAAAATGA

Chimpanzee ---------- ---GCAAAGA ATCTGCAGAG TGTTGTGCTT AGTAAAATGA

Orangutan GGGGCCGGCA GGAGCAAAGA ATCTGCAGAG TGTTGTGCTT AGTAAAATGA

Gorilla GGGGCCGGCA GGAGCAAAGA ATCTGCAGAG TGTTGTGCTT AGTAAAATGA

Marmoset ---------- ---------- ---------- ---------- ----------

Guinea_Pig AGGGCCGGCA GGAGCGAAGA CTCTGCAGAG TGTTGTGCTT AGCAAAATGA

Mouse GGGGCCGGCA GGGAGTAAGA CCCTGCAGAG TGTTGTGCTT AGCAAGATGA

Rat GGGGCCGGCA GGGGCAAAGA CCCTGCAGAC TGTTGTGCTG AGTAAGATGA

Cow GGGGCCGGCA GGAGCAAAGA CTCTGGAGAG TGTTGTGCTT AGTAAAATGA

Dog CGGGCCGGCA GGAACAAAGA CCCTGGAGAG TGTTGTGCTT AGTAAAATGA

Rabbit GGGGCCGGCA GGATCAAAGA CCCTGCAGAG TGTTGTGCTT AGTAAAATGA

Horse GGGGCCGGCA GGAGCAAAGA CTCTGGAGAG TGTTGTGCTT AGTAAAATGA

Elephant TCCCCCTTTA GGAGCAAAGA CTCTGGAGAG TGTTGTGCTT AGTAAAATGA

Chicken -CTTGCCCCA GGCAATAAA- --CTGGAGAG CGTTGTGCTC AGTAAAATGA

Zebra_Finch ---------- GGAACTAAG- --CTTGAGAG TGTTGTGCTT AGTAAAATGA

Zebrafish GGGTTCA--- GGGAACAGG- --TTGGAAAG TGTGGTCCTC AGTAAGATGA

Frog GGGATCAGCA GGAAGCAAGA AACTGGAGAG CGTGGTGCTC AGCAAAATGA

Fugu GGGCTCA--- GGTAGCCGCA GGCTGGAGAG CGTGGTCCTG AGCAAGCTCA

Human ATTTTGAATC TTTTGTAAAA GATCTTCTTC TGGTTCGTCA GTATAGAGTT

Chimpanzee ATTTTGAATC TTTTGTAAAA GATCTTCTTC TGGTTCGTCA GTATAGAGTT

Orangutan ATTTTGAGTC TTTTGTAAAA GATCTTCTTC TGGTTCGTCA GTATAGAGTT

Gorilla ATTTTGAATC TTTTGTAAAA GATCTTCTAC TGGTTCGTCA GTATAGAGTT

Marmoset ---------- ---------- ---------- ---------- ----------

Guinea_Pig ATTTTGAGTC TTTTGTAAAA GATCTTCTTT TGGTTCGTCA ATATAGAGTT

Mouse ACTTTGAGTC TTTCGTGAAA GATCTTCTTC TGGTTCGCCA GTATCGAGTT

Rat ATTTTGAGTC TTTCGTGAAA GATCTTCTTC TGGTTCGTCA CTATAGAGTT

Cow ATTTTGAGTC TTTTGTAAAA GATCTTCTTC TAGTTCGTCA GTACAGAGTT

Dog ATTTTGAATC TTTTGTTAAA GATCTTCTTC TAATTCGACA GTATAGAGTT

Rabbit ATTTTGAGTC CTTTGTAAAA GATCTCCTTC TAGTTCGTCA ATATAGAGTT

Horse ATTTTGAGTC TTTTGTAAAA GATCTTCTTC TAGTTCGTCA ATACAGAGTT

Elephant ATTTTGAGTC TTTTGTAAAA GGTCTTCTTC TAGTCCGTCA GTATAGAGTT

Chicken ACTTCGAGTC TTTTGTGAGA GATCTGCTGC TGGTTCGTCA TTACAGAGTG

Zebra_Finch ACTTTGAATC TTTTGTGAGG GACCTGCTTC TGGTTCGTCA TTACAGAGTT

Zebrafish ACTTTGAGTC ATTTGTGAGG GATTTGCTGC TGGTTAGACA ATACCGGGTG

Frog ACTTCGAGTC TGTAGTAAAG GATCTGCTTC TGGTTCGGCA GTATCGAGTA

Fugu ACTTCGAGGC CCTGGCCAGA GACTTGCTGC TGGTGAGGCA GTACCGGGTG

Human GAAGTTTATA AGAATAGAGC TGGAAATAAG GCATCCAAGG AGAATGATTG

Chimpanzee GAAGTTTATA AGAATAGAGC TGGAAATAAG GCATCCAAGG AGAATGATTG

Orangutan GAAGTTTATA AGAATAGAGC TGGAAATAAG GCATCCAAGG AGAATGATTG

Gorilla GAAGTTTATA AGAATAGAGC TGGAAATAAG GCATCCAAGG AGAATGATTG

Marmoset ---------- ---------- ---------- ---------- ----------

Guinea_Pig GAAGTTTATA AGAATAAAGC TGGAAATAAG GCATCCAAGG AAAACGATTG

Mouse GAAGTTTATA AGAATAAAGC TGGAAATAAG GCGTCTAAGG AGAATGAGTG

Rat GAAGTTTATA AGAATAAAGC TGGAAATAAG GCATCTAAGG AGAACGACTG

Cow GAAGTTTATA AGAACAGAGC TGGAAAT--- GCCTCCAAGG AGAATGATTG

Dog GAAGTTTACA AGAATAGAGC TGGAAGTAAG GCCTCCAAGG AGAACGATTG

Rabbit GAAGTTTATA AAAATAGAGC TGGAAATAAG GCCTCCAAGG AGAATGATTG

Horse GAAGTTTATA AGAATAGAGC TGGAAATAAG GCCTCCAAGG AGAATGATTG

Elephant GAAGTTTATA AGAATAAAGC TGGAAATAAG GCCTCCAAGG AGAATGATTG

Chicken GAAGTTTACA AGAATAAAGC AGGGAGTAAA TCTGTCAAGG AGAACGACTG

Zebra_Finch GAAGTTTACA AGAACAAAGC AGGGAGCAAA TCTGTCAAAG AAAATGACTG

Zebrafish GAGGTTTACA AGAAC----- -GCCAGTAAG AGCAGCAAAG AGCATGACTG

Frog GAAGTTTATA AAAATAAATC TGGAGGCAAA TCCTCAAAAG AGAATGATTG

Fugu GAGGTCTACA AGAAC----- -CACGGCAAA GGCAGCAAAG ACCACGACTG

Human GTATTTGGCA TATAAGGCTT CTCCTGGCAA TCTCTCTCAG TTTGAAGACA

Chimpanzee GTATTTGGCA TATAAGGCTT CTCCTGGCAA TCTCTCTCAG TTTGAAGACA

Orangutan GTATTTGGCA TATAAGGCTT CTCCTGGCAA TCTCTCTCAG TTTGAAGACA

Gorilla GTATTTGGCA TATAAGGCCA GCATAATAAC GTTCATCGAG TTTGGATTTT

Marmoset ---------- ---------- ---------- ---------- ----------

Guinea_Pig GTATTTAGCA TTTAAGGCTT CTCCTGGCAA TCTTTCTCAG TTTGAAGACA

Mouse GTATCTGGCA TTTAAGGCTT CTCCCGGCAA TCTTTCTCAG TTTGAAGACA

Rat GTATTTGGCA TATAAGGCTT CTCCTGGCAA TCTTTCTCAG TTTGAAGACA

Cow GTATTGGCAT TTTAAGTTTT CATACTGTGG TCCGTCAGTT GCCCCAGAAA

Dog GTATTTGGCA TTTAAGTCCC TTTGGGGAAA TTTTTTCAAA TATAACCCAA

Rabbit GTATTTGGCA TTTAAGGCTT CTCCTGGCAA TCTTTCCCAG TTTGAAGATA

Horse GTATTTGGCA TTTAAGGCTT CTCCAGGTAA TCTTTCTCAG TTTGAAGACA

Elephant GTATTTGGCA TTTAAGGCTT CTCCAGGTAA TCTTTCACAG TTTGAAGACA

Chicken GTACTTAGCC TACAAGGGTT CTCCAGGAAA TCTTGCCCAG TTTGAGGAAG

Zebra_Finch GTATTTAGCT TACAAGGGTT CTCCAGGAAA TCTTGCCCAG TTTGAAGAAG

Zebrafish GCAAATCGCT TTCAAGGCAT CTCCAGGAAA CCTTACTCAG TTTGAGGAGA

Frog GCAGCTGGCT TTTAAGGCCT CACCGGGTAA TCTTACACAG TTTGAGGAGA

Fugu GAGGGTCGAG TACAAGGCCT CTCCTGGAAA CCTAACCCAG TTTGAGGAGG

Human TTCTCTTTGG TAACAATGAT ATGTCAGCTT CCATTGGTGT TGTGGGTGTT

Chimpanzee TTCTCTTTGG TAACAATGAT ATGTCAGCTT CCATTGGTGT TGTGGGTGTT

Orangutan TTCTCTTTGG TAACAACGAC ATGTCAGCTT CCATTGGTGT TGTGGGTGTT

Gorilla TCCTTTTTGC TTATAAAATT TTAAAAGCTT CCATTGGTGT TGTGGGTGTT

Marmoset ---------- ---------- ---------- ---------- ----------

Guinea_Pig TTCTCTTTGG TAACAATGAT ATGGCAGCTT CCATTGGTGT TGTGGGCATT

Mouse TCCTGTTTGG TAACAATGAC ATGTCAGCTT CCGTTGGCGT TATGGGTATT

Rat TCCTGTTTGG TAACAATGAC ATGTCCACTT CCATTGGCAT TATGGGTATT

Cow TGCCTCATGT TAACAGGCAG CTGTCTGCTT CCATTGGTGT TGTGGGTGTT

Dog TTTTGTTAGC TACTGGTTCT CTTTCAACCT CCATTGGTGT CGTGGGTGTT

Rabbit TTCTCTTTGG TAACAATGAT ATGTCCTCTT CCATTGGTGT TGTGGGTGTT

Horse TTCTTTTTGG TAACAATGAT ATGTCAGCTT CCATTGGTGT TGTGGGTATT

Elephant TTCTGTTTGG TAACAATGAT ATGTCGTCGT CCATTGGAGT TGTGGGTATT

Chicken TTCTCTTTGC CAACAATGAT ATGTCAATGG CCATCGGGGT TGTGGGGGTG

Zebra_Finch TTCTCTTTGC CAACAATGAC ATGTCCACAG CCATCGGGGT TGTGGGGGTG

Zebrafish TTCTGTTTGG CAGTGGCGGA GCTGAGGGTG CTGTCGGTGT GGTGGGTGTT

Frog TTCTGTTTGG AAACAGTGAC ATGTCCACAG CCGTTGGCGT AGTTGGTATC

Fugu TTCTGTTTGG TAGCGGGTCT TCCGAGGCCT GCGCCGGCGT GGTGGCCGTG

Human AAAATGTCCG CAGTTGATGG CCAGAGACAG GTTGGAGTTG GGTATGTGGA

Chimpanzee AAAATGTCCG CAGTTGATGG CCAGAGACAG GTTGGAGTTG GGTATGTGGA

Orangutan AAAATGTCTG CAGTTGATGG CCAGAGACAG GTTGGAGTTG GGTATGTGGA

Gorilla AAAATGTCCG CAGTTGATGG CCAGAGACAG GTTGGAGTTG GGTATGTGGA

Marmoset ---------- ---------- ---------- ---------- ----------

Guinea_Pig AAAATGTCTG CAGTTGATGG TCAAAGACAG GTTGGAGTTG GGTATGTTGA

Mouse AAAATGGCCG TGGTTGATGG TCAAAGACAT GTTGGAGTTG GGTATGTGGA

Rat AAACTGTCTA CGGTTGATGG TCAGAGACAG GTTGGAGTTG GGTATGTGGA

Cow AAAATGTCTA CAGTCGATGG TCAAAGACAG GTCGGAGTTG GGTATGTTGA

Dog AAAATGTCTA CAGTTGATGG CCAAAGACAG GTCGGAGTTG GGTATGTTGA

Rabbit AAAATGTCCA CAGTTGATGG TCAAAGGCAA GTTGGAGTTG GATATGTTGA

Horse AAAATGTCTA CAGTTGATGG CCAAAGACAG GTTGGAGTTG GGTATGTTGA

Elephant AAAATATCCG TAGTTGACGG CCAAAGACAA GTTGGCGTTG GGTATGTTGA

Chicken AAGCTGTCTT CTGCTGATGG CCAAAGGGTA ATAGGAGTAG GGTATGTGGA

Zebra_Finch AAGCTGTCTG CTGCTGATGG GCAGAGAGTA GTAGGAGTGG GCTATGTCGA

Zebrafish CGTCTTGGCA CTACTGATGG CCAGCGTGTT GTCGGTGTTG GTTATGTGGA

Frog AAGCTGGTCA GTGCTGAAGG CCAGAGGTTG GTTGGGGTTG GTTATGTGGA

Fugu CGCTTCGCCG CGGCCGACGG CCAGCGCGTG GTCGGCGTGG GCTACGTGGA

Human TTCCATACAG AGGAAACTAG GACTGTGTGA ATTCCCTGAT AATGATCAGT

Chimpanzee TTCCATACAG AGGAAACTAG GACTGTGTGA ATTCCCTGAT AATGATCAGT

Orangutan TTCCATACAG AGGAAACTAG GACTGTGTGA ATTCCCTGAT AATGATCAGT

Gorilla TTCCATACAG AGGAAACTAG GACTGTGTGA ATTCCCTGAT AATGATCAGT

Marmoset ---------- ---------- ---------- ---------- ----------

Guinea_Pig CTCTGCGCAG AGGAAGCTAG GACTATGTGA GTTCCCTGAT AATGATCAGT

Mouse TTCCACCCAG AGGAAGCTAG GCTTGTGTGA GTTCCCCGAG AATGATCAGT

Rat TTCCACCCAG AGAAAGCTAG GATTGTGTGA GTTCCCTGAC AACGATCAGT

Cow CTCCACACAG AGGAAGCTGG GACTGTGTGA GTTCCCTGAT AATGATCAGT

Dog CTCCATACAG AGGAAGCTAG GACTTTGTGA GTTCCCTGAT AATGATCAGT

Rabbit TTCCATGCAG AGGAAGCTAG GACTGTGCGA GTTCCCTGAT AATGATCAGT

Horse CTCCATACAG AGGAAGCTGG GACTGTGTGA GTTCCCGGAT AATGATCAGT

Elephant CTCCATCCAG AGGAAGCTAG GACTGTGCGA GTTCCCTGAT AATGATCAGT

Chicken CACTACGCTG AGAAAACTGA GCGTCTGTGA ATTCCCAGAT AATGATCAGT

Zebra_Finch CACTACGCTG AGAAAATTGA GTGTTTGTGA GTTCCCAGAT AATGATCAGT

Zebrafish CAGTACCCTC AGGAAACTTG GTGTGTGCGA GTTCCCTGAT AACGATCAGT

Frog CTCCACACTG AGAAAGCTAG GAGTATGTGA ATTTCCAGAT AATGATCAGT

Fugu CGCCGCACAG AGGACGATGG GCGTGTGTGA GTTTCCAGAC AACGAGATCT

Human TCTCCAATCT TGAGGCTCTC CTCATCCAGA TTGGACCAAA GGAATGTGTT

Chimpanzee TCTCCAATCT TGAGGCTCTC CTCATCCAGA TTGGACCAAA GGAATGTGTT

Orangutan TCTCCAATCT TGAGGCTCTC CTCATCCAGA TTGGACCAAA GGAATGTGTT

Gorilla TCTCCAATCT TGAGGCTCTC CTCATCCAGA TTGGACCAAA GGAATGTGTT

Marmoset ---------- ---------- ---------- ---------- ----------

Guinea_Pig TCTCCAATCT CGAGGCGCTC TTGATTCAGA TTGGACCAAA GGAATGTGTC

Mouse TCTCCAATCT CGAGGCTCTT CTGATTCAGA TTGGACCAAA GGAATGCGTT

Rat TCTCCAATCT CGAGGCTCTT CTGATTCAGA TTGGACCAAA GGAATGCATT

Cow TCTCCAATCT GGAGGCTCTC CTGATTCAGA TTGGACCAAA GGAGTGTGTT

Dog TCTCCAATTT GGAGGCTCTC CTGATTCAGA TTGGACCAAA GGAATGTGTT

Rabbit TCTCCAATCT TGAGGCTCTC CTGATTCAGA TTGGACCAAA GGAATGTGTT

Horse TCTCCAATCT GGAGGCTCTG CTGATTCAGA TTGGACCAAA GGAATGTGTT

Elephant TCTCCAATCT TGAAGCTCTC CTGGTTCAGA TTGGACCCAA GGAATGCGTT

Chicken TCTCAAACCT TGAAGCCCTG CTGGTTCAGC TGGGACCTAA GGAGTGTGTG

Zebra_Finch TCTCCAACCT TGAAGCTCTA CTGGTTCAGC TGGGCCCAAA GGAGTGTGTG

Zebrafish TCTCCAACCT GGAGGCTCTA CTTGTGCAAA TCGGCCCCAA AGAGTGTGTG

Frog TCTCTAATCT GGAAGCTTTG TTGGTTCAGA TTGGCCCAAA GGAATGTGTA

Fugu TCTCAAACTT GGAGGCCCTG CTGGTCCAAA TCAGCCCCAA AGAGTGTCTC

Human TTACCCGGAG GAGAGACTGC TGGAGACATG GGGAAACTGA GACAGATAAT

Chimpanzee TTACCCGGAG GAGAGACTGC TGGAGACATG GGGAAACTGA GACAGATAAT

Orangutan TTACCCGGAG GAGAGACTGC TGGAGACATG GGGAAACTGA GACAGATTAT

Gorilla TTACCCGGAG GAGAGACTGC TGGAGACATG GGGAAACTGA GACAGATTAT

Marmoset ---------- ---------- ---------- ---------- ----------

Guinea_Pig TTACCAGGAG GAGAGACTGC TGGAGACATG GGGAAGCTGC GGCAGATTAT

Mouse TTACCAGGAG GAGAGACTAC TGGAGACATG GGGAAACTGA GGCAGGTTAT

Rat TTACCAGGCG GAGAGACTGC TGGAGACATG GGGAAACTGA GGCAGGTTAT

Cow ATGCCAGGAG GGGAGACTGC TGGAGACATG GGAAAACTGA GGCAGGTTAT

Dog TTACCAGGAG GAGAGACTGC TGGAGACATG GGAAAACTGA GGCAGGTTAT

Rabbit TTACCGGGAG GAGAGACTGC CGGAGACATG GGAAAACTGA GACAGGTTAT

Horse TTACCCGGAG GAGAGACTGC TGGAGACATG GGAAAACTGA GGCAGGTAAG

Elephant TTACCAGGAG GAGAGACTAC CGGAGACATG GGAAAACTGA GGCAGATTAT

Chicken CTGCCGGGAG GGGACACTGC AGGAGAGATG GGGAAACTGC GGCAGGTCGT

Zebra_Finch CTGCCAGGAG GAGAGACTGC AGGAGAGATG GGGAAACTCA GACAAGTCAT

Zebrafish CTTCCTGCAG GAGATTCAGG AGGAGACCTG GGCAAACTTA AACAGGTGGT

Frog ATGCCTGGAG GAGACACGGC TGGAGACATG GGGAAATTGA GGCAGATTGT

Fugu CTGGCCCAGG GCGACGGCGG CGCCGACGGC AGCAAACTGC GAGAGGTGGT

Human TCAAAGAGGA GGAATTCTGA TCACAGAAAG AAAAAAAGCT GACTTTTCCA

Chimpanzee TCAAAGAGGA GGAATTCTGA TCACAGAAAG AAAAAAAGCT GACTTTTCCA

Orangutan TCAAAGAGGA GGAATTCTGA TCACAGAAAG AAAAAAAGCT GACTTTTCCA

Gorilla TCAAAGAGGA GGAATTCTGA TCACAGAAAG AAAAAAAGCT GACTTTTCCA

Marmoset ---------- ---------- ---------- ---------- ----------

Guinea_Pig TCAGAGAGCA GGAATTCTGA TCACAGAAAG AAAAAGAGCT GAGTTTTCCA

Mouse CCAGAGAGGA GGGATTCTGA TCACAGAAAG AAAGAGAGCC GACTTTTCCA

Rat ACAGAGAGGA GGGATTCTGA TCACAGAAAG AAAGAGAATC GACTTTTCCA

Cow TCAGAGAGGA GGAATTTTGA TCACAGAAAG AAAAAGAGCT GACTTTTCCA

Dog TCAGAGAGGA GGAATTCTGA TCACAGAACG AAAAAGAGCT GATTTTTTTA

Rabbit TCAGAGAGGA GGAATTCTGA TCACAGAAAG AAAAAAAGCT GACTTTTCTT

Horse GGAAGCAAGT CTAGTGATAG AGGAGAGTCC AAGCCTAGCA GAGGCTCCAA

Elephant TCTGAGAGGA GGGATTCTGA TCACAGACAG AAAAAGAGCT GAATTTTCCG

Chicken TCAGAGAGGA GGAATCCTGA TTACAGACAG GAAGAAGGCA GATTTCACGA

Zebra_Finch TCAGAGGGGA GGAATCCTGA TTACAGACAG GAAGAAGGCA GATTTCACAA

Zebrafish GCAGCGTGGT GGCATTCTCC TCACAGACAG GAAGAAGTCA GAGTTCACAA

Frog TAAGAGAGGT GGGATTCTGA TTACAGACAG AAAGAAGGCA GAATTCACCA

Fugu CCAGCGCGGC GGCGCTTTGG TCTCGGAGCG GAAGAAAGCA GAGTTTCATA

Human CAAAAGACAT TTATCAGGAC CTCAACCGGT TGTTGAAAGG CAAAAAGGGA

Chimpanzee CAAAAGACAT TTATCAGGAC CTCAACCGGT TGTTGAAAGG CAAAAAGGGA

Orangutan CAAAAGACAT TTATCAGGAC CTTAACCGGT TGTTGAAAGG CAAAAAGGGA

Gorilla CAAAAGACAT TTATCAGGAC CTCAACCGGT TGTTGAAAGG CAAAAAGGGA

Marmoset ---------- ---------- ---------- ---------- ----------

Guinea_Pig CAAAAGACAT TTACCAGGAC CTCAACCGGT TGTTGAAAGG CAAAAAAGGA

Mouse CTAAAGACAT TTATCAGGAT CTCAACCGGT TACTGAAAGG CAAAAAAGGA

Rat CAAAAGACAT CTACCAGGAT CTCAACCGGT TACTGAAAGG CAGAAAAGGA

Cow CAAAAGATAT TTATCAAGAC CTCAACCGGT TGCTGAAAGG CAAGAAGGGA

Dog CAAAAGATAT TTATCAGGAC TTGAACCGGT TATTGAAAGG CAAAAAGGGA

Rabbit CAAAAGACAT TTATCAGGAT CTCAACCGGT TGTTGAAAGG CAAAAAGGGA

Horse TTAACAGGAT ACTAGAGCGC TACAACCGGT TGTTGAAAGG CAAAAAGGGA

Elephant CAAAAGACAT TTATCAGGAT CTCAACCGGT TGTTGAAAGC CAAAAAGGGA

Chicken CAAAGGATAT TGTTCAGGAT CTCAATCGCT TGTTAAAATC GAGGAAAGGA

Zebra_Finch CAAAAGATAT TGTTCAGGAT CTCAATCGCT TGTTGAAACA AAAAAAGGAA

Zebrafish CTAAAGACAT TGTGCAGGAT CTGAATCGGC TACTGAAGGC CCGTAAAGGA

Frog CAAAAGACAG TGTACAAGAC CTTAATCGTC TGCTTAAAGC AAAGAAAGGA

Fugu GCAAGGACCT GGTCCAGGAC CTCAACCGGC TGCTGCGGGC CAAGAAGGGA

Human GAGCAGATGA ATAGTGCTGT ATTGCCAGAA ATGGAGAATC AGGTTGCAGT

Chimpanzee GAGCAGATGA ATAGTGCTGT ATTGCCAGAA ATGGAGAATC AGGTTGCAGT

Orangutan GAACAGATGA ATAGTGCTGT ATTGCCAGAA ATGGAGAATC AGGTTGCAGT

Gorilla GAGCAGATGA ATAGTGCTGT ATTGCCAGAA ATGGAAAATC AGGTTGCAGT

Marmoset ------ATGA ATAGTGCTGT ATTGCCAGAA ATGGAGAATC AGGTTGCAGT

Guinea_Pig GAACAGATGA ATAGTGCTGT CTTGCCAGAA ATGGAAAACC AGGTTGCAGT

Mouse GAACAGATAA ATAGTGCTGC CCTACCAGAG ATGGAGAATC AGGTTGCAGT

Rat GAGCAGATGA ACAGCGCTGT CTTACCAGAG ATGGAGAATC AGGTTGCAGT

Cow GAACAGGTGA ATAGTGCTGT GTTGCCAGAA ATGGAGAACC AGGTTGCAGT

Dog GAACAGGTGA ATAGTGCTGT CTTGCCAGAA ATGGAGAATC AGGTTGCAGC

Rabbit GAACAAATGA ATAGTGCTGT GTTGCCAGAA ATGGAGAATC AGGTTGCAGT

Horse GAACAGATGA ATAGCGCTGT TTTGCCAGAA ATGGAGAATC AGGTTGCTGT

Elephant GAACAGATGA ATAGTGCTGT CTTGCCAGAA ATGGAGAATC AGGTCGCAGT

Chicken GAACAAATGA ACAGCGCAGC ATTGCCAGAG ATGGAAAAGC AGGTTGCCGT

Zebra_Finch GAGCAGCTGA ACAGTGCAGC ACTGCCAGAG ATGGAAAAGC AGGTTGCTGT

Zebrafish GAGACTGTGT CCAGCGCTGC CCTGCCTGAG ATGGAGAAAA AGATTGCAAT

Frog GAGCAGGTTA CAAGCGCTGC ATTACCAGAG ATGGAGAAAC AGGTTGCAAT

Fugu CAGAGCGTGT CCAGCAGGAC GCTCCCCGAG CTGGACAAAC AGGTCGCCGT

Human TTCATCACTG TCTGCGGTAA TCAAGTTTTT AGAACTCTTA TCAGATGATT

Chimpanzee TTCATCACTG TCTGCGGTAA TCAAGTTTTT AGAACTCTTA TCAGATGATT

Orangutan TTCATCACTG TCTGCGGTAA TCAAGTTTTT AGAACTCTTA TCAGATGATT

Gorilla TTCATCACTG TCTGCGGTAA TCAAGTTTTT AGAACTCTTA TCAGATGATT

Marmoset TTCATCATTG TCTGCAGTAA TCAAGTTTTT AGAACTATTA TCTGATGATT

Guinea_Pig TTCATCATTG TCTGCAGTAA TCAAGTTCTT AGACCTTTTA TCTGATGATT

Mouse TTCATCACTA TCTGCAGTAA TCAAGTTTTT AGAACTCTTA TCAGACGATT

Rat TTCATCGTTG TCTGCAGTGA TCAAGTTCCT AGAACTCTTA TCAGATGATT

Cow TTCTTCCTTG TCTGCAGTAA TCAAATTTTT AGAACTCTTA TCAGATGACT

Dog TTCAGCACTG TCTGCAGTAA TCAAGTTTTT AGAACTTTTA TCAGATGATT

Rabbit TTCATCATTA TCTGCAGTAA TCAAGTTTTT AGAACTCTTA TCTGATGATT

Horse TTCGTCGTTG TCTGCAGTAA TCAAGTTTTT AGAACTCTTA TCAGATGACT

Elephant TGCATCGTTG TCTGCAGTAA TCAAGTTTTT AGAACTCTTA TCAGATGATT

Chicken TTCATCTTTG TCAGCCGTTA TCAAGTTTTT AGAGTTGCTG TCAGATGACT

Zebra_Finch TTCATCTTTG TCAGCTGTTA TCAAGTTTTT GGAGTTGCTG TCAGATGAGT

Zebrafish GTCTTGCTTA GAAGCAGTCA TCAAATACCT GGAGCTTCTC GCAGATGAGG

Frog GTCGGCCTTG GCGTCAGTCA TGAAGTATTT AGAACTGTTG TCTGACGAGT

Fugu CTCGTGCCTG GCGGCAGTGG TGCGGTTCCT GGAGCTGCTG TCGGATGAGT

Human CCAACTTTGG ACAGTTTGAA CTGACTACTT TTGACTTCAG CCAGTATATG

Chimpanzee CCAACTTTGG ACAGTTTGAA CTGACTACTT TTGACTTCAG CCAGTATATG

Orangutan CCAATTTTGG ACAGTTTGAA CTGACTACTT TTGACTTCAG CCAGTACATG

Gorilla CCAACTTTGG ACAGTTTGAA CTGACTACTT TTGACTTCAG CCAGTATATG

Marmoset CCAATTTTGG ACAGTTTGAA CTGACTACTT TTGACTTTAG CCAGTACATG

Guinea_Pig CAAATTTTGG GCAGTTTGAA CTGACTACTT TTGACTTCAG CCAATACATG

Mouse CAAATTTTGG GCAGTTTGAA CTGGCCACTT TTGACTTCAG CCAGTACATG

Rat CGAATTTTGG GCAGTTTGAA CTGGCGACTT TTGACTTCAG CCAGTACATG

Cow CAAACTTTGG ACAGTTTGAA CTGACTACTT TTGACTTCAG CCAGTACATG

Dog CAAACTTTGG ACAGTATGAA CTCACTACTT TTGACTTCAG CCAGTATATG

Rabbit CTAATTTTGG ACAATTTGAG CTGACAACTT TTGACTTCAG CCAGTACATG

Horse CAAACTTCGG TCAGTTTGAA CTGACTACTT TTGACTTCAG CCAGTACATG

Elephant CAAACTTTGG ACAGTTTGAA CTGACTACTT TTGACTTCAA CCAGTACATG

Chicken CTAATTTTGG GCAATATGAA CTGACTACTT TTGATCTTAG TCAGTATATG

Zebra_Finch CTAATTTTGG ACAGTTTGAA CTGACTACAT TTGATCTTAG TCAATATATG

Zebrafish CCAATTTTGG GTCTTTCAAG ATGACCACCT TTGACCTCAA CCAGTACATG

Frog CAAACTTTGG GCAGTTTGTG ATGACCAACT TTGACCTGAG CCAGTATATG

Fugu CCAACTTCGG CTCCTTCAGT CTCACGTCCC TGGACCTGTC CCAGTACATG

Human AAATTGGATA TTGCAGCAGT CAGAGCCCTT AACCTTTTTC AGGGTTCTGT

Chimpanzee AAATTGGATA TTGCAGCAGT CAGAGCCCTT AACCTTTTTC AGGGTTCTGT

Orangutan AAATTGGATA TTGCAGCAGT CAGAGCCCTT AACCTTTTTC AGGGTTCTGT

Gorilla AAATTGGATA TTGCAGCAGT CAGAGCCCTT AACCTTTTTC AGGGTTCTGT

Marmoset AAATTGGATA TTGCAGCAGT CAGAGCCCTT AACCTTTTCC AGGGTTCTGT

Guinea_Pig AAATTGGATA TTGCAGCAGT TAAAGCCCTT AATCTATTCC AGGGTTCTGT

Mouse AAGTTGGACA TGGCAGCAGT TAGAGCCCTC AACCTTTTCC AGGGTTCTGT

Rat AAGTTAGACA TGGCGGCAGT TAGAGCCCTC AACCTTTTCC AGGGTTCTGT

Cow AAATTGGATA TTGCAGCAGT GAGAGCCCTT AACCTTTTCC AGGGTTCTGT

Dog AAGTTGGATA TTGCAGCAGT CCGAGCCCTT AACCTTTTCC AGGGTTCTGT

Rabbit AAATTGGATA TTGCAGCTGT CAGAGCCCTT AACCTGTTTC AGGGCTCTGT

Horse AAATTGGATA TTGCAGCAGT CAGAGCCCTT AACCTTTTCC AGGGTTCTGT

Elephant AAATTGGATA TTGCCGCAGT CCGAGCCCTT AACCTTTTCC AGGGTTCTCT

Chicken GTTTTAGATA ACGCAGCTGT CCAAGCCCTC AACCTGTTCC AGAGTTCTGT

Zebra_Finch GTTCTAGACA ATGCAGCTGT TCAAGCCCTC AACCTTTTTC AGAGTTCTGT

Zebrafish CGCCTGGATA ATGCTGCTGT ACAGGCTCTC AATCTCTTTC AGGGTTCCTC

Frog AAGCTAGATA ACGCAGCTGT GGGTGCGCTC AACCTTTTCC CTGTAAGTGT

Fugu AGGCTGGACA ACGCTGCCGT CCGGGCTCTG AACCTGTTCC AGGGATCTCC

Human TGAAGATACC ACTGGCTCTC AGTCTCTGGC TGCCTTGCTG AATAAGTGTA

Chimpanzee TGAAGATACC ACTGGCTGTC AGTCTCTGGC TGCCTTGCTG AATAAGTGTA

Orangutan TGAAGATACC ACTGGCTCTC AGTCTCTGGC TGCCTTGCTG AATAAGTGTA

Gorilla TGAAGATACC TCTGGCTCTC AGTCTCTGGC TGCCTTGCTG AATAAGTGTA

Marmoset TGAAGATACC ACTGGCTCTC AGTCTCTGGC TGCCTTGCTG AATAAGTGTA

Guinea_Pig CGAAGATACC ACTGGCTCTC AGTCTCTGGC TGCATTGCTG AATAAATGCA

Mouse TGAAGACACC ACTGGCTCTC AGTCTCTGGC CGCATTATTG AATAAATGCA

Rat TGAAGACACC ACAGGCTCTC AGTCTCTGGC TGCATTATTG AATAAATGCA

Cow TGAAGATACT TCTGGTTCTC AGTCTCTGGC TGCATTGCTC AATAAATGCA

Dog TGAAGATACT ACTGGCTCTC AGTCTCTGGC TGCATTGCTG AATAAATGCA

Rabbit TGAGGATACT AGTGGCTCTC AGTCTCTGGC TGCGTTGCTA AATAAATGCA

Horse TGAAGATACC ACTGGCTCCC AGTCTCTGGC TGCATTGCTG AATAAGTGCA

Elephant TGAAGATACC ACAGGCTCTC AGTCTCTGGC CGGATTGCTG AATAAATGCA

Chicken GGAAAATGCT AATAATACAC AGTCTCTGGC TGGTTTACTA AATAAATGCA

Zebra_Finch GGAAAATGCA AATACTGCAC AGTCTCTAGC TGGTTTACTG AATAAATGCA

Zebrafish TGATGATGCT ACAGGCACTC ACTCTCTGGC TGGACTTTTG AACAAATGTC

Frog CACACCCATC ATTCATAGCC AGGGACTGAT CTCATTGCTT TTGGGAGCAA

Fugu TGACGACTCC GCTGGAACTC ACTCATTGGC CGGACTCCTG AACAAGTGCC

Human AAACCCCTCA AGGACAAAGA CTTGTTAACC AGTGGATTAA GCAGCCTCTC

Chimpanzee AAACCCCTCA AGGACAAAGA CTTGTTAACC AGTGGATTAA GCAGCCTCTC

Orangutan AAACCCCTCA AGGACAAAGA CTTGTTAACC AGTGGATTAA ACAGCCTCTC

Gorilla AAACCCCTCA AGGACAAAGA CTTGTTAACC AGTGGATTAA GCAGCCTCTC

Marmoset AAACCCCTCA AGGACAAAGA CTTGTTAACC AGTGGATTAA GCAGCCCCTT

Guinea_Pig AAACTCCTCA AGGACAGAGG CTAGTTCACC AATGGATTAA GCAGCCTCTC

Mouse AAACTGCTCA AGGACAAAGA TTGGTTAACC AGTGGATCAA GCAGCCGCTC

Rat AAACTGCTCA AGGACAAAGG CTGGTTAGCC AGTGGATCAA ACAGCCGCTC

Cow AAACTCCTCA AGGACAAAGA CTGGTTAACC AGTGGATTAA GCAGCCTCTT

Dog AAACCCCTCA GGGACAAAGA CTGGTTAACC AGTGGATTAA GCAGCCTCTC

Rabbit AAACCCCTCA AGGACAACGA CTGGTTAACC AATGGATTAA GCAGCCTCTC

Horse AAACCCCTCA AGGACAAAGA CTGGTTAACC AGTGGATTAA GCAGCCGCTC

Elephant AAACCCCTCA AGGACAAAGA TTGGTTAATC AGTGGATTAA GCAGCCGCTC

Chicken GAACCCCTCA AGGACAAAGA CTAGTTAATC AGTGGATCAA ACAGCCACTT

Zebra_Finch GAACGCCTCA AGGACAAAGA CTAGTCAATC AGTGGATCAA GCAACCACTT

Zebrafish GGACTCCACA AGGACAGCGA CTGGTCAACC AATGGATCAA ACAGCCTCTC

Frog ATAACCTTAG AGATATGAAA TTGCTCTCTT CTTGGCTCAG ACAGCCTCTT

Fugu GCACGCCGCA GGGCCAGAGG CTGCTGCAGC AGTGGATCAA GCAGCCGCTG

Human ATGGATAAGA ACAGAATAGA GGAGAGATTG AATTTAGTGG AAGCTTTTGT

Chimpanzee ATGGATAAGA ACAGAATAGA GGAG------ ---------- ----------

Orangutan ATGGATAAGA ACAGAATAGA GGAGAGATTG AATTTAGTGG AAGCTTTTGT

Gorilla ATGGATAAGA ACAGAATAGA GGAGAGATTG AATTTAGTGG AAGCTTTTGT

Marmoset TTGGATAAGA ACAGAATAGA GGAGAGATTG AATTTAGTGG AAGCTTTTGT

Guinea_Pig ATGGATAAGA ACAGAATAGA GGAGAGATTA AATTTAGTGG AGGCTTTTGT

Mouse ATGGATAGGA ACAGGATAGA GGAGAGGTTA AATTTAGTGG AAGCTTTTGT

Rat ATGGATAAAA ACAGGATTGA AGAGAGATTA AATTTAGTGG AAGCTTTTGT

Cow ATGGACAAGA ACAGAATAGA AGAGAGATTA AATTTGGTAG AAGCTTTTGT

Dog ATGGATAAGA ACAGAATAGA AGAGAGACTG AATTTAGTGG AAGCTTTTGT

Rabbit ATGGATAAGA ACAGAATAGA AGAG------ ---------- ----------

Horse ATGGACAAGA ACAGAATAGA AGAGAGATTA AATTTAGTGG AAGCTTTTGT

Elephant ATGGATAAGA ACAGAATAGA AGAGAGATTA AATTTAGTGG AAGCCTTTGT

Chicken ATGGACAAGA ATAGAATTGA AGAAAGGTTG AATTTGGTTG AAGCCTTTGT

Zebra_Finch ATGGACAAGA CCAGAATTGA AGAAAGGTTG AATTTGGTTG AAGCTTTTGT

Zebrafish ATTGACAAAA ACAAGATAGA AGAAAGACTG GACCTTGTGG AGACATTTGT

Frog ATGGACAAGA ACAGAATTGA AGAAAGGTTG AACCTAGTTG AAGCTTTTGT

Fugu CTGGACAGAA CCAGGATCGA GGAGAGGCTG GACCTGGTGG AGAGCTTGGT

Human AGAAGATGCA GAATTGAGGC AGACTTTACA AGAAGATTTA CTTCGTCGAT

Chimpanzee ---------- ---------- ---------- ---------- ----------

Orangutan AGAAGATGCA GAATTGAGGC AGACTTTACA AGAAGATTTA CTTCGTCGAT

Gorilla AGAAGATGCA GAATTGAGGC AGACTTTACA AGAAGATTTA CTTCGTCGAT

Marmoset AGAAGATGCA GAATTGAGGC AAACTTTACA AGAAGATTTA CTTCGTCGAT

Guinea_Pig AGAAGATGCA GAATTGCGGC AGAGTTTACA AGAGGATTTA CTCCGTCGAT

Mouse CGAGGATTCA GAACTGAGGC AGAGTTTACA GGAGGATTTG CTTCGCCGGT

Rat TGAAGATTCA GAATTGAGGC AGAGTCTACA GGAGGATTTA CTTCGCCGAT

Cow AGAAGATGCA GAATTGAGGC AGAATTTACA AGAAGATTTA CTTCGTCGAT

Dog AGAAGATGCA GAATTGAGGC AGAGTTTACA AGAAGATTTA CTTCGTCGAT

Rabbit ---------- ---------- ---------- ---------- ----------

Horse AGAAGATGCA GAATTGAGAC AAAGTTTACA AGAAGATTTA CTTCGTCGAT

Elephant AGAAGACGCA GAATTGAGGC AAATCTTACA AGAAGATTTA CTTCGTCGAT

Chicken GGTAGATACT GAACTGCGTC AGGGCCTTCA AGAAGACCTT TTACGCCGCT

Zebra_Finch GATGGATCCA GAGCTACGTC AGTGCCTTCA AGAAGATCTG TTACGACGCT

Zebrafish GGAGGACTCG GAGCTAAGGA AAAGTTGTCA AGAGGATCTA CTTCGTCGCT

Frog GACGGACGCA GAACTAAGAC AGTGCCTTCA GGAAGATCTG CTCCGTCGCT

Fugu GGGCGACTCG GAGCTCAGGC AGACGTGCCA GGAGGACCTG CTGCGGCGCT

Human TCCCAGATCT TAACCGACTT GCCAAGAAGT TTCAAAGACA AGCAGCAAAC

Chimpanzee ---------- ---------- ---------- ---------- ----------

Orangutan TCCCAGATCT TAACCGACTT GCCAAGAAGT TTCAAAGACA AGCAGCAAAC

Gorilla TCCCAGATCT TAACCGACTT GCCAAGAAGT TTCAAAGACA AGCAGCAAAC

Marmoset TCCCAGATCT TAACCGACTT GCCAAGAAGT TTCTAAGACA AGCAGCAAAC

Guinea_Pig TCCCAGATCT TAACCGTCTT GCCAAGAAAT TTCAGAGACA AGCTGCAAAC

Mouse TTCCAGACCT TAACCGGCTT GCCAAGAAAT TCCAGAGACA AGCAGCGAAT

Rat TTCCAGATCT TAACCGGCTT GCCAAGAAAT TTCAGAGACA AGCAGCGAAT

Cow TTCCAGATCT TAACCGACTT GCCAAGAAAT TTCAAAGACA AGCGGCAAAT

Dog TCCCAGATCT TAACCGACTT GCCAAGAAAT TTCAGAGACA AGCAGCAAAC

Rabbit ---------- ---------- ---------- ---------- ----------

Horse TCCCAGATCT TAACCGACTT GCCAAGAAAT TTCAAAGACA AGCAGCAAAC

Elephant TCCCAGATCT GAACCGACTT GCTAAGAAAT TTCAAAGACA AGCAGCAAAC

Chicken TCCCAGATCT TAACCGGCTA GCAAAGAAAT TTCAGAGGCA AGCAGCAACT

Zebra_Finch TTCCAGACCT CAACCGGCTG GCAAAGAAAT TTCAGAGACA AGCAGCAAAC

Zebrafish TTCCTGACTT AAATCGGATG GCAAAGAAGT TCCAGCGTCA GAGCTCCAAT

Frog TTCCAGATCT TAATCGCTTA GCGAAAAAAT TCCAGAGACA GACTGCTAAC

Fugu TCCCCGACCT CCACCGTCTC TCCAGGAAGT TCCAGCGCCA CAGTGCCACG

Human TTACAAGATT GTTACCGACT CTATCAGGGT ATAAATCAAC TACCTAATGT

Chimpanzee ---------- ---------- ---------- ---------- ----------

Orangutan TTACAAGATT GTTACCGACT CTATCAGGGT ATAAATCAAC TACCTAATGT

Gorilla TTACAAGATT GTTACCGACT CTATCAGGGT ATAAATCAAC TACCTAATGT

Marmoset TTACAAGATT GTTACCGACT CTATCAGGGT ATAAATCAAC TCCCTAATGT

Guinea_Pig TTACAAGATT GTTACCGACT CTACCAGGGC ATAAATCAAC TCCCTAATGT

Mouse TTACAAGACT GTTACCGACT GTATCAGGGT ATTAACCAGC TCCCCAGCGT

Rat TTACAAGATT GTTACCGACT GTATCAGGGT GTGAACCAGC TTCCCAACGT

Cow TTACAAGATT GTTACCGACT CTATCAGGGT ATAAATCAAC TCCCTAATGT

Dog TTACAAGATT GTTACCGACT CTATCAGGGT ATAAATCAAC TCCCTAATGT

Rabbit ---------- ---------- ---------- ---------- ----------

Horse TTACAAGATT GCTATCGACT CTATCAGGGT ATAAATCAGC TCCCTAATGT

Elephant TTACAAAACT GTTACCAAGT CTACCAGGGT GTAAATCAAC TCCCTAACGT

Chicken CTACAGGACT GTTACCGAAT GTATCAAGCT ATTAACCAAC TGCCTAATGT

Zebra_Finch TTACAAGACT GTTACCGAAT GTTCCAGGCT ATCAATCAAC TGCCTAATGT

Zebrafish TTACAGGACT GTTACCGGGT CTATCAATCT GTGGGGCAAC TGCCCAATGT

Frog CTGCAGGATT GCTATAGACT TTACCAGGCT GTGAATCAGC TTCCAGGTGT

Fugu CTGCAGGACT GCTACCGCGT CTACCAAGCC GTGCACCACG TCGCTGCCCT

Human TATACAGGCT CTGGAAAAAC ATGAAGGAAA ACACCAGAAA TTATTGTTGG

Chimpanzee ---------- ---------- --------AA ACACCAGAAA TTATTGTTGG

Orangutan TATACAGGCT CTGGAAAAAC ATGAAGGAAA ACACCAGAAA TTATTGTTGG

Gorilla TATACAGGCT CTGGAAAAAC ATGAAGGAAA ACACCAGAAA TTATTGTTGG

Marmoset TATACAGGCT CTGGAAAAAT ATGAAGGAAA ACACCAGAAA TTGTTGTTGG

Guinea_Pig TATCCAGGCC CTGGAAAAAT ATGAAGGAAG ACATCAGGCA TTGCTGTTGG

Mouse CATCCAGGCT CTGGAGAAAT ACGAAGGAAG ACACCAGGCA CTGTTGTTGG

Rat CATCCAGGCT CTGGAGAAAT ACCAAGGAAG ACACCAGGCA TTGTTGTTGG

Cow TATTCAGGCT TTGGAAAAAT ATGAAGGAAA ACATCAGGCA TTGTTTCTGG

Dog TATTCGGGCT CTGGAGAAAT ATGAAGGAAA ACACCAAGGT TTATTGTTGG

Rabbit ---------- ATTGACAAGT ATGAGGGAAA GCACCAGAAC TTGTTGCTGG

Horse TATACAGGCT CTGGAAAAAT ATGAAGGGAA ACACCAGACA TTGTTGTTGG

Elephant TATACAGGCT CTGGAAAAAT ATGAAGGAAA ACACCAGTCC TTGTTGTTGG

Chicken TGTGCAAGCA CTAGAAAAAC ATGAAGGAGC TCACCAGATG TTGTTGTTGG

Zebra_Finch TGTACAAGCG CTAGAAAAAC ATGAAGGAGC TCACCAGATG TTGTTGTTGG

Zebrafish GGTGCTGGCA CTGGAGAGAT ACAGTGGAAA ACATCAGGTC CTCCTGCATG

Frog TATACAAGCA ATTGAAAAAT ATGAAGGGAC CCATCAGATG CTACTGCTGG

Fugu CCTCTCAGCC CTGGACCGAC ACGCAGGACG CCACCAGGCG CTGATGGACG

Human CAGTTTTTGT GACTCCTCTT ACTGATCTTC GTTCTGACTT CTCCAAGTTT

Chimpanzee CCGTTTTTGT GACTCCTCTT ACTGATCTTC GTTCTGACTT CTCCAAGTTT

Orangutan CAGTTTTTGT GACTCCTCTT ACTGATCTTC GTTCTGATTT CTCCAAGTTT

Gorilla CAGTTTTTGT GACTCCCCTT ACTGATCTTC GTTCTGACTT CTCCAAGTTT

Marmoset CAGTTTTTGT GACTCCTCTT ACTGATCTTC GTTCTGATTT CTCCAAGTTT

Guinea_Pig CAGTGTTTGC AGCTCCTCTT GTTGATCTTC GTTCTGATTT CTCCAAGTTT

Mouse CAGTTTTTGT GACTCCTCTT ATTGATCTTC GTTCTGATTT TTCAAAATTT

Rat CAGTTTTTGT GACTCCTCTT ACTGATCTTC GTTCTGATTT TTCAAAATTT

Cow CAGTTTTTGT GACTCCTCTT ATTGATCTTC GTTCTGACTT CTCCAAGTTT

Dog CAGTTTTTGT CACTCCTCTT ATTGATCTTC GTTCTGATTT CTCCAAATTT

Rabbit CAGTTTTTGT GACTCCTCTT ACTGATCTTC GTTCTGATTT CTCCAAGTTT

Horse CAGTTTTTGT GACTCCTCTT ATTGATCTTC GTTCTGATTT CTCCAAGTTT

Elephant CCATTTTTGT GACCCCTCTT GTCGATCTTC GTTCCGATTT CTCCAAGTTT

Chicken CAGTGTTTAT AACACCTCTT AATGATATCC ACTCCGACTT CTCAAAATTT

Zebra_Finch CTGTATTTAT AACACCTCTT AATGATATCT GCTCTGACTT CTCAAAGTTT

Zebrafish CAGCATTCAT CTCTCCACTA AACGACCTCA TCTCAGACTT CTCCAAATTT

Frog CGGTATTTGC GACACCCCTT TCTGATCTTT CATCAGACTT CTCAAAATTT

Fugu CCGTCTTCAT CTCCCCCCTC AGAGACCTAC AGAGCGACTT TGGGAAGTAC

Human CAGGAAATGA TAGAAACAAC TTTAGATATG GATCAGGTGG AAAACCATGA

Chimpanzee CAGGAAATGA TAGAAACAAC TTTAGATATG GATCAGGTGG AAAACCATGA

Orangutan CAGGAAATGA TAGAAACAAC TTTAGATATG GATCAGGTGG AAAACCATGA

Gorilla CAGGAAATGA TAGAAACAAC TTTAGATATG GATCAGGTGG AAAACCATGA

Marmoset CAGGAAATGA TTGAAACAAC TTTAGATATG GATCAGGTGG AAAACCATGA

Guinea_Pig CAGGAAATGA TCGAAACAAC TTTAGATATG GATCAGGTAG AAAACCATGA

Mouse CAAGAAATGA TAGAAACAAC TTTAGATATG GATCAGGTGG AAAACCACGA

Rat CAAGAAATGA TAGAAACGAC TTTAGATATG GATCAGGTGG AGAACCACGA

Cow CAGGAAATGA TAGAAACAAC TTTAGATATG GATCAGGTGG AAAACCATGA

Dog CAGGAAATGA TTGAAACAAC TCTAGATATG GATCAGGTGG AAAACCATGA

Rabbit CAGGAAATGA TAGAAACAAC TTTAGACATG GATCAGGTGG AAAACCATGA

Horse CAGGAAATGA TAGAAACAAC TTTAGATATG GATCAGGTGG AAAACCATGA

Elephant CAGGAAATGA TAGAAACAAC TTTAGACATG GATCAGGTGG AAAACCATGA

Chicken CTGGAGATGA TAGAAACAAC CCTGGATATG GATAAGGTGG AGAATCATGA

Zebra_Finch CTGGAGATGA TAGAAACAAC GTTGGACATG GAAAAGGTGG AGAATCATGA

Zebrafish CAGGAGATGA TTGAAACCAC ACTGGACATG AACCAGGTTG AGCATCACGA

Frog CAAGAAATGA TTGAAACCAC ATTGGATATG GATCAGGTGG AAAATCATGA

Fugu CAGGAGATGA TTGAAACCAC TCTGGACATG AACCAGGTGG AGCACCACGA

Human ATTCCTTGTA AAACCTTCAT TTGATCCTAA TCTCAGTGAA TTAAGAGAAA

Chimpanzee ATTCCTTGTA AAACCTTCAT TTGATCCTAA TCTCAGTGAA TTAAGAGAAA

Orangutan ATTCCTTGTA AAACCTTCAT TTGATCCTAA TCTCAGTGAA TTAAGAGAAA

Gorilla ATTCCTTGTA AAACCTTCAT TTGATCCTAA TCTTAGTGAA TTAAGAGAAA

Marmoset ATTCCTTGTA AAACCTTCAT TTGATCCTAA TCTCAGTGAA TTAAGAGAAA

Guinea_Pig ATTCCTTGTA AAACCTTCTT TTGATCCGAA TCTGAGTGAA CTAAGAGAAG

Mouse GTTCCTTGTA AAACCTTCAT TTGATCCTAA CCTGAGTGAA CTAAGAGAAG

Rat ATTCCTTGTA AAACCTTCAT TTGATCCTAA CCTGAGTGAG CTAAGAGAAG

Cow ATTCCTTGTA AAACCTTCAT TTGATCCTAA CTTGAGTGAA TTAAGAGAAA

Dog ATTCCTTGTA AAACCTTCAT TTGATCCCAA TCTGAGTGAA TTAAGGGAAA

Rabbit ATTCCTTGTG AAACCTTCCT TTGATCCTAA TCTGAGTGAG TTAAGAGAAG

Horse ATTCCTTGTA AAACCTTCAT TTGATCCTAA TCTGAGTGAA TTAAGAGAAA

Elephant ATTCCTTGTA AAACCCTCTT TTGATCCTAA TCTGACTGAA CTAAGAGAAG

Chicken GTTCCTGGTT AAGGCTTCTT TCGACCCCAA CCTGACAGAA CTGCGAGAGA

Zebra_Finch ATTTCTTGTC AAGGCTTCTT TTGATCCTAA TTTAACAGAA TTGAGAGAGA

Zebrafish GTTTCTGGTG AAACCTTCCT TTGATCCAAC TTTGAGTGAT CTCAGGGAAA

Frog ATTTCTTGTT AAGGCCTCAT TCGATCCGAA CCTGACAGAA TTGAGAGAGA

Fugu GTTCCTGATC AAAGCTTCCT TTGATCCGGC GTTGAGTGAC CTGAGATCAA

Human TAATGAATGA CTTGGAAAAG AAGATGCAGT CAACATTAAT AAGTGCAGCC

Chimpanzee TAATGAATGA CTTGGAAAAG AAGATGCAGT CAACATTAAT AAGTGCAGCC

Orangutan TAATGAATGA CTTGGAAAAG AAGATGCAGT CAACATTAAT AAGTGCAGCC

Gorilla TAATGAATGA CTTGGAAAAG AAGATGCAGT CAACATTAAT AAGTGCAGCC

Marmoset TAATGAATGA CTTGGAAAAG AAGATGCAGT CAACATTAAT AAGTGCAGCC

Guinea_Pig TAATGGATGA CTTGGAAAAG AAGATGCAGT CCACGTTAAT AAGTGCAGCT

Mouse TCATGGATGG CCTGGAGAAG AAGATGCAGT CCACCTTAAT AAATGCAGCC

Rat TCATGGATGG TCTGGAGAAG AAGATGCAGT CCACCTTAAT AAGTGCAGCG

Cow TAATGGATGA CTTAGAAAAG AAGATGCAGT CAACATTAGT AAGTGCAGCT

Dog TAATGGATGA CTTAGAAAAG AAGATGCAGT CAACATTGAT AAGTGCAGCA

Rabbit TAATGGATGA CTTAGAGAAG AAGATGCAGT CAACACTAAT AAGTGCAGCC

Horse TAATGGATGA TTTAGAAAAG AAGATGCAGT CAACATTAAT AAGTGCAGCT

Elephant TGATGGATGA CTTGGAAAAG AAGATGCAGT CAACATTAAC AGGCGCAGCC

Chicken AGATGAATGA GCTGGAAGAA AGCATGCAGA CCTTATTGAA GAGTGCAGCC

Zebra_Finch AGATGAATGA ACTGGAAGAA AAAATGCAGT CCTTACTAAA GACTGCAGCC

Zebrafish ACATGGATCG TTTGGAGAAA GCCATGCAGG CTGCCTTGAG CAGCGCAGCT

Frog AGATGAATGA ACTAGAGAAG AATATGCAGG CTGCACTCAA TGGTGCTGCA

Fugu AGATGGACGA GATGGAGAAG AGCATGCAGG CCGTGCTGAG CAGTGCAGCC

Human AGAGATCTTG GCTTGGACCC TGGCAAACAG ATTAAACTGG ATTCCAGTGC

Chimpanzee AGAGATCTTG GCTTGGACCC TGGCAAACAG ATTAAACTGG ATTCCAGTGC

Orangutan AGAGATCTTG GCTTGGACCC TGGCAAACAG ATTAAACTGG ATTCCAGTGC

Gorilla AGAGATCTTG GCTTGGACCC TGGCAAACAG ATTAAACTGG ATTCCAGTGC

Marmoset AGAGATCTTG GCTTGGACCC TGGCAAACAG ATTAAACTGG ATTCCAGTGC

Guinea_Pig CGAGACCTGG GATTGGATCC TGGCAAGCAG ATTAAATTGG ACTCCAGTGC

Mouse CGGGGGCTCG GATTGGATCC TGGCAAACAG ATTAAATTGG ACTCCAGTGC

Rat CGGGGTCTTG GATTGGACCC TGGCAAACAG ATTAAATTGG ACTCCAGTGC

Cow AGAGATCTAG GCTTAGATCC TGGCAAGCAG ATTAAACTGG ATTCCAGCAC

Dog AGAGATCTAG GTTTGGATCC TGGCAAACAG ATTAAACTGG ATTCCAGTGC

Rabbit AGAGAGCTCG GCTTGGACCC TGGCAAGCAG ATTAAACTAG ATTCCAGTGC

Horse AGAGATCTCG GCTTAGATCC TGGGAAACAG ATTAAACTGG ATTCCAGCAC

Elephant AGAGACCTAG GCTTGGAGCC TGGCAAGCAG ATTAAACTGG ATTCCAGTGC

Chicken AAAGAGCTTG GTCTGGAAGC TGGTAAAAGT ATCAAACTGG AGTCAAATTC

Zebra_Finch AAAGAACTAG GTTTGGAAGC TGGCAAAAGT ATCAAACTGG AGTCAAACTC

Zebrafish CGGGAGCTTG GGTTGGAAGC AGCCAAGACT GTGAAGCTTG AATCCAATGC

Frog AGGGAGCTGG GTCTTGATGC TGGCAAAAGC ATTAAACTGG AATCAAACTC

Fugu AGAGAGCTGG GTCTGGACGC CGGTAAGACG GTGAAGCTGG AGTCCAACGC

Human ACAGTTTGGA TATTACTTTC GTGTAACCTG TAAGGAAGAA AAAGTCCTTC

Chimpanzee ACAGTTTGGA TATTACTTTC GTGTAACCTG TAAGGAAGAA AAAGTCCTTC

Orangutan TCAGTTTGGA TATTACTTTC GTGTAACCTG TAAGGAAGAA AAAGTCCTTC

Gorilla ACAGTTTGGA TATTACTTTC GTGTAACCTG TAAGGAAGAA AAAGTCCTTC

Marmoset ACAGTTTGGA TATTACTTTC GTGTAACCTG TAAGGAAGAA AAAGTCCTTC

Guinea_Pig ACAGTTTGGA TATTACTTTC GGGTAACCTG TAAGGAAGAA AAAGTCCTTC

Mouse ACAGTTTGGA TATTATTTCC GTGTAACCTG CAAGGAAGAG AAAGTGCTTC

Rat ACAGTTTGGG TATTACTTTC GTGTAACCTG CAAGGAAGAG AAAGTCCTCC

Cow ACAGTTTGGC TATTATTTCC GAGTAACATG CAAGGAAGAA AAAGTCCTTC

Dog ACAGTTTGGT TATTATTTTC GAGTAACATG TAAGGAAGAA AAAGTCCTTC

Rabbit ACAGTTTGGA TATTACTTTC GAGTAACCTG TAAGGAAGAA AAGGTCCTTC

Horse ACAATTTGGA TATTACTTTC GTGTAACATG TAAGGAAGAA AAAGTCCTTC

Elephant ACAGTTTGGA TATTACTTTC GAGTAACCTG TAAGGAAGAA AAAGTCCTTC

Chicken ACAGTTTGGA CACCACTTTC GAATTACTTG CAAGGAGGAA AAGGTTCTCA

Zebra_Finch GCAGTTTGGG CATCACTTTA GGGTAACTTG TAAGGAGGAA AAAGCTCTCC

Zebrafish ACAAATTGGT TATTTTTTCC GAGTTACGTG CAAAGAAGAA AAGAGTCTTC

Frog CCAAGTTGGC CACTTTTTCC GGGTAACATG CAAGGAGGAA AAGGCCTTAA

Fugu CGCGCTGGGG TTCTACCTGA GAGTCACCTG CAAGGAGGAG AAGGTGCTGA

Human GTAACAATAA AAACTTTAGT ACTGTAGATA TCCAGAAGAA TGGTGTTAAA

Chimpanzee GTAACAATAA AAACTTTAGT ACTGTAGATA TCCAGAAGAA TGGTGTTAAA

Orangutan GTAACAATAA AAACTTTAGT ACTGTAGATA TCCAGAAGAA TGGTGTTAAA

Gorilla GTAACAATAA AAACTTTAGT ACTGTAGATA TCCAGAAGAA TGGTGTTAAA

Marmoset GTAACAATAA AAACTTTAGT ACTGTAGATA TCCAGAAGAA TGGTGTTAAA

Guinea_Pig GTAACAATAA AAACTTCAGT ACTGTGGATA TCCAGAAAAA TGGTGTTAAA

Mouse GCAACAACAA GAACTTCAGC ACAGTGGACA TCCAGAAGAA TGGCGTGAAG

Rat GCAACAACAA GAACTTCAGC ACCGTGGATA TCCAGAAGAA TGGCGTTAAA

Cow GTAATAATAA AAATTTCAGT ACAGTAGATA TCCAGAAGAA TGGTGTTAAA

Dog GCAACAATAA AAACTTCAGT ACAGTAGATA TCCAGAAGAA TGGTGTTAAA

Rabbit GTAACAATAA AAACTTTAGT ACAGTAGATA TCCAGAAGAA TGGTGTGAAA

Horse GTAACAATAA AAACTTCAGT ACAGTAGATA TCCAGAAGAA TGGTGTTAAA

Elephant GTAATAATAA AAACTTCACC ATAGTAGATA TCCAGAAGAA TGGTGTTAAA

Chicken GGAACAACAT GAAGTACAAG ATAACTGATA CACAGAAGAA TGGTGTGAAG

Zebra_Finch GTAACAATTC AAAATATGGA ATCATTGATA CACAGAAGAA TGGTGTGAAA

Zebrafish GCAACAACAA GAAATTCACT ACTTTAGACG TTCAGAAGAA TGGAGTACGG

Frog GGAACAATAA GAAGTTCACT ACAATCGACA TACAGAAAAA TGGGGTCAGA

Fugu GGAACAACAA GAAGTTCACC ATGCTGGATG TTCAGAAGAA CGGCGTGCGC

Human TTTACCAACA GCAAATTGAC TTCTTTAAAT GAAGAGTATA CCAAAAATAA

Chimpanzee TTTACCAACA GCAAATTGAC TTCTTTAAAC GAAGAGTATA CCAAAAATAA

Orangutan TTTACCAACA GCAAATTGAC TTCTTTAAAT GAAGAGTATA CCAAAAATAA

Gorilla TTTACCAACA GCAAATTGAC TTCTTTAAAT GAAGAGTATA CCAAAAATAA

Marmoset TTTACCAACA GCAAATTGAC TTCTTTAAAT GAAGAATATA CCAAAAATAA

Guinea_Pig TTCACCAACA GTAAGTTGTC CTCTTTGAAT GAAGAGTATA CTAAAAATAA

Mouse TTTACCAACA GTGAATTGTC CTCTTTAAAT GAAGAATATA CTAAGAACAA

Rat TTCACCAACA GTGAATTGTC TTCTTTAAAT GAAGAATACA CTAAGAACAA

Cow TTTACAAACA GTAAACTGAC TTCTCTCAAT GAAGAGTACA CCAAAAATAA

Dog TTTACCAACA GCAAACTGAC TTCTCTCAAT GAAGAGTATA CCAAAAATAA

Rabbit TTTACTAACA GTAAATTGTC TTCTTTCAAT GAAGAATATG CTAAAAATAA

Horse TTTACCAACA GTAAATTGAC TTCTCTCAAT GAAGAGTATA CCAAAAATAA

Elephant TTTGCCAACA GTAAATTGAC TTCTCTCAAT GAAGAGTATA CCAAAAATAA

Chicken TTTACAAACA GCAAGCTGAG TGCCATCAAT GAGGAGTACA TAAAGAACAG

Zebra_Finch TTTACTAACA GCAAACTGAG CACCATCAAT GAAGAATATA TAAGGAACAG

Zebrafish TTCACTAATA GTAAGCTTAG CTCGTTGAAT GAAGAATACA CAAAAAGCAG

Frog TTTACTAACG GTAAACTGAG CTCACTTAGT GAAGAGTACA TGAGAAACAG

Fugu TTCACCAACG GGAAACTGAG CTCGATCAAC GAGGACTACA CCAAGAGCAG

Human AACAGAATAT GAAGAAGCCC AGGATGCCAT TGTTAAAGAA ATTGTCAATA

Chimpanzee AACAGAATAT GAAGAAGCCC AGGATGCCAT TGTTAAAGAA ATTGTCAATA

Orangutan AACAGAATAT GAAGAAGCCC AGGATGCCAT TGTTAAAGAA ATTGTCAATA

Gorilla AACAGAATAT GAAGAAGCCC AGGATGCCAT TGTTAAAGAA ATTGTCAATA

Marmoset AACAGAATAT GAAGAAGCCC AGGATGCCAT TGTTAAAGAA ATTGTCAATA

Guinea_Pig AGGCGAGTAT GAGGAAGCCC AGGATGCCAT TGTTAAGGAA ATCGTCAATA

Mouse AGGCGAGTAT GAAGAGGCCC AGGATGCCAT TGTTAAAGAA ATTGTCAATA

Rat AGGAGAGTAT GAGGAGGCCC AGGATGCCAT TGTTAAAGAA ATCGTCAATA

Cow AACTGAATAT GAGGAAGCCC AGAATGCCAT TGTTAAAGAA ATTGTCAATA

Dog AACCGAGTAC GAGGAAGCCC AGGATGCCAT TGTTAAAGAA ATTGTCAATA

Rabbit AGCTGAATAT GAGGAGGCCC AGGATGCCAT TGTTAAAGAA ATTGTCAATA

Horse AACTGAATAT GAGGAAGCCC AGGATGCCAT TGTTAAAGAA ATTGTCAATA

Elephant AACTGAGTAC GAGGAAGCCC AGAATGCCAT TGTGAAGGAA ATCATCAACA

Chicken AGAAGAGTAT GAAGAGGCTC AGGATGCAAT TGTTAAGGAA ATAATTAACA

Zebra_Finch GGAAGAGTAT GAAGAGGCTC AGGATGCAAT TGTTAAGGAA ATCATTAACA

Zebrafish AGAAGAGTAT GAAGAGGCTC AGAATGCCAT CGTCAAGGAA ATCATCAGCA

Frog AGAGGAGTAT GAGGAAGCAC AGAACGCTAT TGTTAAAGAA ATTATAAGTA

Fugu GGCGGAGTAC GAGGAGGCCC AGAACGCCAT CGTGAAGGAG ATCATCAACA

Human TTTCTTCAGG CTATGTAGAA CCAATGCAGA CACTCAATGA TGTGTTAGCT

Chimpanzee TTTCTTCAGG CTATGTAGAA CCAATGCAAA CACTCAATGA TGTGTTAGCT

Orangutan TTTCTTCAGG CTATGTAGAA CCAATGCAAA CACTCAATGA TGTGTTAGCT

Gorilla TTTCTTCAGG CTATGTAGAA CCAATGCAAA CACTCAATGA TGTGTTAGCT

Marmoset TTTCTTCAGG CTATGTAGAA CCAATGCAAA CACTCAATGA TGTGTTAGCT

Guinea_Pig TATCTTCAGG CTATGTAGAG CCAATGCAGT CACTGAATGA TGTGTTAGCT

Mouse TTTCCTCAGG CTACGTAGAG CCAATGCAGA CGCTCAACGA TGTGCTGGCT

Rat TTTCCTCAGG CTACGTGGAG CCAATGCAGA CGCTCAATGA CGTGCTAGCT

Cow TTTCTTCAGG CTACGTAGAA CCGATGCAGA CACTCAACGA TGTGTTAGCT

Dog TTTCTTCAGG CTATGTCGAA CCAATGCAGA CACTCAATGA TGTGTTAGCT

Rabbit TTTCTTCAGG CTATGTAGAA CCAATGCAGA CCCTCAACGA TGTGCTAGCT

Horse TTTCTTCAGG CTATGTAGAA CCAATGCAGA CGCTCAACGA TGTGCTGGCT

Elephant TTTCTTCAGG CTACGTAGAA CCGATGCAGA CCCTCAACGA TGTGTTAGCT

Chicken TCGCTTCAGG TTATGCAGAG CCAATACAGA CCATGAATGA TGTCATAGCT

Zebra_Finch TTGCTTCAGG TTATGCAGAG CCCATACAGA CCATGAATGA TGTGATAGCT

Zebrafish TTGCTGCAGG TTATGTGGAT CCAGTACAGA CTCTGAATGA GGTGATCGCT

Frog TTTCTGCTGG ATATGTGGAT CCAATTCAGA CTCTGAATGA CGTCATTGCA

Fugu TCGCCGCAGG CTACGTGGAC CCCCTGCAGG CACTGAGTGA TGTCACGGCC

Human CAGCTAGATG CTGTTGTCAG CTTTGCTCAC GTGTCAAATG GAGCACCTGT

Chimpanzee CAGCTAGATG CTGTTGTCAG CTTTGCTCAT GTGTCAAATG GAGCACCTGT

Orangutan CAGCTAGATG CTGTTGTCAG CTTTGCTCAT GTGTCAAATG GAGCACCAGT

Gorilla CAGCTAGATG CTGTTGTCAG CTTTGCTCAT GTGTCAAATG GAGCACCTGT

Marmoset CAACTAGATG CTGTTGTCAG TTTTGCTCAT GTGTCAAATG GAGCACCTGT

Guinea_Pig CAGCTAGATG CTGTTGTCAG CTTTGCTCAT GTGTCAAATG GCGCACCTGT

Mouse CACTTAGACG CCATTGTTAG CTTCGCTCAT GTGTCAAACG CAGCACCCGT

Rat CACTTAGATG CCGTCGTTAG TTTTGCTCAT GTGTCAAACG CAGCTCCTGT

Cow CAGCTGGATG CTGTTGTCAG CTTTGCTCAC GTGTCCGATG CAGCACCTGT

Dog CAGCTAGACG CTGTTGTCAG CTTCGCTCAT GTGTCAAATG GAGCACCTGT

Rabbit CAGCTTGATG CTGTTGTCAG CTTTGCTCAT GCGTCCAATG GAGCACCTGT

Horse CAGCTAGATG CTGTTGTCAG CTTTGCTCAC GTGTCAAATG GAGCGCCTGT

Elephant CAGCTGGATG CCGTCGTCAG CTTTGCTCAC GTCTCAAATG GAGCCCCTGT

Chicken CAGTTAGATG CCATTGTCAG CTTTGCTCAT GTGTCAAACG GAGCACCAGT

Zebra_Finch CAGCTGGATG CCATCGTCAG CTTTGCCCAT GTGTCCAACG GAGCCCCCGT

Zebrafish CAGCTGGATG CAGTGGTAAG CTTCGCTGTG GTGTCACATG CTGCCCCAGT

Frog CAGTTGGACG CTGCGGTCAG CTTTGCCTAC GTTTCTAACA GCGCACCAGT

Fugu CAGCTGGACG CCGTGGTGAG CTTCGCCGTG GCGTCCGTGT CGGCCCCGGT

Human TCCATATGTA CGACCAGCCA TTTTGGAGAA AGGACAAGGA AGAATTATAT

Chimpanzee TCCATATGTA CGACCAGCCA TTTTGGAGAA AGGACAAGGA AGAATTATAT

Orangutan TCCGTATGTA CGACCAGCCA TTTTGGAGAA AGGACAAGGA AGAATTATAT

Gorilla TCCATATGTA CGACCAGCTA TTTTGGAGAA AGGACAAGGA AGAATTATAT

Marmoset TCCATATGTA CGACCAGTCA TTTTGGAGAA AGGACAAGGA AGAATTATTT

Guinea_Pig TCCATACGTA CGACCAGTTA TTTTGGAGAA AGGACAAGGA AGAATCATAC

Mouse TCCTTATGTA CGACCAGTCA TCTTGGAGAA AGGAAAAGGG AGAATTATAT

Rat CCCCTACGTG CGACCGGTCA TCTTGGAGAA AGGAAAAGGA AGAATTATAT

Cow TCCATATGTA CGGCCGGTGA TTTTGGAAAA AGGACGAGGC AGAATTACAC

Dog TCCATATGTA CGACCGGTCA TTTTGGAAAA AGGACAAGGA AGAATTACAT

Rabbit TCCATATGTA CGACCAGTCA TTTTGGAGAA AGGGCAAGGA AGAATTATAT

Horse TCCGTATGTG CGACCAGTCA TTTTGGAGAA AGGACAAGGA AGAATTACAC

Elephant TGCGTACGTA CGACCGGTCA TTTTGGAGAA GGGACGAGGA AGAATTCTGT

Chicken GCCATATGTT CGTCCTGTCA TTCTGGAAAA GGGGCAAGGA AGGATTGTGC

Zebra_Finch GCCCTACGTC CGGCCCGTCG TTCTGGAGAA GGGGCAGGGC AGGATTGTGC

Zebrafish GCCTTTCATA AGACCAAAAA TACTAGAGAA GGGTTCAGGC AGACTCGTTC

Frog ACCGTATGTC CGGCCCGTCA TTTTGGAAAA GGGACAGGGA AAAATTATGC

Fugu GCCCTACGTC CGGCCGGAGC TGCTGGGCGA GGGGCCCAGA CGCGTGGCGC

Human TAAAAGCATC CAGGCATGCT TGTGTTGAAG TTCAAGATGA AATTGCATTT

Chimpanzee TAAAAGCATC CAGGCATGCT TGTGTTGAAG TTCAAGATGA AATTGCATTT

Orangutan TAAAAGCATC CAGGCATGCT TGTGTTGAAG TTCAAGATGA AATCGCATTT

Gorilla TAAAAGCATC CAGGCATGCT TGTGTTGAAG TTCAAGATGA AATTGCATTT

Marmoset TGAAAGCATC CAGACATGCT TGTGTTGAAG TTCAAGATGA AGTTGCATTT

Guinea_Pig TGAAAGCATC CAGACATGCT TGTGTCGAAG TTCAAGATGA AGTTGCATTT

Mouse TGAAAGCCTC CAGGCATGCT TGTGTTGAAG TTCAAGATGA AGTTGCATTT

Rat TGAAAGCCTC CAGGCATGCT TGTGTTGAAG TTCAAGATGA CGTTGCATTT

Cow TGAAAGCTTC CAGACATGCT TGTGTGGAAG TTCAAGATGA AGTCGCATTT

Dog TGAAAGCATC CAGACATGCT TGTGTTGAAG TTCAAGATGA AGTTGCATTT

Rabbit TGAAAGCATC CAGACATGCT TGTGTTGAAG TTCAAGATGA AGTTGCATTT

Horse TGAGAGCATC CAGACATGCT TGTGTTGAAG TTCAAGATGA AGTTGCATTC

Elephant TGACAGCATC TAGACATGCT TGTGTTGAAG TTCAAGATGA AGTTGCCTTT

Chicken TGAAAGGTGC CAGGCATCCT TGCATTGAAG TGCAAGATGA GGTGGCCTTC

Zebra_Finch TGAAAGGCGC CAGGCACCCC TGCATCGAAG TGCAGGACGA GGTGGCCTTC

Zebrafish TGAAGGCAGC AAGACATCCC TGTGTTGAAG CCCAGGATGA AGTGGCTTTC

Frog TCCAGTCGGC CAGACACCCA TGCATTGAGA TGCAAGATGA TGTGGCGTTC

Fugu TGCTGCAGGC CAGACACCCC TGCATGGAGG CGGACGCAGA CACGGCCTTC

Human ATTCCTAATG ACGTATACTT TGAAAAAGAT AAACAGATGT TCCACATCAT

Chimpanzee ATTCCTAATG ACGTATACTT TGAAAAAGAT AAACAGATGT TCCACATCAT

Orangutan ATTCCTAATG ACGTATACTT TGAAAAAGAT AAACAGATGT TCCACATCAT

Gorilla ATTCCTAATG ACGTATACTT TGAAAAAGAT AAACAGATGT TCCACATCAT

Marmoset ATTCCTAATG ATGTATACTT TGAAAAAAAT AAACAGATGT TCCACATCAT

Guinea_Pig ATTCCTAATG ATGTGCATTT TGAAAAAGAC AAACAGATGT TTCACATCAT

Mouse ATTCCAAATG ACGTGCACTT TGAAAAAGAT AAACAGATGT TCCACATCAT

Rat ATTCCAAACG ATGTGCACTT TGAAAAAGAT AAACAGATGT TCCACATCAT

Cow ATTCCTAATG ATGTTCACTT TGAAAAAGAT AAACAGATGT TCCACATCAT

Dog ATTCCTAATG ATGTTCACTT TGAAAAAGAT AAACAGATGT TCCACATCAT

Rabbit ATTCCTAATG ATGTGCACTT TGAAAAAGAT AAACAGATGT TCCACATCAT

Horse ATTCCTAATG ATGTTCACTT TGAAAAAGAT AAACAGATGT TCCACATCAT

Elephant ATTCCCAATG ATGTTCACTT TGAGAAAGAT AAACAGATGT TCCACATCAT

Chicken ATCCCCAATG ACGTTACGTT TGAGAAGGGC AAGCAGATGT TCCACATTAT

Zebra_Finch ATCCCCAATG ACATCACCTT CGAGAAGGGC AAGCAGATGT TCCACATTAT

Zebrafish ATTCCCAATG ATGTCACCTT CATAAGAGGA GAGAAGATGT TTCATATCAT

Frog ATTCCGAATG ATATAACATT TGAAAAGGAA AAGCAGATGT TTCATATTAT

Fugu ATCCCCAACG ACATCACCTT CGTCCAGGGA CAGAAGAGCT TCTACATCAT

Human TACTGGCCCC AATATGGGAG GTAAATCAAC ATATATTCGA CAAACTGGGG

Chimpanzee TACTGGCCCC AATATGGGAG GTAAATCAAC ATATATTCGA CAGACTGGGG

Orangutan TACTGGCCCC AATATGGGAG GTAAATCAAC ATATATTCGA CAAACTGGGG

Gorilla TACTGGCCCC AATATGGGAG GTAAATCAAC ATATATTCGA CAAACTGGGG

Marmoset TACTGGCCCC AATATGGGAG GTAAATCAAC ATATATTCGA CAAACTGGGG

Guinea_Pig TACTGGCCCC AATATGGGAG GTAAATCAAC ATATATTCGC CAGACTGGGG

Mouse TACTGGTCCC AATATGGGAG GTAAATCAAC ATACATTCGT CAGACCGGGG

Rat TACTGGTCCC AATATGGGAG GAAAGTCAAC ATACATCCGT CAGACGGGGG

Cow TACTGGTCCC AATATGGGAG GGAAATCAAC ATATATTCGC CAGACTGGGG

Dog TACTGGCCCC AATATGGGAG GTAAATCAAC ATATATTCGC CAAACTGGGG

Rabbit TACTGGTCCC AATATGGGAG GCAAATCAAC GTATATTCGC CAAACTGGGG

Horse TACTGGCCCC AATATGGGAG GTAAATCAAC ATATATTCGC CAAACGGGGG

Elephant TACTGGCCCC AATATGGGAG GTAAATCAAC ATATATTCGC CAAACCGGGG

Chicken TACTGGCCCG AACATGGGTG GAAAGTCAAC ATATATCCGA CAGACAGGAG

Zebra_Finch TACTGGCCCT AACATGGGGG GAAAATCAAC GTACATCCGA CAGACAGGGG

Zebrafish CACAGGTCCA AATATGGGTG GTAAATCTAC CTACATCCGT CAAGTGGGTG

Frog TACTGGTCCT AACATGGGAG GGAAATCGAC ATATATCCGC CAGACTGGTG

Fugu CACAGGGCCA AATATGGGCG GCAAGTCCAC GTTCATCCGG CAGGTGGGCG

Human TGATAGTACT CATGGCCCAA ATTGGGTGTT TTGTGCCATG TGAGTCAGCA

Chimpanzee TGATAGTACT CATGGCCCAA ATTGGGTGTT TTGTGCCATG TGAGTCAGCA

Orangutan TGATAGTACT CATGGCCCAA ATTGGGTGTT TTGTGCCATG TGAGTCAGCA

Gorilla TGATAGTACT CATGGCCCAA ATTGGGTGTT TTGTGCCATG TGAGTCAGCA

Marmoset TGATAGTACT TATGGCCCAA ATTGGGTGTT TTGTGCCATG TGAGTCAGCG

Guinea_Pig TGATAGTACT TATGGCCCAA ATAGGCTGTT TTGTGCCATG CGAGTCAGCA

Mouse TGATTGTACT CATGGCCCAA ATCGGGTGTT TTGTGCCCTG TGAGTCGGCA

Rat TGATTGTGCT CATGGCCCAA ATTGGGTGTT TTGTGCCGTG TGAGTCAGCA

Cow TGGTAGTTCT CATGGCCCAG ATCGGGTGTT TTGTGCCATG TGAGTGGGCA

Dog TGATAGTTCT CATGGCCCAG ATTGGGTGTT TTGTGCCGTG TGAGTCAGCA

Rabbit TGATAGTACT TATGGCCCAA ATTGGGTGTT TTGTGCCGTG TGAATCAGCA

Horse TGATAGTTCT CATGGCCCAA ATTGGGTGCT TTGTGCCATG TGAGTCGGCA

Elephant TGATAGTTCT CATGGCGCAG ATTGGGTGTT TCGTGCCATG CGAGTCGGCA

Chicken TCATTGTTCT TATGGCCCAA ATTGGGTGTT TTGTGCCGTG CAACTCTGCA

Zebra_Finch TGATTGTCCT TATGGCCCAA ATTGGGTGTT TTGTGCCATG TGAGTCTGCA

Zebrafish TGATTGTGCT GATGGCACAG ATAGGCTGTT TTGTGCCATG TGATGAGGCA

Frog TGATTGTCCT TATGGCTCAG ATTGGTTCCT TTGTACCCTG TGATTCTGCA

Fugu TGATCGCCCT GATGGCTCAG ATTGGCTGCT TCGTGCCGTG CGAGAGGGCG

Human GAAGTGTCCA TTGTGGACTG CATCTTAGCC CGAGTAGGGG CTGGTGACAG

Chimpanzee GAAGTGTCCA TTGTGGACTG CATCTTAGCC CGGGTAGGGG CTGGTGACAG

Orangutan GAAGTGTCCA TTGTGGACTG CATCCTGGCC CGGGTAGGGG CTGGTGACAG

Gorilla GAAGTGTCCA TTGTGGACTG CATCTTAGCC CGGGTAGGGG CTGGTGACAG

Marmoset GAAGTGTCCA TTGTGGACTG CATCTTGGCC CGGGTAGGGG CTGGTGACAG

Guinea_Pig GAAGTGTCCA TTGTGGACTG CATCTTGGCT CGGGTAGGGG CTGGTGACAG

Mouse GAAGTGTCCA TTGTGGATTG CATCCTTGCT CGAGTCGGGG CTGGTGACAG

Rat GAGGTGTCCA TTGTGGATTG CATCTTGGCT CGAGTCGGGG CTGGTGACAG

Cow GAAGTGTCCA TTGTGGACTG CATCTTGGCC CGGGTAGGGG CTGGTGACAG

Dog GAAGTGTCCA TTGTGGACTG CATCTTGGCC CGTGTAGGGG CTGGTGACAG

Rabbit GAGGTGTCCA TCGTGGATTG CATCCTGGCT CGGGTTGGGG CCGGTGACAG

Horse GAAGTGACCA TTGTGGACTG CATCTTGGCC CGGGTGGGTG CTGGTGACAG

Elephant GAAGTGTCCA TTGTGGACTG CATCTTGGCT CGGGTAGGGG CTGGTGACAG

Chicken GAAATAACCA TCGTGGATTG TATCCTGGCT CGTGTGGGAG CTGGAGACAG

Zebra_Finch GAAATAACCA TTGTGGATTG TATCCTGGCT CGGGTGGGAG CAGGAGACAG

Zebrafish GAGCTGAGCG TGGTGGACTG TGTTCTTGCT CGAGTAGGTG CGGGTGACAG

Frog GAAATCAGTA TAGTGGACTG CATACTTGCA CGAGTTGGGG CTGGAGACAG

Fugu GAGCTGAGTG TGATTGACAG CATCCTGGCC CGGGTGGGGG CCGGGGACAG

Human TCAATTGAAA GGAGTCTCCA CGTTCATGGC TGAAATGTTG GAAACTGCTT

Chimpanzee TCAATTGAAA GGAGTCTCCA CGTTCATGGC TGAAATGTTG GAAACTGCTT

Orangutan TCAATTGAAA GGAGTCTCCA CGTTCATGGC TGAAATGTTG GAAACTGCTT

Gorilla TCAATTGAAA GGAGTCTCCA CGTTCATGGC TGAAATGTTG GAAACTGCTT

Marmoset TCAATTGAAA GGAGTCTCCA CATTCATGGC TGAAATGTTG GAAACTGCTT

Guinea_Pig TCAGCTGAAA GGAGTGTCCA CATTCATGGC TGAAATGCTG GAAACTGCAT

Mouse TCAACTGAAA GGCGTCTCCA CATTCATGGC TGAAATGCTG GAGACTGCTT

Rat TCAACTGAAA GGCGTCTCCA CATTCATGGC TGAAATGCTG GAAACTGCTT

Cow TCAGTTGAAA GGAGTCTCCA CATTTATGGC TGAAATGTTA GAAACTGCTT

Dog TCAGTTGAAA GGAGTCTCCA CATTTATGGC TGAAATGCTA GAGACTGCTT

Rabbit TCAGTTGAAA GGAGTCTCGA CGTTCATGGC TGAAATGTTG GAAACCGCTT

Horse TCAGTTGAAA GGAGTCTCCA CATTCATGGC TGAAATGTTA GAAACTGCTT

Elephant TCAATTGAAA GGAGTCTCCA CGTTCATGGC TGAAATGTTA GAAACTGCTT

Chicken TCAGCTGAAA GGTGTGTCTA CTTTCATGGC TGAAATGTTG GAAACTGCTT

Zebra_Finch CCAGCTGAAA GGAGTGTCTA CTTTCATGGC TGAAATGCTG GAAACTGCTT

Zebrafish CCAGATTAAA GGAGTGTCCA CTTTTATGGC TGAGATGCTG GAGACAGCAG

Frog TCAACTGAAA GGAGTGTCAA CATTTATGGC CGAGATGCTA GAAACTGCCT

Fugu CCAGGTCAAA GGGGTGTCCA CCTTCATGGC GGAGATGCTG GAGACGGCCG

Human CTATCCTCAG GTCTGCAACC AAAGATTCAT TAATAATCAT AGATGAATTG

Chimpanzee CTATCCTCAG GTCTGCAACC AAAGATTCAT TAATAATCAT AGATGAATTG

Orangutan CTATCCTCAG GGCATCTCCT ---------- ---------- ----------

Gorilla CTATCCTCAG GTCTGCAACC AAAGATTCAT TAATAATCAT AGATGAATTG

Marmoset CTATCCTCAG GTCTGCAACC AAAGATTCAT TAATAATCAT AGATGAATTG

Guinea_Pig CCATCCTCAG GTCTGCAACC AAAGATTCTT TAATAATCAT AGATGAATTG

Mouse CCATCCTCAG GTCAGCAACC AAAGACTCCT TAATAATCAT TGATGAGCTG

Rat CTATCCTCAG GTCGGCAACC AAAGATTCCT TAATAATCAT TGATGAGCTG

Cow CTATTCTCAG GTCTGCAACC AAAGATTCCT TAATAATCAT AGATGAGTTG

Dog CTATTCTCAG GTCTGCAACC AAAGATTCTT TAATAATCAT AGATGAATTG

Rabbit CAATCCTCAG ATCTGCAACT AAAGATTCTT TAATAATCAT AGATGAATTG

Horse CTATTCTCAG GTCTGCGACC AAAGATTCTT TAATAATCAT AGATGAACTG

Elephant CTATTCTCAG GTCTGCCACC AAAGACTCGT TAATAATCAT AGATGAGTTG

Chicken CAATCCTTAG GACAGCATCT GAAAACTCTC TGATAATCAT TGATGAGCTG

Zebra_Finch CAATCCTTAG GACAGCAACT GAAAACTCCC TGATAATCAT TGATGAGCTG

Zebrafish CTATTCTTCG GTCAGCCAGT GAGGATTCTC TCATTATTAT TGATGAATTG

Frog CAATTCTCAG ATCTGCTACA GAGAATTCGC TGATTATCAT TGATGAATTG

Fugu CCATTCTGCG CTCTGCGACC GCCAACTCGC TCATCATCAT CGACGAGCTG

Human GGAAGAGGAA CTTCTACCTA CGATGGATTT GGGTTAGCAT GGGCTATATC

Chimpanzee GGAAGAGGAA CTTCTACCTA CGATGGATTT GGGTTAGCAT GGGCTATATC

Orangutan ---------- ---------- ---------- ---------- ----------

Gorilla GGAAGAGGAA CTTCTACCTA CGATGGATTT GGGTTAGCAT GGGCTATATC

Marmoset GGAAGAGGCA CTTCTACCTA CGATGGATTT GGGTTAGCAT GGGCTATATC

Guinea_Pig GGAAGAGGAA CTTCTACCTA TGATGGCTTT GGGTTAGCGT GGGCTATATC

Mouse GGAAGAGGAA CCTCTACCTA TGATGGATTT GGGTTAGCAT GGGCTATATC

Rat GGAAGAGGAA CCTCTACATA CGACGGCTTT GGGTTAGCAT GGGCCATATC

Cow GGAAGAGGAA CCTCTACCTA TGATGGGTTT GGATTAGCGT GGGCTATATC

Dog GGAAGAGGAA CCTCTACCTA TGATGGATTT GGATTAGCGT GGGCTATATC

Rabbit GGAAGAGGGA CCTCTACCTA CGATGGCTTT GGGCTAGCAT GGGCTATATC

Horse GGAAGAGGAA CCTCCACCTA CGATGGATTT GGGTTGGCAT GGGCTATATC

Elephant GGAAGAGGAA CCTCAACCTA CGATGGATTT GGGTTGGCGT GGGCCATATC

Chicken GGAAGAGGAA CTTCGACCTA TGATGGCTTT GGCTTGGCAT GGGCTATTTC

Zebra_Finch GGAAGAGGAA CTTCTACCTA TGATGGCTTT GGCTTGGCCT GGGCCATCTC

Zebrafish GGCAGAGGCA CATCCACATA TGATGGCTTT GGTCTGGCCT GGGCGATCTC

Frog GGTAGAGGCA CCTCCACGTA TGATGGGTTT GGGCTGGCCT GGGCAATCTC

Fugu GGCCGCGGCA CGTCCACCTA CGACGGCTTC GGCCTGGCCT GGGCCATCAG

Human AGAATACATT GCAACAAAGA TTGGTGCTTT TTGCATGTTT GCAACCCATT

Chimpanzee AGAATACATT GCAACAAAGA TTGGTGCTTT TTGCATGTTT GCAACCCATT

Orangutan ---------- ---------- ---------- ---------- ----------

Gorilla AGAATACATT GCAACAAAGA TTGGTGCTTT TTGCATGTTT GCAACCCATT

Marmoset AGAATATATC GCAACAAAGA TTGGTGCTTT TTGCATGTTT GCAACCCATT

Guinea_Pig AGATTACATT GCAACAAGGA TTGGTGCTTT TTGCATGTTT GCAACTCATT

Mouse AGATTACATT GCAACGAAGA TTGGTGCCTT TTGCATGTTT GCCACCCATT

Rat GGAGTACATC GCAACGAAGA TTGGTGCCTT TTGCATGTTT GCCACCCATT

Cow AGAGTACATT GCAACAAAGA TCGGGGCTTT TTGCATGTTT GCAACCCATT

Dog AGAGTACATT GCAACAAAGA TTGGTGCTTT TTGCATGTTT GCAACACATT

Rabbit AGATTATATT GCAACCAAGA TCGGGGCTTT TTGCATGTTT GCAACCCATT

Horse AGAGTACATT GCAACCAAGA TCGGTGCTTT TTGCATGTTT GCAACCCATT

Elephant AGAGTACATT GCCACAAGGA TTGGTGCCTT TTGCATGTTT GCAACCCATT

Chicken AGAATACATT GCTAGCAAAA TTTGTGCTTT CTGTATGTTT GCTACACATT

Zebra_Finch AGAGTACATT GCCAGCAAAA TCTGTGGGTT TGCCATGTTT GCTACACACT

Zebrafish AGAGTATATC GCCACCCGCC TCAAGTCCTT CTGCCTGTTC GCAACTCACT

Frog AGAATATATT TCCACTAAGA TCAAGGCTTT TTGCATGTTT GCTACCCATT

Fugu CCAGCACATC GCCGCCGCCA TCCGCTGCTT CTGCCTGTTC GCCTCGCACT

Human TTCATGAACT TACTGCCTTG GCCAATCAGA TACCAACTGT TAATAATCTA

Chimpanzee TTCATGAACT TACTGCCTTG GCCAATCAGA TACCAACTGT TAATAATCTG

Orangutan ---------- ---------- ---------- ---------- ----------

Gorilla TTCATGAACT TACTGCCTTG GCCAATCAGA TACCAACTGT TAATAATCTA

Marmoset TTCATGAACT TACTGCCTTG GCCAATCAGA TACCAACTGT TAATAATCTA

Guinea_Pig TTCATGAACT TACTGCATTA GCCAATCAGA TACCAACTGT TAATAATCTG

Mouse TTCATGAACT TACTGCTTTG GCCAACCAAA TACCAACTGT TAATAATCTA

Rat TTCATGAACT TACCGCTTTG GCCAGTCAGA TACCAACCGT TAACAATCTC

Cow TTCATGAACT TACAGCCTTG GCCAATCAGA TACCAACTGT TAATAATCTA

Dog TTCATGAACT TACTGCCTTG GCCAATCAGA TACCAACTGT TAATAATCTA

Rabbit TTCATGAACT CACTGCCTTG GCCCATCATA TACCAACAGT TAATAATCTA

Horse TTCATGAACT TACTGCCTTG GCCAATCAAA TACCAACTGT TAATAATCTA

Elephant TTCATGAACT TACTGCCTTG GCCGATCAGA TACCAACTGT CAATAATCTG

Chicken TTCATGAACT GACAGCTCTT GCTGATCAAG TTCCAACAGT AAATAACCTA

Zebra_Finch TCCATGAGCT CACAGCTCTT GCTGACCAAG TGCCGACAGT AAACAACCTC

Zebrafish TCCATGAGCT GACGGCGCTG GCACAGCAGG TGCCCACTGT CCGCAACCTC

Frog TTCATGAACT AACAGCCCTG GCTGACCAAG TGCCAACAGT GAATAATTTG

Fugu TCCACGAGCT GACGGCGCTG GCGGCGCAGC AGCCCACCGT GCACAACCTC

Human CATGTCACAG CACTCACCAC TGAAGAGACC TTAACTATGC TTTATCAGGT

Chimpanzee CATGTCACAG CACTCACCAC TGAAGAGACC TTAACTATGC TTTATCAGGT

Orangutan ---------- ---------- ---------- ---------- ----------

Gorilla CATGTTACAG CACTCACCAC TGAAGAGACC TTAACTATGC TTTATCAGGT

Marmoset CATGTCACAG CACTCACCAC TGAAGAGACC TTAACTATGC TTTATCAGGT

Guinea_Pig CATGTCACAG CCCTGACCAC TGCTGAGACC TTAACTATGC TTTATCAGGT

Mouse CATGTCACAG CGCTCACTAC TGAGGAGACC CTAACTATGC TTTACCAAGT

Rat CACGTCACAG CGCTGACTAC CGAAGAGACC CTGACTATGC TTTACCAAGT

Cow CATGTCACAG CACTGACTAC TGAGGAGACT TTAACTATGC TTTATCAGGT

Dog CATGTCACAG CACTAACTAC TGAAGAGACC TTAACTATGC TTTATCAGGT

Rabbit CATGTCACAG CCCTGACCAC TGAAGAAACC TTAACCATGC TTTATCAAGT

Horse CATGTCACAG CATTAACTAC TGAAGAGACC TTAACCATGC TGTATCAGGT

Elephant CATGTCACAG CACTGACCAC TGAAGAGACC CTAACTATGC TTTATCAGGT

Chicken CATGTTACTG CTCTCACCAG TGACGACACG CTGACAATGC TGTATCGTGT

Zebra_Finch CATGTCACTG CTCTGACCAG CGATGACACA TTGACCATGC TTTATCGTGT

Zebrafish CATGTCACCG CGCTGACCAC AGACAGCACT CTTACCATGC TCTACAAGGT

Frog CACGTTTACA TATTAATGGC GTCTAAAACA GTATGTAGTT TTGTTGATGC

Fugu CACGTGACGG CGCTGACGTC ACAGAACACG CTGACGATGC TCTACAGAGT

Human GAAGAAAGGT GTCTGTGATC AAAGTTTTGG GATTCATGTT GCAGAGCTTG

Chimpanzee GAAGAAAGGT GTCTGTGATC AAAGTTTTGG GATTCATGTT GCAGAGCTTG

Orangutan -------GGT GTCTGTGATC AAAGTTTTGG GATTCATGTT GCAGAGCTTG

Gorilla GAAGAAAGGT GTCTGTGATC AAAGTTTTGG GATTCATGTT GCAGAGCTTG

Marmoset GAAGAAAGGT GTCTGCGATC AAAGTTTTGG GATTCATGTT GCAGAGCTTG

Guinea_Pig GAAGAAAGGT GTCTGCGATC AGAGTTTCGG GATTCACGTG GCGGAGCTCG

Mouse GAAAAAAGGT GTCTGTGATC AGAGTTTCGG GATTCACGTG GCTGAGCTCG

Rat GAAAACAGGT GTCTGTGATC AGAGTTTCGG GATTCATGTG GCTGAACTCG

Cow GAAGAAAGGT GTCTGTGATC AAAGTTTTGG GATTCATGTT GCTGAGCTTG

Dog GAAGAAAGGT GTCTGTGATC AAAGTTTCGG GATTCATGTT GCAGAGCTTG

Rabbit GAAGAAAGGT GTCTGTGATC AAAGCTTTGG GATTCACGTT GCAGAGCTTG

Horse GAAGAAAGGT ATCTGTGATC AAAGTTTTGG GATTCACGTT GCAGAGCTTG

Elephant GAAGAAAGGT GTCTGTGATC AAAGTTTTGG GATTCACGTG GCGGAACTTG

Chicken CAAGGCAGGT GTTTGTGACC AGAGCTTTGG AATACATGTA GCAGAGCTGG

Zebra_Finch CAAGGAGGGT GTTTGTGACC AGAGTTTTGG AATACATGTA GCAGAGTTGG

Zebrafish CAAAAAGGGT GTGTGTGACC AGAGCTTTGG GATTCATGTG GCGGAGCTTG

Frog ACACTTAGGG GTTTGTGATC AGAGCTTTGG AATACACGTG GCAGAGCTTG

Fugu CAGACCAGGC GTCTGTGACC AGAGCTTCGG GATCCACGTG GCCGAGCTGG

Human CTAATTTCCC TAAGCATGTA ATAGAGTGTG CTAAACAGAA AGCCCTGGAA

Chimpanzee CTAATTTCCC TAAGCATGTA ATAGAGTGTG CTAAACAGAA AGCCCTGGAA

Orangutan CTAATTTCCC TAAGCATGTA ATAGAGTGTG CTAAACAGAA AGCCCTGGAA

Gorilla CTAATTTCCC TAAGCATGTA ATAGAGTGTG CTAAACAGAA AGCCCTGGAA

Marmoset CTAATTTCCC TAAGCATGTA ATAGAGTGTG CTAAACAGAA AGCCCTGGAA

Guinea_Pig CTAATTTCCC TCGACATGTG GTAGAGTGTG CCAAACAGAA AGCCCTGGAA

Mouse CTAACTTCCC GAGGCACGTG ATAGCGTGCG CCAAGCAGAA GGCTCTAGAG

Rat CTAATTTCCC TAGGCATGTG ATAGAGTGCG CCAAGCAGAA GGCTCTGGAG

Cow CTAATTTTCC AAGGCATGTA ATAGAGTGTG CTAAACAGAA AGCCCTGGAG

Dog CAAATTTCCC TAGACATGTT ATAGAGTGTG CTAAACAAAA AGCCCTGGAA

Rabbit CTAATTTCCC TAGGCATGTA ATAGAATGTG CTAAGCAAAA AGCCCAGGAA

Horse CTAATTTCCC TAGGCATGTA ATAGAGTGTG CTAAACAAAA AGCCCTGGAA

Elephant CTAGTTTCCC CAGGCACGTA ATAGAGTGTG CTAAACAAAA AGCCCTGGAG

Chicken CAGCTTTCCC AAAACATGTG ATAGAAAGCG CGAGAGAGAA AGCACTGGAG

Zebra_Finch CAGCTTTCCC AAAGCATGTG ATAGAAAGTG CAAGAGAGAA AGCATTGGAG

Zebrafish CTAGCTTCCC CAAACATGTA ATTGCAAATG CGCGGGAGAA GGCTTTGGAG

Frog CCAACTTTCC TAAACATGTT ATTGAAACTG CAAAGGAAAA GGCACTGCAA

Fugu CTTCCTTCCC CCCCGCCGTG GTTGCCATGG CAAAAGATAA GGCGGAGGAG

Human CTTGAGGAGT TTCAGTATAT TGGAGAATCG CAAGGATATG ATATCATGGA

Chimpanzee CTTGAGGAGT TTCAGTATAT TGGAGAATCG CAAGGATATG ATATCATGGA

Orangutan CTTGAGGAGT TTCAGTACAT TGGAGAATCA CAAGGATATG ATATCATGGA

Gorilla CTTGAGGAGT TTCAGTATAT TGGAGAATCG CAAGGATATG ATATCATGGA

Marmoset CTGGAGGAGT TTCAGTATAT TGGAGAATCA CAAGGATATG ATGACATGGA

Guinea_Pig CTTGAAGAGT TCCAGAGCAT CGGCGCCTCA CAGGGATGCA ACGACAGGGA

Mouse CTTGAAGAAT TTCAGAACAT TGGAACCTCG CTGGGATGTG ACGAAGCCGA

Rat CTTGAGGAGT TTCAGAGCAT TGGGACCTCA CAGGGACACG ATGAAACCCA

Cow TTGGAGGAGT TTCAGAATAT TGGGAAACCA CAGGAATGTG ATGAAATGGA

Dog CTAGAGGAGT TTCAGAATAT TGGAGGATCA CAAGGATATG ATGAAATGGA

Rabbit CTTGAGGAGT TTCAGAATAT TGGAGAATCA CAAGCATATG ATGGAATGGA

Horse CTAGAAGAGT TTCAGAATAT TGGAGAGTCA CAAGAATATG ATGAAATGGA

Elephant CTGGAGGAGT TTCAGAATAT TGGAAAATCT CAAGACTATG GTGAAACAGA

Chicken CTGGAGGAGT TTCAAGACAT TGGCAGGCCC AAGGAATCTG AAGGA---GA

Zebra_Finch CTGGAGGAGT TTCAAAACAT TGGCAAGTCC AAGGAATCTG AAGGA---GA

Zebrafish CTTGAAGAAT TTCAAGACAT CTCCAGTGTT GGG------G AGGAAGCAGG

Frog TTAGAGGAGT TTCAGTTTGT TGGCAACCCC GATGACTGTG ACGAT---GA

Fugu CTGGAGGAAT TTCAGGAAGC TGCAGGAGGG AAGTGGGAGC AGGAGGAAGG

Human ACCAGCAGCA AAGAAGTGCT ATCTGGAAAG AGAGCAAGGT GAAAAAATTA

Chimpanzee ACCAGCAGCA AAGAAGTGCT ATCTGGAAAG AGAGCAAGGT GAAAAAATTA

Orangutan ACCAGCAGCA AAGAAGTGCT ATCTGGAAAG A--------- ----------

Gorilla ACCAGCAGCA AAGAAGTGCT ATCTGGAAAG AGAGCAAGGT GAAAAAATTA

Marmoset ACCAGCAGCA AAGAGGTGCT TTCTGGAAAG AGAGCAAGGT GAAAAAATTA

Guinea_Pig ACCAGCCGCC AAGAAGTGCT ACCTGGAGAG AGAGCAAGGT GAAAGAATCA

Mouse GCCGGCTGCA AAGAGACGCT GCCTGGAAAG AGAGCAAGGT GAGAAAATTA

Rat GCCAGCTGCA AAGAGGCGCT GCCTGGAAAG AGAGCAAGGT GAGAAGATTA

Cow ACCAGCAGCA AAGAGATGCT ATCTGGAAAG AGAGCAAGGT GAAAAAATTA

Dog ACCAGCAGCA AAGAGGTGCT ATCTGGAAAG AGAGCAAGGC GAAAAAATTA

Rabbit GCCAGCAGCC AAGAAGTGCT ACCTGGAAAG AGAGCAAGGT GAAAAAATTA

Horse ACCAGCAGCA AAGAGGTGCT ATCTGGAAAG AGAGCAAGGT GAAAAAATTA

Elephant ACCAGCAGCA AAACGATGCT ATCTGGAAAG AGAGCAAGGT GAAAAAATTA

Chicken GCCAGCAGCC AAGAGATGCT ACAGAGAAAG AGAGGAAGGC GAAAAGATCA

Zebra_Finch ACCACCAGTC AAGAAATTAT ACAGAGAAAG AGAGGAAGGT GAAAAGATAA

Zebrafish ACCCAAAGCC AAGAAACGCT GCATGGAGAA ACAGGAGGGA GAGAAGATCA

Frog ACCAACTAGG AAGAGGCGCT GCAAAGAAAA GGAGGAAGGG GAAAAGATAA

Fugu AGGTGAAGCC AAGAGACGGC GCCTGGACAA ACAGGTGGGA GAGAAACTGA

Human TTCAGGAGTT CCTGTCCAAG GTGAAACAAA TGCCCTTTAC TGAAATGTCA

Chimpanzee TTCAGGAGTT CCTGTCCAAG GTGAAACAAA TGCCCTTTAC TGAAATGTCA

Orangutan ---------- ---------- ---------- ---------- ----------

Gorilla TTCAGGAGTT CCTGTCCAAG GTGAAACAAA TGCCCTTTAC TGAAATGTCA

Marmoset TTCAGGAGTT CCTGTCCAAG GTGAAACAAA TGCCCTTTAC TGAAATGTCA

Guinea_Pig TCCAGGAGTT TCTGACCAAG GTGAAGCAAG TTCCCTTCAC TGAAATGTCA

Mouse TTCTGGAGTT CCTGTCGAAG GTCAAGCAGG TGCCCTTTAC TGCCATGTCG

Rat TTCTGGAGTT CCTGTCCAAG GTCAAGCAAG TGCCCTTCAC TGACTTGTCG

Cow TTCAAGAGTT TCTATCCAAG GTAAAGCAAG TGCCCTTTAC TGAAATGTCA

Dog TTCAGGAGTT TTTATCCAAG GTGAAGCAAG TGCCTTTTAC TGAAATGTCA

Rabbit TTCAGGAGTT TCTATACAAG GTGAAGCAAG TGCCCTTTAC TGAAATGTCA

Horse TTCAAGAGTT TCTATCCAAG GTGAAGCAAG TGCCCTTTAC TGAAATGTCA

Elephant TTCAGGAGTT TCTGTCCAAG GTGAAGCAAG TGCCCTTTAC CACAATGTCA

Chicken TTCAGGATTT TCTCTCTCAG GTGAAAGCAC TGCCCCTGAC GGATATGTCG

Zebra_Finch TTCAGGATTT TCTTTGTCAA GTGAAAGCAT TGCCCCTGAC AGATATGTCG

Zebrafish TTGAGGCTTT TCTGGCTAAG GTTAAATCAA TGCCTGTTGA TGGGATGTCT

Frog TCCAGGATTT CCTTTCCAGA GTAAAAGCTT TGCCTCTGAC AGAAATGTCT

Fugu TCCAGGACTT CCTGGACAAG GCCAGATCAC TTCCTGTTTC CAGCATGAGT

Human GAAGAAAACA TCACAATAAA GTTAAAACAG CTAAAAGCTG AAGTAATAGC

Chimpanzee GAAGAAAACA TCACAATAAA GTTAAAACAG CTAAAAGCTG AAGTAATAGC

Orangutan ---------- ---------- ---------- ---------- ----------

Gorilla GAAGAAAACA TCACAATAAA GTTAAAACAG CTAAAAGCTG AAGTAATAGC

Marmoset GAAGAAAACA TCACAATAAA GTTAAAACAG CTAAAAGCTG AGGTAATTGC

Guinea_Pig GAAGAAAGCA TCACAAAAAA ATTAAAGCAG CTGAAAGCTG AAGTGATGGC

Mouse GAGGAGAGCA TCTCCGCGAA GCTGAAGCAA CTGAAAGCCG AGGTGGTCGC

Rat GAGGAGAGCG TCTCAGTGAA GCTGAAGCAA CTGAAAGCTG AGGTGCTGGC

Cow GAAGAAAGCA TCACAAGAAA GTTAAAACAG CTAAAAGCTG AGGTGATCGC

Dog GAAGAAAACA TCACAATGAA GTTAAAACAG CTGAAAGCTG AGGTAATAGC

Rabbit GAAGAAAACA TCACAATAAA GTTAAGACAA CTAAAAGCTG AGGTGATAGC

Horse GAAGAAAACA TCACGATAAA GTTAAAACAG CTGAAAGCTG AGGTGATAGC

Elephant GAAGGAGACA TCACAATGAA GTTGAAACAG CTGAAAGCTG AGGTGATAGC

Chicken GAAGAAGACA TCAAAACCAA GCTGAAGCAG CTGAGAGCTG ATGTGTTGGC

Zebra_Finch GAAGAAGACA TTAAAGTCAA GTTGAAACAG CTGAGAAATG ATGTGCTGGC

Zebrafish GACAAGGCTG TCAAAGAGGA GCTGCGCAAA CTCAAGGCAG AGGTCATCAG

Frog GAGGAAGAAA TAAAGAGCAA GCTGCAGCAG TTCAGATCTG ACGTCCTGGC

Fugu GAAGATGAGG TGAAGGCGGA GCTTCGCAGA ATGAAGCAAG AGCTTGTTGC

Human AAAGAATAAT AGCTTTGTAA ATGAAATCAT TTCACGAATA AAAGTTACTA

Chimpanzee AAAGAATAAT AGCTTTGTAA ATGAAATCAT TTCGCGAATA AAAGTTACTA

Orangutan ---------- ---------- ---------- ---------- ----------

Gorilla AAAGAATAAT AGCTTTGTAA ATGAAATCAT TTCACGAATA AAAGTTACTA

Marmoset AAAGAATAAT AGCTTTGTAA ATGAAATCAT TTCACGAATA AAAGTTACTA

Guinea_Pig ACAGAATAAC AGTTTTGTGA ATGAAATCAT TTCACGAATA AAGGTTACTA

Mouse AAAGAACAAC AGCTTCGTAA ACGAGATCAT TTCACGGATA AAGGCTCCGG

Rat GAAGAACAAC AGCTTTGTAA ATGAAATCAT CTCACGGGTA AAGGCTCCG-

Cow AAAGAATAAT AGTTTTGTAA ATGAAATCAT TTCCCGAATA AAGGTAACTG

Dog AAAGAATAAT AGTTTTGTAA ATGAAATCAT TTCACGAATC AAAGTTTCTA

Rabbit AAAGAATAAT AGTTTTGTAA ATGAAATCAT TTCACGAATA AAGGTACCCT

Horse AAAGAATAAT AGTTTTGTAA ATGAAATCAT CTCACGAATA AAGGTTACTG

Elephant AAAGAATAAT GGTTTTGTAA ATGAAATCAT TTCACGAATA AAGGTTACT-

Chicken AAAGAACAAC GGTTTCGTGA ACGAAATCAT TTCCAGAACA AAAGTTACAC

Zebra_Finch AAAGAACAAC AGTTTTGTGA ATGAAATAAT TTCTCGAACG AAAGTTACAT

Zebrafish CCAAAACAAT AGCTTCGTCA ATGAGATAGT GTCACGCGTC AAACTCAGTT

Frog CAGAAATAAC AGTTTTGTCC ATGAGGTTAT TTCCAGAACA AAACCAGGAT

Fugu CGAGGGCAAC GCGTACATCG GAGACATCTT GGCACGCGTG CCGACGGCC-

Human CG

Chimpanzee CG

Orangutan --

Gorilla CG

Marmoset CG

Guinea_Pig CC

Mouse CT

Rat --

Cow CG

Dog CA

Rabbit GC

Horse CA

Elephant --

Chicken CG

Zebra_Finch CA

Zebrafish CA

Frog TG

Fugu --

;

END;

BEGIN DATA;

DIMENSIONS NTAX=19 NCHAR=5436;

FORMAT DATATYPE=DNA INTERLEAVE=yes GAP=-;

[Gene Name: TSC2]

[Name: Human Len: 5436 Check: 0]

[Name: Gorilla Len: 5436 Check: 0]

[Name: Chimpanzee Len: 5436 Check: 0]

[Name: Orangutan Len: 5436 Check: 0]

[Name: Marmoset Len: 5436 Check: 0]

[Name: Cow Len: 5436 Check: 0]

[Name: Dog Len: 5436 Check: 0]

[Name: Guinea_Pig Len: 5436 Check: 0]

[Name: Horse Len: 5436 Check: 0]

[Name: Mouse Len: 5436 Check: 0]

[Name: Pig Len: 5436 Check: 0]

[Name: Rabbit Len: 5436 Check: 0]

[Name: Rat Len: 5436 Check: 0]

[Name: Elephant Len: 5436 Check: 0]

[Name: Frog Len: 5436 Check: 0]

[Name: Zebrafish Len: 5436 Check: 0]

[Name: Chicken Len: 5436 Check: 0]

[Name: Fugu Len: 5436 Check: 0]

[Name: Zebra_Finch Len: 5436 Check: 0]

MATRIX

Human ATGGCCAAAC CAACAAGCAA AGATTCAGGC TTGAAGGAGA AGTTTAAGAT

Gorilla ATGGCCAAAC CAACAAGCAA AGATTCAGGC TTGAAGGAGA AGTTTAAGAT

Chimpanzee ATGGCCAAAC CAACAAGCAA AGATTCAGGC TTGAAGGAGA AGTTTAAGAT

Orangutan ATGGCCAAAC CAACAAGCAA AGATTCAGGC TTGAAGGAGA AGTTTAAGAT

Marmoset ATGGCCAAGC CCACAAGCAA AGATTCGGGC CTGAAGGAGA AGTTTAAGAA

Cow ATGGCTAAGC CAGCAAGCAA AGATTCAGGC TTGAAGGAGA AGTTCAAGAT

Dog ATGGCCAAGC CTACGAGCAA AGACTCAGGC CTGAAGGAGA AGTTCAGGAT

Guinea_Pig ATGGCCAAGC CAGCAAGCAA AGATTCAGGC TTGAAGGAGA AGTTCAAGAC

Horse ATGGCCAAGC CAGCAAGCAA AGATTCAGGC TTGAAGGAGA AGTTCAAGAC

Mouse ATGGCCAAAC CAACAAGCAA AGATTCCGGC TTGAAGGAGA AGTTCAAGAT

Pig ATGGCCAAGC CGACTGGCAA AGATTCAGGC CTGAAGGAGA AGTTCAAGAT

Rabbit ---------- ---------- ---------- ---------- ----------

Rat ATGGCCAAAC CAACTAGCAA AGATTCAGGT TTGAAGGAGA AGTTCAAGAT

Elephant ATGGCCAAGC CCACAAGCAA AGATTCAGGC TTGAAGGAGA AGTTCAAGAT

Frog ATGGCCAAGC AGATGGGTAA AGATCCTGGC CTGAAGGAAA AGGTGAAAAT

Zebrafish ---------- ---------- ---------- ---------- ----------

Chicken ATGGCAAAGC CTCAAAGCAA GGATTCGGGG CTGAAGGAGA AGTTCAAGAA

Fugu ATGAACAAGC AGTCAAGCAA AGAG---AGT CTCCGGGACA AGGTGAAGGG

Zebra_Finch ATGGCAAAGC CTCAGAGCAA GGATTCCGGG CTGAAGGAGA AGTTCAGGAA

Human TCTGTTGGGA CTGGGAACAC CGAGGCCAAA TCCCAGGTCT GCAGAGGGTA

Gorilla TCTTTTGGGA CTGGGAACAC CGAGGCCAAA TCCCAGGTCT GCAGAGGGTA

Chimpanzee TCTGTTGGGA CTGGGAACAC CGAGGCCAAA TCCCAGGTCT GCAGAGGGTA

Orangutan TCTGTTGGGA CTGGGAACAC CGAGGCCAAA TCCCAGGTCT GCAGAGGGTA

Marmoset TCTGTTGGGA CTGGGAACAC CGAGGCCAAA TCCCAGGTCT GCAGAGGGCA

Cow TCTTTTGGGA CTGGGGACTC CACGGCCAAA TCCCAGGTCT GCAGAGGGCA

Dog TCTCTTGGGA CTGGGAACCC CGAGGCCAAA CCCCAGGTCA GCAGAGGGCA

Guinea_Pig TTTTTTGGTC CTGCGAACAA CAAGACCAAA TCCTAGGTCT GCAGAAGGCG

Horse TCTGCTGGGA CTGGGAACTC CGAGGCCAAA TCCCAGGACT GCAGAGGGCA

Mouse ACTGTTGGGA TTGGGAACAT CGAGGCCAAA TCCCAGGTGT GCAGAAGGCA

Pig TCTCCTGGGA CTGGGCACTT CAAGGCCAAA CCCCCGGTCC GCAGAGGGCA

Rabbit ---------- ---------- ---------- ---------- ----------

Rat ACTATTAGGA CTGGGAACGT CAAGGCCAAA TCCTAGGTGT GCAGAAGGCA

Elephant TCTCCTGGGC CTGGGAACAC CAAGGCCACA TCCCAGGTCT GCAGAGGGCA

Frog GCTATTGGGA TTGGTGCCTG CACGAGTTAA AACCAAGCCT TCAGAGTGGA

Zebrafish ---------- ---------- ---------- ---------- ----------

Chicken TCTCTTGGGG CTGGGAACAT CCCGGGGAAG TTCAAAGTCA TCAGAGGGCA

Fugu GATCTTTGGA TTGGGAACGC CACGACCTCC AAATAAACAA AGCGACAGCC

Zebra_Finch CCTCCTGGGG CTGGGAACAT CCCGGGGGAG TTCCAAGTCG TCGGAGGGCA

Human AACAGACGGA GTTTATCATC ACCGCGGAAA TACTGAGAGA ACTGAGCATG

Gorilla AACAGACGGA GTTTATCATC ACCGCGGAAA TACTGAGAAG GCAGGCAGCA

Chimpanzee AACAGACGGA GTTTATCATC ACCGCGGAAA TACTGAGAGA ACTGAGCATG

Orangutan AACAGACGGA GTTTATCATC ACCGCGGAAA TACTGAGAGA ACTGAGCATG

Marmoset AACAGACAGA GTTTATCATC ACCGCGGAAG TCCTGCGGGA GCTGCCTTTG

Cow AACAGACTGA GTTTATCATC ACCGCGGAAA TCCTGAGAGA GCTGAGTGTT

Dog AGCAGACCGA GTTCATCATC ACGGCGGACA TCCTGAGAGA GCTGAGTGTT

Guinea_Pig TTCAGACCGA GTTTATCATC ACAGCGGAAA TCCTGAGAGA ACTGAGTGTT

Horse GACAGACAGA GTTCATCATC ACGGCGGAAA TCCTGAGAGA ACTGAGTGTT

Mouse AACAGACTGA GTTTATCATC ACATCGGAAA TCTTGAGAGA ACTGAGTGGT

Pig AACAGACGGA GTTTATCATC ACGGCGGAAA TCCTGAGAGA ACTGAGTGTT

Rabbit ---------- ---------- ---------- ---------- ----------

Rat AACAGACGGA GTTTATCATC ACAGCGGAAA TCTTGAGAGA ACTGAGTGGT

Elephant GACAGACGGA GTTTATCATC ACAGCAGAAA TCCTGAGAGA ACTGAGTGTC

Frog AACATCCGGA ATATGTCATT ACTGCAGATT TAATGAAGGA GCTGAGCTCT

Zebrafish ---------- ---------- ---------- ---------- ----------

Chicken AGCAAACGGA ATTCATCATC ACTGCAGAAA TACTCAAGGA ACTGAGCATA

Fugu GATCGTCAGA GTTTATCATT ACGTCTGACA TTATCAAGGA GCTCCACCCA

Zebra_Finch AGCAGACAGA GTTTATCATC ACCGCAGAAA TACTCAAGGA ACTAAGCATA

Human GAATGTGGCC TCAACAATCG CATCCGGATG ATAGGGCAGA TTTGTGAAGT

Gorilla GAGGTCGGGC AGGGGCATGG GGTCGAGGGC CTGATGCTGG TGTGTTCCCT

Chimpanzee GAATGTGGCC TCAACAATCG CATCCGGATG ATAGGGCAGA TTTGTGAAGT

Orangutan GAATGTGGCC TCAACAATCG CATCCGGATG ATAGGGCAGA TTTGTGAAGT

Marmoset TGTTTGGGCT ACAGCAACAC AGAAAGGGTT TGTTCTGAAA GCTGTGTGTG

Cow GAATGTGGCC TCAATAATCG CATCCGGGTG ATAGGGCAGA TCTGTGAGGT

Dog GAATGCGGCA TCAACAATCG CATCCGGGTG ATAGGGCAGA TCTGCGAAGT

Guinea_Pig GAATGTGGTC TCAACAATCG CATCCGGGTA ATAGGGCAGA TTTGTGAAGT

Horse GAATGTGGCC TCAACAATCG TATCCGGGTG ATAGGGCAGA TCTGTGAAGT

Mouse GAATGCGGCC TCAACAATCG CATCCGAATG ATAGGGCAGA TCTGTGATGT

Pig GAATGTGGCC TCAACAATCG CATCCGGATG ATAGGACAGA TCTGCGAGGT

Rabbit ---------- ---------- ---------- ---------- ----------

Rat GAATGCGGCC TCAACAATCG AATCCGGATG ATAGGGCAGA TCTGTGATGT

Elephant GAGTGTGGTC TGAACAATCG CATCCGGGTC ATAGGGCAGA TCTGTGAAGT

Frog GAGTCTGCTC TCCACGTTCG CATCCGTGCA ATTATTCAGG TCTGTGAGCT

Zebrafish ---------- ---------- ---------- ---------- ----------

Chicken GAATGTGGAT TAAGTAATAG GATACGAGCA ATCAGCCAAA TCTGTGAAGT

Fugu GACTGCGGCC TGAGCAACCG GGTCCGTATG TTGAATCAAG TATGTGACCT

Zebra_Finch GAGTGTGGAT TGAGTAACAG GATACGAGCA ATCAGTCAAA TTTGTGAAGT

Human CGCAAAAACC AAGAAATTTG AAGAGCACGC AGTGGAAGCA CTCTGGAAGG

Gorilla AGAGGAGAGC CACAAGCAGT TCTGGCACGC AGTGGAAGCA CTCTGGAAGG

Chimpanzee CGCAAAAACC AAGAAATTTG AAGAGCACGC AGTGGAAGCA CTCTGGAAGG

Orangutan TGCAAAAACC AAGAAATTTG AAGAGCACGC AGTGGAAGCA CTTTGGAAGG

Marmoset TGGGTACGCA GCAGCCTTTG AACAGCATGC AGTGGAAGCA CTCTGGAAGG

Cow TGCCAGAACG AAGAAATTCG AAGAGCACGC GGTGGAAGCA CTCTGGAAGG

Dog TGCAAAAACG AAGAAATTTG AAGAGCATGC AGTGGAAGCG CTCTGGAAGG

Guinea_Pig TGCAAAAGCT AAGAAATTTG AAGAGCATGC AGTGGAAGCA CTGTGGAAGG

Horse CGCAAAAACG AAGAAATTTG AAGAGCACGC AGTGGAAGCA CTCTGGAAGG

Mouse GGCAAAAACT AAGAAGCTTG AGGAGCATGC AGTGGAGGCA CTTTGGAAGG

Pig TGCCAGAACC AAAAAGTTTG AAGAGCATGC AGTGGAAGCG CTCTGGAAGG

Rabbit ---------- ---------- ---------- ---------- ----------

Rat GGCAAAAACT AAGAAACTTG AAGAGCATGC AGTGGAGGCA CTTTGGAAGG

Elephant AGCAAAAACT AAGAAGTTTG AGGAGCATGC CGTGGAAGCA CTGTGGAAAG

Frog TGCAAAGACG AAAAAATTTG AAGAGCATGC AGTAGAAGCA ATCTGCCAAT

Zebrafish ---------- ---------- ---------- ---------- ----------

Chicken GGCAAAAACC AAAAAAATTG AAGAGCATGC AGTGGAAGCA ATATGGAAGG

Fugu TGCCAAAACG AAAAAGTTTG AAGAGCACGC AGTGGAGGCA GTATGGAAAT

Zebra_Finch GGCAAAAACC AAAAAAATTG AAGAGCACGC CGTGGAGGCC GTGTGGAAGG

Human CGGTCGCGGA TCTGTTGCAG CCGGAGCGGC CGCTGGAGGC CCGGCACGCG

Gorilla CGGTTGCGGA TCTGTTGCAG CCGGAGCGGC CGCTGGAGGC CCGGCACGCG

Chimpanzee CGGTCGCGGA TCTGTTGCAG CCGGAGCGGC CGCTGGAGGC CCGGCACGCG

Orangutan CGGTCGCGGA TCTGTTGCAG CCGGAGCGGC CACTGGAGGC CCGGCACGCG

Marmoset CAGTTGCGGA TATGTTGCAG CCAGAACGGC CGCTGGAAGC CCGGCACGCG

Cow CCGTCTCGGA CCTGCTGCAG CCAGAGCGGC CACCCGAGGC CCGGCATGCG

Dog CTGTCGCGGA CCTGCTGCAG CCGGAGCGTC CCCCCGAGGC CCGGCATGCA

Guinea_Pig CTGTTGAGGA CCTGTTGCAG CCAGAGCGGC CACCAGAGGC CCGACATGCA

Horse CCGTCGCAGA CCTGCTGCAG CCTGAGCGGC CACCTGAAGC CCGGCACGCA

Mouse CTGTCTCAGA CTTGCTACAG CCAGAGCGGC CACCAGAGGC CCGGCATGCA

Pig CCGTCTCAGA CTTGCTGCAG CCAGAGCGGC CGCCAGAGGC CCGGCACGCA

Rabbit ---------- ---------- ---------- ---------- ----------

Rat CTGTCTCAGA CTTGCTACAG CCAGAGCGGC CACCGGAGGC CCGACATGCA

Elephant CTGTCGCGGA CTTGCTGCAG CCAGACCGGC CACCAGAGGC ACGACATGCA

Frog CAGTTGGTGA CTTGATACAG CCCGAACAGC CAGTAGAAGC TCGACATCAC

Zebrafish ---------- ---------- ---------- ---------- ----------

Chicken TGGTAGCTGA TATGCTCCAG CCAGAGCGCC CAGCTGAAGC CAGACATGCT

Fugu CCGTGGAAGA CATGCTGTCT CCAGAGCAGC CACCTGAAGC CAGACACGCT

Zebra_Finch TGGTGGCTGA CATGCTGCAG CCGGAGCGTC CCGCCGAGGC CAGGCACGCC

Human GTGCTGGCTC TGCTGAAGGC CATCGTGCAG GGGCAGGGCG AGCGTTTGGG

Gorilla GTGCTGGCTC TGCTGAAGGC CATCGTGCAG GGGCAGGGCG AGCGTTTGGG

Chimpanzee GTGCTGGCTC TGCTGAAGGC CATCGTGCAG GGGCAGGGCG AGCGTTTGGG

Orangutan GTGCTGGCTC TGCTGAAGGC CATCGTGCAG GGACAGGGCG AGCGTTTGGG

Marmoset GTGCTGGCTC TGCTGAAGGC CATCGTGCAG GGGCAGGGCG AGCGTTTGGG

Cow GTGCTGGCTC TGCTGAAGGC CATCGTGCAG GGGCAGGGGG ACCGCCTGGG

Dog GTGCTGGCGC TGCTGAAGGC CATTGTGCAG GGGCAGGGGG ACCGCCTGGG

Guinea_Pig GTGCTGGCCC TGCTGAAGGC TATTGTGCAG GGGCAGGGTG ACCGGTTGGG

Horse GTGCTGGCTC TGCTGAAGGC CATCGTGCAG GGGCAGGGGG ACCGCTTGGG

Mouse GTTCTCACCT TATTGAAGGC CATTGTACAG GGACAGGGTG ATCGTTTGGG

Pig GTGCTGGCTC TGCTGAAGGC CATCGTGCAG GGACAGGGGG ACCGCTTGGG

Rabbit ---------- ---------- ---------- ---------- ----------

Rat GTTCTGGCCT TGTTGAAGGC CATTGTGCAG GGACAGGGTG ATCGTTTGGG

Elephant GTGCTGGCCC TGCTCAAGGC CATCGTGCAG GGCCAGGGTG ACCGCCTGGG

Frog GTTCTGCAGT TACTAAAGGC CATTATTGTG GGGCAGGGTG AAAGACTTGG

Zebrafish ---------- ---------- ---------- ------GGCG AGTGGTTGGG

Chicken GTTCTTCATC TCCTAAAGTC AATCATTCAA GGACAGGGTG AGAGATTAGG

Fugu GTCCTGCAGC TTCTGAGGGC AGTCATACAG GGGCAGGGCG AGTGGCTCGG

Zebra_Finch GTGCTCCACC TGCTCAAGTC CATCGTCCAG GGACAGGGTG AGAGGCTGGG

Human GGTCCTCAGA GCCCTCTTCT TTAAGGTCAT CAAGGATTAC CCTTCCAACG

Gorilla GGTCCTCAGA GCCCTCTTCT TTAAGGTCAT CAAGGATTAC CCTTCCAACG

Chimpanzee GGTCCTCAGA GCCCTCTTCT TTAAGGTCAT CAAGGATTAC CCTTCCAACG

Orangutan GATCCTCAGA GCCCTCTTCT TTAAGGTCAT CAAGGATTAC CCTTCCAATG

Marmoset GATCCTCAGG GCTCTCTTCT TTAAGGTCAT CAAGGATTAC CCTTCCAACG

Cow CGTTCTCAGG GCCCTCTTCT TCAAGGTCAT CAAGGATTAT CCCTCTAATG

Dog CGTTCTCCGG GCTCTGTTCT TTAGGGCCAT CAAGGATTAC CCCGCTAACG

Guinea_Pig TATTCTCAGA GCCCTCTTCT TTAAGGTCAT CAAAGATTAC CCTTCCAATG

Horse CATTCTCAGG GCCCTCTTCT TTAAAGTCAT CAAGGATTAC CCCTCTAATG

Mouse AGTTCTCAGA GCCCTCTTCT TCAAAGTGAT CAAGGACTAC CCCTCCAATG

Pig CATCCTCAGG GCCCTCTTCT TTAAGGCCAT CAAGGATTAC CCCTCTCACG

Rabbit ---------- ---------- ---------- ---------- ----------

Rat GGTTCTCCGA GCCCTCTTCT TCAAAGTGAT CAAGGACTAT CCTTCCAACG

Elephant TGTGCTCCGT GCCCTCTTCT TCAAGGTCAT AAAGGATTAC CCTTCGAACA

Frog CGTCCTTCGT GCCCATTTCT TCCGAGTCAT AAAAGAGTAT CCTATGAATG

Zebrafish TCCGCTGAGA GCTTACTTCT TTAAGGTCAT TCTGGACTAT CCTTCTAATG

Chicken GATCCTTAGA GCACATTTTT TTAAGGTTAT TAAGGACTAT CCTTCCAATG

Fugu CCCACTTCGG GCTTACTTTT TCAAGGTGAT CAGGGACTAC CCATGCAACG

Zebra_Finch GATCCTCAGA GCCCATTTTT TTAAGGTTAT TAAGGATTAT CCCTCCAATG

Human AAGACCTTCA CGAAAGGCTG GAGGTTTTCA AGGCCCTCAC AGACAATGGG

Gorilla AAGACCTTCA CGAAAGGCTG GAGGTTTTCA AGGCCCTCAC AGACAATGGG

Chimpanzee AAGACCTTCA CGAAAGGCTG GAGGTTTTCA AGGCCCTCAC AGACAATGGG

Orangutan AAGACCTTCA CGAAAGGCTG GAAGTTTTCA AGGCCCTCAC AGACAATGGG

Marmoset AAGACCTCCA CGAAAGGCTG GAAGTTTTCA AGGCCCTCAC GGACAATGGG

Cow AAGACCTCCA CGAGAGACTG GAAGTTTTCA AGGCCCTCAC AGACAATGGG

Dog AAGACCTCCA TGAAAGACTG GAAGTTTTCA AGGCCCTCAC GGACAATGGG

Guinea_Pig AAGACCTCCA TGAAAGGTTG GAAGTTTTCA AGGCTCTCAC AGACAATGGG

Horse AAGACCTCCA CGAAAGACTG GAAGTTTTCA AGGCCCTCAC GGACAATGGG

Mouse AAGACCTCCA TGAAAGGCTA GAAGTTTTTA AGGCCCTCAC AGACAATGGG

Pig AAGACCTCCA CGAGAGGCTG GAGGTTTTCA AGGCGCTCAC GGACAACGGG

Rabbit ---------- ---------- ---------- ---------- ----------

Rat AAGACCTCCA TGAAAGGCTA GAGGTTTTTA AGGCCCTCAC AGACAATGGA

Elephant AAGACTTGCA CGAAAGGCTG GAGGTGTTCA AGGCCCTGAC GGACAATGGG

Frog AGGACCTTGC AGAGCGACTG GAGGTTTTTA AAGCTCTCAC CGACAATGGG

Zebrafish AAGAGCTGCC GGAGAGACTG GAGGTGTTCA AGGCCCTCAC AGAGAACGGA

Chicken AAGACTTGCA TGAACGGCTT GAAGTTTTTA AGGCTCTAAC AGAGAATGGA

Fugu AAGACCTCTC TGATAGGCTG GAAGTTTTCA AGGCTTTAAC TGAAAACGGC

Zebra_Finch AAGATCTGCA CGAGCGGCTG GAAGTGTTCA AGGCTCTGAC GGAGAACGGA

Human AGACACATCA CCTACTTGGA GGAAGAGCTG GCTGACTTTG TCCTGCAGTG

Gorilla AGACACATCA CGTACTTGGA GGAAGAGCTG GCTGACTTTG TCCTGCAGTG

Chimpanzee AGACACATCA CCTACTTGGA GGAAGAGCTG GCTGACTTTG TCCTGCAGTG

Orangutan AGACACATCA CCTACTTGGA GGAAGAGCTG GCTGACTTTG TCCTGCAGTG

Marmoset AGACATATCA CCTACTTGGA GGAAGAGCTG GCTGACTTTG TCCTGCAGTG

Cow AGACACATCA CCTACTTGGA AGAAGAGCTG GCGGACTTTG TCCTGCAGTG

Dog AGACACATCA CCTACTTGGA AGAAGAACTG GCTGGGTTTG TCCTGCAGTG

Guinea_Pig AGACATATCA CCTACTTGGA AGAAGAACTG GCGGACTTTG TCTTACAATG

Horse AAACACATCA CCTACTTGGA AGAAGAACTG GCTGAGTTTG TCCTGCAGTG

Mouse AGGCACATTA CCTATTTGGA AGAAGAACTG GCAGAGTTTG TCCTGCAGTG

Pig AGGCACATCA ACTACTTGGA AGAAGACCTG GCCGAGTTTG TCCTGCAGTG

Rabbit ---------- ---------- ---------- ---------- ----------

Rat AGGCATATTA CCTACTTGGA AGAAGAACTG GCAGAGTTTG TCCTGCAGTG

Elephant AGACACATAA CCTACTTGGA AGAAGAGCTG GCTGAGTTTG TCCTGCAGTG

Frog CGAGAAATTG CTTACTTAGA AGAAGAGCTC GCTGTCTTCG TCCTCCAGTG

Zebrafish AAGGACATCA CATATCTAGA GGAGGAGATT GCTCGGTTTG TCCTGTTGTG

Chicken CGATACATCA CTTACTTAGA AGAAGAGTTA GCTGACTTCG TGCTACAGTG

Fugu AAAGACATCA CATACCTGGA GGAGGACATA GCACGATTTG TCCTCCTCTG

Zebra_Finch CGCTACATCA CCTACTTAGA GGAGGAGCTG GCTGATTTTG TGCTACAGTG

Human GATGGATGTT GGCTTGTCCT CGGAATTCCT TCTGGTGCTG GTGAACTTGG

Gorilla GATGGATGTT GGCTTGTCCT CGGAATTCCT TCTGGTGCTG GTGAACTTGG

Chimpanzee GATGGATGTT GGCTTGTCCT CGGAATTCCT TCTGGTGCTG GTGAACTTGG

Orangutan GATGGATGTT GGCTTGTCCT CGGAATTCCT TCTGGTGCTG GTGAACTTGG

Marmoset GATGGATGTT GGCTTGTCCT CGGAATTCCT TCTGGTGCTG GTGAATTTGG

Cow GATGGACGTT GGCCTGTCCT CCGAATTCCT CCTGGTGCTC GTGAACTTAG

Dog GATGGACATC GGCCTGTCCT CCGAATTCCT CCTGGTGCTT GTGAACTTGG

Guinea_Pig GATGGATGTT GGCTTGTCTT CAGAATTCCT GCTGGTGCTT GTGAACTTGA

Horse GATGGACATC GGCCTGTCCT CAGAATTCCT GCTGGTGCTT GTGAACTTGG

Mouse GATGGATGTT GGCTTGTCCT CAGAATTCCT TCTGGTACTT GTCAACCTGG

Pig GATGGACGTC GGCCTGACCT CCGAGTTCCT TCTGGTGCTC GTGAACTTGG

Rabbit ---------- ---------- ---------- ---------- ----------

Rat GATGGATGTT GGCTTGTCCT CAGAATTCCT TCTGGTACTC GTGAACCTGG

Elephant GATGGATGTG GGCTTATCCT CAGAATTCCT GCTGGTGCTT GTGAACCTGG

Frog GATGGAAATT GGTTTGACTC CGGATTTCCT GCATGTTTTA GTTAACCTGG

Zebrafish GATGGAGAAC AGCATGTCCT CAGATTTCCT GCACGTCCTC GTCAACCTTG

Chicken GATGGATGTT GGTTTGTCTT CAGAGTTCCT TTTGGTGTTA GTGAACCTGG

Fugu GATGGACATA GGACTTACAT CTGACTTCCT CCATGTACTT GTAAATCTGG

Zebra_Finch GATGGATGTT GGGCTGACTT CTGAGTTCCT CCTGGTTCTG GTGAACCTGG

Human TCAAATTCAA TAGCTGTTAC CTCGACGAGT ACATCGCAAG GATGGTTCAG

Gorilla TCAAATTCAA TAGCTGTTAC CTCGACGAGT ACATCGCAAG GATGGTTCAG

Chimpanzee TCAAATTCAA TAGCTGTTAC CTGGACGAGT ACATCGCAAG GATGGTTCAG

Orangutan TCAAATTCAA TAGCTGTTAC CTCGACGAGT ACATCGCAAG GATGGTTCAG

Marmoset TGAAATTCAA CAGCTGCTAC CTCGACGAAT ACATCGCAAG GATGGTTCAG

Cow TCAAATTCAA CAGCTGTTAC CTTGATGAGT ACATCGCATC CATGGTCCAC

Dog TCAAGTTCAA CAGCTGTTAC CTGGATGAGT ACATTGCATC AATGGTCCAC

Guinea_Pig TCAAGTTCAA CAGCTGTTAT CTTGATGAAC ACATCGCATC AATGGTTCAC

Horse TCAAATTCAA CAGCTGTTAC CTTGATGAAT ACATCGCATC GATGGTCCAC

Mouse TCAAGTTCAA CAGCTGTTAC CTTGACGAAT ACATTGCATC AATGGTTCAC

Pig TGAAGTTCAA CAGCTGTTAC CTTGACGAGT ACATCGCATC CATGGTCCAC

Rabbit ---------- ---------- ---------- ---------- ----------

Rat TCAAGTTCAA CAGCTGCTAC CTTGACGAAT ACATTGCGCC AATGGTTCAC

Elephant TCAAGTTCAA CAGCTGTTAC CTTGATGAAT ACATCGCATC CATGGTTCAC

Frog TTAAGTTCAA TAGCTGTTAC TTGGAACACT TTATTGCTCC TATGGTGCAG

Zebrafish TCAAATTCAA CTCCTGCTAC CTGGATGAGA ACGTTTCGCT TATGATAATG

Chicken TGAAATTCAA TAGCTGCTAT TTGGAAGACT ACGTAGCTGA CATGGTCCAC

Fugu TAAAATTCAA CAGCTGCTAC TTGGACCAAA ATGTCTCCTC CATGGTCCAG

Zebra_Finch TGAAATTCAA TAGCTGCTAT CTGGAAGACT ATGTAGCTGA CATGGTCCAC

Human ATGATCTGTC TGCTGTGCGT CCGGACCGCG TCCTCTGTGG ACATAGAGGT

Gorilla ATGATCTGTC TGCTGTGCGT CCGGACCGCG TCCTCTGTGG ACATAGAGGT

Chimpanzee ATGATCTGTC TGCTGTGCGT CCGGACCGCG TCCTCTGTGG ACATAGAGGT

Orangutan ATGATCTGTC TGCTCTGCGT CCGGACCGCA TCCTCTGTGG ACATAGAGGT

Marmoset ATGATCTGTC TGCTGTGTGT CCGGACCGCG TCCTCTGTGG ACATAGAGGT

Cow ATGGTCTGCC TGCTGTGTGT CCAGACCGTG TCCTCCGTGG ACATAGAGGT

Dog ATGATCTGCC TGCTGTGCGT CCAGACCGTG TCATCCGTGG ACATAGAGGT

Guinea_Pig ATGATCTGTC TGCTGTGCAT CCGGACCATG TCCTCTGTTG ACATAGAGGT

Horse ATGATCTGCC TGCTGTGCGT CCAGACCGTG TCCTCTGTGG ACATAGAGGT

Mouse ATGATTTGTC TGCTATGCAT CCGGACAGTG TCCTCTGTGG ACATTGAGGT

Pig ATGATCTGCC TGCTCTGCGT CCAGACCGTG TCCTCCGTGG ACATAGAGGT

Rabbit ---------- ---------- ---------- ---------- ----------

Rat ATGATTTGTC TGTTATGTAT CCGGACAGTG TCCTCTGTGG ACATCGAGGT

Elephant ATGATCTGTC TGTTGTGCAT CCGGACCTCG TCCTCCGTGG ACATAGAGGT

Frog ATGATATGCC TCCTCTGTAA TCGCACGACA TCTCCTTACG ATATTGAGGT

Zebrafish AAAATCTGCC TTCTGTGCAA CCGGACGACA TCCTCCACAG ACATTGAGGT

Chicken AAGATATGCA TCTTGTGTAT TCAGACTTCA TCATCTGTGG ACATAGAGAT

Fugu AATATTTGTC TCCTGTGCAA CAGAACAACA TCTTCTACTG ATATTGAGGT

Zebra_Finch AAGATCTGCC TCTTGTGCAT TCAGACTTCA TCATCTATGG ACATAGAGAT

Human CTCCCTGCAG GTGCTGGACG CCGTGGTCTG CTACAACTGC CTGCCGGCTG

Gorilla CTCCCTGCAG GTGCTGGACG CCGTGGTCTG CTACAACTGC CTGCCGGCTG

Chimpanzee CTCCCTGCAG GTGCTGGACG CCGTGGTCTG CTACAACTGC CTGCCGGCTG

Orangutan CTCCCTGCAG GTGCTGGACG CTGTGGTCTG CTACAACTGC CTGCCGGCTG

Marmoset CTCCCTGCAG GTGCTGGACG CTGTGGTCTG CTACAACTGC CTGCCGGCCG

Cow GTCTCTGCAG GTGCTGGATG CTGTGGTCTG CTACAACTGT CTGCCAGCCG

Dog CTCCCTGCAG GTGCTGGACG CCGTCGTCTG CTACAACTGC CTGCCGGCTG

Guinea_Pig CTCCCTGCAG GTGCTTGATG CTGTGGTTTG CTACAACTGC CTGCCAGCTG

Horse CTCTCTGCAG GTGCTGGATG CCGTGGTCTG CTACAACTGC CTGCCGGCCG

Mouse GTCCTTGCAA GTGCTGGATG CTGTGGTCTG CTACAACTGC TTACCAGCCG

Pig CTCTCTGCAG GTGCTGGACG CTGTGGTCTG CTACAACTGC CTGCCGGCGG

Rabbit ---------- ---------- ---------- ---------- ----------

Rat GTCCTTGCAA GTGCTGGATG CTGTGGTCTG CTACAACTGC CTGCCTGCTG

Elephant GTCCCTGCAG GTGCTGGATG CCGTGGTCTG CTACAACTGC CTGCCTGCCG

Frog TTCCTTGAAG GTTCTGGATG CAGTGGTCTG CTATAACTGC CTTCCATCAG

Zebrafish TGCGCTGCAG GTGCTGGACG CGGTGGTGTG TTATAACTGT CTGCCATCAG

Chicken TTCTTTGCAA GTTTTAGATG CTGTTGTTTG CTATAACTGT CTGCCGTCAG

Fugu GGCGCTCCAA GTGCTAGATG CTGTTGTGTG CTACAACTGT TTACCCTCAG

Zebra_Finch TTCCTTGCAA GTCCTGGATG CAGTTGTTTG TTACAACTGC CTGCCCTCGG

Human AGAGCCTCCC GCTGTTCATC GTTACCCTCT GTCGCACCAT CAACGTCAAG

Gorilla AGAGCCTCCC GCTGTTCATC GTTACCCTCT GTCGCACCAT CAACGTCAAG

Chimpanzee AGAGCCTCCC GCTGTTCATC GTTACCCTCT GTCGCACCAT CAACGTCAAG

Orangutan AGAGCCTCCC GCTGTTCATC GTTACTCTCT GTCGCACCAT CAACGTCAAG

Marmoset AGAGCCTCCC AGTGTTCATT GTCACTCTCT GTCGCACCGT CAACGCCAAG

Cow AGAGCCTCCC CCTATTCATC GTCACTCTCT GCCGCACCAT CAACGTCAAG

Dog AGAGCCTCCC CCTGTTCATC GTCACCCTGT GCCGCACCAT CAACGTGAAG

Guinea_Pig AGAGCCTCCC TCTGTTCATT GTCACCCTGT GTCGCACCAT AAATGTCAAG

Horse AGAGCCTCCC CCTCTTCATC GTCACCCTCT GCCGCACCAT CAATGTCAAG

Mouse AGAGCCTGCC TCTGTTCATT ATCACCCTGT GCCGCACCAT CAATGTCAAG

Pig AGAGCCTCCC CCTCTTCATT GTCACACTTT GCCGCACCAT CAACGTCAAG

Rabbit ---------- ---------- ---------- ---------- ----------

Rat AGAGCCTGCC TCTGTTCATT ATCACCCTGT GCCGCACCGT CAACGTCAAG

Elephant CGAGCCTGCC CCTATTCATT GTCACATTGT GCCGCACCGT CAACGTCAAG

Frog AGAGCATTCC TATATTTGTT ATGACCCTGT GCCGCACAAT AAATGTTAAG

Zebrafish ACTCTCTAAC AGTCTTCATC ATCACTCTGT GCCGAACTGT AAATGTAAAG

Chicken AGAACCTGCC AGTATTTATC ATCACTTTGT GCCGTACCAT CAATGTGAAG

Fugu ACTCCCTCAC CATCTTCATT ATTACCCTTT GTCGTACAGT CAATGTGAAG

Zebra_Finch AGAACCTGCC CGTGTTCATC ATCACCCTGT GCCGCACCAT CAACGTGAAG

Human GAGCTCTGCG AGCCTTGCTG GAAGCTGATG CGGAACCTCC TTGGCACCCA

Gorilla GAGCTCTGCG AGCCTTGCTG GAAGCTGATG CGGAACCTCC TTGGCACCCA

Chimpanzee GAGCTCTGCN AGCCTTGCTG GAAGCTGATG CGGAACCTCC TTGGCACCCA

Orangutan GAGCTCTGCG AGCCTTGCTG GAAGCTGATG CGGAACCTCC TTGGCACCCA

Marmoset GAGCTCTGCG AGCCTTGCTG GAAGCTGATG CGGAACCTCC TTGGCACCCA

Cow GAGCTCTGTG AACCTTGCTG GAAGCTGATG CGCAACCTCC TGGGCACCCA

Dog GAGCTCTGTG AGCCTTGCTG GAAGCTGATG CGGAACCTGC TGGGCACCCA

Guinea_Pig GAACTCTGCG AGCCTTGCTG GAAGCTGATG CGAAACCTCT TGGGCACCCA

Horse GAGCTCTGTG AGCCTTGCTG GAAGCTGATG CGGAACCTCC TGGGCACCCA

Mouse GAGCTGTGTG AGCCTTGCTG GAAGTTGATG CGTAACCTTC TGGGCACCCA

Pig GAGCTCTGCG AGCCGTGCTG GAAGCTGATG CGTAATCTCC TGGGCACCCA

Rabbit ---------- ---------- ---------- ---------- ----------

Rat GAGCTGTGTG AGCCGTGCTG GAAGCTGATG CGAAACCTTC TAGGTACCCA

Elephant GAGCTCTGCG AGCCCTGCTG GAAGCTGATG CGTAACCTCC TGGGAACCCA

Frog GAGCTGTGTG AACCATGCTG GAAGCTGATG CGAAACCTTC TGGGCACCCA

Zebrafish GAGTTCTGCG AGTCCTGCTG GAAGTTGATG CGCAAAGTGT TGGGAACTCA

Chicken GAGCTCTGTG AGCCATGCTG GAAGCTTATG CGCAACCTTT TGGGAACTCA

Fugu GAATTCTGTG AGGCTTGTTG GAAGCTAATG AGGAAGGTAT TGGGGACTCA

Zebra_Finch GAGCTCTGTG AGCCCTGCTG GAAGCTCATG CGCAACCTCT TGGGAACTCA

Human CCTGGGCCAC AGCGCCATCT ACAACATGTG CCACCTCATG GAGGACAGAG

Gorilla CCTGGGCCAC AGCGCCATCT ACAACATGTG CCACCTCATG GAGGACAGAG

Chimpanzee CCTGGGCCAC AGCGCCATCT ACAACATGTG CCACCTCATG GAGGACAGAG

Orangutan CCTGGGCCAC AGCGCCATCT ATAACATGTG CCACCTCATG GAGGACAGAG

Marmoset CCTGGGTCAC AGCGCCATCT ACAACATGTG CCACCTCATG GAGGACAGAG

Cow CCTGGGCCAC AGCGCCATCT ACCACATGTG CCGCATCATG GAAGACAGAG

Dog CCTGGGCCAC AGTGCCATCT ACAACATGTG TCGCATCATG GAGGACAGAG

Guinea_Pig CCTGGGCCAC AGTGCAATCT ATAACATGTG CCGAATCATG GAGGACAGAG

Horse CCTGGGCCAC AGTGCCATCT ACAACATGTG CCGCCTCATG GAGAACAGGG

Mouse CCTGGGCCAC AGCGCCATCT ACAACATGTG CCGCATCATG GAGGACAGAT

Pig CCTGGGCCAC AGCGCCATCT ACCACATGTG CCGCCTCATG GAGGACAGGG

Rabbit ---------- ---------- ---------- ---------- ----------

Rat TCTAGGCCAC AGTGCCATTT ACAACATGTG CCGTATCATG GAGAACAGAT

Elephant CCTGGGCCAC AGTGCCATCT ACAACATGTG CCGCATCATG GAGGACAGAG

Frog TCTTGGGCAC AGCGCCATTT ACACTATGAG TCGTATAATG GAGGATAGAG

Zebrafish TCTGGGTCAC AGTGCCATAT ACACCATGTG CCGAATCATG GAGGAGAGGG

Chicken TTTGGGACAC AGTGCAATTT ACAACATGTG CAGGATCATG GAAGACAGAG

Fugu CCTTGGTCAC AGTGCCATTT ACACCATGTG CCGCATCATG GAGGAAAGGG

Zebra_Finch CCTGGGGCAC AGTGCCATCT ACAACATGTG CAGGATCATG GAAGACAGAT

Human CCTACATGGA GGACGCGCCC CTGCTGAGAG GAGCCGTGTT TTTTGTGGGC

Gorilla CCTACATGGA GGACGCGCCC CTGCTGAGAG GAGCCGTGTT TTTTGTGGGC

Chimpanzee CCTACATGGA GGACGCGCCC CTGCTGAGAG GAGCTGTGTT TTTTGTGGGC

Orangutan CCTACATGGA GGATGCGCCC CTGCTGAGAG GAGCCGTGTT TTTTGTGGGC

Marmoset CCTACATGGA AGACGCCCCC CTGCTGAGAG GAGCCGTGTT TTTTGTGGGC

Cow CCTACATGGA GGACGCACCA CTGCTGAGAG GAGCCGTGTT CTTTGTTGGC

Dog CCTACATGGA AGATGCCCCA CTGCTGAGAG GGGCTGTGTT CTTTGTGGGG

Guinea_Pig CCTACATGGA AGATGCCCCA CTGCTGAGAG GAGCTGTATT CTTTGTGGGC

Horse CCTACATGGA GGACGCCCCG CTGCTGAGAG GAGCTGTGTT CTTTGTTGGC

Mouse CCTACATGGA AGATGCCCCA CTGCTGAGAG GAGCTGTGTT CTTTGTAGGG

Pig CCTTCATGGA AGACGCCCCG CTGCTGCGGG GAGCCGTGTT CTTTGTGGGC

Rabbit ---------- ---------- ---------- ---------- ----------

Rat CCTACATGGA AGACGCCCCC TTGTTGAGGG GAGCCGTGTT CTTTGTTGGG

Elephant CCTATATGGA GGATGCCCCG CTGCTGAGAG GAGCTGTGTT CTTTGTCGGC

Frog CCCACATGGA AGATGCCCCT TTGCTAAGAG GAGCTGTGTT CTTTGTTGGG

Zebrafish TGTACTCCGA GGACGCCGCT CTCCTCCGCG GTGCTGTGTT TTTTGTTGGG

Chicken CTTACATGGC AGATGCAGCC CTGCTGAGAG GAGCGGTGTT CTTTGTTGGG

Fugu TGTACACGGA GGACGCACCA TTGTTAAGAG GAGCCGTCTT CTTTGTTGGA

Zebra_Finch CCTACATGGC AGATGCAGCC CTGCTGAGAG GAGCTGTGTT CTTTGTTGGG

Human ATGGCTCTCT GGGGAGCCCA CCGGCTCTAT TCTCTCAGGA ACTCGCCGAC

Gorilla ATGGCTCTCT GGGGAGCCCA CCGGCTCTAT TCTCTCAAGA ACTCGCCGAC

Chimpanzee ATGGCTCTCT GGGGAGCCCA CCGGCTCTAT TCTCTCAGGA ACTCGCCGAC

Orangutan ATGGCTCTCT GGGGAGCCCA CCGGCTCTAT TCTCTGAGGA ACTCGCCGAC

Marmoset ATGGCTCTCT GGGGAGCCCA CCGGCTCTAT TCTCTCAGGA ACTCGCCGAC

Cow ATGGCTCTCT GGGGTGCCCA CCGGCTCTAT TCTCTCAAGA ACTCCCCAAC

Dog ATGGCCCTTT GGGGAGCACA CCGACTCTAC TCTCTCAAGA ACTCCCCAAC

Guinea_Pig ATGGCACTTT GGGGAGCCCA CCGACTTTAT TCTCTCAAGA ACTCCCCAAC

Horse ATGGCTCTAT GGGGAGCCCA CCGGCTCTAT ACTCTCAAGA ACTCCCCGAC

Mouse ATGGCACTCT GGGGAGCTCA CCGGCTCTAC TCTCTCAAGA ACTCCCCCAC

Pig ATGGCTCTGT GGGGAGCCCA CCGGCTCTAT TCGCTCAGGA ACTCCCCGAC

Rabbit ---------- ---------- ---------- ---------- ----------

Rat ATGGCGCTCT GGGGAGCTCA CCGACTCTAT TCGCTCAAGA ACTCCCCCAC

Elephant ATGGCGCTGT GGGGGGCCCA CCGACTCTAC TCCCTCAAGA ACTCCCCTAC

Frog ATGGGTCTCT GGGGAGCTCA CAGACTCTTC TGTCTGAAGA ACTCCCCGAT

Zebrafish ATGGCGCTGT GGGGCGCTCA CAGACTCCCC GCCCTCAAAA ACACACCTAC

Chicken ATGGCTCTCT GGGGTGCTCA TCGCCTCAAT TCACTCAAGA ATTCCCCAAC

Fugu ATGGCACTAT GGGGTGCACA CAGACTCCCT GCCCTTAAAA ACACACCTAC

Zebra_Finch ATGGCTCTGT GGGGGGCTCA TCGCCTCAAT TCCCTCAGGA ACTCCCCCAC

Human ATCTGTGTTG CCATCATTTT ACCAGGCCAT GGCATGTCCG AACGAGGTGG

Gorilla ATCCGTGTTG CCATCATTTT ACCAGGCCAT GGCATGTCCG AACGAGGTGG

Chimpanzee ATCTGTGTTG CCATCATTTT ACCAGGCCAT GGCATGTCCG AACGAGGTGG

Orangutan ATCTGTGCTA CCATCATTTT ACCAGGCCAT GGTATGTCCG AACGAGGTGG

Marmoset ATCCGTGCTG CCATCATTTT ACCAGGCTAT GGCGTGTGCG AATGAGGTGG

Cow ATCTGTGTTG CCATCGTTTT ATGAGGCCAT GACCTGTCCG AATGAGGTGG

Dog GTCTGTGTTG CCGTCGTTTT ATGAGGCTAT GACCTGTCCG AATGAAGTGG

Guinea_Pig ATCTGTATTG CCATCTTTTT ATGAGGCCAT GACTTGTCCC AATGAGGTGG

Horse GTCCGTGCTG CCATCGTTCT ATGAGGCCAT GACCTGTCCG AATGAGGTGG

Mouse ATCTGTGCTG CCGTCTTTTT ATGAGGCTAT GACCTGTCCC AATGAGGTGG

Pig GTCGGTGTTG CCGTCCTTCT ATGAGGCCAT GACCTGTCCG AACGAGGTGG

Rabbit ---------- ---------- ---------- ---------- ----------

Rat ATCTGTGCTG CCGTCTTTTT ATGAGGCCAT GACCTGTCCC AATGAGGTGG

Elephant ATCTGTGCTG CCGTCGTTTT ATGAGGCCAT GACCTGCCCC AATGAGGTGG

Frog ATCTGTTCTC CCATCTTTCC TGAAGGCTAT GAACTGTAAC AATGCCGTTG

Zebrafish GCTGGTCCTG CCTTCCTTCT ACAAGGCCAT GAGCTGTGCT AATGAGGTGG

Chicken TTCAGTGCTG CCTTCGTTCC TCAAGGCCAT GACGTGTCCC AATGCAGTTG

Fugu ACTTGTGCTA CCATCTTTCT ATAAGGCCAT GTCCAGTGCC AACGAAGTGG

Zebra_Finch TTCAGTGCTG CCTTCATTCC TCAAGGCCAT GACCTGTCCC AACGCAGTCG

Human TGTCCTATGA GATCGTCCTG TCCATCACCA GGCTCATCAA GAAGTATAGG

Gorilla TGTCCTATGA GATCGTCCTG TCCATCACCA GGCTCATCAA GAAGTATAGG

Chimpanzee TGTCCTATGA GATCGTCCTG TCCATCACCA GGCTCATCAA GAAGTATAGG

Orangutan TGTCCTATGA GATCGTCCTG TCCATCACCA GGCTCATCAA GAAGTACAGG

Marmoset TGTCCTATGA GATTGTCCTG TCAATCACCA GGCTCATCAA GAAGTACAGG

Cow TGTCCTACGA GATTGTCCTC TCCATAACCA GACTCATCAA GAAGTACAGG

Dog TGTCCTATGA AATCGTCCTC TCTATAACCA GACTCATCAA GAAGTACCGG

Guinea_Pig TGTCCTATGA GATCGTGTTG TCCATAACCA GACTCATCAA GAAGTACAGG

Horse TGTCCTATGA GATTGTCCTC TCCATAACAA GACTCATCAA GAAGTACAAG

Mouse TGTCATATGA GATTGTTCTG TCCATAACAA GACTCATCAA GAAGTATAGG

Pig TGTCCTACGA GATCGTCCTG TCCATAACCC GACTCATCAA GAAGTACCGG

Rabbit ---------- ---------- ---------- ---------- ----------

Rat TGTCATATGA GATTGTTCTG TCCATAACAA GACTCATCAA GAAGTACAGG

Elephant TGTCCTACGA AATCGTCCTC TCTATCACCA GACTCATCAA GAAGTACAGG

Frog TCTCCTATGA GATTGTCCTC TCTGTCACTC GGCTAATCAA GAAGTACGGC

Zebrafish TGTCCTATGA GATCGTGCTC TCCATCACGC GCCTCATTAA AAAATACGGC

Chicken TATCTTATGA GATAGTTCTG TCCATCACCC GTCTAATAAA GAAGTATGGG

Fugu TGTCCTATGA AATCGTCCTC TCCATCACCA GACTCATCAA GAAGTATGGC

Zebra_Finch TGTCCTACGA GATCGTTCTG TCCATCACAC GCCTGATCAA GAAATATGGC

Human AAGGAGCTCC AGGTGGTGGC GTGGGACATT CTGCTGAACA TCATCGAACG

Gorilla AAGGAGCTCC AGGTGGTGGC GTGGGACATT CTGCTGAACA TCATCGAACG

Chimpanzee AAGGAGCTCC AGGTGGTGGC GTGGGACATT CTGCTGAACA TCATCGAACG

Orangutan AAGGAGCTCC AGGTGGTGAC GTGGGACATT CTGCTGAACA TCATCGAACG

Marmoset AAGGAGCTCC AGGCGGTGAC ATGGGACATT CTGCTGAACA TCATCGAACG

Cow AGGGAGCTCC AGGCCGTGAC CTGGGACATC CTGCTGAACA TCATCGAGCG

Dog AGGGAGCTGC AAGCTGTGAC CTGGGACATC CTACTGAACA TCATGGAGCG

Guinea_Pig AAGGAGCTCC AGGCAGTGAC ATGGGACATT CTGCTGAATA TCATGGAACG

Horse AGGGAGCTTC AGGCTGTGAC GTGGGACATC CTGCTGAACA TTATTGAACG

Mouse AAGGAGCTCC AGGCTGTGAC ATGGGATATT CTGCTGGACA TCATTGAACG

Pig CGGGAGCTCC AGGCCGTGAC CTGGGACGTC CTGCTGAACA TCATTGAACG

Rabbit ---------- ---------- ---------- ---------- ----------

Rat AAGGAGCTCC AGGCTGTGAC ATGGGACATT CTGCTGGACA TCATTGAACG

Elephant AAGGAGCTGC AGGCTGTGAC GTGGGACATC CTGCTGAGCA TCATCGAACG

Frog AAGGATGTGC AGGCTGCCAC GTGGGACGTT CTCCTTGATA TCATTGAGCG

Zebrafish CGCGAGCTGC AGGTGGTGAC CTGGGACATC CTGCTGTCCA TCATAGACAG

Chicken AAAGAACTGC AAGCTGTAAC CTGGGATATC CTCTTGGACA TCATGGAGCG

Fugu AAAGAGCTAC AGGTGGTCAC ATGGGACATC TTACTGGGAA TCATTGAGAG

Zebra_Finch AAGGGGCTGC AGGCTGTGAC CTGGGATATC CTCCTGGACA TCATGGAGAG

Human GCTCCTTCAG CAGCTCCAGA CCTTGGACAG CCCGGAGCTC AGGACCATCG

Gorilla GCTCCTTCAG CAGCTCCAGA CCTTGGACAG CCCGGAGCTC AGGACCATCG

Chimpanzee GCTCCTTCAG CAGCTCCAGA CCTTGGACAG CCCGGAGCTC AGGACCATCG

Orangutan GCTCCTTCAG CAGCTCCAGA CCTTGGACAG CCCGGAGCTC AGGACCGTTG

Marmoset GCTCCTTCAG CAGCTCCAGA CTCTGGACAG CCTGGAGCTC AGGACCATCG

Cow CCTGCTCCAG CAGCTCCAGA GCCTGGACAG CCCAGAGCTG AGCGCCATCG

Dog CCTCCTGCAC CAGCTCCAGA GCCTGGACAG CCCGGAGCTC AGAGCCATCG

Guinea_Pig GCTGCTTCAG CAGCTCCAGG ACCTGGACAG CCCAGAGCTC AGGACCATCA

Horse CCTGCTTCAG CAGCTCCAGA GCCTGGACAG CCCGGAACTC AGGACCATCG

Mouse ACTACTTCAG CAACTCCAGA ACCTGGACAG CCCGGAACTC AAGACCATCG

Pig CCTGCTCCAG CAGCTCCAGA GCCTGGACAG TCCGGAACTC ACAGCCATCG

Rabbit ---------- ---------- ---------- ---------- ----------

Rat ACTGCTCCAG CAACTCCAGA ACCTGGACAG CCCGGAACTC AGGACCATTG

Elephant TCTGCTCCAG CAGCTCCTGA CCCTCGAGAG TCAGGAGCTC AGAGCCATCG

Frog ATTATTCCAG CAGCTTCAGG TGCTGCGGAG TCAGGAATTA AAGGCTGCGG

Zebrafish ACTCCTGCAG CAGATACAGA CGATGGGCAG TCCTGACCTG AAGGTGATCG

Chicken ATTACTGCAA CAGTTGCAGA GTTTAGAGAG CCAAGAACTT AAGTCCATTG

Fugu ACTCCTACAG CAAATCCAGA CCATAGGTAG TGCAGAGTTG AAGGCCATCG

Zebra_Finch GCTGCTGCAG CAGCTGCAGG TTCTGGAGAG CCAAGAACTG AAGTCCATTG

Human TCCATGACCT GTTGACCACG GTGGAGGAGC TGTGTGACCA GAACGAGTTC

Gorilla TCCATGACCT GTTGACCACG GTGGAGGAGC TGTGTGACCA GAACGAGTTC

Chimpanzee TCCATGACCT GTTGACCACG GTGGAGGAGC TGTGTGACCA GAACGAGTTC

Orangutan TCCATGACCT GTTGACCACG GTGGAGGAGC TGTGTGACCA GAACGAGTTC

Marmoset TCCATGACCT GCTGACCACG GTGGAGGAGC TGTGTGACCA GAACGAGTTC

Cow TGCACGATCT GCTGAGCACA GTGGAGGAGC TGTGTGACCA GAACGAGTTT

Dog TGCACGACCT GCTGACCACA GTGGAAGAGC TGTGTGATCA GAATGAGTTC

Guinea_Pig TCCATGATCT GCTGACCACT GTGGAAGAGC TGTGTGACCA GAATGAGTTC

Horse TCCATGACCT GCTGACCACG GTGGAAGAAC TGTGTGATCA GAATGAGTTC

Mouse TCCATGACCT GCTGACCACT GTGGAGGAGC TATGTGACCA GAACGAGTTC

Pig TCCACGACCT GCTGACCACA GTGGAGGAGC TGTGTGATCA GAACGAGTTC

Rabbit ---------- ---------- ---------- ---------- ----------

Rat TCCATGACCT GCTGACCACT GTGGAGGAGC TGTGTGACCA GAACGAGTTC

Elephant TGCATGACCT GCTGACGACG GTGGAGGAGC TGTGTGACCA GAATGAGTTC

Frog TGCACAATCT CATGACCACC ACAGAGGAGC TTTATAACCA GAACGGATTC

Zebrafish TCTATGAGCT GCTGAGCACC ATCGAGGAGC TTTACGAGCA GAACGACTTC

Chicken TACATGATCT TTTGACTACA GTAGAAGAAC TCTGTGACCA GAATGAATTT

Fugu TCTATGAGCT GTTGACCACC GTGGAGGAGC TGTATGAGCA GAACAGTTAC

Zebra_Finch TACATGATCT TTTGACCACA GTAGAAGAGC TCTGTGACCA GAATGATTTT

Human CACGGGTCTC AGGAGAGATA CTTTGAACTG GTGGAGAGAT GTGCGGACCA

Gorilla CATGGGTCTC AGGAGAGATA CTTTGAACTG GTGGAGAGAT GTGCGGACCA

Chimpanzee CACGGGTCTC AGGAGAGATA CTTTGAACTG GTGGAGAGAT GTGCGGACCA

Orangutan CACGGGTCTC AGGAGAGATA CTTTGAACTG GTGGAGAGAT GTGCGGACCA

Marmoset CACGGTTCCC AGGAGAGATA CTTTGAGCTG GTGGAGAGCT GTGCAGACCA

Cow CACGGCTCCC AGGAGAGATA CTTTGAACTC GTGGAGAGGT GTGCGGACCA

Dog CACGGCTCTC AGGAGAGATA CTTTGAGCTG GTTGAGCGAT GTGCAGACCA

Guinea_Pig CATGGCTCGC AGGAAAGATA TTTCGAGTTG GTGGAGAGCT GTGCGGACCA

Horse CACGGCTCCC AGGAGAGATA CTTTGAGCTT GTTGAGAGAT GTGCAGACCA

Mouse CATGGCTCGC AGGAAAGATA CTATGAACTG GTGGAGAGCT ATGCAGACCA

Pig CATGGCTCTC AGGAGAGATA CTTCGAGCTC GTTGAGAGAT ATGCGGACCA

Rabbit ---------- ---------- ---------- ---------- ----------

Rat CACGGATCGC AAGAAAGATA CTATGAACTG GTGGAGAGCT ATGCAGACCA

Elephant CACGGCTCAC AGGAGAGATA CTTTGAGCTC ATTGAGCGAT GCGCCGACCA

Frog CATGGAGATG AAGAGAGATT TTTCGAGCTG GTTGAAAAAG GGGCAGATGA

Zebrafish CACGGCTCCT CGGCCCGCTT CTTCAGCCTG GTGGAGAAGT GTGCGGATAA

Chicken CATGGTTCTG AGGAGAGATT TTTTGAGCTG GTGGAAAGAT GCGCTGATCA

Fugu CATGGTTCAA TGGAGAAGTT CTTCAATCTG GTGGAGAAAT GTGCTGACAA

Zebra_Finch CATGGCTCTG AAGAGAGATT TTTTGAGCTG GTGGAAAGAT GTGCTGATCA

Human GAGGCCTGAG TCCTCCCTCC TGAACCTGAT CTCCTATAGA GCGCAGTCCA

Gorilla GAGGCCTGAG TCCTCCCTCC TGAACCTGAT CTCCTATAGA GCGCAGTCCA

Chimpanzee GAGGCCTGAG TCCTCCCTTC TGAACCTGAT CTCCTATAGA GCGCAGTCCA

Orangutan GAGGCCGGAG TCCTCCCTCC TGAACCTGAT CTCCTATAGA GCGCAGTCCA

Marmoset GAGGCCTGAG TCCTCCCTCC TGAACCTCAT CTCGTATAGA GCGCAGTCCA

Cow GAGGCCTGAG TCATCCCTTT TAAACTTAAT AACCTACAGG GCTCAGTCCA

Dog GAGGCCGGAG TCTTCCCTCT TAAACTTAAT AACGTACCGG GCTCAGTCAA

Guinea_Pig GAGGCCTGAG TCCTCCCTCT TAAACTTAAT AACCTACAGA GCTCAGTCCA

Horse GAGGCCTGAG TCCTCCCTCT TAAACTTAAT AACCTACAGG GCTCAGTCAA

Mouse GAGACCTGAA TCCTCTCTCT TAAACTTGAT ATCCTACAGA GCCCAGTCCA

Pig GAGGCCTGAG TCCTCACTCC TAAATTTAAT AACCTACAGG GCCCAGTCCA

Rabbit ---------- ---------- ---------- ---------- ----------

Rat GAGGCCTGAA TCCTCTCTCT TAAACCTGAT AACCTACAGA GCCCAGTCTA

Elephant GAGACCTGAG TCCTCCCTCT TGAACCTGAT ATCCTACAGG GCACAGTCCA

Frog AAGACCTTTT GCATCAGTTG TGAACCTTAT CACCTACAGA GCTCAGTCCA

Zebrafish ACGTCCTGAT GCATCTGTGC TCACGCTGGT CTCCTACAGA GCTCAGGCCA

Chicken GAGACCGGAA TCTTCCGTAT TAAACTTGAT AGCATACAGA GCTCAATCCA

Fugu GAGACCTGAT GCATCGGTGC TGACCCTCAT CTCATACAGA GCTCAGTCAA

Zebra_Finch GAGACCAGAA TCTTCTGTGT TAAACCTGAT AACATACAGA GCTCAGTCCA

Human TCCACCCGGC CAAGGACGGC TGGATTCAGA ACCTGCAGGC GCTGATGGAG

Gorilla TCCACCCGGC CAAGGACGGC TGGATTCAGA ACCTGCAGGC GCTGATGGAG

Chimpanzee TCCACCCGGC CAAGGACGGC TGGATTCAGA ACCTGCAGGC GCTGATGGAG

Orangutan TCCACCCGGC CAAGGACGGC TGGATTCAGA ACCTGCAGGC GCTGATGGAG

Marmoset TCCACCCGGC CAAGGATGGC TGGATTCAGA ACCTGCAGGC ACTGATGGAG

Cow TCCATCCAGC GAAGGACGGC TGGATCCACA ACCTGCAGCT GCTGATGGAG

Dog TCCACCCGGC GAAGGATGGC TGGATTCAGA ACCTGCAGTT ACTGATGGAG

Guinea_Pig TTCATCCAGC AAAGGATGGC TGGATCCAGA ACCTACAGTT GCTGATGGAG

Horse TCCATCCTGC AAAGGACGGC TGGATTCACA ACCTACAGTT ACTGATGGAG

Mouse TCCACCCTGC CAAGGATGGC TGGATCCAGA ACTTGCAGTT GTTGATGGAG

Pig TCCATCCGGC CAAGGATGGC TGGATTCACA ACCTGCAGCT GCTGATGGAG

Rabbit ---------- ---------- ---------- ---------- ----------

Rat TCCACCCAGC CAAGGATGGC TGGATCCAGA ACCTGCAGTT GTTGATGGAG

Elephant TCCACCCAGC CAAGGATGAC TGGATCCACA ACCTGCAGCA GTTAATGGAG

Frog TTCACCCTGG GAAGGAAGGG TGGATTATGA ATCTACAGAA ACTGATGGAT

Zebrafish TCCAGCCGGC CAAGGACGGA TGGCTGCAGA ACCTGCTCAA ACTCATGGAC

Chicken TTCATCCTGC CAAAGATGGC TGGATTCACA ACCTGCAGAT GTTAATGGAG

Fugu TACAGCCAGC CAAGGATGGG TGGATTCAGA GTCTCCATCG CCTCATGGAC

Zebra_Finch TCCACCCTGC CAAAGATGGC TGGATCCACA ACCTGCAGCT CTTAATGGAG

Human AGATTCTTCA GGAGCGAGTC CCGAGGCGCC GTGCGCATCA AGGTGCTGGA

Gorilla AGATTCTTCA GGAGCGAGTC CCGAGGCGCC GTGCGCATCA AGGTGCTGGA

Chimpanzee AGATTCTTCA GGAGCGAGTC CCGAGGCGCC GTGCGCATCA AGGTGCTGGA

Orangutan AGATTCTTCA GGAGCGAGTC CCGAGGCGCC GTGCGCATCA AGGTGCTGGA

Marmoset AGATTCTTCA GGAGCGAGTC CCGAGGCGCC GTGCGCATCA AGGTGCTGGA

Cow AGGTTTTTCA GGAACGAGTC ACGCAGTGCC GTGCGCATCA AGGTGCTGGA

Dog AGGTTCTTCA GGAACGAATC TCGCAGTGCT GTGCGCATCA AGGTGCTGGA

Guinea_Pig AGGTTCTTCA GGAATGAGTC CCGCAGTGCT GTTCGCATCA AGGTGTTGGA

Horse AGATTCTTCA GGAACGAATC TCGCAGCGCT GTGCGCATCA AGGTGCTGGA

Mouse AGGTTCTTCA GGAATGAGTG CCGCAGCGCC GTGCGCATCA AGGTGTTGGA

Pig CGATTCTTCA GGAATGAGGC GCGCAGCGCT GTGCGGATCA AGGTACTGGA

Rabbit ---------- ---------- ---------- ---------- ----------

Rat AGGTTCTTCA GGAACGAGTG CCGCAGTGCC GTGCGCATCA AGGTGCTGGA

Elephant AGGTTCTTCA GGAACGAGTC CCGCAGTGCT GTGCGCATCA AGGTGCTGGA

Frog AGATATTTCA GGAGCGAGTC TCGCAGTGTT GTACGCATGA AAATCTTAGA

Zebrafish AAGTTCTTCA GGAATGAGAG CCGCACTATG ATCAGGATCA AGGTTCTGCA

Chicken AGATTCTTCA GGAACGAAAG TCGTAGTGCT GTTCGTATTA AAGTTCTGGA

Fugu AAGTTCTTCA GGAACGAGTC TCGAACTGTG ATAAGGATCA AAGTGCTTCA

Zebra_Finch AGGTTCTTCA GGAACGAGAG CCGTAGTGCT GTTAGGATTA AGGTTCTGGA

Human CGTGCTGTCC TTTGTGCTGC TCATCAACAG GCAGTTCTAT GAGGAGGAGC

Gorilla CGTGCTGTCC TTTGTGCTGC TCATCAACAG GCAGTTCTAT GAGGAGGAGC

Chimpanzee CGTGCTGTCC TTTGTGCTGC TCATCAACAG GCAGTTCTAT GAGGAGGAGC

Orangutan CGTGCTGTCT TTTGTGCTGC TCATCAACAG GCAGTTCTAT GAGGAGGAGC

Marmoset TGTGCTGTCC TTCGTGCTGC TCATCAACAG GCAATTCTAT GAGGAGGAGC

Cow CGTCCTGTCC TTCGTGCTGC TCATCAACAG GCAGTTCTAC GAGGAGGAGC

Dog CGTCCTGTCC TTCGTGCTGC TCATCAACAG GCAGTTCTAT GAGGAGGAGC

Guinea_Pig TGTGCTATCC TTTGTGCTGC TCATTAACAG GCAGTTCTAT GAGGAGGAAC

Horse TGTCCTGTCC TTCGTGTTGC TCATCAACAG GCAGTTCTAC GAGGAGGAGC

Mouse TGTTCTGTCC TTCGTGCTGC TGATCAACAG GCAGTTCTAT GAGGAGGAGC

Pig CGTCCTGTCC TTCGTGTTGC TCATCAACAA ACAGTTCTAC GAGGAGGAGC

Rabbit ---------- ---------- ---------- ---------- CAGGAGGAGC

Rat CGTGCTGTCC TTCGTGCTCC TCATCAACAG GCAGTTCTAC GAGGAGGAGC

Elephant CATCCTGTCC TTTGTGCTGC TTATCAACCG GCAGTTCTAT GAGGAGGAGC

Frog TGTTCTGTCC TTTGCGTTGA GCATCAATCG CCAGTTTTAC GAGGATGAGT

Zebrafish CATCTTGTCG TTCGTCCTCA GCACAAACCG TCAGCTGTAT GAGGAGGAGC

Chicken TGTCTTGTCC TTTGTACTGA GTATTAACAG ACAGTTCTAT GAGGAAGAGC

Fugu TATCCTGTCG TTCGTGCTCA GCACCAACCG ACAGCTCTAT GAGGACGAGT

Zebra_Finch TGTGCTGTCC TTTGTGCTGA GCATCAACAG ACAGTTCTAT GAGGAAGAGC

Human TGATTAACTC AGTGGTCATC TCGCAGCTCT CCCACATCCC CGAGGATAAA

Gorilla TGATTAACTC AGTGGTCATC TCTCAGCTCT CCCACATCCC CGAGGATAAA

Chimpanzee TGATTAACTC AGTGGTCATC TCGCAGCTCT CCCACATCCC CGAGGATAAA

Orangutan TGATTAACTC AGTGGTCATC TCGCAGCTCT CCCACATCCC CGAGGATAAA

Marmoset TGATTAACTC GGTGGTCATC TCCCAGCTCT CCCACATCCC CGAGGATAAA

Cow TTATCAACTC AGTGGTCATC TCGCAGCTCT CCCACATCCC TGAGGATAGA

Dog TGATCAGCTC GGTGGTCATC TCACAGCTCT CCCATGTCCC GGAGGACAAA

Guinea_Pig TCATTAACTC AGTGGTCATC TCGCAGCTGT CCCTCATCCC TGAAGACAGA

Horse TGATCAACTC AGTGGTCATC TCACAGCTCT CCCACATCCC TGAGGATAAA

Mouse TGATTAACTC GGTGGTCATC TCGCAGCTCT CCCACATTCC CGAGGATAAG

Pig TCATCAACTC GGTGGTCATC TCACAGCTCT CCCACATCCC CGAGGACAGA

Rabbit TGATCAACAC GGTGGTCATC TCCCAGCTCT CCCACATCCC CGAGGATAAC

Rat TGATTAACTC GGTGGTCATC TCACAGCTCT CCCACATCCC CGAGGATAAA

Elephant TGATCAACTC AGTGGTCATC TCCCAACTCT CCCACATTCC AGAAGACAGA

Frog TGATCGAGAA GGTAGTTACT TGCCAGCTAG CCCATATACC CGAGGATAAG

Zebrafish TGATTGAGGT GGTGGTCATC CCTCAGCTGG GTCAGATAGC TGAGGATCGT

Chicken TGATAAACTT GGTTGTGATC TCTCAGCTGG CTCACATTCC AGAGGATAAA

Fugu TGATTGAAAC CGTGGTGATC CCTCAGCTCA GCGGGATAGC TGAAGATCGG

Zebra_Finch TGATAAACTT GGTGGTGATC TCCCAGCTGG CTCACATCCC AGAGGATAAA

Human GACCACCAGG TCCGAAAGCT GGCCACCCAG TTGCTGGTGG ACCTGGCAGA

Gorilla GACCACCAGG TCCGAAAGTT GGCCACCCAG TTGCTGGTGG ACCTGGCAGA

Chimpanzee GACCACCAGG TCCGAAAGCT GGCCACCCAG TTGCTGGTGG ACCTGGCAGA

Orangutan GACCACCAGG TCCGAAAGCT GGCCACCCAG TTGCTGGTGG ACCTGGCAGA

Marmoset GACCACCAGG TCCGAAAGCT GGCCACCCAG TTGCTGGTGG ACCTGGCAGA

Cow GACCACCAGG TCCGGAAGCT GGCCACTCAG CTGCTGGTAG ATCTGGCCGA

Dog GACCCCCAAG TCCGGAAACT GGCCACCCAG TTGCTGGTGG ACCTGGCCGA

Guinea_Pig GACCCCCAAG TCCGAAAGCT GGCCACCCAA TTGCTGGTGG ACCTCGCGGA

Horse GACCACCAGG TCCGAAAGCT GGCCACCCAG CTGCTGGTGG ACCTGGCCGA

Mouse GACCATCAGG TCCGAAAGCT GGCTACTCAG CTGCTGGTGG ACCTGGCAGA

Pig GACCACCAGG TCCGAAAGCT TGCCACCCAG CTGCTGGTGG ACCTGGCCGA

Rabbit GACCACCAGG TCCGGAAGCT GGCCACCCAG CTGCTGGTGG ACCTGGCCGA

Rat GACCATCAGG TTCGAAAGCT GGCTACCCAG CTGCTGGTGG ACCTGGCAGA

Elephant GACCCACAGG TCCGCAAGCT GGCCACCCAG TTGCTGGTGG ACCTGGCGGA

Frog GACCACCAGG TGCGCAAACT CGCAACACAG CTCCTTGTGG ACCTGGCTGA

Zebrafish GACCCGTCAG TCAGGAAGCA GGCCACACAG CTGCTGGTGG ATCTGGCCGA

Chicken GACCATCAAG TTCGAAAACT TGCCACTCAG CTACTTGTAG ACCTTGCTGA

Fugu GATTTGGCTG TCAGAAAACA GGCTACTCAA CTACTTGTGG ATCTGGCTGA

Zebra_Finch GACCATCAGG TCCGAAAATT GGCCACTCAG CTGCTGGTGG ACCTGGCTGA

Human GGGCTGCCAC ACACACCACT TCAACAGCCT GCTGGACATC ATCGAGAAGG

Gorilla GGGCTGCCAC ACACACCACT TCAACAGCCT GCTGGACATC ATCGAGAAGG

Chimpanzee GGGCTGCCAC ACACACCACT TCAACAGCCT GCTGGACATC ATCGAGAAGG

Orangutan GGGCTGCCAC ACACACCACT TCAACAGCCT GCTGGACATC ATCGAGAAGG

Marmoset GGGCTGCCAC ACACACCACT TCAACAGCCT GCTGGACATC ATTGAGAAGG

Cow AGGCTGCCAC ACCCACCACT TCAACAGCCT GCTGGACATT GTTGAGAAGG

Dog AGGCTGCCAC ACCCACCACT TCAACAGTCT GTTGGACATT GTGGAAAAGG

Guinea_Pig AGGCTGCCAT ACCCACCACT TCAACAGTCT GCTAGACATA ATTGAAAAAG

Horse AGGCTGCCAT ACCCACCACT TCAACAGCCT GTTGGACATC ATCGAGAAAG

Mouse GGGGTGCCAC ACCCACCACT TCAACAGTCT GCTGGACATC ATTGAAAAGG

Pig AGGCTGCCAC ACCCACCACT TCAACAGCCT GCTGGACATC ATTGAGAAGG

Rabbit AGGCTGCCAC ACTCACCACT TCAACAGCCT GCTGGACGTC ATTGAGAAAG

Rat GGGCTGCCAC ACCCACCACT TCAACAGTCT GTTGGACATC ATTGAAAAGG

Elephant AGGCTGCCAC ACCCACCACT TCAATAATCT TCTGGACATC ATTGAGAAAG

Frog ATGCTGTCAC TCCTGCCACT TCAATAGTCT CATGGATATC ATTGAGAAGG

Zebrafish GGGCTGCAGC ACACACCACT TCAGCAGTCT GCTGGACATC ATCGAGAAGG

Chicken AGGCTGCAAC ACACATCACT TCAACAGCTT GCTTGATATC ATAGAAAAGG

Fugu GGGCTGCAGC ACACATCACT TCACCAGCTT GCTCGACATC ATCGAACGAG

Zebra_Finch AGGCTGCAAC ACTCACCACT TCAACAGCCT GCTGGATATC ATAGCCAAGG

Human TGATGGCCCG CTCCCTCTCC CCACCCCCGG AGCTGGAAGA AAGGGATGTG

Gorilla TGATGGCCCG CTCCCTCTCC CCACCCCCGG AGCTGGAAGA AAGGGATGTG

Chimpanzee TGATGGCCCG CTCCCTCTCC CCACCCCCGG AGCTGGAAGA AAGGGATGTG

Orangutan TGGCGGCCCG CTCCCTCTCC CCACCCCCAG AGCTGGAAGA AAGGGATGCG

Marmoset TGATGGCCCG CTCCCTCTCC CCACCCCCGG AGCTGGAAGA GAGGGATGTG

Cow TGATCGCCCG TTCCCTCTCT CCGCCCCCCG AGCTGGAGGA GAGGGATGTG

Dog TGATTGCCCG CTCTCTCTCT CCACCCCCTG AGCTGGAGGA AAGAGACGTG

Guinea_Pig TGATGGCCCG CTCTCTCTCT CTGCCTCCTG AGCTGGAAGA AAGGGATGTG

Horse TGATTGCCCG CTCCCTCTCC CCGCCCCCCG AGCTGGAGGA AAGAGACCTG

Mouse TGATGGCGCG CTCACTCTCT CCACCCCCGG AGCTGGAAGA AAGGGACCTG

Pig TGATTGCCCG CTCCCTCTCG CCACCCCCCG AGCTGGAGGA GAGGGACGTG

Rabbit TGACGGCCCG CTCCCTCTCT CCACCTCCTG AGCTGGACGA GAGGGGCGCG

Rat TGATGGCTCG CTCCCTTTCT CCACCCCTGG AGCTGGAGGA AAGGGATCTG

Elephant TGATTGCTCG CTCCCTGTTC CCTCCTCCTG AGCTAGAAGA AAGAGATGTG

Frog TGGTGAACCG TTCCTTGTCC ACACCAGAGT CAGGTCATGA T---------

Zebrafish TGGCCAGTCG TCCTCTGAGC TGCTCT---- --GTAGAGGG CGACAGAGAA

Chicken TGGCTGCGCA TTCTCTCTCA TCTCCATCTG AACTGGAAGA GAGGGATTTG

Fugu TGGCCAGTCG CTCTCTAGTG TGTTCGGGAT CTGAGATGCC TGAGCGAGAC

Zebra_Finch TGGCTGCACA TTCTCTGTCA TCTCCTTCTG AACTGGAGGA GAGGGACTTG

Human GCCGCATACT CGGCCTCCTT GGAGGATGTG AAGACAGCCG TCCTGGGGCT

Gorilla GCCGCATACT CGGCCTCCTT GGAGGATGTG AAGACAGCCG TCCTGGGGCT

Chimpanzee GCCGCATACT CGGCCTCCTT GGAGGATGTG AAGACAGCCG TCCTGGGGCT

Orangutan GCCGCACACT CAGCCTCCTT GGAGGATGTG AAGACAGCTG TCCTGGGTCT

Marmoset GCTGCATACT CAGCCTCCCT GGAGGACGTG AAGACAGCAG TCCTGGGGCT

Cow GCCGCGTACT CGGCCTCCTT GGAGGATGTG AAGACTGCGG TCCTGGGGCT

Dog GCGGTGTACT CGGCCTCCTT AGAGGATGTG AAGACTGCTG TCCTGGGGCT

Guinea_Pig ACTGCTTACT CAGCTTCCCT GGAAGATGTG AAGACTGCAG TCCTGGGACT

Horse GAGGCATACT CAGCCTCCTT GGAAGATGTG AAGACCGCGG TCCTGGGGCT

Mouse GCCATGCACT CGGCCTCCCT GGAGGACGTG AAGACCGCGG TCCTGGGGCT

Pig GCGGCGTACT CGGCCTCCTT GGAGGACGTG AAGACAGCGG TCCTGGGGCT

Rabbit GCTGCCTACT CGGCCTCCCT GGAGGACGTG AAGACTGCGG TCCTGGGGCT

Rat GCTGTGTACT CGGCCTCCCT GGAGGATGTG AAGACGGCAG TCCTCGGGCT

Elephant CCTGCGTACT CGGCCTCCTT GGAGGATGTG AAGACTGCGG TCCTGGGGCT

Frog ---------T CTTCTTCTTT GGAAGATGTG AAAACCTCTG TTCTGGGGCT

Zebrafish CTGTGTGTGG AGTCGCCGCT GGAGGACGTG CGCACCGCTA TACTGGGCCT

Chicken CTATCATATT CAGCTTCTTT GGAGGACGTG AAGACAGCAG TTCTTGGACT

Fugu CCCACAGCTG AGTCTGCTAT GGAGGATGTC AGAACTGCAG TTCTGGGCCT

Zebra_Finch CTGTCATATT CAGCTTCTCT GGAGGATGTC AAGACAGCAG TTCTTGGGCT

Human TCTGGTCATC CTTCAGACCA AGCTGTACAC CCTGCCTGCA AGCCACGCCA

Gorilla TCTGGTCATC CTTCAGACCA AGCTGTACAC CCTGCCTGCA AGCCACGCCA

Chimpanzee TCTGGTCATC CTTCAGACCA AGCTGTACAC CCTGCCTGCA AGCCACGCCA

Orangutan TCTGGTCATC CTTCAGACCA AGCTGTACAC CCTGCCTGCA AGCCACGCCA

Marmoset CCTGGTCATC CTTCAGACCA AGCTGTACAC CCTGCCTGCG AGCCACGCCA

Cow CCTGGTCATC CTTCAGACTA AGCTGTATGC CCTGCCTGCC AGCCATGCCA

Dog CCTGGTCATC CTTCAGACCA AGCTGTACGC CTTGCCTGCC AGCCATGCGA

Guinea_Pig CCTAGTCATC CTTCAGACCA AGCTGTACAC CCTGCCTGCC AGCCATGCCA

Horse CCTGGTCATC CTTCAGACCA AGCTGTACAC CTTGCCTGCC AGCCACGCAA

Mouse CCTGGTCATC CTTCAGACCA AGCTCTACAC CTTGCCTGCC AGCCACGCCA

Pig CCTGGTCATC CTGCAGACCA AGCTCTACGC CCTGCCCGCC AGCCACGCCA

Rabbit CCTGGTCATC CTTCAGACCA AGCTGTACAC CCTGCCGGCC AGCCACGCCA

Rat CCTGGTCATC CTTCAGACCA AGCTGTATAC CTTGCCCGCC AGCCATGCCA

Elephant CCTGGTCATC CTTCAGACCA AGCTCTACAC TTTGCCTGCC AGCCATGCAG

Frog GATGGTTATT CTACAGACCA AGCTATACAT TCTGCCAGCC AGCCATGCCA

Zebrafish GCTGGACATC CTGCAGAGCA AACTCTATAG TCTGCCTGCC AGTCATGCCA

Chicken TCTGATAATT CTTCAGACAA AACTCTACAG CTTACCTTCC AGCCATGCCA

Fugu GCTGGAAATC CTGCAGAGCA AACTTTACAG CCTTCCAGCC GTCCACGCCA

Zebra_Finch CCTGATAATT CTTCAGACCA AGCTGTACAG TTTGCCCTCC AGCCACGCCA

Human CGCGTGTGTA TGAGATGCTG GTCAGCCACA TTCAGCTCCA CTACAAGCAC

Gorilla CGCGTGTGTA TGAGATGCTG GTCAGCCACA TTCAGCTCCA CTACAAGCAC

Chimpanzee CGCGTGTGTA TGAGATGCTG GTCAGCCACA TTCAGCTCCA CTACAAGCAC

Orangutan CGCGCGTGTA TGAGATGCTG GTCAGCCACA TTCAGCTCCA CTACAAGCAC

Marmoset CGCGCGTGTA TGAGATGCTG GTCAGCCACA TCCAGCTCCA CTATAAGCAC

Cow CACGCGTGTA CGAGACGCTG GTCAGCCACA TTCAGCTCCA CTACAGGCAT

Dog TGCGCGTGTA CGAGACACTC GTCAGCCACA TCCAGCTCCA CTACAAGCAT

Guinea_Pig TGCGTGTGTA TGAGATGCTT GTTAGCCACA TTCAGCTCCA CTACAAGCAT

Horse TGCGTGTGTA CGAGACGCTC GTCAGCCACA TCCAGCTCCA CTACAAGCAC

Mouse CTCGAGTGTA TGAGAGCCTC ATTAGTCACA TCCAGCTCCA TTACAAGCAC

Pig TGCGCGTGTA TGAGACGCTC GTCAGCCACC TCCAGCTCCA CTACAAGCAC

Rabbit CTCGTGTGTA CGAGACGCTC GTCAGCCACC TGCAGCTGCA CTACAAGCAC

Rat CACGAGTGTA TGAGACACTC ATTAGCCACA TCCAGCTCCA TTACAAGCAC

Elephant CACGGGTATA CGAGATGCTT GTCCACCACA TCCAGCTGCA CTACAAGCAC

Frog TGCGTGTGTA CGAGATGCTG ATTAATCATA TCCAGCTTCA CTACAAATAC

Zebrafish GTCGAGTGTA TGAGCTGCTC ATCTCACACC TGCAGCTGCA CTACAAGAAC

Chicken TGCGGGTGTA TGAGATGCTG ATTCACCACA TCCAGCGCCA CTATATGTAT

Fugu GTCGTGTGTA CGAGCTGCTT ATCAGCCACT TGCAGCTTCA CTACAAGAAC

Zebra_Finch CGTGTGTGTA CGAGATGCTG ATCCAGCACG TCCAGCGCCA CTACAAGTAC

Human AGCTACACCC TGCCAATCGC GAGCAGCATC CGGCTGCAGG CCTTTGACTT

Gorilla AGCTACACCC TGCCAATCGC GAGCAGCATC CGGCTGCAGG CCTTTGACTT

Chimpanzee AGCTACACCC TGCCAATCGC GAGCAGCATC CGGCTGCAGG CCTTTGACTT

Orangutan AGCTACACCC TGCCAATTGC GAGCAGCATC CGGCTGCAGG CCTTTGACTT

Marmoset AAGTACACCC TGCCCATTGC CAGCAGCATC CGGCTACAGG CCTTTGACTT

Cow AGCTACACCC TGCCCATCGC CAGCAGCATC CGTCTGCAGG CCTTCGACTT

Dog AGCTATACCC TGCCCATTGC CAGTAGCATC CGGCTGCAGG CTTTTGACTT

Guinea_Pig AGCTACTCCC TGCCCATTGC CAGCAGCATC CGGCTACAGG CCTTTGACTT

Horse AGCTACACCC TGCCCATTGC CAGCAGCATC CGGCTACAGG CCTTCGATTT

Mouse GGCTACTCCC TGCCCATTGC TAGCAGCATC CGACTACAGG CCTTTGACTT

Pig AGTTACACCC TGCCCATCGC CAGCAGCATC CGGCTGCAGG CCTTCGACTT

Rabbit AACTACACCC TGCCCATCGC CAGCAGCATC CGGCTGCAGG CATTCGACTT

Rat GGCTACTCCT TGCCCATTGC TAGCAGCATC CGGCTGCAGG CCTTTGACTT

Elephant ATGTACACCT TGCCCATTGC CAGCAGCATC CGGCTTCAGG CCTTTGACTT

Frog AATTACAGCT CACCCATTGC TACCAGTATA CGCCTCCAGG TTTTTGACTT

Zebrafish AAATACTGCA GCGCCATCAG CGAGTGTGTG TGTTTTGAGG TGTTTGATTT

Chicken GCGTACAGCC TTGCTGTTGC TAGCAGCATC AGACTGCAGG TGTTTGATTT

Fugu AAGTACAGTT CAGCTATTGC CTCTAGTATT AGGCTACAGG TCTTTGACTT

Zebra_Finch TCCTACAGCC TCCCCGTGGC CAGCAGCATC AGGCTGCAGG TGTTTGATTT

Human CCTGTTGCTG CTGCGGGCCG ACTCACTGCA CCGCCTGGGC CTGCCCAACA

Gorilla CCTGTTGCTG CTGCGGGCCG ACTCACTGCA CCGCCTGGGC CTGCCCAACA

Chimpanzee CCTGTTGCTG CTGCGGGCCG ACTCACTGCA CCGCCTGGGC CTGCCCAACA

Orangutan CCTGTTGCTG CTGCGGGCCG ACTCACTGCA CCGCCTCGGC CTGCCCAACA

Marmoset CCTGTTGCTG CTGCGGGCTG ACTCACTGCA CCGCCTCGGC CTGCCCAACA

Cow CCTGCTGCAG CTGCGGGCCG ACTCACTGCA CCGCCTGGGC CTGCCCAGCA

Dog TCTACTGCTG CTGAGGGCCG ACTCGCTGCA CCGCCTCGGC CTGCCCAGCA

Guinea_Pig CCTGCTGCTG CTGCGGGCAG ATGCGCTGCA CCGCCTGGGT TTGCCCACCA

Horse CCTGCTGCTG CTGCGGGCCG ACTCGTTGCA CCGCCTCGGT CTGCCCAACA

Mouse CCTGCTGCTA CTGCGGGCTG ACTCGCTGCA TCGACTGGGC CTGCCCAACA

Pig CCTGCTGATG CTGCGGGCCG ATTCCTTGCA CCGCCTCGGG CTGCCCAACA

Rabbit CCTGCTGCTG CTGCGCGCCG ACTCCCTGCA CCGCCTGGGC CTGCCCAACA

Rat CCTGCTGCTA CTGCGGGCTG ACTCGCTGCA TCGACTGGGC CTGCCCAACA

Elephant CCTGCTGCTG CTCCGGGCAG ATGCACTACA CCGCCTGGGC CTGCCTAACA

Frog CCTCCTACAC CTCCGAGCTG ACTCTTTGCG GCGTGTTGGG CTACCTAACA

Zebrafish CCTGCTGCTG ATGAGGGCTG ATTCTCTACA TCGTCTCGGG GTCCCCAATA

Chicken CCTCCTGATG CTTCGAGCTG ACTCCCTCCA CCGTCTTGGC CTTTCTAACA

Fugu CTTTCTGATG ATGCGAGCAG ACTCTCTTCA CCGTATCGGA GTTCCAAACA

Zebra_Finch CCTGCTGATG CTCCGAGCTG ACTCCCTGCA CCGCCTCGGC CTTTCCAACA

Human AGGATGGAGT CGTGCGGTTC AGCCCCTACT GCGTCTGCGA CTACATGGAG

Gorilla AGGATGGAGT CGTGCGGTTC AGCCCCTACT GCGTCTGCGA CTACATGGAG

Chimpanzee AGGATGGAGT CGTGCGGTTC AGCCCCTACT GCGTCTGCGA CTACATGGAG

Orangutan AGGATGGAGT CGTGCGGTTC AGCCCCTACT GCGTCTGCGA CTACATGGAG

Marmoset AGGACGGGGT CGTGCGGTTC AGCCCCTACT GCGTCTGTGA CTACATGGAG

Cow AAGACGGGCT TGTGAGGTTC AGCCCCTACT GCGTCTGTGA CTGCCTGGAG

Dog AGGACGGGGC CGTGAGGTTC AGCCCTTACT GCGTCTGCGA TGCCATGGAG

Guinea_Pig AGGATGGGAT TGTGAGATTC AGCCCCTACT GCCTGTGTGA CTGCGTGGAG

Horse AGGACGGGGT TGTGAGGTTC AGCCCCTATT GCCTCTGTGA CTACATGGAG

Mouse AGGATGGGGT CGTGAGATTC AGCCCTTACT GCCTCTGTGA CTGCATGGAA

Pig AGGACGGGGT TGTGAGGTTC AGCCCCTACT GCCTCTGTGA CTACGTGGAG

Rabbit AGGATGGGGT GGTGCGGTTC AGCCCGTACT GCCTCTGCGA CTACATCGAG

Rat AGGATGGGGT CGTGCGATTC AGCCCTTACT GCCTCTGTGA CTGCGCGGAA

Elephant AGGATGGTGC TGTACGGTTC AGCTCCTACT GCCTCTGTGA CCACATGGAG

Frog AAGATGGCGC CTTCAGATTC AGTCCCTATT GTCTTTGTGA ATCTGTAGAA

Zebrafish AAGACGGAGC TCTGAGATTC AGCCCGTACT GCCACTGTGA CCCAGGGGAG

Chicken AGGATGGGGC AGTGAGATTC AGCCCTTACT GTCTGTGTGA TTTTGTAGAA

Fugu AAGACAGAGC CATGAGGTTC AGCCCTTACT GCTACTGTGA CAATGGGGAG

Zebra_Finch AGGATGGAGC AGTGAGGTTC AGCCCCTACT GCCTCTGTGA CTTTGTAGAG

Human CCAGAGAGAG GCTCTGAGAA GAAGACCAGC GGCCCCCTTT CTCCTCCCAC

Gorilla CCAGAGAGAG GCTCTGAGAA GAAG---AGC GGCCCCCTTT CTCCTCCCAC

Chimpanzee CCAGAGAGAG GCTCTGAGAA GAAGACCAGC GGCCCCCTTT CTCCTCCCAC

Orangutan CCAGAGAGAG GCTCTGAGAA GAAGACCAGC GGCCCCCTTT CTCCTCCCTC

Marmoset CTGGAGAGAG GCTCTGAGAA GAAGGCCGGC GGCTCCCTTT CTCCTCCCAC

Cow ACAGAGAGGA GCTCCGAGAA GAAGGCCGGG TGCCCCCTGT CCCCGCCCGC

Dog CCAGAGAGAG GCTCAGAGAA GAAGGCCAGT GGCCCCCTGT CACCTCCCAC

Guinea_Pig CCTGAGAGGG CCTCTGACAA GAAGGCTGGC AGTACCCTTT CACCTCCCAC

Horse CCGGAGAGAG GCTCTGAGAA GAAGGCCAGT GGTCCCTTGT CACCTCCCAC

Mouse CTGGATAGAG CCTCAGAGAA GAAAGCCAGT GGGCCCCTTT CACCTCCAAC

Pig CCGGAGAGAG GCTCTGAGAA GAAGGCCAGT GGTCCCCTGT CACCTCCCAC

Rabbit CCGGAGCGGA GCTCTGAGAA GAAGGCCAGC GGCCCCCTTT CCCCTCCCAC

Rat CTGGACAGGG CCTCAGAGAA GAAAGCCAGC GGACCCCTTT CACCGCCAAC

Elephant CCAGAGCACG GCTTGGAGAA GAAGGCCAGT GGCCCCGTCT CGCCTCCCAC

Frog CAAGAGAGAG CAGCTGAACG CAAGCCAGCA GGCACCTTGT CACCCCCCTC

Zebrafish TCCGAGAGAG TGTCAGAGAA GAAGCCGACA GGAAGTGTGT CTCCACCGGC

Chicken GCAGAGAGAG CTTCTGAGAA AAAGCCTACT GGTACGCTCT CTCCACCTTC

Fugu CCAGAGCGAG GGGGTGAAAA GAAACCCACA GGTTCAACGT CCCCCCCGGT

Zebra_Finch GCAGAGAGGG CTTCTGAGAA AAAGCCCCCT GGCACCCTGT CCCCACCTTC

Human AGGGCCTCCT GGCCCGGCGC CTGCAGGCCC CGCCGTGCGG CTGGGGTCCG

Gorilla AGGGCCTCCT GGCCCGGCGC CTGCAGGCCC CGCCGTGCGG CTGGGGTCCG

Chimpanzee AGGGCCTCCT GGCCCGGCGC CTGCAGGCCC CGCCGTGCGG CTGGGGTCCG

Orangutan AGGGCCGCCT GGCCCGGCGC CTGCAGGTCC CGCCGTGCGG CTGGGTTCCC

Marmoset AGGG------ ---------C CTGCGGGCCC GGCTGTGCGG CTGGGCTCCC

Cow CGGGCCCCCT GGCCCTGCGC CTGCGGGCCC TGCCGTGCGC CTTGGGTCCC

Dog GGGGCCTCCT GGTCCTGCGC CCGCAGGCCC CGCCGTGCGG CTTGGCTCCC

Guinea_Pig TGGGGCACCA GGTTCTGCAT CAGGAAGCTC TGCCTTGCGG CTTGGCCATC

Horse CGGGCCGCCT GGCCCAGCGT CTCCAGGCCC CGCCACACGG CCCGGCTCTC

Mouse TGGGCCCCCT AGCCCTGTGC CTATGGGCCC TGCTGTGCGG CTCGGCTACC

Pig CGGGCCACCT GGCCCAGTGC CCACAGGCCC CACCGTGCGG CTGGGCTCGC

Rabbit CGGGCCGCCT GGCCCTGCAC ACGCAGGCCC CGCTGTGCGG CTTGGCTACC

Rat TGGGCCCCCC AGCCCTGTGC CAACGGGCCC TGCTGTGAGG CTCGGCCACC

Elephant GGGGCCTACC GTCCCAGCAC CTGCAGGCCC CACTGTGCGG CTTGGCTATC

Frog TGGTAGCCCC AGTGTCCCAT CACAGACTGT CACTCCACGC ATGGGATTCT

Zebrafish AGGAAGTCCC ACACCGGCAG CTCCGCCCTC CTCCATCCGC ACCGCTTACC

Chicken AGGAAGTCCC AGTGTGCCTT CCCAGAACAG CACCATTCGA ATAGGGCATT

Fugu TGGCAGCCCC GCTCCTCCAC CGGCTCCCTC GTCTGTTCGT TCGGCCTTTC

Zebra_Finch AGGCAGCCCC AGTGTGCCTT CCCAGGGTGG CACCGTCCGG GTGGGACACC

Human TGCCCTACTC CCTGCTCTTC CGCGTCCTGC TGCAGTGCTT GAAGCAGGAG

Gorilla TGCCCTACTC CCTGCTCTTC CGCGTCCTGC TGCAGTGCTT GAAGCAGGAG

Chimpanzee TGCCCTACTC CCTGCTCTTC CGCGTCCTGC TGCAGTGCTT GAAGCAGGAG

Orangutan TGCCCTACTC CCTGCTCTTC CGTGTCCTGC TGCAGTGCTT GAAGCAGGAG

Marmoset TGCCCTACTC TCTGCTCTTC CGCGTCTTGC TGCAGTGCTT GAAGCAGGAG

Cow TGCCCTACTC CCTGCTCTTC CGTGTTCTGC TGCAGTGTCT GAAGCAGGAG

Dog TGCCCTACTC CCTGCTCTTC CGTGTCCTGC TGCAGTGCCT GAAGCTGGAG

Guinea_Pig TTCCCTACTC CCTGCTTTTT CGAGTTCTGC TACAGTGTTT GAAGCAGGAG

Horse TGCCCTACTC TCTCCTCTTC CGTGTCCTGC TGCAGTGCTT GAAGCAGGAG

Mouse TACCCTACTC CCTGCTCTTC CGTGTCCTGT TGCAGTGTTT GAAGCAGGAG

Pig TGCCCTACTC CCTGCTCTTC CGTGTCCTTC TGCAGTGTTT GAAGCAGGAG

Rabbit TGCCCTACTC CCTGCTCTTC GGCCTCCTCC TGCAGTGCCT GAAGCAGGAG

Rat TGCCCTACTC CCTGCTTTTC CGTGTCCTGC TGCAGTGTTT GAAGCAGGAG

Elephant TGCCCTACTC CTTGCTCTTC CAAGTCCTGC TGCAGTGTCT GAAGCAGGAG

Frog TGCCATTCTC ACTAGCATTT GGGGTTATCC TGCAGTGCCT AAAGCAGGAA

Zebrafish TGCCCTACAG TCTGGCTTTC AGTGTGCTGC TGCAGTGCCT CAAGACGGAG

Chicken TGCCATACTC AATGGTCTTT GGTGTCCTTC TGCAGTGTTT GAAGCAAGAG

Fugu TGCCCTATGC ACCCGCCTTC AACGTCCTAC TGCAGTGCCT CAAAATGGAA

Zebra_Finch TGCCCTATTC CATGGTCTTT GGGGTCCTCC TGCAGTGTTT GAAGCAGGAG

Human TCTGACTGGA AGGTGCTGAA GCTGGTTCTG GGCAGGCTGC CTGAGTCCCT

Gorilla TCTGACTGGA AGGTGCTGAA GCTGGTTCTG GGCAGGCTGC CTGAGTCCCT

Chimpanzee TCTGACTGGA AGGTGCTGAA GCTGGTTCTG GGCAGGCTGC CTGAGTCCCT

Orangutan TCTGACTGGA AGGTGCTGAA GCTGGTTCTG GGCAGGCTGC CCGAGTCCCT

Marmoset ACTGACTGGA AGGTGCTAAA GCTGGTTCTA GGCAGGCTGC CCGAGTCCCT

Cow ACCGACTGGA AGGTGCTGAA GCTGGTGCTC AGCAAACTGC CCGAGTCGCT

Dog GTGGACTGGA AGGTACTGAA GCTCGTGCTT AGCAAGCTGC CAGAGTCCCT

Guinea_Pig ACTGACTGGA AGGTGCTGAA GCTCGTCCTC AGTAAGCTGC CAGAGTCACT

Horse ACCGACTGGA AGGTGCTGAA GCTTGTGCTC AGCAAGCTCC CTGAGTCACT

Mouse AGCGACTGGA AGGTGCTGAA GCTGGTGCTC AGCAGGCTGC CGGAGTCACT

Pig ACGGACTGGA AGGTGCTCCG GCTGGTGCTC AGCAAGCTGC CCGAGTCGCT

Rabbit ACGGACTGGA AGGTGCTGAA GCTGGTGCTC AGCAAGCTGC CCGAGTCTCT

Rat ACCGACTGGA AGGTGCTGAA GCTGGTGCTC AGCAAACTCC CTGAGTCGCT

Elephant ACGGACTGGA AGGTGCTGAA GCTGGTACTC AGCAAGCTGC CGGAGTCCCT

Frog ACTGACTGGA AAGTCCTGAA ACTAGTCCTC AACAAGCTGC CAGAGTGTCT

Zebrafish ACCGACTGGA AGGTCCTGAA GCTGGTTCTG GATAAGATGT CCTGCACCAT

Chicken ACAGACTGGA AGGTGTTGAA GTTGGTCCTC AACAAACTAC CAGAATCACT

Fugu ACAGACTGGA AGGTGCTGAA ACTGGTTCTT GACAAACTGC CCTGGATGCT

Zebra_Finch ACAGACTGGA AGGTGCTGAA GTTGGTTCTC AACAAATTAC CCGAATCTCT

Human GCGCTATAAA GTGCTCATCT TTACTTCCCC TTGCAGTGTG GACCAGCTGT

Gorilla GCGCTATAAA GTGCTCATCT TTACTTCCCC TTGCAGTGTG GACCAGCTGT

Chimpanzee GCGCTATAAA GTGCTCATCT TTACTTCCCC TTGCAGTGTG GACCAGCTGT

Orangutan GCGCTACAAA GTGCTCATCT TTACTTCCCC TTGCAGTGTG GACCAGCTGT

Marmoset GCGCTACAAA GTGCTCATCT TCACCTCCCC ATGCAGTGTG GACCAGCTGT

Cow GCGCTATAAG GTCCTCATCT TCACCTCCCC ATGCAGTGTC GACCAGCTGT

Dog GCGTTATAAG GTGCTCATCT TCACCTCGCC CTGCAGCGTG GACCAGCTGT

Guinea_Pig GCGCTACAAG GTCCTCATCT TCACATCATC ATGCAATGTT GACCAGCTCT

Horse GCGCTATAAG GTCCTCATTT TCAACTCTCC ATGCAGTGTG GACCAGCTGT

Mouse GCGCTACAAA GTCCTCATCT TCACCTCCCC CTGCAGTGTC GACCAGCTGT

Pig CCGCTACAAG GTCCTCACCT TCACGTCGCC CTGCAGCGTG GACCAGCTGT

Rabbit GCGCTACAAG GTGCTCGTCT TCACCTCCCC CTGCAGCCTC GACCAGCTGT

Rat GCGCTACAAA GTCCTCATTT TCACCTCCCC CTGCAGTGTT GACCAGCTGT

Elephant GCGCTACAAG GTCCTCATCT TCACATCCCC CTGCAGCGTC GACCAGCTGT

Frog GCGTTACAAA CTGCTCATTC TGTCATCTCC CTGCAATATA GATCAACTGG

Zebrafish CCAGTATAAA GTGCTGATCC TGACGTCTCC ATGTAACATT GATAATCTGT

Chicken CCGCTATAAA GTGCTGTTTT TAACCTCTCC TTGTAACATA GACCTCCTGG

Fugu TCAGTACAAA GTGCTGCTGC TTACTTCCCC CTGCAGCTTG GACCATCTTT

Zebra_Finch CCGCTATAAA GTGCTATTTT TAACTTCTCC TTGCAACATA GACCAGCTGG

Human GCTCTGCTCT CTGCTCCATG CTTTCAGGCC CAAAGACACT GGAGCGGCTC

Gorilla GCTCTGCTCT CTGCTCCATG CTTTCAGGCC CAAAGACACT GGAGCGGCTC

Chimpanzee GCTCTGCTCT CTGCTCCATG CTTTCAGGCC CAAAGACACT GGAGCGGCTC

Orangutan GCTCTGCTCT CTGCTCCATG CTTTCAGGCC CAAAGACACT GGAGCGGCTC

Marmoset GCTCCGCCCT CTGCTCCATG CTTTCAAGCC CAAAGACAGT GGAGCGGCTC

Cow CCGCCGCCCT CTGCTCCATG CTTTCAGGTC CCAAGACCCT GGAGCGGCTC

Dog CCTCCGCCCT CTGCTCCATG CTCTCAGGTC CCAGAACTCT GGAGCGGCTC

Guinea_Pig CTTCCGCCCT CTGCTCTATG CTTTCAGGCC CAAAGACACT TGAGCGCCTC

Horse CCTCCGCCCT CTGCTCTATG CTTTCAGGTC CCAAGACCCT CGAGCGGCTC

Mouse CTTCTGCCCT CTGCTCCATG CTTTCAGCTC CAAAGACCCT TGAGCGGCTC

Pig CGGCCGCCCT CTGCTCCATG CTTTCAGGCC CCAAGACCCT TGAGCGGCTC

Rabbit CTTCCGCGCT CTGCTCCATG CTTTCAGGCC CAAAGACCCT GGAGCGGCTC

Rat CTTCTGCTCT CTGCTCCATG CTTTCAGCCC CGAAGACCCT TGAGCGGCTC

Elephant CCTCTGCCCT CTGCTCCATG CTTTCAGGTC CCAAGACCCT GGAACGACTC

Frog CTGGCTCTCT CTGTGCTATG CTTACAGACA AGACTGCAAG TGATCGCCTT

Zebrafish GCGCAACGCT CTGCTCGATG GTGACGGACC GCATTATTTT GGAGCGTCTG

Chicken CATCTGCACT GAGCTACATG CTCACAGACA AGAAGACAAC CGACAGGCTC

Fugu GCTCCACGCT CTGCTGCATG GTGACGGATC GGCTGATATC GGAGCGCTTG

Zebra_Finch CCTCTGCACT GACTTGTACT CTAAGCCTCC CCAAGACCAC GGACAGGCTC

Human CGAGGCGCCC CAGAAGGCTT CTCCAGAACT GACTTGCACC TGGCCGTGGT

Gorilla CGAGGTGCCC CAGAAGGCTT CTCCAGAACT GACTTGCACC TGGCCGTGGT

Chimpanzee CGAGGCGCCC CAGAAGGCTT CTCCAGAACT GACTTGCACC TGGCCGTGGT

Orangutan CGAGGCGCCC CAGAAGGCTT CTCCAGAACT GACCTGCACC TGGCTGTGGT

Marmoset CGAGGCGCCC CGGAAGGCTT CTCCAGAACT GACCTGCACC TGGCCGTGGT

Cow CGAGGCACTC CAGAAGGCTT CTCCAGAACC GACCTGCATC TCGCTGTGGT

Dog CGAGGCACCC CGGAAGGGTT CTCCAGAACC GACCTGCACC TGGCTGTGGT

Guinea_Pig CGAGGCACCC CAGAAGGATT CTCCAGAACT GACCTGCACT TGGCTGTGGT

Horse CGAGGCACCC CAGAAGGGTT CTCTAGAACC GACCTGCATT TGGCTGTGGT

Mouse CGAGGTACCC CAGAAGGCTT CTCGAGAACT GACCTGCACT TGGCTGTGGT

Pig CGAGGCACCC CGGAAGGGTT TTCCAGAACC GACCTGCATC TCGCTGTGGT

Rabbit CGGGGCACTC CAGAAGGCTT CTGCAGGACC GACCTGCACC TGGCTGTGGT

Rat CGAGGCACCC CAGAAGGCTT CTCCAGAACT GACCTGCACT TGGCTGTGGT

Elephant CGAGGCACAC CAGAGGGCTT CTCTAGAACC GACTTACACT TGGCAGTGGT

Frog TACATTACTC CCGATGGCTT CTCTCGTACT GACCTTCAGC TGGCAGTGGT

Zebrafish AAGAAAACCC CTGATGGATT TTCCCTCACT GACGTTCAGC TCGCTGTGGT

Chicken CATGGTACCC CAGAAGGCTT TTCCCGCACC GATTTGCACC TGGCTGTAGT

Fugu AAGAAGACCC CCGAGGGATT TTCCCGCACT GATGTTCAGT TGGCGGTGGT

Zebra_Finch CACGGGACCC CCGAAGGCTT TTCCCGCACG GATTTGCACC TGGCTGTGGT

Human TCCAGTGCTG ACAGCATTAA TCTCTTACCA TAACTACCTG GACAAAACCA

Gorilla TCCAGTGCTG ACAGCATTAA TCTCTTACCA TAACTACCTG GACAAAACCA

Chimpanzee TCCAGTGCTG ACAGCATTAA TCTCTTACCA TAACTACCTG GACAAAACCA

Orangutan TCCAGTGCTG ACAGCATTAA TCTCTTACCA TAACTACCTG GACAAAACCA

Marmoset TCCAGTGCTG ACAGCGTTAA TCTCTTACCA TAACTACCTG GACAAAACCA

Cow TCCCGTGCTG ACAGCCCTGA TCTCTTACCA TAACTACTTG GACAAAACTA

Dog CCCTGTGCTG ACGGCGCTCA TCTCTTACCA TAACTACCTG GACAAAACCA

Guinea_Pig TCCTGTTCTG ACGGTGTTAA TCTCTTACCA TAACTACTTG GACAAGAGCA

Horse TCCCGTGCTG ACGGCGCTAA TCTCTTACCA CAACTACCTG GATAAAACCA

Mouse TCCCGTGCTA ACAGCATTAA TATCTTATCA CAACTATTTG GACAAGACCA

Pig TCCCGTGCTG ACGGCATTAA TCTCGTACCA TAACTACTTG GACAAAACCA

Rabbit CCCAGTGCTC ACGGCGTTGA TCTCCTACCA TAACTACCTG GACAAGAGCA

Rat CCCTGTACTG ACAGCGTTAA TATCTTACCA CAACTATTTG GATAAGACTA

Elephant TCCAGTGTTG ACAGCACTGA TCTCTTACCA TAACTACCTG GACAAAACCA

Frog TCCTGTACTG ACAGCCTTAA CCTCATACCA CCAACACCTA GAGCTCTCTA

Zebrafish TCCTGTTCTC ACGGCCATCA CGTCCTAC-- ---------- ----------

Chicken TCCAGTACTG ACAGCGTTGA TATCTTATCA TAATTATCTG GACAAAGCTA

Fugu TCCTGTCCTC ACAGCAATCA CCTCCTACCA TAATTACCTG GAGCAGTCCA

Zebra_Finch CCCTGTTCTG ACAGCACTGA TATCTTACCA CAACTACCTG GACAAAGCCA

Human AACAGCGCGA GATGGTCTAC TGCCTGGAGC AGGGCCTCAT CCACCGCTGT

Gorilla AACAGCGCGA GATGGTCTAC TGCCTGGAGC AGGGCCTCAT CCACCGCTGT

Chimpanzee AACAGCGCGA GATGGTCTAC TGCCTGGAGC AGGGCCTCAT CCACCGCTGT

Orangutan AACAGCGCGA GATGGTCTAC TGCCTGGAGC AGGGCCTCAT CCACCGCTGT

Marmoset AGCAGCGCGA GATGGTCTAC TGCCTGGAGC AGGGCCTCGT CCACCGCTGT

Cow GACAGCGGGA GATGGTGTAC TGCCTGGAGC AAGGCCTCAT CTACCGCTGT

Dog GACAGCGGGA GATGGTGTAC TGCCTGGAGC AAGGCCTGAT CTACCGCTGC

Guinea_Pig AGCAGCGTGA AATGGTTTAC TGCCTGGAGC AAGGCCTCAT CTACCGCTGT

Horse GACAGCGGGA GATGGTGTAC TGCCTGGAGC AAGGCCTCAT CTACCGCTGC

Mouse GACAGCGTGA GATGGTGTAC TGCCTGGAAC AAGGCCTTAT CTACCGCTGC

Pig GACAGCGGGA GATGGTGTAC TGCCTGGAGC AAGGCCTCAT CTACCGCTGC

Rabbit AGCAGCGGGA GATGGTGTAC TGCCTGGAGC AAGGGCTGAT CCACCGCTGT

Rat GACAGCGTGA GATGGTTTAC TGCCTAGAAC AAGGCCTTAT CTACCGCTGT

Elephant AACAGCGCGA GATGGTGTAC TGCTTGGAGC AAGGCCTCAT CTACCGCTGT

Frog AACAGAGGGA AATGGTAAGG TGTCTGGAAA CAGGCCTGAT CTTTCGCTGC

Zebrafish ---------- ---------- ---------- ---------- ----------

Chicken AACAGCGTGA AATAGTGTAC TGCCTGGAGC ATGGCCTTAT TTATCGCTGT

Fugu GACAGAGGGA GCTGGTTCAG TGCCTTGAGA CTGGTCTCAT TCACCGCTGT

Zebra_Finch AGCAGCGAGA GATCGTGTAC TGCCTGGAGC ACGGGCTCAT CTACCGCTGT

Human GCCAGCCAGT GCGTCGTGGC CTTGTCCATC TGCAGCGTGG AGATGCCTGA

Gorilla GCCAGCCAGT GCGTCGTGGC CTTGTCCATC TGCAGCGTGG AGATGCCTGA

Chimpanzee GCCAGCCAGT GCGTCGTGGC CTTGTCCATC TGCAGCGTGG AGATGCCTGA

Orangutan GCCAGCCAGT GCGTCGTGGC CTTGTCCATC TGCAGCGTGG AGATGCCTGA

Marmoset GCCAGCCAGT GCGTGGTGGC CCTGTCCATC TGCAGCGTGG AGATGCCTGA

Cow GCCAGCCAGT GTGTGGTGGC CCTGGCCGTG TGCAGCGTGG AGATGCCCGA

Dog GCCAGCCAGT GTGTGGTGGC CCTGGCCATC TGCAGCGTGG AGATGCCTGA

Guinea_Pig GCCAGTCAGT GTGTGGTGGC CCTGGCCATC TGCAGTGTGG AGATGCCTGA

Horse GCCAGCCAGT GTGTGGTGGC CCTGGCCATC TGCAGCGTGG AGATGCCAGA

Mouse GCCAGCCAGT GCGTGGTAGC CTTGGCCATC TGCAGTGTGG AGATGCCCGA

Pig GCCAGCCAGT GTGTGGTGGC GCTGGCCGTC TGCAGCGTGG AGATGCCGGA

Rabbit GCCGGCCAGT GCGTGGTGGC CCTGGCCGTG TGCAGCGTGG AGATGCCCGA

Rat GCCAGCCAGT GTGTGGTAGC CTTGGCCATC TGCAGTGTGG AGATGCCCGA

Elephant GCCAGCCAAT GTGTGGTGGC CCTGGCTGTG TGCAGTGTGG AGATGCCTGA

Frog GGCAAGCAGT GCGTTGTGGC CCTTTCCATG TGTGTGATCG AGATGCCAAG

Zebrafish ---------- ---------- ---------- ---------- ----------

Chicken GCAAACCAGT GCGTTGTGGC ACTCTCTGTC TGCAGCGTTG AGATGCCCGA

Fugu GCCAAGCAGT GTGTGGTGGC CCTAACAATG TGCACAGTGG AGATGCCCGA

Zebra_Finch GCCAACCAGT GCGTGGTGGC ACTGTCAGTC TGCAGCGTGG AGATGCCCGA

Human CATCATCATC AAGGCGCTGC CTGTTCTGGT GGTGAAGCTC ACGCACATCT

Gorilla CATCATCATC AAGGCGCTGC CTGTCCTGGT GGTGAAGCTC ACGCATATCT

Chimpanzee CATCATCATC AAGGCGCTGC CTGTTCTGGT GGTGAAGCTC ACGCACATCT

Orangutan CATCATCATC AAGGCGCTGC CCGTCCTGGT GGTGAAGCTC ACACACATCT

Marmoset CATCATCATC AAGGCGCTGC CTGTTCTGGT GGTGAAGCTC ACGCACATTT

Cow CGTCATCCTC AAGGCACTGC CCGTCCTGGT TGTGAAGCTC ACACACATCT

Dog CATCATCATC AAGGCGCTTC CCGTCCTGGT CGTGAAGCTC ACACACATCT

Guinea_Pig TATTATGATC AAGGCTCTGC CCGTCCTGGT GGTGAAGCTC ACACACATCT

Horse CATCATCATC AAAGCATTGC CTGTCTTGGT CGTGAAACTC ACGCACATCT

Mouse CATCATCATC AAGGCGCTTC CTGTCCTGGT GGTGAAGCTC ACACACATTT

Pig TGTCATCATC AAGGCGCTGC CTGTCCTGGT GGTGAAGCTC ACGCACATCT

Rabbit CATCATCATC AAGGCCCTGC CCGTCCTGGT GGTGAAGCTC ACGCACATCT

Rat CATCATCATC AAGGCGCTCC CTGTCCTGGT GGTGAAGCTG ACACACATCT

Elephant CGTCATCATC AAGGCACTGC CCGTACTGAT TGTAAAGCTC ACGCACATCT

Frog CATTCTGATA AAATCCCTCC CGGTCCTTAT CGTGAAGCTA ACCCATATCT

Zebrafish ---------- ---------- ---------- ---------- ----------

Chicken CATCATCATA AAGGCACTTC CAGTCTTAAT AGTCAAATTA ACCCACATAT

Fugu TATCATGATC AAGCTCCTCC CGTCCCTAAT GGTCAAGCTC ACCCACATCT

Zebra_Finch CATCATCATC AAAGCACTGC CCGTCCTGAT TGTCAAACTC ACCCACATTT

Human CAGCCACAGC CAGCATGGCC GTCCCACTGC TGGAGTTCCT GTCCACTCTG

Gorilla CGGCCACAGC CAGCATGGCC GTCCCACTGC TGGAGTTCCT GTCCACTCTG

Chimpanzee CGGCCACAGC CAGCATGGCC GTCCCACTGC TGGAGTTCCT GTCCACTCTG

Orangutan CGGCCACAGC CAGCATGGCC GTCCCACTGC TGGAGTTCCT GTCCACTCTG

Marmoset CGGCCACAGC CAGCATGGCC GTCCCGCTGC TGGAGTTCCT GTCCACCCTG

Cow CGGCCACGGC CAGCATGGCC ATCCCGCTCC TCGAGTTCCT GTCCACTCTG

Dog CGGCCACGGC CAGTATGGCC ATTCCACTCC TCGAGTTCCT GTCAACGCTG

Guinea_Pig CTGCCACGGC CAGCATGGCT GTCCCGCTTC TAGAGTTTCT GTCCACTCTG

Horse CGGCCACAGC CAGCATGGCC ATTCCGCTCC TCGAGTTCCT GTCAACTCTG

Mouse CTGCCACAGC CAGCATGGCC ATCCCGCTCC TGGAGTTCCT GTCTACTTTG

Pig CGGCCACGGC CAGCATGGCC GTCCCGCTCC TCGAGTTCCT GTCCACTCTG

Rabbit CCGCCACAGC CAGCATGGCC GTCCCGCTGC TGGAGTTCCT GTCCACTCTG

Rat CTGCTACAGC TAGCATGGCC ATCCCCCTCC TGGAGTTCCT GTCTACCTTG

Elephant CGGCCACAGC CAGCATGGCC ATCCCGCTGC TCGAGTTCCT GTCGACTCTG

Frog CGGCCACTGT CACCATGGCC ATACCAATGC TGGAGTTTCT TTCAACTTTG

Zebrafish ---------- ---------- ---------- ---------- ----------

Chicken CTGCTACAGC CAACATGGCA ATCCCTCTCT TAGAATTTCT TTCAACTCTA

Fugu CTGCAACCGT TGCCATGGCG TCTCCCATGC TCGAGTTCCT CTCCACTCTG

Zebra_Finch CTGCCACAGC CAACATGGCA ATCCCTCTGC TGGAATTTCT GTCCACTCTG

Human GCCAGGCTGC CGCACCTCTA CAGGAACTTT GCCGCGGAGC AGTATGCCAG

Gorilla GCCAGGCTGC CGCACCTCTA CAGGAACTTT GCCGCGGAGC AGTATGCCAG

Chimpanzee GCCAGGCTGC CGCACCTCTA CAGGAACTTT GCCGCGGAGC AGTATGCCAG

Orangutan GCCAGGCTGC CGCACCTCTA CAGGAACTTT GCCGCGGAGC AGTATGCCAG

Marmoset GCCAGGTTGC CTCACCTCTA CAGGAACTTC GCCGCGGAAC AGTACGCCAG

Cow GCCAGACTGC CTCACCTCTA CAGGAACTTC GCAGCAGAGC AGTATGCCAG

Dog GCCAGGTTGC CCCACCTGTA CAGGAACTTC GCAGCAGAGC AGTACGCGAG

Guinea_Pig GCCAGGCTGC CACATCTCTA CAGGAACTTT GCCGCTGAAC AGTATGCCAG

Horse GCCAGGCTGC CTCACCTCTA CAGGAACTTC GCCGCAGAGC AGTACGCGAG

Mouse GCCAGGCTGC CCCACCTCTA CAGGAACTTT GCTGCAGAGC AGTATGCCAG

Pig GCCAGGCTGC CTCACCTCTA CCGGAACTTT GCGGCGGAGC AGTATGCCAG

Rabbit GCCCGGCTGC CGCACCTCTA CAGGAACTTC GCCGCAGAGC AGTACGCCAG

Rat GCCAGGCTGC CTCACCTCTA CAGGAACTTC GCCGCAGAGC AGTATGCAAG

Elephant GCCCGGCTGC CGCATCTCTA CAGGAACTTT GCTGCTGAGC AGTACGCCAG

Frog GTCCGCCTGC CCCACTTATA TGCAAACTTT GCCGCAGAGC AGTATGCCAG

Zebrafish ---------- ---------- ---------- ---------- ----------

Chicken GCAAGGCTGC CTCACCTCTA CAGGAACTTT GCTGCAGAGC AGTATGCCAG

Fugu GTGCGCCTGC CTCATCTCTA TGCCAACTTT GTAGCAGAGC AGTATGTCAG

Zebra_Finch GCCAGGCTGC CTCACCTGTA CCGGAACTTC GCGGCAGAGC AGTACGCCAG

Human TGTGTTCGCC ATCTCCCTGC CGTACACCAA CCCCTCCAAG TTTAATCAGT

Gorilla TGTGTTCGCC ATATCCCTGC CGTACACCAA CCCCTCCAAG TTTAATCAGT

Chimpanzee TGTGTTCGCC ATCTCCCTGC CGTACACCAA CCCCTCCAAG TTTAATCAGT

Orangutan TGTGTTCGCC ATCTCCCTGC CGTACACCAA CCCCTCCAAG TTTAATCAGT

Marmoset CGTGTTTGCC ATCTCCCTGC CGTACACCAA CCCCTCCAAG TTCAATCAGT

Cow TGTGTTTGCC ATCTCCCTGC CGTATACCAA CCCCTCCAAG TTCAATCAGT

Dog TGTGTTTGCC ATCTCCCTGC CCTACACCAA TCCCTCCAAG TTCAATCAGT

Guinea_Pig TGTGTTTGCT ATCTCCCTGC CGTATACCAA CCCTTCCAAG TTCAATCAGT

Horse CGTGTTCGCC ATCTCCCTGC CGTATACCAA CCCCTCTAAG TTCAACCAGT

Mouse CGTGTTTGCC ATCTCCTTGC CATACACCAA CCCCTCCAAG TTCAACCAGT

Pig CGTGTTCGCC ATCTCCCTGC CGTACACCAA CCCCTCCAAG TTCAATCAGT

Rabbit CGTGTTCGCC ATCTCGCTGC CCTACACCAA CCCCTCCAAG TTCAACCAGT

Rat TGTGTTTGCC ATCTCCTTGC CCTACACCAA CCCCTCCAAG TTCAACCAGT

Elephant CGTTTTCGCC ATTTCACTGC CCTACACCAA CCCATCCAAA TTCAATCAGT

Frog TGTGTTTGCC ATCTCCCTTC CCTACACCAA CCCATCAAAG TTTAATCAAT

Zebrafish ---------- ---------- ---------- ---------- ----------

Chicken TGTGTTTGCC ATCTCCCTGC CATACACAAA CCCTTCCAAG TTTAACCAGT

Fugu CGTGTTTGCC ATCTCTCTCC CCTACACTAA CCCTTCCAAG TTCAACCAGT

Zebra_Finch CGTCTTCGCC ATCTCCCTGC CCTACACAAA CCCCTCCAAG TTTAACCAGT

Human ACATCGTGTG TCTGGCCCAT CACGTCATAG CCATGTGGTT CATCAGGTGC

Gorilla ACATCGTGTG TCTGGCCCAT CACGTCATAG CCATGTGGTT CATCAGGTGC

Chimpanzee ACATCGTGTG TCTGGCCCAT CACGTCATAG CCATGTGGTT CATCAGGTGC

Orangutan ACATCGTGTG TCTGGCCCAT CACGTCATAG CCATGTGGTT CATCAGGTGC

Marmoset ATATCGTGTG CCTGGCCCAT CATGTCATAG CCATGTGGTT CATCAGGTGC

Cow ACATCGTGTG CCTGGCCCAT CACGTCATAG CCATGTGGTT CATCAGGTGC

Dog ACATCGTGTG CCTGGCCCAT CATGTCATAG CCATGTGGTT CATTAGATGC

Guinea_Pig ACATCGTGTG CCTGGCACAT CATGTCATTG CCATGTGGTT CATCAGGTGC

Horse ACATTGTGTG CCTGGCCCAT CATGTCATAG CCATGTGGTT CATTAGGTGC

Mouse ACATTGTGTG TCTGGCTCAC CACGTCATAG CCATGTGGTT CATCAGGTGC

Pig ACATTGTGTG CCTGGCCCAT CACGTCATAG CCATGTGGTT CATTAGGTGC

Rabbit ACATCGTGTG CCTGGCCCAC CACGTGATCG CCATGTGGTT CATCAGGTGT

Rat ACATTGTGTG TCTGGCCCAT CACGTCATAG CCATGTGGTT CATTAGGTGC

Elephant ATATTGTGTG CCTGGCCCAC CACGTGATAG CCATGTGGTT CATTCGGTGC

Frog ACATTGTATT CCTTGCACAC CATGTCATTG CCATGTGGTT CATCCGGTGC

Zebrafish ---------- ---------- ---------- ---------- ----------

Chicken ACATTGTTTG CCTGGCCCAC CACGTGATAG CTATGTGGTT CATTCGGTGT

Fugu ATATCGTATC GCTGGCCCAC CATGTTATTG CCATGTGGTT CATACGCTGC

Zebra_Finch ACATCGTGTG CCTGGCCCAC CACGTCATTG CCATGTGGTT CATCCGCTGC

Human CGCCTGCCCT TCCGGAAGGA TTTTGTCCCT TTCATCACTA AGGGCCTGCG

Gorilla CGCCTGCCCT TCCGGAAGGA TTTCGTCCCT TTCATCACTA AGGGCCTGCG

Chimpanzee CGCCTGCCCT TCCGGAAGGA TTTCGTCCCT TTCATCACTA AGGGCCTGCG

Orangutan CGCCTGCCCT TCCGGAAGGA TTTCGTCCCT TTCATCACTA AGGGCCTGCG

Marmoset CGCCTGCCCT TCCGGAAGGA TTTCGTCCCA TTCATCACTA AGGGCCTGCG

Cow CGCCTGCCCT TCCGGAAGGA CTTTGTTCCT TACATCACCA AGGGTCTGCG

Dog CGCCTGCCCT TCCGGAAGGA TTTTGTTCCT TATATCACTA AGGGCCTGCG

Guinea_Pig CGCCTACCCT TCCGGAAAGA TTTTGTCCCA TTTATCACTA AGGGTTTGCG

Horse CGCCTGCCCT TCCGGAAAGA TTTTGTTCCT TACATCACTA AGGGCCTGCG

Mouse CGACTGCCCT TTCGGAAGGA TTTCGTCCCT TATATCACTA AGGGTTTGCG

Pig CGCCTGCCCT TCCGGAAAGA TTTTGTTCCT TACATCACGA AGGGCCTGCG

Rabbit CGCCTGCCCT TCCGCAAGGA CTTCGTCCCC TACATCACCA AGGGCCTGCG

Rat CGACTGCCCT TTCGGAAGGA TTTTGTCCCT TATATCACTA AGGGTCTGCG

Elephant CGCCTGCCCT TCCGGAAAGA TTTTGTTCCG TATATCACTA AGGGCCTTCG

Frog AGACTATCCC TACGGAGAGA CTTTGTGCCA TATATAACAA AGGGTTTGCG

Zebrafish ---------- ---------- ---------- ---------- ----------

Chicken CGTCTTCCCT TCCGAAAGGA CTTTGTTCCC TATATTACAA AGGGTTTGCG

Fugu AGACTTCCCT TCCGCAAGGA CTTTGTTCAA TACATCACAA AGGGTTTGCG

Zebra_Finch CGCCTGCCCT TCCGCAAGGA CTTCGTCCCC TACATCACCA AGGGCCTGCG

Human GTCCAATGTC CTCTTGTCTT TTGATGACAC CCCCGAGAAG GACAGCTTCA

Gorilla GTCCAATGTT CTCTTGTCTT TTGATGACAC CCCCGAGAAG GACAGCTTCA

Chimpanzee GTCCAATGTC CTCTTGTCTT TTGATGACAC CCCCGAGAAG GACAGCTTCA

Orangutan GTCCAATGTC CTCTTGTCTT TTGATGACAC CCCCGAGAAG GACAGCTTCA

Marmoset CTCCAATGTT CTCTTGTCTT TTGATGACAC CCCTGAGAAG GACAGCTTCA

Cow CTCCAACGTC CTCCTGTCTT TCGATGACAC CCCCGAGAAG GACAGCTTCC

Dog CTCCAACGTC CTCCTGTCTT TTGATGACAC CCCCGAGAAG GACAGTTTCA

Guinea_Pig CTCCAATGTC CTGCTGTCTT TTGATGATAC CCCTGAGAAG GACAGCTTCA

Horse CTCCAATGTC CTCCTGTCTT TTGATGACAC CCCTGAGAAG GACAGCTTCA

Mouse TTCCAATGTC CTCCTGTCTT TTGATGATAC CCCTGAGAAG GACAGTTTCA

Pig CTCCAACGTT CTCCTGTCTT TTGATGACAC CCCCGAGAAG GACAGCTTTA

Rabbit CTCCAATGTG CTGATGTCTT TTGATGACAC CCCTGAGAAG GACAGCTTCA

Rat TTCCAATGTC CTCCTGTCCT TTGATGATAC CCCTGAGAAG GACAGTTTCA

Elephant CTCCAATGTC CTCCTGTCTT TTGATGACAC CCCCGAGAAG GACAGCTTCA

Frog ATCCAATGTC CTGCTGAGCT TTGATGACAC ACCGGAGAAG GGCAGCTTTA

Zebrafish ---------- ---------- ---------- ---------- ---AGTTTCA

Chicken CTCCAATGTG CTGCTCTCCT TTGATGACAC ACCTGAGAAG GACAGTTTCA

Fugu CTCCAACGCC CTGCTGCCAT TCGATGACAG CCACGAGCAA AGTTCTTTTC

Zebra_Finch CTCCAACGTG CTCCTGTCCT TCGATGACAC CCCAGAGAAG GACAGCTTCA

Human GGGCCCGGAG TACTAGTCTC AACGAGAGAC CCAAGAGTCT GAGGATAGCC

Gorilla GGGCCCGGAG TACTAGTCTC AACGAGAGAC CCAAGAGTCT GAGGATAGCC

Chimpanzee GGGCCCGGAG TACTAGTCTC AACGAGAGAC CCAAGAGTCT GAGGATAGCC

Orangutan GGGCCCGGAG TACTAGTCTC AACGAGAGAC CCAAGAGTCT GAGGATAGCC

Marmoset GAGCACGGAG TACTAGTCTC AACGAGAGGC CCAAGAGTCT GAGGATAGCC

Cow GAGCGCGGAG CACCAGTCTC AACGAGAGGC CCAAGAGTTT GAGGATAGCC

Dog GAGCACGAAG CACCAGTCTC AACGAGAGGC CCAAGAGTTT GAGGATAGCC

Guinea_Pig GAGCACGGAG CACCAGCCTC AATGAGAGGC CTAAAAGTTT GAGGATAGCC

Horse GAGCACGGAG CACCAGTCTC AACGAGAGGC CGAAGAGTTT GAGGATAGCC

Mouse GAGCACGGAG CACCAGTCTT AATGAGAGAC CCAAA----- ----------

Pig GAGCTCGGAG TACCAGTCTC AACGAGAGGC CCAAGAGTTT GAGGATAGCC

Rabbit GAGCGCGGAG CACCAGCCTC AACGAGCGGC CCAAA----- ----------

Rat GAGCACGGAG CACCAGTCTT AATGAGAGAC CCAAAAGTTT GAGGATAGCC

Elephant GGGCACGGAG CACCAGTCTC AACGAGAGGC CCAAAAGTTT AAAGATAGCC

Frog GAGCTCGCAG CACCAGCTTG AACGAGAGAC CCAAAAGCTT GAAGCTATCT

Zebrafish GAGCACGCAG CACCAGCCTG AACGAGAGGC CCAAGAGTTT GCGGACGACC

Chicken GGGCTCGTAG TACAAGCCTC AATGAGAGGC CAAAAAGTTT AAGACTAGCC

Fugu GGGCGAGGAG CACGAGCCTC AACGAAAGAC CTAAGAGTCT GCGGGCAGCA

Zebra_Finch GGGCTCGCAG CACCAGCCTG AATGAGAGGC CAAAGAGTTT AAGACTAGCC

Human AGACCCCCCA AACAAGGCTT GAATAACTCT CCACCCGTGA AAGAATTCAA

Gorilla AGACCCCCCA AACAAGGCTT GAATAACTCT CCACCCGTGA AAGAATTCAA

Chimpanzee AGACCCCCCA AACAAGGCTT GAATAACTCT CCACCCGTGA AAGAATTCAA

Orangutan AGACCCCCCA AACAAGGCTT GAATAACTCT CCACCCGTGA AAGAATTCAA

Marmoset AGACCCCCCA AACAAGGCTT GAATAACTCT CCACCCGTGA AAGAATTCAA

Cow AGACCCCCCA AACAAGGCTT GAATAACTCT CCACCCGTGA AAGAACTCAA

Dog AGACCCCCCA AACAAGGCTT GAATAACTCT CCACCCGTGA AAGAATTCAA

Guinea_Pig AGAGCCCCCA AACAAGGCCT GAATAACTCC CCACCTGTGA AAGAATTCAA

Horse AGACCCCCCA AACAAGGCTT GAATAACTCT CCACCTGTGA AAGAATTCAA

Mouse ---------- ---------- ---------- ---------- ----------

Pig AGGCCCCCCA AACAAGGCTT GAATAACTCC CCACCCGTGA AAGAATTCAA

Rabbit ---------- ---------- ---------- ---------- ----------

Rat AGAGCCCCCA AACAAGGCCT GAATAACTCT CCACCTGTGA AAGAATTCAA

Elephant AGGCCCCCCA AACAA----- -AGTAATTCT CCACTCGTGA AAGAATGCAA

Frog AAACACACAA AGCCTGGCTT CAGTGACACG TCCCCAGTTA AGGAGATGAG

Zebrafish AAAGTGGCGA AGCAAGGCCC CAGCGCAAAC TCTCCCGTTA AAGACCTGAA

Chicken AAAAATGCAA AGCAAGGCTT GAATAACTCT CCTCCAGTGA AAGAGCTGAA

Fugu AAGGTGGTGA AGGCAACAGC AGCTGTAACC TCACCAGTTA AAGAGCTGAG

Zebra_Finch AAAAATGCAA AGCAAGGCTT GAATAACTCT CCTCCAGTGA AAGAGCTGAA

Human GGAGAGCTCT GCAGCCGAGG CCTTCCGGTG CCGCAGCATC AGTGTGTCTG

Gorilla GGAGAGCTCT GCAGCCGAGG CCTTCCGGTG CCGCAGCATC AGTGTGTCTG

Chimpanzee GGAGAGCTCT GCAGCCGAGG CCTTCCGGTG CCGCAGCATC AGTGTGTCTG

Orangutan GGAGAGCTCT GCAGCCGAGG CCTTCCGGTG CCGCAGCATC AGTGTGTCTG

Marmoset GGAGAGCTCA GCAGCCGAGG CCTTCCGATG CCGCAGCATC AGTGTGTCTG

Cow GGAGAGCTCT GCAGCCGATG CCTTCCGGTG CCGCAGCATC AGTGTGTCTG

Dog AGAGAGCTCT GCCGCCGAGG CCTTCCGATG CCGCAGCATC AGTGTGTCTG

Guinea_Pig GGAGAGCTCT GCAGCAGAGG CCTTCCGGTG CCGAAGCATC AGTGTGTCTG

Horse GGAGAGCTCT GCAGCCGATG CCTTCCGGTG CCGCAGCATC AGTGTGTCTG

Mouse ---------- ---------- ---------- ---------- ----------

Pig GGAGAGCCTT GCAGCCGATG CCTTCCGGTG CCGCAGCATC AGTGTTTCAG

Rabbit ---------- ---------- ---------- ---------- ----------

Rat AGAGAGCTGT GCAGCCGAGG CCTTCCGGTG CCGCAGCATC AGTGTATCTG

Elephant GGAGAGCTGT GCATCCGAGG CCTTCCAGTG CCACAGCATC AGTGTGTCTG

Frog AGATCTCTCC AAT------G CCTTCCGCTC TCGCAGCATC AGCGTGTCGG

Zebrafish AGACCTTTCG GCCATGGACG CCTTCCGCTC CCGCAGCATC AGTGTGTCCG

Chicken AGAACCCTCT GCAGTTGATG CCTTCCGATC CCGCAGCATC AGTGTGTCTG

Fugu GGACCTGTCA GCCATGGACG CTTTTCGCTC CCGCAGCATC AGCGTCTCTG

Zebra_Finch AGAACCCTCT GCAGTTGATG CCTTCCGATC CCGCAGCATC AGTGTGTCTG

Human AACATGTGGT CCGCAGCAGG ATACAGACGT CCCTCACCAG TGCCAGCTTG

Gorilla AACATGTGGT CCGCAGCAGG ATACAGACGT CCCTCACCAG TGCCAGCTTG

Chimpanzee AACATGTGGT CCGCAGCAGG ATACAGACGT CCCTCACCAG TGCCAGCTTG

Orangutan AACATGTGGT CCGCAGCAGG ATACAGACGT CCCTCACCAG TGCCAGCTTG

Marmoset AACATGTGGT CCGCAGCAGG ATACAGGCGT CCCTCACCAG TGCCAGCCTG

Cow AACATGTGGT CCGCAGCAGG ATCCAGACCT CTCTCACGAG CGCCAGCTTG

Dog AACATGTGGT CCGCAGCAGG ATCCAGACGT CCCTCACAAG CGCCAGCTTG

Guinea_Pig AACATGTGGT CCGCAGCAGA ATACAGACAT CCCTCACCAG TGCCAGCCTT

Horse AACATGTGGT CCGCAGCAGG ATCCAGACAT CCCTCACGAG TGCCAGCTTG

Mouse ---------- ----AGCAGA ATACAGACAT CCCTTACGAG CGCCAGCCTG

Pig AGCACGCGGT CCGCAGCAGG ATCCAGACAT CGCTCACGAG CGCCAGCTTG

Rabbit ---------- -------AGG ATCCAGACGT CGCTCACCAG CGCCAGCCTG

Rat AACATGTGGT CCGCAGCAGA ATACAGACAT CTCTTACCAG TGCCAGCTTG

Elephant AACAGGTGGT CTGCAGTAGG ATCCAGACCT CCTTCACCAG TGCCAGCTTG

Frog AGCATGCTGC GTACAGCCGT ATGCAGACTT CTGTCACCAG CTCCAGCTTG

Zebrafish AGCATGCTGT GCGCAGTAAG ATGCAGACGT CCAGCACCAC CTGCAGTCTG

Chicken AACATGTGGT CCGCAGTCGG ATACAGACAT CCATCACTAG TTCCAGCTTG

Fugu AACATGCGGT TCGATGTAGG ATGCACACCT CCACCACCAC CTGCAGCCTG

Zebra_Finch AACATGTGGT CCGCAGCCGC ATCCAAACGT CCATCACCAG CTCCAGCCTG

Human GGGTCTGCAG ATGAGAACTC CGTGGCCCAG GCTGACGATA GCCTGAAAAA

Gorilla GGGTCTGCAG ACGAGAACTC CGTGGCCCAG GCTGACGATA GCCTGAAAAA

Chimpanzee GGGTCTGCAG ATGAGAACTC GGTGGCCCAG GCTGACGATA GCCTGAAAAA

Orangutan GGGTCTGCAG ACGAGAACTC CGTGGCCCAG GCTGACGATA GCCTGAAAAA

Marmoset GGGTCTGCAG ACGAGAACTC CTTGGCCCAG GCTGACGACA GCCTGAAAAC

Cow GGGTCTGCAG ACGAGAACTC GATGGCCCAG GCCGACGACA ACTTGAAAAA

Dog GGGTCTGCTG ACGAGAACTC GATGGCCCAG GCTGATGACA ACTTGAAAAA

Guinea_Pig GGGTCTGCAG ATGAGAACTC CATGGCCCAG GCTGATGACA ACTTGAAAAA

Horse GGGTCTGCAG ATGAGAACTC AATGGCCCAG GCTGATGACA ACTTGAAAAA

Mouse GGGTCTGCGG ATGAGAACTC TATGGCCCAG GCTGATGACA ACTTGAAGAA

Pig GGGTCTGCAG ATGAGAACTC GATGGCCCAG GCTGACGACA GCCTGAAGAA

Rabbit GGGTCCGCAG ATGAGAACTC CATGGCCCAG GCTGACGACA ACTTGAAAAA

Rat GGGTCTGCGG ATGAGAACTC TATGGCCCAG GCTGATGACA ACCTGAAGAA

Elephant GGTTCTGCAG ACGAAAACTC GATGGCCCAG GCAGACGACA ACCTGAAAAA

Frog AGTTCAGCTG ATGAGAGCTC TATGGCCCAG GCTGATGACA GCCTGAAAAA

Zebrafish GGCTCAGCTG ATGAGAACGC TGTGATGCAG GCGGACGACG CTCTGAAGAC

Chicken GGCTCTGCAG ATGAAAATTC AATGGCTCAG GCTGATGACA ACTTAAAAAA

Fugu GGCTCAGCCG ACGAAAACGC CGTGACCCAA GCGGACGAGG GCCTGAAGAC

Zebra_Finch GGCTCTGCAG ATGAAAACTC CATGGCTCAG GCTGATGACA ACTTGAAAAA

Human CCTCCACCTG GAGCTCACGG AAACCTGTCT GGACATGATG GCTCGATACG

Gorilla CCTCCACCTG GAGCTCACGG AAACCTGTCT GGACATGATG GCTCGATACG

Chimpanzee CCTCCACCTG GAGCTGACGG AAACCTGTCT GGACATGATG GCTCGATACG

Orangutan CCTCCACCTG GAGCTCACGG AAACCTGTCT GGACATGATG GCTCGATACG

Marmoset CCTCCACCTG GAGCTCACGG AAACCTGTCT GGACATGATG GCTCGATACG

Cow CCTCCACCTG GAGCTCACAG AGACGTGTCT GGACATGATG GCCAGATATG

Dog TCTCCACCTG GAGCTCACGG AAACATGTCT GGACATGATG GCCAGATATG

Guinea_Pig TCTCCACCTG GAGCTCACAG AAACATGTCT GGACATGATG GCCCGATACG

Horse TCTCCACTTG GAGCTCACAG AAACGTGTCT GGACATGATG GCCAGATATG

Mouse TCTCCACTTG GAGCTCACAG AAACATGTCT GGACATGATG GCCCGATATG

Pig CCTGCACCTG GAGCTCACAG AAACCTGTCT AGACATGATG GCCAGATACG

Rabbit CCTCCACCTG GAGCTCACAG AAACCTGCCT GGACATGATG GCCCGATACG

Rat TCTCCACTTG GAACTCACAG AAACATGTCT GGACATGATG GCCCGATATG

Elephant TCTCCACCTG GAGCTCACCG AGACGTGCCT GGACATGATG GCCAGATACG

Frog CCTTCACCTG GAATTGACCG AAATCTGCCT GGATATGATG GCACGATACG

Zebrafish AGTCCATCTG GAGCTCACAG AGACCTGCCT GGACATGATG GCCAGATACG

Chicken TCTCCATCTG GAACTCACTG AGACTTGTCT GGATATGATG GCCCGATACG

Fugu TGTTCATTTG GAGCTCACAG AAACGTGTCT AGATATGATG GCGCGATACG

Zebra_Finch CCTGCACCTG GAGCTGACAG AGACCTGCCT GGACATGATG GCTCGATACG

Human TCTTCTCCAA CTTCACGGCT GTCCCGAAGA GGTCTCCTGT GGGCGAGTTC

Gorilla TCTTCTCCAA CTTCACGGCT GTCCCGAAGA GGTCTCCTGT GGGCGAGTTC

Chimpanzee TCTTCTCCAA CTTCACGGCT GTGCCGAAGA GGTCTCCTGT GGGCGAGTTC

Orangutan TCTTCTCCAA CTTCACGGCT GTCCCGAAGA GGTCTCCTGT GGGCGAGTTC

Marmoset TCTTCTCTAA CTTCACGGCT GTGCCAAAGA GGTCTCCCGT GGGAGAATTC

Cow TGTTCTCCAA CTTCACGGCG GTCCCCAAGA GGTCCCCCGT GGGAGAGTTC

Dog TGTTCTCCAA CTTCACAGCA GTCCCCAAGA GGTCCCCGGT GGGAGAGTTC

Guinea_Pig TATTCTCCAA TTTCACTGCA GTCCCCAAGA GGTCTCCTGT AGGAGAGTTT

Horse TGTTCTCCAA CTTCACAGCG GTCCCCAAGA GGTCCCCCGT GGGAGAGTTC

Mouse TGTTCTCCAA TTTCACTGCA GTCCCCAAGA GGTCCCCTGT GGGAGAGTTC

Pig TGTTCTCCAA CTTCACGGCC GTGCCCAAGA GGTCTCCCGT CGGGGAGTTC

Rabbit TGTTCTCCAA CTTCACGGCC GTGCCCAAGA GGTCCCCCGT CGGAGAGTTC

Rat TGTTCTCCAA CTTCACTGCG GTTCCCAAGA GGTCCCCTGT GGGAGAATTC

Elephant TGTTCTCCAA CTTCACAGCT GTCCCCAAGA GGTCTCCCGT GGGAGAATTC

Frog CCTTTGCCAA CTTCACAGCC GTGCCCAAAA GGTCGCCTGT AGGTGAGTTC

Zebrafish TCTTCTCCAA CTTCTCTGCT CTGCCCAAGA GGTCTCCCAT CGCTGATTTC

Chicken TCTTTTCCAA CTTCACTGCG GTGCCTAAGA GGTCTCCTGT GGGTGAGTTT

Fugu TCTTTTCCAA CTTCTCTGCT GCCAATTCAA GGTCCCCCAT CGCTGAGTTC

Zebra_Finch TCTTCTCCAA CTTCACTGCT GTGCCCAAGA GGTCTCCCGT GGGTGAGTTT

Human CTCCTAGCGG GTGGCAGGAC CAAAACCTGG CTGGTTGGGA ACAAGCTTGT

Gorilla CTCCTAGCGG GTGGCAGGAC CAAAACCTGG CTGGTTGGGA ACAAGCTTGT

Chimpanzee CTCCTAGCGG GTGGCAGGAC CAAAACCTGG CTGGTTGGGA ACAAGCTTGT

Orangutan CTCCTGGCGG GTGGCAGGAC CAAAACCTGG CTGGTTGGGA ACAAGCTTGT

Marmoset CTCCTGGCTG GTGGCAGGAC TAAAACTTGG CTGGTTGGGA ACAAGCTTGT

Cow CTCCTGGCTG GTGGCAGGAC CAAAACCTGG CTGGTTGGGA ACAAGCTTGT

Dog CTCCTGGCTG GTGGCAGGAC CAAAACCTGG CTGGTTGGGA ACAAGCTTGT

Guinea_Pig CTCCTGGCTG GTGGCAGGAC CAAAACCTGG CTGGTTGGAA ATAAGCTTGT

Horse CTCCTGGCTG GCGGCAGGAC CAAAACCTGG CTGGTTGGGA ACAAGCTTGT

Mouse CTCCTGGCAG GGGGTAGGAC CAAAACCTGG CTGGTTGGAA ACAAGCTTGT

Pig CTTCTGGCTG GTGGCAGGAC CAAAACCTGG CTGGTTGGGA ACAAGCTGGT

Rabbit CTCCTGGCCG GCGGCAGGAC CAAAACCTGG CTGGTGGGGA ACAAGCTCGT

Rat CTCCTGGCAG GGGGCAGGAC CAAAACGTGG CTGGTTGGAA ATAAGCTGGT

Elephant CTCCTGGCTG GAGGCAGGAC CAAAACCTGG CTGGTGGGGA ACAAGCTGGT

Frog CTGCTGGATG GCGGAAGAAC GAAGACCTGG CTAGTTGGGA ACAAGCTGAT

Zebrafish CTGTTATCTG GTGGTCCTAG TATGACGTGG CTGGTTGGGA ATAAGTTAGT

Chicken CTGTTGGCTG GAGGAAGGAC AAAGACATGG CTGGTTGGCA ACAAGTTGGT

Fugu CTTCTGGCAG GGGGTCGCAG CATGACCTGG CTGGTAGGCA ACAAGCTGGT

Zebra_Finch CTGCTGGCTG GAGGAAGGAC CAAGACGTGG CTGGTTGGTA ATAAGCTGGT

Human CACTGTGACG ACAAGCGTGG GAACCGGGAC CCGGTCGTTA CTAGGCCTGG

Gorilla CACTGTGACG ACAAGCGTGG GAACCGGGAC CCGGTCGTTA CTAGGCCTGG

Chimpanzee CACTGTGACG ACAAGCGTGG GAACCGGGAC CCGGTCGTTA CTAGGCCTGG

Orangutan CACTGTGACG ACAAGCGTGG GAACCGGGAC CCGGTCGTTA CTAGGCCTGG

Marmoset CACCGTGACC ACAAGCGTGG GAACGGGGAC GCGTTCGTTG CTAGGTCTGG

Cow CACCGTGACG ACAAGCGTGG GGACCGGGAC CCGGTCACTG CTAGGCCTGG

Dog CACTGTGACG ACGAGCGTGG GGACGGGGAC CCGGTCGCTG CTGGGCTTGG

Guinea_Pig CACTGTGACC ACAAGTGTTG GGGCTGGGAC CCGGTCATTG CTGGGCCTGG

Horse CACCGTGACA ACGAGTGTGG GGACTGGTAC CCGGTCACTG CTGGGCCTGG

Mouse CACTGTGACA ACAAGTGTGG GAACTGGTAC ACGGTCGCTG CTGGGCCTGG

Pig CACCGTGACG ACAAGCGTGG GGACCGGGAC GCGGTCACTC CTGGGCTTGG

Rabbit CACGGTGACG ACAAGCGTGG GGACTGGGAC CCGGTCCCTG CTGGGCCTGG

Rat CACTGTGACA ACGAGTGTGG GAACTGGTAC GCGGTCGTTG CTGGGCCTGG

Elephant TACCGTAACG ACGAGTGTGG GAACTGGCAC ACGGTCGCTG CTGGGCCTAG

Frog TACCATTACA ACAAGTGTGG GCATGGGGAC ACGTTCTCTT CTTGGCCTGG

Zebrafish GACCATAACC ACCAGCGGCG GCTCGCGAAC ACAAGCTCTT CTGGGTCTGG

Chicken GACCATCACT ACAAGTGTCG GAACAGGGAC AAGGTCCTTG CTTGGCCTGG

Fugu GACCATAACC ACTAGTGGAG GTGTCAGAAC ACAAGCGCTG CTCGGCCTGG

Zebra_Finch GACCATCACT ACAAGTGTTG GAACAGGGAC AAGGTCCCTG CTTGGCCTGG

Human ACTCGGGGGA GCTGCAGTCC GGCCCGGAGT CGAGCTCCAG CCCCGGGGTG

Gorilla ACTCGGGGGA GCTGCAGTCC GGCCCGGAGT CGAGCTCCAG CCCCGGTGTG

Chimpanzee ACTCGGGGGA GCTGCAGTCC GGCCCGGAGT CGAGCTCCAG CCCCGGTGTG

Orangutan ACTCGGGGGA GTTGCAGTCC GGCCCAGAGT CGAGCTCCAG CCCCGGCGTG

Marmoset ACTCGGGGGA GCTGCAGGCC GGCCCAGAGT TGAGCTCCAG CCCTGTTGTG

Cow ACTCTGGAGA GCTGCAGGGT GGCCCGGAGT TGAGCTCTGA CCCCAGCATG

Dog ACTCAGGAGA TCAGCAAGGT GGCCCCGAGT TGAGCTCGGA CCCTGGCATG

Guinea_Pig ACTCAGGCGA GCTGCAAGGT GCCCCAGAGT CAAGCTCTGA GCCTACTATG

Horse ACTCAGGAGA GCTGCAGGGC TGCCCAGAGT TAAGCTCCGA CCCCAGTGTG

Mouse ACTCTGGGGA CCTGCAGGGT GGCTCGGATT CAAGCTCTGA TCCTAGCACA

Pig ACTCAGGAGA GCTGCAGGGC GGCCCAGAGT TGAGCTCCGA CCCCGGTGTG

Rabbit ATTCCGGGGA GCTGCAGGGC GGCCCAGAGT TGAGCTCTGA CCCTGGCGTG

Rat ACTCCGGGGA TCTGCAGGGT GGCTCAGCAT CAAGCTCTGA TCCTGGCACA

Elephant ACCCAGGCGA GCTGCAGGGT GGCTCAGAGC TTAGCTCCAG CCCTGGTGTA

Frog ATACAGGAGG GGAGAACCAT GGT---GACA CTGAGAACAG CTCCCCCGTG

Zebrafish ACATGACAGA GAGACATGGA GGAGAAATGA CCAGGTCTGA CCCGTCTCTG

Chicken ACTCTGGAGA TCTCCAAAGC AGCACAGAAT CAGGCTCAGA CCCGGTTTTG

Fugu ATGTGGTTGA ACGCTTGGGA GGAGAAATGA CAAGGTCGGA TCCATCACTG

Zebra_Finch ACTCTGGAGA ATTCCAGAGC ACCCCAGAAT CAAGCTCAGA CCCTGTTTTG

Human CATGTGAGAC AGACCAAGGA GGCGCCGGCC AAGCTGGAGT CCCAGGCTGG

Gorilla CATGTGAGAC AGACCAAGGA GGCGCCGGCC AAGCTGGAGT CCCAGGCTGG

Chimpanzee CATGTGAGAC AGACCAAGGA GGCGCCGGCC AAGCTGGAGT CCCAGGCTGG

Orangutan CATGTGAGAC AGACAAAGGA GGCGCCGGCC AAGCTGGAGT CCCAGGCTGG

Marmoset CACGTGAGAC AGACGAAGGA GGCGCCAGCC AAGCTGGAGT CCCAGGCTGG

Cow CATGTGAGAC AGACAAAGGA GGCACCCGCC AAGCTGGAGT CCCAAGCTGG

Dog CATGTGAGAC AGGCAAAGGA GGCACCTGCC AAGCTGGAGT CGCAGGCTGG

Guinea_Pig CACATAAGAC AGACCAAGGA GGCACCTGCA AAGCTGGAGT CCCAGGCTGG

Horse CACGTGAGAC AGACAAAGGA GGCGCCAGCC AAGCTGGAGT CCCAGGCTGG

Mouse CATGTGAGGC AGACAAAGGA AGCACCGGCC AAACTGGAGT CCCAGGCTGG

Pig CACGTGAGAC AGATAAAGGA GGCGCCGGCC AAGCTGGAGT CCCAGGCTGG

Rabbit CACTTGAGAC AGACAAAGGA GGCACCGGCC AAGCTGGAGT CCCAGGCCGG

Rat CATGTGAGGC AGACAAAGGA AGCACCGGCC AAACTGGAGT CCCAGGCTGG

Elephant CACGTGAGGC AGACGAAAGA GGCGCCAGCC AAGCTGGAGT CCCAGGCTGG

Frog CAAACAGAGC AAGTCAAGGA GTTTCCGTCC ATGCTCGATC TTCAGCAAAG

Zebrafish CACACCCGAC AGACCAAAGA AGCTCCCGCT AAACTAGAGT CTCAGTCCAG

Chicken CAAGTGAGAC AAACTAAAGA AGCTCCAGCA AAGCTCGAAT CTCAAGCTGG

Fugu CACACCCGGC TAACCAAAGA GGCTCCAGCC AAGCTTGAGT CCCAGTCCAG

Zebra_Finch CAAGTGAGAC AGACCAAGGA GGCTCCAGCC AAGCTGGAAT CCCAGGCTGG

Human GCAGCAGGTG TCCCGTGGGG CCCGGGATCG GGTCCGTTCC ATGTCGGGGG

Gorilla GCAGCAGGTG TCCCGTGGGG CCCGGGACCG GGTCCGTTCC ATGTCAGGGG

Chimpanzee GCAGCAGGTG TCCCGTGGGG CCCGGGACCG CGTCCGTTCC ATGTCGGGGG

Orangutan GCAGCAGGTG TCCCGTGGGG CCCGGGACCG GGTCCGTTCC ATGTCAGGGG

Marmoset GCAGCAGGTG TCCCGTGGGG CCCGGGACCG GGTCCGCTCC ATGTCGGGGG

Cow GCAGCAGGTG TACCACGGAG CCCGGGACCG GGTCCGCTCC ATGTCCGGGG

Dog GCAGCAGGTG TGCCGCGGCG CCCGGGACCG GGTCCGCTCC ATGTCCGGGG

Guinea_Pig GCAGCAGGTA TCCCGTGGGG CCCGGGACCG TGTCCGCTCC ATGTCTGGGG

Horse GCAGCAGGTG TGCCGCGGGG CCCGGGACCG GGTCCGCTCC ATGTCTGGGG

Mouse GCAGCAGGTG TCCCGTGGGG CCCGGGACCG GGTCCGCTCC ATGTCAGGGG

Pig GCAGCAGGTG TGCCGCGGGG CCCGGGACCG GGTCCGCTCC ATGTCTGGGG

Rabbit GCAGCAGGTG TCCCGTGGGG CCCGGGACCG CGTCCGTTCC ATGTCGGGAG

Rat GCAGCAGGTG TCCCGTGGGG CCCGGGACCG AGTCCGCTCC ATGTCAGGGG

Elephant GGTACAGGTG TGCCGCAGGG CCCGGGACCG GGTCCGCTCC ATGTCAGGTG

Frog AAAGCCGGGT ATGCAAACGC CCCGTCACAG AGTCCGCTCC ATGTCTGGAG

Zebrafish CCAACAGATC AACACCAACA CACGCACACG AGTCCGCTCT ATATCAGGTG

Chicken GCAGCAGGTT TGCAGAAGCT CTCGCAACAG AGTCAGATCC ATGTCTGGTG

Fugu CCAGCAGCAA AGCAGAGCCA CGCGCACCAG AGTCCGCTCC ATGTCAGGTG

Zebra_Finch GCAGCAGGTT TGCAGGAGCT CCCGCAACAG AGTCCGATCC ATGTCGGGTG

Human GCCATGGTCT TCGAGTTGGC GCCCTGGACG TGCCGGCCTC CCAGTTCCTG

Gorilla GCCATGGTCT TCGAGTTGGC GCCCTGGACG TGCCGGCCTC CCAGTTCCTG

Chimpanzee GCCATGGTCT TCGAGTTGGC GCCCTGGACG TGCCGGCTTC CCAGTTCCTG

Orangutan GCCATGGTCT TCGAGTTGGC GCCCTGGACA TGCCGGCCTC CCAGTTCCTG

Marmoset GACACGGTCT TCGAGTCGGC GCCCTGGACG TGCCAGCCTC CCAGTTCCCG

Cow GCCATGGCCT TCGCGTGGGT GCCTTGGACG CTCCAGCCTG CTACTTCCCC

Dog GCCACGGTCT TCGTGTTGGC GCCCTGGACA CTCCAGCCTC CCACTTGCCG

Guinea_Pig GCCATGGCCT TCGGGTTGGT GCTCTGGATA CACCAGCCCC CCATACTACT

Horse GCCATGGCCT TCGCGTTGGT GCCCTGGATG CTTCAGCCTC CCACTTCCCA

Mouse GCCATGGCCT TCGAGTTGGT GTCCTGGATA CTTCAGCTCC CTATTCCCCA

Pig GCCACGGCCT TCGTGTTGGC ACCTTGGATG CTCCGGCCTC ACACTTCCCA

Rabbit GCCACGGCCT TCGGGTCGGT GCCCTGGACC CACCGACCTC CCACTTCCCC

Rat GCCACGGCCT TCGAGTTGGT GTCCTGGACA CTTCAGCTCC CTACACCCCA

Elephant GCCATGGCCT GCGCGTGGGA GCCCTGGACA CTCTAGCCTC CTACTTCCCT

Frog GGACTACACT GAGAGCTGGA TCACTAGAGG GG------AC TCCTCTTTCG

Zebrafish GTCATGCTCT GCGGGCCGGG CCAGGCGAGT ACAGCGGCCC GCTGCCTCCT

Chicken GTCATGCCTT ACGTGTTGGT GCCTTAGACA GCACAGCCTC CCACTTCCCT

Fugu GCCATGCTCT TCGGTCTCTT CCTACCCAGA ATCTCAGTCC TCTTGTGTCA

Zebra_Finch GTCATGCCTT ACGTGTTGGT GCCTTAGACA GCTCAGCCTC CCATTTTCCC

Human GGCAGTGCCA CTTCTCCAGG ACCACGGACT GCACCAGCCG CGAAACCTGA

Gorilla GGCAGTGCCA CTTCTCCAGG ACCACGGACT ACACCAGCCG CGAAACCTGA

Chimpanzee GGCAGTGCCA CTTCTCCAGG ACCACGGACT GCACCAGCCG TGAAACCTGA

Orangutan GGCAGTGCCA CTTCTCCAGG ACCACGGACT GCACCAGCCG CGAAGCCTGA

Marmoset GGCAGTGCAA CTTCTCCAGG ACCACAGACT GCACCAGCTA TGAAGGCTGA

Cow AGCAGCCCCA CGTCCCCGGG CTCCCAGACC GCTCCAGCCA GCCAGCCCGA

Dog GGTGGCCCCA CTTCCCCAGG CACGCAGGCA GCGCTGGCCA GCAAGTCTGA

Guinea_Pig GGTGGCCCTG CTGCCCCGGG CCTGCAGACT GCACCAGCCA CAAAGCCTGA

Horse GGCGGCCTCA CTTCCCCGGG CCCACAGACC ACACCAGCCA GCAAGCCTGA

Mouse GGTGGCTCTG CTTCTCTGGG ACCACAGACG GCGGTGGCAG CGAAGCCCGA

Pig GGCAGCCCCT CTTCCCCGGG CTCAGAGACT GCCCCAGCCG GCAAGCCCGA

Rabbit GGCAGCCCGT CTGCTCCGGG AACACAGGCT GCGCCAGCTG CGAAGCCTGA

Rat GGAGGCCCTG CTTCTCTGGG AGCACAGGCA GCACCAGCAG CAAGGCCTGA

Elephant GGAGGCCCTA TTTGTCCAGG GCCGCAGACC ATGCCGGTCG TGAAGCCTCA

Frog GCCAGTATGG GGCAGCCGGG GGCCACT--- ---CCAGTGA CCAGAGCAGA

Zebrafish CCC---AGCT CTCAGAGGGG G------GAG CACCCGCCAC CCTCCGCCTG

Chicken GGTGGCTCGG CTTCACAAGG GACACAGCCT GCTCCTGCAC CTCGGTCAGA

Fugu CCCTCTTCTG CCTCTACTGG CCCATCAGGA CTGGGCCCCC CGTCCTCATC

Zebra_Finch AGTGGCTCAA CTTCCCAAGG GACACAGCCT GCTGCTGCAG CCCGCCCAGA

Human GAAGGCCTCA GCTGGCACCC GGGTTCCTGT GCAG---GAG AAGACGAACC

Gorilla GAAGGCCTCA GCTGGCACCC GGGTTCCTGT GCAG---GAG AAGACGAACT

Chimpanzee GAAGGCCTCA GCTGGCACCC GGGTTCCTAT GCAG---GAG AAGACGAACC

Orangutan GAAGGCGTCA GCTGGCACCC GGGTTCCTGT GCAG---GAG AAGACGAACT

Marmoset GAAAGCCAGC ------ACCC GGCTTCCCGT GCAG------ AAGACAAACC

Cow GAAGGCCTCA GCTGGCAGCC CGCTCCCAGC ACAGAAGGAG AAGACAAACC

Dog CAGGGCCTCG GCTGGCACCC GGTTCCCAGC ACAG---GAG AAGACGAGCC

Guinea_Pig GCAGACTTCT GCAAGTGCAC CGGTTTTAGC ACCA---GAG AAGACGAACC

Horse GAAGGCCTCA GCTGGCACCC AGCTCCCAGC GCAGAAGGAG AAGACGAACC

Mouse GAAGCCCCCT GCAGGAGCCC AGCTTCCAAC AGCA---GAG AAGACGAATC

Pig AAAGGCCTCG GCTGGCTCCC AGCTCCCTGC GCAGAAGGAG AAAACAAACC

Rabbit CAGGGCCTCA GCAGGCACCC AGGGCCCAGC ACAG---GAG AGGACGAACC

Rat GAAGCCCTGT GCAGGAGCCC AACTTCCGGC AGCG---GAG AAGGCGAACC

Elephant GAAAGCCTCA GCGAGCACCC AGATGCCAGT GCCAAAGGAA AAGACAAACC

Frog GAAACCTTCC CGTAGGACAC AGATG----- ----AAAGAA AAGACCACCC

Zebrafish CAACCCATCA CCA------- -----CCGCT C---CACAAG CAC---AGCC

Chicken GAAGACTAAT CCTGCTCCAC AGACACCTCT GCAGAAAGAA AAAGCGAACT

Fugu TTGCCCCACC CCGAATGCTC CCCCGCCCCT C---AAAGAC AACCCAAGTC

Zebra_Finch GAAGGCCAGT CCTGCTCCAC AGACAGCCCT GCAGAAGGAA AAAGCCAACT

Human TGGCGGCCTA TGTGCCCCTG CTGACCCAGG GCTGGGCGGA GATCCTGGTC

Gorilla TGGCGGCCTA TGTGCCCCTG CTGACCCAGG GCTGGGCGGA GATCCTGGTC

Chimpanzee TGGCGGCCTA TGTGCCCCTG CTGACCCAGG GCTGGGCGGA GATCCTGGTC

Orangutan TGGCGGCCTA CGTACCCCTG CTGACCCAGG GCTGGGCGGA GATCCTGGTC

Marmoset TGGCGGCCTA TGTGCCCCTG CTGACCCAGG GCTGGGCGGA GATCCTGGTC

Cow TGGCCGCTTA CGTGCCCCTG CTGACCCAGG GCTGGGCAGA GATCCTGGTC

Dog TGGCAGCCTA CGTACCGCTG CTGACCCAGG GCTGGGCGGA GATCTTGGTC

Guinea_Pig TGGCAGCCTA TGTGCCACTG CTTACCCAGG GCTGGGCAGA AATCCTGGTC

Horse TGGCAGCCTA TGTGCCCTTG CTGACCCAGG GCTGGGCTGA GATCTTGGTC

Mouse TGGCAGCCTA TGTGCCTTTG TTAACCCAGG GCTGGGCAGA AATCTTAGTC

Pig TGGCGGCCTA CGTCCCCCTG TTGACCCAGG GCTGGGCGGA GATCTTGGTC

Rabbit TGGCAGCCTA CGTGCCTCTG CTGACCCAGG GCTGGGCTGA GATCTTGGTC

Rat TGGCAGCCTA TGTGCCTTTG CTGACCCAGG GCTGGGCAGA GATCCTGGTC

Elephant TGGCTGCCTA CGTGCCCCTC CTGACCCAGG GCTGGGCTGA GATCCTGGTC

Frog TGGCTGACTT TGTTCCCGTG CTGACACAAG GCTGGGCAGA GGTATTGGTT

Zebrafish TGGCAGAGTT TGTGCCCATG CTGACGCAGG GCTGGGCCGA GATCTTCATC

Chicken TAGCTGCATA TGTCCCACTG CTGACACAGG GCTGGGCAGA AATCCTTGTG

Fugu TAGCGGAGTT TGTCCCCATG CTCACTCAGG GCTGGGCCGA AATCTTCATA

Zebra_Finch TGGCTGCCTA CGTGCCACTG CTGACCCAGG GCTGGGCAGA GATCCTGGTG

Human CGGAGGCCCA CAGGGAACAC CAGCTGGCTG ATGAGCCTGG AGAACCCGCT

Gorilla CGGAGGCCCA CAGGGAACAC CAGTTGGCTG ATGAGCCTGG AGAACCCGCT

Chimpanzee CGGAGGCCCA CAGGGAACAC CAGCTGGCTG ATGAGCCTGG AGAACCCGCT

Orangutan CGGAGGCCCA CAGGGAACAC CAGCTGGCTG ATGAGCCTGG AGAACCCGCT

Marmoset CGGAGGCCCA CAGGGAACAC CAGCTGGCTG ATGAGCCTGG AGAACCCCCT

Cow CGCAGGCCCA CAGGGAACAC CAGCTGGCTG ATGAGCCTGG AGAACCCGCT

Dog CGGAGGCCTA CAGGGAACAC CAGCTGGCTG ATGAGCCTGG AGAACCCGCT

Guinea_Pig CGGAGGCCCA CAGGGAACAC CAGCTGGCTG ATGAGCCTGG AGAATCCACT

Horse CGGAGGCCCA CAGGGAACAC CAGCTGGCTG ATGAGCCTGG AGAACCCGCT

Mouse CGCAGACCCA CAGGAAACAC CAGCTGGCTG ATGAGCTTGG AGAACCCGCT

Pig CGAAGACCCA CAGGGAACAC CAGCTGGCTG ATGAGCCTGG AGAACCCGCT

Rabbit CGCAGGCCCA CAGGGAACAC AAGCTGGCTG ATGAGCCTGG AGAACCCGCT

Rat CGCAGGCCCA CAGGAAACAC CAGCTGGCTG ATGAGCTTGG AGAACCCGCT

Elephant CGGAGGCCCA CAGGGAACAC CAGTTGGTTG ATGAGCCTGG AGAATCCACT

Frog CGCAGACCCA CAGGTAACAC CAGCTGGTTG ATGAGCCTGG AAAATCCTCT

Zebrafish CGCAGGCCCT CAGGGAACAC GAGCTGGCTG ATGTGTCTGG AGAACCCGCC

Chicken CGCAGACCCA CAGGTAACAC AAGCTGGCTA ATGAGTCTGG AGAACCCGCT

Fugu CGGAGACCAT CAGGCAACAC CAGCTGGCTG ATGTGTCTGG AGAATCCACC

Zebra_Finch CGCAGGCCCA CAGGAAACAC GAGCTGGCTG ATGAGCCTGG AGAACCCTCT

Human CAGCCCTTTC TCCTCGGACA TCAACAACAT GCCCCTGCAG GAGCTGTCTA

Gorilla CAGCCCTTTC TCCTCGGACA TCAACAACAT GCCCCTGCAG GAGCTGTCCA

Chimpanzee CAGCCCTTTC TCCTCGGACA TCAACAACAT GCCCCTGCAG GAGCTGTCCA

Orangutan CAGCCCTTTC TCTTCGGACA TCAACAACAT GCCCCTGCAG GAGCTGTCCA

Marmoset CAGCCCCTTC TCTTCAGACA TCAACAGCAT GCCGCTGCAG GAGCTGTCCA

Cow CAGCCCCTTC TCCTCGGACA TCAACAGCAT GCCGCTGCAG GAGCTGTCCA

Dog CAGCCCCTTC TCGTCGGACA TCAACAACAT GCCTCTGCAG GAGCTGTCCA

Guinea_Pig CAGCCCCTTC TCCTCGGACA TCAACAACAT GCCCCTGCAG GAGCTGTCCA

Horse CAGCCCCTTC TCCTCGGACA TCAACAACAT GCCTCTGCAG GAGCTCTCCA

Mouse CAGCCCCTTC TCCTCAGACA TCAACAACAT GCCCCTGCAA GAGCTGTCCA

Pig CAGCCCCTTC TCCTCGGACA TCAACAACAT GCCCCTGCAG GAGCTGTCCA

Rabbit GAGCCCCTTC TCGTCCGACA TCAACAACAT GCCCCTGCAG GAGCTGTCCA

Rat CAGCCCCTTT TCCTCAGACA TCAACAACAT GCCCCTGCAA GAGCTGTCTA

Elephant AAGCCCTTTC TCTTCGGACA TCAACAATAT GCCACTGCAG GAGTTGTCCA

Frog GAGCCCCTTC TCTTCTGACA TAAACAACAT GCCGCTGCAG GAGCTTTCTA

Zebrafish AAGCCCGTTC TCCTCTGAGC TGGGTAATCT GCCTCTGCAG GAGCTCTCCA

Chicken CAGTCCCTTT TCTTCAGACA TTAACAATAT GCCCCTGCAA GAGCTGTCCA

Fugu AAGCCCCTTT TCCTCCGAGC TTGGCAACAT GCCGCTGCAG GAGCTTTCCA

Zebra_Finch GAGCCCCTTC TCCTCAGACA TCAACAACAT GCCCCTGCAG GAGCTCTCCA

Human ACGCCCTCAT GGCGGCTGAG CGCTTCAAGG AGCACCGGGA CACAGCCCTG

Gorilla ATGCCCTCAT GGCGGCTGAG CGCTTCAAGG AGCACCGGGA CACAGCCCTG

Chimpanzee ACGCAAACAT GGCGGCTGAG CGCTTCAAGG AGCACCGGGA CACATTACGG

Orangutan ACGCCCTCAT GGCGGCTGAG CGCTTCAAGG AGCACCGGGA CACAGCCCTG

Marmoset ACGCCCTCAT GGCGGCTGAG CGCTTCAAGG AGCACCGGGA CACAGCCCTG

Cow ATGCCCTCAT GGCCGCCGAG CGCTTCAAGG AGCGCCGCGA CACAGCCCTG

Dog ACGCGCTCAT GGCCGCTGAG CGCTTCAAGG AGCGTCGTGA CACGGCTCTG

Guinea_Pig ATGCCCTTAT GGCTGCTGAG CGCTTCAAGG AGCACCGGGA CACTGCCTTG

Horse ACGCCCTGAT GGCCGCTGAG CGCTTCAAGG AGCGCCGCGA CACGGCCCTG

Mouse ATGCCCTTAT GGCTGCTGAA CGTTTCAAGG AGCACCGGGA CACGGCCCTG

Pig ATGCCCTCAT GGCCGCCGAG CGCTTCAAGG AGCGCCGGGA TACGGCGCTG

Rabbit ACGCCCTCAT GGCGGCCGAG CGCTTCAAGG AGCATCGCGA CACCGCCCTG

Rat ATGCCCTTAT GGCTGCTGAG CGCTTCAAGG AGCACCGGGA CACGGCCCTG

Elephant ATGCTCTCAT GGCCGCTGAG CGCTTCAAGG AGCACCGTGA CACGGCCCTG

Frog ATGCCCTAAT GGCAGCCGAG AGGCTCAAGG AGCATCGGGA GACAGCGCTC

Zebrafish GCGTCCTCAT GGCCATGGAC GGAGTGAAGG AGCCACCG-- -------GCC

Chicken ACGCACTCAT GGCTGCAGAG CGTTTTAAAG AGCACCGAGA AACAGCTCTC

Fugu GCGTCCTTAT GGCCATGGAG GGGGTGAAGG AACCTCCTAG CACTGCTGCT

Zebra_Finch ACGCCCTGAT GGCCGCAGAA CGCTTCAAGG AGCACCGGGA GACGGCTCTG

Human TACAAGTCAC TGTCGGTGCC GGCAGCCAGC ACGGCCAAAC CCCCTCCTCT

Gorilla TACAAGTCAC TGTCGGTGCC GGCAGCCAGC ACGGCCAAAC CCCCTCCTCT

Chimpanzee GACAAGTCAC TGTCGGTGCC GGCAGCCAGC ACGGCC---- ----------

Orangutan TACAAGTCAT TGTCGGTGCC GGCGGCCAGC ACGGCCAGAC CCCCTCCTCT

Marmoset TATAAGTCGC TGTCAGTGCC CGCGGCCAGC ACGGCCAAAC CCCCTCCTCT

Cow TACAAGTCGC TGTCGGTGCC GGCGGCCGGC TCAGCCAAGC CATCCCCGCC

Dog TACAAGTCGC TGTCGGTGCC TGCCGCAGGC TCGGCCAAGC CCCCTCCGCC

Guinea_Pig TACAAGTCCC TGTCGGTGCC AGCAGCTGGC ACAGCCAAGC CCCCTCCTCT

Horse TACAAGTCAC TGTCAGTGCC AGCAGCTGGC TCAGCCAAGC CTTCCCCACC

Mouse TACAAGTCAT TGTCAGTGCC AGCGGCTGGC ACCGCCAAGC CTCCTACTCT

Pig TACAAGTCCC TGTCGGTGCC GGCGGCCGGC TCGGCAAAGC CTCCCCCGCC

Rabbit TACAAGTCGC TGTCCGTGCC GGCCGCCAGC ACAGCCAGGC CCCCTCCTCT

Rat TACAAGTCAT TGTCAGTGCC AGCGGCTGGC ACAGCCAAGC CTCCTACTCT

Elephant TACAAGTCAT TGTCGGTGCC AGTGGCCAGC GCCTCCAAGC CCTCCCTCCT

Frog TACAAATCTC TCTCTGTGCC ATCACCCTCA ACAACCAAGT GTACCCTTCT

Zebrafish GAGCGTCCCA CAGACGTGTC CCAGCCACAG GGC---AAAC CGGCTCCCAT

Chicken TACAAATCCC TGTCTGTCCC TGCCACTGGG ACAGCCAAGC CTTCACTTCT

Fugu CCTGCGCCGA CGCTCGTCTC CGAGCCCCCA AACCAGAGAC CAAATGTGAT

Zebra_Finch TACAAATCCC TGTCCGTGCC CGCCACGGGC ACGGCCAAGC CCTCGCTGCT

Human GCCTCGCTCC AACACAGTGG CCTCTTTCTC CTCCCTGTAC CAGTCCAGCT

Gorilla GCCTCGCTCC AACACAGTGG CCTCTTTCTC CTCCCTGTAC CAGTCCAGCT

Chimpanzee ---------- ------ATGG CCTCTTTCTC CTCCCTGTAC CAGTCCAGCT

Orangutan GCCTCGCTCC AACACAGTGG CCTCTTTCTC CTCCTTGTAC CAGTCCAGCT

Marmoset GCCACGCTCC AACACAGTGG CCTCTTTCTC CTCCCTGTAC CAGTCCAGCT

Cow CCCACGCTCC AAC------- ---------- ---------- ----------

Dog CCCGAGATCT AACACGGTGG CCTCTTTCTC CTCCCTGTAC CAGTCCAGTT

Guinea_Pig CCCACGCTCT AATACAGTGG CCTCTTTCTC CTCCTTGTAC CAGTCCAGCT

Horse CCCACGGTCT AACACAGTGG CCTCTTTCTT CTCCCTGTAC CAGTCCAGTT

Mouse CCCACGCTCT AAT------- ---------- ---------- ----------

Pig CCCGCGCTCT AAC------- ---------- ---------- ----------

Rabbit CCCTCGCTCT AAC------- ---------- ---------- ----------

Rat CCCACGTTCT AACACAGTGG CTTCTTTCTC CTCCCTGTAC CAGCCCAGCT

Elephant GCCCCGCTCC AACACAGTGG CCTCTTTCTC CTCCCTGAGC CAGTCCAGTT

Frog ACAGCGGGCA AACACAGTGG CCTCTGTGTC TCTCGTAACC CAGTCCAGTG

Zebrafish CCAGCGATCC AACACTGTGG CCTCTCTCAG CTCTCTCTGC TCCTCTGAAT

Chicken CCAGCGTTCC AACACAGTGG CCTCTTTCTC TTCCATGTAC CAGTCCAGTT

Fugu CCAGCGCTCC AACACTGTTT TTTCCCATCT TTACCACTGT GCTGCCCCT-

Zebra_Finch GCAGCGCTCC AACACAGTGG CCTCTTTCTC TTCCATGTAC CAGTCCAGTT

Human GCCAAGGACA GCTGCACAGG AGCGTTTCCT GGGCAGACTC CGCCGTGGTC

Gorilla GCCAAGGACA GCTGCACAGG AGCGTTTCCT GGGCAGACTC CGCCGTGGTC

Chimpanzee GCCAAGGACA GCTGCACAGG AGCGTTTCCT GGGCAGACTC CGCCGTGGTC

Orangutan GCCAAGGACA GCTGCACAGG AGCGTTTCCT GGGCAGACTC CGCTGCGGTC

Marmoset GCCAAGGACA GCTGCACAGG AGCATTTCCT GGGCAGACTC GGCCGTGGTC

Cow ---------- ---------- ---------- --ACAGATTC TGCGGTGGTC

Dog GCCAAGGAAA GCTGCACAGG AGCATTTCCT GGGCAGACTC TGCGGTGGTT

Guinea_Pig GCCAAGGACA GCTGCACAGG AGCATTTCCT GGGCAGACTC CGCTGTGGTT

Horse GCCAAGGAAA GCTGCACAGG AGCATTTCCT GGGCAGACTC TGCGGTGGTT

Mouse ---------- ---------- ---------- --ACAGACTC AGCCATGGTC

Pig ---------- ---------- ---------- --ACAGACTC TGCGGTGGTT

Rabbit ---------- ---------- ---------- ---------- ----------

Rat GCCAAGGACA GCTGCACAGG AGCGTTTCCT GGGCAGACTC GGCCGTGGTT

Elephant GCCAAGGAAA GCTGCACAGG AGCGTTTCCT GGGCAGACTC CCCGGGGGTT

Frog GTCAAGGAAA GCTGCTTAGG AGCATTTCCT GGGCAGAATC TTGTGCTGTG

Zebrafish CATCCGGCAG GTTTCACAGG AGTATATCCT GGGCAGATTC AGTAGTGATG

Chicken GTCAAGGGAA GTTGCACAGG AGCATATCCT GGGCAGAGTC TGCGGTCGTG

Fugu -----GGCAG GCTGCACAGG AGCATTTCCT GGGCAGGTAC TGTTCATCAC

Zebra_Finch GTCAAGGGAA GTTGCACAGG AGCATATCCT GGGCAGAATC CGCCGTGGTG

Human ATGGAGGAGG GAAGTCCGGG CGAGGTTCCT GTGCTGGTGG AGCCCCCAGG

Gorilla ATGGAGGAGG GAAGTCCGGG CGAGGTTCCT GTGCCGGTGG AGCCCCCAGG

Chimpanzee ATGGAGGAGG GAATTCCGGG CGAGGTTCCT GTGCCGGTGG CGCCCCCAGG

Orangutan GTGGAGGAGG GAAGTCCTGG CGAGGTTCCT GTGCCAGTGG AGCCCCCAGG

Marmoset ATGGAGGAGG GAAATCCT-- ---------- GCCCCAGTGG AGCCCCCAGA

Cow CTGGAGGAGG GAGGTCCAGG TGAGGCTGGT TTGTCTGCAG AGCCTCCCGA

Dog CTGGAGGAGG GAAATTCAAG CGAGACCAGT CTGCCAGTGG AGCACACGGA

Guinea_Pig CTGGAGGAAG GAAGTCCATG CGAGACTCAC ATACCAGGGG AGCCCCCAGA

Horse CTGGAGGAGG GAAGTCCAGG CGAGGCTAAT GTGCCAGTGG AACCCCCCGA

Mouse CTTGAGGAGG GAAGTCCAGG AGAGACTCAG GTGCCAGTGG AGCCCCCCGA

Pig CTGGAGGAGG GAGGTCCAGG CGAGCCTGGT TGGCCAGGGG GGGTCCCCGA

Rabbit ---------- ---------- ---------- ---------- ----------

Rat CTTGAGGAGG GAAGTCCAGG AGAGGCTCAT GTGCCAGTGG AGCCCCCCGA

Elephant CTGGAGGAAG GATGTGCAGG TGAGATGAAC TTGTCCACTG AGCTTCCTGA

Frog CCAGAGGAAG GGGCGCAAGC TGAAGTGTCC ACTCCCGGGG GCTCTCAAGA

Zebrafish GAGGAAAGTG GGCGGAGT-- -GCGTCTGTT TCACCTGCTG AGTCTAAAGA

Chicken CTGGAGGAAG GGAGCCCCTT GGAAACTGAG TCCCCAGCTG AGTCTCCAGA

Fugu ---------- ---------- -TGTGTCACC GCTCCACCCC TTCAGAAAAC

Zebra_Finch CTGGAGGAGG GCAGCCCGGG GGAAGCCGAG TCCCCCGCCG AGTCCCCCGA

Human GTTGGAGGAC GTTGAGGCAG CGCTAGGCAT GGACAGG--- ------CGCA

Gorilla GTTGGAGGAC GTTGAGGCGG CGCTAGGCAT GGACAGG--- ------TGCA

Chimpanzee GTTGGAGGAC GTCGAGGCAG CGCTAGGCAT GGACAGG--- ------CGCA

Orangutan CTTGGAGGAC TTTGAGGCAG CGCTAGGCAT GGACAGG--- ------CGCA

Marmoset GTTGGAGGAA TTCGAGCCGG CTCTGGGCAC AGACAGA--- ------CGCA

Cow GTTGGAAGAT TTTGAGGCGA CACTGGGCTC CGACGGGCGC TGTGGGCGCA

Dog GCTGGAGGAC TTCGAGGCGA CACTAGGCAC AGACCGGCGC TGTGGCCGCG

Guinea_Pig GTTGGAGGAC TTCGAAGCAG CACTAGATAC AGACAAGCGC TGCCAACGCC

Horse CTTAGAGGAC TTCGAGGCAA CACTCGGCAC AGACAGGCGC TGTCCGCGCA

Mouse ATTGGAGGAT TTCGAGGCGG CGTTAGGTAC AGACAGGCAC TGCCAGCGGC

Pig GTTGGAAGAC TTGGAGGCGC CGCTAGACAC AGGTCAGCGC TGCTGCCGCG

Rabbit ---------- ---------- ---------- ---------- ----------

Rat ATTGGAGGAC TTTGAGGCAG CATTAGGTAC AGACAGGCAC TGCCAGCGGC

Elephant GCTTGAGGAC TTCAAGGCAG CCTCAGGTTC AGACAGGCGC TCCCAGCGTA

Frog GCTGGAAGAT TTTGAGGCTG TGGTTAGTGA AGAGAAGAGT GCTAAGAAGA

Zebrafish GGTGGAGGAG TTTGAGGCCG TTCCTTTCAT GAACACCAGC ACGACTGCAC

Chicken GAGTGAAGAT TTTGAGACGT CGTGTAGCGA GGAAAGGTTT GGTAAAGCCC

Fugu CCCTGGTTTC TTCTCACTTG TACAACCAGC ACAAACTCGG ----------

Zebra_Finch GAGCGAGGAC ACAGAGACCT CGTGTTCCGA GCACAGGTTT GGAAAAGCCC

Human CGGATGCCTA CAGCAGGTCG TCCTCAGTCT CCAGCCAGGA GGAGAAGTCG

Gorilla CGGATGCCTA CAGCAGGTCG TCCTCAGTCT CCAGCCAGGA GGAGAAGTCG

Chimpanzee CAGACGCCTA CAGCAGGTCG TCCTCAGTCT CCAGCCAGGA GGAGAAGTCG

Orangutan CGGATGCCTA CAGCAGGTCG TCCTCAGTCT CCAGCCAGGA GGAGAAGTCG

Marmoset CGGATGCTTA CAGCCGGTCT TCCTCAACCT CCAGCCAGGA GGAGAAGTCA

Cow GCGACGCTTT CAGCAGGTCA TCTTCCACCT CCAGCCAGGA GGAGAAGTCA

Dog CGGAAGCTTA CAGCAGGTCG TCTTCAAGCT CCAGCCAGGA GGAGAAG---

Guinea_Pig CTGACACTTA CAGCAGGTCA TCCTCAGCCT CCAGCCAGGA AGAAAAGTTG

Horse CTGAAGCTTA CAGCAGGTCG TCTTCAACCT CCAGCCAGGA GGAGAAGTCA

Mouse CTGACACCTA CAGCCGGTCA TCCTCAGCAT CTAGCCAGGA AGAAAAGTCC

Pig CCGAAGCTTT CAGCAGGTCG TCCTCAACCT CCAGCCAGGA GGAGAAGCTG

Rabbit ---------- ---------- ---------- ---------- ----------

Rat CTGACGCCTA CAGCAGGTCT TCCTCAGCAT CTAGCCAGGA AGAAAAGTCC

Elephant CCGATGCTTA CAGTAGGTCA TCTTCGATCT CCAGCCAAGA GGAGAAGCAG

Frog TGGAGTGCAT TAGCAGGTCT TCATCCACAT CCAGTCAGGA AGAGGCTGCT

Zebrafish CCGGCATGAT GAGCAGATCC TCCTCCACAT CCAGTCAGGA TGATGATAAG

Chicken AGGAGTTTTA CAGCAGGTCG TCCTCGACCT CTAGCCAAGA TGAAAAATCT

Fugu -----ATGCA TTCAGTCTCT TCCTCAACCT CCAGTCAGGA CGAAGAAAAG

Zebra_Finch AGGACTCGTA CAGCCGGTCC TCCTCCACGT CCAGCCAGGA GGAGAAGTCG

Human CTCCACGCGG AGGAGCTGGT TGGCAGGGGC ATCCCCATCG AGCGAGTCGT

Gorilla CTCCACGCGG AGGAGCTGGT TGGCAGGGGC ATCCCCATCG AGCGCGCCGT

Chimpanzee CTCCACGCGG AGGAGCTGGT TGGCAGGGGC ATCCCCATCG AGCGAGCCGT

Orangutan CTCCACGCGG AGGAGCTGGT TGCCAGGGGC ATCCCCATCG AGCGAGCCGT

Marmoset TTCCACGCAG AGGAGCTGGC C---CGGGGT ATCCCCATCG AGCGCGCCGT

Cow TTCCATGCCG AGGAGTTGCC TCCTGGGGGC ATCCCCATCG AGCGGGCGGT

Dog TTCCCTGCAG AGGAGCTGGC TGCCGGAGGG ATCCCCATCG AACGAGCTGT

Guinea_Pig TCTCACGTGG AAGAGCTGGC TGTGGGAGGC ATACCCATCG AGCGAGCTGT

Horse TTCCGTGGAG AGGAGTTGGC TGTTGGGGGG ATCCCCATCG AGCGGGCCGT

Mouse ---CATTTGG AGGAGCTTGC TGCAGGGGGT ATCCCCATTG AGCGGGCCAT

Pig TTCCACGCAG AGGACTTGGC AGCCGGGGGC ATCCCCATCG AGCGGGCCGT

Rabbit ---------- ---------- ---------- ---------- ----------

Rat ---CACTTGG AGGAGCTGGC TGCAGGGGGT ATCCCCATTG AGCGGGCCAT

Elephant CTGTGTGCAG AGGAGCTGGC TGCCAGGGGA ATCCCCATTG AGCAGGTGGT

Frog ACCCAAACGG AGGAATCCGA GGCTGGTGGA ATACCCATTG AGGGCTCCAC

Zebrafish TCCACCCTGG AGGAGGTCAG TGAAGGAGGA ATCCCCATCG ACCAGCCTCC

Chicken CTGAAGTCGG ACGAGCTGGT GGAGGGCGGT ATTCCCATCG GGAGAGTGCT

Fugu TCAACACTGG AGGAAGTGAG CGAGGGCGCA ATTCCCATTG ATAACCCCAA

Zebra_Finch CTGCGGTCGG AGGAGCTGGC GGAGGGCGGG ATCCCCATCG GGCGG-----

Human CTCCTCGGAG GGTGGCCGGC CCTCTGTGGA CCTCTCCTTC CAGCCCTCGC

Gorilla CTCCTCGGAG GGTGGCCGGC CCTCTGTGGA CCTCTCCTTC CAGCCCTCGC

Chimpanzee CTCCTCAGAG GGTGGCCGGC CCTCTGTGGA CCTCTCCTTC CAGCCCTCGC

Orangutan CTCCTCGGAG GGTGGCCGGC CCTCTGTGGA CCTCTCCTTC CAGCCCTCGC

Marmoset CTCCTCCGAG GGTGGCCGGC CCTCCGTGGA CCTCCCCTTC CAGCCTTCGC

Cow CTCG---GAG GGCTCCCGGG CCTCCGTGGA CCTCGCCTTC CAGCCCTCTC

Dog CTCTTCCGAG GGTGCCCGGC CTTCTGTGGA GCTCTCCTTC CAGCCCTCCC

Guinea_Pig CTCTTCTGAG GGTGGCGGGC CCTCCGTGGA CCTCTCCTTT CAGCCCTCAC

Horse CTCCTCTGAG GGAGCCCGGC CCTCTGTGGA CCTCTCCTTC CAGCCCTCAC

Mouse CTCCTCTGAG GGGGCTCGGC CTGCCGTGGA CCTCTCCTTC CAACCCTCAC

Pig CTCCTCGGAG GGCTCCCGGT CCTCTGTGGA CCTCACCTTC CAGCCCTCAC

Rabbit ---------- ---------- ---------- ---------- ----------

Rat CTCCTCTGAG GGGGCTCGGC CTACAGTGGA CCTGTCCTTC CAACCCTCAC

Elephant GTCCTCCAGG GGTGGCCAGC CCTGTGTGGA CCTCTCCTTT CAGCCCTCGC

Frog TCGG------ ---------- ---------- -------GAG GTCCCATTTC

Zebrafish GCTCAGCCTC TGCACCCCTG GAGCCCCCGA GCTTTCCTTC ACCCAAAGCA

Chicken GCCAGCAGAG GATGGCCGGG CACTGGAGGA GCTCTCCTTC CAGCCCTCCC

Fugu CCTTGGACCC TCCACCCCAG GCAGTCCTGA GCTCCCCCTC CAGACCCACA

Zebra_Finch -------GAG GAAGGGCGGG CGCTGGAGGA GCTCTCCTTC CAGCCCTCAC

Human AGCCCCTGAG CAAGTCCAGC TCCTCTCCCG AGCTGCAGAC TCTGCAGGAC

Gorilla AGCCCCTGAG CAAGTCCAGC TCCTCTCCCG AGCTGCAGAC TCTGCAGGAC

Chimpanzee AGCCCCTGAG CAAGTCCAGC TCCTCTCCCG AGCTGCAGAC TCTGCAGGAC

Orangutan AGCCCCTGAG CAAGTCCAGC TCCTCTCCCG AGCTGCAGAC CCTGCAGGAC

Marmoset AGCCCCTGAG CAAGTCCAGC TCCTCTCCTG AGCTGCAGAC GCTGCAGGAC

Cow AGCCCCTGAG CAAGTCCAGC TCGTCGCCTG AACTGCAGAC CCTGCAGGAC

Dog AGCCCCTCAG CAAGTCCAGC TCCTCCCCTG AGCTGCAGAC CCTGCAGGAC

Guinea_Pig AGCCACTGAG CAAGTCCAGC TCCTCTCCTG AGCTACAGAC CCTGCAGGAC

Horse AGCCCCTAAG CAAGTCCAGC TCCTCACCTG AACTGCAGAC CCTGCAGGAC

Mouse AGCCTTTGAG CAAGTCTAGC TCTTCTCCGG AGTTGCAGAC CTTACAGGAC

Pig AGCCCCTGAG CAAGTCCAGC TCATCGCCTG AACTGCAGAC CCTGCAGGAT

Rabbit ---------- ---------- ---------- ---------- ----------

Rat AGCCTTTGAG CAAGTCTAGC TCTTCTCCTG AGTTACAGAC CTTACAGGAC

Elephant AGCCTCTAAG CAAATCCAGC TCCTCACCCG AACTGCAGAC CTTGCAGGAC

Frog AGACTTTGAG CAAGTCCAGC TCATCTCCTG AACTTCAGAC CTTGCAGGAG

Zebrafish GCAGCCTTAA TAAATCCAGC TCCTCTCCTG AGCTCCAGAC CCTTCCAGAG

Chicken AGCCGCTCAG CAAATCGAGC TCTTCCCCCG AGCTGCAGAC TTTGCAGGAG

Fugu CCCAGCTGAA CAAGTCCAGC TCTTCTCCTG AGCTCCAAAC CTTACCTGAG

Zebra_Finch AGCCCCTCAG CAAGTCCAGC TCCTCCCCGG AGCTCCAGAC GCTGCAGGAG

Human ATCCTCGGGG ACCCTGGGGA CAAGGCCGAC GTGGGCCGGC TGAGCCCTGA

Gorilla ATTCTCGGGG ACCCTGGGGA CAAGGCCGAC GTGGGCCGGC TGAGCCCCGA

Chimpanzee ATCCTTGGGG ACCCTGGGGA CAAGGCCGAC GTGGGCCGGC TGAGCCCTGA

Orangutan ATCCTCGGGG ACCCTGGGGA CAAGGCCGAC GTGGGCCGGC TGAGCCCTGA

Marmoset ATCCTCGGCG ACCCGGGGGA CAAGGCCGAA GTAGGCCGGC TGAGCCCTGA

Cow ATCCTGGGGG ACCCCGGGGA CAAGGCTGAG GTTGGCCGGC TGAGCCCCGA

Dog ATACTCGGGG ACCCCGGGGA CAAGTCTGAT GTTGGCCGGC TCAGCCCTGA

Guinea_Pig ATCCTTGGAG AACCTGGGGA CAAGGCTGAT GTTGGCCAGT TGAGTCCTGA

Horse ATCCTTGGGG ACCCTGGGGA CAAGGCTGAC GTTGGCCGGC TGAGCCCCGA

Mouse ATCCTTGGAG ACCTAGGGGA CAAGATTGAT ATTGGACGGC TGAGTCCTGA

Pig GTCCTTGGGG ACCTTGGGGA CAAGGCCGAT GCCGGCCGAC TGAGCCCTGA

Rabbit ---------- ---------- ---------- ---------- ----------

Rat ATCCTTGGAG ACCTAGGGGA CAAGACTGAC ATTGGACGGC TGAGTCCTGA

Elephant ATCTTTGGGG ACGCTGGGGA CAAGGCCGAT GGAGGCTGGC TGAGCCCTGA

Frog CTTCCTAAAG AGTTGGGCCC TACAGAAGAT ATAAAGAAGC CTTCTGCAGA

Zebrafish GCCTTCAGCA AAGCCTCGTC CCAGAGCGTG GATCCAGCTC CCACCAGCAA

Chicken ATCCTCAAGG ATGCAAATGG CAGAGAAGTA ACAAGGAGGC TGAGCACGGA

Fugu GCCTTCTCCA AAGCAGCCAT GGAGTCTGAG TCCGCTACTG GAGATGTGTC

Zebra_Finch GTGCTCAAGG ACGCCAACGG CCGCGAGGTG CCCCGGCGGC TGAGCACGGA

Human GGTTAAGGCC CGGTCACAGT CAGGGACCCT GGACGGGGAA AGTGCTGCCT

Gorilla GGTTAAGGCC CGGTCACAGT CAGGGACCCT GGACGGGGAA AGTGCTGCCT

Chimpanzee GGTTAAGGCC CGGTCACAGT CAGGGACCCT GGACGGGGAA AGTGCTGCCT

Orangutan GGTTAAGGCC CGGTCACAGT CAGGGACCCT GGACGGGGAA AGTGCTGCCT

Marmoset GGTTAAGGCC CGGTCACAGT CAGGGATCCT GGATGGGGAC AGTGCTACCT

Cow GGCCAAGGCC CGGTCGCAGT CAGGGATCCT GGACGGGGAA GGTGCCGCCT

Dog GGCCAAGGCC CGGTCACAGT CCGGGATTCT GGACGGGGTG GGTGCCTCCT

Guinea_Pig GGCCAAAGCC CGATCCCAGT CAGGAATATT GGATGGAGAA GGTGCCACCT

Horse GGTCAAGGCC CGGTCACAGT CTGGGATCCT GGATGGGGAA GGCGCTGCTT

Mouse GGCTAAGGTC CGGTCCCAGT CAGGGATCCT GGATGGGGAA GCAGCTACCT

Pig GGCCAAGGCC CGGTCGCAGT CAGGGATCCT GGATGGGGAA GGCGCTGCCT

Rabbit ---------- ---------- ---------- ---------- ----------

Rat GGCTAAGGTC CGATCCCAGT CGGGGATCCT GGATGGGGAA GCAGCTACCT

Elephant GGCCAAGGCC CGGTGGCAGT CGGGGACCCT GGATGGGGAG TGTGCTGCCT

Frog CTTCAAAACA CTACCCCATG TAGAAAACCC TTTAAGCCAC AGAACTGAGG

Zebrafish AATCCCTACA GTCCAGGCCT GTGTGGAGGC TCCAGGAGAG AGCGCTAGCA

Chicken AGTGAAGTCC AAATCTCAGT CTGGGAACCT GGAAGGGGAA GGACTGAGTT

Fugu ATGGTCCAGA ACACCATCAG AGGGCAAGCC CCCACCCCCG CCACCCATAA

Zebra_Finch CGTGAAATCC AAATCGCAGT CGGGGAATCT GGAAGGGGAA GGGCTGAGCT

Human GGTCGGCCTC GGGCGAAGAC AGTCGGGGCC AG-------- ----CCCGAG

Gorilla GGTCGGCCTC GGGCGAAGAC AGTCGGGGCC GG-------- ----CCCGAG

Chimpanzee GGTCGGCCTC GGGCGAAGAC AGTCGGGGCC AG-------- ----CCCGAG

Orangutan GGTCAGCCTC GGGCGAAGAC AGTCGGGGCC AG-------- ----CCGGAG

Marmoset GGTCAGGCCC AGGAGAAGAC AGCCGGGGCC AG-------- ----CCCGAG

Cow GGTCAGCCCC GGGTGAGGAA CGCCGGGGTC GGGGCCCTGC CCAGCCAGAG

Dog GGTCAGCCCC AGGTGAAGAG AACCAGGGCC AGGGCCCCGC ACAGCCCGAG

Guinea_Pig GGTCAGCTGC AGGTGAAGAG AGCCGGGGTA CAGCTCCA-- ----CCCGAA

Horse GGTCAGCCCC AGGCGAAGAG AGCCAGGGCC AGAGCCCTGC TCAGCCTGAG

Mouse GGTCAGCTAC GGGTGAAGAG AGCCGTATCA CAGTCCCA-- ----CCTGAA

Pig GGCCAGCTCC GGGCGAAGAG GGCCGGGGCC GGGGTCCTTC CCAGACCGAG

Rabbit ---------- ---------- ---------- ---------- ----------

Rat GGTCAGCTCC GGGTGAAGAG AGCCGTATCA CAGTCCCA-- ----CCTGAA

Elephant GGCTGGCTCC AGGTGAGGAG GGCCGGGCCC ATGCCCCAGC CCAGCACGAG

Frog AACGCAGTGC TGGGGCATTA GCCAAACCAG AAGGCGAAAT GCGACTTGAA

Zebrafish GCTCTAGCGT GTCCGTGTCC TCCTCCTCCA CCTCCAGAAT GAGGCTGGAG

Chicken GGCTGAACAA GGGTGAGGAT GCCAGAGCAG CAGGCTCGGG GGGGTTGGAT

Fugu CAGACGCTAA TGGACTCGAG GGTCACGGGC AAGGA----- ----AGTGAA

Zebra_Finch GGCTGGGCAG GGCGGAGGAC GGCAGGGCAG CCGGGCCGGG C------GCT

Human GGTCCCTTGC CTTCCAGCTC CCCCCGCTCG CCCAGTGGCC GGCCCCGAGG

Gorilla GGTCCCTTGC CTTCCAGCTC CCCCCGCTCG CCCAGTGGCC GGCCCCGAGG

Chimpanzee GGTCCCTTGC CTTCCAGCTC CCCCCGCTCG CCCAGTGGCC GGCCCCGAGG

Orangutan GGTCCCTTGC CTTCCAGCTC CCCCCGCTCG CCCAGTGGCC GGCCCCGAGG

Marmoset GGTCCCTTGC CTTCCAGCTC CCCCCGCTCG CCCAGTGGCC GGCCCCGAGG

Cow GGCCCCCTGC CTTCCAGCTG TCCTCGCTCC CCCAGCGGGC GGCCCCGAGG

Dog GGTCCCTTGC CTTCCAGCTG CCCCCGCTCC CCCAGTGGCC GGCCCAGAGG

Guinea_Pig GGCCCCTTGC CATCCAGCTC TCCCCGCTCT CCTAGTGGCC GCCCCCGTGG

Horse AGTCCCTTGC CTTCCAGCTG CCCCCGCTCC CCCAGTGGCC GGCCCCGAGG

Mouse GGTCCTCTGC CTTCCAGTTC TCCCCGCTCC CCCAGTGGCC GGCCCCGAGG

Pig GGCCCCTTAC CTTCCAGCTG CCCCCGCTCC CCCAGTGGCC GGCCTCGAGG

Rabbit ---------- ---------- ---------- ---------- ----------

Rat GGTCCCCTGC CTTCCAGTTC TCCCCGCTCC CCTAGTGGCC GGCCCCGAGG

Elephant GGCTCACTGC CCTGCAGCTC CCCACGCTCC CCACACGGCC GGCCCCGAGG

Frog CCAGAGTTGC CTGTTCTGTC CCCACAATCT CCAACTGGGC GCCCAAGGGG

Zebrafish TTCCCGCCAG TGCAGACAGG ACCCCTGTCT CCTACTGGAC GGCCACGGGG

Chicken GGCAGTGGCC CCACCACCTC GCCACGCTCC CCATCTGGGC GGCCCCGGGG

Fugu GGCCCAGCTG CAGGAGCAGG ACCAATCTCC CCCAGCGGGC GACCCCGGGG

Zebra_Finch GGCAGCGCGG CCGCCACCTC GCCCCGCTCG CCCTCGGGCC GGCCCCGCGG

Human TTACACCATC TCCGACTCGG CCCCATCACG CAGGGGCAAG AGAGTAGAGA

Gorilla TTACACCATC TCCGACTCGG CCCCATCACG CAGGGGCAAG AGGGTAGAGA

Chimpanzee TTACACCATC TCCGACTCGG CCCCATCACG CAGGGGCAAG AGAGTAGAGA

Orangutan TTACACCATC TCCGACTCGG CCCCATCACG GAGGGGCAAG AGGGTAGAGA

Marmoset ATACACCATC TCGGACTCGG CCCCATCACG CAGGGGCAAG AGGGTAGAGA

Cow CTACACCATC TCGGATTCGG CCCCGTCACG CAGGGGCAAG AGGGTGGAGA

Dog CTACACCATC TCTGATTCAG CCCCGTCACG CAGAGGCAAG AGGGTAGAGC

Guinea_Pig CTACACCATC TCGGACTCCG CCCCATCACG CAGGGGCAAG AGAGTAGAGA

Horse CTACACCATC TCTGATTCAG CCCCTTCACG CAGGGGCAAG AGGGTGGAGA

Mouse CTATACCATC TCTGATTCCG CTCCATCACG AAGGGGAAAG AGGGTAGAAA

Pig CTACACCATC TCTGATTCGG CCCCGTCGCG CAGGGGCAAG CGGGTGGAGA

Rabbit ---------- ---------- ---------- ---------- ----------

Rat CTATACCATC TCTGATTCAG CTCCATCACG AAGGGGAAAG AGGGTAGAAA

Elephant CTACACCATT TCCGACCCAG TCCAGTTGCG CAGGGGCAAG ACAGTGGAGA

Frog TCACACTATC TCTGATTCTG AGCCGTCCCG CAGAGGCAGG AGATTTCAGG

Zebrafish ACACACCATA TCAGTGTCTG CTCCATCGTC CAGGAGAGAC CGCAAAACAC

Chicken GTACACCATC TCTGACTCTG CCCCATCACG GAGAGGGAAG AGGATGGAGA

Fugu GCACACAATC TCAGTATCGG CCCCCTCCAG AAGGGAGAGG AGGACAGACA

Zebra_Finch CTACACCATC TCCGACTCCG CGCCCTCCAG GAGGGGCAAG AGGATCGACA

Human GGGACGCCTT AAAGAGCAGA GCCACAGCCT CCAATGCAGA GAAAGTGCCA

Gorilla GGGATGCCTT AAAGAGCAGA GCCACAGCCT CTAATGCAGA GAAAGTGCCG

Chimpanzee GGGATGCCTT AAAGAGCAGA GCCACAGCCT CCAATGCAGA GAAAGTGCCG

Orangutan GGGATGCCTT TAAGAGCAGA GCCACAGCCT CCAATGCAGA GAAAGTGCCG

Marmoset GGGACGCGTT TAAGAGCAGA GCCGCGGCCT CCAGTGCAGA GAAAGTGCCA

Cow GGGATGCCTT CAAGAACCGA GCAGGGACCT CCAACACCGA GAAGGTGCCA

Dog GGGACGCCTT CAGGAGCAGA GCTGGGGCTT CCAACACTGA GAAGGTGCCA

Guinea_Pig GGGATGCTTT CAGAAACAGA GCTGCCACTT CCAATACTGA GAAAGTCCCG

Horse GGGAAGCCTT CAAGAGCAGA GCTGGGGCCT CTAATACTGA GAAGGTGCCG

Mouse GGGATAACTT CAAGAGCAGA GCTGCGGCCT CCAGTGCTGA GAAGGTGCCA

Pig GGGATGCCTT CAAGGGCAGA GCCAGGACCC CCAACACGGA GAAGGTGCCG

Rabbit ---------- ---------- ---------- ---------- ----------

Rat GGGACAACTT CAAGAGCAGA ACTGCGGCCT CCAGTGCTGA AAAGGTGCCA

Elephant GAGACACCTC CAACAGCCGA GCTGCTGCCT CCAACGCGGA GAAGGTGCCA

Frog TTGATCCTTT TAAGAACAGC ACCAAATCCA CCAAAGCTGA GAAAGTGCCT

Zebrafish TGGAGCGG-- -GACGCCAGA GGAGGAGCGA CCAATGTGGA GAAGAGCTCC

Chicken GAGATGCCTT CAAGAGCAGA ACAGCAGCCA CCAATGCTGA GAAAGTACCT

Fugu GAGACTCGTA CCACAGTCGA TCAGGGCCAG GCAACACGGA GAAGATCGCT

Zebra_Finch GAGACGCCTT CAAGGGCAGG GCAGCGGCGT CCAACGCGGA GAAAGTGCCT

Human GGCATCAACC CCAGTTTCGT GTTCCTGCAG CTCTACCATT CCCCCTTCTT

Gorilla GGCATCAACC CCAGTTTCGT GTTCCTGCAG CTCTACCATT CCCCCTTCTT

Chimpanzee GGCATCAACC CCAGTTTCGT GTTCCTGCAG CTCTACCATT CCCCCTTCTT

Orangutan GGCATCAACC CC-------- ---------- ---------- ----------

Marmoset GGCATCAACC CCAGTTTCGT GTTCCTGCAG CTCTACCATT CACCTTTCTT

Cow GGCATCAACC CCAGCTTCGT GTTCCTGCAG CTCTACCACT CACCGTTCTT

Dog GGCATCAATC CCAGCTTTGT GTTCCTGCAG CTCTACCACT CACCCTTCTT

Guinea_Pig GGCATCAACC CCAGCTTTGT GTTCCTGCAG CTCTACCATT CACCTTTCTT

Horse GGCATCAACC CCAGCTTCGT GTTCCTGCAG CTCTACCATT CGCCTTTCTT

Mouse GGCATCAACC CTAGCTTTGT GTTCCTACAG CTCTACCATT CGCCCTTTTT

Pig GGCATCAACC CCAGCTTCGT GTTCCTGCAG CTCTACCACT CCCCTTTCTT

Rabbit ---------- ---------- ---------- ---------- ----------

Rat GGCATCAACC CTAGCTTTGT GTTCCTACAG CTCTACCATT CACCCTTTTT

Elephant GGCATCAACC CCAGCTTTGT GTTCCTGCAG CTCTACCACT CGCCCTTCTT

Frog GGAATAGATC CAAGCTTTGT ATTTTTGCAG CTGTACCATT CCCCATTCTT

Zebrafish GGACTCAGCC CCAGTTTTGT CTTCCTGCAG CTTTATCACT CGCCGTTCTT

Chicken GGAATAAACC CAAGCTTTGT GTTTTTACAA TTGTACCATT CCCCGTTCTT

Fugu GGTCTCAGCC CCAGCTTTGT CTTCCTCCAG CTCTATCACT CCCCTTTCTT

Zebra_Finch GGGATAAACC CCAGCTTTGT GTTTTTACAG TTGTACCACT CCCCCTTCTT

Human TGGCGACGAG TCAAACAAGC CAATCCTGCT GCCCAATGAG TCATTTGAGC

Gorilla TGGCGACGAG TCAAACAAGC CAATCCTGCT TCCCAATGAG TCATTTGAGC

Chimpanzee TGGCGACGAG TCAAACAAGC CAATCCTGCT GCCCAATGAG TCATTTGAGC

Orangutan ---------- ---------- --------AG GCCCAATGAG TCATTTGAGC

Marmoset TGGAGACGAG TCCAACAAGC CCATCCTTCT GCCCAATGAG TCATTTGAGC

Cow TGGTGACGAG TCCAACAAGC CAATCCTTTT GCCTAACGAG TCTTTCGAAC

Dog TGGTGACGAG TCCAACAAGC CCATCCTTCT ACCCAATGAG TCCTTCGAGC

Guinea_Pig TGGGGATGAG TCCAACAAAC CCATCCTGTT GCCCAATGAG TCCTTTGAGC

Horse CGGCGACGAG TCCAACAAGC CAATCCTTTT GCCTAACGAG TCCTTCGAGC

Mouse TGGTGATGAG TCCAATAAGC CCATCCTATT GCCCAATGAG TCCTTTGAAC

Pig CGGTGACGAG TCCAACAAGC CCATCCTTCT GCCCAGCGAG TCTTTTGAGC

Rabbit ---------- ---------- ---------- ---------- ---TTCGAGC

Rat TGGTGATGAG TCCAATAAGC CCATCCTGTT GCCCAACGAG TCCTTTGAAC

Elephant TGGGGACGAG TCCAACAAGC CAATTCTGTT ACCCAACGAG TCCTTCGAGC

Frog TGGTGATGAG TCCAATAAGC CTATACTGGT CCCTAACACA ---TTTGAGA

Zebrafish CGGCAATGAG GCCAACAAAC CCCTGCTGCT TCCTAAATCA ---ATTGACC

Chicken TGGTGATGAA AACAACAAGC CCCTCCTGTT GCCAAATGAG ACGTTCGAAA

Fugu TGGAAATGAA GCCAACAAGC CGCTGCTTCT CCCTAAAACA ---ATCGACC

Zebra_Finch TGGTGATGAA AACAACAAAC CCCTCTTGTT GCCAAATGAG ACGTTTGAGA

Human GGTCGGTGCA GCTCCTCGAC CAGATCCCAT CATACGACAC CCACAAGATC

Gorilla GGTCGGTGCA GCTCCTCGAC CAGATCCCAT CATACGACAC CCACAAGATC

Chimpanzee GGTCGGTGCA GCTCCTCGAC CAGATCCCAT CATACGACAC CCACAAGATC

Orangutan GGTCGGTGCA GCTCCTCGAC CAGATCCCAT CATACGACAC CCACAAGATC

Marmoset GGTCGGTGCA GCTCCTCGAT CAGATCCCGT CGTATGACAC CCACAAGATC

Cow GATCAGTGCA GCTCCTTGAT CAGATCCCGT CGTACGACAC GCACAAGATC

Dog GGTCAGTGCA GCTTCTCGAC CAGATTCCAT CATACGACAC ACACAAGATT

Guinea_Pig GGTCAGTGCA GCTGCTTGAC CAGATCCCAT CCTACGACAC GCACAAGATT

Horse GATCGGTGCA GCTCCTTGAC CAGATCCCGT CCTACGACAC ACACAAGATT

Mouse GGTCAGTACA GCTGCTTGAC CAGATTCCAT CCTATGACAC TCACAAGATT

Pig GGTCGGTGCA GCTCCTCGAC CAGATCCCGT CATATGACAC GCACAAGATC

Rabbit GCTCGGTGCA GCTCCTGGAC CAGATTCCGT CCTACGACAC GCACAAGATC

Rat GGTCAGTGCA GCTCCTTGAC CAGATCCCAT CCTATGACAC TCACAAGATT

Elephant GCTCGGTGCA GCTCCTTGAC CAGATCCCTT CGTATGACAC ACACAAGATC

Frog GAACAATGGG CCTCCTAGAT CGAATCCCGC CCTATGACAC CCACAAAATT

Zebrafish GTGCAGTGAA GGTTTTGGAT CAGATGCCTC CGTACGACAC TCATAAGATC

Chicken AATCGGTGCA GCTCCTGGAT CAAATTCCTT CGTATGACAC CCACAAGATC

Fugu GTGCTGTGAA GGTTCTGGAC CAGATGCCTC CATACGACAC CCACAAGATC

Zebra_Finch GGTCAGTGCA GCTCCTGGAT CAGATCCCAT CCTATGACAC ACACAAGATT

Human GCCGTCCTGT ATGTTGGAGA AGGCCAGAGC AACAGCGAGC TCGCCATCCT

Gorilla GCTGTCCTGT ATGTTGGAGA AGGCCAGAGC AACAGCGAGC TCGCCATCCT

Chimpanzee GCCGTCCTCT ATGTTGGAGA AGGCCAGAGC AACAGCGAGC TCGCCATCCT

Orangutan GCCGTCCTGT ATGTTGGAGA AGGCCAGAGC AACAGCGAGC TCGCCATCCT

Marmoset GCCGTCCTGT ATGTGGGCGA AGGCCAGAGC AACAGCGAGC TCGCCATCCT

Cow GCGGTCCTGT ACGTGGGAGA AGGCCAGAGC AACAGCGAGC TCGCCATCCT

Dog GCCGTCCTGT ACGTGGGAGA AGGCCAGAGC AGCAGTGAGC TCGCCATCCT

Guinea_Pig GCTGTCCTGT ACGTGGGAGA AGGCCAGAGC AGCAGCGAGC TGGCCATCCT

Horse GCCGTCCTGT ACGTGGGAGA AGGCCAGAGC ACCAGTGAGC TCGCCATCCT

Mouse GCTGTCCTGT ATGTGGGAGA AGGCCAGAGC AGCAGTGAGC TGGCCATCCT

Pig GCCGTCCTGT ACGTGGGAGA AGGCCAGAGC AACAGTGAGC TTGCCATCCT

Rabbit GCCGTGCTGT ATGTGGGAGA GGGCCAGAGC AACAGTGAGC TGGCCATCCT

Rat GCTGTCCTGT ATGTGGGAGA AGGCCAGAGC AGCAGTGAGC TGGCCATCCT

Elephant GCTGTTTTGT ACGTGGGAGA GGGCCAGAGC AACAGTGAGC TCGCCATCCT

Frog GGTGTTCTTT ATGTTGGAGA GGGGCAGGTT GGCAACGAGC GCGCAATCCT

Zebrafish GGTGTGGTGT TTGTGGGAGC CGGACAGGCC AATAATGAGG TGTCGATCCT

Chicken GCAGTGCTGT ACGTTGGAGA GGGCCAGAGC AACAATGAAA TCGCTATTCT

Fugu GGCGTCGTGT TTGTAGGCGC TGGTCAGGTC AACAACGAGG TCGCCATTCT

Zebra_Finch GCAGTGCTCT ACGTTGGGGA GGGCCAGAGC AACAACGAGA TCGCCATCCT

Human GTCCAATGAG CATGGCTCCT ACAGGTACAC GGAGTTCCTG ACGGGCCTGG

Gorilla GTCCAACGAG CATGGCTCCT ACAGGTACAC GGAGTTCCTG ACGGGCCTGG

Chimpanzee GTCCAACGAG CATGGCTCCT ACAGGTACAC GGAGTTCCTG ACGGGCCTGG

Orangutan GTCCAACGAG CATGGCTCCT ACAGGTACAC GGAGTTCCTG ACGGGCCTGG

Marmoset GTCCAACGAG CATGGCTCCT ACAGGTACAC AGAGTTCCTG ACGGGCCTGG

Cow GTCCAACGAG CATGGCTCCT ACAGGTACAC AGAGTTCCTC ACGGGGCTGG

Dog GTCCAATGAG CATGGCTCGT ACAGGTACAC GGAGTTCCTG ACAGGCCTGG

Guinea_Pig CTCCAATGAG CATGGCTCCT ATAGGTACAC AGAGTTCCTG ACGGGCCTGG

Horse GTCCAATGAG CACGGCTCCT ACAGGTACAC GGAATTCCTG ACAGGCCTGG

Mouse GTCAAATGAG CATGGCTCTT ACAGGTACAC GGAGTTTCTG ACAGGCCTGG

Pig GTCCAACGAG CACGGCTCCT ACAGGTACAC GGAGTTCCTG ACGGGGCTGG

Rabbit GTCCAACGAG CACGGCTCCT ACCGGTACGC GGAGTTCCTG ACGGGGCTCG

Rat ATCAAATGAG CATGGCTCTT ATAGGTACAC AGAGTTTCTG ACAGGCCTGG

Elephant CTCCAACGAG CACGGCTCCT ACAGGTATAC AGAATTCCTG ACGGGCCTGG

Frog ATCCAATGAG CATGGCTCGT ACCGCTACAC ACAGTTCCTC ACGGGGCTTG

Zebrafish GTCTAATGAG TACGGCTCGA AGCGCTATGC TCAGTTCCTG ACGGGTCTGG

Chicken GTCCAATGAA CACGGCTCCT ATCGCTACAC CGAGTTCCTG ACAGGCTTAG

Fugu ATCAAACGAG TACGGGTCAA ACCGCTACGC TGCCTTCCTG ACAGGACTGG

Zebra_Finch GTCCAACGAG CACGGCTCCT ACCGCTACAC CGAGTTCCTC ACGGGCCTGG

Human GCCGGCTCAT CGAGCTGAAG GACTGCCAGC CGGACAAGGT GTACCTGGGA

Gorilla GCCGGCTCAT CGAGCTGAAG GACTGCCAGC CGGACAAGGT GTACCTGGGA

Chimpanzee GCCGGCTCAT CGAGCTGAAG GACTGCCAGC CGGACAAGGT GTACCTGGGA

Orangutan GCCGGCTCAT CGAGCTGAAA GACTGCCAGC CCGACAAGGT GTACCTGGGA

Marmoset GCCGGCTCAT CGAGCTGAAG GACTGCCAGC CAGACAAGGT GTACCTGGGA

Cow GCAAGCTCAT CGAGCTCAAG GACTGCCAGC CGGACAAGGT GTACCTGGGC

Dog GCAAGCTCAT TGAGCTCAAA GACTGCCAGC CGGACAAGGT GTACCTGGGG

Guinea_Pig GCAGGCTCAT TGAGCTCAAG GATTGCCAGC CTGACAAGGT GTACCTGGGT

Horse GCAAGCTCAT CGAGCTCAAG GACTGCCAGC CAGATAAGGT GTACCTGGGC

Mouse GTCGGCTTAT TGAGCTCAAG GACTGCCAAC CAGACAAGGT GTACTTAGGT

Pig GCAAGCTCAT CGAGCTCAAG GACTGCCAGC CGGACAAGGT GTACCTGGGT

Rabbit GCAAGCTCAT CGAACTCAAG GACTGCCAGC CCGACAAGGT GTACCTGGGC

Rat GTCGGCTTAT TGAGCTGAAG GACTGCCAAC CAGACAAGGT GTACTTAGGT

Elephant GCAAGCTCAT TGAGCTCAAG GACTGCCAGC CTGACAAGGT GTACCTGGGT

Frog GGAAGCTCAT CGAGCTGAAA GACACTCAGC CAGACAAGAT CTTCCTGGGA

Zebrafish GGAAACTCAT CCACTTGAAA GACTGCGATC CCGATCAGAT CTTCCTTGGT

Chicken GGAAGCTCAT TGAGCTCAAA GACTGCCAGC CAGATAAAAT CTACCTCGGT

Fugu GCAAATTAAT CCACTTGAAA GACTGCGACC CTGACCAGAT CTTCCTCGGA

Zebra_Finch GCAAGCTCAT CGAGCTCAAG GACTGCCAGC CAGACAAGAT CTACCTGGGT

Human GGCCTGGACG TGTGTGGTGA GGACGGCCAG TTCACCTACT GCTGGCACGA

Gorilla GGCCTGGACG TGTGTGGTGA GGACGGCCAG TTCACCTACT GCTGGCACGA

Chimpanzee GGCCTGGACG TGTGTGGTGA GGACGGCCAG TTCACNTACT GCTGGCACGA

Orangutan GGCCTGGACG TGTGTGGTGA GGATGGTCAG TTCACCTACT GCTGGCACGA

Marmoset GGCCTGGATG TGTGTGGTGA GGACGGCCAG TTCACCTATT GCTGGCACGA

Cow GGCCTGGACG TGTGCGGCGA GGATGGCCAG TTCACCTACT GCTGGCACGA

Dog GGCCTGGACG TGTGTGGCGA AGATGGCCAG TTCACCTACT GCTGGCATGA

Guinea_Pig GGCCTTGACG TGTGTGGTGA GGATGGCCAA TTCACCTACT GCTGGCATGA

Horse GGCCTGGATG TGTGTGGCGA GGATGGCCAG TTCACCTACT GCTGGCACGA

Mouse GGACTGGATG TATGTGGCGA GGATGGACAG TTCACCTACT GCTGGCATGA

Pig GGCTTGGACG TGTGTGGCGA GGACGGCCAG TTCACCTACT GCTGGCACGA

Rabbit GGCCTGGACG TGTGCGGCGA GGACGGCCAG TTCACCTACT GCTGGCACGA

Rat GGACTGGATG TGTGTGGCGA GGATGGACAG TTCACGTACT GCTGGCATGA

Elephant GGCTTGGATG TGGGCGGCGA GGACGGCCAG TTCACCTACT GCTGGCATGA

Frog GGGTTGGACG GTTGTGGAGA TGACGGGCAG TTCACCTACT GCTGGCATGA

Zebrafish GGACTGGACC AGTACGGAGA CGATGGAGAG TTCACATACT GCTGGCATGA

Chicken GGCCTGGACG TGTGTGGGGA GGATGGACAG TTTACCTACT GCTGGCACGA

Fugu GGGCTGGACC AGTACGGCGA TGACGGAGAG TTCACCTACT GCTGGCACGA

Zebra_Finch GGCCTGGATG TGTGTGGGGA GGACGGACAG TTCACCTACT GCTGGCACGA

Human TGACATCATG CAAGCCGTCT TCCACATCGC CACCCTGATG CCCACCAAGG

Gorilla TGACATCATG CAAGCCGTCT TCCACATCGC CACCCTGATG CCCACCAAGG

Chimpanzee TGACATCATG CAAGCCGTCT TCCACATCGC CACCCTGATG CCCACCAAGG

Orangutan TGACATCATG CAT------- ---------- ---------- ----------

Marmoset CGACATCATG CAAGCCATCT TCCACATCGC CACCCTGATG CCCACCAAGG

Cow CGACATCATG CAAGCTGTCT TCCACATCGC CACCCTGATG CCCACCAAGG

Dog CGACATCATG CAAGCCGTCT TCCACATTGC TACCCTGATG CCCACCAAGG

Guinea_Pig TGACATAATG CAAGCTATCT TTCACATTGC CACCCTGATG CCTACCAAGG

Horse CGACATCATG CAAGCCGTCT TCCACATCGC CACCCTGATG CCCACCAAGG

Mouse TGACATCATG CAAGCTGTTT TCCACATTGC CACCCTGATG CCCACCAAGG

Pig CGACATCATG CAAGCCGTCT TCCACATCGC CACCCTGATG CCCACCAAGG

Rabbit CGACATCATG CAAGCCGTCT TCCACATCGC CACCCTGATG CCCACCAAGG

Rat TGACATCATG CAAGCTGTTT TCCACATTGC TACCCTGATG CCCACCAAGG

Elephant TGACATCATG CAAGCAGTCT TTCACATCGC CACCCTGATG CCCACCAAGG

Frog TGATATTATG CAAGTCATTT TTCAGATCAC CACCCTGATG CCAAACAAAG

Zebrafish TGACATTATG CAGGCTATTT TCCACATCGC CACGCTGATG CCGAACCGTG

Chicken TGACATCATG CAAGCTATTT TCCACATTGC TACACTGATG CCCACAAAGG

Fugu TGACATCATG CAGGCCATTT TCCACATCGC CACACTGATG CCAAACAGAG

Zebra_Finch TGACATCATG CAAGCCATTT TCCACATTGC TACTCTGATG CCCACAAAGG

Human ACGTGGACAA GCACCGCTGC GACAAGAAGC GCCACCTGGG CAACGACTTT

Gorilla ACGTGGACAA GCACCGCTGC GACAAGAAGC GCCACCTGGG CAACGACTTT

Chimpanzee ACGTGGACAA GCACCGCTGC GACAAGAAGC GCCACCTGGG CAACGACTTT

Orangutan ---------- ---------- ---------- ---------- ----------

Marmoset ATGTGGACAA GCACCGCTGT GACAAGAAGC GCCACTTGGG CAATGACTTT

Cow ACGTGGACAA GCACCGCTGT GACAAGAAGC GCCACCTGGG CAACGACTTC

Dog AAGTGGACAA GCACCGCTGT GACAAGAAGC GCCACCTGGG CAATGACTTT

Guinea_Pig ATGTGGACAA GCACCGCTGT GACAAGAAGC GGCACCTGGG TAATGACTTT

Horse ACGTGGACAA GCACCGTTGC GACAAGAAGC GCCACCTGGG CAATGATTTC

Mouse ATGTGGACAA GCACCGCTGT GACAAGAAAC GGCACCTGGG CAATGACTTT

Pig ACGTGGACAA GCACCGCTGT GACAAGAAGC GCCACCTGGG CAATGATTTC

Rabbit ACGTGGACAA GCACCGCTGT GACAAGAAGC GCCACCTGGG CAACGACTTC

Rat ATGTGGACAA GCACCGTTGT GACAAGAAAC GGCACCTGGG CAATGACTTT

Elephant ATGGGGACAA GCACCGCTGT GACAAGAAGC GCCACCTGGG CAATGACTTT

Frog AGATAGATCC GAACCGCTGC AACAAGAAAA GGCACGTGGG CAACAACTTT

Zebrafish AGAGCGACCG CGGCTGCTGC AACAAGAAGC GGCACATCGG CAACGACTTC

Chicken ATTTGGATAA ATATCGCTGT GACAAGAAGA GGCACCTTGG GAATGATTTT

Fugu AGAGCGACAA GGGATGTTGC AATAAAAAGC GACACATTGG CAACGATTTT

Zebra_Finch ATTTGGATAA ATACCGCTGT GACAAGAAGA GGCACCTGGG GAATGATTTT

Human GTGTCCATTG TCTACAATGA CTCCGGTGAG GACTTCAAGC TTGGCACCAT

Gorilla GTGTCCATTG TCTACAATGA CTCCGGTGAG GACTTTAAGC TCGGCACCAT

Chimpanzee GTGTCCATTG TCTACAATGA CTCTGGTGAG GACTTCAAGC TCGGCACCAT

Orangutan ---------- ---------- ---------- ---------- ----------

Marmoset GTGTCCATTG TCTACAATGA CTCCGGCGAG GACTTCAAGC TCGGTACCAT

Cow GTGTCCATCG TCTACAACGA CTCTGGCGAG GACTTCAAGT TGGGCACCAT

Dog GTGTCCATCG TCTACAACGA CTCTGGTGAG GACTTCAAGC TGGGCACCAT

Guinea_Pig GTTTCCATTA TCTACAACGA CTCTGGTGAG GAATTCAAGC TAGGCACCAT

Horse GTGTCCATTG TCTACAACGA CTCTGGCGAG GACTTCAAGC TGGGCACCAT

Mouse GTTTCTATCA TCTACAATGA TTCTGGTGAG GACTTCAAAC TGGGCACCAT

Pig GTGTCCATCG TCTACAACGA CTCGGGCGAG GACTTCAAGC TGGGCACCAT

Rabbit GTCTCCATCG TCTACAACGA CTCTGGCGAG GACTTCAAGC TGGGCACCAT

Rat GTTTCTATCA TCTACAATGA TTCTGGTGAG GACTTCAAGC TGGGCACCAT

Elephant GTGTCCATTG TCTACAACGA CTCTGGCGAG GACTTCAGGC TGGGCACCAT

Frog GTCAATATCA TCTACAACGA CTCGGGCGAG TTCTACAAGT TGGGCACCCT

Zebrafish GTGGTGGTGG TTTATAACGA CTCGGGGGAG GACTACAAAC TGGGCACCAT

Chicken GTTTCTATTG TTTACAATGA TTCTGGTGAG GACTTCAAAC TGGGAACAAT

Fugu GTTATTGTGG TTTACAATGA CTCTGGAGAG GAATACAAGT TGGGAACCAT

Zebra_Finch GTTTCCATTG TTTACAATGA TTCTGGAGAG GATTTTAAGT TGGGCACAAT

Human CAAGGGCCAG TTCAACTTTG TCCACGTGAT CGTCACCCCG CTGGACTACG

Gorilla CAAGGGCCAG TTCAACTTTG TCCACGTGAT CGTCACCCCG CTGGACTACG

Chimpanzee CAAGGGCCAG TTCAACTTTG TCCACGTGAT CGTCACCCCG CTGGACTACG

Orangutan ----GGCCAG TTCAACTTTG TCCACGTGAT CGTCACCCCG CTGGACTATG

Marmoset CAAGGGCCAG TTCAACTTTG TCCACGTGAT CATCACCCCG CTGGACTATG

Cow CAAGGGCCAA TTCAACTTCG TCCACGTGAT TATCACGCCC CTGGACTACG

Dog CCGAGGCCAG TTCAACTTCG TCCACGTGAT CATCACCCCC CTGGACTATG

Guinea_Pig CAAGGGTCAG TTCAACTTTG TTCATGTGAT CATCACACCC CTGGACTATG

Horse CAAAGGCCAG TTCAACTTTG TCCATGTGAT TATCACCCCC CTGGACTACG

Mouse TAAGGGCCAG TTCAACTTTG TCCATGTGAT CATCACACCG CTGGACTACA

Pig CAAAGGCCAG TTCAACTTTG TCCACGTGAT CATCACGCCC CTGGACTACG

Rabbit CAGAGGCCAG TTCAACTTTG TGCACGTGAT CATCACGCCA CTGGACTACG

Rat TAAGGGCCAG TTCAACTTCG TCCATGTGAT CATCACACCG CTGGACTATA

Elephant CAAGGGCCAG TTCAACTTCG TGCATGTGAT TATCACTCCT CTGGACTATG

Frog CAAGGGTCAG TTCAACTTTG TAGATGTCAA CATTCAGCCT TTGGACTGCG

Zebrafish CAAGGGTCAG TTCAACTTTG TGGAAGTGCT CATAAAGCCG CTGGACTACG

Chicken AAAGGGTCAG TTCAATTTTG TGCATGTTAT CATAACCCCA CTTGACTATG

Fugu TAAGGGGCAG TTTAACTTTG TAGAAGTCAT TATCAAACCG CTCGACTATG

Zebra_Finch AAAGGGGCAG TTCAATTTTG TCCATGTGAT CATCACCCCT CTGGACTACG

Human AGTGCAACCT GGTGTCCCTG CAGTGCAGGA AAGACATGGA GGGCCTTGTG

Gorilla AGTGCAACCT GGTGTCCTTG CAGTGCAGGA AAGACATGGA GGGCCTTGTG

Chimpanzee AGTGCAACCT GGTGTCCCTG CAGTGCAGGA AAGACATGGA GGGCCTTGTG

Orangutan AGTGCAACCT GGTGTCCCTG CAGTGCAGGA AAGACATGGA GGGCCTTGTG

Marmoset AGTGCAACCT GGTGTCGCTG CAGTGCAGGA ATGACATGGA GGGCCTTGTG

Cow AGTGCAACCT GGTGTCTCTG CAGTGCAGGA AAGACATGGA GGGCCTTGTG

Dog AGTGCAACCT GGTGTCCCTG CAGTGCAGGA AAGACATGGA AGGTCTTGTG

Guinea_Pig AGTGCAACCT GCTGTCACTG CAGTGCAGGA AAGACATGGA GGGCCTGGTG

Horse AGTGCAACCT GGTGTCGCTG CAGTGCAGGA AAGACATGGA GGGCCTCGTG

Mouse AGTGCAACCT ATTGACCCTG CAGTGCAGGA AAGACATGGA AGGCCTCGTG

Pig AGTGCAACCT GGTGTCGCTG CAGTGCAGGA AAGACATGGA GGGCCTCGTG

Rabbit AGTGCAACCT TGTGTCCCTG CAGTGCAGGA AGGACATGGA AGGCCTGGTG

Rat AATGCAACCT GTTGACCCTG CAGTGCAGGA AAGACATGGA GGGCCTTGTG

Elephant AGTGCAACCT GGTCTCACTC CAGTGCCGGA AAGACATGGA GGGCCTGGTG

Frog AGAGCAACCT GGTGACACTG CAATGCCGAA AAGATATGGA AGGCCTTGTA

Zebrafish AGAGTAACCT GGTGACACTG CAGTGCCGGA AAGATCTGGA GGGTTTGGTG

Chicken ACTGCAACCT GGTCACACTG CAGTGTCGAA AAGACATGGA GGGCCTTGTA

Fugu AATGTAATCT TGTGACTCTC CAGTGCCGCA AAGACCTCGA AGGTTTGGTG

Zebra_Finch ACTGTAACCT GGTGACGCTG CAGTGCCGGA AAGACATGGA GGGTCTGGTG

Human GACACCAGCG TGGCCAAGAT CGTGTCTGAC CGCAACCTGC CCTTCGTGGC

Gorilla GACACCAGCG TGGCCAAGAT CGTGTCTGAC CGCAACCTGC CCTTCGTGGC

Chimpanzee GACACCAGCG TGGCCAAGAT CGTGTCTGAC CGCAACCTGC CCTTCGTGGC

Orangutan GACACCAGCG TGGCCAAGAT CGTGTCTGAC CGCAACCTGC CCTTCGTGGC

Marmoset GACACCAGCG TGGCCAAGAT TGTGTCTGAC CGTAACCTGT CCTTTGTGGC

Cow GATACCAGCA TGGCCAAGAT CGTGTCTGAC CGAAACCTGC CCTTCGTGGC

Dog GATACTAGCG TGGCCAAGAT CGTGTCTGAC CGCAACTTGC CTTTTGTGGC

Guinea_Pig GATACAAGTG TGGCCAAGAT TGTGTCTGAT CGGAACCTGC CCTTTGTGGC

Horse GACACCAGCG TGGCCAAGAT CGTGTCTGAC CGCAACCTCC CTTTTGTGGC

Mouse GACACCAGTG TGGCCAAGAT TGTGTCTGAC CGCAACCTGT CCTTTGTGGC

Pig GACACCAGTG TGGCCAAGAT TGTGTCTGAC CGAAACCTGC CCTTCGTGGC

Rabbit GACACCAGCA TGGCCAAGAT CGTGTCTGAC CGCAACCTCC CGTTCGTGGC

Rat GACACCAGTG TGGCCAAGAT TGTGTCTGAC CGCAACCTGT CCTTTGTGGC

Elephant GACACCAGTA TGGCCAAGAT CGTGTCTGAC TGTAACCTGC CCTTCGTGGC

Frog GATACCAGCG TGGCAAAAAT TGTGTCTGAC AAAAACCTAC CCTTCCTGGC

Zebrafish GACACCAGTG TAGCGAAGAT CGTCTCTGAC CGGAACCTTC CCCTGCTAGT

Chicken GACACGAGTG TTGCTAAGAT CATCTCTGAC AAGAACCTGC CATTCGTGGC

Fugu GACACGACGG TAACAAAAAT AGTCTCGGAC CGGAACCTTC CCCTGTTGGT

Zebra_Finch GACACGAGTG TTGCCAAGAT CATCTCTGAC AAGAACCTGC CCTTCGTGGC

Human CCGCCAGATG GCCCTGCACG CAAATATGGC CTCACAGGTG CATCATAGCC

Gorilla CCGCCAGATG GCCCTGCACG CAAATATGGC CTCACAGGTG CATCATAGCC

Chimpanzee CCGCCAGATG GCCCTGCACG CAAATATGGC CTCACAGGTG CATCATAGCC

Orangutan CCGCCAGATG GCCCTGCACG CAAATATGGC CTCACAGGTG CACCATAGCC

Marmoset CCGCCAGATG GCCCTGCATG CAAATATGGC CTCACAGGTG CACCACAGCC

Cow CCGCCAGATG GCCCTGCACG CCAATATGGC CTCGCAGGTG CACCATAGCC

Dog ACGTCAGATG GCTTTGCATG CAAATATGGC CTCACAAGTG CATCATAGCC

Guinea_Pig CCGCCAGATG GCTCTACATG CAAATATGGC CTCGCAGGTG CACCACAGCC

Horse CCGCCAGATG GCCCTGCATG CAAATATGGC CTCGCAGGTA CACCACAGCC

Mouse CCGACAGATG GCCCTGCATG CAAATATGGC CTCACAGGTA CACCACAGCC

Pig CCGCCAGATG GCCCTGCATG CGAACATGGC CTCGCAGGTG CACCATAGCC

Rabbit CCGCCAGATG GCCCTGCATG CGAACATGGC CTCGCAGGTG CACCACAGCC

Rat CCGACAGATG GCCCTGCATG CAAATATGGC CTCACAGGTA CACCACAGCC

Elephant CCGCCAGATG GCCTTGCATG CCAACATGGC CTCCCAGGTC CACCACAGCC

Frog CCGCCAGATG GCTCTACATT CAAATATGGC TTCACAAGTC CATCACAGCA

Zebrafish CAGACAGATG GCGCTTCATG CTAACGTATA TTCCAATCTC CACAATCCCA

Chicken CCGTCAGATG GCCCTGCATG CTAACATGGC TTCCCAGGTG CACCACAGTC

Fugu CAGACAGATG GCTCTCCATG CAAACATGGC CTCTCTGGTG CATCAATTTA

Zebra_Finch CCGTCAGATG GCCCTGCATG CCAACATGGC TTCCCAGGTC CACCACAGCA

Human GCTCCAACCC CACCGATATC TACCCCTCCA AGTGGATTGC CCGGCTCCGC

Gorilla GCTCCAACCC CACCGACATC TACCCCTCCA AGTGGATTGC CCGGCTCCGC

Chimpanzee GCTCCAACCC CACCGACATC TACCCCTCCA AGTGGATTGC CCGGCTCCGC

Orangutan GCTCCAACCC CACCGACATC TACCCCTCCA AGTGGATTGC CCGGCTCCGC

Marmoset GCTCCAACCC CACTGACATC TACCCCTCCA AGTGGATCGC CCGGCTCCGC

Cow GCTCCAATCC CACCGACATT TATCCCTCCA AGTGGATTGC CAGGCTCCGC

Dog GCTCCAACCC CACCGACATC TATCCCTCCA AGTGGATTGC CAGGCTTCGC

Guinea_Pig GTTCTAACCC TACGGACATC TACCCCTCCA AGTGGATTGC TCGGCTTCGC

Horse GCTCCAACCC CACTGACATC TATCCCTCCA AGTGGATTGC CAGGCTCCGC

Mouse GATCCAACCC CACTGACATC TATCCCTCCA AGTGGATCGC AAGACTCCGC

Pig GATCCAACCC CACGGACATC TACCCCTCCA AGTGGATCGC CAGGCTCCGC

Rabbit GCTCCAACCC CACCGACATC TACCCCTCCA AGTGGATCGC CCGGCTGCGG

Rat GATCCAACCC CACGGACATC TATCCCTCCA AGTGGATCGC AAGACTCCGC

Elephant GCTCTAACCC CACTGACATC TACCCTTCCA AGTGGATTGC CCGGCTCCGC

Frog GGTCCAACCC CACAGATATT TACCCTTCTA AATGGATTGC CAGGCTGCGA

Zebrafish GAGCGAACCC ATCAGACGCA TACGCTTCAA AATGGCTGGC CAGACTGAGA

Chicken GATCCAACCC TACTGACACC TACCCATCCA AATGGATCGC TCGGCTGCGG

Fugu GAGCCAACCC CTCCGACGCA TACGCTTCCA AGTGGCTGGC CAGACTGCGA

Zebra_Finch GATCCAACCC CACGGACACC TACCCCTCCA AATGGATCGC GAGGCTGCGC

Human CACATCAAGC GGCTCCGCCA GCGGATCTGC GAGGAAGCCG CCTACTCCAA

Gorilla CACATCAAGA GGCTCCGCCA GCGGATCTGC GAGGAAGCCG CCTACTCCAA

Chimpanzee CACATCAAGA GGCTCCGCCA GCGGATCTGC GAGGAAGCCG CCTACTCCAA

Orangutan CACATCAAGA GGCTCCGCCA GCGGATCTGT GAGGAAGCCG CCTACTCCAA

Marmoset CACATCAAGA GGCTCCGCCA GCGGATCTGC GAGGAAGCCC CGTACTCCAA

Cow CACATCAAGC GGCTGCGCCA GCGGATCCGT GAGGAAGCCC ACTACTCAAG

Dog CACATCAAGC GACTCCGCCA CCGGATCCGC GAGGAAGCCC ACTACACCAA

Guinea_Pig CACATCAAGC GGCTCCGTCA GCGGATCCGT GAGGAAGCCC ACTACTCAAA

Horse CACATCAAGC GGCTCCGCCA CCGGATCCGG GAGGAAGCCC AGTACTCGAA

Mouse CACATTAAGC GTCTCCGCCA GAGGATCCGT GAAGAGGTGC ACTATTCCAA

Pig CACATCAAGC GGCTGCGCCA ACGGATCCGA GAGGAGGCCC ACTACTCGAA

Rabbit CACATCAAGC GGCTTCGCCA GCGGATCCGC GAGGCGGCCC AGTACTCCAA

Rat CACATTAAGC GTCTCCGTCA GCGGATCCGT GAAGAAGTGC ACTATTCAAA

Elephant CACATCAAGC GGCTACGTCA CCGGATCCGG GAGGAAGCCA ATTACCTGAA

Frog CACATAAAGA AGATGAGACC AAAGATACAA GAGGAGTTGC AGGAGCTGAG

Zebrafish CACATCAAGA GGATCAGGAC GCGGGCTCAG GAGGAGATTC AGTCCCGCCC

Chicken CACATCAAAC GGCTGAGGCA CCGGCTACGT GAAGAAACCC AGTACCAAAC

Fugu CACATTAAGA GGATCAGGAC CAGGGCCCTG GAGGATATTC AGTCCCGGCC

Zebra_Finch CACATCAAGA GGCTGCGACA CCGGCTCCGT GAAGAGACGC AGTACCAGAG

Human CCCCAGCCTA CCTCTGGTGC ACCCTCCGTC CCATAGCAAA GCCCCTGCAC

Gorilla CCCTAGCCTG CCTCTGGTGC ACCCCCCGTC CTATACCAAA GCCCCTGCAC

Chimpanzee CCCCAGCCTG CCTCTGGTGC ACCCTCCGTC CCATACCAAA GCCCCTGCAC

Orangutan CCCCAGCCTG CCTCTGGTGC ACCCCCCGTC CCATACCAAA GCCCCTGCAC

Marmoset CCCCAGCCTG CCTCTGGTGC ACCCCCCGGC CCATGCCAAA GCCCCTGCAC

Cow CGCCAGCCTG CCCCTGATGC AGACACCTGG TCATGCCAAG GCTCCGGCAC

Dog CCCCAGCCTG CCACTGATGC AGATGCCGGG CCACCCCAAA GCCTCTGCTC

Guinea_Pig CCCCAGCCTG CCTCTGATGC ACCCCCCAGC CCATGCCAAA GCCTCAGCCC

Horse CTCCAGCCTG ACCCTGATGC AGACGCCGGG CCACGCCAAA GCCCCAGCAC

Mouse CCCAAGCTTG CCTCTGATGC ACCCTCCAGC CCACACCAAA GCCCCAGCTC

Pig CGCCAGCCTG CCCCAGATGC AGACGCCCGG CCACGCCAAG GCCCCGGCGC

Rabbit CTCCAGCCTG CCGGTGCTCC ACACGCCCGC CCACGCCAAG GCCCCAGCCC

Rat CCCAAGCTTG CCTCTGATGC ACCCTCCAGC CCATACCAAA GTCCCAGCTC

Elephant CCCTAGCCTG CCTCTGGTGC AGATGCCGGC CAACACCAAG GCC-------

Frog TGCCAACATG CCCTACCAGG TCCCTACTAT GGCCAACAAG CCCCCTTCAC

Zebrafish CTCTCACGGC ATCTCTCTGA CTCAAGCCTC CATGCAGAAT AAACCCGCCC

Chicken TCCTGGCCTT CCCCTGCAGA TGCACTCTGC TCCAACAAAG CCCTCTCCCC

Fugu CACTCCAGGG ATCTCTGTGA CACAAAGT-- -CAGCAGAAC AAGTCGATGC

Zebra_Finch CCCTGGCCTC CCCCTGCAGC TGCACTCTGC CCCCACAAAA CCCCCTCCCC

Human AGACTCCAGC CGAGCCCACA CCTGGCTATG AGGTGGGCCA GCGGAAGCGC

Gorilla AGACTCCAGC CGAGCCCACA CCTGGCTATG AGGTGGGCCA GCGGAAGCGC

Chimpanzee AGACTCCAGC CGAGCCCACA CCTGGCTATG AGGTGGGCCA GCGGAAGCGC

Orangutan AGACTCCAGC CGAGCCCACA CCTGGCTACG AGATGGGCCA GCGGAAGCGC

Marmoset AGACTCCAGC CGAGCCCGCA CCTGGCTACG AGATGGGACA GCGGAAGCGC

Cow AGGCCCCGGC CGAGTCCACG CCCACCTATG AGACGGGCCA GCGGAAACGC

Dog AGGCGCCGGC AGAGCCTGTG CCCACGTATG AGACAGGCCA GCGGAAGCGT

Guinea_Pig AGGCCCCACC AGAACCCACG CCTGCCTACG AGACAGGTCA GCGGAAGCGC

Horse AGGCTCCAGC CGAGCCCATG CCCACCTACG AGACGGGCCA GCGGAAGCGC

Mouse AAGCCCCT-- -GAGGCTACA CCCACCTATG AAACAGGCCA GCGGAAGCGC

Pig AGGCCCCAGC GGAGCCCACG CCCACCTACG AGACGGGCCA GCGCAAGCGT

Rabbit AGGCCCCCGC CGAGCCGGCG CCCCCCTACG AGACGGGCCA GCGCAAGCGC

Rat AAGCCCCTAC GGAGGCTACA CCCACCTACG AGACAGGCCA GCGGAAACGC

Elephant -----CCTGC AGAGGCCACA CCCACCTATG AAACCGGCCA GAGAAAGCGC

Frog AAAACCCACA GACTCCCCCC GGCGCATTTG AAACCGGCCA AAGGACAAGG

Zebrafish ACCAGCCGCC GGGAGCAGCA CAGAATCCAG ACGCCGGCCA GAGGAAGAGG

Chicken AGATGGCTCA GGACCCACCG CCGGCCTACG AGACCGGTCA GAGGAAGCGG

Fugu AGCAGAGCAC TTCAGCGGGG AACCCAGAGG TCACCGGGCA GAGAAAAAGA

Zebra_Finch AGATCCCCCA GGACCCCCCT CCCACCTACG AGACGGGGCA GAGGAAGAGG

Human CTCATCTCCT CGGTGGAGGA CTTCACCGAG TTTGTG

Gorilla CTCATCTCCT CGGTGGAGGA CTTCACCGAG TTTGTG

Chimpanzee CTCATCTCCT CGGTGGAGGA CTTCACCGAG TTTGTG

Orangutan CTCGTCTCCT CCGTGGAGGA CTTCACCGAG TTCGTG

Marmoset CTCATCTCGT CTGTGGAGGA CTTCACCGAG TTCGTG

Cow CTGGTCTCCT CTGTGGACGA CTTCACTGAG TTCGTG

Dog CTCATCTCCT CTGTGGATGA CTTCACGGAG TTTGTG

Guinea_Pig CTCATCTCCT CAGTGGATGA CTTCACAGAG TTTGTG

Horse CTCATCTCTT CCGTGGACGA CTTCACAGAG TTTGTG

Mouse CTCATTTCCT CCGTGGATGA CTTCACAGAG TTTGTG

Pig CTCATCTCCT CCGTGGACGA CTTCACCGAG TTTGTG

Rabbit CTCCTCTCCT CCGTGGATGA CTTCACGGAG TTCGTG

Rat CTCATTTCCT CTGTGGATGA CTTCACAGAG TTTGTG

Elephant CTCATCTCCT CAGTAGACGA CTTCACAGAA TTTGTG

Frog CTCATTTCTG CTGTGGACGA TTTCACGGAG TTTGCT

Zebrafish CTGGTGTCCA CCGTCGACGA CTTCACTGAC TTTGTG

Chicken CTGATCTCCT CGGTGGATGA CTTCACTGAG TTTGTG

Fugu CTGGTGTCCA CAGTGGACGA CTTCACCGAT TTTGTT

Zebra_Finch CTGATCTCCT CCGTGGACGA CTTCACCGAG TTCGTG

;

END;

BEGIN DATA;

DIMENSIONS NTAX=21 NCHAR=4146;

FORMAT DATATYPE=DNA INTERLEAVE=yes GAP=-;

[Gene Name: MET]

[Name: Human Len: 4146 Check: 0]

[Name: Gorilla Len: 4146 Check: 0]

[Name: Orangutan Len: 4146 Check: 0]

[Name: Chimpanzee Len: 4146 Check: 0]

[Name: Marmoset Len: 4146 Check: 0]

[Name: Dog Len: 4146 Check: 0]

[Name: Mouse Len: 4146 Check: 0]

[Name: Rabbit Len: 4146 Check: 0]

[Name: Guinea_Pig Len: 4146 Check: 0]

[Name: Horse Len: 4146 Check: 0]

[Name: Pig Len: 4146 Check: 0]

[Name: Cow Len: 4146 Check: 0]

[Name: Rat Len: 4146 Check: 0]

[Name: Elephant Len: 4146 Check: 0]

[Name: Opossum Len: 4146 Check: 0]

[Name: Platypus Len: 4146 Check: 0]

[Name: Frog Len: 4146 Check: 0]

[Name: Zebra_Finch Len: 4146 Check: 0]

[Name: Chicken Len: 4146 Check: 0]

[Name: Zebrafish Len: 4146 Check: 0]

[Name: Fugu Len: 4146 Check: 0]

MATRIX

Human ATGAAGGCCC CCGCTGTGCT TGCACCTGGC ATCCTCGTGC TCCTGTTTAC

Gorilla ATGAAGGCCC CCGCTGTGCT TGCACCTGGC ATCCTCGTGC TCCTGTTTAC

Orangutan ATGAAGGCCC CCGCTGTGCT TGCACCTGGC ATCCTCGTGC TCCTGTTTAC

Chimpanzee ATGAAGGCCC CCGCTGTGCT TGCACCTGGC ATCCTCGTGC TCCTGTTTAC

Marmoset ATGAAGGCCC CCGCTGTGCT TGCACCTGGC ATCCTCGTGC TCCTGTTTAC

Dog ATGAAGGCTC CTGCTGTGCT TGCACCTGGC ATCCTTGTGC TTCTGTTTAC

Mouse ATGAAGGCTC CCACCGTGCT GGCACCTGGC ATTCTGGTGC TGCTGTTGTC

Rabbit ATGAAGGCGC CCGCTGTGCT TGCACCTGGC ATCCTTGTGC TCCTGTTTAC

Guinea_Pig ATGAAGGCCC CCACTGCGCT TGCACCTGGC GTCCTGGTGC TGCTGTTTAC

Horse ATGAAGGCAC CTGCTGTGCT TGCACCTGGC ATCCTTGTGC TTCTGTTTAC

Pig ATGAAGGCCC CTGCTGTGCT TGCACCTGGC ATCCTTGTGC TTCTGTTTAC

Cow ATGAAGGCCC CTGCTGTGCT TGCACCTGGC ATTCTTGTGC TTCTGTTTAC

Rat ATGAAGGCTC CCACCGCGCT GGCACCTGGC ATTCTGCTGC TGCTGCTGAC

Elephant ATGAAGGCCC CTGCTGTGCT TGCACCTGGT GTCCTTGTGC TCCTGTTTAC

Opossum ATGAAGCCCA CTGCTATGCT TGTGCCTGGG TTTCTGATCT TCCTGCTTGC

Platypus ---------- ---------- ---------- ---------- ----------

Frog GTACATGCCA AGATGCTCGT GGCTGTTCCT GTTGCTTTTG TGTTCCTAAG

Zebra_Finch ATGAAGCCTT TTACTGCATA TCCTTCTGGG TTTATCGTAT TCCTGTTTGC

Chicken ATGAAGCCTG TTACTGCCTA TCCTTCTGGG ATTATCCTAT TCCTGTTTGC

Zebrafish ATGACAATTC ACTATTCTGA AGCTGCTTCC ATCCTAATCA TCCTTCAGTC

Fugu ATG------A ATCTCTGCTC CTTTCCTGCC GCCGTGCTGC TGCTGTGGGC

Human CTTGGTGCAG AGGAGCAATG GGGAGTGTAA AGAGGCACTA GCAAAGTCCG

Gorilla CTTGGTGCAG AGGAGCAATG GGGAGTGTAA AGAGGCACTA GCAAAGTCCG

Orangutan CTTGGTGCAG AGGAGCAATG GGGAGTGTAA AGAGGCACTA GCAAAGTCCG

Chimpanzee CTTGGTGCAG AGGAGCAATG GGGAGTGTAA AGAGGCACTA GCAAAGTCCG

Marmoset CTTGGTGCAG AGGAGCAATG GGGAGTGTAA AGAGGCACTA ACGAAGTCTG

Dog CTTGGTGCAG AAGAGCTATG GGGAGTGCAA AGAGGCACTA GTAAAGTCTG

Mouse CTTGGTGCAG AGGAGCCATG GGGAGTGCAA GGAGGCCCTA GTGAAGTCTG

Rabbit CTTGGTGCCG AGGAGCCATG GGGAGTGTAA AGAGGCACTG GTGAAGTCCG

Guinea_Pig CTTGGTGCAG AGGAGCGATG GGGAATGTGC GGAGGCACTA GTGAAGTCTG

Horse CTTGGTGCAG AAGAGCGATG GGGAGTGTAA AGAGGCGCTA GTAAAGTCCG

Pig CTTGGTGCAG AAGAGCAAGG GAGAGTGTAA AGAGGCACTG GTAAAGTCCA

Cow CTTCGTGCAG AAGAGCAATG GGGAGTGTAA AGAGGCACTA GTAAAATCCA

Rat CTTGGCGCAG AGGAGCCATG GGGAGTGCAA GGAGGCCCTA GTGAAGTCTG

Elephant CTTGGTGCGG AAGAGCCATG GGGAGTGTGA AGAGGCACTT GCCAAGTCCA

Opossum CTTGGTGCAG AATTGCAAAG GAGAATGTAA AGAAGCCCTC GTCAAGTCTG

Platypus ---------- ---------- ---------- ---------- ----------

Frog CTTGCTGCTG AAATGTATGG GACAATGCGA GGAAGCAGCC AAAATGGCAG

Zebra_Finch CTTGCTGCAG AGGAGCCATG GACAATGCAA AGAAGCAGCT AAAAAATCAG

Chicken GTTGCTGCAA AGAAGTCATG GACAATGCAA AGAGGCAGCT AAAAAATCAG

Zebrafish GCTGTGGTGG GGCTTGAATT GTCAATGTGA GGAACCAATA GAAAGCTCCA

Fugu CCTGAGTGCC AGCGCCGAGG GCCACTGCCA AAAATCCCCT GAGCCCAACA

Human AGATGAATGT GAATATGAAG TATCAGCTTC CCAACTTCAC CGCGGAAACA

Gorilla AGATGAATGT GAATATGAAG TATCAGCTTC CCAACTTCAC CGCGGAAACA

Orangutan AGATGAATGT GAATATGAAG TATCAGCTTC CCAACTTCAC CGCGGAAACA

Chimpanzee AGATGAATGT GAATATGAAG TATCAGCTTC CCAACTTCAC CGCGGAAACA

Marmoset AGATGAATGT GAATATGAAA TATCAGCTTC CCAACTTCAC TGCAGAAACA

Dog AGATGAATGT GAACATGAAG TATCAGCTTC CCAACTTCAC TGCCGAAACA

Mouse AGATGAACGT GAACATGAAG TATCAGCTCC CCAACTTCAC GGCAGAAACC

Rabbit AGATGAATGT GAACATGAAG TATCAGCTTC CCAACTTCAC CGCTGAAACG

Guinea_Pig AGATGAACAT GAACATGCAG TATCAGCTTC CCAACTTCAC TGCAGAAACA

Horse AGATGAATGT GAACATGAAG TATCAGCTTC CCAACTTCAC TGCAGAAACA

Pig CGATGAATTT GAACATGCAA TATCAGCTTC CTAACTTCAC TGCGGAAACA

Cow GGATGAATGT GAACATGCAA TATCAGCTTC CTAACTTCAC TGCAGAAACA

Rat AGATGAACGT GAACATGAAG TACCAGCTTC CCAACTTCAC CGCAGAAACC

Elephant AGATGAATGT GAACATGAAG TATCAGCTTC CCAACTTCAC TGCAGACACA

Opossum AGATGGACGT TAATAGGAAG TATCCACTTC CCAACTTTAT TGCAGAAACT

Platypus ---------- ---------- ---------- ---------- ----------

Frog AAATGGATTT GAATATAAAG CACAACCTCC AGAGTTTCAC TACAGATACA

Zebra_Finch AGATGAATCT TGGTGTAAAA TATGATCTTC CTAACTTCAT TGCAGACACA

Chicken AGATGAACTT GAATGTAAAA TATGATCTTC CTAACTTCAT CACAGAGACG

Zebrafish AACTCGACCT CTCAGTGACC TATGACCTCC CTTACTTTGT GTCTGACACC

Fugu AGCTGAACCT CTCCGTCAGC TACGAGCTGC CGACCTTCAC TGCAGAATTC

Human CCCATCCAGA ATGTCATTCT ACATGAGCAT CACATTTTCC TTGGTGCCAC

Gorilla CCCATCCAGA ATGTCATTCT ACATGAGCAT CACATTTTCC TCGGTGCCAC

Orangutan CTCATCCAGA ATGTCATTCT ACATGAGCAT CACATTTTCC TTGGTGCCAC

Chimpanzee CCCATCCAGA ATGTCATTCT ACATGAGCAT CACATTTTCC TCGGTGCCAC

Marmoset CCCATCCAGA ATGTCATTCT ACATGAGCAT CACATTTTCC TTGGTGCCAC

Dog CCCATCCAGA ATGTTGTTTT ACACAAGCAT CATATTTACC TTGGTGCAGT

Mouse CCCATCCAGA ATGTCGTCCT ACACGGCCAT CATATTTATC TCGGAGCCAC

Rabbit CCCATCCAGA ACGTCCTTCT GCACCAGCAT CACGTCTACC TCGGTGCCAT

Guinea_Pig CCCATCCAGC ACGTTGCTCT ACACAGGCAC CACATTTACC TGGGGGCCAC

Horse CCCATCCAGA ACGTCGTTTT ACACAAGCAT CACATTTACC TCGGGGCCAC

Pig CCCATTCAGA ATGTCGTTTT ACACAAGCAT CACATTTACC TGGGTGCCCT

Cow TCCATTCAGA ATGTTGTTTT GCACAAACAT CACATTTACC TCGGTGCCAT

Rat CCCATCCACA ATGTCGTCCT CCATGGGCAC CATATTTATC TCGGAGCCAC

Elephant CCCATCCAGA ACGTCGTTTT GCATGAGCAT CACATTTTCC TTGGTGCCAT

Opossum GAGATCCAAA ACATTATTTT ACACAATAAC CACATCTTCC TGGGTGCAGT

Platypus ---------- ---------- ---------- ---------- ----------

Frog CCAATACAAA ATATTATCAT GTTTAAGGGC TACATATATG TGGGAGCAGT

Zebra_Finch CCAATTCAGA ATGTAGTTTT ATACAAGCAT CATGTTTATA TTGGAGCAGT

Chicken CCAATTCAGA ATGTAGTTTT ATACAAGCAT CATGTTTATA TTGGAGCAGT

Zebrafish CCCATTCAGA AGCTGTTGGA AATCAATGGA ACAGTGTATG TCGGTGCCGT

Fugu CCCATCCAGA ACGTGGTGAC CCTCGACGGC ATCATCTACG TGGGGGCCAC

Human TAACTACATT TATGTTTTAA ATGAGGAAGA CCTTCAGAAG GTTGCTGAGT

Gorilla TAATTACATT TATGTTTTAA ACGAGGAAGA CCTTCAGAAG GTTGCTGAGT

Orangutan TAACTACATT TATGTTTTAA ATGAGGAAGA CCTTCAGAAG GTTGCTGAGT

Chimpanzee TAACTACATT TATGTTTTAA ATGAGGAAGA CCTTCAAAAG GTTGCTGAGT

Marmoset TAACTACATT TATGTTTTAA ATGAGGAAGA CCTTCAGAAG GTTGCTGAGT

Dog TAACTATATT TACGTTTTAA ATGACAAAGA CCTTCAGAAG GTTGCTGAGT

Mouse AAACTACATT TATGTTTTAA ATGACAAAGA CCTTCAGAAG GTATCCGAAT

Rabbit CAACCACATC TACGTTTTAA ATGACAAAGA CCTTCAGAAG GTTGCTGAAT

Guinea_Pig CAACTACCTT TATGTTTTAA ATGACAAAGA CCTTCAGAAG GTAGCTGAGT

Horse TAACTACATT TATGTTTTAA ATGACAAAGA TCTTCAGAAA GTTGCTGAGT

Pig TAACTACATT TATGTTTTAA ATGACATAGA CCTTCGGAAG GTTGCTGAGT

Cow TAACTACATT TACGTTTTAA ATGACAAAGA CCTTCAGAAG GTTGCTGAGT

Rat AAACTACATT TATGTTTTAG ATGACAAAGA CCTTCAGAAG GTATCTGAGT

Elephant TAACAACATT TATGTTCTAA ATGACAAAGA CCTCCAGAAG GTTGCTGAGT

Opossum CAACAAGATT TATGTCTTAA AT---GAAGA TCTCCATAAT GTTTCTGAGT

Platypus ---------- ---------- ---------- ---------- ----------

Frog GAATAAGATT TATGTCTTGA AT---GAAAA TCTTACAAAG GTTTCTGAGT

Zebra_Finch GAATAAGATT TACGTACTAA AT---GAAAC TCTCCAGAAC ATTTCTGTGT

Chicken CAACAAGATT TATGTACTAA AC---GAGAC TCTCCAAAAC ATTTCTGTCT

Zebrafish CAATAGACTT TACGCTCTGT CG---AAAGA CCTGAAGAAG AAACATGAGT

Fugu GAACAGGATC TACGCCTTGG CC---CCGAG CCTCACCAAG CTGTCGGAGT

Human ACAAGACTGG GCCTGTGCTG GAACACCCAG ATTGTTTCCC ATGTCAGGAC

Gorilla ACAAGACCGG GCCTGTGCTG GAACACCCAG ATTGTTTCCC ATGTCAGGAC

Orangutan ACAAGACCGG GCCTGTGTTG GAACACCCAG ATTGTTTCCC ATGTCAGGAC

Chimpanzee ACAAGACTGG GCCTGTGCTG GAACACCCAG ATTGTTTCCC ATGTCAGGAC

Marmoset ACAGGACGGG GCCTGTGCTA GAACACCCAG ATTGTTTCCC ATGTCAGGAC

Dog ACAAGACTGG GCCCGTGCTG GAACACCCAG ATTGTTCCCC ATGTCAGGAC

Mouse TCAAGACCGG GCCCGTGTTG GAACACCCAG ATTGTTTACC TTGTCGGGAC

Rabbit ACACGACCGG GCCCGTGCTG GAACACCCGG ACTGTTTGCC GTGTCAGGAC

Guinea_Pig ATAAGACTGG ACCTGTGCTG GAGCATCCGG ATTGTCTTCC ATGTCAGGAC

Horse ACAAGACTGG GCCCGTGCTG GAACACCCAG ATTGTTTCCC ATGTCAGGAC

Pig ACAAAACTGG GCCCGTGCTG GAACACCCAG ATTGTTTCCC ATGTCAGGAC

Cow ACAAGACTGG GCCTGTGCTG GAACACCCAG ATTGTTTCCC ATGTCAGGAC

Rat TCAAGACCGG GCCCGTGGTG GAACACCCAG ATTGTTTTCC TTGTCAGGAC

Elephant ACAAGACCGG GCCCGTGTTG GAGCACCCAG ATTGTTTGCC GTGTCAGGAC

Opossum ACAAGACAGG ACCTGTGCTA GAACACCCGG ACTGTGCTCC ATGTCAAGAC

Platypus ---------- ---------- ---------- ---------- ----------

Frog ATAAAACAGG TCCATTATTG GTACATTCTC ATTGTTTGCC ATGTAAAAAC

Zebra_Finch ACAAGACTGG GCCTGTTTTG GAGAACCCTG ACTGTGCACC ATGTGAAGAC

Chicken ACAAGACTGG GCCAATTTTG GAGAGTCCTG GCTGCGCACC ATGTGAAGAC

Zebrafish ATAAGACTGG ACCGGTCCAT GAGGGTCCAG ACTGCAAGAC CCCAACAGAT

Fugu ACCGCACGGG GCCACTTCTC GCCAACCAGA CTTGTGGTCA GAAAGTAGCC

Human TGCAGCAGCA AAGCCAATTT ATCAGGAGGT GTTTGGAAAG ATAACATCAA

Gorilla TGCAGCAGCA AAGCCAATTT ATCAGGAGGT ATTTGGAAAG ATAACATCAA

Orangutan TGCAGCAGCA AAGCCAATTT ATCAGGAGGC GTTTGGAAAG ATAACATCAA

Chimpanzee TGCAGCAGCA AAGCCAATTT ATCAGGAGGT GTTTGGAAAG ATAACATCAA

Marmoset TGCAGCAGCA AAGCCAATTT ATCAGGAGGA GTTTGGAAAG ATAACATCAA

Dog TGCAGCCACA AAGCCAATTT ATCAGGTGGT GTTTGGGAAG ATAACATCAA

Mouse TGCAGCAGCA AAGCCAATTC ATCAGGAGGG GTTTGGAAAG ACAACATCAA

Rabbit TGCAGCAGCA AAGCCAATTC CTCAGGTGCT GTTTGGAAAG ATAACATCAA

Guinea_Pig TGCAGCAGTA AAGCCAATTT ATCAGGAGCC ATTTGGAAAG ATAACATCAA

Horse TGCAGCCGCA AAGCCAATTT ATCAGGTGGC GCTTGGAAAG ATAACATCAA

Pig TGCAGCCACA AAGCCAATTT ATCAGGTGGC ATTTGGAGAG ATAACATCAA

Cow TGCAGCCATA AAGCCAATTT ATCAGGTGGT GTTTGGAAAG ATAACATCAA

Rat TGCAGCAGCA AAGCCAATGT GTCAGGAGGT GTTTGGAAAG ACAACGTCAA

Elephant TGCAGTAGCA AAGCCAATTT ATCAGGTAGT GTTTGGAAAG ATAACATCAA

Opossum TGTAGTCACA AAGCTAACTT ATCTGGTAGC ATTTGGAAGG ATAATGTCAA

Platypus ---------- ---------- ---------- ---------- ----------

Frog TGCACGGATA ATTTTCTGTC CCCAAATGGC ACCTGGAAAG ACAACGTTAA

Zebra_Finch TGCCGAGAAA AAGCCAATTT ATCTAACAGC ATATGGAAGG ATAACGTCAA

Chicken TGCAAAGATA AAGCTAATCT GTCTAACAGC GTATGGAAGG ATAATGTCAA

Zebrafish CAATGCAGT- ---------- -GGTTGTGAA AACAAGCCCC GTAACATAAA

Fugu AACGCGAGC- ---------- -TCTGGCGGC GGCAGAAAGG ACAACCTCAA

Human CATGGCTCTA GTTGTCGACA CCTACTATGA TGATCAACTC ATTAGCTGTG

Gorilla CATGGCTCTA GTTGTCGACA CCTACTATGA TGATCAACTC ATTAGCTGTG

Orangutan CATGGCTCTA GTTGTCGACA CCTACTATGA TGATCAACTC ATTAGCTGTG

Chimpanzee CATGGCTCTA GTTGTCGACA CCTACTATGA TGATCAACTC ATTAGCTGTG

Marmoset CATGGCTCTG GTTGTCGACA CCTACTATGA TGACCAACTC ATTAGCTGTG

Dog CATGGCTCTG CTTGTTGACA CATACTACGA TGACCAACTC ATTAGCTGTG

Mouse CATGGCTCTG CTTGTTGACA CATACTATGA TGATCAACTC ATTAGCTGTG

Rabbit CCTGGCTCTG CTTGTTGACA AGTACTATGA TGACCAACTC ATTAGCTGTG

Guinea_Pig CATGGCTATG CTTATTGACA CGTACTATGA TGACCAGCTC ATTAGTTGTG

Horse CATGGCTCTG CTTGTTGACA CATACTATGA CGATCAACTC ATTAGTTGCG

Pig CATGGCTCTG CTTGTTGACA CGTATTATGA TGACCAACTC ATTAGCTGCG

Cow CATGGCTCTG CTTGTTGACA CGTACTATGA TGATCAACTC ATTAGCTGTG

Rat CATGGCGCTG CTTGTTGACA CTTACTATGA CGACCAGCTC ATCAGCTGTG

Elephant CATGGCCCTG CTTGTTGACA CATACTATGA TGACCAACTC ATTACCTGTG

Opossum CAAAGCCTTG CTCGTTGATA CTTACTATGA CGATCAGCTT ATTAGTTGTG

Platypus ---------- ---------- ---------- ---------- ----------

Frog TATGGCTCTG TTTGTGGAGG ATTTCTATGA TGACCAGCTC ATTAGTTGTG

Zebra_Finch CATGGCACTG CTTTTAGAAA CTTATTATGA TGATCAACTC ATCAGCTGTG

Chicken CATGGCACTG CTCTTAGAAA CCTATTACGA TGATCAGCTC ATCAGCTGTG

Zebrafish CATGGCCCTG TTAATGGAGA CGTTCTATGA CCTGGAACTT TTCAGCTGTG

Fugu CGTCGCTCTG GTGGTGGAAA ACATTTACGA CAAAGGGCTG TTCAGCTGCG

Human GCAGCGTCAA CAGAGGGACC TGCCAGCGAC ATGTCTTTCC CCACAATCAT

Gorilla GCAGCGTCAA CAGAGGGACC TGCCAGCGAC ATGTCTTTCC CCACAATCAT

Orangutan GCAGCGTCAA CAGAGGGACC TGCCAGCGAC ATGTCTTTCC CCACAATCAT

Chimpanzee GCAGCGTCAA CAGAGGGACC TGCCAGCGAC ATGTCTTTCC CCACAATCAT

Marmoset GCAGCGTCAA CAGAGGAACC TGCCAGCGAC ACGTCTTTCC CCACAATCAT

Dog GCAGTGTCCA CAGAGGGACC TGCCAGCGAC ATATCCTTCC ACCCAGCAAT

Mouse GCAGTGTCAA CAGAGGGACT TGCCAGCGGC ATGTCCTTCC TCCTGACAAT

Rabbit GCAGCGTCAA CAGAGGGACC TGCCAGCGAC ACGTCCTCCC ACCTGATAAT

Guinea_Pig GCAGTGTCAA CAGAGGGACC TGCCAGCGAC ACGTCCTTCC ACTCAGTAAT

Horse GCAGCGTCCA CAGAGGGACC TGCCAGCGGC ACGTCCTTCC ACTCAACAAT

Pig GCAGTGTCCA CAGAGGGACC TGCCAGCGGC ATGTCCTTCC ACCTGATAAT

Cow GCAGTGTCCA CAGAGGGACC TGCCAGCGAC ACGTCCTTCC ACCCAACAAT

Rat GCAGCGTCAA CAGAGGGACC TGCCAAAGGC ATGTCCTTCC TCCTGACAAT

Elephant GCAGCGTCAA CAGGGGGACC TGCCAGCGGC ACGTCCTACC ACCTGATAAT

Opossum GTAGTGTTAA TCGAGGGACC TGCCAGCGGC ATGTGTTTCA ACCTAATAAT

Platypus ---------- ---------- ---------- ---------- ----------

Frog GAAATATACG TAGAGGTGAA TGTCAGCGTC ATACTTTGCA TTCTGATAAA

Zebra_Finch GTAGTGTGTC CGGAGGCATC TGCCACCGTC ACATCATTCA CCCTGACAAC

Chicken GTAGTGTGTC TGGAGGTGTC TGTCACCGTC ACATCATTCC CCCTGACAAC

Zebrafish GCTCAGTCGG GAATGGCGTC TGCAGTCGTC ATGTGTTAGA GGATGGGCCT

Fugu GCTCGGCGGA CAACGGCGTT TGCCGCCGCC ACGTCCTGGA AGACGACGTT

Human ACTGCTGACA TACAGTCGGA GGTTCACTGC ATATTCTCCC CACAGATA--

Gorilla ACTGTTGACA TACAGTCGGA GGTTCACTGC ATATTCTCCC CACAGATA--

Orangutan ACTGCTGACA TACAGTCGGA GGTTCACTGC ATATTCTCCC CACAGATA--

Chimpanzee ACTGCTGACA TACAGTCGGA GGTTCACTGC ATATTCTCCC CACAGATA--

Marmoset ACTGCTGACA TTCAGTCGGA GGTTCACTGC ATATTCTCCG CGCAGACA--

Dog ATTGCTGACA TACAGTCGGA AGTGCATTGC ATGTACTCCT CACAGGCAGA

Mouse TCTGCTGACA TCCAGTCTGA GGTCCACTGC ATGTTCTCCC CA------GA

Rabbit CCTGCTGACA TACAGTCGGG GGTTTACTGT ATGTTCTCCC CACAGGCAGA

Guinea_Pig ACTGCTGACA TACAGGCAAA AGTATATTGC ATGTTCTCAC AG---GCAGA

Horse GTTGCTGACA TACAGTCAGA AGTTTATTGC ATGTACTCTC CACAAGCA--

Pig ATCGCTGACA TCAAGTCAGA GGTTCACTGC ATGTACTCCC CACAGGTAGA

Cow ACTGCAGACA TAGAGTCGGA GGTTCACTGC ATGTACTCTC CTCAGGCAGA

Rat GCTGCCGACA TTCAGTCCGA GGTTCACTGC ATGTTCTCCC CACTTGCGGA

Elephant CCTGCTGACA TACACTCGAA GGTGCATTGC ATGTACTCCC CACAGGCAGA

Opossum ACTGCTGACA TACAGTCAAA TGTTTATTGT ATATACTCCC CACAGATGGA

Platypus ---------- ---------- ---------- ---------- ----------

Frog CCCTGGGATA TAGCAAGTAA TGTTCATTGC TTATATTCAT CACAAATGAT

Zebra_Finch CCTGCTGACA TTGAAAGTGA GGTGCACTGC ATGTACTCAC CCCAGGCAGA

Chicken CCTGCTGACA TAGAAAGCGA GGTGCACTGC ATGTATTCAC CGCAGGTAGA

Zebrafish ---------C TGGGTGCGGA AGTAACTTGC ATGTACACCA AAAAGAATGA

Fugu AGCCTGGACG TGGACGAACT GGTCTATTGC TTCACTGACC TTAAACAGGA

Human -GAAGAGCCC AGCCAGTGTC CTGACTGTGT GGTGAGCGCC CTGGGAGCCA

Gorilla -GAAGAGCCC AGCCAGTGTC CTGACTGTGT GGTGAGCGCC CTGGGAGCCA

Orangutan -GAAGAGCCC AGCCAGTGTC CTGACTGTGT GGTGAGCGCC CTGGGAGCCA

Chimpanzee -GAAGAGCCC AGCCAGTGTC CTGACTGTGT GGTGAGCGCC CTGGGAGCCA

Marmoset -GAAGAGCCC AGCCAGTGTC CTGACTGCAT GGTGAGCGCC CTGGGAACCA

Dog CGAAGAGCCC AGCCAGTGCC CTGACTGTGT GGTGAGTGCT CTAGGAACCA

Mouse AGAGGAGTCA GGGCAGTGTC CTGACTGTGT AGTGAGTGCC CTCGGAGCCA

Rabbit TGAAGAGCCT GGCCAGTGTC CTGACTGCGT GGTGAGCCCC CTGGGAGCCA

Guinea_Pig TGAAGAGTCC GGCCAGTGTC CTGACTGTGT GGTGAGCGCT CTGGGAACCA

Horse -GAAGAGCCC CACCAGTGTC CTGACTGTGT GGTGAGTGCC CTGGGAACCA

Pig TGAAGAGCCC AGCCAGTGTC CTGACTGTGT GGTGAGTGCG CTGGGAACCA

Cow CGAAGAGACC AACCAGTGTC CTGACTGTGT GGTGAGTGCC CTGGGAACCA

Rat GGAAGAGTCA GGCCAGTGTC CCGACTGTGT AGTGAGTGCC CTGGGAGCCA

Elephant CGAGGAGCCC AGCAAGTGTC CTGACTGCGT GGTGAGCGCT CTGGGAACCA

Opossum TGAGGAGTCC CTCCAATGTC CTGACTGTGT GGTAAGCACT TTAGGTACTA

Platypus ---------- ---------- ---------- ---------- ----------

Frog AGAAGATAAA GACAATTGTC CTGACTGCAT TGTTAGTACA ACAGGAAGCA

Zebra_Finch TGGAGAAGCA GACAACTGTC CTGATTGTGT TGTTAGCACG TTAGGAACCA

Chicken CGGAGAGGCA GATAATTGTC CTGACTGTGT TGTAAGCACT TTAGGAACCA

Zebrafish AGGCAGCAGC CATGGATGCC CAGACTGCCT GGCTGGACCT GCGGGCACTC

Fugu CAAGGGCCAA CCCCGAGACT CCGATGTGGT GGTCAGCCCT TCGGGTTCTC

Human AAGTCCTTTC ATCTGTAAAG GACCGGTTCA TCAACTTCTT TGTAGGCAAT

Gorilla AAGTCCTTTC ATCTGTAAAG GACCGGTTCA TCAACTTCTT TGTAGGCAAT

Orangutan AAGTCCTTTC ATCTGTAAAG GACCGGTTCA TCAACTTCTT TGTAGGTAAT

Chimpanzee AAGTCCTTTC ATCTGTAAAG GACCGGTTCA TCAACTTCTT TGTAGGCAAT

Marmoset AAGTCCTTTT ATCTGTAAAG GACCGGTTCC TCAACTTCTT TGTAGGCAAT

Dog AAGTCCTGAT ATCAGAAAAG GACCGGTTCA TCAACTTCTT CGTAGGCAAT

Mouse AAGTCCTCCT GTCGGAAAAG GACCGGTTCA TCAATTTCTT TGTGGGGAAT

Rabbit AAGTTCTCCT ATCCAAAAAG CAGCGCTTCA TCTACTTCTT TGTGGGCAAT

Guinea_Pig AAGTCCTTCT GTCTGAAAAG AGTCGGTTCA TCAACTTCTT TGTGGGCAAT

Horse AAGTCCTGCT GTCTGAAAAG GACCGATTTG TCACCTTCTT CGTGGGCAAT

Pig AAGTCCTGCT GTCTGAAAAG GACCGGTTCA TCAACTTCTT CGTGGGTAAT

Cow AAGTCCTACT GTCTGAAAAG GATCGATTCA TCAACTTCTT CGTGGGCAAT

Rat AAGTCCTCCT GTCTGAAAAG GACCGGTTCA TCAATTTCTT CGTGGGGAAT

Elephant AAGTCCTACT TACCGAGAAG GACAGGTTCA TCAACTTCTT TGTAGGTAAT

Opossum AAGTTCTGCT GTCAGAAAAA GAACGATTTA TAAACTTCTT TGTGGGCAAT

Platypus ---------- ---------- ---------- ---------- ----------

Frog AGATCCTGGT AACAGTGAGA GACAGGTTTG TAAAGTTTTT TGTGGGTAGC

Zebra_Finch AAGTTTTGGT GACTGAAAAG GACAGGTTTG TCAACTTTTT TGTTGGAAAT

Chicken AAGTTCTGGT GACTGAAAAG GACAGGTTTG TCAACTTCTT TGTTGGAAAC

Zebrafish AGATCCTCAA CATAATGAGC GGTCGTGTTG TGAGGTTCTT CGTTGCGAAC

Fugu AGGTGCTTAA TGTGGAGAGC AAC---ATGA TCATGTTTTT CGTTGGAAAC

Human ACCATAAATT CTTCTTATTT CCCAGATCAT CCATTGCATT CGATATCAGT

Gorilla ACCATAAATT CTTCTTATTT CCCAGATCAT CCGTTGCATT CGATATCAGT

Orangutan ACCATAAATT CTTCTTATTT CCCAGATCAT CCATTGCATT CAATATCAGT

Chimpanzee ACCATAAATT CTTCTTATTT CCCAGATCAT CCGTTGCATT CAATATCAGT

Marmoset ACCATAAATT CTTCTTACTT CCCAGATCAT TCATTGCATT CGATATCAGT

Dog ACCATAAATT CCTCGGACCA TCCAGATCAT TCATTGCATT CGATATCGGT

Mouse ACGATCAATT CCTCCTATCC TCCTGGTTAT TCACTGCATT CGATATCGGT

Rabbit ACCATAAATT CTTCTTACCT TCCAGATCAC TCATTGCATT CGATATCAGT

Guinea_Pig ACCATAAATT CTTCTTACCT GCCAGATCAC TCATTGCATT CAATATCAGT

Horse ACCATAAATT CTTCTTACCT TCCAGATCAT TCATTGCATT CGATATCGGT

Pig ACCATAAATT CTTCTTACCT TCCGGATCAT TCATTGCATT CAATATCAGT

Cow ACCATAAATT CTTCTTACCT TCCAGATTAT ATATTGCACT CGATATCAGT

Rat ACGATAAACT CTTCCTACCC TCCCGATTAT TCATTGCATT CAATATCGGT

Elephant ACTGTCAATT CTTCCTACCT TCCAGATCAT TCATTGCATT CGATATCGGT

Opossum ACCATTAACT CTTCT---GA TCCCGACAAT TCTTTACATT CAATATCTGT

Platypus ---------- ---------- ---------- ---------- ----------

Frog ACACTAACAG GACAA----- ----CCTTCG ACGATTCATT CTGTGTCAGT

Zebra_Finch ACTGTGACAT CTACATTTCA ACCTCCCCAT GTGCTGCATT CAATATCGGT

Chicken ACCGTGACAT CTGCATTTCA ACCTCCCCAT GTACTGCATT CAATATCAGT

Zebrafish TCTGAACCTC TTGAGTCAGG TCCACGTCTC CAC---CACA CTATTTCCAT

Fugu TCGGAAATTC CTGGCTCCGG ACCCACGGCG CGTCCCCACA CCATGTCCCT

Human GAGAAGGCTA AAGGAAACGA AAGATGGTTT TATGTTTTTG ACGGACCAGT

Gorilla GAGAAGGCTA AAGGAAACGA AAGATGGTTT TATGTTTTTG ACGGACCAGT

Orangutan GAGAAGGCTA AAGGAAACGA AAGATGGTTT TATGTTTTTG ACGGACCAGT

Chimpanzee GAGAAGGCTA AAGGAAACGA AAGATGGTTT TATGTTTTTG ACAGACCAGT

Marmoset GAGAAGGCTA AAGGAAACAA AAGATGGTTT TATGTTTTTG ACGGACCAGT

Dog GAGAAGGCTA AAGGAAACGC AAGATGGGTT CAAGTTTTTG ACAGACCAGT

Mouse GAGACGGCTG AAGGAAACCC AAGATGGTTT TAAGTTTTTG ACAGACCAGT

Rabbit GAGAAGGCTG AAGGAAACAC AGGATGGTTT TAAGTTTCTG ACGGATCAGT

Guinea_Pig GAGAAGGCTA AAAGAAACAC TAGATGGTTT TAAGTTTTTG ACAGACCAGT

Horse GAGAAGGCTA AAGGAAACGC AAGATGGTTT TAAGTTTTTG ACAGATCAGT

Pig GAGAAGGCTA AAGGAAACGC AAGATGGTTT TAAGTTTTTG ACAGACCAGT

Cow GAGAAGGCTA AAGGAAACAC AAGATGGTTT TAAGTTTTTG ACAGACCAAT

Rat GAGGCGGCTG AAGGAAACCC AGGACGGTTT TAAGTTTTTG ACAGACCAGT

Elephant GAGAAGGCTA AAGGAAACGC AAGATGGATT TAAGTTTTTG ACGGACCAGT

Opossum CAGGAGGTTA AAGGAAACAC AAGACGGTTT TAAATTTTTG ACAGACCAGT

Platypus ---------- ---------- ---------- ---------- ----------

Frog GCGTAGACTG AAGGAGACCC AGGATGGCTT TGAATACCTC ACAGATCAGT

Zebra_Finch TAGAAGATTA AAAGAAACGC AGGATGGTTT TGAGTTTCTC ACAGATCAAT

Chicken TAGAAGGTTG AAAGAGACAC AGGATGGGTT TGAATTTCTT ACAGATCAGT

Zebrafish TAGGAAGATG CGTGAAACTC AAGATGGCTT TGAGTTCTTT TCCGATCAGT

Fugu GCGGAAAATG AAGACCAGCC AGAACGGGTT CACCTTTTTC TCCAATCGGT

Human CCTACATTGA TGTTTTACCT GAGTTCAGAG ATTCTTACCC CATTAAGTAT

Gorilla CCTACATTGA TGTTTTACCT GAGTTCAGAG ATTCTTACCC CATTAAATAT

Orangutan CCTACATTGA TGTTTTACCT GAGTTCAGAG ATTCTTACCC CATTAAGTAT

Chimpanzee CCTACATTGA TGTTTTACCT GAGTTCAGAG ATTCTTACCC CATTAAGTAT

Marmoset CCTACGTTGA TGTTTTACCT GAGTTCAGAG ATTCTTACCC CATTAAGTAC

Dog CTTACATTGA TGTTCTACCG GAGTTCAGAG ACTCCTACCC CATTAAATAT

Mouse CCTATATTGA TGTCTTACCA GAATTCCAAG ATTCCTACCC CATAAAGTAC

Rabbit CCTATATTGA TGTCCTACCC GAGTTCCGAG ATTCCTACCC CATTAAGTAT

Guinea_Pig CCTATATTGA TGTTTTACCT GAGTTCCGAG ATTCTTACCC CATTAAGTAC

Horse CCTATATTGA TGTTCTACCT GAGTTCCGAG ATTCTTACCC CATTAAGTAT

Pig CCTATATTGA TGTTCTACCT GAGTTCCGAG ATTCCTACCC CATTAAGTAT

Cow CCTATATTGA TGTTCTACCT GAACTCCGAG ATTCCTACCC CATTAAGTAT

Rat CCTACATTGA TGTCCTGCCA GAATTCCGAG ATTCCTACCC CATCAAGTAC

Elephant CCTATATTGA TGTTCTACCT GAGTTCCGAG ATTCGTACCC CATTAAGTAT

Opossum CCTATATTGA TGTTCTACCA GAATTCCAAG ATTCTTACCC CATTAAATAC

Platypus ---------- ---------- ---------- ---------- ----------

Frog CATACATAGA TGTACTACCC CAGCTCAGAG ATATATATCC CATTAAATAT

Zebra_Finch CTTATATAGA TATCCTCCCT CAGTTCCGTG ACTCATATCC TATTAGGTAT

Chicken CTTATATAGA TATCCTCCCT CAGTTCCGCG ACTCGTATCC CATTAAGTAT

Zebrafish CCTACATGGA TTTGGCCCCT TCACTGCGGG GGAACTATCC ACTACATTAT

Fugu CATACATGGA CCTGATCCCC CCCCTCCGCG GGAGCTACTA CCTGCGATAC

Human GTCCATGCCT TTGAAAGCAA CAATTTTATT TACTTCTTGA CGGTCCAAAG

Gorilla GTCCATGCCT TTGAAAGCAA CAATTTTATT TACTTCTTGA CGGTCCAAAG

Orangutan GTCCATGCCT TTGAAAGCAA CAATTTTATT TACTTCTTGA CGGTCCAAAG

Chimpanzee GTCCATGCCT TTGAAAGCAA CAATTTTATT TACTTCTTGA CGGTCCAAAG

Marmoset GTCCATGCCT TTGAAAGCAA CAATTTTATT TACTTCTTGA CGGTCCAAAG

Dog GTCCACGCCT TTGAAAGCAA CCACTTTATT TACTTTTTGA CAGTCCAGCG

Mouse ATACATGCCT TCGAAAGCAA CCATTTTATT TACTTTCTGA CTGTCCAAAA

Rabbit GTCCACGCTT TTGAAAGCAA CCATTTTATT TACTTCCTGA CGGTCCAAAA

Guinea_Pig GTCCATGCCT TTGAAAGCAG CCATTTCATT TACTTTCTAA CTGTCCAAAA

Horse ATCCACGCCT TTGAAAGCAA CCATTTTATT TACTTTTTAA CGGTCCAACG

Pig GTCCATGCCT TTGAAAGCAA CCATTTTATT TACTTTTTGA CAGTCCAACG

Cow GTCCACGCCT TTGAAAGCAA CCATTTTATT TACTTTTTGA CGGTCCAGCG

Rat ATACATGCCT TCGAAAGCAA CCATTTTATC TACTTTCTGA CTGTCCAGAA

Elephant GTCCATGCCT TCAAACACAA CCAGTTTATT TACTTTTTGA CTGTCCAACG

Opossum ATACATGCCT TTGAAAGCAG TGACTTCATT TACTTTTTAA CAGTCCAAAG

Platypus ---------- ---------- ---------- ---------- ----------

Frog ATTTATACAT TTGAAAGTAA CAATTTTGTT TACTTTTTAA CTGTACAGCG

Zebra_Finch GTCCATGCCT TTGAAAACGA TCACTTTGTC TATTTTTTGA CTGTACAGAG

Chicken GTCCATGCCT TTGAGCATGA CCACTTTGTC TATTTTTTGA CTGTCCAGAG

Zebrafish GTCTACTCTT TCCAGAGTGG TCCTTATGTA TATTTTCTCA CCGTCCAACG

Fugu GTCTACTCCT TCCACAGCGG GCCGTTCACC TACTTCCTCA CCGTGCAGCA

Human GGAAACTCTA GATGCTCAGA CTTTTCACAC AAGAATAATC AGGTTCTGTT

Gorilla GGAAACTCTA GATGCTCAGA CTTTTCACAC AAGAATAATC AGGTTCTGTT

Orangutan GGAAACTCTA GATGCTCAGA CTTTTCACAC AAGAATAATC AGGTTCTGTT

Chimpanzee GGAAACTCTA GATGCTCAGA CTTTTCACAC AAGAATAATC AGGTTCTGTT

Marmoset GGAAACTCTA AATGCTCAGA CTTTTCACAC AAGAATAATC AGGTTCTGTT

Dog AGAAACTCTA GATGCTCAGA CTTTTCACAC GAGAATAATC AGGTTCTGTT

Mouse GGAAACTCTA GATGCTCAGA CTTTTCATAC AAGAATAATC AGGTTCTGTT

Rabbit GGAAACTCTA GATGCTCAGA CTTTCCACAC AAGAATCATC AGGTTCTGTT

Guinea_Pig GGAAACTCTG GATGCTCAGA CTTTTCACAC AAGAATCATC AGGTTCTGTT

Horse GGAAACTCTA GATGCTCAGA CTTTTCACAC AAGAATAATC AGGTTCTGTT

Pig GGAAACTCTC GACGCTCAGA CTTTTCACAC AAGAATAATC AGGTTCTGTT

Cow GGAAACTCTA GATGCTCAGA CTTTTCACAC AAGAATAATC AGGTTCTGTT

Rat GGAAACCCTA GATGCTCAGA CTTTCCATAC AAGAATAATC AGGTTCTGTT

Elephant GGAAACTCTA GAATCTCAGA CCTTTCACAC AAGAATCATC AGGTTCTGTT

Opossum AGAAACTCTG GAATCTCAGA CTTATCACAC AAGAATAATC AGGTTCTGTT

Platypus ---------- ---------- ---------- ---------- ----------

Frog AGAGTCCATT GACTCTCAGG CCTATCACAC TAGAATTGTG AGGGTCTGTT

Zebra_Finch AGAAACTCTT GACTCACAAT CTTTTCACAC TAGAATTATC CGCTTCTGCA

Chicken AGAATCTCTT GACTCCCAAA CTTTTCACAC TAGAATTATT CGCTTCTGCA

Zebrafish CGAAGGTGGC AACTCGAAAG CTTTCCACAC GAGAATCGTA CGCATGTGTT

Fugu GGTGAGCAAG GACTCCCAGA CCTACCACAC GCGCATCGTG CGCATGTGCT

Human CCATAAACTC TGGATTGCAT TCCTACATGG AAATGCCTCT GGAGTGTATT

Gorilla CCATAAACTC TGGATTGCAT TCCTACATGG AAATGCCTCT GGAGTGTATT

Orangutan CCATAAACTC TGGATTGCAT TCCTACATGG AAATGCCTCT GGAGTGTATT

Chimpanzee CCATAAACTC TGGATTGCAT TCCTACATGG AAATGCCTCT GGAGTGTATT

Marmoset CCATAAACTC TGCATTGCAT TCCTACATGG AAATGCCTCT GGAGTGTATT

Dog CTGTAGACTC TGGATTGCAT TCCTACATGG AAATGCCTCT GGAGTGTATT

Mouse CCGTAGACTC TGGGTTGCAC TCCTACATGG AAATGCCCCT GGAATGCATC

Rabbit CCAAGGACTC CGGCTTGCAT TCCTACATGG AAATGCCTCT GGAGTGTATT

Guinea_Pig CTGCAGACTC TGGACTGCAC TCGTACATGG AAATGCCTCT GGAGTGTATT

Horse CCGTGGACTC TGGATTGCAT TCCTACATGG AAATGCCTCT GGAGTGTATT

Pig CTGTAGACTC TGGATTGCAT TCCTACATGG AAATGCCTCT GGAGTGTATT

Cow CCGCAGACTC TGGATTGCAT TCGTACATGG AAATGCCTCT GGAGTGTATT

Rat CTGTAGACTC TGGGTTGCAC TCCTACATGG AAATGCCTCT GGAGTGCATT

Elephant CTGTAGACTC TGGATTGCAT TCCTACATGG AAATGCCTCT GGAGTGTATT
[truncated: 1,240,947 more chars]
