# Supplementary material for: Colon cancer associated genes exhibit signatures of positive selection at functionally significant positions
Source: BMC Evol Biol. 2012 Jul 12;12:114. doi: 10.1186/1471-2148-12-114 (PMC3563467; doi:10.1186/1471-2148-12-114)
Supplement: Additional file 3 — Likelihood ratio tests performed and their associated significance values. [file 1471-2148-12-114-S3.doc]

**Supplementary File 3: Likelihood ratio tests performed and their associated significance values.**

| Comparison | *df* | *Dl* | Critical χ^2^ values |
| --- | --- | --- | --- |
| M0 v M3k2 | 2 | X2 | ≥ 5.99 |
| M3k2 v M3k3 | - | X1 | ≥ 1.00 |
| M1 v M2 | 2 | X2 | ≥ 5.99 |
| M7 v M8 | 2 | X2 | ≥ 5.99 |
| M8 v M8a^1^ | 1 | X2 | ≥ 2.71 (@ 5%) |
|  |  |  | ≥ 5.41 (@ 1%) |
| M1 v Model A | 2 | X2 | ≥ 5.99 |
| Model A v Model A1^2^ | 1 | X2 | ≥ 3.84 (@ 5%) |
