# Supplementary material for: Colon cancer associated genes exhibit signatures of positive selection at functionally significant positions
Source: BMC Evol Biol. 2012 Jul 12;12:114. doi: 10.1186/1471-2148-12-114 (PMC3563467; doi:10.1186/1471-2148-12-114)
Supplement: Additional file 4 — Full set of models, associated likelihood scores and parameter estimates for all genes in the colon cancer gene dataset. This information is given alphabetically on a gene-by-gene basis. All estimated parameters, Likelihood values and BEB or NEB sites are listed. [file 1471-2148-12-114-S4.doc]

| **Model** | **Tree** | **Model Type** | **p** | **w (t=0)** | **lnL** | **LRT Result** | **Parameter Estimates** | **Positive Selection** | **Positively Selected Sites** |
| --- | --- | --- | --- | --- | --- | --- | --- | --- | --- |
| **CDH1 Site Analysis** | | | | | | | | | |
| m0 | Sites | Homogeneous | 1 | 2 | -17243.15855 | N/A | w=0.18208 | No |  |
| m1Neutral | Sites | Site-specific | 1 | 2 | -16662.7682 | N/A | p0=0.75935 p1=0.24065 w0=0.05698 w1=1.00000 | Not Allowed |  |
| m2Selection | Sites | Site-specific | 2 | 10 | -16651.32597 | m2Selection | p0=0.75679 p1=0.23783 p2=0.00537 w0=0.05722 w1=1.00000 w2=6.66719 | Yes | Alignment (7 BEB sites): 132 392 553 657 684 699 700 |
| m3Discrtk2 | Sites | Site-specific | 3 | 2 | -16635.71746 | m3Discrtk2 | p0=0.70519 p1=0.29481 w0=0.03699 w1=0.66288 | No |  |
| m3Discrtk3 | Sites | Site-specific | 5 | 2 | -16597.32098 | m3Discrtk3 | p0=0.60001 p1=0.28812 p2=0.11187 w0=0.01830 w1=0.33215 w2=1.12920 | Yes | Alignment (91 NEB sites): 4 8 20 29 56 64 71 78 89 90 96 99 107 118 119 120 125 127 129 131 132 134 135 136 137 141 144 145 147 174 224 231 240 265 268 276 285 293 303 310 311 314 315 321 322 340 351 366 368 379 381 383 392 395 410 416 420 421 430 457 460 465 467 478 503 511 518 529 553 562 574 584 586 599 611 645 646 657 660 670 684 691 693 696 698 699 700 701 732 736 845 |
| m7 | Sites | Site-specific | 2 | 2 | -16604.06254 | N/A | p=0.20752 q=0.72855 | Not Allowed |  |
| m8 | Sites | Site-specific | 4 | 2 | -16589.88768 | m8 | p=0.21848 p0=0.99291 p1=0.00709 q=0.80842 w=4.53766 | Yes | Alignment (15 BEB sites): 132 137 224 392 421 457 465 467 553 645 657 684 699 700 736 |
| m8a | Sites | Site-specific | 4 | 1 | -16598.1084 | N/A | p=0.28844 p0=0.88884 p1=0.11116 q=1.94552 w=1.00000 | Not Allowed |  |
| **CDH1 Branch-site Analysis** | | | | | | | | | |
| modelA | Chimpanzee | Model A | 3 | 2 | -16662.7682 | m1Neutral, modelAnull | p0=0.75936 p1=0.24064 p2=0.00000 p3=0.00000 w0=0.05698 w1=1.00000 w2=1.00000 | No |  |
| modelAnull | Chimpanzee | Model A | 3 | 1 | -16662.7682 | N/A | p0=0.75936 p1=0.24064 p2=0.00000 p3=0.00000 w0=0.05698 w1=1.00000 w2=1.00000 | Not Allowed |  |
| modelA | Euarchontoglires | Branch-site | 3 | 2 | -16662.7682 | m1Neutral, modelAnull | p0=0.75935 p1=0.24065 p2=0.00000 p3=0.00000 w0=0.05698 w1=1.00000 w2=1.00000 | No |  |
| modelAnull | Euarchontoglires | Branch-site | 3 | 1 | -16662.7682 | N/A | p0=0.75935 p1=0.24065 p2=0.00000 p3=0.00000 w0=0.05698 w1=1.00000 w2=1.00000 | Not Allowed |  |
| modelA | Glires | Branch-site | 3 | 2 | -16662.7682 | m1Neutral, modelAnull | p0=0.75936 p1=0.24064 p2=0.00000 p3=0.00000 w0=0.05698 w1=1.00000 w2=1.00000 | No |  |
| modelAnull | Glires | Branch-site | 3 | 1 | -16662.7682 | N/A | p0=0.75936 p1=0.24064 p2=0.00000 p3=0.00000 w0=0.05698 w1=1.00000 w2=1.00000 | Not Allowed |  |
| modelA | Gorilla | Branch-site | 3 | 2 | -16662.76755 | m1Neutral, modelAnull | p0=0.75703 p1=0.23990 p2=0.00233 p3=0.00074 w0=0.05697 w1=1.00000 w2=1.00000 | No |  |
| modelAnull | Gorilla | Branch-site | 3 | 1 | -16662.76755 | N/A | p0=0.75702 p1=0.23990 p2=0.00234 p3=0.00074 w0=0.05697 w1=1.00000 w2=1.00000 | Not Allowed |  |
| modelA | Guinea_Pig | Branch-site | 3 | 1 | -16660.57303 | m1Neutral, modelAnull | p0=0.75690 p1=0.23739 p2=0.00434 p3=0.00136 w0=0.05711 w1=1.00000 w2=94.39978 | No |  |
| modelAnull | Guinea_Pig | Branch-site | 3 | 1 | -16661.80885 | N/A | p0=0.73906 p1=0.23224 p2=0.02184 p3=0.00686 w0=0.05594 w1=1.00000 w2=1.00000 | Not Allowed |  |
| modelA | Homindae | Branch-site | 3 | 2 | -16661.18449 | m1Neutral, modelAnull | p0=0.73467 p1=0.23307 p2=0.02449 p3=0.00777 w0=0.05588 w1=1.00000 w2=2.86495 | No |  |
| modelAnull | Homindae | Branch-site | 3 | 1 | -16661.35289 | N/A | p0=0.70607 p1=0.22407 p2=0.05303 p3=0.01683 w0=0.05590 w1=1.00000 w2=1.00000 | Not Allowed |  |
| modelA | Homininae | Branch-site | 3 | 2 | -16662.7682 | m1Neutral, modelAnull | p0=0.75935 p1=0.24065 p2=0.00000 p3=0.00000 w0=0.05698 w1=1.00000 w2=1.00000 | No |  |
| modelAnull | Homininae | Branch-site | 3 | 1 | -16662.7682 | N/A | p0=0.75936 p1=0.24064 p2=0.00000 p3=0.00000 w0=0.05698 w1=1.00000 w2=1.00000 | Not Allowed |  |
| modelA | Hominini | Branch-site | 3 | 2 | -16662.7682 | m1Neutral, modelAnull | p0=0.75935 p1=0.24065 p2=0.00000 p3=0.00000 w0=0.05698 w1=1.00000 w2=1.00000 | No |  |
| modelAnull | Hominini | Branch-site | 3 | 1 | -16662.76821 | N/A | p0=0.75935 p1=0.24064 p2=0.00001 p3=0.00000 w0=0.05698 w1=1.00000 w2=1.00000 | Not Allowed |  |
| modelA | Human | Branch-site | 3 | 2 | -16662.7682 | m1Neutral, modelAnull | p0=0.75935 p1=0.24065 p2=0.00000 p3=0.00000 w0=0.05698 w1=1.00000 w2=1.00000 | No |  |
| modelAnull | Human | Branch-site | 3 | 1 | -16662.7682 | N/A | p0=0.75935 p1=0.24065 p2=0.00000 p3=0.00000 w0=0.05698 w1=1.00000 w2=1.00000 | Not Allowed |  |
| modelA | Marmoset | Branch-site | 3 | 2 | -16662.7682 | m1Neutral, modelAnull | p0=0.75935 p1=0.24065 p2=0.00000 p3=0.00000 w0=0.05698 w1=1.00000 w2=1.00000 | No |  |
| modelAnull | Marmoset | Branch-site | 3 | 1 | -16662.7682 | N/A | p0=0.75935 p1=0.24065 p2=0.00000 p3=0.00000 w0=0.05698 w1=1.00000 w2=1.00000 | Not Allowed |  |
| modelA | Mouse | Branch-site | 3 | 10 | -16662.64982 | m1Neutral, modelAnull | p0=0.75885 p1=0.23957 p2=0.00120 p3=0.00038 w0=0.05699 w1=1.00000 w2=7.17133 | No |  |
| modelAnull | Mouse | Branch-site | 3 | 1 | -16662.7682 | N/A | p0=0.75936 p1=0.24064 p2=0.00000 p3=0.00000 w0=0.05698 w1=1.00000 w2=1.00000 | Not Allowed |  |
| modelA | Muridae | Branch-site | 3 | 2 | -16662.7682 | m1Neutral, modelAnull | p0=0.75935 p1=0.24065 p2=0.00000 p3=0.00000 w0=0.05698 w1=1.00000 w2=1.00000 | No |  |
| modelAnull | Muridae | Branch-site | 3 | 1 | -16662.7682 | N/A | p0=0.75935 p1=0.24065 p2=0.00000 p3=0.00000 w0=0.05698 w1=1.00000 w2=1.00000 | Not Allowed |  |
| modelA | Murinae | Branch-site | 3 | 2 | -16661.19237 | m1Neutral, modelAnull | p0=0.75765 p1=0.23772 p2=0.00353 p3=0.00111 w0=0.05666 w1=1.00000 w2=10.17290 | No |  |
| modelAnull | Murinae | Branch-site | 3 | 1 | -16662.636 | N/A | p0=0.75543 p1=0.23860 p2=0.00454 p3=0.00143 w0=0.05671 w1=1.00000 w2=1.00000 | Not Allowed |  |
| modelA | Orangutan | Branch-site | 3 | 2 | -16662.74055 | m1Neutral, modelAnull | p0=0.74947 p1=0.23753 p2=0.00987 p3=0.00313 w0=0.05690 w1=1.00000 w2=1.00000 | No |  |
| modelAnull | Orangutan | Branch-site | 3 | 1 | -16662.74055 | N/A | p0=0.74946 p1=0.23753 p2=0.00988 p3=0.00313 w0=0.05690 w1=1.00000 w2=1.00000 | Not Allowed |  |
| modelA | Primates | Branch-site | 3 | 2 | -16658.03484 | modelA | p0=0.75454 p1=0.23453 p2=0.00834 p3=0.00259 w0=0.05683 w1=1.00000 w2=10.20516 | Yes | Alignment (9 BEB sites): 95 97 146 182 227 496 604 797 799 |
| modelAnull | Primates | Branch-site | 3 | 1 | -16660.14001 | N/A | p0=0.72212 p1=0.22637 p2=0.03922 p3=0.01229 w0=0.05618 w1=1.00000 w2=1.00000 | Not Allowed |  |
| modelA | Rabbit | Branch-site | 3 | 0 | -16662.7682 | m1Neutral, modelAnull | p0=0.75936 p1=0.24064 p2=0.00000 p3=0.00000 w0=0.05698 w1=1.00000 w2=1.00000 | No |  |
| modelAnull | Rabbit | Branch-site | 3 | 1 | -16662.7682 | N/A | p0=0.75935 p1=0.24065 p2=0.00000 p3=0.00000 w0=0.05698 w1=1.00000 w2=1.00000 | Not Allowed |  |
| modelA | Rat | Branch-site | 3 | 2 | -16662.23384 | m1Neutral, modelAnull | p0=0.73298 p1=0.23287 p2=0.02592 p3=0.00823 w0=0.05604 w1=1.00000 w2=1.00000 | No |  |
| modelAnull | Rat | Branch-site | 3 | 1 | -16662.23384 | N/A | p0=0.73297 p1=0.23287 p2=0.02592 p3=0.00823 w0=0.05604 w1=1.00000 w2=1.00000 | Not Allowed |  |
| **PMS1 Site Analysis** | | | | | | | | | |
| m1Neutral | Sites | Site-specific | 1 | 2 | -26664.66425 | N/A | p0=0.63955 p1=0.36045 w0=0.11032 w1=1.00000 | Not Allowed |  |
| m2Selection | Sites | Site-specific | 2 | 2 | -26664.66425 | m1Neutral | p0=0.63955 p1=0.15979 p2=0.20066 w0=0.11032 w1=1.00000 w2=1.00000 | No |  |
| m3Discrtk2 | Sites | Site-specific | 3 | 2 | -26572.17096 | m3Discrtk2 | p0=0.53043 p1=0.46957 w0=0.06416 w1=0.52749 | No |  |
| m3Discrtk3 | Sites | Site-specific | 5 | 2 | -26493.05565 | m3Discrtk3 | p0=0.36830 p1=0.42797 p2=0.20373 w0=0.03242 w1=0.25791 w2=0.88276 | No |  |
| m7 | Sites | Site-specific | 2 | 2 | -26493.11028 | N/A | p=0.52291 q=1.27718 | Not Allowed |  |
| m8 | Sites | Site-specific | 4 | 2 | -26480.39761 | m8 | p=0.61337 p0=0.93580 p1=0.06420 q=1.93110 w=1.32691 | Yes | Alignment (25 BEB sites): 249 346 387 405 413 417 433 438 440 444 449 451 453 458 462 465 467 471 473 491 525 529 660 691 692 |
| m8a | Sites | Site-specific | 4 | 1 | -26482.71773 | N/A | p=0.66085 p0=0.88746 p1=0.11254 q=2.47031 w=1.00000 | Not Allowed |  |
| **PMS1 Branch-site Analysis** | | | | | | | | | |
| modelA | Chimpanzee | Branch-site | 3 | 2 | -26663.68782 | m1Neutral, modelAnull | p0=0.00004 p1=0.00002 p2=0.63928 p3=0.36066 w0=0.11010 w1=1.00000 w2=1.00000 | No |  |
| modelAnull | Chimpanzee | Branch-site | 3 | 1 | -26663.68782 | N/A | p0=0.00004 p1=0.00002 p2=0.63928 p3=0.36066 w0=0.11010 w1=1.00000 w2=1.00000 | Not Allowed |  |
| modelA | Euarchontoglires | Branch-site | 3 | 2 | -26664.62481 | m1Neutral, modelAnull | p0=0.61447 p1=0.34618 p2=0.02517 p3=0.01418 w0=0.11024 w1=1.00000 w2=1.00000 | No |  |
| modelAnull | Euarchontoglires | Branch-site | 3 | 1 | -26664.62481 | N/A | p0=0.61449 p1=0.34619 p2=0.02515 p3=0.01417 w0=0.11024 w1=1.00000 w2=1.00000 | Not Allowed |  |
| modelA | Glires | Branch-site | 3 | 2 | -26664.61793 | m1Neutral, modelAnull | p0=0.61632 p1=0.34751 p2=0.02313 p3=0.01304 w0=0.11019 w1=1.00000 w2=1.00000 | No |  |
| modelAnull | Glires | Branch-site | 3 | 1 | -26664.61793 | N/A | p0=0.61632 p1=0.34751 p2=0.02313 p3=0.01304 w0=0.11019 w1=1.00000 w2=1.00000 | Not Allowed |  |
| modelA | Gorilla | Branch-site | 3 | 2 | -26664.16347 | m1Neutral, modelAnull | p0=0.59899 p1=0.33708 p2=0.04091 p3=0.02302 w0=0.11017 w1=1.00000 w2=4.27841 | No |  |
| modelAnull | Gorilla | Branch-site | 3 | 1 | -26664.2751 | N/A | p0=0.51026 p1=0.28720 p2=0.12960 p3=0.07295 w0=0.11018 w1=1.00000 w2=1.00000 | Not Allowed |  |
| modelA | Guinea_Pig | Branch-site | 3 | 2 | -26663.96803 | m1Neutral, modelAnull | p0=0.63738 p1=0.35778 p2=0.00310 p3=0.00174 w0=0.10992 w1=1.00000 w2=5.80921 | No |  |
| modelAnull | Guinea_Pig | Branch-site | 3 | 1 | -26664.54985 | N/A | p0=0.63345 p1=0.35671 p2=0.00630 p3=0.00355 w0=0.11003 w1=1.00000 w2=1.00000 | Not Allowed |  |
| modelA | Homindae | Branch-site | 3 | 2 | -26664.66425 | m1Neutral, modelAnull | p0=0.63955 p1=0.36045 p2=0.00000 p3=0.00000 w0=0.11032 w1=1.00000 w2=1.00000 | No |  |
| modelAnull | Homindae | Branch-site | 3 | 1 | -26664.66425 | N/A | p0=0.63955 p1=0.36045 p2=0.00000 p3=0.00000 w0=0.11032 w1=1.00000 w2=1.00000 | Not Allowed |  |
| modelA | Homininae | Branch-site | 3 | 2 | -26664.66425 | m1Neutral, modelAnull | p0=0.63955 p1=0.36045 p2=0.00000 p3=0.00000 w0=0.11032 w1=1.00000 w2=1.00000 | No |  |
| modelAnull | Homininae | Branch-site | 3 | 1 | -26664.66425 | N/A | p0=0.63955 p1=0.36045 p2=0.00000 p3=0.00000 w0=0.11032 w1=1.00000 w2=1.00000 | Not Allowed |  |
| modelA | Hominini | Branch-site | 3 | 2 | -26664.66425 | m1Neutral, modelAnull | p0=0.63955 p1=0.36045 p2=0.00000 p3=0.00000 w0=0.11032 w1=1.00000 w2=1.00000 | No |  |
| modelAnull | Hominini | Branch-site | 3 | 1 | -26664.66425 | N/A | p0=0.63955 p1=0.36045 p2=0.00000 p3=0.00000 w0=0.11032 w1=1.00000 w2=1.00000 | Not Allowed |  |
| modelA | Human | Branch-site | 3 | 2 | -26662.85014 | m1Neutral, modelAnull | p0=0.00926 p1=0.00522 p2=0.63019 p3=0.35533 w0=0.10995 w1=1.00000 w2=1.00000 | No |  |
| modelAnull | Human | Branch-site | 3 | 1 | -26662.85017 | N/A | p0=0.00317 p1=0.00179 p2=0.63628 p3=0.35877 w0=0.10995 w1=1.00000 w2=1.00000 | Not Allowed |  |
| modelA | Marmoset | Branch-site | 3 | 2 | -26664.2132 | m1Neutral, modelAnull | p0=0.59265 p1=0.33257 p2=0.04791 p3=0.02688 w0=0.10999 w1=1.00000 w2=1.00000 | No |  |
| modelAnull | Marmoset | Branch-site | 3 | 1 | -26664.2132 | N/A | p0=0.59265 p1=0.33256 p2=0.04791 p3=0.02688 w0=0.10999 w1=1.00000 w2=1.00000 | Not Allowed |  |
| modelA | Mouse | Branch-site | 3 | 0 | -26663.74579 | m1Neutral, modelAnull | p0=0.63359 p1=0.35020 p2=0.01044 p3=0.00577 w0=0.10988 w1=1.00000 w2=2.35701 | No |  |
| modelAnull | Mouse | Branch-site | 3 | 1 | -26663.71155 | N/A | p0=0.61778 p1=0.34187 p2=0.02597 p3=0.01437 w0=0.10945 w1=1.00000 w2=1.00000 | Not Allowed |  |
| modelA | Muridae | Branch-site | 3 | 2 | -26664.66425 | m1Neutral, modelAnull | p0=0.63955 p1=0.36045 p2=0.00000 p3=0.00000 w0=0.11032 w1=1.00000 w2=1.00000 | No |  |
| modelAnull | Muridae | Branch-site | 3 | 1 | -26664.66425 | N/A | p0=0.63955 p1=0.36045 p2=0.00000 p3=0.00000 w0=0.11032 w1=1.00000 w2=1.00000 | Not Allowed |  |
| modelA | Orangutan | Branch-site | 3 | 2 | -26662.42683 | m1Neutral, modelAnull | p0=0.41325 p1=0.23239 p2=0.22681 p3=0.12755 w0=0.10979 w1=1.00000 w2=1.00000 | No |  |
| modelAnull | Orangutan | Branch-site | 3 | 1 | -26662.42683 | N/A | p0=0.41326 p1=0.23240 p2=0.22680 p3=0.12754 w0=0.10979 w1=1.00000 w2=1.00000 | Not Allowed |  |
| modelA | Primates | Branch-site | 3 | 2 | -26664.66425 | m1Neutral, modelAnull | p0=0.63955 p1=0.36045 p2=0.00000 p3=0.00000 w0=0.11032 w1=1.00000 w2=1.00000 | No |  |
| modelAnull | Primates | Branch-site | 3 | 1 | -26664.66425 | N/A | p0=0.63955 p1=0.36045 p2=0.00000 p3=0.00000 w0=0.11032 w1=1.00000 w2=1.00000 | Not Allowed |  |
| modelA | Rabbit | Branch-site | 3 | 10 | -26662.39566 | m1Neutral | p0=0.63939 p1=0.35695 p2=0.00235 p3=0.00131 w0=0.11070 w1=1.00000 w2=998.99997 | No |  |
| modelAnull | Rabbit | Branch-site | 3 | 1 | -26664.66425 | N/A | p0=0.63955 p1=0.36045 p2=0.00000 p3=0.00000 w0=0.11032 w1=1.00000 w2=1.00000 | Not Allowed |  |
| **MLH1 Site Analysis** | | | | | | | | | |
| m0 | Sites | Homogeneous | 1 | 2 | -20275.39659 | N/A | w=0.11677 | No |  |
| m1Neutral | Sites | Site-specific | 1 | 2 | -19522.86098 | N/A | p0=0.81231 p1=0.18769 w0=0.05365 w1=1.00000 | Not Allowed |  |
| m2Selection | Sites | Site-specific | 2 | 2 | -19522.86098 | m1Neutral | p0=0.81231 p1=0.10181 p2=0.08589 w0=0.05365 w1=1.00000 w2=1.00000 | No |  |
| m3Discrtk2 | Sites | Site-specific | 3 | 2 | -19455.08453 | m3Discrtk2 | p0=0.76492 p1=0.23508 w0=0.03748 w1=0.51375 | No |  |
| m3Discrtk3 | Sites | Site-specific | 5 | 2 | -19368.04517 | m3Discrtk3 | p0=0.53642 p1=0.31707 p2=0.14651 w0=0.01196 w1=0.13919 w2=0.74033 | No |  |
| m7 | Sites | Site-specific | 2 | 2 | -19381.94781 | N/A | p=0.26279 q=1.34867 | Not Allowed |  |
| m8 | Sites | Site-specific | 4 | 2 | -19358.60145 | m8a | p=0.38951 p0=0.91021 p1=0.08979 q=4.00954 w=1.00000 | No |  |
| m8a | Sites | Site-specific | 4 | 1 | -19358.60145 | N/A | p=0.38951 p0=0.91021 p1=0.08979 q=4.00952 w=1.00000 | Not Allowed |  |
| **MLH1 Branch-site Analysis** | | | | | | | | | |
| modelA | Chimpanzee | Branch-site | 3 | 2 | -19522.86098 | m1Neutral, modelAnull | p0=0.81230 p1=0.18770 p2=0.00000 p3=0.00000 w0=0.05365 w1=1.00000 w2=1.00000 | No |  |
| modelAnull | Chimpanzee | Branch-site | 3 | 1 | -19522.86098 | N/A | p0=0.81231 p1=0.18769 p2=0.00000 p3=0.00000 w0=0.05365 w1=1.00000 w2=1.00000 | Not Allowed |  |
| modelA | Euarchontoglires | Branch-site | 3 | 2 | -19522.83026 | m1Neutral, modelAnull | p0=0.79573 p1=0.18388 p2=0.01656 p3=0.00383 w0=0.05361 w1=1.00000 w2=1.00000 | No |  |
| modelAnull | Euarchontoglires | Branch-site | 3 | 1 | -19522.83026 | N/A | p0=0.79573 p1=0.18388 p2=0.01656 p3=0.00383 w0=0.05361 w1=1.00000 w2=1.00000 | Not Allowed |  |
| modelA | Glires | Branch-site | 3 | 2 | -19522.86098 | m1Neutral, modelAnull | p0=0.81231 p1=0.18769 p2=0.00000 p3=0.00000 w0=0.05365 w1=1.00000 w2=1.00000 | No |  |
| modelAnull | Glires | Branch-site | 3 | 1 | -19522.86098 | N/A | p0=0.81231 p1=0.18769 p2=0.00000 p3=0.00000 w0=0.05365 w1=1.00000 w2=1.00000 | Not Allowed |  |
| modelA | Gorilla | Branch-site | 3 | 2 | -19522.86098 | m1Neutral, modelAnull | p0=0.81231 p1=0.18769 p2=0.00000 p3=0.00000 w0=0.05365 w1=1.00000 w2=1.00000 | No |  |
| modelAnull | Gorilla | Branch-site | 3 | 1 | -19522.86098 | N/A | p0=0.81231 p1=0.18769 p2=0.00000 p3=0.00000 w0=0.05365 w1=1.00000 w2=1.00000 | Not Allowed |  |
| modelA | Guinea_Pig | Branch-site | 3 | 2 | -19521.87028 | m1Neutral, modelAnull | p0=0.80787 p1=0.18594 p2=0.00503 p3=0.00116 w0=0.05329 w1=1.00000 w2=2.85365 | No |  |
| modelAnull | Guinea_Pig | Branch-site | 3 | 1 | -19522.18867 | N/A | p0=0.80222 p1=0.18481 p2=0.01054 p3=0.00243 w0=0.05328 w1=1.00000 w2=1.00000 | Not Allowed |  |
| modelA | Homindae | Branch-site | 3 | 2 | -19522.86098 | m1Neutral, modelAnull | p0=0.81231 p1=0.18769 p2=0.00000 p3=0.00000 w0=0.05365 w1=1.00000 w2=1.00000 | No |  |
| modelAnull | Homindae | Branch-site | 3 | 1 | -19522.86098 | N/A | p0=0.81231 p1=0.18769 p2=0.00000 p3=0.00000 w0=0.05365 w1=1.00000 w2=1.00000 | Not Allowed |  |
| modelA | Homininae | Branch-site | 3 | 2 | -19522.86098 | m1Neutral, modelAnull | p0=0.81231 p1=0.18769 p2=0.00000 p3=0.00000 w0=0.05365 w1=1.00000 w2=1.00000 | No |  |
| modelAnull | Homininae | Branch-site | 3 | 1 | -19522.86098 | N/A | p0=0.81231 p1=0.18769 p2=0.00000 p3=0.00000 w0=0.05365 w1=1.00000 w2=1.00000 | Not Allowed |  |
| modelA | Hominini | Branch-site | 3 | 2 | -19522.86098 | m1Neutral, modelAnull | p0=0.81231 p1=0.18769 p2=0.00000 p3=0.00000 w0=0.05365 w1=1.00000 w2=1.00000 | No |  |
| modelAnull | Hominini | Branch-site | 3 | 1 | -19522.86098 | N/A | p0=0.81231 p1=0.18769 p2=0.00000 p3=0.00000 w0=0.05365 w1=1.00000 w2=1.00000 | Not Allowed |  |
| modelA | Human | Branch-site | 3 | 2 | -19522.31458 | m1Neutral, modelAnull | p0=0.66852 p1=0.15444 p2=0.14381 p3=0.03322 w0=0.05355 w1=1.00000 w2=1.01313 | No |  |
| modelAnull | Human | Branch-site | 3 | 1 | -19522.31458 | N/A | p0=0.66669 p1=0.15402 p2=0.14564 p3=0.03365 w0=0.05355 w1=1.00000 w2=1.00000 | Not Allowed |  |
| modelA | Mouse | Branch-site | 3 | 2 | -19522.37051 | m1Neutral, modelAnull | p0=0.81123 p1=0.18739 p2=0.00112 p3=0.00026 w0=0.05354 w1=1.00000 w2=700.58372 | No |  |
| modelAnull | Mouse | Branch-site | 3 | 1 | -19522.86098 | N/A | p0=0.81231 p1=0.18769 p2=0.00000 p3=0.00000 w0=0.05365 w1=1.00000 w2=1.00000 | Not Allowed |  |
| modelA | Muridae | Branch-site | 3 | 2 | -19522.71014 | m1Neutral, modelAnull | p0=0.77930 p1=0.18009 p2=0.03298 p3=0.00762 w0=0.05353 w1=1.00000 w2=1.00000 | No |  |
| modelAnull | Muridae | Branch-site | 3 | 1 | -19522.71014 | N/A | p0=0.77930 p1=0.18009 p2=0.03299 p3=0.00762 w0=0.05353 w1=1.00000 w2=1.00000 | Not Allowed |  |
| modelA | Murinae | Branch-site | 3 | 2 | -19522.86098 | m1Neutral, modelAnull | p0=0.81231 p1=0.18769 p2=0.00000 p3=0.00000 w0=0.05365 w1=1.00000 w2=1.00000 | No |  |
| modelAnull | Murinae | Branch-site | 3 | 1 | -19522.86098 | N/A | p0=0.81231 p1=0.18769 p2=0.00000 p3=0.00000 w0=0.05365 w1=1.00000 w2=1.00000 | Not Allowed |  |
| modelA | Orangutan | Branch-site | 3 | 2 | -19521.6594 | m1Neutral, modelAnull | p0=0.71324 p1=0.16506 p2=0.09883 p3=0.02287 w0=0.05335 w1=1.00000 w2=1.00000 | No |  |
| modelAnull | Orangutan | Branch-site | 3 | 1 | -19521.6594 | N/A | p0=0.71325 p1=0.16506 p2=0.09882 p3=0.02287 w0=0.05335 w1=1.00000 w2=1.00000 | Not Allowed |  |
| modelA | Rabbit | Branch-site | 3 | 2 | -19516.63525 | modelA | p0=0.80595 p1=0.18541 p2=0.00703 p3=0.00162 w0=0.05262 w1=1.00000 w2=7.52747 | Yes | Alignment (5 BEB sites): 120 208 474 512 530 |
| modelAnull | Rabbit | Branch-site | 3 | 1 | -19519.40774 | N/A | p0=0.79297 p1=0.18289 p2=0.01961 p3=0.00452 w0=0.05255 w1=1.00000 w2=1.00000 | Not Allowed |  |
| modelA | Rat | Branch-site | 3 | 2 | -19522.86098 | m1Neutral, modelAnull | p0=0.81231 p1=0.18769 p2=0.00000 p3=0.00000 w0=0.05365 w1=1.00000 w2=1.00000 | No |  |
| modelAnull | Rat | Branch-site | 3 | 1 | -19522.86098 | N/A | p0=0.81231 p1=0.18769 p2=0.00000 p3=0.00000 w0=0.05365 w1=1.00000 w2=1.00000 | Not Allowed |  |
| **MSH2 Site Analysis** | | | | | | | | | |
| m0 | Sites | Homogeneous | 1 | 2 | -19717.73068 | N/A | w=0.08199 | No |  |
| m1Neutral | Sites | Site-specific | 1 | 2 | -19545.19149 | N/A | p0=0.92900 p1=0.07100 w0=0.06501 w1=1.00000 | Not Allowed |  |
| m2Selection | Sites | Site-specific | 2 | 2 | -19545.19149 | m1Neutral | p0=0.92900 p1=0.07100 p2=0.00000 w0=0.06501 w1=1.00000 w2=79.52689 | No |  |
| m3Discrtk2 | Sites | Site-specific | 3 | 2 | -19403.27471 | m3Discrtk2 | p0=0.67916 p1=0.32084 w0=0.02386 w1=0.23540 | No |  |
| m3Discrtk3 | Sites | Site-specific | 5 | 0 | -19374.73688 | m3Discrtk3 | p0=0.57173 p1=0.38927 p2=0.03900 w0=0.01493 w1=0.15668 w2=0.65842 | No |  |
| m7 | Sites | Site-specific | 2 | 2 | -19381.09343 | N/A | p=0.45854 q=4.17033 | Not Allowed |  |
| m8 | Sites | Site-specific | 4 | 2 | -19372.49435 | m8a | p=0.52785 p0=0.98497 p1=0.01503 q=5.63325 w=1.00000 | No |  |
| m8a | Sites | Site-specific | 4 | 1 | -19372.49435 | N/A | p=0.52785 p0=0.98497 p1=0.01503 q=5.63326 w=1.00000 | Not Allowed |  |
| **MSH2 Branch-site Analysis** | | | | | | | | | |
| modelA | Chimpanzee | Branch-site | 3 | 2 | -19545.19149 | m1Neutral, modelAnull | p0=0.92878 p1=0.07098 p2=0.00022 p3=0.00002 w0=0.06501 w1=1.00000 w2=1.00000 | No |  |
| modelAnull | Chimpanzee | Branch-site | 3 | 1 | -19545.19149 | N/A | p0=0.92868 p1=0.07098 p2=0.00031 p3=0.00002 w0=0.06501 w1=1.00000 w2=1.00000 | Not Allowed |  |
| modelA | Euarchontoglires | Branch-site | 3 | 10 | -19545.19149 | m1Neutral, modelAnull | p0=0.92900 p1=0.07100 p2=0.00000 p3=0.00000 w0=0.06501 w1=1.00000 w2=9.19601 | No |  |
| modelAnull | Euarchontoglires | Branch-site | 3 | 1 | -19545.19149 | N/A | p0=0.92900 p1=0.07100 p2=0.00000 p3=0.00000 w0=0.06501 w1=1.00000 w2=1.00000 | Not Allowed |  |
| modelA | Glires | Branch-site | 3 | 2 | -19544.49067 | m1Neutral, modelAnull | p0=0.79878 p1=0.06076 p2=0.13053 p3=0.00993 w0=0.06474 w1=1.00000 w2=1.00000 | No |  |
| modelAnull | Glires | Branch-site | 3 | 1 | -19544.49067 | N/A | p0=0.79878 p1=0.06076 p2=0.13053 p3=0.00993 w0=0.06474 w1=1.00000 w2=1.00000 | Not Allowed |  |
| modelA | Gorilla | Branch-site | 3 | 2 | -19485.4338 | modelA | p0=0.92233 p1=0.06298 p2=0.01375 p3=0.00094 w0=0.06427 w1=1.00000 w2=999.00000 | Yes | Alignment (15 BEB sites): 124 125 126 127 128 129 130 132 133 134 137 138 140 141 142 |
| modelAnull | Gorilla | Branch-site | 3 | 1 | -19534.23363 | N/A | p0=0.75791 p1=0.05277 p2=0.17700 p3=0.01232 w0=0.06397 w1=1.00000 w2=1.00000 | Not Allowed |  |
| modelA | Guinea_Pig | Branch-site | 3 | 0 | -19545.19149 | m1Neutral, modelAnull | p0=0.92900 p1=0.07100 p2=0.00000 p3=0.00000 w0=0.06501 w1=1.00000 w2=1.00000 | No |  |
| modelAnull | Guinea_Pig | Branch-site | 3 | 1 | -19545.1915 | N/A | p0=0.92900 p1=0.07100 p2=0.00000 p3=0.00000 w0=0.06501 w1=1.00000 w2=1.00000 | Not Allowed |  |
| modelA | Homindae | Branch-site | 3 | 2 | -19543.53063 | m1Neutral, modelAnull | p0=0.92210 p1=0.06880 p2=0.00847 p3=0.00063 w0=0.06483 w1=1.00000 w2=18.85777 | No |  |
| modelAnull | Homindae | Branch-site | 3 | 1 | -19544.28565 | N/A | p0=0.83706 p1=0.06342 p2=0.09251 p3=0.00701 w0=0.06464 w1=1.00000 w2=1.00000 | Not Allowed |  |
| modelA | Homininae | Branch-site | 3 | 1 | -19545.19149 | m1Neutral, modelAnull | p0=0.92900 p1=0.07100 p2=0.00000 p3=0.00000 w0=0.06501 w1=1.00000 w2=1.00000 | No |  |
| modelAnull | Homininae | Branch-site | 3 | 1 | -19545.19149 | N/A | p0=0.92900 p1=0.07100 p2=0.00000 p3=0.00000 w0=0.06501 w1=1.00000 w2=1.00000 | Not Allowed |  |
| modelA | Hominini | Branch-site | 3 | 2 | -19545.19149 | m1Neutral, modelAnull | p0=0.92900 p1=0.07100 p2=0.00000 p3=0.00000 w0=0.06501 w1=1.00000 w2=4.93289 | No |  |
| modelAnull | Hominini | Branch-site | 3 | 1 | -19545.1915 | N/A | p0=0.92899 p1=0.07100 p2=0.00001 p3=0.00000 w0=0.06501 w1=1.00000 w2=1.00000 | Not Allowed |  |
| modelA | Human | Branch-site | 3 | 2 | -19545.19149 | m1Neutral, modelAnull | p0=0.92900 p1=0.07100 p2=0.00000 p3=0.00000 w0=0.06501 w1=1.00000 w2=1.00000 | No |  |
| modelAnull | Human | Branch-site | 3 | 1 | -19545.19149 | N/A | p0=0.92900 p1=0.07100 p2=0.00000 p3=0.00000 w0=0.06501 w1=1.00000 w2=1.00000 | Not Allowed |  |
| modelA | Marmoset | Branch-site | 3 | 2 | -19545.15506 | m1Neutral, modelAnull | p0=0.91638 p1=0.07001 p2=0.01265 p3=0.00097 w0=0.06492 w1=1.00000 w2=1.00000 | No |  |
| modelAnull | Marmoset | Branch-site | 3 | 1 | -19545.15506 | N/A | p0=0.91638 p1=0.07001 p2=0.01264 p3=0.00097 w0=0.06492 w1=1.00000 w2=1.00000 | Not Allowed |  |
| modelA | Mouse | Branch-site | 3 | 2 | -19545.19149 | m1Neutral, modelAnull | p0=0.92900 p1=0.07100 p2=0.00000 p3=0.00000 w0=0.06501 w1=1.00000 w2=1.00000 | No |  |
| modelAnull | Mouse | Branch-site | 3 | 1 | -19545.19149 | N/A | p0=0.92900 p1=0.07100 p2=0.00000 p3=0.00000 w0=0.06501 w1=1.00000 w2=1.00000 | Not Allowed |  |
| modelA | Muridae | Branch-site | 3 | 10 | -19545.19149 | m1Neutral, modelAnull | p0=0.92900 p1=0.07100 p2=0.00000 p3=0.00000 w0=0.06501 w1=1.00000 w2=11.63093 | No |  |
| modelAnull | Muridae | Branch-site | 3 | 1 | -19545.19149 | N/A | p0=0.92900 p1=0.07100 p2=0.00000 p3=0.00000 w0=0.06501 w1=1.00000 w2=1.00000 | Not Allowed |  |
| modelA | Murinae | Branch-site | 3 | 2 | -19545.19149 | m1Neutral, modelAnull | p0=0.92900 p1=0.07100 p2=0.00000 p3=0.00000 w0=0.06501 w1=1.00000 w2=1.00000 | No |  |
| modelAnull | Murinae | Branch-site | 3 | 1 | -19545.19186 | N/A | p0=0.92900 p1=0.07100 p2=0.00000 p3=0.00000 w0=0.06501 w1=1.00000 w2=1.00000 | Not Allowed |  |
| modelA | Orangutan | Branch-site | 3 | 2 | -19545.14533 | m1Neutral, modelAnull | p0=0.91427 p1=0.06995 p2=0.01465 p3=0.00112 w0=0.06492 w1=1.00000 w2=1.00000 | No |  |
| modelAnull | Orangutan | Branch-site | 3 | 1 | -19545.14533 | N/A | p0=0.91427 p1=0.06996 p2=0.01465 p3=0.00112 w0=0.06492 w1=1.00000 w2=1.00000 | Not Allowed |  |
| modelA | Primates | Branch-site | 3 | 1 | -19545.19149 | m1Neutral, modelAnull | p0=0.92900 p1=0.07100 p2=0.00000 p3=0.00000 w0=0.06501 w1=1.00000 w2=1.00000 | No |  |
| modelAnull | Primates | Branch-site | 3 | 1 | -19545.1915 | N/A | p0=0.92900 p1=0.07100 p2=0.00000 p3=0.00000 w0=0.06501 w1=1.00000 w2=1.00000 | Not Allowed |  |
| modelA | Rabbit | Branch-site | 3 | 2 | -19545.19149 | m1Neutral, modelAnull | p0=0.92900 p1=0.07100 p2=0.00000 p3=0.00000 w0=0.06501 w1=1.00000 w2=1.00000 | No |  |
| modelAnull | Rabbit | Branch-site | 3 | 1 | -19545.19149 | N/A | p0=0.92900 p1=0.07100 p2=0.00000 p3=0.00000 w0=0.06501 w1=1.00000 w2=1.00000 | Not Allowed |  |
| modelA | Rat | Branch-site | 3 | 2 | -19543.65514 | m1Neutral, modelAnull | p0=0.92463 p1=0.07094 p2=0.00412 p3=0.00032 w0=0.06450 w1=1.00000 w2=5.07123 | No |  |
| modelAnull | Rat | Branch-site | 3 | 1 | -19544.11603 | N/A | p0=0.90825 p1=0.06973 p2=0.02045 p3=0.00157 w0=0.06432 w1=1.00000 w2=1.00000 | Not Allowed |  |
| **TSC2 Site Analysis** | | | | | | | | | |
| m0 | Sites | Homogeneous | 1 | 10 | -43198.47115 | N/A | w=0.06235 | No |  |
| m1Neutral | Sites | Site-specific | 1 | 1 | -42677.90304 | N/A | p0=0.90576 p1=0.09424 w0=0.04443 w1=1.00000 | Not Allowed |  |
| m2Selection | Sites | Site-specific | 2 | 1 | -42677.90304 | m1Neutral | p0=0.90576 p1=0.04306 p2=0.05117 w0=0.04443 w1=1.00000 w2=1.00000 | No |  |
| m3Discrtk2 | Sites | Site-specific | 3 | 0 | -42164.50171 | m3Discrtk2 | p0=0.72503 p1=0.27497 w0=0.01695 w1=0.21680 | No |  |
| m3Discrtk3 | Sites | Site-specific | 5 | 1 | -42104.55675 | m3Discrtk3 | p0=0.64116 p1=0.28640 p2=0.07244 w0=0.01138 w1=0.12361 w2=0.45519 | No |  |
| m7 | Sites | Site-specific | 2 | 1 | -42112.48794 | N/A | p=0.34591 q=3.94570 | Not Allowed |  |
| m8 | Sites | Site-specific | 4 | 0 | -42096.22214 | m8a | p=0.38383 p0=0.98468 p1=0.01532 q=5.25554 w=1.00000 | No |  |
| m8a | Sites | Site-specific | 4 | 1 | -42096.22213 | N/A | p=0.38385 p0=0.98468 p1=0.01532 q=5.25583 w=1.00000 | Not Allowed |  |
| **TSC2 Branch-site Analysis** | | | | | | | | | |
| modelA | Chimpanzee | Branch-site | 3 | 2 | -42659.27711 | modelA | p0=0.90352 p1=0.09434 p2=0.00194 p3=0.00020 w0=0.04404 w1=1.00000 w2=190.09480 | Yes | Alignment (6 BEB sites): 1236 1249 1250 1251 1273 1314 |
| modelAnull | Chimpanzee | Branch-site | 3 | 1 | -42670.21941 | N/A | p0=0.82370 p1=0.08591 p2=0.08185 p3=0.00854 w0=0.04397 w1=1.00000 w2=1.00000 | Not Allowed |  |
| modelA | Euarchontoglires | Branch-site | 3 | 1 | -42677.90307 | m1Neutral, modelAnull | p0=0.90574 p1=0.09424 p2=0.00002 p3=0.00000 w0=0.04443 w1=1.00000 w2=1.00000 | No |  |
| modelAnull | Euarchontoglires | Branch-site | 3 | 1 | -42677.90312 | N/A | p0=0.90569 p1=0.09424 p2=0.00006 p3=0.00001 w0=0.04443 w1=1.00000 w2=1.00000 | Not Allowed |  |
| modelA | Glires | Branch-site | 3 | 1 | -42678.02638 | m1Neutral, modelAnull | p0=0.80180 p1=0.08334 p2=0.10405 p3=0.01081 w0=0.04442 w1=1.00000 w2=2.46405 | No |  |
| modelAnull | Glires | Branch-site | 3 | 1 | -42678.02468 | N/A | p0=0.85678 p1=0.08906 p2=0.04907 p3=0.00510 w0=0.04442 w1=1.00000 w2=1.00000 | Not Allowed |  |
| modelA | Gorilla | Branch-site | 3 | 10 | -42569.22884 | modelA | p0=0.89862 p1=0.08796 p2=0.01222 p3=0.00120 w0=0.04339 w1=1.00000 w2=999.00000 | Yes | Alignment (27 BEB sites): 47 48 49 50 52 54 55 56 57 58 59 60 61 62 63 64 66 67 68 69 70 71 73 74 75 313 1333 |
| modelAnull | Gorilla | Branch-site | 3 | 1 | -42647.73379 | N/A | p0=0.79419 p1=0.07831 p2=0.11605 p3=0.01144 w0=0.04323 w1=1.00000 w2=1.00000 | Not Allowed |  |
| modelA | Guinea_Pig | Branch-site | 3 | 2 | -42677.31031 | m1Neutral, modelAnull | p0=0.90542 p1=0.09346 p2=0.00102 p3=0.00011 w0=0.04433 w1=1.00000 w2=474.11904 | No |  |
| modelAnull | Guinea_Pig | Branch-site | 3 | 1 | -42677.9031 | N/A | p0=0.90575 p1=0.09424 p2=0.00000 p3=0.00000 w0=0.04443 w1=1.00000 w2=1.00000 | Not Allowed |  |
| modelA | Homindae | Branch-site | 3 | 2 | -42676.59315 | m1Neutral, modelAnull | p0=0.90369 p1=0.09388 p2=0.00220 p3=0.00023 w0=0.04423 w1=1.00000 w2=6.40277 | No |  |
| modelAnull | Homindae | Branch-site | 3 | 1 | -42677.15292 | N/A | p0=0.89388 p1=0.09280 p2=0.01206 p3=0.00125 w0=0.04420 w1=1.00000 w2=1.00000 | Not Allowed |  |
| modelA | Homininae | Branch-site | 3 | 1 | -42677.80241 | m1Neutral, modelAnull | p0=0.89441 p1=0.09313 p2=0.01128 p3=0.00117 w0=0.04436 w1=1.00000 w2=1.00000 | No |  |
| modelAnull | Homininae | Branch-site | 3 | 1 | -42677.80241 | N/A | p0=0.89441 p1=0.09313 p2=0.01128 p3=0.00117 w0=0.04436 w1=1.00000 w2=1.00000 | Not Allowed |  |
| modelA | Hominini | Branch-site | 3 | 2 | -42677.90307 | m1Neutral, modelAnull | p0=0.90576 p1=0.09424 p2=0.00000 p3=0.00000 w0=0.04443 w1=1.00000 w2=1.00000 | No |  |
| modelAnull | Hominini | Branch-site | 3 | 1 | -42677.90308 | N/A | p0=0.90576 p1=0.09423 p2=0.00001 p3=0.00000 w0=0.04443 w1=1.00000 w2=1.00000 | Not Allowed |  |
| modelA | Human | Branch-site | 3 | 10 | -42677.86383 | m1Neutral, modelAnull | p0=0.89433 p1=0.09304 p2=0.01144 p3=0.00119 w0=0.04441 w1=1.00000 w2=1.00000 | No |  |
| modelAnull | Human | Branch-site | 3 | 1 | -42677.86383 | N/A | p0=0.89426 p1=0.09303 p2=0.01151 p3=0.00120 w0=0.04441 w1=1.00000 w2=1.00000 | Not Allowed |  |
| modelA | Marmoset | Branch-site | 3 | 10 | -42616.04524 | modelA | p0=0.89841 p1=0.09019 p2=0.01035 p3=0.00104 w0=0.04325 w1=1.00000 w2=235.10448 | Yes | Alignment (38 BEB sites): 44 49 51 52 54 57 58 61 63 64 67 68 69 70 71 72 75 88 238 247 249 330 378 413 662 743 797 993 1008 1017 1226 1320 1323 1379 1495 1623 1694 1714 |
| modelAnull | Marmoset | Branch-site | 3 | 1 | -42654.78548 | N/A | p0=0.86337 p1=0.08739 p2=0.04472 p3=0.00453 w0=0.04276 w1=1.00000 w2=1.00000 | Not Allowed |  |
| modelA | Mouse | Branch-site | 3 | 2 | -42677.90306 | m1Neutral, modelAnull | p0=0.90576 p1=0.09424 p2=0.00000 p3=0.00000 w0=0.04443 w1=1.00000 w2=1.00000 | No |  |
| modelAnull | Mouse | Branch-site | 3 | 1 | -42677.90306 | N/A | p0=0.90577 p1=0.09423 p2=0.00000 p3=0.00000 w0=0.04443 w1=1.00000 w2=1.00000 | Not Allowed |  |
| modelA | Muridae | Branch-site | 3 | 2 | -42677.90304 | m1Neutral, modelAnull | p0=0.90576 p1=0.09424 p2=0.00000 p3=0.00000 w0=0.04443 w1=1.00000 w2=1.00000 | No |  |
| modelAnull | Muridae | Branch-site | 3 | 1 | -42677.90316 | N/A | p0=0.90576 p1=0.09424 p2=0.00000 p3=0.00000 w0=0.04443 w1=1.00000 w2=1.00000 | Not Allowed |  |
| modelA | Murinae | Branch-site | 3 | 10 | -42677.90306 | m1Neutral, modelAnull | p0=0.90576 p1=0.09424 p2=0.00000 p3=0.00000 w0=0.04443 w1=1.00000 w2=8.05343 | No |  |
| modelAnull | Murinae | Branch-site | 3 | 1 | -42677.90313 | N/A | p0=0.90576 p1=0.09424 p2=0.00000 p3=0.00000 w0=0.04443 w1=1.00000 w2=1.00000 | Not Allowed |  |
| modelA | Orangutan | Branch-site | 3 | 0 | -42673.92339 | modelA | p0=0.90414 p1=0.09295 p2=0.00263 p3=0.00027 w0=0.04433 w1=1.00000 w2=40.47366 | Yes | Alignment (9 BEB sites): 328 383 535 553 667 1263 1527 1621 1802 |
| modelAnull | Orangutan | Branch-site | 3 | 1 | -42677.0761 | N/A | p0=0.87691 p1=0.09067 p2=0.02938 p3=0.00304 w0=0.04427 w1=1.00000 w2=1.00000 | Not Allowed |  |
| modelA | Primates | Branch-site | 3 | 2 | -42677.71925 | m1Neutral, modelAnull | p0=0.90333 p1=0.09366 p2=0.00273 p3=0.00028 w0=0.04433 w1=1.00000 w2=1.50272 | No |  |
| modelAnull | Primates | Branch-site | 3 | 1 | -42677.90295 | N/A | p0=0.90576 p1=0.09424 p2=0.00000 p3=0.00000 w0=0.04443 w1=1.00000 w2=1.00000 | Not Allowed |  |
| modelA | Rabbit | Branch-site | 3 | 2 | -42677.90307 | m1Neutral, modelAnull | p0=0.90576 p1=0.09424 p2=0.00000 p3=0.00000 w0=0.04443 w1=1.00000 w2=1.00000 | No |  |
| modelAnull | Rabbit | Branch-site | 3 | 1 | -42677.90305 | N/A | p0=0.90577 p1=0.09423 p2=0.00000 p3=0.00000 w0=0.04443 w1=1.00000 w2=1.00000 | Not Allowed |  |
| modelA | Rat | Branch-site | 3 | 0 | -42677.90307 | m1Neutral, modelAnull | p0=0.90576 p1=0.09424 p2=0.00000 p3=0.00000 w0=0.04443 w1=1.00000 w2=1.00000 | No |  |
| modelAnull | Rat | Branch-site | 3 | 1 | -42677.90318 | N/A | p0=0.90576 p1=0.09423 p2=0.00000 p3=0.00000 w0=0.04443 w1=1.00000 w2=1.00000 | Not Allowed |  |
| **MET Site Analysis** | | | | | | | | | |
| m0 | Sites | Homogeneous | 1 | 2 | -36660.849 | N/A | w=0.11716 | No |  |
| m1Neutral | Sites | Site-specific | 1 | 0 | -36065.25728 | N/A | p0=0.84816 p1=0.15184 w0=0.07289 w1=1.00000 | Not Allowed |  |
| m2Selection | Sites | Site-specific | 2 | 2 | -36065.25728 | m1Neutral | p0=0.84815 p1=0.07292 p2=0.07892 w0=0.07289 w1=1.00000 w2=1.00000 | No |  |
| m3Discrtk2 | Sites | Site-specific | 3 | 2 | -35767.9965 | m3Discrtk2 | p0=0.58774 p1=0.41226 w0=0.02506 w1=0.28220 | No |  |
| m3Discrtk3 | Sites | Site-specific | 5 | 0 | -35647.75335 | m3Discrtk3 | p0=0.43956 p1=0.43135 p2=0.12909 w0=0.01016 w1=0.13797 w2=0.57698 | No |  |
| m7 | Sites | Site-specific | 2 | 2 | -35659.84696 | N/A | p=0.41818 q=2.50652 | Not Allowed |  |
| m8 | Sites | Site-specific | 4 | 1 | -35651.2865 | m8a | p=0.46200 p0=0.97676 p1=0.02324 q=3.28130 w=1.00000 | No |  |
| m8a | Sites | Site-specific | 4 | 1 | -35651.2865 | N/A | p=0.46199 p0=0.97676 p1=0.02324 q=3.28129 w=1.00000 | Not Allowed |  |
| **MET Branch-site Analysis** | | | | | | | | | |
| modelA | Chimpanzee | Branch-site | 3 | 2 | -36065.25728 | m1Neutral, modelAnull | p0=0.84816 p1=0.15184 p2=0.00000 p3=0.00000 w0=0.07289 w1=1.00000 w2=6.67524 | No |  |
| modelAnull | Chimpanzee | Branch-site | 3 | 1 | -36065.25728 | N/A | p0=0.84815 p1=0.15185 p2=0.00000 p3=0.00000 w0=0.07289 w1=1.00000 w2=1.00000 | Not Allowed |  |
| modelA | Euarchontoglires | Branch-site | 3 | 2 | -36065.25728 | m1Neutral, modelAnull | p0=0.84815 p1=0.15185 p2=0.00000 p3=0.00000 w0=0.07289 w1=1.00000 w2=1.00000 | No |  |
| modelAnull | Euarchontoglires | Branch-site | 3 | 1 | -36065.25728 | N/A | p0=0.84816 p1=0.15184 p2=0.00000 p3=0.00000 w0=0.07289 w1=1.00000 w2=1.00000 | Not Allowed |  |
| modelA | Glires | Branch-site | 3 | 2 | -36064.49302 | m1Neutral, modelAnull | p0=0.74402 p1=0.13302 p2=0.10431 p3=0.01865 w0=0.07263 w1=1.00000 w2=1.00000 | No |  |
| modelAnull | Glires | Branch-site | 3 | 1 | -36064.49302 | N/A | p0=0.74402 p1=0.13302 p2=0.10431 p3=0.01865 w0=0.07263 w1=1.00000 w2=1.00000 | Not Allowed |  |
| modelA | Gorilla | Branch-site | 3 | 2 | -36065.25728 | m1Neutral, modelAnull | p0=0.84815 p1=0.15185 p2=0.00000 p3=0.00000 w0=0.07289 w1=1.00000 w2=1.00000 | No |  |
| modelAnull | Gorilla | Branch-site | 3 | 1 | -36065.25728 | N/A | p0=0.84815 p1=0.15185 p2=0.00000 p3=0.00000 w0=0.07289 w1=1.00000 w2=1.00000 | Not Allowed |  |
| modelA | Guinea_Pig | Branch-site | 3 | 2 | -36062.92603 | m1Neutral, modelAnull | p0=0.84626 p1=0.14916 p2=0.00390 p3=0.00069 w0=0.07262 w1=1.00000 w2=8.64785 | No |  |
| modelAnull | Guinea_Pig | Branch-site | 3 | 1 | -36064.02479 | N/A | p0=0.82899 p1=0.14667 p2=0.02068 p3=0.00366 w0=0.07226 w1=1.00000 w2=1.00000 | Not Allowed |  |
| modelA | Homindae | Branch-site | 3 | 0 | -36065.25728 | m1Neutral, modelAnull | p0=0.84815 p1=0.15185 p2=0.00000 p3=0.00000 w0=0.07289 w1=1.00000 w2=1.00000 | No |  |
| modelAnull | Homindae | Branch-site | 3 | 1 | -36065.25728 | N/A | p0=0.84815 p1=0.15185 p2=0.00000 p3=0.00000 w0=0.07289 w1=1.00000 w2=1.00000 | Not Allowed |  |
| modelA | Homininae | Branch-site | 3 | 1 | -36065.25728 | m1Neutral, modelAnull | p0=0.84815 p1=0.15185 p2=0.00000 p3=0.00000 w0=0.07289 w1=1.00000 w2=1.00000 | No |  |
| modelAnull | Homininae | Branch-site | 3 | 1 | -36065.25728 | N/A | p0=0.84816 p1=0.15184 p2=0.00000 p3=0.00000 w0=0.07289 w1=1.00000 w2=1.00000 | Not Allowed |  |
| modelA | Hominini | Branch-site | 3 | 1 | -36065.25728 | m1Neutral, modelAnull | p0=0.84816 p1=0.15184 p2=0.00000 p3=0.00000 w0=0.07289 w1=1.00000 w2=1.00000 | No |  |
| modelAnull | Hominini | Branch-site | 3 | 1 | -36065.25728 | N/A | p0=0.84815 p1=0.15185 p2=0.00000 p3=0.00000 w0=0.07289 w1=1.00000 w2=1.00000 | Not Allowed |  |
| modelA | Human | Branch-site | 3 | 10 | -36065.25728 | m1Neutral, modelAnull | p0=0.84816 p1=0.15184 p2=0.00000 p3=0.00000 w0=0.07289 w1=1.00000 w2=8.10114 | No |  |
| modelAnull | Human | Branch-site | 3 | 1 | -36065.25728 | N/A | p0=0.84816 p1=0.15184 p2=0.00000 p3=0.00000 w0=0.07289 w1=1.00000 w2=1.00000 | Not Allowed |  |
| modelA | Marmoset | Branch-site | 3 | 2 | -36064.14826 | m1Neutral, modelAnull | p0=0.83934 p1=0.15000 p2=0.00904 p3=0.00162 w0=0.07242 w1=1.00000 w2=5.50496 | No |  |
| modelAnull | Marmoset | Branch-site | 3 | 1 | -36064.16529 | N/A | p0=0.80845 p1=0.14466 p2=0.03977 p3=0.00712 w0=0.07232 w1=1.00000 w2=1.00000 | Not Allowed |  |
| modelA | Mouse | Branch-site | 3 | 2 | -36065.25728 | m1Neutral, modelAnull | p0=0.84815 p1=0.15185 p2=0.00000 p3=0.00000 w0=0.07289 w1=1.00000 w2=1.00000 | No |  |
| modelAnull | Mouse | Branch-site | 3 | 1 | -36065.25728 | N/A | p0=0.84815 p1=0.15185 p2=0.00000 p3=0.00000 w0=0.07289 w1=1.00000 w2=1.00000 | Not Allowed |  |
| modelA | Muridae | Branch-site | 3 | 2 | -36065.25728 | m1Neutral, modelAnull | p0=0.84816 p1=0.15184 p2=0.00000 p3=0.00000 w0=0.07289 w1=1.00000 w2=1.00000 | No |  |
| modelAnull | Muridae | Branch-site | 3 | 1 | -36065.25728 | N/A | p0=0.84815 p1=0.15185 p2=0.00000 p3=0.00000 w0=0.07289 w1=1.00000 w2=1.00000 | Not Allowed |  |
| modelA | Murinae | Branch-site | 3 | 2 | -36065.00871 | m1Neutral, modelAnull | p0=0.84772 p1=0.15138 p2=0.00077 p3=0.00014 w0=0.07281 w1=1.00000 w2=11.62973 | No |  |
| modelAnull | Murinae | Branch-site | 3 | 1 | -36065.25728 | N/A | p0=0.84815 p1=0.15185 p2=0.00000 p3=0.00000 w0=0.07289 w1=1.00000 w2=1.00000 | Not Allowed |  |
| modelA | Orangutan | Branch-site | 3 | 0 | -36065.25728 | m1Neutral, modelAnull | p0=0.84815 p1=0.15185 p2=0.00000 p3=0.00000 w0=0.07289 w1=1.00000 w2=1.00000 | No |  |
| modelAnull | Orangutan | Branch-site | 3 | 1 | -36065.25728 | N/A | p0=0.84816 p1=0.15184 p2=0.00000 p3=0.00000 w0=0.07289 w1=1.00000 w2=1.00000 | Not Allowed |  |
| modelA | Primates | Branch-site | 3 | 0 | -36063.15365 | m1Neutral, modelAnull | p0=0.83960 p1=0.14941 p2=0.00933 p3=0.00166 w0=0.07239 w1=1.00000 w2=3.69368 | No |  |
| modelAnull | Primates | Branch-site | 3 | 1 | -36063.57253 | N/A | p0=0.81484 p1=0.14488 p2=0.03420 p3=0.00608 w0=0.07230 w1=1.00000 w2=1.00000 | Not Allowed |  |
| modelA | Rabbit | Branch-site | 3 | 0 | -36065.25728 | m1Neutral, modelAnull | p0=0.84815 p1=0.15185 p2=0.00000 p3=0.00000 w0=0.07289 w1=1.00000 w2=1.00000 | No |  |
| modelAnull | Rabbit | Branch-site | 3 | 1 | -36065.25728 | N/A | p0=0.84815 p1=0.15185 p2=0.00000 p3=0.00000 w0=0.07289 w1=1.00000 w2=1.00000 | Not Allowed |  |
| modelA | Rat | Branch-site | 3 | 2 | -36065.25728 | m1Neutral, modelAnull | p0=0.84816 p1=0.15184 p2=0.00000 p3=0.00000 w0=0.07289 w1=1.00000 w2=1.00000 | No |  |
| modelAnull | Rat | Branch-site | 3 | 1 | -36065.25739 | N/A | p0=0.84815 p1=0.15184 p2=0.00000 p3=0.00000 w0=0.07289 w1=1.00000 w2=1.00000 | Not Allowed |  |
| **BMPR1A Site Analysis** | | | | | | | | | |
| m0 | Sites | Homogeneous | 1 | 2 | -9904.747462 | N/A | w=0.03148 | No |  |
| m1Neutral | Sites | Site-specific | 1 | 2 | -9836.488908 | N/A | p0=0.94760 p1=0.05240 w0=0.01795 w1=1.00000 | Not Allowed |  |
| m2Selection | Sites | Site-specific | 2 | 2 | -9836.488908 | m1Neutral | p0=0.94760 p1=0.01230 p2=0.04011 w0=0.01795 w1=1.00000 w2=1.00000 | No |  |
| m3Discrtk2 | Sites | Site-specific | 3 | 2 | -9745.158323 | m3Discrtk2 | p0=0.79960 p1=0.20040 w0=0.00754 w1=0.14124 | No |  |
| m3Discrtk3 | Sites | Site-specific | 5 | 1 | -9727.457773 | m3Discrtk3 | p0=0.52337 p1=0.38717 p2=0.08946 w0=0.00037 w1=0.03038 w2=0.26115 | No |  |
| m7 | Sites | Site-specific | 2 | 2 | -9733.989707 | N/A | p=0.21780 q=5.17753 | Not Allowed |  |
| m8 | Sites | Site-specific | 4 | 2 | -9733.072631 | m7, m8a | p=0.22539 p0=0.99696 p1=0.00304 q=5.75659 w=1.00000 | No |  |
| m8a | Sites | Site-specific | 4 | 1 | -9733.072631 | N/A | p=0.22539 p0=0.99696 p1=0.00304 q=5.75660 w=1.00000 | Not Allowed |  |
| **BMPR1A Branch-site Analysis** | | | | | | | | | |
| modelA | Chimpanzee | Branch-site | 3 | 2 | -9836.488908 | m1Neutral, modelAnull | p0=0.94760 p1=0.05240 p2=0.00000 p3=0.00000 w0=0.01795 w1=1.00000 w2=1.00000 | No |  |
| modelAnull | Chimpanzee | Branch-site | 3 | 1 | -9836.488908 | N/A | p0=0.94760 p1=0.05240 p2=0.00000 p3=0.00000 w0=0.01795 w1=1.00000 w2=1.00000 | Not Allowed |  |
| modelA | Euarchontoglires | Branch-site | 3 | 2 | -9836.488908 | m1Neutral, modelAnull | p0=0.94760 p1=0.05240 p2=0.00000 p3=0.00000 w0=0.01795 w1=1.00000 w2=1.00000 | No |  |
| modelAnull | Euarchontoglires | Branch-site | 3 | 1 | -9836.488908 | N/A | p0=0.94760 p1=0.05240 p2=0.00000 p3=0.00000 w0=0.01795 w1=1.00000 w2=1.00000 | Not Allowed |  |
| modelA | Glires | Branch-site | 3 | 2 | -9836.488908 | m1Neutral, modelAnull | p0=0.94760 p1=0.05240 p2=0.00000 p3=0.00000 w0=0.01795 w1=1.00000 w2=1.00000 | No |  |
| modelAnull | Glires | Branch-site | 3 | 1 | -9836.488908 | N/A | p0=0.94760 p1=0.05240 p2=0.00000 p3=0.00000 w0=0.01795 w1=1.00000 w2=1.00000 | Not Allowed |  |
| modelA | Gorilla | Branch-site | 3 | 2 | -9836.488908 | m1Neutral, modelAnull | p0=0.94760 p1=0.05240 p2=0.00000 p3=0.00000 w0=0.01795 w1=1.00000 w2=1.00000 | No |  |
| modelAnull | Gorilla | Branch-site | 3 | 1 | -9836.488908 | N/A | p0=0.94760 p1=0.05240 p2=0.00000 p3=0.00000 w0=0.01795 w1=1.00000 w2=1.00000 | Not Allowed |  |
| modelA | Guinea_Pig | Branch-site | 3 | 2 | -9836.488908 | m1Neutral, modelAnull | p0=0.94760 p1=0.05240 p2=0.00000 p3=0.00000 w0=0.01795 w1=1.00000 w2=35.71237 | No |  |
| modelAnull | Guinea_Pig | Branch-site | 3 | 1 | -9836.48891 | N/A | p0=0.94760 p1=0.05240 p2=0.00000 p3=0.00000 w0=0.01795 w1=1.00000 w2=1.00000 | Not Allowed |  |
| modelA | Homindae | Branch-site | 3 | 2 | -9836.488908 | m1Neutral, modelAnull | p0=0.94760 p1=0.05240 p2=0.00000 p3=0.00000 w0=0.01795 w1=1.00000 w2=1.00000 | No |  |
| modelAnull | Homindae | Branch-site | 3 | 1 | -9836.488908 | N/A | p0=0.94760 p1=0.05240 p2=0.00000 p3=0.00000 w0=0.01795 w1=1.00000 w2=1.00000 | Not Allowed |  |
| modelA | Homininae | Branch-site | 3 | 2 | -9836.488908 | m1Neutral, modelAnull | p0=0.94760 p1=0.05240 p2=0.00000 p3=0.00000 w0=0.01795 w1=1.00000 w2=1.00000 | No |  |
| modelAnull | Homininae | Branch-site | 3 | 1 | -9836.488908 | N/A | p0=0.94760 p1=0.05240 p2=0.00000 p3=0.00000 w0=0.01795 w1=1.00000 w2=1.00000 | Not Allowed |  |
| modelA | Hominini | Branch-site | 3 | 2 | -9836.488908 | m1Neutral, modelAnull | p0=0.94760 p1=0.05240 p2=0.00000 p3=0.00000 w0=0.01795 w1=1.00000 w2=1.00000 | No |  |
| modelAnull | Hominini | Branch-site | 3 | 1 | -9836.488908 | N/A | p0=0.94760 p1=0.05240 p2=0.00000 p3=0.00000 w0=0.01795 w1=1.00000 w2=1.00000 | Not Allowed |  |
| modelA | Human | Branch-site | 3 | 10 | -9835.571676 | m1Neutral, modelAnull | p0=0.85513 p1=0.04759 p2=0.09216 p3=0.00513 w0=0.01769 w1=1.00000 w2=1.00000 | No |  |
| modelAnull | Human | Branch-site | 3 | 1 | -9835.571676 | N/A | p0=0.85512 p1=0.04759 p2=0.09216 p3=0.00513 w0=0.01769 w1=1.00000 w2=1.00000 | Not Allowed |  |
| modelA | Marmoset | Branch-site | 3 | 2 | -9836.488908 | m1Neutral, modelAnull | p0=0.94760 p1=0.05240 p2=0.00000 p3=0.00000 w0=0.01795 w1=1.00000 w2=1.00000 | No |  |
| modelAnull | Marmoset | Branch-site | 3 | 1 | -9836.488908 | N/A | p0=0.94760 p1=0.05240 p2=0.00000 p3=0.00000 w0=0.01795 w1=1.00000 w2=1.00000 | Not Allowed |  |
| modelA | Mouse | Branch-site | 3 | 2 | -9836.488908 | m1Neutral, modelAnull | p0=0.94760 p1=0.05240 p2=0.00000 p3=0.00000 w0=0.01795 w1=1.00000 w2=1.00000 | No |  |
| modelAnull | Mouse | Branch-site | 3 | 1 | -9836.488908 | N/A | p0=0.94760 p1=0.05240 p2=0.00000 p3=0.00000 w0=0.01795 w1=1.00000 w2=1.00000 | Not Allowed |  |
| modelA | Muridae | Branch-site | 3 | 0 | -9836.488908 | m1Neutral, modelAnull | p0=0.94754 p1=0.05240 p2=0.00006 p3=0.00000 w0=0.01795 w1=1.00000 w2=1.00000 | No |  |
| modelAnull | Muridae | Branch-site | 3 | 1 | -9836.488908 | N/A | p0=0.94759 p1=0.05240 p2=0.00000 p3=0.00000 w0=0.01795 w1=1.00000 w2=1.00000 | Not Allowed |  |
| modelA | Murinae | Branch-site | 3 | 2 | -9836.487413 | m1Neutral, modelAnull | p0=0.94699 p1=0.05238 p2=0.00059 p3=0.00003 w0=0.01792 w1=1.00000 w2=1.12752 | No |  |
| modelAnull | Murinae | Branch-site | 3 | 1 | -9836.487423 | N/A | p0=0.94693 p1=0.05238 p2=0.00066 p3=0.00004 w0=0.01792 w1=1.00000 w2=1.00000 | Not Allowed |  |
| modelA | Orangutan | Branch-site | 3 | 2 | -9836.488908 | m1Neutral, modelAnull | p0=0.94760 p1=0.05240 p2=0.00000 p3=0.00000 w0=0.01795 w1=1.00000 w2=1.00000 | No |  |
| modelAnull | Orangutan | Branch-site | 3 | 1 | -9836.488908 | N/A | p0=0.94760 p1=0.05240 p2=0.00000 p3=0.00000 w0=0.01795 w1=1.00000 w2=1.00000 | Not Allowed |  |
| modelA | Primates | Branch-site | 3 | 2 | -9836.488908 | m1Neutral, modelAnull | p0=0.94760 p1=0.05240 p2=0.00000 p3=0.00000 w0=0.01795 w1=1.00000 w2=1.00000 | No |  |
| modelAnull | Primates | Branch-site | 3 | 1 | -9836.488908 | N/A | p0=0.94760 p1=0.05240 p2=0.00000 p3=0.00000 w0=0.01795 w1=1.00000 w2=1.00000 | Not Allowed |  |
| modelA | Rabbit | Branch-site | 3 | 10 | -9836.488908 | m1Neutral, modelAnull | p0=0.94760 p1=0.05240 p2=0.00000 p3=0.00000 w0=0.01795 w1=1.00000 w2=29.16723 | No |  |
| modelAnull | Rabbit | Branch-site | 3 | 1 | -9836.488908 | N/A | p0=0.94760 p1=0.05240 p2=0.00000 p3=0.00000 w0=0.01795 w1=1.00000 w2=1.00000 | Not Allowed |  |
| modelA | Rat | Branch-site | 3 | 2 | -9836.354657 | m1Neutral, modelAnull | p0=0.93813 p1=0.05167 p2=0.00967 p3=0.00053 w0=0.01778 w1=1.00000 w2=1.00000 | No |  |
| modelAnull | Rat | Branch-site | 3 | 1 | -9836.488904 | N/A | p0=0.94760 p1=0.05240 p2=0.00000 p3=0.00000 w0=0.01795 w1=1.00000 w2=1.00000 | Not Allowed |  |
| **MSH6 Site Analysis** | | | | | | | | | |
| m0 | Sites | Homogeneous | 1 | 2 | -34979.24911 | N/A | w=0.12474 | No |  |
| m1Neutral | Sites | Site-specific | 1 | 2 | -34194.37989 | N/A | p0=0.80184 p1=0.19816 w0=0.07096 w1=1.00000 | Not Allowed |  |
| m2Selection | Sites | Site-specific | 2 | 2 | -34194.37989 | m1Neutral | p0=0.80184 p1=0.12858 p2=0.06958 w0=0.07096 w1=1.00000 w2=1.00000 | No |  |
| m3Discrtk2 | Sites | Site-specific | 3 | 2 | -33912.77428 | m3Discrtk2 | p0=0.63468 p1=0.36532 w0=0.02806 w1=0.35136 | No |  |
| m3Discrtk3 | Sites | Site-specific | 5 | 0 | -33799.62016 | m3Discrtk3 | p0=0.47826 p1=0.37974 p2=0.14200 w0=0.01000 w1=0.16426 w2=0.63539 | No |  |
| m7 | Sites | Site-specific | 2 | 2 | -33798.06336 | N/A | p=0.33546 q=1.74250 | Not Allowed |  |
| m8 | Sites | Site-specific | 4 | 2 | -33789.74458 | m8a | p=0.37918 p0=0.96857 p1=0.03143 q=2.44366 w=1.13741 | No |  |
| m8a | Sites | Site-specific | 4 | 1 | -33790.08082 | N/A | p=0.38730 p0=0.96004 p1=0.03996 q=2.61545 w=1.00000 | Not Allowed |  |
| **MSH6 Branch-site Analysis** | | | | | | | | | |
| modelA | Chimpanzee | Branch-site | 3 | 2 | -34194.37989 | m1Neutral, modelAnull | p0=0.80184 p1=0.19816 p2=0.00000 p3=0.00000 w0=0.07096 w1=1.00000 w2=1.00000 | No |  |
| modelAnull | Chimpanzee | Branch-site | 3 | 1 | -34194.37989 | N/A | p0=0.80184 p1=0.19816 p2=0.00000 p3=0.00000 w0=0.07096 w1=1.00000 w2=1.00000 | Not Allowed |  |
| modelA | Euarchontoglires | Branch-site | 3 | 2 | -34192.67414 | m1Neutral, modelAnull | p0=0.79961 p1=0.19784 p2=0.00204 p3=0.00051 w0=0.07075 w1=1.00000 w2=39.29807 | No |  |
| modelAnull | Euarchontoglires | Branch-site | 3 | 1 | -34193.93104 | N/A | p0=0.75669 p1=0.18718 p2=0.04500 p3=0.01113 w0=0.07076 w1=1.00000 w2=1.00000 | Not Allowed |  |
| modelA | Glires | Branch-site | 3 | 2 | -34194.15634 | m1Neutral, modelAnull | p0=0.75497 p1=0.18627 p2=0.04713 p3=0.01163 w0=0.07087 w1=1.00000 w2=1.00000 | No |  |
| modelAnull | Glires | Branch-site | 3 | 1 | -34194.15634 | N/A | p0=0.75497 p1=0.18626 p2=0.04714 p3=0.01163 w0=0.07087 w1=1.00000 w2=1.00000 | Not Allowed |  |
| modelA | Gorilla | Branch-site | 3 | 2 | -34009.90221 | modelA | p0=0.78382 p1=0.18418 p2=0.02591 p3=0.00609 w0=0.06974 w1=1.00000 w2=999.00000 | Yes | Alignment (46 BEB sites): 157 158 159 160 162 163 165 166 167 170 171 173 174 175 177 178 179 180 181 182 183 184 185 186 187 188 189 191 192 193 194 195 196 197 198 199 200 201 202 203 208 209 210 212 213 1247 |
| modelAnull | Gorilla | Branch-site | 3 | 1 | -34160.71699 | N/A | p0=0.64704 p1=0.15321 p2=0.16151 p3=0.03824 w0=0.06930 w1=1.00000 w2=1.00000 | Not Allowed |  |
| modelA | Guinea_Pig | Branch-site | 3 | 2 | -34194.37989 | m1Neutral, modelAnull | p0=0.80184 p1=0.19816 p2=0.00000 p3=0.00000 w0=0.07096 w1=1.00000 w2=1.00000 | No |  |
| modelAnull | Guinea_Pig | Branch-site | 3 | 1 | -34194.37989 | N/A | p0=0.80184 p1=0.19816 p2=0.00000 p3=0.00000 w0=0.07096 w1=1.00000 w2=1.00000 | Not Allowed |  |
| modelA | Homindae | Branch-site | 3 | 2 | -34194.37989 | m1Neutral, modelAnull | p0=0.80184 p1=0.19816 p2=0.00000 p3=0.00000 w0=0.07096 w1=1.00000 w2=1.00000 | No |  |
| modelAnull | Homindae | Branch-site | 3 | 1 | -34194.37989 | N/A | p0=0.80185 p1=0.19815 p2=0.00000 p3=0.00000 w0=0.07096 w1=1.00000 w2=1.00000 | Not Allowed |  |
| modelA | Homininae | Branch-site | 3 | 0 | -34194.37989 | m1Neutral, modelAnull | p0=0.80184 p1=0.19816 p2=0.00000 p3=0.00000 w0=0.07096 w1=1.00000 w2=1.00000 | No |  |
| modelAnull | Homininae | Branch-site | 3 | 1 | -34194.37989 | N/A | p0=0.80184 p1=0.19816 p2=0.00000 p3=0.00000 w0=0.07096 w1=1.00000 w2=1.00000 | Not Allowed |  |
| modelA | Hominini | Branch-site | 3 | 10 | -34194.37989 | m1Neutral, modelAnull | p0=0.80184 p1=0.19816 p2=0.00000 p3=0.00000 w0=0.07096 w1=1.00000 w2=8.03475 | No |  |
| modelAnull | Hominini | Branch-site | 3 | 1 | -34194.3799 | N/A | p0=0.80184 p1=0.19816 p2=0.00000 p3=0.00000 w0=0.07096 w1=1.00000 w2=1.00000 | Not Allowed |  |
| modelA | Human | Branch-site | 3 | 2 | -34194.0311 | m1Neutral, modelAnull | p0=0.73832 p1=0.18269 p2=0.06333 p3=0.01567 w0=0.07084 w1=1.00000 w2=1.00000 | No |  |
| modelAnull | Human | Branch-site | 3 | 1 | -34194.0311 | N/A | p0=0.73832 p1=0.18269 p2=0.06332 p3=0.01567 w0=0.07084 w1=1.00000 w2=1.00000 | Not Allowed |  |
| modelA | Marmoset | Branch-site | 3 | 2 | -34084.0252 | modelA | p0=0.77824 p1=0.19182 p2=0.02402 p3=0.00592 w0=0.06843 w1=1.00000 w2=260.51094 | Yes | Alignment (45 BEB sites): 46 157 159 160 161 163 165 166 170 171 173 174 175 177 178 179 181 182 185 186 187 190 193 194 195 196 197 201 202 203 204 205 206 207 208 209 212 288 540 667 758 807 844 867 1106 |
| modelAnull | Marmoset | Branch-site | 3 | 1 | -34160.96357 | N/A | p0=0.69662 p1=0.17090 p2=0.10638 p3=0.02610 w0=0.06777 w1=1.00000 w2=1.00000 | Not Allowed |  |
| modelA | Mouse | Branch-site | 3 | 2 | -34192.77321 | m1Neutral, modelAnull | p0=0.80065 p1=0.19780 p2=0.00124 p3=0.00031 w0=0.07074 w1=1.00000 w2=16.11064 | No |  |
| modelAnull | Mouse | Branch-site | 3 | 1 | -34193.05973 | N/A | p0=0.77925 p1=0.19265 p2=0.02253 p3=0.00557 w0=0.07035 w1=1.00000 w2=1.00000 | Not Allowed |  |
| modelA | Muridae | Branch-site | 3 | 2 | -34193.72656 | m1Neutral, modelAnull | p0=0.74620 p1=0.18409 p2=0.05591 p3=0.01379 w0=0.07064 w1=1.00000 w2=1.00000 | No |  |
| modelAnull | Muridae | Branch-site | 3 | 1 | -34193.72656 | N/A | p0=0.74620 p1=0.18409 p2=0.05591 p3=0.01379 w0=0.07064 w1=1.00000 w2=1.00000 | Not Allowed |  |
| modelA | Murinae | Branch-site | 3 | 2 | -34190.13821 | modelA | p0=0.79911 p1=0.19671 p2=0.00335 p3=0.00082 w0=0.07057 w1=1.00000 w2=126.22513 | Yes | Alignment (3 BEB sites): 379 766 1268 |
| modelAnull | Murinae | Branch-site | 3 | 1 | -34193.10977 | N/A | p0=0.78729 p1=0.19407 p2=0.01496 p3=0.00369 w0=0.07035 w1=1.00000 w2=1.00000 | Not Allowed |  |
| modelA | Orangutan | Branch-site | 3 | 2 | -34193.18925 | m1Neutral, modelAnull | p0=0.70981 p1=0.17564 p2=0.09183 p3=0.02272 w0=0.07067 w1=1.00000 w2=1.00000 | No |  |
| modelAnull | Orangutan | Branch-site | 3 | 1 | -34193.18925 | N/A | p0=0.70981 p1=0.17564 p2=0.09183 p3=0.02272 w0=0.07067 w1=1.00000 w2=1.00000 | Not Allowed |  |
| modelA | Primates | Branch-site | 3 | 10 | -34194.04524 | m1Neutral, modelAnull | p0=0.80210 p1=0.19711 p2=0.00063 p3=0.00015 w0=0.07100 w1=1.00000 w2=32.53392 | No |  |
| modelAnull | Primates | Branch-site | 3 | 1 | -34194.37989 | N/A | p0=0.80184 p1=0.19816 p2=0.00000 p3=0.00000 w0=0.07096 w1=1.00000 w2=1.00000 | Not Allowed |  |
| modelA | Rabbit | Branch-site | 3 | 2 | -34191.79998 | m1Neutral, modelAnull | p0=0.79994 p1=0.19643 p2=0.00291 p3=0.00072 w0=0.07069 w1=1.00000 w2=10.08322 | No |  |
| modelAnull | Rabbit | Branch-site | 3 | 1 | -34193.47205 | N/A | p0=0.78907 p1=0.19433 p2=0.01332 p3=0.00328 w0=0.07054 w1=1.00000 w2=1.00000 | Not Allowed |  |
| modelA | Rat | Branch-site | 3 | 2 | -34194.37989 | m1Neutral, modelAnull | p0=0.80184 p1=0.19816 p2=0.00000 p3=0.00000 w0=0.07096 w1=1.00000 w2=1.00000 | No |  |
| modelAnull | Rat | Branch-site | 3 | 1 | -34194.37989 | N/A | p0=0.80184 p1=0.19816 p2=0.00000 p3=0.00000 w0=0.07096 w1=1.00000 w2=1.00000 | Not Allowed |  |
| **SDHB Site Analysis** | | | | | | | | | |
| m0 | Sites | Homogeneous | 1 | 2 | -5235.819523 | N/A | w=0.05498 | No |  |
| m1Neutral | Sites | Site-specific | 1 | 2 | -5109.594985 | N/A | p0=0.89158 p1=0.10842 w0=0.02630 w1=1.00000 | Not Allowed |  |
| m2Selection | Sites | Site-specific | 2 | 2 | -5109.594985 | m1Neutral | p0=0.89158 p1=0.06512 p2=0.04330 w0=0.02630 w1=1.00000 w2=1.00000 | No |  |
| m3Discrtk2 | Sites | Site-specific | 3 | 2 | -5053.112286 | m3Discrtk2 | p0=0.72009 p1=0.27991 w0=0.00199 w1=0.22970 | No |  |
| m3Discrtk3 | Sites | Site-specific | 5 | 0 | -5035.829731 | m3Discrtk3 | p0=0.66828 p1=0.23923 p2=0.09249 w0=0.00000 w1=0.11424 w2=0.51871 | No |  |
| m7 | Sites | Site-specific | 2 | 2 | -5041.279372 | N/A | p=0.12411 q=1.43252 | Not Allowed |  |
| m8 | Sites | Site-specific | 4 | 0 | -5041.280071 | m7, m8a | p=0.12411 p0=0.99999 p1=0.00001 q=1.43262 w=1.00000 | No |  |
| m8a | Sites | Site-specific | 4 | 1 | -5041.280071 | N/A | p=0.12411 p0=0.99999 p1=0.00001 q=1.43262 w=1.00000 | Not Allowed |  |
| **SHDB Branch-site Analysis** | | | | | | | | | |
| modelA | Chimpanzee | Branch-site | 3 | 2 | -5109.594991 | m1Neutral, modelAnull | p0=0.76110 p1=0.09256 p2=0.13047 p3=0.01587 w0=0.02630 w1=1.00000 w2=3.11095 | No |  |
| modelAnull | Chimpanzee | Branch-site | 3 | 1 | -5109.594991 | N/A | p0=0.78119 p1=0.09500 p2=0.11039 p3=0.01342 w0=0.02630 w1=1.00000 w2=1.00000 | Not Allowed |  |
| modelA | Euarchontoglires | Branch-site | 3 | 2 | -5109.594985 | m1Neutral, modelAnull | p0=0.89158 p1=0.10842 p2=0.00000 p3=0.00000 w0=0.02630 w1=1.00000 w2=1.00000 | No |  |
| modelAnull | Euarchontoglires | Branch-site | 3 | 1 | -5109.594985 | N/A | p0=0.89158 p1=0.10842 p2=0.00000 p3=0.00000 w0=0.02630 w1=1.00000 w2=1.00000 | Not Allowed |  |
| modelA | Gorilla | Branch-site | 3 | 2 | -5109.594985 | m1Neutral, modelAnull | p0=0.89158 p1=0.10842 p2=0.00000 p3=0.00000 w0=0.02630 w1=1.00000 w2=1.00000 | No |  |
| modelAnull | Gorilla | Branch-site | 3 | 1 | -5109.594985 | N/A | p0=0.89158 p1=0.10842 p2=0.00000 p3=0.00000 w0=0.02630 w1=1.00000 w2=1.00000 | Not Allowed |  |
| modelA | Guinea_Pig | Branch-site | 3 | 2 | -5109.594985 | m1Neutral, modelAnull | p0=0.89158 p1=0.10842 p2=0.00000 p3=0.00000 w0=0.02630 w1=1.00000 w2=1.00000 | No |  |
| modelAnull | Guinea_Pig | Branch-site | 3 | 1 | -5109.594985 | N/A | p0=0.89158 p1=0.10842 p2=0.00000 p3=0.00000 w0=0.02630 w1=1.00000 w2=1.00000 | Not Allowed |  |
| modelA | Homindae | Branch-site | 3 | 2 | -5109.18816 | m1Neutral, modelAnull | p0=0.85727 p1=0.10322 p2=0.03527 p3=0.00425 w0=0.02611 w1=1.00000 w2=1.42306 | No |  |
| modelAnull | Homindae | Branch-site | 3 | 1 | -5109.195665 | N/A | p0=0.84540 p1=0.10182 p2=0.04711 p3=0.00567 w0=0.02611 w1=1.00000 w2=1.00000 | Not Allowed |  |
| modelA | Homininae | Branch-site | 3 | 2 | -5109.594985 | m1Neutral, modelAnull | p0=0.89158 p1=0.10842 p2=0.00000 p3=0.00000 w0=0.02630 w1=1.00000 w2=1.00000 | No |  |
| modelAnull | Homininae | Branch-site | 3 | 1 | -5109.594985 | N/A | p0=0.89158 p1=0.10842 p2=0.00000 p3=0.00000 w0=0.02630 w1=1.00000 w2=1.00000 | Not Allowed |  |
| modelA | Hominini | Branch-site | 3 | 0 | -5109.594991 | m1Neutral, modelAnull | p0=0.78153 p1=0.09504 p2=0.11005 p3=0.01338 w0=0.02630 w1=1.00000 w2=1.41849 | No |  |
| modelAnull | Hominini | Branch-site | 3 | 1 | -5109.594992 | N/A | p0=0.76746 p1=0.09333 p2=0.12411 p3=0.01509 w0=0.02630 w1=1.00000 w2=1.00000 | Not Allowed |  |
| modelA | Human | Branch-site | 3 | 2 | -5109.594985 | m1Neutral, modelAnull | p0=0.89158 p1=0.10842 p2=0.00000 p3=0.00000 w0=0.02630 w1=1.00000 w2=1.00000 | No |  |
| modelAnull | Human | Branch-site | 3 | 1 | -5109.594986 | N/A | p0=0.89158 p1=0.10842 p2=0.00000 p3=0.00000 w0=0.02630 w1=1.00000 w2=1.00000 | Not Allowed |  |
| modelA | Marmoset | Branch-site | 3 | 2 | -5109.594985 | m1Neutral, modelAnull | p0=0.89158 p1=0.10842 p2=0.00000 p3=0.00000 w0=0.02630 w1=1.00000 w2=1.00000 | No |  |
| modelAnull | Marmoset | Branch-site | 3 | 1 | -5109.594985 | N/A | p0=0.89158 p1=0.10842 p2=0.00000 p3=0.00000 w0=0.02630 w1=1.00000 w2=1.00000 | Not Allowed |  |
| modelA | Mouse | Branch-site | 3 | 2 | -5109.490916 | m1Neutral, modelAnull | p0=0.89012 p1=0.10630 p2=0.00320 p3=0.00038 w0=0.02647 w1=1.00000 w2=9.60778 | No |  |
| modelAnull | Mouse | Branch-site | 3 | 1 | -5109.594985 | N/A | p0=0.89158 p1=0.10842 p2=0.00000 p3=0.00000 w0=0.02630 w1=1.00000 w2=1.00000 | Not Allowed |  |
| modelA | Muridae | Branch-site | 3 | 2 | -5107.84368 | m1Neutral, modelAnull | p0=0.89040 p1=0.10552 p2=0.00365 p3=0.00043 w0=0.02634 w1=1.00000 w2=43.31805 | No |  |
| modelAnull | Muridae | Branch-site | 3 | 1 | -5109.497409 | N/A | p0=0.87966 p1=0.10576 p2=0.01302 p3=0.00156 w0=0.02633 w1=1.00000 w2=1.00000 | Not Allowed |  |
| modelA | Murinae | Branch-site | 3 | 2 | -5109.526608 | m1Neutral, modelAnull | p0=0.89018 p1=0.10832 p2=0.00133 p3=0.00016 w0=0.02618 w1=1.00000 w2=8.06668 | No |  |
| modelAnull | Murinae | Branch-site | 3 | 1 | -5109.594985 | N/A | p0=0.89158 p1=0.10842 p2=0.00000 p3=0.00000 w0=0.02630 w1=1.00000 w2=1.00000 | Not Allowed |  |
| modelA | Orangutan | Branch-site | 3 | 2 | -5109.272792 | m1Neutral, modelAnull | p0=0.84103 p1=0.10196 p2=0.05085 p3=0.00616 w0=0.02613 w1=1.00000 w2=1.00000 | No |  |
| modelAnull | Orangutan | Branch-site | 3 | 1 | -5109.272792 | N/A | p0=0.84103 p1=0.10196 p2=0.05084 p3=0.00616 w0=0.02613 w1=1.00000 w2=1.00000 | Not Allowed |  |
| modelA | Primates | Branch-site | 3 | 2 | -5109.594985 | m1Neutral, modelAnull | p0=0.89158 p1=0.10842 p2=0.00000 p3=0.00000 w0=0.02630 w1=1.00000 w2=1.00000 | No |  |
| modelAnull | Primates | Branch-site | 3 | 1 | -5109.594985 | N/A | p0=0.89158 p1=0.10842 p2=0.00000 p3=0.00000 w0=0.02630 w1=1.00000 w2=1.00000 | Not Allowed |  |
| modelA | Rat | Branch-site | 3 | 10 | -5108.66736 | m1Neutral, modelAnull | p0=0.88794 p1=0.10839 p2=0.00327 p3=0.00040 w0=0.02593 w1=1.00000 w2=243.27424 | No |  |
| modelAnull | Rat | Branch-site | 3 | 1 | -5108.882964 | N/A | p0=0.86978 p1=0.10490 p2=0.02259 p3=0.00273 w0=0.02596 w1=1.00000 w2=1.00000 | Not Allowed |  |
| **STK11 Site Analysis** | | | | | | | | | |
| m0 | Sites | Homogeneous | 1 | 2 | -8682.828081 | N/A | w=0.04297 | No |  |
| m1Neutral | Sites | Site-specific | 1 | 2 | -8608.294605 | N/A | p0=0.93996 p1=0.06004 w0=0.03370 w1=1.00000 | Not Allowed |  |
| m2Selection | Sites | Site-specific | 2 | 2 | -8608.294605 | m1Neutral | p0=0.93996 p1=0.06004 p2=0.00000 w0=0.03370 w1=1.00000 w2=1.86298 | No |  |
| m3Discrtk2 | Sites | Site-specific | 3 | 2 | -8494.348233 | m3Discrtk2 | p0=0.71423 p1=0.28577 w0=0.00618 w1=0.15995 | No |  |
| m3Discrtk3 | Sites | Site-specific | 5 | 0 | -8478.832407 | m3Discrtk3 | p0=0.56707 p1=0.33108 p2=0.10185 w0=0.00041 w1=0.06800 w2=0.30418 | No |  |
| m7 | Sites | Site-specific | 2 | 2 | -8481.06595 | N/A | p=0.22162 q=3.55407 | Not Allowed |  |
| m8 | Sites | Site-specific | 4 | 2 | -8481.068571 | m7, m8a | p=0.22163 p0=0.99999 p1=0.00001 q=3.55429 w=1.00000 | No |  |
| m8a | Sites | Site-specific | 4 | 1 | -8481.068571 | N/A | p=0.22163 p0=0.99999 p1=0.00001 q=3.55427 w=1.00000 | Not Allowed |  |
| **STK11 Branch-site Analysis** | | | | | | | | | |
| modelA | Chimpanzee | Branch-site | 3 | 2 | -8608.150595 | m1Neutral, modelAnull | p0=0.91282 p1=0.05842 p2=0.02703 p3=0.00173 w0=0.03362 w1=1.00000 w2=1.14209 | No |  |
| modelAnull | Chimpanzee | Branch-site | 3 | 1 | -8608.150746 | N/A | p0=0.90911 p1=0.05819 p2=0.03074 p3=0.00197 w0=0.03362 w1=1.00000 w2=1.00000 | Not Allowed |  |
| modelA | Euarchontoglires | Branch-site | 3 | 2 | -8602.921472 | modelA | p0=0.93299 p1=0.05633 p2=0.01007 p3=0.00061 w0=0.03346 w1=1.00000 w2=197.90897 | Yes | Alignment (3 BEB sites): 206 301 415 |
| modelAnull | Euarchontoglires | Branch-site | 3 | 1 | -8605.848095 | N/A | p0=0.82004 p1=0.04999 p2=0.12251 p3=0.00747 w0=0.03349 w1=1.00000 w2=1.00000 | Not Allowed |  |
| modelA | Gorilla | Branch-site | 3 | 2 | -8608.294605 | m1Neutral, modelAnull | p0=0.93996 p1=0.06004 p2=0.00000 p3=0.00000 w0=0.03370 w1=1.00000 w2=1.00000 | No |  |
| modelAnull | Gorilla | Branch-site | 3 | 1 | -8608.294605 | N/A | p0=0.93996 p1=0.06004 p2=0.00000 p3=0.00000 w0=0.03370 w1=1.00000 w2=1.00000 | Not Allowed |  |
| modelA | Guinea_Pig | Branch-site | 3 | 2 | -8605.860589 | m1Neutral, modelAnull | p0=0.90514 p1=0.05850 p2=0.03415 p3=0.00221 w0=0.03260 w1=1.00000 w2=1.00000 | No |  |
| modelAnull | Guinea_Pig | Branch-site | 3 | 1 | -8605.860589 | N/A | p0=0.90514 p1=0.05850 p2=0.03415 p3=0.00221 w0=0.03260 w1=1.00000 w2=1.00000 | Not Allowed |  |
| modelA | Homindae | Branch-site | 3 | 2 | -8601.056009 | modelA | p0=0.93574 p1=0.05920 p2=0.00476 p3=0.00030 w0=0.03323 w1=1.00000 w2=44.31709 | Yes | Alignment (3 BEB sites): 271 347 381 |
| modelAnull | Homindae | Branch-site | 3 | 1 | -8605.281628 | N/A | p0=0.92566 p1=0.05852 p2=0.01489 p3=0.00094 w0=0.03310 w1=1.00000 w2=1.00000 | Not Allowed |  |
| modelA | Homininae | Branch-site | 3 | 2 | -8607.908274 | m1Neutral, modelAnull | p0=0.86666 p1=0.05509 p2=0.07357 p3=0.00468 w0=0.03360 w1=1.00000 w2=1.00000 | No |  |
| modelAnull | Homininae | Branch-site | 3 | 1 | -8607.908274 | N/A | p0=0.86666 p1=0.05509 p2=0.07357 p3=0.00468 w0=0.03360 w1=1.00000 w2=1.00000 | Not Allowed |  |
| modelA | Hominini | Branch-site | 3 | 2 | -8608.294605 | m1Neutral, modelAnull | p0=0.93996 p1=0.06004 p2=0.00000 p3=0.00000 w0=0.03370 w1=1.00000 w2=1.00000 | No |  |
| modelAnull | Hominini | Branch-site | 3 | 1 | -8608.294605 | N/A | p0=0.93996 p1=0.06004 p2=0.00000 p3=0.00000 w0=0.03370 w1=1.00000 w2=1.00000 | Not Allowed |  |
| modelA | Human | Branch-site | 3 | 2 | -8608.294605 | m1Neutral, modelAnull | p0=0.93996 p1=0.06004 p2=0.00000 p3=0.00000 w0=0.03370 w1=1.00000 w2=1.00000 | No |  |
| modelAnull | Human | Branch-site | 3 | 1 | -8608.294605 | N/A | p0=0.93996 p1=0.06004 p2=0.00000 p3=0.00000 w0=0.03370 w1=1.00000 w2=1.00000 | Not Allowed |  |
| modelA | Mouse | Branch-site | 3 | 2 | -8608.294605 | m1Neutral, modelAnull | p0=0.93996 p1=0.06004 p2=0.00000 p3=0.00000 w0=0.03370 w1=1.00000 w2=1.00000 | No |  |
| modelAnull | Mouse | Branch-site | 3 | 1 | -8608.294605 | N/A | p0=0.93996 p1=0.06004 p2=0.00000 p3=0.00000 w0=0.03370 w1=1.00000 w2=1.00000 | Not Allowed |  |
| modelA | Muridae | Branch-site | 3 | 2 | -8605.81358 | m1Neutral | p0=0.93439 p1=0.05899 p2=0.00623 p3=0.00039 w0=0.03337 w1=1.00000 w2=998.99956 | No |  |
| modelAnull | Muridae | Branch-site | 3 | 1 | -8608.079898 | N/A | p0=0.92821 p1=0.05889 p2=0.01214 p3=0.00077 w0=0.03344 w1=1.00000 w2=1.00000 | Not Allowed |  |
| modelA | Murinae | Branch-site | 3 | 0 | -8606.389736 | m1Neutral, modelAnull | p0=0.93661 p1=0.05951 p2=0.00365 p3=0.00023 w0=0.03345 w1=1.00000 w2=345.05372 | No |  |
| modelAnull | Murinae | Branch-site | 3 | 1 | -8607.975008 | N/A | p0=0.93465 p1=0.05931 p2=0.00568 p3=0.00036 w0=0.03345 w1=1.00000 w2=1.00000 | Not Allowed |  |
| modelA | Orangutan | Branch-site | 3 | 2 | -8608.294605 | m1Neutral, modelAnull | p0=0.93996 p1=0.06004 p2=0.00000 p3=0.00000 w0=0.03370 w1=1.00000 w2=1.00000 | No |  |
| modelAnull | Orangutan | Branch-site | 3 | 1 | -8608.294605 | N/A | p0=0.93996 p1=0.06004 p2=0.00000 p3=0.00000 w0=0.03370 w1=1.00000 w2=1.00000 | Not Allowed |  |
| modelA | Rat | Branch-site | 3 | 2 | -8608.294605 | m1Neutral, modelAnull | p0=0.93996 p1=0.06004 p2=0.00000 p3=0.00000 w0=0.03370 w1=1.00000 w2=1.00000 | No |  |
| modelAnull | Rat | Branch-site | 3 | 1 | -8608.294605 | N/A | p0=0.93996 p1=0.06004 p2=0.00000 p3=0.00000 w0=0.03370 w1=1.00000 w2=1.00000 | Not Allowed |  |
| **PMS2 Site Analysis** | | | | | | | | | |
| m0 | Sites | Homogeneous | 1 | 2 | -29148.89615 | N/A | w=0.19700 | No |  |
| m1Neutral | Sites | Site-specific | 1 | 2 | -27702.39377 | N/A | p0=0.69993 p1=0.30007 w0=0.06344 w1=1.00000 | Not Allowed |  |
| m2Selection | Sites | Site-specific | 2 | 0 | -27695.83883 | m2Selection | p0=0.69660 p1=0.28611 p2=0.01728 w0=0.06407 w1=1.00000 w2=2.44568 | Yes | Alignment (14 BEB sites): 396 409 468 469 479 521 541 546 547 553 557 562 564 602 |
| m3Discrtk2 | Sites | Site-specific | 3 | 2 | -27644.57217 | m3Discrtk2 | p0=0.65187 p1=0.34813 w0=0.04337 w1=0.64276 | No |  |
| m3Discrtk3 | Sites | Site-specific | 5 | 2 | -27460.91763 | m3Discrtk3 | p0=0.47362 p1=0.29225 p2=0.23413 w0=0.01250 w1=0.19537 w2=0.88039 | No |  |
| m7 | Sites | Site-specific | 2 | 2 | -27467.42544 | N/A | p=0.24066 q=0.69394 | Not Allowed |  |
| m8 | Sites | Site-specific | 4 | 2 | -27449.3651 | m8 | p=0.29104 p0=0.91064 p1=0.08936 q=1.31619 w=1.28855 | Yes | Alignment (37 BEB sites): 7 396 402 406 409 468 469 475 476 478 479 483 485 486 491 508 521 522 530 541 544 546 547 548 551 552 553 557 560 562 563 564 571 572 574 586 602 |
| m8a | Sites | Site-specific | 4 | 1 | -27452.45543 | N/A | p=0.32679 p0=0.84668 p1=0.15332 q=2.10213 w=1.00000 | Not Allowed |  |
| **PMS2 Branch-site Analysis** | | | | | | | | | |
| modelA | Chimpanzee | Branch-site | 3 | 2 | -27701.54411 | m1Neutral, modelAnull | p0=0.61147 p1=0.26171 p2=0.08881 p3=0.03801 w0=0.06333 w1=1.00000 w2=1.00000 | No |  |
| modelAnull | Chimpanzee | Branch-site | 3 | 1 | -27701.54411 | N/A | p0=0.61147 p1=0.26171 p2=0.08881 p3=0.03801 w0=0.06333 w1=1.00000 w2=1.00000 | Not Allowed |  |
| modelA | Euarchontoglires | Branch-site | 3 | 2 | -27702.39377 | m1Neutral, modelAnull | p0=0.69993 p1=0.30007 p2=0.00000 p3=0.00000 w0=0.06344 w1=1.00000 w2=1.00000 | No |  |
| modelAnull | Euarchontoglires | Branch-site | 3 | 1 | -27702.39377 | N/A | p0=0.69993 p1=0.30007 p2=0.00000 p3=0.00000 w0=0.06344 w1=1.00000 w2=1.00000 | Not Allowed |  |
| modelA | Glires | Branch-site | 3 | 2 | -27702.39377 | m1Neutral, modelAnull | p0=0.69993 p1=0.30007 p2=0.00000 p3=0.00000 w0=0.06344 w1=1.00000 w2=1.00000 | No |  |
| modelAnull | Glires | Branch-site | 3 | 1 | -27702.39377 | N/A | p0=0.69993 p1=0.30007 p2=0.00000 p3=0.00000 w0=0.06344 w1=1.00000 w2=1.00000 | Not Allowed |  |
| modelA | Gorilla | Branch-site | 3 | 2 | -27700.02079 | m1Neutral, modelAnull | p0=0.50883 p1=0.21820 p2=0.19105 p3=0.08193 w0=0.06303 w1=1.00000 w2=1.00000 | No |  |
| modelAnull | Gorilla | Branch-site | 3 | 1 | -27700.02079 | N/A | p0=0.50883 p1=0.21820 p2=0.19105 p3=0.08193 w0=0.06303 w1=1.00000 w2=1.00000 | Not Allowed |  |
| modelA | Guinea_Pig | Branch-site | 3 | 10 | -27702.39373 | m1Neutral, modelAnull | p0=0.69993 p1=0.30007 p2=0.00000 p3=0.00000 w0=0.06344 w1=1.00000 w2=27.68595 | No |  |
| modelAnull | Guinea_Pig | Branch-site | 3 | 1 | -27702.39377 | N/A | p0=0.69993 p1=0.30007 p2=0.00000 p3=0.00000 w0=0.06344 w1=1.00000 w2=1.00000 | Not Allowed |  |
| modelA | Homindae | Branch-site | 3 | 2 | -27701.44044 | m1Neutral, modelAnull | p0=0.69717 p1=0.29900 p2=0.00268 p3=0.00115 w0=0.06329 w1=1.00000 w2=80.69856 | No |  |
| modelAnull | Homindae | Branch-site | 3 | 1 | -27702.34903 | N/A | p0=0.68779 p1=0.29476 p2=0.01222 p3=0.00524 w0=0.06338 w1=1.00000 w2=1.00000 | Not Allowed |  |
| modelA | Homininae | Branch-site | 3 | 10 | -27702.39377 | m1Neutral, modelAnull | p0=0.69993 p1=0.30007 p2=0.00000 p3=0.00000 w0=0.06344 w1=1.00000 w2=7.52453 | No |  |
| modelAnull | Homininae | Branch-site | 3 | 1 | -27702.39377 | N/A | p0=0.69993 p1=0.30007 p2=0.00000 p3=0.00000 w0=0.06344 w1=1.00000 w2=1.00000 | Not Allowed |  |
| modelA | Hominini | Branch-site | 3 | 2 | -27701.91801 | m1Neutral, modelAnull | p0=0.67848 p1=0.29116 p2=0.02124 p3=0.00912 w0=0.06328 w1=1.00000 w2=5.31331 | No |  |
| modelAnull | Hominini | Branch-site | 3 | 1 | -27702.11523 | N/A | p0=0.63832 p1=0.27387 p2=0.06145 p3=0.02636 w0=0.06331 w1=1.00000 w2=1.00000 | Not Allowed |  |
| modelA | Human | Branch-site | 3 | 2 | -27702.39377 | m1Neutral, modelAnull | p0=0.69993 p1=0.30007 p2=0.00000 p3=0.00000 w0=0.06344 w1=1.00000 w2=1.00000 | No |  |
| modelAnull | Human | Branch-site | 3 | 1 | -27702.39377 | N/A | p0=0.69993 p1=0.30007 p2=0.00000 p3=0.00000 w0=0.06344 w1=1.00000 w2=1.00000 | Not Allowed |  |
| modelA | Marmoset | Branch-site | 3 | 2 | -27697.00877 | modelAnull | p0=0.62306 p1=0.26668 p2=0.07721 p3=0.03305 w0=0.06229 w1=1.00000 w2=1.00000 | No |  |
| modelAnull | Marmoset | Branch-site | 3 | 1 | -27697.00877 | N/A | p0=0.62306 p1=0.26668 p2=0.07721 p3=0.03305 w0=0.06229 w1=1.00000 w2=1.00000 | Not Allowed |  |
| modelA | Mouse | Branch-site | 3 | 2 | -27702.39377 | m1Neutral, modelAnull | p0=0.69993 p1=0.30007 p2=0.00000 p3=0.00000 w0=0.06344 w1=1.00000 w2=1.00000 | No |  |
| modelAnull | Mouse | Branch-site | 3 | 1 | -27702.39377 | N/A | p0=0.69993 p1=0.30007 p2=0.00000 p3=0.00000 w0=0.06344 w1=1.00000 w2=1.00000 | Not Allowed |  |
| modelA | Muridae | Branch-site | 3 | 2 | -27699.59257 | m1Neutral, modelAnull | p0=0.59672 p1=0.25473 p2=0.10411 p3=0.04444 w0=0.06290 w1=1.00000 w2=1.00000 | No |  |
| modelAnull | Muridae | Branch-site | 3 | 1 | -27699.59257 | N/A | p0=0.59672 p1=0.25473 p2=0.10411 p3=0.04444 w0=0.06290 w1=1.00000 w2=1.00000 | Not Allowed |  |
| modelA | Murinae | Branch-site | 3 | 2 | -27701.85722 | m1Neutral, modelAnull | p0=0.69627 p1=0.29865 p2=0.00356 p3=0.00153 w0=0.06314 w1=1.00000 w2=2.73234 | No |  |
| modelAnull | Murinae | Branch-site | 3 | 1 | -27702.0492 | N/A | p0=0.69296 p1=0.29722 p2=0.00688 p3=0.00295 w0=0.06313 w1=1.00000 w2=1.00000 | Not Allowed |  |
| modelA | Orangutan | Branch-site | 3 | 2 | -27699.92264 | m1Neutral, modelAnull | p0=0.68848 p1=0.29404 p2=0.01225 p3=0.00523 w0=0.06327 w1=1.00000 w2=13.90513 | No |  |
| modelAnull | Orangutan | Branch-site | 3 | 1 | -27700.66512 | N/A | p0=0.58442 p1=0.25011 p2=0.11588 p3=0.04959 w0=0.06318 w1=1.00000 w2=1.00000 | Not Allowed |  |
| modelA | Primates | Branch-site | 3 | 2 | -27702.39377 | m1Neutral, modelAnull | p0=0.69993 p1=0.30007 p2=0.00000 p3=0.00000 w0=0.06344 w1=1.00000 w2=1.00000 | No |  |
| modelAnull | Primates | Branch-site | 3 | 1 | -27702.39377 | N/A | p0=0.69993 p1=0.30007 p2=0.00000 p3=0.00000 w0=0.06344 w1=1.00000 w2=1.00000 | Not Allowed |  |
| modelA | Rabbit | Branch-site | 3 | 2 | -27701.96386 | m1Neutral, modelAnull | p0=0.69821 p1=0.29745 p2=0.00305 p3=0.00130 w0=0.06351 w1=1.00000 w2=130.53734 | No |  |
| modelAnull | Rabbit | Branch-site | 3 | 1 | -27702.39377 | N/A | p0=0.69993 p1=0.30007 p2=0.00000 p3=0.00000 w0=0.06344 w1=1.00000 w2=1.00000 | Not Allowed |  |
| modelA | Rat | Branch-site | 3 | 2 | -27700.76245 | m1Neutral, modelAnull | p0=0.65393 p1=0.28058 p2=0.04583 p3=0.01966 w0=0.06262 w1=1.00000 w2=1.00000 | No |  |
| modelAnull | Rat | Branch-site | 3 | 1 | -27700.76245 | N/A | p0=0.65393 p1=0.28058 p2=0.04583 p3=0.01966 w0=0.06262 w1=1.00000 w2=1.00000 | Not Allowed |  |
| **MUTYH Site Analysis** | | | | | | | | | |
| m0 | Sites | Homogeneous | 1 | 2 | -16473.91813 | N/A | w=0.22218 | No |  |
| m1Neutral | Sites | Site-specific | 1 | 2 | -15925.868 | N/A | p0=0.61956 p1=0.38044 w0=0.07926 w1=1.00000 | Not Allowed |  |
| m2Selection | Sites | Site-specific | 2 | 10 | -15920.08053 | m2Selection | p0=0.61676 p1=0.36920 p2=0.01404 w0=0.07945 w1=1.00000 w2=3.72592 | Yes | Alignment (8 BEB sites): 12 25 38 62 388 443 505 515 |
| m3Discrtk2 | Sites | Site-specific | 3 | 2 | -15878.10932 | m3Discrtk2 | p0=0.55639 p1=0.44361 w0=0.05076 w1=0.58471 | No |  |
| m3Discrtk3 | Sites | Site-specific | 5 | 2 | -15812.35814 | m3Discrtk3 | p0=0.35713 p1=0.36241 p2=0.28046 w0=0.01338 w1=0.20002 w2=0.81929 | No |  |
| m7 | Sites | Site-specific | 2 | 2 | -15808.22006 | N/A | p=0.34559 q=0.81941 | Not Allowed |  |
| m8 | Sites | Site-specific | 4 | 2 | -15797.6226 | m8 | p=0.37255 p0=0.97242 p1=0.02758 q=1.00900 w=2.44412 | Yes | Alignment (18 BEB sites): 5 11 12 25 27 36 38 62 290 355 382 388 409 443 489 498 505 515 |
| m8a | Sites | Site-specific | 4 | 1 | -15804.49256 | N/A | p=0.42167 p0=0.87064 p1=0.12936 q=1.67729 w=1.00000 | Not Allowed |  |
| **MUTYH Branch-site Analysis** | | | | | | | | | |
| modelA | Chimpanzee | Branch-site | 3 | 2 | -15925.868 | m1Neutral, modelAnull | p0=0.61956 p1=0.38044 p2=0.00000 p3=0.00000 w0=0.07926 w1=1.00000 w2=1.00000 | No |  |
| modelAnull | Chimpanzee | Branch-site | 3 | 1 | -15925.868 | N/A | p0=0.61956 p1=0.38044 p2=0.00000 p3=0.00000 w0=0.07926 w1=1.00000 w2=1.00000 | Not Allowed |  |
| modelA | Euarchontoglires | Branch-site | 3 | 10 | -15925.01989 | m1Neutral, modelAnull | p0=0.61549 p1=0.37865 p2=0.00363 p3=0.00223 w0=0.07907 w1=1.00000 w2=140.53103 | No |  |
| modelAnull | Euarchontoglires | Branch-site | 3 | 1 | -15925.868 | N/A | p0=0.61956 p1=0.38044 p2=0.00000 p3=0.00000 w0=0.07926 w1=1.00000 w2=1.00000 | Not Allowed |  |
| modelA | Glires | Branch-site | 3 | 2 | -15923.87848 | m1Neutral, modelAnull | p0=0.00000 p1=0.00000 p2=0.61902 p3=0.38098 w0=0.07859 w1=1.00000 w2=1.00000 | No |  |
| modelAnull | Glires | Branch-site | 3 | 1 | -15923.87848 | N/A | p0=0.00000 p1=0.00000 p2=0.61902 p3=0.38098 w0=0.07859 w1=1.00000 w2=1.00000 | Not Allowed |  |
| modelA | Gorilla | Branch-site | 3 | 2 | -15925.47616 | m1Neutral, modelAnull | p0=0.61685 p1=0.37895 p2=0.00261 p3=0.00160 w0=0.07906 w1=1.00000 w2=60.00253 | No |  |
| modelAnull | Gorilla | Branch-site | 3 | 1 | -15925.61104 | N/A | p0=0.54652 p1=0.33573 p2=0.07294 p3=0.04481 w0=0.07908 w1=1.00000 w2=1.00000 | Not Allowed |  |
| modelA | Guinea_Pig | Branch-site | 3 | 2 | -15925.868 | m1Neutral, modelAnull | p0=0.61956 p1=0.38044 p2=0.00000 p3=0.00000 w0=0.07926 w1=1.00000 w2=1.00001 | No |  |
| modelAnull | Guinea_Pig | Branch-site | 3 | 1 | -15925.868 | N/A | p0=0.61956 p1=0.38044 p2=0.00000 p3=0.00000 w0=0.07926 w1=1.00000 w2=1.00000 | Not Allowed |  |
| modelA | Homindae | Branch-site | 3 | 10 | -15925.74102 | m1Neutral, modelAnull | p0=0.61863 p1=0.37980 p2=0.00097 p3=0.00060 w0=0.07924 w1=1.00000 w2=57.64003 | No |  |
| modelAnull | Homindae | Branch-site | 3 | 1 | -15925.77777 | N/A | p0=0.59920 p1=0.36701 p2=0.02096 p3=0.01284 w0=0.07917 w1=1.00000 w2=1.00000 | Not Allowed |  |
| modelA | Homininae | Branch-site | 3 | 2 | -15925.868 | m1Neutral, modelAnull | p0=0.61956 p1=0.38044 p2=0.00000 p3=0.00000 w0=0.07926 w1=1.00000 w2=1.00000 | No |  |
| modelAnull | Homininae | Branch-site | 3 | 1 | -15925.868 | N/A | p0=0.61956 p1=0.38044 p2=0.00000 p3=0.00000 w0=0.07926 w1=1.00000 w2=1.00000 | Not Allowed |  |
| modelA | Hominini | Branch-site | 3 | 0 | -15925.86801 | m1Neutral, modelAnull | p0=0.49444 p1=0.30361 p2=0.12512 p3=0.07683 w0=0.07926 w1=1.00000 w2=1.07777 | No |  |
| modelAnull | Hominini | Branch-site | 3 | 1 | -15925.86801 | N/A | p0=0.51659 p1=0.31721 p2=0.10297 p3=0.06323 w0=0.07926 w1=1.00000 w2=1.00000 | Not Allowed |  |
| modelA | Human | Branch-site | 3 | 2 | -15925.868 | m1Neutral, modelAnull | p0=0.61956 p1=0.38044 p2=0.00000 p3=0.00000 w0=0.07926 w1=1.00000 w2=1.00000 | No |  |
| modelAnull | Human | Branch-site | 3 | 1 | -15925.868 | N/A | p0=0.61956 p1=0.38044 p2=0.00000 p3=0.00000 w0=0.07926 w1=1.00000 w2=1.00000 | Not Allowed |  |
| modelA | Marmoset | Branch-site | 3 | 10 | -15925.73635 | m1Neutral, modelAnull | p0=0.59934 p1=0.36824 p2=0.02008 p3=0.01234 w0=0.07893 w1=1.00000 w2=1.00000 | No |  |
| modelAnull | Marmoset | Branch-site | 3 | 1 | -15925.73635 | N/A | p0=0.59934 p1=0.36824 p2=0.02008 p3=0.01234 w0=0.07893 w1=1.00000 w2=1.00000 | Not Allowed |  |
| modelA | Mouse | Branch-site | 3 | 2 | -15925.868 | m1Neutral, modelAnull | p0=0.61956 p1=0.38044 p2=0.00000 p3=0.00000 w0=0.07926 w1=1.00000 w2=1.00000 | No |  |
| modelAnull | Mouse | Branch-site | 3 | 1 | -15925.868 | N/A | p0=0.61956 p1=0.38044 p2=0.00000 p3=0.00000 w0=0.07926 w1=1.00000 w2=1.00000 | Not Allowed |  |
| modelA | Muridae | Branch-site | 3 | 2 | -15925.868 | m1Neutral, modelAnull | p0=0.61956 p1=0.38044 p2=0.00000 p3=0.00000 w0=0.07926 w1=1.00000 w2=1.00000 | No |  |
| modelAnull | Muridae | Branch-site | 3 | 1 | -15925.868 | N/A | p0=0.61956 p1=0.38044 p2=0.00000 p3=0.00000 w0=0.07926 w1=1.00000 w2=1.00000 | Not Allowed |  |
| modelA | Murinae | Branch-site | 3 | 2 | -15923.20462 | m1Neutral, modelAnull | p0=0.57017 p1=0.34956 p2=0.04976 p3=0.03051 w0=0.07733 w1=1.00000 w2=1.10259 | No |  |
| modelAnull | Murinae | Branch-site | 3 | 1 | -15923.2065 | N/A | p0=0.56538 p1=0.34675 p2=0.05447 p3=0.03341 w0=0.07730 w1=1.00000 w2=1.00000 | Not Allowed |  |
| modelA | Orangutan | Branch-site | 3 | 2 | -15924.37119 | m1Neutral, modelAnull | p0=0.49167 p1=0.30195 p2=0.12786 p3=0.07852 w0=0.07850 w1=1.00000 w2=1.00000 | No |  |
| modelAnull | Orangutan | Branch-site | 3 | 1 | -15924.37119 | N/A | p0=0.49167 p1=0.30195 p2=0.12786 p3=0.07852 w0=0.07850 w1=1.00000 w2=1.00000 | Not Allowed |  |
| modelA | Primates | Branch-site | 3 | 10 | -15924.43512 | m1Neutral, modelAnull | p0=0.61771 p1=0.37990 p2=0.00148 p3=0.00091 w0=0.07918 w1=1.00000 w2=408.58757 | No |  |
| modelAnull | Primates | Branch-site | 3 | 1 | -15925.868 | N/A | p0=0.61956 p1=0.38044 p2=0.00000 p3=0.00000 w0=0.07926 w1=1.00000 w2=1.00000 | Not Allowed |  |
| modelA | Rabbit | Branch-site | 3 | 2 | -15911.6175 | modelA | p0=0.61027 p1=0.37605 p2=0.00846 p3=0.00522 w0=0.07703 w1=1.00000 w2=998.99697 | Yes | Alignment (5 BEB sites): 33 462 463 464 507 |
| modelAnull | Rabbit | Branch-site | 3 | 1 | -15921.35043 | N/A | p0=0.58566 p1=0.36031 p2=0.03345 p3=0.02058 w0=0.07688 w1=1.00000 w2=1.00000 | Not Allowed |  |
| modelA | Rat | Branch-site | 3 | 2 | -15925.68078 | m1Neutral, modelAnull | p0=0.59772 p1=0.36653 p2=0.02216 p3=0.01359 w0=0.07888 w1=1.00000 w2=1.00000 | No |  |
| modelAnull | Rat | Branch-site | 3 | 1 | -15925.68078 | N/A | p0=0.59772 p1=0.36653 p2=0.02216 p3=0.01359 w0=0.07888 w1=1.00000 w2=1.00000 | Not Allowed |  |
| **VHL Site Analysis** | | | | | | | | | |
| m0 | Sites | Homogeneous | 1 | 10 | -4357.285562 | N/A | w=0.14125 | No |  |
| m1Neutral | Sites | Site-specific | 1 | 2 | -4267.326674 | N/A | p0=0.74063 p1=0.25937 w0=0.05942 w1=1.00000 | Not Allowed |  |
| m2Selection | Sites | Site-specific | 2 | 2 | -4267.326674 | m1Neutral | p0=0.74063 p1=0.10653 p2=0.15284 w0=0.05942 w1=1.00000 w2=1.00000 | No |  |
| m3Discrtk2 | Sites | Site-specific | 3 | 2 | -4257.020117 | m3Discrtk2 | p0=0.62159 p1=0.37841 w0=0.04003 w1=0.45332 | No |  |
| m3Discrtk3 | Sites | Site-specific | 5 | 2 | -4237.251766 | m3Discrtk3 | p0=0.43061 p1=0.37295 p2=0.19644 w0=0.01387 w1=0.13536 w2=0.94822 | No |  |
| m7 | Sites | Site-specific | 2 | 2 | -4244.200844 | N/A | p=0.37215 q=1.39180 | Not Allowed |  |
| m8 | Sites | Site-specific | 4 | 2 | -4237.612263 | m8a | p=0.60851 p0=0.82230 p1=0.17770 q=6.75887 w=1.00691 | No |  |
| m8a | Sites | Site-specific | 4 | 1 | -4237.613008 | N/A | p=0.60959 p0=0.82167 p1=0.17833 q=6.80003 w=1.00000 | Not Allowed |  |
| **VHL Branch-site Analysis** | | | | | | | | | |
| modelA | Chimpanzee | Branch-site | 3 | 2 | -4262.098043 | modelA | p0=0.73571 p1=0.25251 p2=0.00877 p3=0.00301 w0=0.05976 w1=1.00000 w2=262.72662 | Yes | Alignment (3 BEB sites): 22 39 42 |
| modelAnull | Chimpanzee | Branch-site | 3 | 1 | -4266.712213 | N/A | p0=0.61202 p1=0.21104 p2=0.13157 p3=0.04537 w0=0.05849 w1=1.00000 w2=1.00000 | Not Allowed |  |
| modelA | Euarchontoglires | Branch-site | 3 | 2 | -4267.326674 | m1Neutral, modelAnull | p0=0.74063 p1=0.25937 p2=0.00000 p3=0.00000 w0=0.05942 w1=1.00000 w2=1.00000 | No |  |
| modelAnull | Euarchontoglires | Branch-site | 3 | 1 | -4267.326674 | N/A | p0=0.74063 p1=0.25937 p2=0.00000 p3=0.00000 w0=0.05942 w1=1.00000 w2=1.00000 | Not Allowed |  |
| modelA | Gorilla | Branch-site | 3 | 2 | -4267.326674 | m1Neutral, modelAnull | p0=0.74063 p1=0.25937 p2=0.00000 p3=0.00000 w0=0.05942 w1=1.00000 w2=1.00000 | No |  |
| modelAnull | Gorilla | Branch-site | 3 | 1 | -4267.326674 | N/A | p0=0.74063 p1=0.25937 p2=0.00000 p3=0.00000 w0=0.05942 w1=1.00000 w2=1.00000 | Not Allowed |  |
| modelA | Guinea_Pig | Branch-site | 3 | 2 | -4266.109645 | m1Neutral, modelAnull | p0=0.74197 p1=0.25046 p2=0.00566 p3=0.00191 w0=0.05952 w1=1.00000 w2=999.00000 | No |  |
| modelAnull | Guinea_Pig | Branch-site | 3 | 1 | -4267.326674 | N/A | p0=0.74063 p1=0.25937 p2=0.00000 p3=0.00000 w0=0.05942 w1=1.00000 w2=1.00000 | Not Allowed |  |
| modelA | Homindae | Branch-site | 3 | 2 | -4263.853291 | modelA | p0=0.73748 p1=0.25109 p2=0.00853 p3=0.00290 w0=0.05985 w1=1.00000 w2=220.34533 | Yes | Alignment (1 BEB sites): 8 |
| modelAnull | Homindae | Branch-site | 3 | 1 | -4267.326674 | N/A | p0=0.74063 p1=0.25937 p2=0.00000 p3=0.00000 w0=0.05942 w1=1.00000 w2=1.00000 | Not Allowed |  |
| modelA | Homininae | Branch-site | 3 | 2 | -4267.326674 | m1Neutral, modelAnull | p0=0.74063 p1=0.25937 p2=0.00000 p3=0.00000 w0=0.05942 w1=1.00000 w2=1.00000 | No |  |
| modelAnull | Homininae | Branch-site | 3 | 1 | -4267.326674 | N/A | p0=0.74063 p1=0.25937 p2=0.00000 p3=0.00000 w0=0.05942 w1=1.00000 w2=1.00000 | Not Allowed |  |
| modelA | Hominini | Branch-site | 3 | 2 | -4267.326674 | m1Neutral, modelAnull | p0=0.74063 p1=0.25937 p2=0.00000 p3=0.00000 w0=0.05942 w1=1.00000 w2=1.00000 | No |  |
| modelAnull | Hominini | Branch-site | 3 | 1 | -4267.326674 | N/A | p0=0.74063 p1=0.25937 p2=0.00000 p3=0.00000 w0=0.05942 w1=1.00000 w2=1.00000 | Not Allowed |  |
| modelA | Human | Branch-site | 3 | 2 | -4267.326674 | m1Neutral, modelAnull | p0=0.74061 p1=0.25936 p2=0.00002 p3=0.00001 w0=0.05942 w1=1.00000 w2=1.00000 | No |  |
| modelAnull | Human | Branch-site | 3 | 1 | -4267.326674 | N/A | p0=0.74061 p1=0.25936 p2=0.00002 p3=0.00001 w0=0.05942 w1=1.00000 w2=1.00000 | Not Allowed |  |
| modelA | Marmoset | Branch-site | 3 | 2 | -4262.443441 | modelA | p0=0.72045 p1=0.22453 p2=0.04195 p3=0.01307 w0=0.05886 w1=1.00000 w2=90.26952 | Yes | Alignment (10 BEB sites): 5 7 10 12 16 25 26 44 50 56 |
| modelAnull | Marmoset | Branch-site | 3 | 1 | -4267.133202 | N/A | p0=0.71712 p1=0.24269 p2=0.03003 p3=0.01016 w0=0.05850 w1=1.00000 w2=1.00000 | Not Allowed |  |
| modelA | Mouse | Branch-site | 3 | 2 | -4266.9278 | m1Neutral, modelAnull | p0=0.67852 p1=0.23560 p2=0.06375 p3=0.02214 w0=0.05734 w1=1.00000 w2=1.00000 | No |  |
| modelAnull | Mouse | Branch-site | 3 | 1 | -4266.9278 | N/A | p0=0.67852 p1=0.23560 p2=0.06375 p3=0.02214 w0=0.05734 w1=1.00000 w2=1.00000 | Not Allowed |  |
| modelA | Muridae | Branch-site | 3 | 2 | -4267.326674 | m1Neutral, modelAnull | p0=0.74063 p1=0.25937 p2=0.00000 p3=0.00000 w0=0.05942 w1=1.00000 w2=1.00000 | No |  |
| modelAnull | Muridae | Branch-site | 3 | 1 | -4267.326674 | N/A | p0=0.74063 p1=0.25937 p2=0.00000 p3=0.00000 w0=0.05942 w1=1.00000 w2=1.00000 | Not Allowed |  |
| modelA | Murinae | Branch-site | 3 | 10 | -4267.045345 | m1Neutral, modelAnull | p0=0.73663 p1=0.25568 p2=0.00571 p3=0.00198 w0=0.05949 w1=1.00000 w2=998.96785 | No |  |
| modelAnull | Murinae | Branch-site | 3 | 1 | -4267.326674 | N/A | p0=0.74063 p1=0.25937 p2=0.00000 p3=0.00000 w0=0.05942 w1=1.00000 w2=1.00000 | Not Allowed |  |
| modelA | Orangutan | Branch-site | 3 | 2 | -4267.307312 | m1Neutral, modelAnull | p0=0.72559 p1=0.25399 p2=0.01513 p3=0.00530 w0=0.05919 w1=1.00000 w2=1.00000 | No |  |
| modelAnull | Orangutan | Branch-site | 3 | 1 | -4267.307312 | N/A | p0=0.72559 p1=0.25399 p2=0.01513 p3=0.00530 w0=0.05919 w1=1.00000 w2=1.00000 | Not Allowed |  |
| modelA | Primates | Branch-site | 3 | 2 | -4267.326674 | m1Neutral, modelAnull | p0=0.74063 p1=0.25937 p2=0.00000 p3=0.00000 w0=0.05942 w1=1.00000 w2=1.00000 | No |  |
| modelAnull | Primates | Branch-site | 3 | 1 | -4267.326674 | N/A | p0=0.74063 p1=0.25937 p2=0.00000 p3=0.00000 w0=0.05942 w1=1.00000 w2=1.00000 | Not Allowed |  |
| modelA | Rat | Branch-site | 3 | 2 | -4267.119278 | m1Neutral, modelAnull | p0=0.74044 p1=0.25217 p2=0.00551 p3=0.00188 w0=0.05995 w1=1.00000 w2=10.09517 | No |  |
| modelAnull | Rat | Branch-site | 3 | 1 | -4267.326674 | N/A | p0=0.74063 p1=0.25937 p2=0.00000 p3=0.00000 w0=0.05942 w1=1.00000 w2=1.00000 | Not Allowed |  |
| **APC Site Analysis** | | | | | | | | | |
| m0 | Sites | Homogeneous | 1 | 2 | -60135.91923 | N/A | w=0.07671 | No |  |
| m1Neutral | Sites | Site-specific | 1 | 2 | -59006.82299 | N/A | p0=0.87177 p1=0.12823 w0=0.04049 w1=1.00000 | Not Allowed |  |
| m2Selection | Sites | Site-specific | 2 | 2 | -59006.82299 | m1Neutral | p0=0.87177 p1=0.04560 p2=0.08263 w0=0.04049 w1=1.00000 w2=1.00000 | No |  |
| m3Discrtk2 | Sites | Site-specific | 3 | 2 | -58385.71977 | m3Discrtk2 | p0=0.73327 p1=0.26673 w0=0.01512 w1=0.28692 | No |  |
| m3Discrtk3 | Sites | Site-specific | 5 | 2 | -58279.77636 | m3Discrtk3 | p0=0.62334 p1=0.26492 p2=0.11174 w0=0.00729 w1=0.12350 w2=0.48342 | No |  |
| m7 | Sites | Site-specific | 2 | 2 | -58281.5485 | N/A | p=0.23606 q=2.18351 | Not Allowed |  |
| m8 | Sites | Site-specific | 4 | 2 | -58272.08079 | m8a | p=0.25185 p0=0.98835 p1=0.01165 q=2.66372 w=1.00451 | No |  |
| m8a | Sites | Site-specific | 4 | 1 | -58272.08092 | N/A | p=0.25196 p0=0.98822 p1=0.01178 q=2.66767 w=1.00000 | Not Allowed |  |
| **APC Branch-site Analysis** | | | | | | | | | |
| modelA | Chimpanzee | Branch-site | 3 | 2 | -59004.42105 | m1Neutral, modelAnull | p0=0.74687 p1=0.11005 p2=0.12470 p3=0.01837 w0=0.04032 w1=1.00000 w2=1.00000 | No |  |
| modelAnull | Chimpanzee | Branch-site | 3 | 1 | -59004.42105 | N/A | p0=0.74687 p1=0.11005 p2=0.12470 p3=0.01838 w0=0.04032 w1=1.00000 w2=1.00000 | Not Allowed |  |
| modelA | Euarchontoglires | Branch-site | 3 | 0 | -59006.73033 | m1Neutral, modelAnull | p0=0.86086 p1=0.12664 p2=0.01090 p3=0.00160 w0=0.04045 w1=1.00000 w2=1.00000 | No |  |
| modelAnull | Euarchontoglires | Branch-site | 3 | 1 | -59006.82299 | N/A | p0=0.87177 p1=0.12823 p2=0.00000 p3=0.00000 w0=0.04049 w1=1.00000 w2=1.00000 | Not Allowed |  |
| modelA | Glires | Branch-site | 3 | 2 | -59006.82299 | m1Neutral, modelAnull | p0=0.87177 p1=0.12823 p2=0.00000 p3=0.00000 w0=0.04049 w1=1.00000 w2=1.00000 | No |  |
| modelAnull | Glires | Branch-site | 3 | 1 | -59006.82299 | N/A | p0=0.87177 p1=0.12823 p2=0.00000 p3=0.00000 w0=0.04049 w1=1.00000 w2=1.00000 | Not Allowed |  |
| modelA | Gorilla | Branch-site | 3 | 2 | -59006.54483 | m1Neutral, modelAnull | p0=0.83699 p1=0.12298 p2=0.03490 p3=0.00513 w0=0.04047 w1=1.00000 w2=1.00000 | No |  |
| modelAnull | Gorilla | Branch-site | 3 | 1 | -59006.54483 | N/A | p0=0.83698 p1=0.12298 p2=0.03491 p3=0.00513 w0=0.04047 w1=1.00000 w2=1.00000 | Not Allowed |  |
| modelA | Guinea_Pig | Branch-site | 3 | 0 | -59006.55865 | m1Neutral, modelAnull | p0=0.86437 p1=0.12715 p2=0.00739 p3=0.00109 w0=0.04028 w1=1.00000 w2=1.00000 | No |  |
| modelAnull | Guinea_Pig | Branch-site | 3 | 1 | -59006.55865 | N/A | p0=0.86437 p1=0.12715 p2=0.00739 p3=0.00109 w0=0.04028 w1=1.00000 w2=1.00000 | Not Allowed |  |
| modelA | Homindae | Branch-site | 3 | 2 | -59006.82299 | m1Neutral, modelAnull | p0=0.87177 p1=0.12823 p2=0.00000 p3=0.00000 w0=0.04049 w1=1.00000 w2=1.00000 | No |  |
| modelAnull | Homindae | Branch-site | 3 | 1 | -59006.82299 | N/A | p0=0.87177 p1=0.12823 p2=0.00000 p3=0.00000 w0=0.04049 w1=1.00000 w2=1.00000 | Not Allowed |  |
| modelA | Homininae | Branch-site | 3 | 2 | -59006.82299 | m1Neutral, modelAnull | p0=0.87177 p1=0.12823 p2=0.00000 p3=0.00000 w0=0.04049 w1=1.00000 w2=1.00000 | No |  |
| modelAnull | Homininae | Branch-site | 3 | 1 | -59006.82299 | N/A | p0=0.87177 p1=0.12823 p2=0.00000 p3=0.00000 w0=0.04049 w1=1.00000 w2=1.00000 | Not Allowed |  |
| modelA | Hominini | Branch-site | 3 | 10 | -59006.82299 | m1Neutral, modelAnull | p0=0.87177 p1=0.12823 p2=0.00000 p3=0.00000 w0=0.04049 w1=1.00000 w2=8.34898 | No |  |
| modelAnull | Hominini | Branch-site | 3 | 1 | -59006.82299 | N/A | p0=0.87177 p1=0.12823 p2=0.00000 p3=0.00000 w0=0.04049 w1=1.00000 w2=1.00000 | Not Allowed |  |
| modelA | Human | Branch-site | 3 | 2 | -58999.43396 | modelAnull | p0=0.51149 p1=0.07525 p2=0.36027 p3=0.05300 w0=0.04025 w1=1.00000 w2=1.00000 | No |  |
| modelAnull | Human | Branch-site | 3 | 1 | -58999.43396 | N/A | p0=0.51147 p1=0.07524 p2=0.36029 p3=0.05300 w0=0.04025 w1=1.00000 w2=1.00000 | Not Allowed |  |
| modelA | Marmoset | Branch-site | 3 | 10 | -59006.82176 | m1Neutral, modelAnull | p0=0.87176 p1=0.12823 p2=0.00001 p3=0.00000 w0=0.04049 w1=1.00000 w2=9.61162 | No |  |
| modelAnull | Marmoset | Branch-site | 3 | 1 | -59006.82299 | N/A | p0=0.87177 p1=0.12823 p2=0.00000 p3=0.00000 w0=0.04049 w1=1.00000 w2=1.00000 | Not Allowed |  |
| modelA | Mouse | Branch-site | 3 | 2 | -59000.96925 | modelAnull | p0=0.82928 p1=0.12173 p2=0.04272 p3=0.00627 w0=0.03963 w1=1.00000 w2=1.00000 | No |  |
| modelAnull | Mouse | Branch-site | 3 | 1 | -59000.96925 | N/A | p0=0.82928 p1=0.12173 p2=0.04272 p3=0.00627 w0=0.03963 w1=1.00000 w2=1.00000 | Not Allowed |  |
| modelA | Muridae | Branch-site | 3 | 2 | -59003.93455 | m1Neutral | p0=0.87159 p1=0.12783 p2=0.00051 p3=0.00007 w0=0.04047 w1=1.00000 w2=53.52147 | No |  |
| modelAnull | Muridae | Branch-site | 3 | 1 | -59006.82299 | N/A | p0=0.87177 p1=0.12823 p2=0.00000 p3=0.00000 w0=0.04049 w1=1.00000 w2=1.00000 | Not Allowed |  |
| modelA | Murinae | Branch-site | 3 | 10 | -59006.82299 | m1Neutral, modelAnull | p0=0.87177 p1=0.12823 p2=0.00000 p3=0.00000 w0=0.04049 w1=1.00000 w2=44.34084 | No |  |
| modelAnull | Murinae | Branch-site | 3 | 1 | -59006.82319 | N/A | p0=0.87177 p1=0.12823 p2=0.00000 p3=0.00000 w0=0.04049 w1=1.00000 w2=1.00000 | Not Allowed |  |
| modelA | Orangutan | Branch-site | 3 | 2 | -59006.63122 | m1Neutral, modelAnull | p0=0.85389 p1=0.12552 p2=0.01795 p3=0.00264 w0=0.04045 w1=1.00000 w2=1.00000 | No |  |
| modelAnull | Orangutan | Branch-site | 3 | 1 | -59006.82299 | N/A | p0=0.87177 p1=0.12823 p2=0.00000 p3=0.00000 w0=0.04049 w1=1.00000 w2=1.00000 | Not Allowed |  |
| modelA | Primates | Branch-site | 3 | 2 | -59006.82299 | m1Neutral, modelAnull | p0=0.87177 p1=0.12823 p2=0.00000 p3=0.00000 w0=0.04049 w1=1.00000 w2=1.00000 | No |  |
| modelAnull | Primates | Branch-site | 3 | 1 | -59006.82299 | N/A | p0=0.87177 p1=0.12823 p2=0.00000 p3=0.00000 w0=0.04049 w1=1.00000 w2=1.00000 | Not Allowed |  |
| modelA | Rabbit | Branch-site | 3 | 1 | -59006.47528 | m1Neutral, modelAnull | p0=0.87119 p1=0.12821 p2=0.00052 p3=0.00008 w0=0.04041 w1=1.00000 w2=4.75061 | No |  |
| modelAnull | Rabbit | Branch-site | 3 | 1 | -59006.74568 | N/A | p0=0.87069 p1=0.12810 p2=0.00105 p3=0.00015 w0=0.04043 w1=1.00000 w2=1.00000 | Not Allowed |  |
| modelA | Rat | Branch-site | 3 | 2 | -59003.44297 | modelAnull | p0=0.84721 p1=0.12470 p2=0.02449 p3=0.00360 w0=0.03974 w1=1.00000 w2=1.00000 | No |  |
| modelAnull | Rat | Branch-site | 3 | 1 | -59003.44297 | N/A | p0=0.84721 p1=0.12470 p2=0.02449 p3=0.00360 w0=0.03974 w1=1.00000 w2=1.00000 | Not Allowed |  |
| **TP53 Site Analysis** | | | | | | | | | |
| m0 | Sites | Homogeneous | 1 | 2 | -9031.169748 | N/A | w=0.17276 | No |  |
| m1Neutral | Sites | Site-specific | 1 | 2 | -8783.358904 | N/A | p0=0.66545 p1=0.33455 w0=0.06391 w1=1.00000 | Not Allowed |  |
| m2Selection | Sites | Site-specific | 2 | 10 | -8779.527606 | m2Selection | p0=0.65799 p1=0.32557 p2=0.01644 w0=0.06416 w1=1.00000 w2=4.24525 | Yes | Alignment (6 BEB sites): 47 50 51 52 56 126 |
| m3Discrtk2 | Sites | Site-specific | 3 | 2 | -8738.043103 | m3Discrtk2 | p0=0.53498 p1=0.46502 w0=0.03362 w1=0.44780 | No |  |
| m3Discrtk3 | Sites | Site-specific | 5 | 2 | -8693.974583 | m3Discrtk3 | p0=0.44007 p1=0.44191 p2=0.11801 w0=0.01652 w1=0.23659 w2=1.31181 | Yes | Alignment (42 NEB sites): 4 10 31 35 37 38 39 40 46 47 48 49 50 51 52 53 55 56 58 61 62 64 67 68 69 70 74 75 78 79 103 126 145 182 302 309 316 317 354 355 378 382 |
| m7 | Sites | Site-specific | 2 | 2 | -8700.084322 | N/A | p=0.34010 q=1.07263 | Not Allowed |  |
| m8 | Sites | Site-specific | 4 | 2 | -8688.19126 | m8 | p=0.40362 p0=0.94645 p1=0.05355 q=1.77507 w=1.97385 | Yes | Alignment (14 BEB sites): 39 46 47 49 50 51 52 53 56 69 78 126 317 354 |
| m8a | Sites | Site-specific | 4 | 1 | -8693.911882 | N/A | p=0.44791 p0=0.88154 p1=0.11846 q=2.73989 w=1.00000 | Not Allowed |  |
| **TP53 Branch-site Analysis** | | | | | | | | | |
| modelA | Chimpanzee | Branch-site | 3 | 2 | -8783.358904 | m1Neutral, modelAnull | p0=0.66545 p1=0.33455 p2=0.00000 p3=0.00000 w0=0.06391 w1=1.00000 w2=1.00000 | No |  |
| modelAnull | Chimpanzee | Branch-site | 3 | 1 | -8783.358904 | N/A | p0=0.66545 p1=0.33455 p2=0.00000 p3=0.00000 w0=0.06391 w1=1.00000 w2=1.00000 | Not Allowed |  |
| modelA | Euarchontoglires | Branch-site | 3 | 2 | -8783.354599 | m1Neutral, modelAnull | p0=0.66077 p1=0.33230 p2=0.00461 p3=0.00232 w0=0.06383 w1=1.00000 w2=1.00000 | No |  |
| modelAnull | Euarchontoglires | Branch-site | 3 | 1 | -8783.354599 | N/A | p0=0.66077 p1=0.33230 p2=0.00461 p3=0.00232 w0=0.06383 w1=1.00000 w2=1.00000 | Not Allowed |  |
| modelA | Glires | Branch-site | 3 | 2 | -8783.23559 | m1Neutral, modelAnull | p0=0.63861 p1=0.32176 p2=0.02635 p3=0.01328 w0=0.06343 w1=1.00000 w2=1.00000 | No |  |
| modelAnull | Glires | Branch-site | 3 | 1 | -8783.23559 | N/A | p0=0.63861 p1=0.32176 p2=0.02635 p3=0.01328 w0=0.06343 w1=1.00000 w2=1.00000 | Not Allowed |  |
| modelA | Gorilla | Branch-site | 3 | 2 | -8783.358904 | m1Neutral, modelAnull | p0=0.66545 p1=0.33455 p2=0.00000 p3=0.00000 w0=0.06391 w1=1.00000 w2=1.00000 | No |  |
| modelAnull | Gorilla | Branch-site | 3 | 1 | -8783.358904 | N/A | p0=0.66545 p1=0.33455 p2=0.00000 p3=0.00000 w0=0.06391 w1=1.00000 w2=1.00000 | Not Allowed |  |
| modelA | Guinea_Pig | Branch-site | 3 | 2 | -8781.361788 | m1Neutral, modelAnull | p0=0.65493 p1=0.32434 p2=0.01386 p3=0.00687 w0=0.06151 w1=1.00000 w2=2.51912 | No |  |
| modelAnull | Guinea_Pig | Branch-site | 3 | 1 | -8781.597067 | N/A | p0=0.63950 p1=0.31745 p2=0.02877 p3=0.01428 w0=0.06113 w1=1.00000 w2=1.00000 | Not Allowed |  |
| modelA | Homindae | Branch-site | 3 | 2 | -8782.558335 | m1Neutral, modelAnull | p0=0.66894 p1=0.32440 p2=0.00448 p3=0.00217 w0=0.06442 w1=1.00000 w2=22.49066 | No |  |
| modelAnull | Homindae | Branch-site | 3 | 1 | -8783.358904 | N/A | p0=0.66545 p1=0.33455 p2=0.00000 p3=0.00000 w0=0.06391 w1=1.00000 w2=1.00000 | Not Allowed |  |
| modelA | Homininae | Branch-site | 3 | 2 | -8783.353113 | m1Neutral, modelAnull | p0=0.65935 p1=0.33169 p2=0.00596 p3=0.00300 w0=0.06383 w1=1.00000 w2=1.00000 | No |  |
| modelAnull | Homininae | Branch-site | 3 | 1 | -8783.353113 | N/A | p0=0.65935 p1=0.33169 p2=0.00596 p3=0.00300 w0=0.06383 w1=1.00000 w2=1.00000 | Not Allowed |  |
| modelA | Hominini | Branch-site | 3 | 1 | -8783.358906 | m1Neutral, modelAnull | p0=0.65083 p1=0.32720 p2=0.01462 p3=0.00735 w0=0.06391 w1=1.00000 w2=1.19189 | No |  |
| modelAnull | Hominini | Branch-site | 3 | 1 | -8783.358919 | N/A | p0=0.52536 p1=0.26412 p2=0.14009 p3=0.07043 w0=0.06391 w1=1.00000 w2=1.00000 | Not Allowed |  |
| modelA | Human | Branch-site | 3 | 2 | -8783.358904 | m1Neutral, modelAnull | p0=0.66543 p1=0.33455 p2=0.00002 p3=0.00001 w0=0.06391 w1=1.00000 w2=1.00000 | No |  |
| modelAnull | Human | Branch-site | 3 | 1 | -8783.358904 | N/A | p0=0.66503 p1=0.33435 p2=0.00041 p3=0.00021 w0=0.06391 w1=1.00000 w2=1.00000 | Not Allowed |  |
| modelA | Marmoset | Branch-site | 3 | 2 | -8783.168484 | m1Neutral, modelAnull | p0=0.66079 p1=0.32743 p2=0.00787 p3=0.00390 w0=0.06338 w1=1.00000 w2=4.11155 | No |  |
| modelAnull | Marmoset | Branch-site | 3 | 1 | -8783.18923 | N/A | p0=0.64361 p1=0.31846 p2=0.02537 p3=0.01255 w0=0.06335 w1=1.00000 w2=1.00000 | Not Allowed |  |
| modelA | Mouse | Branch-site | 3 | 10 | -8783.358903 | m1Neutral, modelAnull | p0=0.66545 p1=0.33455 p2=0.00000 p3=0.00000 w0=0.06391 w1=1.00000 w2=27.98316 | No |  |
| modelAnull | Mouse | Branch-site | 3 | 1 | -8783.358904 | N/A | p0=0.66545 p1=0.33455 p2=0.00000 p3=0.00000 w0=0.06391 w1=1.00000 w2=1.00000 | Not Allowed |  |
| modelA | Muridae | Branch-site | 3 | 2 | -8783.329713 | m1Neutral, modelAnull | p0=0.64026 p1=0.32160 p2=0.02539 p3=0.01275 w0=0.06372 w1=1.00000 w2=1.00000 | No |  |
| modelAnull | Muridae | Branch-site | 3 | 1 | -8783.329713 | N/A | p0=0.64026 p1=0.32160 p2=0.02539 p3=0.01275 w0=0.06372 w1=1.00000 w2=1.00000 | Not Allowed |  |
| modelA | Orangutan | Branch-site | 3 | 2 | -8783.358904 | m1Neutral, modelAnull | p0=0.66545 p1=0.33455 p2=0.00000 p3=0.00000 w0=0.06391 w1=1.00000 w2=1.00000 | No |  |
| modelAnull | Orangutan | Branch-site | 3 | 1 | -8783.358904 | N/A | p0=0.66545 p1=0.33455 p2=0.00000 p3=0.00000 w0=0.06391 w1=1.00000 w2=1.00000 | Not Allowed |  |
| modelA | Primates | Branch-site | 3 | 10 | -8781.979814 | m1Neutral, modelAnull | p0=0.65653 p1=0.33389 p2=0.00635 p3=0.00323 w0=0.06361 w1=1.00000 w2=67.66565 | No |  |
| modelAnull | Primates | Branch-site | 3 | 1 | -8783.358904 | N/A | p0=0.66545 p1=0.33455 p2=0.00000 p3=0.00000 w0=0.06391 w1=1.00000 w2=1.00000 | Not Allowed |  |
| modelA | Rabbit | Branch-site | 3 | 2 | -8783.358904 | m1Neutral, modelAnull | p0=0.66545 p1=0.33455 p2=0.00000 p3=0.00000 w0=0.06391 w1=1.00000 w2=12.87156 | No |  |
| modelAnull | Rabbit | Branch-site | 3 | 1 | -8783.358904 | N/A | p0=0.66545 p1=0.33455 p2=0.00000 p3=0.00000 w0=0.06391 w1=1.00000 w2=1.00000 | Not Allowed |  |
| **MADH4 Site Analysis** | | | | | | | | | |
| m0 | Sites | Homogeneous | 1 | 2 | -6194.2839 | N/A | w=0.02977 | No |  |
| m1Neutral | Sites | Site-specific | 1 | 2 | -6186.752724 | N/A | p0=0.98112 p1=0.01888 w0=0.02423 w1=1.00000 | Not Allowed |  |
| m2Selection | Sites | Site-specific | 2 | 0 | -6186.752724 | m1Neutral | p0=0.98112 p1=0.01888 p2=0.00000 w0=0.02423 w1=1.00000 w2=18.97887 | No |  |
| m3Discrtk2 | Sites | Site-specific | 3 | 0 | -6171.476355 | m3Discrtk2 | p0=0.83555 p1=0.16445 w0=0.00775 w1=0.16225 | No |  |
| m3Discrtk3 | Sites | Site-specific | 5 | 0 | -6171.476355 | m3Discrtk2 | p0=0.18738 p1=0.64817 p2=0.16445 w0=0.00775 w1=0.00776 w2=0.16224 | No |  |
| m7 | Sites | Site-specific | 2 | 2 | -6171.570512 | N/A | p=0.19813 q=5.19791 | Not Allowed |  |
| m8 | Sites | Site-specific | 4 | 2 | -6171.572866 | m7, m8a | p=0.19816 p0=0.99999 p1=0.00001 q=5.19895 w=1.00000 | No |  |
| m8a | Sites | Site-specific | 4 | 1 | -6171.572866 | N/A | p=0.19815 p0=0.99999 p1=0.00001 q=5.19885 w=1.00000 | Not Allowed |  |
| **MADH4 Branch-site Analysis** | | | | | | | | | |
| modelA | Chimpanzee | Branch-site | 3 | 2 | -6186.752724 | m1Neutral, modelAnull | p0=0.98112 p1=0.01888 p2=0.00000 p3=0.00000 w0=0.02423 w1=1.00000 w2=17.54666 | No |  |
| modelAnull | Chimpanzee | Branch-site | 3 | 1 | -6186.752726 | N/A | p0=0.98112 p1=0.01888 p2=0.00000 p3=0.00000 w0=0.02423 w1=1.00000 w2=1.00000 | Not Allowed |  |
| modelA | Euarchontoglires | Branch-site | 3 | 10 | -6186.752724 | m1Neutral, modelAnull | p0=0.98112 p1=0.01888 p2=0.00000 p3=0.00000 w0=0.02423 w1=1.00000 w2=9.53493 | No |  |
| modelAnull | Euarchontoglires | Branch-site | 3 | 1 | -6186.752725 | N/A | p0=0.98112 p1=0.01888 p2=0.00000 p3=0.00000 w0=0.02423 w1=1.00000 w2=1.00000 | Not Allowed |  |
| modelA | Glires | Branch-site | 3 | 2 | -6186.752724 | m1Neutral, modelAnull | p0=0.98109 p1=0.01888 p2=0.00003 p3=0.00000 w0=0.02423 w1=1.00000 w2=1.00000 | No |  |
| modelAnull | Glires | Branch-site | 3 | 1 | -6186.752724 | N/A | p0=0.98105 p1=0.01888 p2=0.00006 p3=0.00000 w0=0.02423 w1=1.00000 w2=1.00000 | Not Allowed |  |
| modelA | Gorilla | Branch-site | 3 | 2 | -6186.752724 | m1Neutral, modelAnull | p0=0.98112 p1=0.01888 p2=0.00000 p3=0.00000 w0=0.02423 w1=1.00000 w2=1.00000 | No |  |
| modelAnull | Gorilla | Branch-site | 3 | 1 | -6186.752724 | N/A | p0=0.98112 p1=0.01888 p2=0.00000 p3=0.00000 w0=0.02423 w1=1.00000 w2=1.00000 | Not Allowed |  |
| modelA | Guinea_Pig | Branch-site | 3 | 0 | -6186.752724 | m1Neutral, modelAnull | p0=0.98112 p1=0.01888 p2=0.00000 p3=0.00000 w0=0.02423 w1=1.00000 w2=1.00000 | No |  |
| modelAnull | Guinea_Pig | Branch-site | 3 | 1 | -6186.752724 | N/A | p0=0.98112 p1=0.01888 p2=0.00000 p3=0.00000 w0=0.02423 w1=1.00000 w2=1.00000 | Not Allowed |  |
| modelA | Homindae | Branch-site | 3 | 2 | -6186.752724 | m1Neutral, modelAnull | p0=0.98112 p1=0.01888 p2=0.00000 p3=0.00000 w0=0.02423 w1=1.00000 w2=1.00000 | No |  |
| modelAnull | Homindae | Branch-site | 3 | 1 | -6186.752724 | N/A | p0=0.98112 p1=0.01888 p2=0.00000 p3=0.00000 w0=0.02423 w1=1.00000 w2=1.00000 | Not Allowed |  |
| modelA | Homininae | Branch-site | 3 | 2 | -6186.752724 | m1Neutral, modelAnull | p0=0.98112 p1=0.01888 p2=0.00000 p3=0.00000 w0=0.02423 w1=1.00000 w2=1.00000 | No |  |
| modelAnull | Homininae | Branch-site | 3 | 1 | -6186.752724 | N/A | p0=0.98112 p1=0.01888 p2=0.00000 p3=0.00000 w0=0.02423 w1=1.00000 w2=1.00000 | Not Allowed |  |
| modelA | Hominini | Branch-site | 3 | 2 | -6186.752781 | m1Neutral, modelAnull | p0=0.96449 p1=0.01856 p2=0.01663 p3=0.00032 w0=0.02423 w1=1.00000 w2=3.32599 | No |  |
| modelAnull | Hominini | Branch-site | 3 | 1 | -6186.752787 | N/A | p0=0.91701 p1=0.01765 p2=0.06411 p3=0.00123 w0=0.02423 w1=1.00000 w2=1.00000 | Not Allowed |  |
| modelA | Human | Branch-site | 3 | 2 | -6186.752724 | m1Neutral, modelAnull | p0=0.98112 p1=0.01888 p2=0.00000 p3=0.00000 w0=0.02423 w1=1.00000 w2=1.00000 | No |  |
| modelAnull | Human | Branch-site | 3 | 1 | -6186.752724 | N/A | p0=0.98112 p1=0.01888 p2=0.00000 p3=0.00000 w0=0.02423 w1=1.00000 w2=1.00000 | Not Allowed |  |
| modelA | Marmoset | Branch-site | 3 | 2 | -6186.752724 | m1Neutral, modelAnull | p0=0.98112 p1=0.01888 p2=0.00000 p3=0.00000 w0=0.02423 w1=1.00000 w2=1.00000 | No |  |
| modelAnull | Marmoset | Branch-site | 3 | 1 | -6186.752808 | N/A | p0=0.98112 p1=0.01888 p2=0.00000 p3=0.00000 w0=0.02423 w1=1.00000 w2=1.00000 | Not Allowed |  |
| modelA | Mouse | Branch-site | 3 | 2 | -6186.752724 | m1Neutral, modelAnull | p0=0.98112 p1=0.01888 p2=0.00000 p3=0.00000 w0=0.02423 w1=1.00000 w2=1.00000 | No |  |
| modelAnull | Mouse | Branch-site | 3 | 1 | -6186.752724 | N/A | p0=0.98112 p1=0.01888 p2=0.00000 p3=0.00000 w0=0.02423 w1=1.00000 w2=1.00000 | Not Allowed |  |
| modelA | Muridae | Branch-site | 3 | 2 | -6186.752724 | m1Neutral, modelAnull | p0=0.98112 p1=0.01888 p2=0.00000 p3=0.00000 w0=0.02423 w1=1.00000 w2=1.00000 | No |  |
| modelAnull | Muridae | Branch-site | 3 | 1 | -6186.752725 | N/A | p0=0.98112 p1=0.01888 p2=0.00000 p3=0.00000 w0=0.02423 w1=1.00000 w2=1.00000 | Not Allowed |  |
| modelA | Murinae | Branch-site | 3 | 2 | -6186.752724 | m1Neutral, modelAnull | p0=0.98112 p1=0.01888 p2=0.00000 p3=0.00000 w0=0.02423 w1=1.00000 w2=1.00000 | No |  |
| modelAnull | Murinae | Branch-site | 3 | 1 | -6186.752724 | N/A | p0=0.98112 p1=0.01888 p2=0.00000 p3=0.00000 w0=0.02423 w1=1.00000 w2=1.00000 | Not Allowed |  |
| modelA | Orangutan | Branch-site | 3 | 10 | -6186.752724 | m1Neutral, modelAnull | p0=0.98112 p1=0.01888 p2=0.00000 p3=0.00000 w0=0.02423 w1=1.00000 w2=8.64460 | No |  |
| modelAnull | Orangutan | Branch-site | 3 | 1 | -6186.752724 | N/A | p0=0.98112 p1=0.01888 p2=0.00000 p3=0.00000 w0=0.02423 w1=1.00000 w2=1.00000 | Not Allowed |  |
| modelA | Primates | Branch-site | 3 | 2 | -6185.716072 | m1Neutral, modelAnull | p0=0.95139 p1=0.01815 p2=0.02988 p3=0.00057 w0=0.02365 w1=1.00000 w2=1.75529 | No |  |
| modelAnull | Primates | Branch-site | 3 | 1 | -6185.720196 | N/A | p0=0.93047 p1=0.01777 p2=0.05080 p3=0.00097 w0=0.02364 w1=1.00000 w2=1.00000 | Not Allowed |  |
| modelA | Rabbit | Branch-site | 3 | 2 | -6186.752724 | m1Neutral, modelAnull | p0=0.98112 p1=0.01888 p2=0.00000 p3=0.00000 w0=0.02423 w1=1.00000 w2=1.00000 | No |  |
| modelAnull | Rabbit | Branch-site | 3 | 1 | -6186.752725 | N/A | p0=0.98112 p1=0.01888 p2=0.00000 p3=0.00000 w0=0.02423 w1=1.00000 w2=1.00000 | Not Allowed |  |
| modelA | Rat | Branch-site | 3 | 10 | -6092.186945 | modelA | p0=0.93360 p1=0.01536 p2=0.05021 p3=0.00083 w0=0.01379 w1=1.00000 w2=102.33013 | Yes | Alignment (24 BEB sites): 484 486 487 488 490 492 494 495 496 497 499 501 503 504 506 508 510 513 514 516 518 519 522 523 |
| modelAnull | Rat | Branch-site | 3 | 1 | -6122.022857 | N/A | p0=0.83796 p1=0.01354 p2=0.14614 p3=0.00236 w0=0.01292 w1=1.00000 w2=1.00000 | Not Allowed |  |
| **SHDC Site Analysis** | | | | | | | | | |
| m0 | Sites | Homogeneous | 1 | 2 | -3882.638901 | N/A | w=0.19659 | No |  |
| m1Neutral | Sites | Site-specific | 1 | 2 | -3855.248355 | N/A | p0=0.90291 p1=0.09709 w0=0.15887 w1=1.00000 | Not Allowed |  |
| m2Selection | Sites | Site-specific | 2 | 2 | -3855.248355 | m1Neutral | p0=0.90291 p1=0.03957 p2=0.05751 w0=0.15887 w1=1.00000 w2=1.00000 | No |  |
| m3Discrtk2 | Sites | Site-specific | 3 | 2 | -3850.590828 | m3Discrtk2 | p0=0.65104 p1=0.34896 w0=0.09872 w1=0.43787 | No |  |
| m3Discrtk3 | Sites | Site-specific | 5 | 2 | -3844.114024 | m3Discrtk3 | p0=0.38937 p1=0.56570 p2=0.04493 w0=0.05594 w1=0.26805 w2=1.25868 | Yes | Alignment (7 NEB sites): 47 111 114 115 136 154 164 |
| m7 | Sites | Site-specific | 2 | 2 | -3848.26879 | N/A | p=1.06517 q=3.74049 | Not Allowed |  |
| m8 | Sites | Site-specific | 4 | 2 | -3844.788817 | m8a | p=1.49122 p0=0.96361 p1=0.03639 q=6.32777 w=1.34361 | No |  |
| m8a | Sites | Site-specific | 4 | 1 | -3845.178978 | N/A | p=1.57366 p0=0.95032 p1=0.04968 q=7.02265 w=1.00000 | Not Allowed |  |
| **SDHC Branch-site Analysis** | | | | | | | | | |
| modelA | Chimpanzee | Branch-site | 3 | 2 | -3855.248355 | m1Neutral, modelAnull | p0=0.90286 p1=0.09708 p2=0.00006 p3=0.00001 w0=0.15887 w1=1.00000 w2=1.00000 | No |  |
| modelAnull | Chimpanzee | Branch-site | 3 | 1 | -3855.24836 | N/A | p0=0.81071 p1=0.08717 p2=0.09220 p3=0.00991 w0=0.15887 w1=1.00000 w2=1.00000 | Not Allowed |  |
| modelA | Euarchontoglires | Branch-site | 3 | 2 | -3855.026522 | m1Neutral, modelAnull | p0=0.54209 p1=0.05891 p2=0.35989 p3=0.03911 w0=0.15803 w1=1.00000 w2=1.00000 | No |  |
| modelAnull | Euarchontoglires | Branch-site | 3 | 1 | -3855.066042 | N/A | p0=0.11641 p1=0.01264 p2=0.78563 p3=0.08532 w0=0.15769 w1=1.00000 w2=1.00000 | Not Allowed |  |
| modelA | Glires | Branch-site | 3 | 2 | -3855.248355 | m1Neutral, modelAnull | p0=0.90291 p1=0.09709 p2=0.00000 p3=0.00000 w0=0.15887 w1=1.00000 w2=1.00000 | No |  |
| modelAnull | Glires | Branch-site | 3 | 1 | -3855.248355 | N/A | p0=0.90289 p1=0.09708 p2=0.00003 p3=0.00000 w0=0.15887 w1=1.00000 w2=1.00000 | Not Allowed |  |
| modelA | Gorilla | Branch-site | 3 | 0 | -3854.417743 | m1Neutral, modelAnull | p0=0.00000 p1=0.00000 p2=0.90245 p3=0.09755 w0=0.15780 w1=1.00000 w2=999.00000 | No |  |
| modelAnull | Gorilla | Branch-site | 3 | 1 | -3854.54174 | N/A | p0=0.00000 p1=0.00000 p2=0.90231 p3=0.09769 w0=0.15786 w1=1.00000 w2=1.00000 | Not Allowed |  |
| modelA | Guinea_Pig | Branch-site | 3 | 2 | -3854.186622 | m1Neutral, modelAnull | p0=0.75142 p1=0.07934 p2=0.15309 p3=0.01616 w0=0.15427 w1=1.00000 w2=1.00000 | No |  |
| modelAnull | Guinea_Pig | Branch-site | 3 | 1 | -3854.186622 | N/A | p0=0.75142 p1=0.07934 p2=0.15309 p3=0.01616 w0=0.15427 w1=1.00000 w2=1.00000 | Not Allowed |  |
| modelA | Homininae | Branch-site | 3 | 2 | -3855.248355 | m1Neutral, modelAnull | p0=0.90291 p1=0.09709 p2=0.00000 p3=0.00000 w0=0.15887 w1=1.00000 w2=1.00000 | No |  |
| modelAnull | Homininae | Branch-site | 3 | 1 | -3855.248355 | N/A | p0=0.90291 p1=0.09709 p2=0.00000 p3=0.00000 w0=0.15887 w1=1.00000 w2=1.00000 | Not Allowed |  |
| modelA | Hominini | Branch-site | 3 | 2 | -3855.248355 | m1Neutral, modelAnull | p0=0.90291 p1=0.09709 p2=0.00000 p3=0.00000 w0=0.15887 w1=1.00000 w2=1.00000 | No |  |
| modelAnull | Hominini | Branch-site | 3 | 1 | -3855.248355 | N/A | p0=0.90291 p1=0.09709 p2=0.00000 p3=0.00000 w0=0.15887 w1=1.00000 w2=1.00000 | Not Allowed |  |
| modelA | Human | Branch-site | 3 | 10 | -3855.248355 | m1Neutral, modelAnull | p0=0.90291 p1=0.09709 p2=0.00000 p3=0.00000 w0=0.15887 w1=1.00000 w2=7.96523 | No |  |
| modelAnull | Human | Branch-site | 3 | 1 | -3855.248356 | N/A | p0=0.90291 p1=0.09709 p2=0.00000 p3=0.00000 w0=0.15887 w1=1.00000 w2=1.00000 | Not Allowed |  |
| modelA | Marmoset | Branch-site | 3 | 2 | -3854.592689 | m1Neutral, modelAnull | p0=0.90456 p1=0.08822 p2=0.00658 p3=0.00064 w0=0.15939 w1=1.00000 w2=20.86410 | No |  |
| modelAnull | Marmoset | Branch-site | 3 | 1 | -3855.248355 | N/A | p0=0.90291 p1=0.09709 p2=0.00000 p3=0.00000 w0=0.15887 w1=1.00000 w2=1.00000 | Not Allowed |  |
| modelA | Mouse | Branch-site | 3 | 2 | -3854.243281 | m1Neutral, modelAnull | p0=0.85671 p1=0.08461 p2=0.05340 p3=0.00527 w0=0.15561 w1=1.00000 w2=2.94441 | No |  |
| modelAnull | Mouse | Branch-site | 3 | 1 | -3854.55026 | N/A | p0=0.78374 p1=0.07734 p2=0.12644 p3=0.01248 w0=0.15555 w1=1.00000 w2=1.00000 | Not Allowed |  |
| modelA | Muridae | Branch-site | 3 | 2 | -3854.778172 | m1Neutral, modelAnull | p0=0.84059 p1=0.08573 p2=0.06686 p3=0.00682 w0=0.15736 w1=1.00000 w2=5.23447 | No |  |
| modelAnull | Muridae | Branch-site | 3 | 1 | -3854.937109 | N/A | p0=0.67570 p1=0.06933 p2=0.23124 p3=0.02373 w0=0.15751 w1=1.00000 w2=1.00000 | Not Allowed |  |
| modelA | Murinae | Branch-site | 3 | 2 | -3846.690164 | modelA | p0=0.87666 p1=0.08131 p2=0.03846 p3=0.00357 w0=0.15340 w1=1.00000 w2=253.61375 | Yes | Alignment (9 BEB sites): 56 74 101 119 128 144 148 149 150 |
| modelAnull | Murinae | Branch-site | 3 | 1 | -3851.590278 | N/A | p0=0.67557 p1=0.06457 p2=0.23719 p3=0.02267 w0=0.15066 w1=1.00000 w2=1.00000 | Not Allowed |  |
| modelA | Primates | Branch-site | 3 | 2 | -3855.248355 | m1Neutral, modelAnull | p0=0.90291 p1=0.09709 p2=0.00000 p3=0.00000 w0=0.15887 w1=1.00000 w2=1.00000 | No |  |
| modelAnull | Primates | Branch-site | 3 | 1 | -3855.248356 | N/A | p0=0.90291 p1=0.09709 p2=0.00000 p3=0.00000 w0=0.15887 w1=1.00000 w2=1.00000 | Not Allowed |  |
| modelA | Rabbit | Branch-site | 3 | 2 | -3822.683246 | modelA | p0=0.57771 p1=0.06636 p2=0.31926 p3=0.03667 w0=0.12047 w1=1.00000 w2=3.59059 | Yes | Alignment (51 BEB sites): 4 11 15 19 21 23 40 50 55 62 64 65 66 67 72 77 79 86 87 89 93 94 95 108 109 110 120 121 123 124 125 126 128 129 130 131 132 133 134 136 137 138 141 142 146 148 154 155 157 160 164 |
| modelAnull | Rabbit | Branch-site | 3 | 1 | -3826.772477 | N/A | p0=0.33745 p1=0.03920 p2=0.55846 p3=0.06488 w0=0.11835 w1=1.00000 w2=1.00000 | Not Allowed |  |
| modelA | Rat | Branch-site | 3 | 2 | -3854.790181 | m1Neutral, modelAnull | p0=0.89789 p1=0.09262 p2=0.00860 p3=0.00089 w0=0.15763 w1=1.00000 w2=11.17615 | No |  |
| modelAnull | Rat | Branch-site | 3 | 1 | -3855.176153 | N/A | p0=0.87182 p1=0.09185 p2=0.03287 p3=0.00346 w0=0.15780 w1=1.00000 w2=1.00000 | Not Allowed |  |
| **ATM Site Analysis** | | | | | | | | | |
| m0 | Sites | Homogeneous | 1 | 2 | -70732.8608 | N/A | w=0.16215 | No |  |
| m1Neutral | Sites | Site-specific | 1 | 0 | -69588.12378 | N/A | p0=0.81803 p1=0.18197 w0=0.09971 w1=1.00000 | Not Allowed |  |
| m2Selection | Sites | Site-specific | 2 | 2 | -69588.12378 | m1Neutral | p0=0.81803 p1=0.18197 p2=0.00000 w0=0.09971 w1=1.00000 w2=1.00000 | No |  |
| m3Discrtk2 | Sites | Site-specific | 3 | 2 | -69265.32635 | m3Discrtk2 | p0=0.64671 p1=0.35329 w0=0.04983 w1=0.41926 | No |  |
| m3Discrtk3 | Sites | Site-specific | 5 | 0 | -69124.27462 | m3Discrtk3 | p0=0.42468 p1=0.43284 p2=0.14248 w0=0.01987 w1=0.18636 w2=0.68711 | No |  |
| m7 | Sites | Site-specific | 2 | 2 | -69140.52078 | N/A | p=0.48782 q=2.10007 | Not Allowed |  |
| m8 | Sites | Site-specific | 4 | 2 | -69117.70742 | m8a | p=0.58887 p0=0.95455 p1=0.04545 q=3.23735 w=1.00000 | No |  |
| m8a | Sites | Site-specific | 4 | 1 | -69117.70742 | N/A | p=0.58887 p0=0.95455 p1=0.04545 q=3.23741 w=1.00000 | Not Allowed |  |
| **ATM Branch-site Analysis** | | | | | | | | | |
| modelA | Chimpanzee | Branch-site | 3 | 2 | -69586.68022 | m1Neutral, modelAnull | p0=0.68300 p1=0.15207 p2=0.13490 p3=0.03003 w0=0.09949 w1=1.00000 w2=1.00000 | No |  |
| modelAnull | Chimpanzee | Branch-site | 3 | 1 | -69586.68022 | N/A | p0=0.68296 p1=0.15206 p2=0.13494 p3=0.03004 w0=0.09948 w1=1.00000 w2=1.00000 | Not Allowed |  |
| modelA | Euarchontoglires | Branch-site | 3 | 2 | -69585.52179 | m1Neutral | p0=0.81731 p1=0.18195 p2=0.00060 p3=0.00013 w0=0.09956 w1=1.00000 w2=50.35645 | No |  |
| modelAnull | Euarchontoglires | Branch-site | 3 | 1 | -69587.79241 | N/A | p0=0.80423 p1=0.17898 p2=0.01373 p3=0.00306 w0=0.09961 w1=1.00000 w2=1.00000 | Not Allowed |  |
| modelA | Glires | Branch-site | 3 | 10 | -69588.12378 | m1Neutral, modelAnull | p0=0.81803 p1=0.18197 p2=0.00000 p3=0.00000 w0=0.09971 w1=1.00000 w2=7.83732 | No |  |
| modelAnull | Glires | Branch-site | 3 | 1 | -69588.12381 | N/A | p0=0.81803 p1=0.18197 p2=0.00001 p3=0.00000 w0=0.09971 w1=1.00000 w2=1.00000 | Not Allowed |  |
| modelA | Gorilla | Branch-site | 3 | 2 | -69374.08393 | modelA | p0=0.80673 p1=0.17971 p2=0.01109 p3=0.00247 w0=0.09745 w1=1.00000 w2=999.00000 | Yes | Alignment (48 BEB sites): 520 1361 1365 1450 1881 2070 2071 2072 2073 2075 2076 2077 2078 2079 2080 2081 2083 2086 2087 2089 2090 2091 2100 2101 2102 2103 2104 2105 2106 2110 2111 2112 2113 2114 2115 2116 2117 2118 2590 2591 2592 2593 2594 2595 2596 2597 2598 2599 |
| modelAnull | Gorilla | Branch-site | 3 | 1 | -69540.69611 | N/A | p0=0.62007 p1=0.13694 p2=0.19904 p3=0.04396 w0=0.09725 w1=1.00000 w2=1.00000 | Not Allowed |  |
| modelA | Guinea_Pig | Branch-site | 3 | 10 | -69587.37725 | m1Neutral, modelAnull | p0=0.81458 p1=0.18092 p2=0.00368 p3=0.00082 w0=0.09942 w1=1.00000 w2=3.24651 | No |  |
| modelAnull | Guinea_Pig | Branch-site | 3 | 1 | -69587.76829 | N/A | p0=0.80986 p1=0.17998 p2=0.00831 p3=0.00185 w0=0.09941 w1=1.00000 w2=1.00000 | Not Allowed |  |
| modelA | Homindae | Branch-site | 3 | 2 | -69586.07367 | m1Neutral, modelAnull | p0=0.81233 p1=0.17976 p2=0.00648 p3=0.00143 w0=0.09943 w1=1.00000 w2=11.73313 | No |  |
| modelAnull | Homindae | Branch-site | 3 | 1 | -69586.65823 | N/A | p0=0.75792 p1=0.16831 p2=0.06036 p3=0.01340 w0=0.09931 w1=1.00000 w2=1.00000 | Not Allowed |  |
| modelA | Homininae | Branch-site | 3 | 2 | -69588.1238 | m1Neutral, modelAnull | p0=0.81804 p1=0.18196 p2=0.00000 p3=0.00000 w0=0.09971 w1=1.00000 w2=1.00000 | No |  |
| modelAnull | Homininae | Branch-site | 3 | 1 | -69588.12379 | N/A | p0=0.81803 p1=0.18197 p2=0.00000 p3=0.00000 w0=0.09971 w1=1.00000 w2=1.00000 | Not Allowed |  |
| modelA | Hominini | Branch-site | 3 | 1 | -69588.12378 | m1Neutral, modelAnull | p0=0.81804 p1=0.18196 p2=0.00000 p3=0.00000 w0=0.09971 w1=1.00000 w2=1.00000 | No |  |
| modelAnull | Hominini | Branch-site | 3 | 1 | -69588.12381 | N/A | p0=0.81803 p1=0.18197 p2=0.00000 p3=0.00000 w0=0.09971 w1=1.00000 w2=1.00000 | Not Allowed |  |
| modelA | Human | Branch-site | 3 | 1 | -69588.12379 | m1Neutral, modelAnull | p0=0.81803 p1=0.18197 p2=0.00000 p3=0.00000 w0=0.09971 w1=1.00000 w2=1.00000 | No |  |
| modelAnull | Human | Branch-site | 3 | 1 | -69588.12389 | N/A | p0=0.81797 p1=0.18195 p2=0.00007 p3=0.00001 w0=0.09971 w1=1.00000 w2=1.00000 | Not Allowed |  |
| modelA | Marmoset | Branch-site | 3 | 2 | -69583.23068 | modelA | p0=0.81640 p1=0.18148 p2=0.00173 p3=0.00038 w0=0.09939 w1=1.00000 w2=46.82466 | Yes | Alignment (2 BEB sites): 721 1049 |
| modelAnull | Marmoset | Branch-site | 3 | 1 | -69585.18222 | N/A | p0=0.77527 p1=0.17213 p2=0.04304 p3=0.00956 w0=0.09889 w1=1.00000 w2=1.00000 | Not Allowed |  |
| modelA | Mouse | Branch-site | 3 | 10 | -69587.28509 | m1Neutral, modelAnull | p0=0.81708 p1=0.18130 p2=0.00133 p3=0.00029 w0=0.09957 w1=1.00000 w2=9.18704 | No |  |
| modelAnull | Mouse | Branch-site | 3 | 1 | -69588.09724 | N/A | p0=0.81562 p1=0.18134 p2=0.00249 p3=0.00055 w0=0.09965 w1=1.00000 w2=1.00000 | Not Allowed |  |
| modelA | Muridae | Branch-site | 3 | 2 | -69588.07597 | m1Neutral, modelAnull | p0=0.80269 p1=0.17846 p2=0.01542 p3=0.00343 w0=0.09962 w1=1.00000 w2=1.00000 | No |  |
| modelAnull | Muridae | Branch-site | 3 | 1 | -69588.07597 | N/A | p0=0.80270 p1=0.17846 p2=0.01541 p3=0.00343 w0=0.09963 w1=1.00000 w2=1.00000 | Not Allowed |  |
| modelA | Murinae | Branch-site | 3 | 2 | -69585.97707 | m1Neutral, modelAnull | p0=0.79686 p1=0.17712 p2=0.02129 p3=0.00473 w0=0.09862 w1=1.00000 w2=1.00000 | No |  |
| modelAnull | Murinae | Branch-site | 3 | 1 | -69585.97707 | N/A | p0=0.79688 p1=0.17712 p2=0.02127 p3=0.00473 w0=0.09862 w1=1.00000 w2=1.00000 | Not Allowed |  |
| modelA | Orangutan | Branch-site | 3 | 10 | -69588.12378 | m1Neutral, modelAnull | p0=0.81803 p1=0.18197 p2=0.00000 p3=0.00000 w0=0.09971 w1=1.00000 w2=8.10251 | No |  |
| modelAnull | Orangutan | Branch-site | 3 | 1 | -69588.12384 | N/A | p0=0.81803 p1=0.18196 p2=0.00001 p3=0.00000 w0=0.09971 w1=1.00000 w2=1.00000 | Not Allowed |  |
| modelA | Primates | Branch-site | 3 | 0 | -69588.12378 | m1Neutral, modelAnull | p0=0.81803 p1=0.18197 p2=0.00000 p3=0.00000 w0=0.09971 w1=1.00000 w2=1.00000 | No |  |
| modelAnull | Primates | Branch-site | 3 | 1 | -69588.12379 | N/A | p0=0.81803 p1=0.18197 p2=0.00000 p3=0.00000 w0=0.09971 w1=1.00000 w2=1.00000 | Not Allowed |  |
| modelA | Rabbit | Branch-site | 3 | 10 | -69582.95152 | modelA | p0=0.81572 p1=0.18045 p2=0.00313 p3=0.00069 w0=0.09930 w1=1.00000 w2=7.41594 | Yes | Alignment (6 BEB sites): 607 1712 2243 2259 2510 2784 |
| modelAnull | Rabbit | Branch-site | 3 | 1 | -69586.31694 | N/A | p0=0.80643 p1=0.17882 p2=0.01208 p3=0.00268 w0=0.09914 w1=1.00000 w2=1.00000 | Not Allowed |  |
| modelA | Rat | Branch-site | 3 | 10 | -69587.82226 | m1Neutral, modelAnull | p0=0.81781 p1=0.18150 p2=0.00056 p3=0.00013 w0=0.09966 w1=1.00000 w2=15.32107 | No |  |
| modelAnull | Rat | Branch-site | 3 | 1 | -69588.12381 | N/A | p0=0.81803 p1=0.18197 p2=0.00000 p3=0.00000 w0=0.09971 w1=1.00000 w2=1.00000 | Not Allowed |  |
| **BHD Site Analysis** | | | | | | | | | |
| m0 | Sites | Homogeneous | 1 | 2 | -13703.00686 | N/A | w=0.04208 | No |  |
| m1Neutral | Sites | Site-specific | 1 | 2 | -13557.3622 | N/A | p0=0.94092 p1=0.05908 w0=0.03111 w1=1.00000 | Not Allowed |  |
| m2Selection | Sites | Site-specific | 2 | 2 | -13557.3622 | m1Neutral | p0=0.94092 p1=0.00081 p2=0.05828 w0=0.03111 w1=1.00000 w2=1.00000 | No |  |
| m3Discrtk2 | Sites | Site-specific | 3 | 2 | -13380.62896 | m3Discrtk2 | p0=0.70380 p1=0.29620 w0=0.00733 w1=0.14027 | No |  |
| m3Discrtk3 | Sites | Site-specific | 5 | 2 | -13341.98381 | m3Discrtk3 | p0=0.60680 p1=0.32255 p2=0.07065 w0=0.00366 w1=0.07254 w2=0.35293 | No |  |
| m7 | Sites | Site-specific | 2 | 2 | -13347.85066 | N/A | p=0.25572 q=4.24679 | Not Allowed |  |
| m8 | Sites | Site-specific | 4 | 2 | -13343.32569 | m8a | p=0.27697 p0=0.99122 p1=0.00878 q=5.33503 w=1.00000 | No |  |
| m8a | Sites | Site-specific | 4 | 1 | -13343.32569 | N/A | p=0.27696 p0=0.99122 p1=0.00878 q=5.33504 w=1.00000 | Not Allowed |  |
| **BHD Branch-site Analysis** | | | | | | | | | |
| modelA | Chimpanzee | Branch-site | 3 | 2 | -13557.3622 | m1Neutral, modelAnull | p0=0.94092 p1=0.05908 p2=0.00000 p3=0.00000 w0=0.03111 w1=1.00000 w2=1.00000 | No |  |
| modelAnull | Chimpanzee | Branch-site | 3 | 1 | -13557.3622 | N/A | p0=0.94092 p1=0.05908 p2=0.00000 p3=0.00000 w0=0.03111 w1=1.00000 w2=1.00000 | Not Allowed |  |
| modelA | Euarchontoglires | Branch-site | 3 | 10 | -13557.3622 | m1Neutral, modelAnull | p0=0.94092 p1=0.05908 p2=0.00000 p3=0.00000 w0=0.03111 w1=1.00000 w2=1.00000 | No |  |
| modelAnull | Euarchontoglires | Branch-site | 3 | 1 | -13557.3622 | N/A | p0=0.94092 p1=0.05908 p2=0.00000 p3=0.00000 w0=0.03111 w1=1.00000 w2=1.00000 | Not Allowed |  |
| modelA | Glires | Branch-site | 3 | 0 | -13557.35593 | m1Neutral, modelAnull | p0=0.93446 p1=0.05869 p2=0.00645 p3=0.00041 w0=0.03109 w1=1.00000 w2=1.00000 | No |  |
| modelAnull | Glires | Branch-site | 3 | 1 | -13557.35593 | N/A | p0=0.93446 p1=0.05869 p2=0.00645 p3=0.00040 w0=0.03109 w1=1.00000 w2=1.00000 | Not Allowed |  |
| modelA | Gorilla | Branch-site | 3 | 2 | -13557.3622 | m1Neutral, modelAnull | p0=0.94092 p1=0.05908 p2=0.00000 p3=0.00000 w0=0.03111 w1=1.00000 w2=1.00000 | No |  |
| modelAnull | Gorilla | Branch-site | 3 | 1 | -13557.3622 | N/A | p0=0.94092 p1=0.05908 p2=0.00000 p3=0.00000 w0=0.03111 w1=1.00000 w2=1.00000 | Not Allowed |  |
| modelA | Guinea_Pig | Branch-site | 3 | 2 | -13557.3622 | m1Neutral, modelAnull | p0=0.94092 p1=0.05908 p2=0.00000 p3=0.00000 w0=0.03111 w1=1.00000 w2=1.00000 | No |  |
| modelAnull | Guinea_Pig | Branch-site | 3 | 1 | -13557.3622 | N/A | p0=0.94092 p1=0.05908 p2=0.00000 p3=0.00000 w0=0.03111 w1=1.00000 w2=1.00000 | Not Allowed |  |
| modelA | Homindae | Branch-site | 3 | 10 | -13557.3622 | m1Neutral, modelAnull | p0=0.94092 p1=0.05908 p2=0.00000 p3=0.00000 w0=0.03111 w1=1.00000 w2=1.00000 | No |  |
| modelAnull | Homindae | Branch-site | 3 | 1 | -13557.3622 | N/A | p0=0.94092 p1=0.05908 p2=0.00000 p3=0.00000 w0=0.03111 w1=1.00000 w2=1.00000 | Not Allowed |  |
| modelA | Homininae | Branch-site | 3 | 10 | -13557.20479 | m1Neutral, modelAnull | p0=0.93962 p1=0.05904 p2=0.00126 p3=0.00008 w0=0.03105 w1=1.00000 w2=39.96028 | No |  |
| modelAnull | Homininae | Branch-site | 3 | 1 | -13557.21736 | N/A | p0=0.91325 p1=0.05739 p2=0.02763 p3=0.00174 w0=0.03105 w1=1.00000 w2=1.00000 | Not Allowed |  |
| modelA | Hominini | Branch-site | 3 | 2 | -13557.3622 | m1Neutral, modelAnull | p0=0.94092 p1=0.05908 p2=0.00000 p3=0.00000 w0=0.03111 w1=1.00000 w2=1.00000 | No |  |
| modelAnull | Hominini | Branch-site | 3 | 1 | -13557.3622 | N/A | p0=0.94092 p1=0.05908 p2=0.00000 p3=0.00000 w0=0.03111 w1=1.00000 w2=1.00000 | Not Allowed |  |
| modelA | Human | Branch-site | 3 | 2 | -13556.91758 | m1Neutral, modelAnull | p0=0.91874 p1=0.05768 p2=0.02219 p3=0.00139 w0=0.03103 w1=1.00000 w2=3.66394 | No |  |
| modelAnull | Human | Branch-site | 3 | 1 | -13556.97245 | N/A | p0=0.87593 p1=0.05500 p2=0.06499 p3=0.00408 w0=0.03103 w1=1.00000 w2=1.00000 | Not Allowed |  |
| modelA | Marmoset | Branch-site | 3 | 2 | -13557.3622 | m1Neutral, modelAnull | p0=0.94092 p1=0.05908 p2=0.00000 p3=0.00000 w0=0.03111 w1=1.00000 w2=1.00000 | No |  |
| modelAnull | Marmoset | Branch-site | 3 | 1 | -13557.3622 | N/A | p0=0.94092 p1=0.05908 p2=0.00000 p3=0.00000 w0=0.03111 w1=1.00000 w2=1.00000 | Not Allowed |  |
| modelA | Mouse | Branch-site | 3 | 2 | -13557.08445 | m1Neutral, modelAnull | p0=0.92446 p1=0.05814 p2=0.01637 p3=0.00103 w0=0.03086 w1=1.00000 w2=1.00000 | No |  |
| modelAnull | Mouse | Branch-site | 3 | 1 | -13557.08445 | N/A | p0=0.92446 p1=0.05814 p2=0.01637 p3=0.00103 w0=0.03086 w1=1.00000 w2=1.00000 | Not Allowed |  |
| modelA | Muridae | Branch-site | 3 | 2 | -13557.3622 | m1Neutral, modelAnull | p0=0.94092 p1=0.05908 p2=0.00000 p3=0.00000 w0=0.03111 w1=1.00000 w2=1.00000 | No |  |
| modelAnull | Muridae | Branch-site | 3 | 1 | -13557.3622 | N/A | p0=0.94092 p1=0.05908 p2=0.00000 p3=0.00000 w0=0.03111 w1=1.00000 w2=1.00000 | Not Allowed |  |
| modelA | Murinae | Branch-site | 3 | 2 | -13553.09236 | modelAnull | p0=0.93368 p1=0.05550 p2=0.01021 p3=0.00061 w0=0.03060 w1=1.00000 w2=5.11892 | No |  |
| modelAnull | Murinae | Branch-site | 3 | 1 | -13554.80938 | N/A | p0=0.92389 p1=0.05574 p2=0.01921 p3=0.00116 w0=0.03054 w1=1.00000 w2=1.00000 | Not Allowed |  |
| modelA | Orangutan | Branch-site | 3 | 2 | -13557.3622 | m1Neutral, modelAnull | p0=0.94092 p1=0.05908 p2=0.00000 p3=0.00000 w0=0.03111 w1=1.00000 w2=1.00000 | No |  |
| modelAnull | Orangutan | Branch-site | 3 | 1 | -13557.3622 | N/A | p0=0.94092 p1=0.05908 p2=0.00000 p3=0.00000 w0=0.03111 w1=1.00000 w2=1.00000 | Not Allowed |  |
| modelA | Primates | Branch-site | 3 | 2 | -13557.3622 | m1Neutral, modelAnull | p0=0.94092 p1=0.05908 p2=0.00000 p3=0.00000 w0=0.03111 w1=1.00000 w2=1.00000 | No |  |
| modelAnull | Primates | Branch-site | 3 | 1 | -13557.36221 | N/A | p0=0.94092 p1=0.05908 p2=0.00000 p3=0.00000 w0=0.03111 w1=1.00000 w2=1.00000 | Not Allowed |  |
| modelA | Rabbit | Branch-site | 3 | 2 | -13523.51719 | modelA | p0=0.90728 p1=0.05930 p2=0.03137 p3=0.00205 w0=0.02817 w1=1.00000 w2=6.50017 | Yes | Alignment (10 BEB sites): 61 66 68 71 72 74 78 80 81 83 |
| modelAnull | Rabbit | Branch-site | 3 | 1 | -13527.88414 | N/A | p0=0.88287 p1=0.05758 p2=0.05590 p3=0.00365 w0=0.02741 w1=1.00000 w2=1.00000 | Not Allowed |  |
| modelA | Rat | Branch-site | 3 | 2 | -13557.3622 | m1Neutral, modelAnull | p0=0.94092 p1=0.05908 p2=0.00000 p3=0.00000 w0=0.03111 w1=1.00000 w2=1.00000 | No |  |
| modelAnull | Rat | Branch-site | 3 | 1 | -13557.36222 | N/A | p0=0.94092 p1=0.05908 p2=0.00000 p3=0.00000 w0=0.03111 w1=1.00000 w2=1.00000 | Not Allowed |  |
| **TSC1 Site Analysis** | | | | | | | | | |
| m0 | Sites | Homogeneous | 1 | 2 | -24547.02149 | N/A | w=0.15658 | No |  |
| m1Neutral | Sites | Site-specific | 1 | 2 | -24131.46105 | N/A | p0=0.81292 p1=0.18708 w0=0.08319 w1=1.00000 | Not Allowed |  |
| m2Selection | Sites | Site-specific | 2 | 2 | -24131.46105 | m1Neutral | p0=0.81291 p1=0.11647 p2=0.07062 w0=0.08319 w1=1.00000 w2=1.00000 | No |  |
| m3Discrtk2 | Sites | Site-specific | 3 | 2 | -24050.12864 | m3Discrtk2 | p0=0.68301 p1=0.31699 w0=0.04904 w1=0.47204 | No |  |
| m3Discrtk3 | Sites | Site-specific | 5 | 1 | -24010.86719 | m3Discrtk3 | p0=0.38391 p1=0.44545 p2=0.17064 w0=0.01393 w1=0.14810 w2=0.68313 | No |  |
| m7 | Sites | Site-specific | 2 | 10 | -24020.75491 | N/A | p=0.43804 q=1.88760 | Not Allowed |  |
| m8 | Sites | Site-specific | 4 | 2 | -24011.27194 | m8a | p=0.55890 p0=0.93619 p1=0.06381 q=3.40415 w=1.00000 | No |  |
| m8a | Sites | Site-specific | 4 | 1 | -24011.27194 | N/A | p=0.55890 p0=0.93619 p1=0.06381 q=3.40403 w=1.00000 | Not Allowed |  |
| **TSC1 Branch-site Analysis** | | | | | | | | | |
| modelA | Chimpanzee | Branch-site | 3 | 0 | -24131.46106 | m1Neutral, modelAnull | p0=0.81291 p1=0.18709 p2=0.00000 p3=0.00000 w0=0.08319 w1=1.00000 w2=1.00000 | No |  |
| modelAnull | Chimpanzee | Branch-site | 3 | 1 | -24131.46108 | N/A | p0=0.81291 p1=0.18708 p2=0.00001 p3=0.00000 w0=0.08319 w1=1.00000 w2=1.00000 | Not Allowed |  |
| modelA | Euarchontoglires | Branch-site | 3 | 2 | -24130.00092 | m1Neutral, modelAnull | p0=0.61814 p1=0.14184 p2=0.19523 p3=0.04480 w0=0.08267 w1=1.00000 w2=1.00000 | No |  |
| modelAnull | Euarchontoglires | Branch-site | 3 | 1 | -24130.00092 | N/A | p0=0.61807 p1=0.14182 p2=0.19529 p3=0.04481 w0=0.08267 w1=1.00000 w2=1.00000 | Not Allowed |  |
| modelA | Glires | Branch-site | 3 | 2 | -24131.46106 | m1Neutral, modelAnull | p0=0.81292 p1=0.18708 p2=0.00000 p3=0.00000 w0=0.08319 w1=1.00000 w2=1.00000 | No |  |
| modelAnull | Glires | Branch-site | 3 | 1 | -24131.46108 | N/A | p0=0.81292 p1=0.18708 p2=0.00000 p3=0.00000 w0=0.08319 w1=1.00000 w2=1.00000 | Not Allowed |  |
| modelA | Gorilla | Branch-site | 3 | 0 | -24131.45561 | m1Neutral, modelAnull | p0=0.80304 p1=0.18477 p2=0.00992 p3=0.00228 w0=0.08317 w1=1.00000 w2=1.00000 | No |  |
| modelAnull | Gorilla | Branch-site | 3 | 1 | -24131.45561 | N/A | p0=0.80309 p1=0.18478 p2=0.00987 p3=0.00227 w0=0.08317 w1=1.00000 w2=1.00000 | Not Allowed |  |
| modelA | Guinea_Pig | Branch-site | 3 | 10 | -24116.58577 | modelA | p0=0.80206 p1=0.18611 p2=0.00961 p3=0.00223 w0=0.08093 w1=1.00000 w2=284.22603 | Yes | Alignment (9 BEB sites): 4 382 389 392 393 394 395 396 397 |
| modelAnull | Guinea_Pig | Branch-site | 3 | 1 | -24128.46528 | N/A | p0=0.79073 p1=0.18153 p2=0.02256 p3=0.00518 w0=0.08106 w1=1.00000 w2=1.00000 | Not Allowed |  |
| modelA | Homindae | Branch-site | 3 | 2 | -24131.46106 | m1Neutral, modelAnull | p0=0.81291 p1=0.18709 p2=0.00000 p3=0.00000 w0=0.08319 w1=1.00000 w2=1.00000 | No |  |
| modelAnull | Homindae | Branch-site | 3 | 1 | -24131.46106 | N/A | p0=0.81292 p1=0.18708 p2=0.00000 p3=0.00000 w0=0.08319 w1=1.00000 w2=1.00000 | Not Allowed |  |
| modelA | Homininae | Branch-site | 3 | 2 | -24131.46105 | m1Neutral, modelAnull | p0=0.81292 p1=0.18708 p2=0.00000 p3=0.00000 w0=0.08318 w1=1.00000 w2=1.00000 | No |  |
| modelAnull | Homininae | Branch-site | 3 | 1 | -24131.46105 | N/A | p0=0.81292 p1=0.18708 p2=0.00000 p3=0.00000 w0=0.08319 w1=1.00000 w2=1.00000 | Not Allowed |  |
| modelA | Hominini | Branch-site | 3 | 0 | -24130.94781 | m1Neutral, modelAnull | p0=0.49134 p1=0.11302 p2=0.32165 p3=0.07399 w0=0.08307 w1=1.00000 w2=1.00000 | No |  |
| modelAnull | Hominini | Branch-site | 3 | 1 | -24130.94781 | N/A | p0=0.49136 p1=0.11302 p2=0.32163 p3=0.07398 w0=0.08307 w1=1.00000 w2=1.00000 | Not Allowed |  |
| modelA | Human | Branch-site | 3 | 10 | -24131.46105 | m1Neutral, modelAnull | p0=0.81292 p1=0.18708 p2=0.00000 p3=0.00000 w0=0.08319 w1=1.00000 w2=8.37599 | No |  |
| modelAnull | Human | Branch-site | 3 | 1 | -24131.46106 | N/A | p0=0.81292 p1=0.18708 p2=0.00000 p3=0.00000 w0=0.08319 w1=1.00000 w2=1.00000 | Not Allowed |  |
| modelA | Marmoset | Branch-site | 3 | 0 | -24131.07392 | m1Neutral, modelAnull | p0=0.77688 p1=0.17869 p2=0.03612 p3=0.00831 w0=0.08275 w1=1.00000 w2=1.00000 | No |  |
| modelAnull | Marmoset | Branch-site | 3 | 1 | -24131.07392 | N/A | p0=0.77686 p1=0.17869 p2=0.03614 p3=0.00831 w0=0.08275 w1=1.00000 w2=1.00000 | Not Allowed |  |
| modelA | Mouse | Branch-site | 3 | 1 | -24131.46106 | m1Neutral, modelAnull | p0=0.81292 p1=0.18708 p2=0.00000 p3=0.00000 w0=0.08319 w1=1.00000 w2=1.00000 | No |  |
| modelAnull | Mouse | Branch-site | 3 | 1 | -24131.46122 | N/A | p0=0.81291 p1=0.18708 p2=0.00000 p3=0.00000 w0=0.08319 w1=1.00000 w2=1.00000 | Not Allowed |  |
| modelA | Muridae | Branch-site | 3 | 10 | -24126.17894 | modelA | p0=0.80995 p1=0.18416 p2=0.00481 p3=0.00109 w0=0.08293 w1=1.00000 w2=999.00000 | Yes | Alignment (1 BEB sites): 403 |
| modelAnull | Muridae | Branch-site | 3 | 1 | -24131.46106 | N/A | p0=0.81292 p1=0.18708 p2=0.00000 p3=0.00000 w0=0.08319 w1=1.00000 w2=1.00000 | Not Allowed |  |
| modelA | Murinae | Branch-site | 3 | 10 | -24127.69107 | modelAnull | p0=0.80279 p1=0.18235 p2=0.01210 p3=0.00275 w0=0.08176 w1=1.00000 w2=4.87160 | No |  |
| modelAnull | Murinae | Branch-site | 3 | 1 | -24128.68326 | N/A | p0=0.77086 p1=0.17603 p2=0.04324 p3=0.00987 w0=0.08118 w1=1.00000 w2=1.00000 | Not Allowed |  |
| modelA | Orangutan | Branch-site | 3 | 2 | -24068.71106 | modelA | p0=0.79963 p1=0.18828 p2=0.00978 p3=0.00230 w0=0.08020 w1=1.00000 w2=999.00000 | Yes | Alignment (13 BEB sites): 257 258 259 260 261 262 264 265 405 421 422 443 620 |
| modelAnull | Orangutan | Branch-site | 3 | 1 | -24112.24213 | N/A | p0=0.60051 p1=0.14098 p2=0.20936 p3=0.04915 w0=0.07981 w1=1.00000 w2=1.00000 | Not Allowed |  |
| modelA | Primates | Branch-site | 3 | 10 | -24131.46106 | m1Neutral, modelAnull | p0=0.81292 p1=0.18708 p2=0.00000 p3=0.00000 w0=0.08319 w1=1.00000 w2=10.40568 | No |  |
| modelAnull | Primates | Branch-site | 3 | 1 | -24131.46106 | N/A | p0=0.81291 p1=0.18709 p2=0.00000 p3=0.00000 w0=0.08319 w1=1.00000 w2=1.00000 | Not Allowed |  |
| modelA | Rabbit | Branch-site | 3 | 2 | -24131.01503 | m1Neutral, modelAnull | p0=0.81124 p1=0.18676 p2=0.00162 p3=0.00037 w0=0.08288 w1=1.00000 w2=6.34513 | No |  |
| modelAnull | Rabbit | Branch-site | 3 | 1 | -24131.36932 | N/A | p0=0.80842 p1=0.18595 p2=0.00458 p3=0.00105 w0=0.08299 w1=1.00000 w2=1.00000 | Not Allowed |  |
| modelA | Rat | Branch-site | 3 | 2 | -24131.1374 | m1Neutral, modelAnull | p0=0.78589 p1=0.18081 p2=0.02707 p3=0.00623 w0=0.08265 w1=1.00000 w2=1.00000 | No |  |
| modelAnull | Rat | Branch-site | 3 | 1 | -24131.1374 | N/A | p0=0.78588 p1=0.18081 p2=0.02708 p3=0.00623 w0=0.08265 w1=1.00000 w2=1.00000 | Not Allowed |  |
| **PTEN Site Analysis** | | | | | | | | | |
| m0 | Sites | Homogeneous | 1 | 2 | -4524.059756 | N/A | w=0.02865 | No |  |
| MLH1 | Sites | Site-specific | 1 | 2 | -4497.578401 | N/A | p0=0.96860 p1=0.03140 w0=0.02047 w1=1.00000 | Not Allowed |  |
| m2Selection | Sites | Site-specific | 2 | 2 | -4497.578401 | m1Neutral | p0=0.96860 p1=0.01651 p2=0.01488 w0=0.02046 w1=1.00000 w2=1.00000 | No |  |
| m3Discrtk2 | Sites | Site-specific | 3 | 2 | -4473.416129 | m3Discrtk2 | p0=0.86074 p1=0.13926 w0=0.00702 w1=0.19892 | No |  |
| m3Discrtk3 | Sites | Site-specific | 5 | 0 | -4473.096704 | m3Discrtk2 | p0=0.82511 p1=0.15619 p2=0.01870 w0=0.00533 w1=0.13788 w2=0.44984 | No |  |
| m7 | Sites | Site-specific | 2 | 2 | -4473.452897 | N/A | p=0.14486 q=3.61688 | Not Allowed |  |
| m8 | Sites | Site-specific | 4 | 2 | -4473.441809 | m7, m8a | p=0.14746 p0=0.99885 p1=0.00115 q=3.77215 w=1.00000 | No |  |
| m8a | Sites | Site-specific | 4 | 1 | -4473.441809 | N/A | p=0.14746 p0=0.99885 p1=0.00115 q=3.77219 w=1.00000 | Not Allowed |  |
| **PTEN Branch-site Analysis** | | | | | | | | | |
| modelA | Chimpanzee | Branch-site | 3 | 2 | -4497.578401 | m1Neutral, modelAnull | p0=0.96860 p1=0.03140 p2=0.00000 p3=0.00000 w0=0.02046 w1=1.00000 w2=1.00000 | No |  |
| modelAnull | Chimpanzee | Branch-site | 3 | 1 | -4497.578401 | N/A | p0=0.96860 p1=0.03140 p2=0.00000 p3=0.00000 w0=0.02046 w1=1.00000 w2=1.00000 | Not Allowed |  |
| modelA | Euarchontoglires | Branch-site | 3 | 2 | -4497.578401 | m1Neutral, modelAnull | p0=0.96860 p1=0.03140 p2=0.00000 p3=0.00000 w0=0.02046 w1=1.00000 w2=1.00000 | No |  |
| modelAnull | Euarchontoglires | Branch-site | 3 | 1 | -4497.578401 | N/A | p0=0.96860 p1=0.03140 p2=0.00000 p3=0.00000 w0=0.02046 w1=1.00000 w2=1.00000 | Not Allowed |  |
| modelA | Glires | Branch-site | 3 | 0 | -4497.5784 | m1Neutral, modelAnull | p0=0.96860 p1=0.03140 p2=0.00000 p3=0.00000 w0=0.02046 w1=1.00000 w2=1.00000 | No |  |
| modelAnull | Glires | Branch-site | 3 | 1 | -4497.578401 | N/A | p0=0.96860 p1=0.03140 p2=0.00000 p3=0.00000 w0=0.02046 w1=1.00000 w2=1.00000 | Not Allowed |  |
| modelA | Gorilla | Branch-site | 3 | 2 | -4497.578401 | m1Neutral, modelAnull | p0=0.96860 p1=0.03140 p2=0.00000 p3=0.00000 w0=0.02046 w1=1.00000 w2=1.00000 | No |  |
| modelAnull | Gorilla | Branch-site | 3 | 1 | -4497.578401 | N/A | p0=0.96860 p1=0.03140 p2=0.00000 p3=0.00000 w0=0.02047 w1=1.00000 w2=1.00000 | Not Allowed |  |
| modelA | Homindae | Branch-site | 3 | 0 | -4497.578403 | m1Neutral, modelAnull | p0=0.96367 p1=0.03124 p2=0.00494 p3=0.00016 w0=0.02046 w1=1.00000 w2=1.00000 | No |  |
| modelAnull | Homindae | Branch-site | 3 | 1 | -4497.578433 | N/A | p0=0.88906 p1=0.02882 p2=0.07954 p3=0.00258 w0=0.02046 w1=1.00000 w2=1.00000 | Not Allowed |  |
| modelA | Homininae | Branch-site | 3 | 10 | -4497.5784 | m1Neutral, modelAnull | p0=0.96860 p1=0.03140 p2=0.00000 p3=0.00000 w0=0.02046 w1=1.00000 w2=7.37833 | No |  |
| modelAnull | Homininae | Branch-site | 3 | 1 | -4497.578401 | N/A | p0=0.96860 p1=0.03140 p2=0.00000 p3=0.00000 w0=0.02046 w1=1.00000 w2=1.00000 | Not Allowed |  |
| modelA | Hominini | Branch-site | 3 | 0 | -4497.5784 | m1Neutral, modelAnull | p0=0.96859 p1=0.03140 p2=0.00001 p3=0.00000 w0=0.02046 w1=1.00000 w2=1.00000 | No |  |
| modelAnull | Hominini | Branch-site | 3 | 1 | -4497.578426 | N/A | p0=0.88451 p1=0.02867 p2=0.08409 p3=0.00273 w0=0.02046 w1=1.00000 w2=1.00000 | Not Allowed |  |
| modelA | Human | Branch-site | 3 | 2 | -4497.578401 | m1Neutral, modelAnull | p0=0.96860 p1=0.03140 p2=0.00000 p3=0.00000 w0=0.02046 w1=1.00000 w2=1.00000 | No |  |
| modelAnull | Human | Branch-site | 3 | 1 | -4497.578401 | N/A | p0=0.96860 p1=0.03140 p2=0.00000 p3=0.00000 w0=0.02046 w1=1.00000 w2=1.00000 | Not Allowed |  |
| modelA | Marmoset | Branch-site | 3 | 0 | -4497.5784 | m1Neutral, modelAnull | p0=0.96860 p1=0.03140 p2=0.00000 p3=0.00000 w0=0.02046 w1=1.00000 w2=1.00000 | No |  |
| modelAnull | Marmoset | Branch-site | 3 | 1 | -4497.578401 | N/A | p0=0.96860 p1=0.03140 p2=0.00000 p3=0.00000 w0=0.02046 w1=1.00000 w2=1.00000 | Not Allowed |  |
| modelA | Mouse | Branch-site | 3 | 10 | -4497.5784 | m1Neutral, modelAnull | p0=0.96860 p1=0.03140 p2=0.00000 p3=0.00000 w0=0.02046 w1=1.00000 w2=9.17702 | No |  |
| modelAnull | Mouse | Branch-site | 3 | 1 | -4497.5784 | N/A | p0=0.96860 p1=0.03140 p2=0.00000 p3=0.00000 w0=0.02046 w1=1.00000 w2=1.00000 | Not Allowed |  |
| modelA | Murinae | Branch-site | 3 | 10 | -4497.5784 | m1Neutral, modelAnull | p0=0.96860 p1=0.03140 p2=0.00000 p3=0.00000 w0=0.02046 w1=1.00000 w2=9.60972 | No |  |
| modelAnull | Murinae | Branch-site | 3 | 1 | -4497.578401 | N/A | p0=0.96860 p1=0.03140 p2=0.00000 p3=0.00000 w0=0.02046 w1=1.00000 w2=1.00000 | Not Allowed |  |
| modelA | Orangutan | Branch-site | 3 | 2 | -4496.35394 | m1Neutral, modelAnull | p0=0.76108 p1=0.02496 p2=0.20717 p3=0.00679 w0=0.02006 w1=1.00000 w2=1.00000 | No |  |
| modelAnull | Orangutan | Branch-site | 3 | 1 | -4496.35394 | N/A | p0=0.76108 p1=0.02496 p2=0.20717 p3=0.00679 w0=0.02006 w1=1.00000 w2=1.00000 | Not Allowed |  |
| modelA | Primates | Branch-site | 3 | 2 | -4497.578401 | m1Neutral, modelAnull | p0=0.96860 p1=0.03140 p2=0.00000 p3=0.00000 w0=0.02046 w1=1.00000 w2=1.00000 | No |  |
| modelAnull | Primates | Branch-site | 3 | 1 | -4497.578401 | N/A | p0=0.96860 p1=0.03140 p2=0.00000 p3=0.00000 w0=0.02046 w1=1.00000 w2=1.00000 | Not Allowed |  |
| modelA | Rabbit | Branch-site | 3 | 0 | -4497.5784 | m1Neutral, modelAnull | p0=0.96860 p1=0.03140 p2=0.00000 p3=0.00000 w0=0.02046 w1=1.00000 w2=1.00000 | No |  |
| modelAnull | Rabbit | Branch-site | 3 | 1 | -4497.5784 | N/A | p0=0.96860 p1=0.03140 p2=0.00000 p3=0.00000 w0=0.02046 w1=1.00000 w2=1.00000 | Not Allowed |  |
| modelA | Rat | Branch-site | 3 | 1 | -4497.5784 | m1Neutral, modelAnull | p0=0.96860 p1=0.03140 p2=0.00000 p3=0.00000 w0=0.02046 w1=1.00000 w2=1.00000 | No |  |
| modelAnull | Rat | Branch-site | 3 | 1 | -4497.5784 | N/A | p0=0.96860 p1=0.03140 p2=0.00000 p3=0.00000 w0=0.02046 w1=1.00000 w2=1.00000 | Not Allowed |  |
| **NF1 Site Analysis** | | | | | | | | | |
| m0 | Sites | Homogeneous | 1 | 2 | -38005.96444 | N/A | w=0.03205 | No |  |
| m1Neutral | Sites | Site-specific | 1 | 2 | -37856.05303 | N/A | p0=0.97500 p1=0.02500 w0=0.02434 w1=1.00000 | Not Allowed |  |
| m2Selection | Sites | Site-specific | 2 | 2 | -37856.05303 | m1Neutral | p0=0.97500 p1=0.02500 p2=0.00000 w0=0.02434 w1=1.00000 w2=25.92690 | No |  |
| m3Discrtk2 | Sites | Site-specific | 3 | 2 | -37672.49234 | m3Discrtk2 | p0=0.84376 p1=0.15624 w0=0.00764 w1=0.18293 | No |  |
| m3Discrtk3 | Sites | Site-specific | 5 | 0 | -37665.26209 | m3Discrtk3 | p0=0.77183 p1=0.21123 p2=0.01695 w0=0.00419 w1=0.11493 w2=0.47670 | No |  |
| m7 | Sites | Site-specific | 2 | 2 | -37669.39768 | N/A | p=0.17285 q=4.18991 | Not Allowed |  |
| m8 | Sites | Site-specific | 4 | 2 | -37666.58318 | m7, m8a | p=0.18442 p0=0.99676 p1=0.00324 q=4.83685 w=1.00000 | No |  |
| m8a | Sites | Site-specific | 4 | 1 | -37666.58318 | N/A | p=0.18442 p0=0.99676 p1=0.00324 q=4.83687 w=1.00000 | Not Allowed |  |
| **NF1 Branch-site Analysis** | | | | | | | | | |
| modelA | Chimpanzee | Branch-site | 3 | 1 | -37856.05303 | m1Neutral, modelAnull | p0=0.97500 p1=0.02500 p2=0.00000 p3=0.00000 w0=0.02434 w1=1.00000 w2=1.00000 | No |  |
| modelAnull | Chimpanzee | Branch-site | 3 | 1 | -37856.05303 | N/A | p0=0.97500 p1=0.02500 p2=0.00000 p3=0.00000 w0=0.02434 w1=1.00000 w2=1.00000 | Not Allowed |  |
| modelA | Euarchontoglires | Branch-site | 3 | 10 | -37856.05303 | m1Neutral, modelAnull | p0=0.97500 p1=0.02500 p2=0.00000 p3=0.00000 w0=0.02434 w1=1.00000 w2=9.39982 | No |  |
| modelAnull | Euarchontoglires | Branch-site | 3 | 1 | -37856.05305 | N/A | p0=0.97500 p1=0.02500 p2=0.00000 p3=0.00000 w0=0.02434 w1=1.00000 w2=1.00000 | Not Allowed |  |
| modelA | Glires | Branch-site | 3 | 10 | -37856.05303 | m1Neutral, modelAnull | p0=0.97500 p1=0.02500 p2=0.00000 p3=0.00000 w0=0.02434 w1=1.00000 w2=1.00000 | No |  |
| modelAnull | Glires | Branch-site | 3 | 1 | -37856.05305 | N/A | p0=0.97500 p1=0.02500 p2=0.00000 p3=0.00000 w0=0.02434 w1=1.00000 w2=1.00000 | Not Allowed |  |
| modelA | Gorilla | Branch-site | 3 | 2 | -37842.19272 | modelAnull | p0=0.75640 p1=0.01956 p2=0.21838 p3=0.00565 w0=0.02372 w1=1.00000 w2=1.00000 | No |  |
| modelAnull | Gorilla | Branch-site | 3 | 1 | -37842.19272 | N/A | p0=0.75640 p1=0.01956 p2=0.21838 p3=0.00565 w0=0.02372 w1=1.00000 w2=1.00000 | Not Allowed |  |
| modelA | Guinea_Pig | Branch-site | 3 | 10 | -37849.50819 | modelA | p0=0.97375 p1=0.02506 p2=0.00116 p3=0.00003 w0=0.02414 w1=1.00000 w2=171.64068 | Yes | Alignment (3 BEB sites): 2628 2629 2630 |
| modelAnull | Guinea_Pig | Branch-site | 3 | 1 | -37853.783 | N/A | p0=0.96969 p1=0.02493 p2=0.00524 p3=0.00013 w0=0.02408 w1=1.00000 w2=1.00000 | Not Allowed |  |
| modelA | Homindae | Branch-site | 3 | 10 | -37856.05303 | m1Neutral, modelAnull | p0=0.97500 p1=0.02500 p2=0.00000 p3=0.00000 w0=0.02434 w1=1.00000 w2=13.77373 | No |  |
| modelAnull | Homindae | Branch-site | 3 | 1 | -37856.05306 | N/A | p0=0.97500 p1=0.02500 p2=0.00000 p3=0.00000 w0=0.02434 w1=1.00000 w2=1.00000 | Not Allowed |  |
| modelA | Homininae | Branch-site | 3 | 2 | -37856.05303 | m1Neutral, modelAnull | p0=0.97500 p1=0.02500 p2=0.00000 p3=0.00000 w0=0.02434 w1=1.00000 w2=1.00000 | No |  |
| modelAnull | Homininae | Branch-site | 3 | 1 | -37856.05303 | N/A | p0=0.97500 p1=0.02500 p2=0.00000 p3=0.00000 w0=0.02434 w1=1.00000 w2=1.00000 | Not Allowed |  |
| modelA | Hominini | Branch-site | 3 | 10 | -37856.05303 | m1Neutral, modelAnull | p0=0.97500 p1=0.02500 p2=0.00000 p3=0.00000 w0=0.02434 w1=1.00000 w2=9.36176 | No |  |
| modelAnull | Hominini | Branch-site | 3 | 1 | -37856.05308 | N/A | p0=0.97500 p1=0.02500 p2=0.00001 p3=0.00000 w0=0.02434 w1=1.00000 w2=1.00000 | Not Allowed |  |
| modelA | Human | Branch-site | 3 | 10 | -37856.05303 | m1Neutral, modelAnull | p0=0.97500 p1=0.02500 p2=0.00000 p3=0.00000 w0=0.02434 w1=1.00000 w2=6.45060 | No |  |
| modelAnull | Human | Branch-site | 3 | 1 | -37856.05304 | N/A | p0=0.97500 p1=0.02500 p2=0.00000 p3=0.00000 w0=0.02434 w1=1.00000 w2=1.00000 | Not Allowed |  |
| modelA | Marmoset | Branch-site | 3 | 1 | -37856.05304 | m1Neutral, modelAnull | p0=0.97500 p1=0.02500 p2=0.00000 p3=0.00000 w0=0.02434 w1=1.00000 w2=1.00000 | No |  |
| modelAnull | Marmoset | Branch-site | 3 | 1 | -37856.05303 | N/A | p0=0.97500 p1=0.02500 p2=0.00000 p3=0.00000 w0=0.02434 w1=1.00000 w2=1.00000 | Not Allowed |  |
| modelA | Mouse | Branch-site | 3 | 10 | -37856.05304 | m1Neutral, modelAnull | p0=0.97500 p1=0.02500 p2=0.00000 p3=0.00000 w0=0.02434 w1=1.00000 w2=3.65477 | No |  |
| modelAnull | Mouse | Branch-site | 3 | 1 | -37856.05308 | N/A | p0=0.97499 p1=0.02500 p2=0.00001 p3=0.00000 w0=0.02434 w1=1.00000 w2=1.00000 | Not Allowed |  |
| modelA | Muridae | Branch-site | 3 | 2 | -37856.05305 | m1Neutral, modelAnull | p0=0.97500 p1=0.02500 p2=0.00000 p3=0.00000 w0=0.02434 w1=1.00000 w2=1.00000 | No |  |
| modelAnull | Muridae | Branch-site | 3 | 1 | -37856.05303 | N/A | p0=0.97500 p1=0.02500 p2=0.00000 p3=0.00000 w0=0.02434 w1=1.00000 w2=1.00000 | Not Allowed |  |
| modelA | Murinae | Branch-site | 3 | 1 | -37853.80006 | m1Neutral | p0=0.97477 p1=0.02473 p2=0.00049 p3=0.00001 w0=0.02430 w1=1.00000 w2=311.64013 | No |  |
| modelAnull | Murinae | Branch-site | 3 | 1 | -37856.01407 | N/A | p0=0.97454 p1=0.02492 p2=0.00053 p3=0.00001 w0=0.02432 w1=1.00000 w2=1.00000 | Not Allowed |  |
| modelA | Orangutan | Branch-site | 3 | 1 | -37855.96105 | m1Neutral, modelAnull | p0=0.96515 p1=0.02477 p2=0.00983 p3=0.00025 w0=0.02430 w1=1.00000 w2=1.00000 | No |  |
| modelAnull | Orangutan | Branch-site | 3 | 1 | -37855.96105 | N/A | p0=0.96515 p1=0.02477 p2=0.00983 p3=0.00025 w0=0.02430 w1=1.00000 w2=1.00000 | Not Allowed |  |
| modelA | Primates | Branch-site | 3 | 0 | -37856.05303 | m1Neutral, modelAnull | p0=0.97500 p1=0.02500 p2=0.00000 p3=0.00000 w0=0.02434 w1=1.00000 w2=1.00000 | No |  |
| modelAnull | Primates | Branch-site | 3 | 1 | -37856.05307 | N/A | p0=0.97500 p1=0.02500 p2=0.00000 p3=0.00000 w0=0.02434 w1=1.00000 w2=1.00000 | Not Allowed |  |
| modelA | Rabbit | Branch-site | 3 | 10 | -37856.05303 | m1Neutral, modelAnull | p0=0.97500 p1=0.02500 p2=0.00000 p3=0.00000 w0=0.02434 w1=1.00000 w2=43.78496 | No |  |
| modelAnull | Rabbit | Branch-site | 3 | 1 | -37856.05309 | N/A | p0=0.97500 p1=0.02500 p2=0.00000 p3=0.00000 w0=0.02434 w1=1.00000 w2=1.00000 | Not Allowed |  |
| modelA | Rat | Branch-site | 3 | 10 | -37750.29866 | modelA | p0=0.96609 p1=0.02476 p2=0.00892 p3=0.00023 w0=0.02265 w1=1.00000 w2=999.00000 | Yes | Alignment (39 BEB sites): 286 294 460 486 640 704 750 941 942 2443 2444 2445 2446 2448 2449 2450 2451 2452 2453 2454 2459 2462 2463 2465 2466 2467 2468 2470 2471 2472 2473 2474 2475 2478 2479 2481 2484 2486 2500 |
| modelAnull | Rat | Branch-site | 3 | 1 | -37803.90769 | N/A | p0=0.92257 p1=0.02326 p2=0.05284 p3=0.00133 w0=0.02234 w1=1.00000 w2=1.00000 | Not Allowed |  |
|  |  |  |  |  |  |  |  |  |  |
