# Supplementary material for: Colon cancer associated genes exhibit signatures of positive selection at functionally significant positions
Source: BMC Evol Biol. 2012 Jul 12;12:114. doi: 10.1186/1471-2148-12-114 (PMC3563467; doi:10.1186/1471-2148-12-114)
Supplement: Additional file 5 — Full set of recombination test results on a per gene and per species basis. The value highlighted in yellow for TP53 represents a region where recombination was detected with reasonable confidence that also coincided with a positively selected residue (i.e. false positive). [file 1471-2148-12-114-S5.doc]

| **Seq Name** | **Sim Pvalue** | **BC KA Value** | **Alignment start (nuc)** | **Alignment End (nuc)** | **Len** | **Positively Selected Sites Removed (AA positions)** |
| --- | --- | --- | --- | --- | --- | --- |
|  |  |  |  |  |  |  |
| **CDH1** |  |  |  |  |  |  |
| NA | NA | NA | NA | NA | NA | NA |
| **PMS1** |  |  |  |  |  |  |
| NA | NA | NA | NA | NA | NA | NA |
| **MLH1** |  |  |  |  |  |  |
| Chicken;Fugu | 0.0037 | 0.00967 | 1713 | 1739 | 27 | None |
| Horse;Platypus | 0.0051 | 0.01317 | 952 | 985 | 34 | None |
| Dog;Platypus | 0.0093 | 0.02324 | 952 | 985 | 34 | None |
| Orangutan;Platypus | 0.015 | 0.03912 | 958 | 985 | 28 | None |
| Human;Platypus | 0.0189 | 0.04617 | 958 | 985 | 28 | None |
| Gorilla;Platypus | 0.0198 | 0.04772 | 958 | 985 | 28 | None |
| Chimpanzee;Platypus | 0.0209 | 0.04931 | 958 | 985 | 28 | None |
| **MSH2** |  |  |  |  |  |  |
| Zebra_Finch;Frog | 0.034 | 0.10025 | 1435 | 1463 | 29 | None |
| **TSC2** |  |  |  |  |  |  |
| Orangutan;Zebrafish | 0.0392 | 0.08831 | 1579 | 1604 | 26 | None |
| **MET** |  |  |  |  |  |  |
| Rabbit;Opossum | 0.001 | 0.00217 | 2530 | 2606 | 77 | None |
| Human;Opossum | 0.0054 | 0.01115 | 2530 | 2606 | 77 | None |
| Orangutan;Opossum | 0.0054 | 0.01115 | 2530 | 2606 | 77 | None |
| Chimpanzee;Opossum | 0.0054 | 0.01115 | 2530 | 2606 | 77 | None |
| Marmoset;Dog | 0.0485 | 0.11156 | 2875 | 3032 | 158 | None |
| **BMPR1A** |  |  |  |  |  |  |
| Cow;Dog | 0 | 0.00015 | 205 | 362 | 158 | None |
| Rabbit;Elephant | 0.0027 | 0.00958 | 226 | 284 | 59 | None |
| Orangutan;Rabbit | 0.0141 | 0.0455 | 217 | 285 | 69 | None |
| Chimpanzee;Rabbit | 0.0141 | 0.0455 | 217 | 285 | 69 | None |
| Human;Rabbit | 0.016 | 0.0494 | 217 | 285 | 69 | None |
| Gorilla;Rabbit | 0.0201 | 0.05818 | 217 | 285 | 69 | None |
| Marmoset;Dog | 0.0317 | 0.09429 | 241 | 332 | 92 | None |
| Marmoset;Rabbit | 0.0493 | 0.14138 | 226 | 285 | 60 | None |
| **MSH6** |  |  |  |  |  |  |
| Gorilla;Chimpanzee | 0 | 0.00006 | 1066 | 1529 | 464 | None |
| Human;Gorilla | 0 | 0.00007 | 1066 | 1529 | 464 | None |
| Zebrafish;Chicken | 0.0037 | 0.00783 | 532 | 550 | 19 | None |
| Gorilla;Orangutan | 0.0073 | 0.01704 | 2632 | 2885 | 254 | None |
| **SDHB** |  |  |  |  |  |  |
| NA | NA | NA | NA | NA | NA | NA |
| **STK11** |  |  |  |  |  |  |
| Cow;Rat | 0.0009 | 0.00338 | 379 | 446 | 68 | None |
| Cow;Horse | 0.0028 | 0.00893 | 890 | 951 | 62 | None |
| Cow;Pig | 0.0128 | 0.0475 | 895 | 951 | 57 | None |
| **PMS2** |  |  |  |  |  |  |
| NA | NA | NA | NA | NA | NA | NA |
| **MUTYH** |  |  |  |  |  |  |
| NA | NA | NA | NA | NA | NA | NA |
| **VHL** |  |  |  |  |  |  |
| NA | NA | NA | NA | NA | NA | NA |
| **APC** |  |  |  |  |  |  |
| Elephant;Mouse | 0.0003 | 0.00041 | 1045 | 1280 | 236 | None |
| Dog;Rat | 0.0025 | 0.00565 | 994 | 1157 | 164 | None |
| Horse;Mouse | 0.0028 | 0.00724 | 1105 | 1307 | 203 | None |
| Cow;Rat | 0.0032 | 0.0089 | 994 | 1157 | 164 | None |
| Horse;Rat | 0.0052 | 0.01403 | 985 | 1157 | 173 | None |
| Orangutan;Mouse | 0.0085 | 0.02012 | 1045 | 1241 | 197 | None |
| Cow;Mouse | 0.009 | 0.02047 | 1105 | 1280 | 176 | None |
| Human;Mouse | 0.0105 | 0.02385 | 1045 | 1241 | 197 | None |
| Pig;Rat | 0.0108 | 0.02465 | 994 | 1157 | 164 | None |
| Chimpanzee;Mouse | 0.0112 | 0.02552 | 1045 | 1241 | 197 | None |
| Cow;Pig | 0.0118 | 0.02559 | 916 | 1289 | 374 | None |
| Gorilla;Mouse | 0.0123 | 0.0273 | 1045 | 1241 | 197 | None |
| Mouse;Pig | 0.0235 | 0.05456 | 1105 | 1280 | 176 | None |
| **TP53** |  |  |  |  |  |  |
| Cow;Guinea_Pig | 0.0127 | 0.07289 | 927 | 970 | 44 | 317 |
| **MADH4** |  |  |  |  |  |  |
| Rat;Guinea_Pig | 0.0157 | 0.07756 | 682 | 752 | 71 | None |
| **SDHC** |  |  |  |  |  |  |
| Cow;Guinea_Pig | 0.0127 | 0.07289 | 927 | 970 | 44 | None |
| **ATM** |  |  |  |  |  |  |
| Gorilla;Chicken | 0.0026 | 0.00616 | 3541 | 3587 | 47 | None |
| Gorilla;Chimpanzee | 0.0035 | 0.00819 | 3049 | 3793 | 745 | None |
| Chimpanzee;Chicken | 0.0047 | 0.01096 | 3541 | 3587 | 47 | None |
| Human;Chicken | 0.005 | 0.0115 | 3541 | 3587 | 47 | None |
| Orangutan;Chicken | 0.0052 | 0.01222 | 3541 | 3587 | 47 | None |
| Human;Gorilla | 0.0093 | 0.01851 | 4927 | 5641 | 715 | None |
| Rabbit;Chicken | 0.0173 | 0.03145 | 3547 | 3588 | 42 | None |
| **BHD** |  |  |  |  |  |  |
| Cow;Zebrafish | 0.001 | 0.00282 | 236 | 254 | 19 | None |
| Marmoset;Zebrafish | 0.0011 | 0.00313 | 236 | 254 | 19 | None |
| Mouse;Zebrafish | 0.0011 | 0.00313 | 236 | 254 | 19 | None |
| Opossum;Zebrafish | 0.0013 | 0.00365 | 238 | 257 | 20 | None |
| Cow;Mouse | 0.0048 | 0.01657 | 208 | 266 | 59 | None |
| Elephant;Zebrafish | 0.0136 | 0.04138 | 238 | 251 | 14 | None |
| **TSC1** |  |  |  |  |  |  |
| Orangutan;Horse | 0.0242 | 0.06153 | 1037 | 1136 | 100 | None |
| Horse;Marmoset | 0.0474 | 0.12863 | 1030 | 1136 | 107 | None |
| **PTEN** |  |  |  |  |  |  |
| NA | NA | NA | NA | NA | NA | NA |
| **NF1** |  |  |  |  |  |  |
| Cow;Mouse | 0.0218 | 0.05108 | 7390 | 7445 | 56 | None |
| Gorilla;Opossum | 0.0448 | 0.10141 | 7102 | 7148 | 47 | None |
| Opossum;Orangutan | 0.0463 | 0.10479 | 7102 | 7148 | 47 | None |
| Chicken;Mouse | 0.0464 | 0.10541 | 7406 | 7439 | 34 | None |
| Human;Opossum | 0.0472 | 0.10828 | 7102 | 7148 | 47 | None |
| Chimpanzee;Opossum | 0.048 | 0.11007 | 7102 | 7148 | 47 | None |
|  |  |  |  |  |  |  |
